# The interplay between electron transport chain function and iron regulatory factors influences melanin formation in *Cryptococcus neoformans*

**DOI:** 10.1128/msphere.00250-24

**Published:** 2024-04-30

**Authors:** Peng Xue, Eddy Sánchez-León, Guanggan Hu, Christopher W. J. Lee, Braydon Black, Anna Brisland, Haohua Li, Won Hee Jung, James W. Kronstad

**Affiliations:** 1 Michael Smith Laboratories, Department of Microbiology and Immunology, University of British Columbia, Vancouver, British Columbia, Canada; 2 Department of Systems Biotechnology, Chung-Ang University, Anseong, South Korea; University of Guelph, Guelph, Ontario, Canada

**Keywords:** electron transport chain, reactive oxygen species, iron regulation, fungal pathogenesis, melanin formation, RNA-Seq

## Abstract

Mitochondrial functions are critical for the ability of the fungal pathogen *Cryptococcus neoformans* to cause disease. However, mechanistic connections between key functions such as the mitochondrial electron transport chain (ETC) and virulence factor elaboration have yet to be thoroughly characterized. Here, we observed that inhibition of ETC complex III suppressed melanin formation, a major virulence factor. This inhibition was partially overcome by defects in Cir1 or HapX, two transcription factors that regulate iron acquisition and use. In this regard, loss of Cir1 derepresses the expression of laccase genes as a potential mechanism to restore melanin, while HapX may condition melanin formation by controlling oxidative stress. We hypothesize that ETC dysfunction alters redox homeostasis to influence melanin formation. Consistent with this idea, inhibition of growth by hydrogen peroxide was exacerbated in the presence of the melanin substrate L-DOPA. In addition, loss of the mitochondrial chaperone Mrj1, which influences the activity of ETC complex III and reduces ROS accumulation, also partially overcame antimycin A inhibition of melanin. The phenotypic impact of mitochondrial dysfunction was consistent with RNA-Seq analyses of WT cells treated with antimycin A or L-DOPA, or cells lacking Cir1 that revealed influences on transcripts encoding mitochondrial functions (e.g., ETC components and proteins for Fe-S cluster assembly). Overall, these findings reveal mitochondria-nuclear communication *via* ROS and iron regulators to control virulence factor production in *C. neoformans*.

**IMPORTANCE:**

There is a growing appreciation of the importance of mitochondrial functions and iron homeostasis in the ability of fungal pathogens to sense the vertebrate host environment and cause disease. Many mitochondrial functions such as heme and iron-sulfur cluster biosynthesis, and the electron transport chain (ETC), are dependent on iron. Connections between factors that regulate iron homeostasis and mitochondrial activities are known in model yeasts and are emerging for fungal pathogens. In this study, we identified connections between iron regulatory transcription factors (e.g., Cir1 and HapX) and the activity of complex III of the ETC that influence the formation of melanin, a key virulence factor in the pathogenic fungus *Cryptococcus neoformans*. This fungus causes meningoencephalitis in immunocompromised people and is a major threat to the HIV/AIDS population. Thus, understanding how mitochondrial functions influence virulence may support new therapeutic approaches to combat diseases caused by *C. neoformans* and other fungi.

## INTRODUCTION

Mitochondria play a central role in respiratory metabolism for fungal pathogens of plants and animals and support the ability of fungi to sense environmental and host conditions (1–5). The importance of mitochondrial functions in pathogenesis has been demonstrated for a number of fungal pathogens of humans including *Cryptococcus neoformans* and the related species *C. gattii* (1, 6–8). These basidiomycete yeasts have a global impact on human health because of their propensity to cause life-threatening meningoencephalitis in immunocompromised hosts, including the HIV/AIDS population, organ transplant recipients, and patients undergoing chemotherapy (9–11). Key features of these species connected to mitochondrial activities and contributing to the disease include the formation of a polysaccharide capsule, titan cells, and the cell-wall pigment melanin (12–14). These connections are demonstrated, for example, by the reduction in capsule formation upon inhibition of the electron transport chain (ETC) in *C. neoformans* and a fascinating “division of labor” role for mitochondria in *C. gattii* (8, 15). In the latter case, a subset of fungal cells displays a tubular mitochondrial morphology in response to oxidative stress provoked by the host. These cells support the proliferation of other *C. gattii* cells with non-tubular organelle morphology within host phagocytic cells. These results, combined with the role of mitochondria in generating reactive oxygen species (ROS) (16–18), indicate a central role for the organelle in fungal pathogenesis.

A number of additional studies demonstrate the role of mitochondria in virulence factor regulation and the virulence of *C. neoformans* (19–27). For example, a mutation in the promoter of the gene encoding mitochondrial complex I protein NADH dehydrogenase increases capsule and melanin formation (19). In addition, recent work identified the heat shock transcription factor Hsf3 as an important regulator of ROS homeostasis in mitochondria, although mutants lacking the factor displayed only minor attenuation of virulence in mice (22). Other factors that influence mitochondria in *C. neoformans* include the temperature-responsive J domain protein Mrj1 that supports ETC function, influences the shedding of capsule polysaccharide, and impacts cell wall structure (24). A detailed investigation revealed that Mrj1 plays a positive role in maintaining ETC function at the complex III step (24).

During infection, pathogenic fungi use transcription factors to sense iron availability and regulate iron transport and homeostasis, ensuring successful competition for iron to support proliferation *in vivo* (28–30). We previously identified Cir1 as an iron-responsive transcription factor in *C. neoformans* (28). Genetic studies revealed that Cir1 is not only a master regulator of iron homeostasis but also regulates key virulence factors including melanin and capsule. In particular, the *cir1Δ* mutant exhibited markedly reduced infectivity, defective capsule production, and impaired growth at 30°C or 37°C. By contrast, the mutant showed enhanced melanin production, and this regulation is due in part to direct binding of Cir1 to the promoter of the laccase gene *LAC1* and repression of transcription (28, 31). Melanin is deposited in the cell wall and is a major virulence factor for *C. neoformans* that contributes to protection against phagocytosis and oxidative killing by phagocytic cells (12, 13, 32–34). *C. neoformans* synthesizes melanin from exogenous catecholamine substrates such as L-3,4-dihydroxyphenylalanine (L-DOPA) through both laccase-catalyzed reactions and spontaneous oxidation (33).

Cir1 is also a binding partner for the monothiol glutaredoxin Grx4 that participates in iron homeostasis in *C. neoformans* (35). In addition, mutation of the glutaredoxin (GRX) domain of Grx4 results in defective melanin synthesis. Thus, Grx4 and Cir1 provide a key connection between iron homeostasis and melanin formation, but the underlying signaling mechanisms are poorly understood. In other fungi, Grx4 also interacts with the HapX/Php4 component of the CCAAT binding complex (29, 36). This observation is consistent with the participation of HapX in iron homeostasis in *C. neoformans* including repression of iron-dependent functions (e.g., mitochondrial gene expression) upon iron limitation (23, 31). Loss of HapX also results in reduced susceptibility to agents such as H_2_O_2_ and menadione that cause oxidative stress (20). Our previous RNA-Seq and ChIP-Seq experiments revealed that *HAPX* is also a direct target of Cir1 repression under iron-replete conditions (23, 31). Thus, Cir1 and HapX are key iron regulators that control iron homeostasis and mitochondrial functions in *C. neoformans* (37). Furthermore, the pH-responsive transcription factor Rim101 also contributes to iron regulation in this pathogen (38, 39).

In this study, we examined the impact of ETC inhibition on melanin formation and found a balancing influence of ROS and the activities of Cir1 and HapX. We also established the influence of the melanin substrate L-DOPA, inhibition of ETC complex III, or loss of Cir1 on the transcript levels of mitochondrial functions. Thus, mitochondrial functions and the iron regulatory factors appear to influence the proper balance between oxidation and reduction to establish the conditions necessary for melanin formation. In general, the regulation of melanization in fungi is complex (13, 40–42), and our investigation adds new insights into connections between mitochondrial function and virulence factor deployment.

## RESULTS

### Inhibition of ETC complexes I and III impairs melanin formation

**Fig 1:**
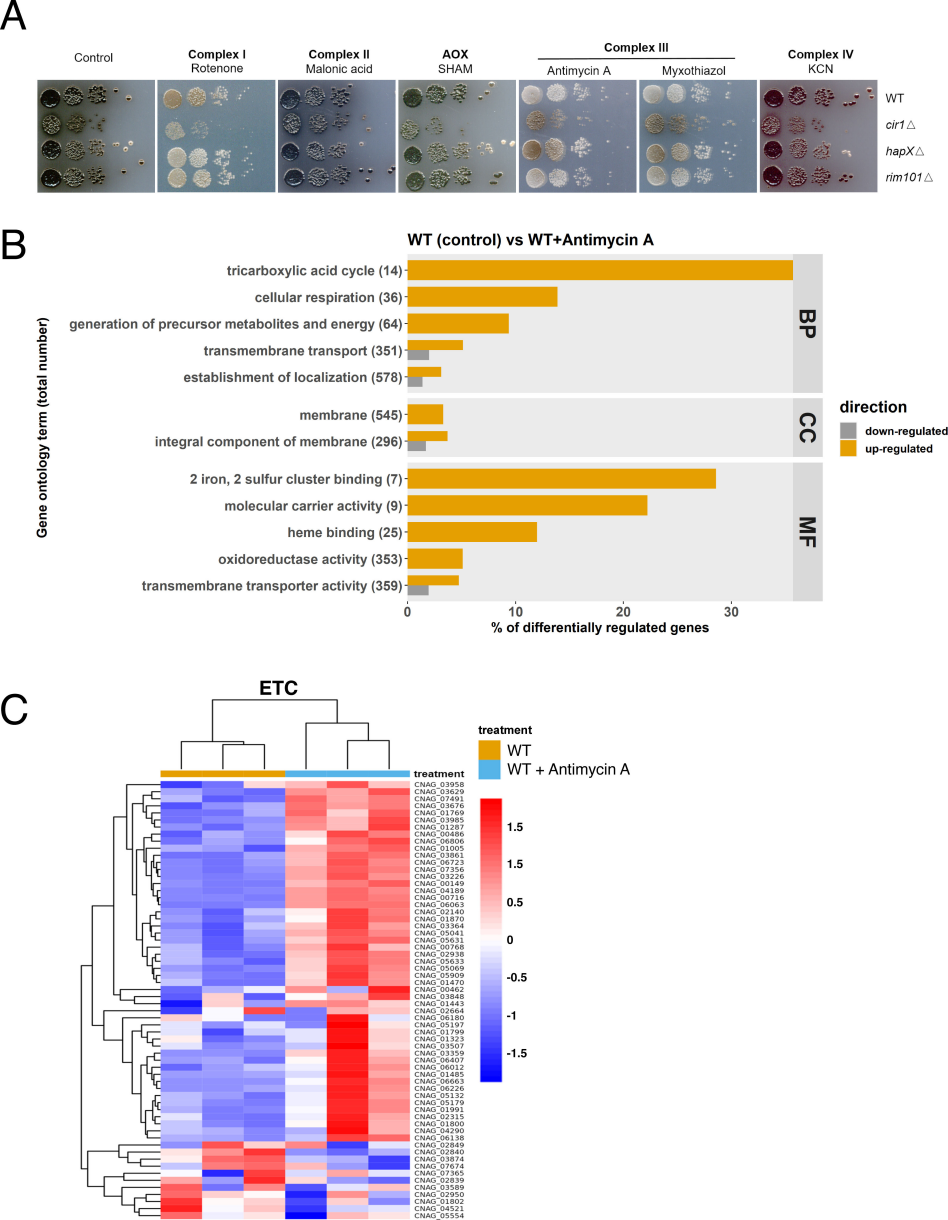
ETC inhibition influences melanin formation and the transcription of genes for mitochondrial functions. (**A**) Spot assays of WT and mutant strains on L-DOPA agar plates with or without inhibitors of the ETC including 1.2 µg/mL rotenone, 100 µM malonic acid, 0.2 mM salicylhydroxamic acid (SHAM), 0.5 µg/mL antimycin A, 0.5 µM myxothiazol, and 100 µM KCN. Plates were incubated for 72 h at 30°C in the dark. (**B**) Gene ontology (GO) categories of the differentially expressed genes identified by RNA-seq analysis of WT cells with and without antimycin A (0.5 µg/mL) treatment. The total number of genes in each functional category is displayed in parentheses, and the percent of all differentially expressed genes is indicated. BP: biological process; CC: cell component; MF: molecular function. (**C**) Heatmap of the expression of genes encoding components of ETC in the WT versus WT with antimycin A treatment. Samples are clustered according to expression similarity, the graphs represent triplicate repeats of the same two conditions, and the differences in transcript levels were significant (*P* < 0.05). The corresponding genes are listed in Table S1.

Given that melanin formation is influenced by a mutation in the gene for mitochondrial NADH dehydrogenase, a component of complex I of the ETC (19), we hypothesized that mitochondrial functions influence laccase expression, trafficking, and/or activity. In particular, inhibition of ETC complexes I and III generates ROS including superoxide anion radical and hydrogen peroxide, and the subsequent influence on intracellular redox conditions may impact melanin formation (16–18, 22, 43). In addition, mitochondria play important roles in metal ion homeostasis, heme biosynthesis, and Fe-S cluster biogenesis that may influence the activity of regulatory factors such as Cir1 which control laccase expression (31, 44). We investigated mitochondrial contributions to melanin formation by examining pigment formation on a medium with L-DOPA and inhibitors of each ETC complex (Fig. 1A). We found that melanin formation in the wild-type (WT) strain and the *cir1Δ* mutant was inhibited by the complex I inhibitor rotenone and that growth of the *cir1Δ* mutant was impaired on rotenone compared with the other strains. The growth of the mutant was also diminished in the presence of malonic acid, SHAM, and KCN although melanin formation was still observed. A more striking reduction in melanin accumulation was observed for the WT strain in the presence of the complex III inhibitors antimycin A or myxothiazol (Fig. 1A). Interestingly, the impact of complex III inhibitors on melanization was partially overcome in the *cir1Δ* and *hapXΔ* deletion mutants (Fig. 1A). In addition, the growth of the *cir1Δ* mutant was not markedly impaired by antimycin A or myxothiazol compared to treatment with rotenone. A mutant lacking the pH-responsive transcription factor Rim101 was included as a control and did not influence melanin formation in the presence of ETC inhibitors. Overall, we conclude that inhibition of ETC complex III activity impairs melanin formation, Cir1 and HapX influence the phenotypic impact of inhibition, and that inhibition of complex I also has an impact on melanin formation.

### Inhibition of ETC complex III impacts transcripts for mitochondrial functions but not laccase genes

To investigate the connection between the ETC and melanin, we focused our subsequent experiments on antimycin A and complex III inhibition by analyzing the transcriptome of the WT strain treated with this inhibitor. In particular, we hypothesized that an influence on the expression of *LAC1* (encoding the major laccase) or regulatory factors for *LAC1* (e.g., Cir1) might account for the melanin defect. Our analysis identified differential transcript levels for 210 genes, with 169 upregulated and 41 downregulated (Fig. S1). Moreover, subsequent analysis of gene ontology (GO) terms revealed upregulation of molecular function categories associated with Fe-S cluster and heme binding, and molecular carrier activity (Fig. 1B). These GO terms prompted an analysis of transcript levels for components of the ETC as well as mitochondrial (ISC) and cytoplasmic (CIA) Fe-S cluster assembly machinery to examine the impact of ETC complex III inhibition. As shown in the heatmaps in Fig. 1C and Fig. S2A, we found that inhibition resulted in elevated transcript levels for ETC components as well as for functions for ISC and CIA machinery in the WT strain. Furthermore, the ETC and mitochondrial ISC assembly pathways, but not the CIA pathway, were found to be significantly enriched through Gene Set Enrichment Analysis (GSEA). Table S1 lists the specific genes represented by the heatmaps and Fig. S3 documents the output from the GSEA. The RNA-Seq analysis revealed a minimal impact of complex III inhibition on the transcript levels of *LAC1* or *CIR1,* thus refuting our hypothesis.

We employed quantitative reverse transcription-PCR (qRT-PCR) to validate the RNA-Seq data. This approach confirmed the transcript levels of the genes CNAG_01881 and CNAG_05199 (ISC pathway genes), *ATM1* (mitochondrial ISC transporter)*,* and CNAG_05840 (CIA pathway gene) in agreement with the RNA-Seq data (Fig. S2B). The transcript levels for the *LAC1* and *CIR1* genes were also confirmed by qRT-PCR (Fig. S2B). A specific examination of the transcript levels for *LAC1, CIR1, LAC2* (a second gene for laccase), and *HAPX* revealed that inhibition of ETC complex III caused only modest reductions in mRNA levels for the *LAC1*, *LAC2,* and *HAPX* genes and a slight increase in mRNA levels for *CIR1* (Fig. S4). As mentioned, our previous results showed that Cir1 directly and negatively regulates *LAC1* and *HAPX* gene transcription (23, 31). Overall, we conclude that inhibition of complex III has a major influence on transcript levels for mitochondrial functions involved in the ETC and Fe-S biogenesis, but only a minor impact on transcript levels for the laccase genes that is likely insufficient to explain the impaired melanin formation.

### Oxidative stress influences melanin formation

**Fig 2:**
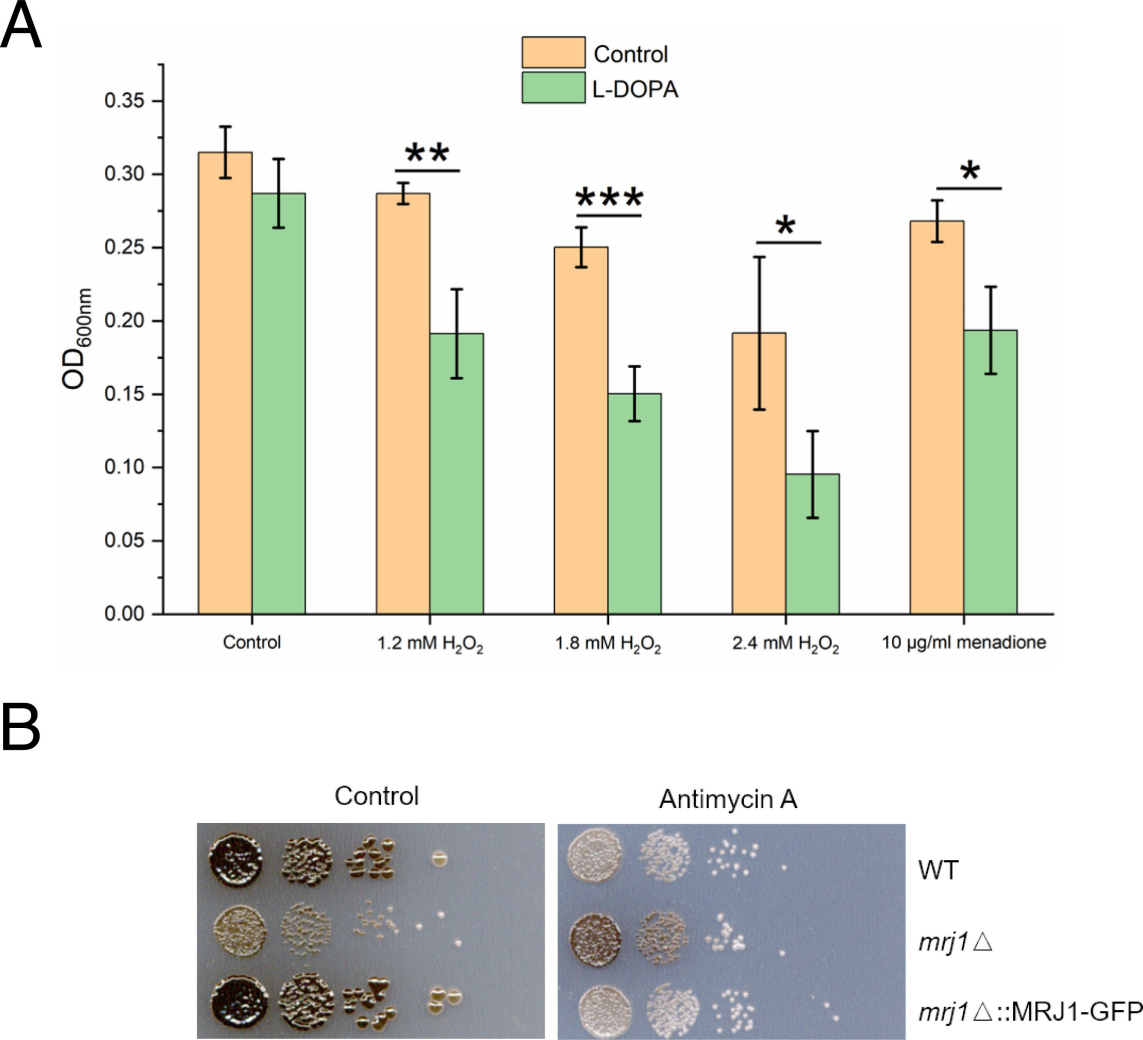
Growth and melanin formation are influenced by L-DOPA, oxidative stress, and the mitochondrial regulator Mrj1. (**A**) The growth of WT cells was tested in a liquid medium with and without 0.7 mM L-DOPA in the presence of hydrogen peroxide (H_2_O_2_) or menadione at the indicated concentrations for 18 h at 30°C and 180 rpm in the dark. The assays were performed using 96-well microplates with each well containing a final volume of 200 µL. The initial cell density was set at 1 × 10^5^ cells/mL. Mean values of three biological replicates are shown ±standard deviation (SD). Significant differences were determined by *t-*tests and are indicated by * (*P* < 0.05), ** (*P* < 0.01), or *** (*P* < 0.005). (**B**) Spot assays of WT, *mrj1Δ,* and *mrj1Δ::MRJ1-GFP* strains showing that the absence of *MRJ1* rescues melanin formation on L-DOPA plates in the presence of antimycin A (0.5 µg/mL). Plates were incubated for 72 h at 30°C in the dark.

Given the minor influence of antimycin A on *LAC1* and *LAC2* gene transcription and the fact that the inhibitor triggers ROS in *C. neoformans* and other organisms (16–18, 22, 43), we hypothesized that antimycin A influenced melanin by establishing redox conditions that interfere with laccase activity. To support this idea, we examined the transcript levels for genes responsive to oxidative stress and found that the transcript levels for the *CAT1* and *CAT3* genes encoding catalases were elevated ~2-fold upon antimycin A treatment (Table 1). The transcript levels for these genes are known to be responsive to H_2_O_2_, among a larger group of oxidative stress genes (45). These observations prompted a closer examination of the impact of ROS during melanin formation and we found that the combination of H_2_O_2_ or menadione with the melanin substrate L-DOPA impaired growth in a liquid medium (Fig. 2A).

**TABLE 1:** Genes encoding proteins for resistance to oxidative stress with transcript levels influenced by antimycin A, L-DOPA or Cir1, as determined by RNA-seq analysis^*b*^

Gene ID	Gene	Function	Melanin conditions			Low iron conditions^*a*^	
			WT vs WT + AA	WT vs WT + L DOPA	WT vs cir1△ ETC	WT vs cir1△ LIM	WT vs hapXΔ LIM
**CNAG_04981**	*CAT1*	Catalase 1	**1.104**	**1.321**	**1.361**	0.905	**2.386**
CNAG_05256	*CAT2*	Catalase 2	nd	**1.187**	**2.228**	**4.037**	**1.714**
**CNAG_00575**	*CAT3*	Catalase 3	**1.177**	**2.312**	1.083	1.740	**5.696**
CNAG_05015	*CAT4*	Catalase 4	nd	nd	**1.894**	**4.636**	**1.852**
CNAG_00654	*SRX1*	Sulfiredoxin	nd	nd	**3.355**	**4.267**	**2.194**
**CNAG_01138**	*CCP1*	Cytochrome c peroxidase	**0.360**	**−2.098**	**3.842**	**116.565**	**45.527**
**CNAG_05847**	*TRR1*	Thioredoxin reductase	**−0.419**	−0.449	**3.385**	**24.919**	0.976
**CNAG_00162**	*AOX1*	Alternative oxidase	**2.595**	nd	**2.126**	**12.621**	**5.664**
CNAG_04219	*GLX1*	Lactoylglutathione lyase	**0.277**	**−0.419**	**0.765**	**0.607**	**6.632**

^*a*^ The transcript levels for these genes were characterized previously (31). The transcript levels for the gene IDs indicated in bold are responsive to H_2_O_2_ and were characterized by Upadhya et al. (45). nd, not detected.

^*b*^ The log2 fold changes (experimental group/control group) in bold are significantly different (*P <* 0.001).

The connection with oxidative stress and redox homeostasis was examined further using a mutant known to influence the ETC at complex III and to have reduced ROS levels. Specifically, we examined a mutant lacking the *MRJ1* gene encoding mitochondrial respiration J-domain protein 1 that influences the activity of ETC complex III by directly binding to Qcr2, a subunit of ubiquinol cytochrome c reductase (24). We found that the effect of complex III inhibition on melanin formation was partially ablated in the *mrj1Δ* mutant, a result consistent with the influence of Mrj1 on ROS accumulation (Fig. 2B). These results further support the idea that melanin formation is conditioned at least in part through a balance in redox homeostasis.

### L-DOPA negatively impacts the transcription of mitochondrial functions and genes encoding catalases

**Fig 3:**
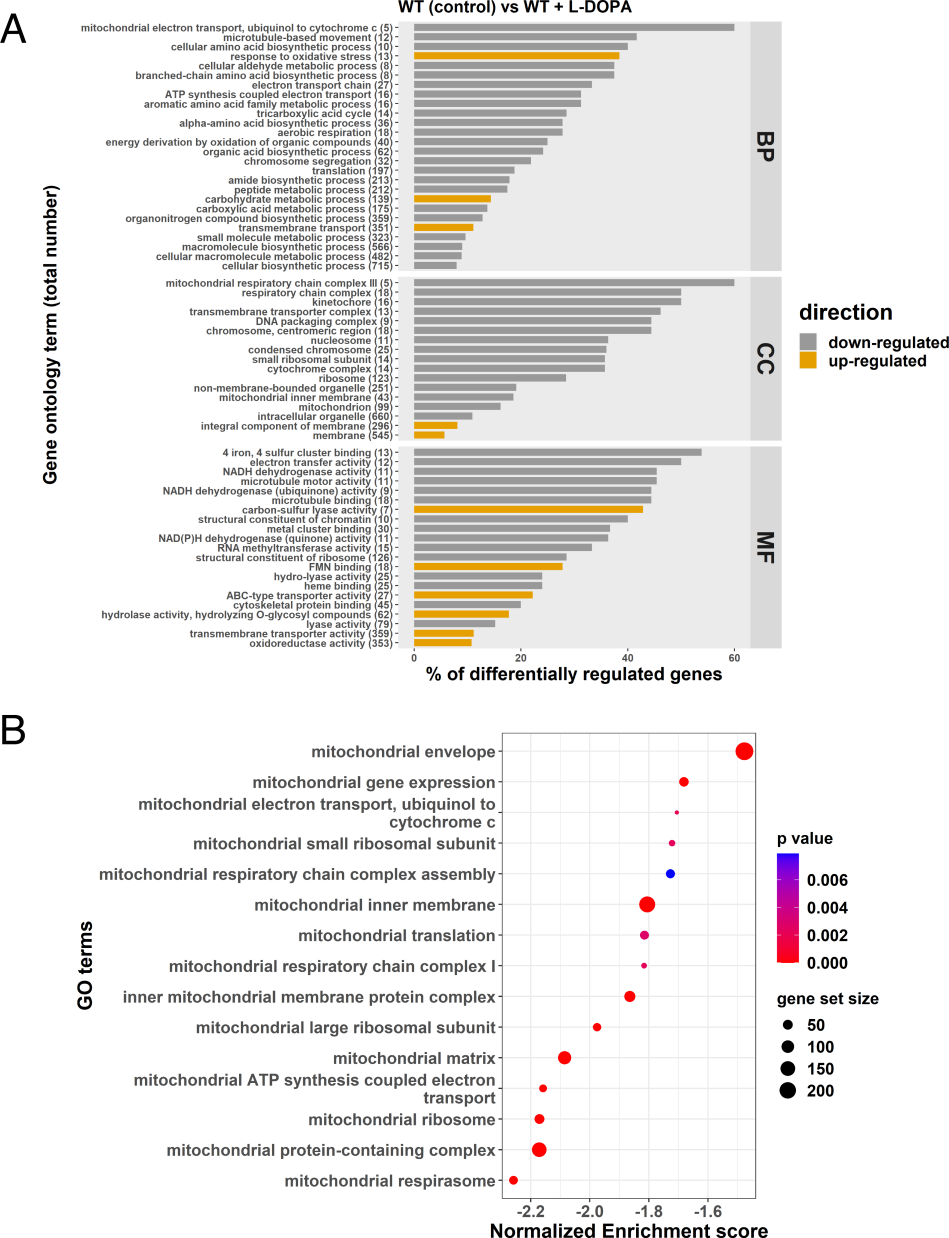
L-DOPA provokes downregulation of mitochondrial functions. (**A**) GO terms for genes with differential transcription upon treatment with L-DOPA (0.7 mM). The total number of genes in each functional category is displayed in parentheses, and the percent of all differentially expressed genes is indicated on the x-axis. BP: biological process; CC: cell component; MF: molecular function. (**B**) Gene Set Enrichment Analysis demonstrating negative enrichment for pathways directly involved in mitochondrial function. Significantly regulated pathways were determined using a 0.05 cut-off *P*-value and a false discovery rate of 0.25. The list of regulated genes for components of the ETC and iron-sulfur cluster biogenesis is presented in Table S2.

Given that the combination of L-DOPA and H_2_O_2_ impaired growth, we hypothesized that L-DOPA treatment might also influence oxidative stress and mitochondrial functions. We therefore examined the impact of L-DOPA by performing RNA-Seq to compare the transcript profiles for WT cells in the presence and absence of L-DOPA. We found that L-DOPA treatment resulted in differential transcript levels for 1,262 genes, with 836 upregulated and 426 downregulated (Fig. S5). Analysis of the GO categories for the differentially transcribed genes indicated a negative impact of L-DOPA treatment on functions associated with mitochondria (Fig. 3A). This observation was further supported by GSEA using pathways from the KEGG database and homologous gene IDs based on the genome of the *C. neoformans* strain JEC21. This analysis revealed that 15 KEGG terms for mitochondrial functions, including ETC components, were negatively enriched upon growth with L-DOPA (Fig. 3B). The differential regulation of representative genes encoding ETC components (e.g., genes CNAG_01287, CNAG_05041, and CNAG_07491) was confirmed by qRT-PCR (Fig. S6). We also noted the transcript levels of genes encoding Cir1, HapX, or Rim101 were not differentially expressed in the RNA-Seq analysis of the response to L-DOPA, and only a minor influence was observed by qRT-PCR (Fig. S6). A summary of the differential transcript levels for mitochondrial functions is presented in Table S2. A specific examination of the genes for oxidative stress (Table 1) also identified the *CAT1, CAT2,* and *CAT3* transcripts as being significantly elevated upon treatment with L-DOPA. Together, these results revealed that conditions supporting melanin formation (i.e., the presence of L-DOPA) have an impact on the expression of mitochondrial functions as well as oxidative stress. These results reinforce the idea that mitochondria are responsive to environmental signals, including L-DOPA, and control ROS generation to establish conducive redox conditions that influence virulence factor (melanin) production.

### Cir1 influences melanin formation through the de-repression of *LAC1* transcription and an influence of mitochondrial gene expression

**Fig 4:**
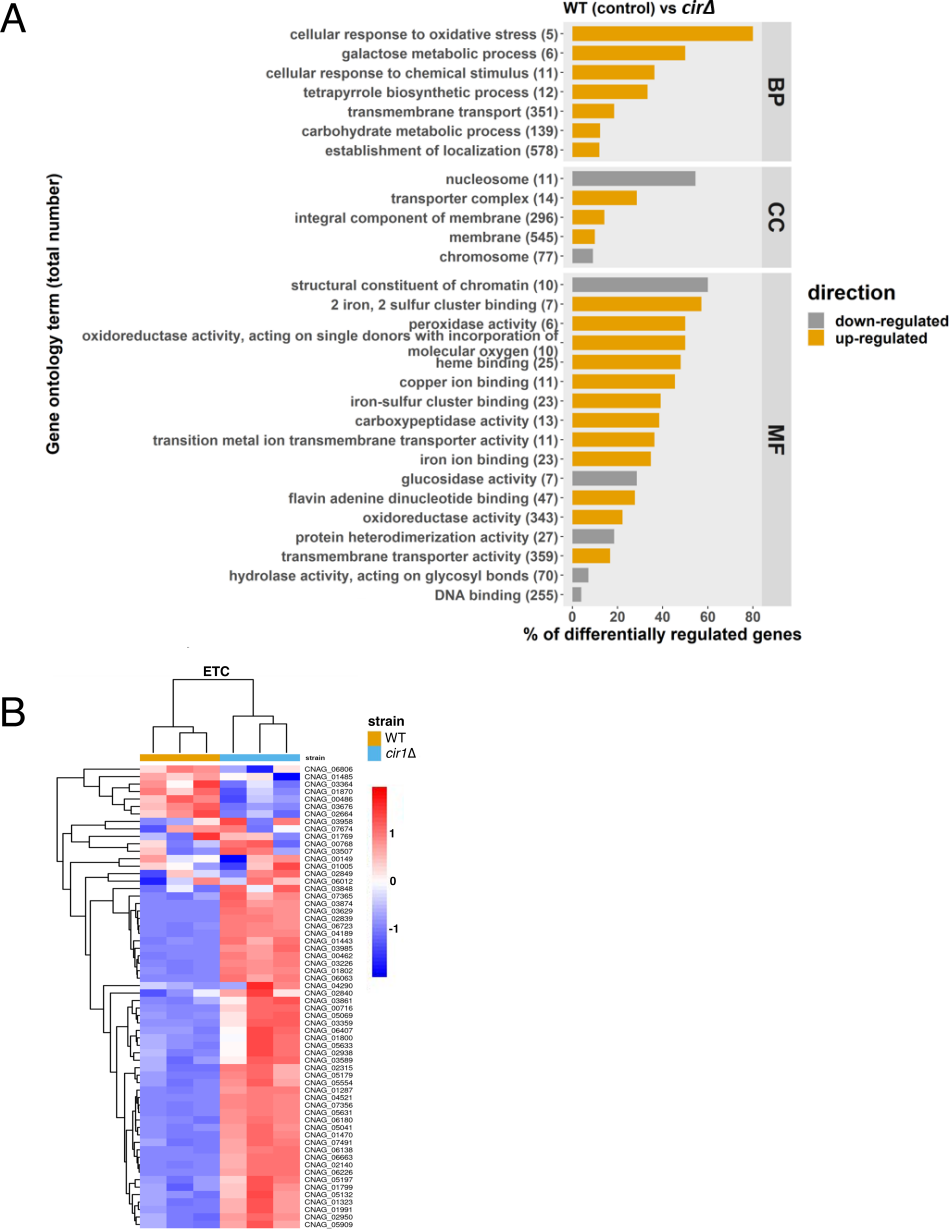
Loss of Cir1 results in the upregulation of mitochondrial functions. (**A**) GO terms for RNA-Seq analysis of the WT strain vs the *cir1*Δ mutant. The total number of genes in each functional category is displayed in parentheses, and the percent of all differentially expressed genes is indicated. BP: biological process; CC: cell component; MF: molecular function. (**B**) Heatmap of the expression of genes encoding components of ETC in the WT and *cir1*Δ mutant strains. The differences in transcript levels were significant (*P* < 0.05), and the corresponding genes are listed in Table S3.

As shown in Fig. 1A, complex III inhibitors impaired melanin formation in the WT strain, and the *cir1Δ* and *hapXΔ* mutants partially restored melanin. Our previous microarray and RNA-Seq experiments with *cir1Δ* mutant demonstrated that Cir1 represses *LAC1* transcription (23, 31). Furthermore, ChiP-seq analysis revealed that Cir1 directly binds the promoter of the *LAC1* gene to repress transcription (31). These results suggest that de-repression upon loss of Cir1 during antimycin A treatment may contribute to restored melanin formation. We therefore examined the influence of Cir1 on *LAC1* transcription, ETC function, and oxidative stress genes by performing an RNA-Seq experiment with WT and the *cir1Δ* mutant under the minimal medium conditions used for the analysis of the impact of antimycin A on the transcriptome (Materials and Methods). We identified 853 differentially expressed genes (710 upregulated and 143 downregulated) and an overview of the analysis is shown in Fig. S7. As with previous transcriptome studies with Cir1 (23, 28, 31), an examination of the GO terms for molecular functions that were upregulated in the *cir1Δ* mutant revealed categories that included oxidoreductase activity (acting on peroxide as acceptor, and acting on single donors with incorporation of molecular oxygen), Fe-S cluster, copper ion binding, heme binding, antioxidant activity, transition metal iron transmembrane transporter activity, and iron ion binding (Fig. 4). For example, the transcript levels of mitochondrial ISC assembly machinery components were significantly upregulated in the *cir1Δ* mutant compared to the WT, and we note that there are many Fe-S cluster binding proteins in complexes I, II, and III of the ETC. In addition, genes encoding oxidative stress functions including the catalase genes were upregulated in the *cir1Δ* mutant (Table 1). Heatmaps of the regulation of transcripts for ETC components and mitochondrial ISC assembly pathway components in the *cir1Δ* mutant as compared to the WT strain are shown in Fig. 4B and Fig. S8A; the corresponding genes are listed in Table S3, and Fig. S9 presents the GSEA analysis of the ETC and mitochondrial ISC pathways. Overall, these data confirm the influence of Cir1 on mitochondrial functions—an effect that may impact susceptibility to complex III inhibitors such as antimycin A through enhanced expression of specific organelle transcripts.

To confirm the RNA-Seq data, the transcript levels of the ISC pathway gene CNAG_03395, the ETC genes CNAG_06063, CNAG_07491 and CNAG_02839, and *LAC1* were examined by qRT-PCR. We found comparable patterns of regulation between the RNA-Seq and qRT-PCR results (Fig. S8B). Consistent with previous analyses of the regulatory influence of Cir1 (23, 28, 31), we verified the differences between the WT strain and the *cir1Δ* mutant for the transcript levels of the laccase genes *LAC1* and *LAC2* and confirmed the regulatory influence of Cir1 on the transcription of the genes using qRT-PCR (Fig. S8C). Overall, these results suggest that loss of Cir1 leads to de-repression of *LAC1* to influence melanin in the presence of complex III inhibitors. As mentioned above, an additional influence on the expression of mitochondrial and oxidative stress functions may also condition susceptibility to ETC inhibition.

### Cir1 and HapX respond differently to oxidative stress

**Fig 5:**
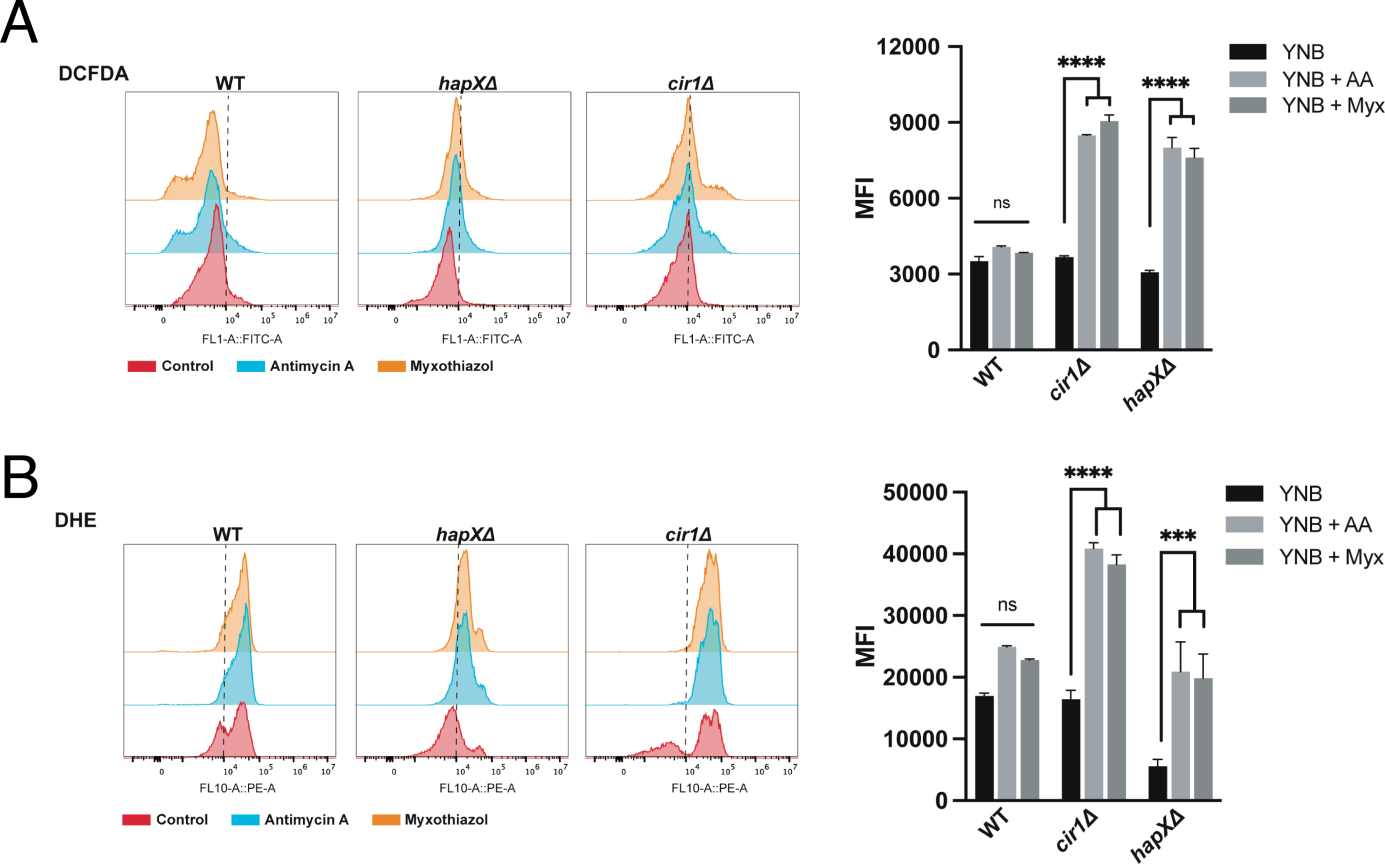
Loss of Cir1 or HapX results in the accumulation of ROS in response to complex III inhibition. (**A**) Flow cytometry analysis of WT and mutant cells stained for 1 h with 2′,7′-dichlorofluorescein diacetate (DCFDA, 16 µM) to detect ROS accumulation in response to exposure to antimycin A (ΑA, 50 µM) or myxothiazol (Myx, 7 µM) for 24 h at 30°C. (**B**) Flow cytometry analysis of WT and mutant cells stained with dihydroethidium (DHE, 2.5 µg/mL) to detect ROS accumulation in response to ETC-III inhibitors as in (**A**). The data represent the mean fluorescent intensity (MFI, geometric means) from three biological replicates ±standard errors of the means. The statistical comparisons employed a two-way ANOVA test, followed by *post hoc* Šídák’s or Tukey’s multiple comparison tests (*P < 0.05, **P < 0.01 ***P < 0.001; ****P < 0.0001). ns: not significant. The gating strategy for the left panels is shown in Fig. S11.

**Fig 6:**
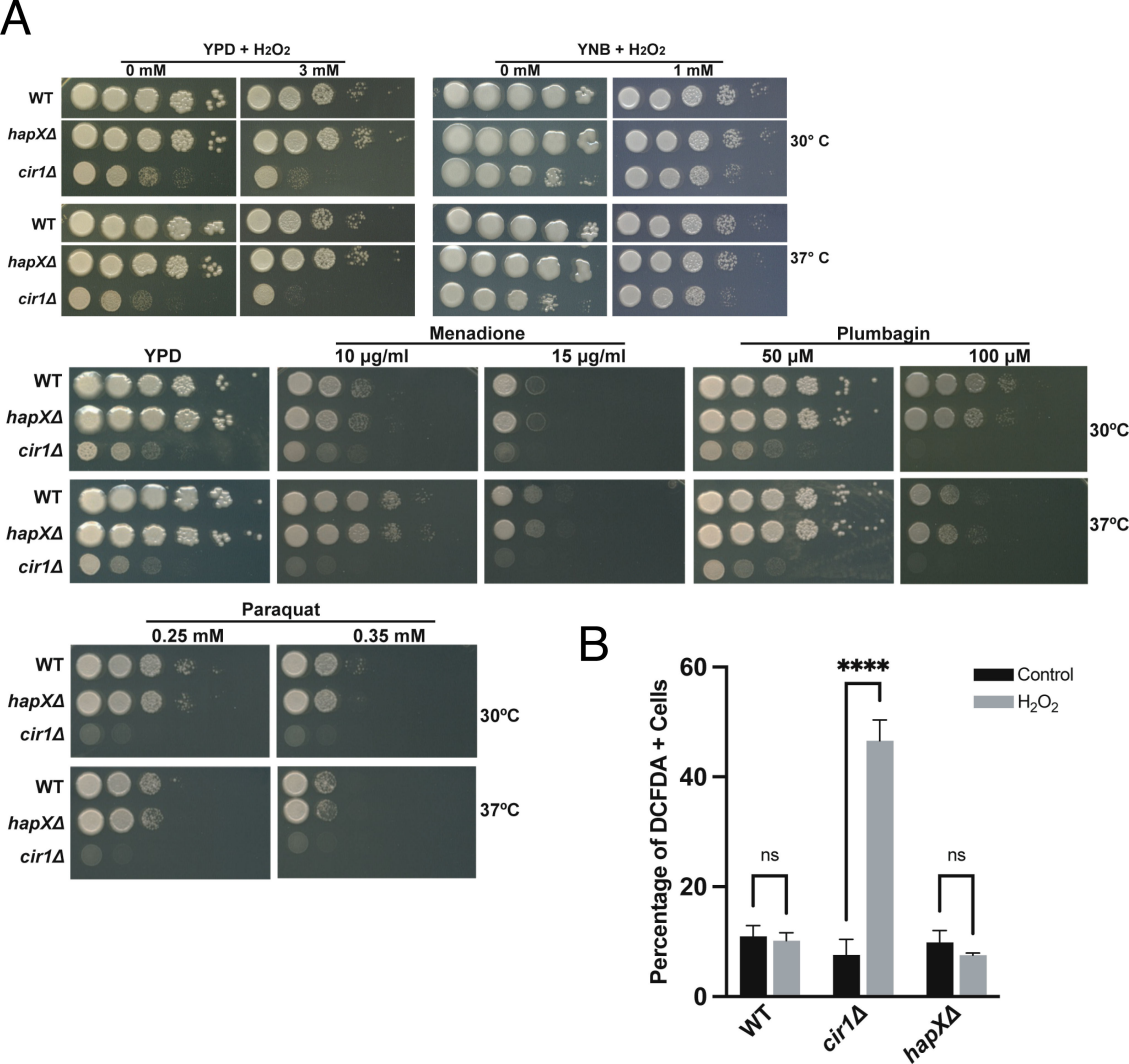
Loss of Cir1 but not HapX causes sensitivity to oxidative stress. (**A**) Spot assays of the WT strain and the *hapX*Δ or *cir1*Δ mutants on the indicated media were performed with 10-fold serial dilutions from an initial concentration of 2 × 10^7^ cells per mL. Five microliters were spotted into solid YPD or YNB plates supplemented with different compounds and incubated at 30°C and 37°C for 2–3 days before being scanned. The media were supplemented at the indicated concentrations with the following compounds: hydrogen peroxide (H_2_O_2_), menadione, plumbagin, or paraquat. (**B**) Graph of the percentage of DCFDA-positive cells upon exposure to H_2_O_2_ [5 mM] for 1 h at 30°C for the WT strain and the *hapX*Δ and *cir1*Δ mutants as determined by flow cytometry using the gating strategy in Fig. S11. The data represent the average from at least three biological replicates ±standard errors of the means. Statistical comparisons employed a two-way ANOVA test, followed by *post hoc* Šídák’s multiple comparison test (****P < 0.0001). ns: not significant.

In contrast to the regulation of *LAC1* expression by Cir1, previous experiments revealed that HapX does not regulate laccase expression (23, 31). This observation suggests a different mechanism for melanin recovery upon antimycin A treatment, and we hypothesized that an influence of HapX on oxidative stress and the redox environment impacts melanin formation. We therefore examined oxidative stress in more detail by measuring ROS accumulation in the WT and *cir1Δ* and *hapXΔ* mutant strains in response to antimycin A and myxothiazol treatment. Both mutants showed higher levels of DCFDA staining compared to the WT strain indicating an accumulation of ROS (Fig. 5A). The actions of multiple types of ROS (e.g., hydroxyl radicals, H_2_O_2_) can oxidize DCFDA to generate a fluorescent signal (46). By contrast, staining with dihydroethidium (DHE), which is responsive to superoxide accumulation, revealed greater levels in the *cir1Δ* mutant and lower endogenous levels/accumulation in the *hapXΔ* mutant. The differences between the mutants prompted an examination of the sensitivities of each strain to oxidative stress caused by H_2_O_2_, menadione, plumbagin, and paraquat (Fig. 6A). This experiment revealed greater sensitivity for the *cir1Δ* mutant compared to the WT strain and the *hapXΔ* mutant. Consistent with this differential sensitivity, we found that treatment with H_2_O_2_ provoked enhanced DCFDA straining in the *cir1Δ* mutant but not in the WT strain or the *hapXΔ* mutant (Fig. 6B). These observations are consistent with our previous finding that the *hapXΔ* mutant behaves mainly like WT with regard to sensitivity to oxidative stress (20). HapX is known to control the expression of functions for the response to oxidative stress, including the *CAT1* and *CAT3* catalase genes influenced by antimycin A and L-DOPA (Table 1) (23, 31). We confirmed this regulation by qRT-PCR for a set of the oxidative stress genes from Table 1 (Fig. S10). Overall, these results suggest that antimycin A-induced inhibition of melanin results from changes to the redox environment that are unfavorable for melanin synthesis. Cir1 and particularly HapX appear to modulate this influence by regulating the expression of antioxidant functions, along with the de-repression of laccase expression upon loss of Cir1.

## DISCUSSION

Melanin deposition in the cell wall of *C. neoformans* confers protection against various stresses (e.g., oxidative killing by phagocytic cells) and reduces susceptibility to antifungal drugs (2, 3, 13, 41). These properties are relevant to disease because mutants with defects in melanin formation show reduced virulence in mouse models, and melanin influences dissemination to the brain and aspects of the immune response including phagocytosis (13, 41). In this study, we made the following discoveries regarding the regulation of melanin. First, we found that inhibition of ETC complex III activity caused a defect in melanin production on the substrate L-DOPA. This finding was bolstered by the discovery that the *mrj1*Δ mutant, which lacks an interacting partner of the complex III component Qcr2 and has reduced ROS accumulation (24), also influences melanin formation in the presence of complex III inhibition. These results are consistent with the known influence of antimycin A on ROS accumulation (22), and with our new finding that the melanin substrate L-DOPA is inhibitory for growth in the presence of H_2_O_2_. Second, our analysis revealed that defects in each of the major iron regulatory factors in *C. neoformans*, Cir1 and HapX, partially overcame the defect in melanin formation caused by inhibition of complex III. Our mechanistic analysis revealed that Cir1 and HapX influenced the observed phenotypes by different mechanisms, thus illustrating the complex interplay between iron regulation, the ETC, and the elaboration of melanin. Specifically, we found that loss of Cir1 depressed laccase gene expression and also influenced the transcription of genes for mitochondrial functions, while loss of HapX regulated the response to oxidative stress and ROS accumulation. The observations on Cir1 and HapX are consistent with previous studies of these factors in C. *neoformans,* findings with orthologs in other fungi, and studies on mitochondrial biogenesis (23, 28, 31, 47, 48). Finally, the experiments are bolstered by novel transcriptome experiments on the impact of antimycin A or L-DOPA treatment, or loss of Cir1. These experiments revealed that the underlying transcriptional contributions to the observed melanin phenotypes including the novel finding that L-DOPA influences the expression of mitochondrial functions. The associated transcriptome data will be valuable for further studies of *C. neoformans* interactions with host-relevant substrates for melanin formation and the research community.

Along with the interplay between iron regulators and the ETC, the connection between melanin, L-DOPA, and oxidative stress, as demonstrated by the influence of H_2_O_2_ on growth in combination with L-DOPA, is a key finding of our study. Melanin formation by *C. neoformans* is responsive to a variety of signals including glucose levels, temperature, cell density, and metal ions (calcium, copper, iron) (49–52). In addition, a previous study on the transcriptional response of *C. neoformans* revealed that H_2_O_2_ treatment decreased the expression of the *LAC1* gene and influenced the transcript levels for a set of genes encoding functions for oxidative stress resistance (45). Together, these observations support the idea that a balanced level of ROS, as influenced by mitochondrial functions, is needed to support melanin formation. Our analysis of cells exposed to L-DOPA revealed a substantial impact on the transcriptome including the downregulation of mitochondrial functions. This impact is interesting because laccase activity and melanin may contribute to the neurotropism of *C. neoformans* (49, 53). The possibility that L-DOPA is a signal as well as a substrate is intriguing because our observations of an influence on mitochondria draw parallels with studies on the role of dopamine in neurons and in neurodegenerative diseases (54, 55). For example, an examination of the potential toxicity of L-DOPA in the treatment of Parkinson’s disease revealed an impact on mitochondrial and lysosomal activities (56).

A previous study examined the transcriptional response of *C. neoformans* to L-DOPA using microarray analysis and, in contrast to our observations, found differential expression of only eight genes (57). Some of these genes encoded functions predicted to be involved in the response to stress and, importantly, this result is consistent with our observed categories of GO terms and our demonstration that cells are more sensitive to H_2_O_2_ in the presence of L-DOPA. The differences in the number of regulated genes could be due to several factors including the use of microarray versus RNA-Seq approaches (and associated differences in evaluating differential expression), the use of different strains (JEC21 versus H99), different preculture conditions, and the timing and level of L-DOPA exposure. However, four of the JEC21 genes had regulated orthologs in the RNA-Seq data for H99. Interestingly, regulation of five of the genes was not observed in a laccase mutant suggesting that the formation of melanin may have a regulatory influence (40, 57).

**Fig 7:**
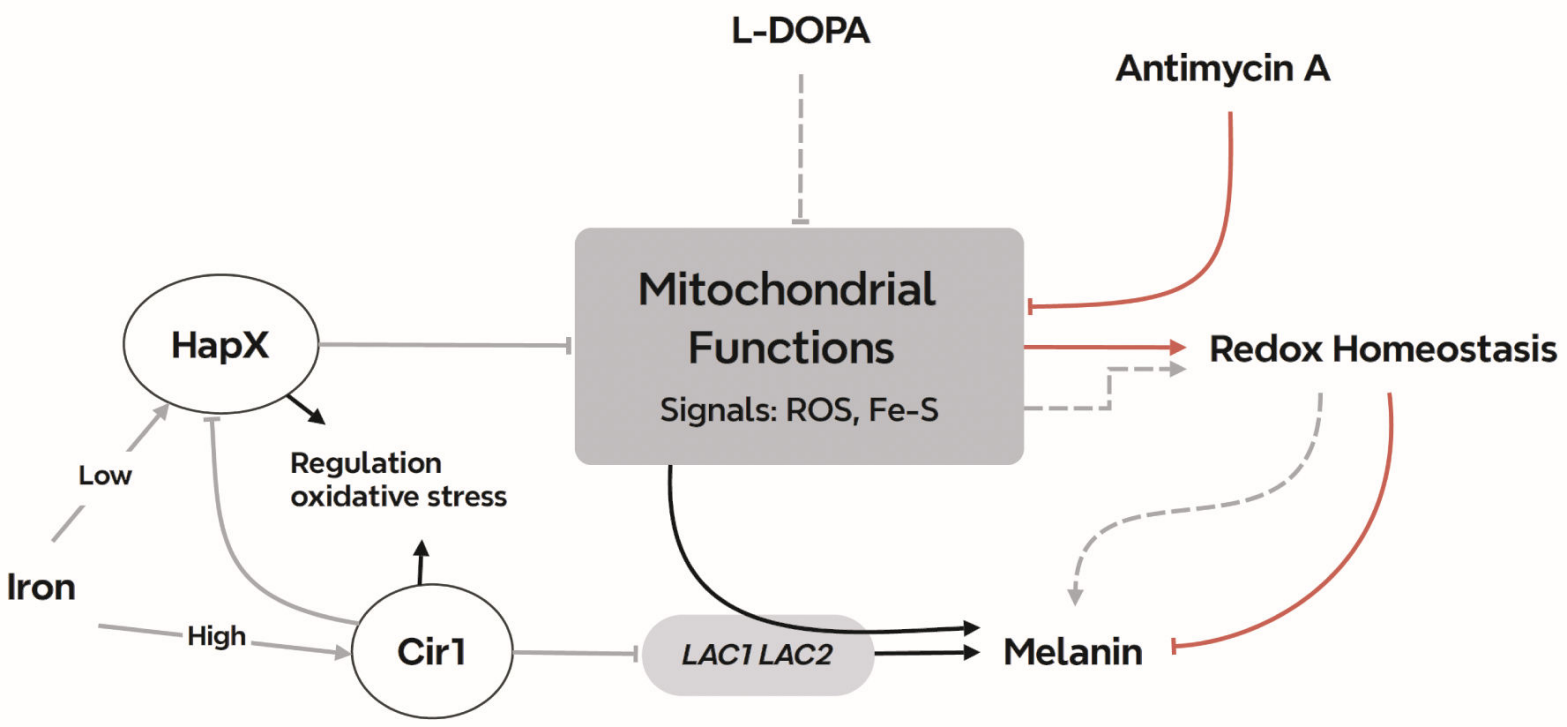
Summary model of the interplay between mitochondrial ETC function and the iron regulators that influence melanin formation in *C. neoformans*. Inhibitors of ETC complex III provoke ROS accumulation which inhibits melanin formation through an influence on laccase activity and/or localization. Cir1 directly represses transcription of the *LAC1* and *LAC2* genes encoding laccases, and loss of Cir1 may derepress the genes to a level sufficient to partially restore melanin formation. Cir1 also represses the transcription of the *HAPX* gene (23, 31). HapX is a key regulator of iron-requiring functions in mitochondria, and the loss of HapX derepresses genes for the response to oxidative stress. This de-repression may be sufficient to overcome ROS accumulation to partially restore melanin. Other signals including iron-sulfur clusters may be generated by ETC inhibition to influence the activities of the iron regulators and the laccases.

In summary, we have established that impaired ETC complex III activity influences the elaboration of melanin in *C. neoformans* and that iron regulatory factors buffer this influence. These observations reinforce the emerging view that mitochondrial activity is important for fungi to resist host defenses and to cause disease, and are consistent with an impact of iron levels on the size of the polysaccharide capsule and the expression of laccase (58–60). An overview of the regulatory pattern uncovered by our study is presented in Fig. 7. Additional work is needed to fully understand the connections between ROS generated by mitochondrial activities and the activity and/or localization of laccase to support melanization. Furthermore, the involvement of other proteins in the iron regulatory network that influence melanin formation, such as the monothiol glutaredoxin Grx4, should be investigated (61). Finally, the discovery that L-DOPA treatment impacts the expression of mitochondrial functions warrants a focused investigation into a potential signaling role for this important substrate.

## MATERIALS AND METHODS

### Strains, growth conditions, and spot assays with inhibitors

The following strains were used in this study: WT strain *Cryptococcus neoformans* var. *grubii* H99, deletion mutants *cir1*Δ (28), *hapX*Δ (23), *rim101*Δ (62, 63), *atm1*Δ (26), and *mrj1Δ* (16) (24), and complemented mutants *cir1*Δ::*CIR1* (28), *hapX*Δ::*HAPX* (23), *rim101*Δ::*RIM101* (62, 63), *atm1*Δ::*ATM1-GFP* (26), and *mrj1*Δ::*MRJ1-GFP* (24). Routine growth was in YPD or YNB liquid or solid media.

Melanin production was examined in a medium with L-DOPA (0.1% glucose, 0.1% L-asparagine, 0.3% KH_2_PO_4_, 0.7 mM L-DOPA, 0.025% MgSO4 · 7H_2_O, and pH 5.6). Agar was added at 2% for solid medium. For spot assays, cells from overnight cultures at 30°C were washed twice with liquid YPD medium, density was adjusted to 1 × 10^5^ cells m^L−1^, and 10-fold serial dilutions were prepared. Next, 5 µL was spotted on the 2% L-DOPA agar plates with or without the ETC inhibitors at the indicated concentrations. Plates were incubated in the dark for 72 h at 30°C, and photographed to document melanin formation.

To examine *C. neoformans* WT (H99) or mutant strains, cells were grown in YPD or YNB for ~16 h at 30°C. Precultures were washed and resuspended in H_2_O, and 10-fold serial dilutions were performed from an initial concentration of 2 × 10^7^ cells per milliliter. Five microliters was spotted into solid YPD or YNB plates supplemented with different compounds and incubated at 30°C and 37°C for 2–3 days before being scanned. For the reactive oxygen stress response, rich (YPD) and minimal (YNB) media were supplemented with the following compounds: hydrogen peroxide solution (H_2_O_2_, 1–5 mM), menadione (10, 15 µg/mL), plumbagin (50, 100 µM), and paraquat (0.25, 0.35 mM).

### RNA extraction, RNA sequencing and analysis, and quantitative reverse transcription-PCR

To examine the impact of complex III inhibition on the transcriptome, the WT strain and the *cir1Δ* and *hapXΔ* mutants were grown in 50 mL of YPD at 30°C for 16 h. The cells were washed twice with sterile water and then incubated in 50 mL of YNB with 0.06% glucose for 16 h at 30°C. The cells were then diluted to 4.0 × 10^7^ cells in 50 mL of YNB medium (2% glucose) with or without 0.5 µg/mL antimycin A, followed by a 6-h incubation at 30°C. For the analysis of the influence of L-DOPA on the transcriptome, three replicate cultures of WT cells were grown in 50 mL of YPD (2% glucose) at 30°C for 16 h. Cells were then washed twice with sterile water, and diluted to 4.0 × 10^7^ cells in 50 mL of melanin induction medium with or without 0.7 mM L-DOPA. After incubation for 6 h at 30°C, the cells were harvested and flash-frozen in liquid nitrogen. The total RNA from frozen cells was extracted using an RNeasy-Mini kit (QIAGEN), followed by treatment with DNase I (TURBO DNA-free Kit). RNA-Seq was performed by Genewiz (Azenta Life Sciences, South Plainfield, NJ, USA).

Differentially expressed genes were analyzed in R (version 4.3.2) using the raw gene counts provided by Genewiz and the DEseq2 package. All genes were assessed for significance using DEseq2’s inherent Wald test (*P* value cut-off = 0.05) and normalized between samples using the median of ratios method (64). The identification of enriched pathways was performed by two independent approaches. First, to identify significantly enriched functional groups, the RNA-Seq data were analyzed with respect to KEGG database annotations. For compatibility with the KEGG database, FungiDB was used to convert gene IDs from strain H99 to strain JEC21. Based on both a ranked gene list (Ranked score = −(log2FoldChange) × log10 (*P* value)) derived from the DESeq2 output and the KEGG/PATHWAY database, the identification of enriched pathways was performed by Gene Set Enrichment Analysis (GSEA) with 10,000 permutations (65). The output was visualized using an Enrichment Map with a Benjamini Hochberg FDR value of 0.25, and a *P*-value cut-off of 0.05 for all comparisons. Gene sets between 10 and 400 were included (66). Gene sets were visualized in heatmaps of the normalized expression generated using R (67) and the DittoSeq package (68). Clustering was performed to organize samples and genes of similar expression (68). Second, we also analyzed sets of over-represented pathways for enrichment in protein functions. *De novo* GO term assignments of predicted proteins were performed by InterProScan 5.26–65 (69). We kept the GO terms with a total term size in the genome of at least five. To test for enrichment, we performed hypergeometric tests. GO terms were only considered significant if the FDR *P*-values were less than <0.001. The R packages GSEABase (70) and GOstats (71) were used to analyze all enrichments, and the R package ggplot2 (72) was used to visualize the outcomes of enrichment tests. Gene expression in the samples for RNA-Seq was verified by qRT-PCR following published procedures (73). The primers for qPCR validation are listed in Table S4.

### RNA extraction and qPCR to analyze oxidative stress

Overnight grown wild type (H99) and *hapXΔ* cells (2.5 mL) in YPD media were washed thrice with low iron water and incubated in 50 mL of YNB media supplemented with BPS (150 µM) for 24 h at 30°C and 140–150 rpm. Subsequently, cultures were collected and washed twice with YNB-BPS, and 500 µL of cell resuspensions were treated in YNB-BPS for 1 h at 30°C and ~220 rpm. Cells were harvested and washed with ice-cold low-iron water and pellets were frozen in liquid nitrogen and kept at −80°C. Total RNA was extracted from lysed cells using the RNeasy Mini kit (Qiagen) and DNA was removed using Turbo DNA-*free* Kit (Invitrogen). Synthesis of cDNA was obtained using the High-Capacity cDNA Reverse Transcription Kit (Applied Biosystems ) using oligo (dT). Real-time PCR (qPCR) was performed using the primers listed in Table S5, the cDNA and Green-2-Go qPCR Mastermix-low Rox (BioBasic) as described by the manufacturer. Samples were run on an Applied Biosystems 7500 Fast real-time PCR System. Relative gene expression was quantified using the ΔCT method normalized to the *ACT1* gene. Statistical analysis was performed using a two-way ANOVA test followed by a *post hoc* Šídák’s multiple comparison test.

### Flow cytometry analysis

Flow cytometric measurements were performed using CytoFLEX S (Beckman Coulter) Flow Cytometer equipped with four laser lines (405, 488, 561, and 633 nm) fitted with filters FITC (525/40) and PE (585/42). The number of cells measured per experiment was set to 30,000–40,000 unless otherwise stated. For the study of mitochondrial-related ROS under the influence of ETC III inhibition, cells were grown overnight at ~200 rpm and 30°C in YPD media and washed twice with PBS. Subsequently, 0.3 OD cells were grown on minimal media (YNB) for 24 h and then treated in YNB media with and without the ETC III inhibitors antimycin A (ΑA, 50 µM) or myxothiazol (Myx, 7 µM) for 24 h at 200 rpm and 30°C. After treatment, cells were stained with the intracellular ROS detector 2ʹ,7ʹ-dichlorofluorescein diacetate (DCFDA, 16 µM; Sigma-Aldrich) or dihydroethidium (DHE, 2.5 µg/mL; EMD Millipore Corp.) for 1 h at 30°C. For the analysis of ROS stressors, cells were grown on YPD media overnight as mentioned above. Next, cells were grown in YNB media and collected on log phase to subsequently treat them with or without hydrogen peroxide solution (H_2_O_2_, 5 mM; Sigma-Aldrich) for 1 h at 30°C. Afterward, cells were washed with PBS and stained with DCFDA as mentioned above. Data analysis and evaluation were conducted using FlowJo software version (10.8.2; 2006–2022). The gating strategy for cryptococcus cells is depicted in Fig. S11. Statistical analysis involved conducting a two-way ANOVA test, followed by *post hoc* Šídák’s or Tukey’s multiple comparison tests. GraphPad Prism software was utilized for the statistical analysis.

## Data Availability

The RNA-Seq data have been deposited in Gene Expression Omnibus record GSE222564. The full gene lists for the RNA-Seq data for each condition are given in Tables S6 to S8.

## SUPPLEMENTAL MATERIAL

Supplemental Figures msphere.00250-24-s0001.pdf Figures S1 to S11.

Supplemental Tables msphere.00250-24-s0002.pdf Tables S1-S8.

ASM does not own the copyrights to Supplemental Material that may be linked to, or accessed through, an article. The authors have granted ASM a non-exclusive, world-wide license to publish the Supplemental Material files. Please contact the corresponding author directly for reuse.
