# Supplementary material for: The interplay between electron transport chain function and iron regulatory factors influences melanin formation in Cryptococcus neoformans
Source: mSphere. 2024 Apr 30;9(5):e00250-24. doi: 10.1128/msphere.00250-24 (PMC11237718; doi:10.1128/msphere.00250-24)
Supplement: Supplemental Tables — Tables S1-S8. [file msphere.00250-24-s0002.pdf]

TABLE S1 The effect of antimycin A on the gene expression of components of the mitochondrial ISC assembly pathway, the CIA pathway and the ETC (KEGG) for the WT strain. The log2 fold change (experimental group/control group) in bold was significantly different (*P* < 0.05).

Gene ID	Gene name	Function	WT (control) vs WT + antimycin A
**Mitochondrial ISC assembly pathway**			
CNAG_00389	*IBA57*	Mitochondrial protein	0.04
CNAG_01442	*NFS1*	Cysteine desulferase	**-0.183**
CNAG_04039	*FRR3*	Scaffold protein	-0.001
CNAG_05011	*FRR4*	Frataxin	0.031
CNAG_02131	*ISA1*	Iron sulfur assembly protein 1	**0.328**
CNAG_00491	*ISA2*	Iron sulfur assembly protein 2	**0.304**
CNAG_03985	*GRX5*	Monothiol glutaredoxin-5	**0.297**
CNAG_07446	*ISD11*	Mitochondrial protein	**0.249**
CNAG_03395	*NFU1*	NifU-like protein C	**0.329**
CNAG_05035	*ARH1*	Ferredoxin-NADP reductase	**0.472**
CNAG_05199	*SSQ1*	HSP70-type chaperone	**0.943**
CNAG_01881	*MGE1*	Nucleotide exchange factor co-chaperone	**1.048**
CNAG_03352	*IND1*	Hypothetical protein	**0.2**
CNAG_04288	*JAC1*	J-domain co-chaperone	**0.218**
**CIA pathway**			
CNAG_01802	*DRE2*	Cytoplasmic protein	-0.102
CNAG_04202	*NAR1*	Iron hydrogenase	0.134
CNAG_01839	*TAH18*	Peptidyl-prolyl cis-trans isomerase NIMA- interacting 1	**0.154**
CNAG_02467	*MMS19*	DNA repair/transcription protein	**0.142**
CNAG_05840	*CIA2*	Hypothetical protein	**0.379**
CNAG_00085	*CIA1*	Histone chaperone	0.133
CNAG_00082	*CFD1*	Cytosolic Fe-S cluster assembly factor	0.086
CNAG_02231	*NBP35*	Cytosolic Fe-S cluster assembly factor NBP35	0.036
**Mitochondrial ETC**			
CNAG_03958		Hypothetical protein	**0.251**
CNAG_03629	*YHB1*	NADH dehydrogenase (quinone), G subunit	**0.159**
CNAG_07491		Glutaredoxin	**0.769**
CNAG_03676		D-lactate dehydrogenase	**0.226**
CNAG_01769		Solute carrier family 25 (mitochondrial aspartate/glutamate transporter), member 12/13	**0.396**

CNAG_03985	*GRX5*	Grx4 family monothiol glutaredoxin	**0.297**
CNAG_01287		NADH-ubiquinone oxidoreductase 51 kDa subunit, mitochondrial	**0.216**
CNAG_00486		Succinate dehydrogenase assembly factor 2, mitochondrial	**0.430**
CNAG_06806	*ETF1*	Electron transfer flavoprotein alpha subunit	**0.204**
CNAG_01005		Glutaredoxin	**0.256**
CNAG_03861		Cytochrome c oxidase subunit 5a	**0.391**
CNAG_06723		Succinate dehydrogenase (ubiquinone) membrane anchor subunit	**0.647**
CNAG_07356		Succinate dehydrogenase, cytochrome b556 subunit	**0.679**
CNAG_03226		Succinate dehydrogenase [ubiquinone] iron-sulfur subunit, mitochondrial	**1.076**
CNAG_00149		NADH dehydrogenase (ubiquinone) 1 alpha subcomplex 4	**0.783**
CNAG_04189	*SDH1*	Succinate dehydrogenase [ubiquinone] flavoprotein subunit, mitochondrial	**1.060**
CNAG_00716	*CYC7*	Cytochrome c	**0.670**
CNAG_06063		Cytochrome c oxidase assembly protein subunit 15	**1.407**
CNAG_02140		NADH dehydrogenase (ubiquinone) 1 alpha subcomplex 12	**0.133**
CNAG_01870		Electron transfer flavoprotein beta subunit	**0.333**
CNAG_03364		Hypothetical protein	0.162
CNAG_05041		NADH-ubiquinone oxidoreductase subunit 8	**0.302**
CNAG_05631		NADH-ubiquinone oxidoreductase 49 kDa subunit, mitochondrial	**0.187**
CNAG_00768		Hypothetical protein	**0.296**
CNAG_02938		Cytochrome c oxidase subunit 6a	**0.290**
CNAG_05633		Ubiquinol-cytochrome c reductase subunit 9	**0.341**
CNAG_05069		Ubiquinol-cytochrome c reductase subunit 10	**0.510**
CNAG_05909	*CYT1*	Cytochrome c1, heme protein	**0.440**
CNAG_01470		NADH dehydrogenase (ubiquinone) flavoprotein 2	**0.255**
CNAG_00462		Electron-transferring-flavoprotein dehydrogenase	**0.169**
CNAG_03848		Glutathione transferase	0.253
CNAG_01443		Hypothetical protein	0.138
CNAG_02664		D-lactate dehydrogenase	0.003
CNAG_06180		NADH dehydrogenase (ubiquinone) Fe-S protein 4	0.050
CNAG_05197		NADH dehydrogenase (ubiquinone) 1 alpha subcomplex 8	0.078
CNAG_01799		Ubiquinol-cytochrome c reductase subunit 8	**0.162**

CNAG_01323	*QCR7*	Ubiquinol-cytochrome c reductase subunit 7	**0.169**
CNAG_03507		Mitochondrial-processing peptidase subunit beta	**0.234**
CNAG_03359		Cytochrome c oxidase subunit 7	**0.526**
CNAG_06407		Cytochrome c oxidase subunit 7c	**0.343**
CNAG_06012		NADH-cytochrome b5 reductase 1	**0.217**
CNAG_01485		D-lactate dehydrogenase (cytochrome)	**0.159**
CNAG_06663		Ubiquinol-cytochrome c reductase subunit 6	**0.283**
CNAG_06226		NADH dehydrogenase (ubiquinone) 1 alpha subcomplex 5	**0.251**
CNAG_05132		Cytochrome c oxidase subunit 5b	**0.314**
CNAG_05179	*QCR2*	ubiquinol-cytochrome c reductase core subunit 2	**0.215**
CNAG_01991		Cytochrome c oxidase subunit 4	**0.269**
CNAG_02315		Ubiquinol-cytochrome c reductase, iron-sulfur subunit	**0.284**
CNAG_01800		Hypothetical protein	**0.306**
CNAG_04290		Hypothetical protein	0.132
CNAG_06138		NADH dehydrogenase (ubiquinone) Fe-S protein 6	**0.206**
CNAG_02849		Glutathione transferase	-0.283
CNAG_02840		Hypothetical protein	**-0.564**
CNAG_03874		Oxidoreductase	**-0.190**
CNAG_07674		Hypothetical protein	-0.168
CNAG_07365		Oxidoreductase	0.011
CNAG_02839		Hypothetical protein	-0.300
CNAG_03589		Adrenodoxin-type ferredoxin	-0.035
CNAG_02950	*GRX3*	Grx4 family monothiol glutaredoxin	-0.120
CNAG_01802		Fe-S cluster assembly protein	-0.102
CNAG_04521		Oxidoreductase	**-0.504**
CNAG_05554		Cytochrome b5	-0.088

TABLE S2 The impact of L-DOPA on gene expression for components of the mitochondrial ISC assembly pathway, the CIA pathway and the ETC (KEGG) in the WT strain. The log2 fold change (experimental group/control group) in bold was significantly different ($P < 0.05$).

Gene ID	Gene name	Function	WT (control) vs WT + L-DOPA
**Mitochondrial ISC assembly pathway**			
CNAG_00389	*IBA57*	Mitochondrial protein	**-0.468**
CNAG_01442	*NFS1*	Cysteine desulferase	0.202
CNAG_04039	*FRR3*	Scaffold protein	**0.459**

CNAG_05011	*FRR4*	Frataxin	**-0.527**
CNAG_02131	*ISA1*	Iron sulfur assembly protein 1	**-0.655**
CNAG_00491	*ISA2*	Iron sulfur assembly protein 2	-0.166
CNAG_03985	*GRX5*	Monothiol glutaredoxin-5	-0.310
CNAG_07446	*ISD11*	Mitochondrial protein	0.290
CNAG_03395	*NFU1*	NifU-like protein C	**-0.830**
CNAG_05035	*ARH1*	Ferredoxin-NADP reductase	**-0.564**
CNAG_05199	*SSQ1*	HSP70-type chaperone	**0.624**
CNAG_01881	*MGE1*	Nucleotide exchange factor co-chaperone	0.257
CNAG_03352	*IND1*	Hypothetical protein	-0.150
CNAG_04288	*JAC1*	J-domain co-chaperone	**1.069**

CIA pathway

CNAG_01802	*DRE2*	Cytoplasmic protein	-0.171
CNAG_04202	*NAR1*	Iron hydrogenase	-0.080
CNAG_01839	*TAH18*	Peptidyl-prolyl cis-trans isomerase NIMA- interacting 1	-0.013
CNAG_02467	*MMS19*	DNA repair/transcription protein	0.006
CNAG_05840	*CIA2*	Hypothetical protein	**-0.326**
CNAG_00085	*CIA1*	Histone chaperone	**-0.433**
CNAG_00082	*CFD1*	Cytosolic Fe-S cluster assembly factor	**0.327**
CNAG_02231	*NBP35*	Cytosolic Fe-S cluster assembly factor NBP35	0.148

Mitochondrial ETC

CNAG_03958		Hypothetical protein	**1.346**
CNAG_03629	*YHB1*	NADH dehydrogenase (quinone), G subunit	**-1.33**
CNAG_07491		Glutaredoxin	**0.940**
CNAG_03676		D-lactate dehydrogenase	**-0.363**
CNAG_01769		Solute carrier family 25 (mitochondrial aspartate/glutamate transporter), member 12/13	-0.168
CNAG_03985	*GRX5*	Grx4 family monothiol glutaredoxin	-0.310
CNAG_01287		NADH-ubiquinone oxidoreductase 51 kDa subunit, mitochondrial	**-1.255**
CNAG_00486		Succinate dehydrogenase assembly factor 2, mitochondrial	**-0.631**
CNAG_06806	*ETF1*	Electron transfer flavoprotein alpha subunit	0.209
CNAG_01005		Glutaredoxin	0.236
CNAG_03861		Cytochrome c oxidase subunit 5a	**-0.816**
CNAG_06723		Succinate dehydrogenase (ubiquinone) membrane anchor subunit	**-1.582**

CNAG_07356		Succinate dehydrogenase, cytochrome b556 subunit	**-1.458**
CNAG_03226		Succinate dehydrogenase [ubiquinone] iron-sulfur subunit, mitochondrial	**-1.694**
CNAG_00149		NADH dehydrogenase (ubiquinone) 1 alpha subcomplex 4	**-1.171**
CNAG_04189	*SDH1*	Succinate dehydrogenase [ubiquinone] flavoprotein subunit, mitochondrial	**-1.467**
CNAG_00716	*CYC7*	Cytochrome c	**-1.678**
CNAG_06063		Cytochrome c oxidase assembly protein subunit 15	**-0.649**
CNAG_02140		NADH dehydrogenase (ubiquinone) 1 alpha subcomplex 12	**-1.258**
CNAG_01870		Electron transfer flavoprotein beta subunit	-0.488
CNAG_03364		Hypothetical protein	**-0.454**
CNAG_05041		NADH-ubiquinone oxidoreductase subunit 8	**-1.309**
CNAG_05631		NADH-ubiquinone oxidoreductase 49 kDa subunit, mitochondrial	**-1.105**
CNAG_00768		Hypothetical protein	0.161
CNAG_02938		Cytochrome c oxidase subunit 6a	**-0.666**
CNAG_05633		Ubiquinol-cytochrome c reductase subunit 9	**-0.929**
CNAG_05069		Ubiquinol-cytochrome c reductase subunit 10	**-1.142**
CNAG_05909	*CYT1*	Cytochrome c1, heme protein	**-1.476**
CNAG_01470		NADH dehydrogenase (ubiquinone) flavoprotein 2	**-1.564**
CNAG_00462		Electron-transferring-flavoprotein dehydrogenase	**-1.866**
CNAG_03848		Glutathione transferase	**-1.548**
CNAG_01443		Hypothetical protein	0.197
CNAG_02664		D-lactate dehydrogenase	**-0.709**
CNAG_06180		NADH dehydrogenase (ubiquinone) Fe-S protein 4	**-1.356**
CNAG_05197		NADH dehydrogenase (ubiquinone) 1 alpha subcomplex 8	**-0.969**
CNAG_01799		Ubiquinol-cytochrome c reductase subunit 8	**-0.747**
CNAG_01323	*QCR7*	Ubiquinol-cytochrome c reductase subunit 7	**-1.119**
CNAG_03507		Mitochondrial-processing peptidase subunit beta	**-0.974**
CNAG_03359		Cytochrome c oxidase subunit 7	**-0.900**
CNAG_06407		Cytochrome c oxidase subunit 7c	**-0.851**
CNAG_06012		NADH-cytochrome b5 reductase 1	**-0.591**
CNAG_01485		D-lactate dehydrogenase (cytochrome)	-0.062
CNAG_06663		Ubiquinol-cytochrome c reductase subunit 6	**-1.391**
CNAG_06226		NADH dehydrogenase (ubiquinone) 1 alpha subcomplex 5	**-2.017**

CNAG_05132		Cytochrome c oxidase subunit 5b	**-1.190**
CNAG_05179	*QCR2*	ubiquinol-cytochrome c reductase core subunit 2	**-1.073**
CNAG_01991		Cytochrome c oxidase subunit 4	**-0.865**
CNAG_02315		Ubiquinol-cytochrome c reductase, iron-sulfur subunit	**-1.132**
CNAG_01800		Hypothetical protein	**-1.634**
CNAG_04290		Hypothetical protein	**-0.612**
CNAG_06138		NADH dehydrogenase (ubiquinone) Fe-S protein 6	**-1.248**
CNAG_02849		Glutathione transferase	**-3.585**
CNAG_02840		Hypothetical protein	**1.781**
CNAG_03874		Oxidoreductase	0.255
CNAG_07674		Hypothetical protein	0.345
CNAG_07365		Oxidoreductase	**0.284**
CNAG_02839		Hypothetical protein	**4.074**
CNAG_03589		Adrenodoxin-type ferredoxin	**-0.548**
CNAG_02950	*GRX3*	Grx4 family monothiol glutaredoxin	-0.402
CNAG_01802		Fe-S cluster assembly protein	-0.171
CNAG_04521		Oxidoreductase	**1.373**
CNAG_05554		Cytochrome b5	**-0.962**

TABLE S3 The loss of *CIR1* gene affects the gene transcript levels of components of the mitochondrial ISC assembly pathway and the ETC (KEGG). The log2 fold change (experimental group/control group) in bold was significantly different ($P < 0.05$).

**Gene ID**	**Gene name**	**Function**	**WT (control) vs *cir1*Δ**
**Mitochondrial ISC assembly pathway**			
CNAG_00491	*ISA2*	Iron sulfur assembly protein 2	-0.157
CNAG_05011	*FRR4*	Frataxin	**-0.207**
CNAG_07446	*ISD11*	Mitochondrial protein	**-0.168**
CNAG_04288	*JAC1*	J-domain co-chaperone	**-0.323**
CNAG_05035	*ARH1*	Ferredoxin-NADP reductase	0.013
CNAG_01442	*NFS1*	Cysteine desulferase	**0.169**
CNAG_02131	*ISA1*	Iron sulfur assembly protein 1	**1.504**
CNAG_05199	*SSQ1*	HSP70-type chaperone	**0.569**
CNAG_01881	*MGE1*	Nucleotide exchange factor co-chaperone	**0.477**
CNAG_03352	*IND1*	Hypothetical protein	**0.391**
CNAG_04039	*FRR3*	Scaffold protein	**0.804**
CNAG_00389	*IBA57*	Mitochondrial protein	**0.236**
CNAG_03985	*GRX5*	Monothiol glutaredoxin-5	**2.146**

CNAG_03395	*NFU1*	NifU-like protein C	**2.176**
**Mitochondrial ETC**			
CNAG_06806	*ETF1*	Electron transfer flavoprotein alpha subunit	-0.09
CNAG_01485		D-lactate dehydrogenase (cytochrome)	-0.047
CNAG_03364		Hypothetical protein	-0.11
CNAG_01870		Electron transfer flavoprotein beta subunit	**-0.237**
CNAG_00486		Succinate dehydrogenase assembly factor 2, mitochondrial	**-0.375**
CNAG_03676		D-lactate dehydrogenase	**-0.231**
CNAG_02664		D-lactate dehydrogenase	**-0.275**
CNAG_03958		Hypothetical protein	0.24
CNAG_07674		Hypothetical protein	-0.008
CNAG_01769		Solute carrier family 25 (mitochondrial aspartate/glutamate transporter), member 12/13	0.002
CNAG_00768		Hypothetical protein	0.083
CNAG_03507		Mitochondrial-processing peptidase subunit beta	0.104
CNAG_00149		NADH dehydrogenase (ubiquinone) 1 alpha subcomplex 4	-0.042
CNAG_01005		Glutaredoxin	0.023
CNAG_02849		Glutathione transferase	0.352
CNAG_06012		NADH-cytochrome b5 reductase 1	0.026
CNAG_03848		Glutathione transferase	**0.423**
CNAG_07365		Oxidoreductase	**0.615**
CNAG_03874		Oxidoreductase	**2.534**
CNAG_03629	*YHB1*	NADH dehydrogenase (quinone), G subunit	**0.852**
CNAG_02839		Hypothetical protein	**5.304**
CNAG_06723		Succinate dehydrogenase (ubiquinone) membrane anchor subunit	**1.283**
CNAG_04189	*SDH1*	Succinate dehydrogenase [ubiquinone] flavoprotein subunit, mitochondrial	**1.287**
CNAG_01443		Hypothetical protein	**1.197**
CNAG_03985	*GRX5*	Grx4 family monothiol glutaredoxin	**2.146**
CNAG_00462		Electron-transferring-flavoprotein dehydrogenase	**1.597**
CNAG_03226		Succinate dehydrogenase [ubiquinone] iron-sulfur subunit, mitochondrial	**1.57**
CNAG_01802		Fe-S cluster assembly protein	**1.429**
CNAG_06063		Cytochrome c oxidase assembly protein subunit 15	**1.916**
CNAG_04290		Hypothetical protein	0.131
CNAG_02840		Hypothetical protein	**0.39**
CNAG_03861		Cytochrome c oxidase subunit 5a	**0.355**
CNAG_00716	*CYC7*	Cytochrome c	**1.1**

CNAG_05069		Ubiquinol-cytochrome c reductase subunit 10	**0.835**
CNAG_03359		Cytochrome c oxidase subunit 7	**0.716**
CNAG_06407		Cytochrome c oxidase subunit 7c	**0.53**
CNAG_01800		Hypothetical protein	**0.635**
CNAG_05633		Ubiquinol-cytochrome c reductase subunit 9	**0.6**
CNAG_02938		Cytochrome c oxidase subunit 6a	**0.354**
CNAG_03589		Adrenodoxin-type ferredoxin	**0.365**
CNAG_02315		Ubiquinol-cytochrome c reductase, iron-sulfur subunit	**0.572**
CNAG_05197		NADH dehydrogenase (ubiquinone) 1 alpha subcomplex 8	**0.404**
CNAG_05554		Cytochrome b5	**0.669**
CNAG_01287		NADH-ubiquinone oxidoreductase 51 kDa subunit, mitochondrial	**0.781**
CNAG_04521		Oxidoreductase	**5.652**
CNAG_07356		Succinate dehydrogenase, cytochrome b556 subunit	**1.207**
CNAG_05631		NADH-ubiquinone oxidoreductase 49 kDa subunit, mitochondrial	**0.863**
CNAG_06180		NADH dehydrogenase (ubiquinone) Fe-S protein 4	**1.064**
CNAG_05041		NADH-ubiquinone oxidoreductase subunit 8	**0.983**
CNAG_01470		NADH dehydrogenase (ubiquinone) flavoprotein 2	**1.077**
CNAG_07491		Glutaredoxin	**1.279**
CNAG_06138		NADH dehydrogenase (ubiquinone) Fe-S protein 6	**0.937**
CNAG_06663		Ubiquinol-cytochrome c reductase subunit 6	**0.63**
CNAG_02140		NADH dehydrogenase (ubiquinone) 1 alpha subcomplex 12	**0.975**
CNAG_06226		NADH dehydrogenase (ubiquinone) 1 alpha subcomplex 5	**1.053**
CNAG_05179	*QCR2*	ubiquinol-cytochrome c reductase core subunit 2	**0.464**
CNAG_01799		Ubiquinol-cytochrome c reductase subunit 8	**0.415**
CNAG_05132		Cytochrome c oxidase subunit 5b	**0.463**
CNAG_01323	*QCR7*	Ubiquinol-cytochrome c reductase subunit 7	**0.682**
CNAG_01991		Cytochrome c oxidase subunit 4	**0.468**
CNAG_02950	*GRX3*	Grx4 family monothiol glutaredoxin	**0.399**
CNAG_05909	*CYT1*	Cytochrome c1, heme protein,	**0.461**

Table S4. Primers for qPCR validation of RNA-seq data

**Name**	**Sequence 5'-3'**	**Reference**
Cir1_F	ctcagcaaggcgaaaagtct	This Study

Cir1_R	tcctaacgtcaaaactccacaa	This Study
HapX_F	cccctacgccaattcctatc	This Study
HapX _R	cgttgcctcgtccaataact	This Study
Rim101_F	gctgatcaagagttggcttgt	This Study
Rim101_R	ccacttcaatcctctccctct	This Study
Lac1_F	agggaaagttggctgtggtt	This Study
Lac1_R	tcgcgcagatactggaaagt	This Study
CNAG_01881_F	aaaacttgaagggcgaacag	This Study
CNAG_01881_R	ggcctcaagaccctagaacc	This Study
CNAG_05840_F	gagggctttacctccacgat	This Study
CNAG_05840_R	aacagccttccaccacactc	This Study
CNAG_03395_F	tttggatgaggaagaacttatcg	This Study
CNAG_03395_R	cctttccagacttccctgct	This Study
CNAG_01287_F	caacgacttgctcaattcca	This Study
CNAG_01287_R	aattcgcctaatggcatctc	This Study
CNAG_05041_F	caaaaccaggaattctctaccg	This Study
CNAG_05041_R	tgaaggttagcagcgatttc	This Study
CNAG_07491_F	tgaagaaatgggtgctttcc	This Study
CNAG_07491_R	tgatctccgaaccttttttcg	This Study
CNAG_01769_F	gttgtccacaggccagttct	This Study
CNAG_01769_R	taagcgatttggggataagc	This Study
CNAG_03848_F	gagataatggcaatgcacga	This Study
CNAG_03848_R	tcggcttgttcgtcaccta	This Study
CNAG_02849_F	attgagcttgagacgaaaggag	This Study
CNAG_02849_R	ggcgtcttcaatccgttg	This Study
CNAG_06063_F	gaagattacccttccctgctg	This Study
CNAG_06063_R	tcctactgggcttcctcaaa	This Study
CNAG_02839_F	atgctcaccttgccttggt	This Study
CNAG_02839_R	ttggacaaatacacatcgtcaa	This Study
CNAG_04521_F	ctcgcgaggaacattgtagc	This Study
CNAG_04521_R	tgtaggaaggctcagtgtcg	This Study
CNAG_05199_F	gctcagcttgacgccact	This Study
CNAG_05199_R	tggaaaagaccgagagaagc	This Study

Table S5. Primers for qPCR analysis of genes involved in the response to oxidative stress.

Function	Name	Sequence 5’-3’	Reference
cytochrome c peroxidase	CCP1_F	CCGTCGGAGCTGCATATTAC	This Study
cytochrome c peroxidase	CCP1_R	TCGTAACCCTCCTTGTCGAG	This Study
catalase 3	CAT3_F	CAAGGGTTTGGCGTTCATAC	This Study
catalase 3	CAT3_R	GGTCTCTTCGGTGGAAATCA	This Study
lactoylglutathione lyase (Glyoxalate I)	GLX1_F	GATGCCAATTTCAAGGGCTA	This Study
lactoylglutathione lyase (Glyoxalate I)	GLX1_R	ACTCTCATCCTGCCCTCCTC	This Study
thioredoxin-disulfide reductase	TRR1_F	ATGTCTCCCATCGCCAAC	This Study
thioredoxin-disulfide reductase	TRR1_R	GTAGAGGACAGGCTCGAGGT	This Study
alternative oxidase 1	AOX1_F	CTCTGCCGTTTCAAGCACTC	This Study
alternative oxidase 1	AOX1_R	CCCCTGCCTTCTGATCCTAC	This Study
actin	ACTIN_F	CACCATTGGTAACGAGCGATTC	Horianopolous, 2020
actin	ACTIN_R	TGGTAGTACCACCAGACATGAC	Horianopolous, 2020

Table S6. Data sheets of the RNA-seq data for antimycin A treatment.

	baseMean	log2FoldCh	lfcSE	stat	pvalue	padj
CNAG_07304	612.5685	-0.29494	0.112575	-2.61992	0.008795	0.030559
CNAG_00003	534.756	-0.19602	0.099228	-1.97543	0.04822	0.121038
CNAG_00005	102.6655	-1.11235	0.208394	-5.33774	9.41E-08	9.58E-07
CNAG_12001	26.87368	-0.90325	0.391048	-2.30983	0.020897	0.061774
CNAG_00006	4305.682	0.190281	0.072586	2.621461	0.008755	0.030449
CNAG_00007	4644.062	0.177629	0.060768	2.92306	0.003466	0.013907
CNAG_00010	2963.125	0.189944	0.077087	2.464028	0.013739	0.043941
CNAG_00011	604.333	0.303674	0.112453	2.700462	0.006924	0.024856
CNAG_00012	1651.897	-0.31942	0.088189	-3.62203	0.000292	0.001613
CNAG_00013	507.8788	0.530465	0.10127	5.238128	1.62E-07	1.59E-06
CNAG_00015	226.4114	0.373218	0.140901	2.648795	0.008078	0.028525
CNAG_00019	2990.429	0.148963	0.071209	2.091933	0.036445	0.096608
CNAG_00020	1074.6	-0.21745	0.108093	-2.01171	0.04425	0.113297
CNAG_00021	938.083	-0.52979	0.090653	-5.84415	5.09E-09	6.22E-08
CNAG_00025	1209.621	0.263638	0.078601	3.354135	0.000796	0.003888
CNAG_00030	167.1032	-0.35569	0.161896	-2.19702	0.028019	0.078967
CNAG_00032	108.6737	-0.40832	0.203916	-2.00241	0.045241	0.115228
CNAG_00034	45017.86	-0.24964	0.071719	-3.48077	0.0005	0.002608
CNAG_00035	159.1964	-0.40602	0.168876	-2.40425	0.016206	0.050167
CNAG_00038	785.118	-0.30876	0.09572	-3.22564	0.001257	0.005777
CNAG_00039	1157.736	-0.38318	0.102317	-3.74504	0.00018	0.00104
CNAG_00040	4557.666	-0.76178	0.071666	-10.6295	2.17E-26	1.30E-24
CNAG_07308	142.4692	1.393547	0.195485	7.12867	1.01E-12	1.91E-11
CNAG_12006	223.733	1.04596	0.170028	6.151682	7.67E-10	1.04E-08
CNAG_00044	2577.006	0.169627	0.067553	2.511031	0.012038	0.039439
CNAG_00046	4115.64	-0.20565	0.073753	-2.7884	0.005297	0.019741
CNAG_00047	578.3429	1.175948	0.11686	10.06287	8.06E-24	4.08E-22
CNAG_00052	668.8482	1.387881	0.122917	11.29119	1.45E-29	1.01E-27
CNAG_00053	532.5399	0.348061	0.099251	3.506883	0.000453	0.002397
CNAG_00054	126.3972	0.961799	0.185727	5.178567	2.24E-07	2.16E-06
CNAG_00055	1084.783	-0.17307	0.086204	-2.00772	0.044674	0.114081
CNAG_00057	5954.624	0.68475	0.075798	9.033892	1.66E-19	5.72E-18
CNAG_00059	315.0006	-0.46235	0.133703	-3.45806	0.000544	0.00281
CNAG_00061	26638.51	0.908546	0.068598	13.2445	4.86E-40	6.65E-38
CNAG_00062	3405.8	-0.22812	0.065848	-3.46435	0.000532	0.002756
CNAG_00067	3079.263	-0.13164	0.066223	-1.9879	0.046823	0.118101
CNAG_00068	1109.52	-0.37845	0.104762	-3.61247	0.000303	0.001663
CNAG_00070	1099.423	0.188667	0.08321	2.267369	0.023368	0.067635
CNAG_00072	2526.276	0.247378	0.085082	2.90754	0.003643	0.014487
CNAG_00073	3947.203	0.13903	0.065553	2.120876	0.033932	0.09125
CNAG_00074	4738.152	0.174589	0.061994	2.816242	0.004859	0.018354
CNAG_00075	1959.588	0.22914	0.093655	2.446643	0.014419	0.045688
CNAG_00076	1510.456	0.200363	0.084751	2.364129	0.018073	0.054793
CNAG_00078	2451.457	0.477263	0.067671	7.052691	1.75E-12	3.22E-11
CNAG_00079	679.7749	0.809134	0.10366	7.805653	5.92E-15	1.35E-13
CNAG_00081	2846.796	-0.19661	0.065485	-3.00229	0.00268	0.011123
CNAG_00083	1491.087	-0.33131	0.078706	-4.20942	2.56E-05	0.000178

CNAG_00084	3643.087	-0.33026	0.07206	-4.58319	4.58E-06	3.64E-05
CNAG_00086	2145.345	-0.34185	0.068481	-4.99181	5.98E-07	5.40E-06
CNAG_00087	1302.887	0.268695	0.080521	3.33695	0.000847	0.00411
CNAG_00088	3710.145	0.197523	0.0678	2.913335	0.003576	0.014309
CNAG_00089	1447.737	-0.20811	0.081954	-2.53931	0.011107	0.036948
CNAG_00091	6500.386	1.218663	0.186992	6.517183	7.16E-11	1.10E-09
CNAG_00093	3471.411	1.120857	0.080934	13.84905	1.29E-43	2.05E-41
CNAG_00094	238.4408	-0.36972	0.141182	-2.61876	0.008825	0.030609
CNAG_00096	627.9364	0.269681	0.101835	2.648208	0.008092	0.028562
CNAG_00098	466.4089	0.375887	0.107911	3.483302	0.000495	0.002591
CNAG_00100	7128.253	-0.13309	0.063947	-2.08133	0.037404	0.098561
CNAG_07309	2149.173	0.271326	0.079746	3.402373	0.000668	0.003357
CNAG_07310	1971.141	0.149273	0.071469	2.088639	0.03674	0.09716
CNAG_00104	7200.725	-0.18522	0.063465	-2.91848	0.003517	0.014098
CNAG_00107	2663.085	0.930289	0.089767	10.3634	3.64E-25	2.01E-23
CNAG_00108	4355.37	-0.13485	0.066779	-2.01939	0.043447	0.111635
CNAG_00116	40784.8	-0.22366	0.081314	-2.7506	0.005949	0.021764
CNAG_00117	4085.103	-0.18918	0.069073	-2.73881	0.006166	0.022467
CNAG_00118	343.3595	-0.24609	0.124979	-1.96906	0.048946	0.122416
CNAG_00120	889.5944	-0.23844	0.104361	-2.28477	0.022326	0.065111
CNAG_00121	6866.365	0.595662	0.060555	9.836673	7.83E-23	3.61E-21
CNAG_00122	752.1669	0.276631	0.11081	2.496449	0.012544	0.040927
CNAG_00123	514.0227	0.843377	0.111255	7.580548	3.44E-14	7.42E-13
CNAG_00124	1179.171	0.354346	0.082911	4.273802	1.92E-05	0.000137
CNAG_00126	1963.757	-0.62194	0.091651	-6.78593	1.15E-11	1.95E-10
CNAG_00130	7654.827	0.795493	0.081378	9.775261	1.44E-22	6.49E-21
CNAG_00134	830.2532	0.295645	0.096073	3.077291	0.002089	0.008923
CNAG_00137	1722.345	0.232793	0.07502	3.103099	0.001915	0.008307
CNAG_00139	2816.956	0.250353	0.076925	3.254514	0.001136	0.005302
CNAG_00142	1705.801	-0.68108	0.08859	-7.68806	1.49E-14	3.31E-13
CNAG_12024	508.1234	-0.30319	0.141595	-2.14127	0.032252	0.087822
CNAG_00143	4192.941	-0.18787	0.062767	-2.99309	0.002762	0.011422
CNAG_00148	2884.437	-0.17552	0.080684	-2.17542	0.029599	0.082354
CNAG_00149	9503.464	0.783376	0.072579	10.79346	3.70E-27	2.38E-25
CNAG_00150	3061.492	0.312527	0.081104	3.853386	0.000116	0.000706
CNAG_00152	839.036	-0.31734	0.090898	-3.49113	0.000481	0.002521
CNAG_00156	1325.854	0.217107	0.09473	2.291842	0.021915	0.064222
CNAG_00157	1284.159	0.236278	0.08247	2.865022	0.00417	0.016222
CNAG_00161	265.0703	-0.43068	0.141348	-3.04691	0.002312	0.009743
CNAG_00162	22065.14	2.595444	0.074148	35.00338	2.00E-268	7.80E-265
CNAG_00164	852.8536	-0.73035	0.103953	-7.02573	2.13E-12	3.86E-11
CNAG_00165	2513.732	-0.22396	0.073254	-3.05727	0.002234	0.009453
CNAG_00166	1151.103	-0.5723	0.07972	-7.17891	7.03E-13	1.34E-11
CNAG_07314	498.1941	0.365118	0.106563	3.426305	0.000612	0.003119
CNAG_00172	1096.888	-0.22997	0.115522	-1.99069	0.046515	0.117477
CNAG_00173	589.2888	0.435593	0.107228	4.062323	4.86E-05	0.000321
CNAG_00180	1004.457	0.199758	0.093983	2.125484	0.033546	0.090445
CNAG_00183	928.6153	0.52378	0.121474	4.311887	1.62E-05	0.000118

CNAG_00184	2559.452	-0.19691	0.076619	-2.57002	0.010169	0.034296
CNAG_00185	1430.245	0.181852	0.079151	2.297519	0.021589	0.063434
CNAG_00187	1534.297	-0.14527	0.074044	-1.96192	0.049772	0.124176
CNAG_00190	1546.54	0.430034	0.078442	5.482161	4.20E-08	4.50E-07
CNAG_00192	2384.082	-0.66538	0.092063	-7.22748	4.92E-13	9.62E-12
CNAG_00193	4456.988	-0.16506	0.074072	-2.22835	0.025857	0.073781
CNAG_00195	885.2141	-0.41049	0.089658	-4.57841	4.69E-06	3.71E-05
CNAG_07317	1758.987	0.341201	0.087215	3.912209	9.15E-05	0.000567
CNAG_07319	2425.49	0.609632	0.08731	6.982382	2.90E-12	5.15E-11
CNAG_07320	1297.58	0.293439	0.081389	3.605408	0.000312	0.001703
CNAG_07322	3408.414	-0.16154	0.067756	-2.38412	0.01712	0.052459
CNAG_07326	3923.709	0.308122	0.066725	4.617806	3.88E-06	3.13E-05
CNAG_07333	2292.936	-0.24592	0.083986	-2.92806	0.003411	0.013699
CNAG_07334	639.9327	-0.20389	0.094109	-2.16658	0.030267	0.083702
CNAG_07028	2694.725	-0.19425	0.070434	-2.75787	0.005818	0.021356
CNAG_07338	753.2874	0.360327	0.093361	3.859492	0.000114	0.000691
CNAG_07339	1547.973	0.439683	0.077433	5.678232	1.36E-08	1.57E-07
CNAG_07340	396.7786	0.295938	0.11007	2.688637	0.007174	0.025589
CNAG_07342	1684.305	0.964963	0.07583	12.72533	4.28E-37	5.06E-35
CNAG_00232	32143.55	-0.18428	0.073296	-2.51421	0.01193	0.0392
CNAG_00233	1659.769	0.358204	0.078809	4.545208	5.49E-06	4.30E-05
CNAG_00234	645.4123	-0.40336	0.093407	-4.31833	1.57E-05	0.000114
CNAG_00235	3472.864	0.229618	0.066152	3.471085	0.000518	0.002697
CNAG_00236	740.1669	0.509973	0.089849	5.675918	1.38E-08	1.59E-07
CNAG_00237	10935.84	-0.58614	0.062854	-9.32532	1.11E-20	4.25E-19
CNAG_00238	18091.05	-0.15884	0.066482	-2.38918	0.016886	0.051902
CNAG_00241	1733.516	-0.24152	0.080757	-2.99066	0.002784	0.011494
CNAG_00248	506.4665	0.345364	0.106381	3.246491	0.001168	0.005421
CNAG_00249	729.7675	0.284002	0.101151	2.807693	0.00499	0.018784
CNAG_00250	1932.82	0.275109	0.076988	3.573389	0.000352	0.001906
CNAG_00254	1225.319	0.655775	0.093269	7.031001	2.05E-12	3.74E-11
CNAG_00256	4157.907	-0.21348	0.088599	-2.40955	0.015972	0.04964
CNAG_00257	2874.265	-0.51019	0.096154	-5.30597	1.12E-07	1.12E-06
CNAG_00259	2018.895	-0.51914	0.074272	-6.98966	2.76E-12	4.90E-11
CNAG_00260	3007.873	-0.20286	0.068439	-2.96416	0.003035	0.012362
CNAG_00261	9593.965	0.539915	0.081374	6.635005	3.24E-11	5.15E-10
CNAG_00264	1945.682	0.312288	0.090958	3.433306	0.000596	0.00305
CNAG_00265	1540.903	-0.28047	0.082645	-3.39363	0.00069	0.003453
CNAG_00268	7224.141	-0.36042	0.060252	-5.98191	2.21E-09	2.82E-08
CNAG_00271	742.249	-0.22803	0.094061	-2.42427	0.015339	0.048075
CNAG_00272	1532.992	-0.23822	0.096928	-2.45766	0.013985	0.044562
CNAG_00275	442.0507	0.918975	0.131282	6.999981	2.56E-12	4.58E-11
CNAG_00280	3737.381	0.195597	0.071559	2.733355	0.006269	0.022788
CNAG_00283	352.0719	-0.84711	0.117508	-7.20894	5.64E-13	1.09E-11
CNAG_00290	1382.506	0.285991	0.076852	3.721331	0.000198	0.001134
CNAG_00292	4623.515	-0.52873	0.073595	-7.18426	6.76E-13	1.30E-11
CNAG_00299	1284.349	-0.35778	0.099558	-3.59374	0.000326	0.001774
CNAG_00301	975.2737	0.887984	0.093119	9.536034	1.48E-21	6.16E-20

CNAG_00304	654.0667	-0.22983	0.101062	-2.27412	0.022959	0.066681
CNAG_00305	8055.46	-0.1663	0.069457	-2.39426	0.016654	0.051208
CNAG_12043	31.57865	-0.6879	0.344128	-1.99898	0.045611	0.115903
CNAG_00306	8806.955	-0.61466	0.187011	-3.28675	0.001014	0.004811
CNAG_00307	1548.027	0.399382	0.073427	5.439164	5.35E-08	5.62E-07
CNAG_00309	1216.865	-0.20835	0.081493	-2.55673	0.010566	0.035435
CNAG_00314	716.0909	0.379305	0.097037	3.908873	9.27E-05	0.000574
CNAG_12046	472.7305	1.713788	0.132903	12.89505	4.80E-38	6.04E-36
CNAG_00317	933.9949	-0.22678	0.087129	-2.60278	0.009247	0.031817
CNAG_00328	977.4552	0.198737	0.084643	2.347937	0.018878	0.056706
CNAG_00331	80.57773	-0.92553	0.226837	-4.08016	4.50E-05	0.000298
CNAG_00337	1496.629	0.233003	0.077249	3.016265	0.002559	0.010663
CNAG_00342	906.4604	-0.1833	0.087486	-2.09522	0.036151	0.096025
CNAG_00346	505.6638	0.373277	0.107707	3.465679	0.000529	0.002744
CNAG_00347	1159.904	-0.25834	0.097897	-2.63894	0.008317	0.029135
CNAG_07356	4529.25	0.679481	0.068589	9.906546	3.90E-23	1.87E-21
CNAG_00351	472.5929	-0.27446	0.107217	-2.55987	0.010471	0.035147
CNAG_00358	1046.005	-0.30488	0.084489	-3.60859	0.000308	0.001684
CNAG_00360	221.164	0.4254	0.173681	2.449319	0.014313	0.045386
CNAG_00363	4451.252	0.168388	0.082392	2.043755	0.040978	0.106526
CNAG_00369	771.6722	-0.18517	0.094429	-1.96094	0.049885	0.124299
CNAG_00370	35031.63	-0.15093	0.073894	-2.04254	0.041098	0.106766
CNAG_00371	237.6394	-0.34371	0.143296	-2.39857	0.016459	0.050809
CNAG_00373	785.7733	0.357517	0.089799	3.981287	6.85E-05	0.000441
CNAG_00375	2140.087	-0.23139	0.089707	-2.57942	0.009897	0.033575
CNAG_00377	5619.067	-0.15004	0.07592	-1.97626	0.048125	0.12099
CNAG_00378	231.9003	-0.65013	0.13945	-4.66209	3.13E-06	2.54E-05
CNAG_00385	2059.542	-0.27524	0.070485	-3.90491	9.43E-05	0.000582
CNAG_00386	7230.443	-0.15623	0.060675	-2.57492	0.010026	0.033916
CNAG_00387	475.8273	-0.21478	0.106452	-2.01763	0.043629	0.111928
CNAG_00388	1009.156	-0.19899	0.083934	-2.37076	0.017752	0.054037
CNAG_07951	1039.823	-0.48115	0.083607	-5.75491	8.67E-09	1.02E-07
CNAG_00391	283.0611	-0.45031	0.133133	-3.3824	0.000719	0.003574
CNAG_00394	642.9983	-0.28869	0.097731	-2.95388	0.003138	0.012728
CNAG_00400	3001.344	0.34487	0.074452	4.632127	3.62E-06	2.92E-05
CNAG_00404	169.9867	0.343097	0.161665	2.122266	0.033815	0.090998
CNAG_00407	7836.201	0.338023	0.066294	5.098864	3.42E-07	3.22E-06
CNAG_00409	1596.008	0.567575	0.07825	7.253355	4.07E-13	8.01E-12
CNAG_00411	1467.664	-0.19746	0.097178	-2.03194	0.04216	0.109018
CNAG_00413	3851.946	0.24036	0.072625	3.309611	0.000934	0.004478
CNAG_00414	362.2834	0.346945	0.116499	2.978091	0.002901	0.011907
CNAG_00418	19488.02	-0.41692	0.066973	-6.2253	4.81E-10	6.65E-09
CNAG_00421	757.4651	0.292685	0.099006	2.956244	0.003114	0.012651
CNAG_00423	599.0324	0.288689	0.097305	2.966853	0.003009	0.012267
CNAG_00429	435.1978	-0.59395	0.112616	-5.27413	1.33E-07	1.32E-06
CNAG_07359	2353.146	0.254707	0.114801	2.218679	0.026509	0.075254
CNAG_00433	252.7549	-0.77569	0.146338	-5.30069	1.15E-07	1.15E-06
CNAG_00439	2102.633	-0.22165	0.096579	-2.29503	0.021731	0.06378

CNAG_00441	13181.91	-0.55977	0.060253	-9.29041	1.54E-20	5.82E-19
CNAG_00442	2195.8	0.344578	0.070897	4.860278	1.17E-06	1.01E-05
CNAG_00443	264.8006	-0.30773	0.139765	-2.20178	0.027681	0.078184
CNAG_12058	49.59243	0.605379	0.280973	2.154583	0.031194	0.085591
CNAG_00447	7757.861	-0.18269	0.061152	-2.98743	0.002813	0.011603
CNAG_00449	1713.257	-0.55521	0.071454	-7.7702	7.84E-15	1.78E-13
CNAG_00451	439.2307	-0.56628	0.111795	-5.06531	4.08E-07	3.78E-06
CNAG_00453	3375.061	0.91773	0.073666	12.45807	1.26E-35	1.32E-33
CNAG_00455	2021.609	-0.47565	0.076063	-6.25334	4.02E-10	5.63E-09
CNAG_00456	3305.442	-0.19767	0.078369	-2.52225	0.011661	0.038527
CNAG_00457	23872.13	0.259532	0.077625	3.343418	0.000828	0.004024
CNAG_00461	3307.501	-0.20125	0.070036	-2.87346	0.00406	0.015874
CNAG_00462	6061.184	0.169238	0.080778	2.095089	0.036163	0.096025
CNAG_12060	194.537	-0.68935	0.155256	-4.44007	8.99E-06	6.83E-05
CNAG_00465	1870.958	0.603871	0.101681	5.938892	2.87E-09	3.61E-08
CNAG_00467	323.3853	0.326864	0.128916	2.535489	0.011229	0.037242
CNAG_00468	1891.303	0.222921	0.071192	3.131273	0.001741	0.007652
CNAG_00471	1754.085	0.841611	0.083609	10.06599	7.81E-24	3.98E-22
CNAG_00474	214.193	0.506579	0.169162	2.994643	0.002748	0.011375
CNAG_07362	9966.675	-0.41621	0.080972	-5.14013	2.75E-07	2.63E-06
CNAG_07363	11222.87	1.12379	0.075682	14.84874	7.09E-50	1.50E-47
CNAG_00480	1485.43	0.810909	0.093749	8.649831	5.16E-18	1.50E-16
CNAG_00483	17895.43	-0.13196	0.062602	-2.108	0.035031	0.093691
CNAG_00485	1293.975	1.141769	0.12783	8.931964	4.19E-19	1.38E-17
CNAG_00486	1953.556	0.430115	0.082976	5.183586	2.18E-07	2.10E-06
CNAG_00491	822.6642	0.303663	0.090725	3.347059	0.000817	0.003979
CNAG_07364	670.5411	-0.2181	0.0926	-2.35528	0.018509	0.055856
CNAG_00497	2584.491	0.757063	0.076035	9.956818	2.35E-23	1.15E-21
CNAG_00498	636.582	0.249318	0.096121	2.593784	0.009493	0.032477
CNAG_00500	2300.725	0.171479	0.074516	2.301222	0.021379	0.062919
CNAG_00503	966.7956	0.17297	0.082749	2.090295	0.036591	0.096832
CNAG_00505	349.4323	-0.48189	0.125645	-3.8353	0.000125	0.000756
CNAG_00513	2992.838	-0.5183	0.068539	-7.56201	3.97E-14	8.49E-13
CNAG_00519	2665.757	-0.56765	0.073357	-7.73823	1.01E-14	2.27E-13
CNAG_00520	4963.452	-0.17281	0.085985	-2.00981	0.044452	0.113668
CNAG_00521	1297.149	0.50388	0.080117	6.289331	3.19E-10	4.54E-09
CNAG_00522	3246.738	0.654199	0.068037	9.615336	6.89E-22	2.91E-20
CNAG_00527	3783.329	-0.22197	0.069067	-3.21379	0.00131	0.005992
CNAG_00528	674.6286	0.312526	0.098636	3.168471	0.001532	0.006849
CNAG_00529	1056.068	-0.54534	0.091699	-5.94708	2.73E-09	3.44E-08
CNAG_00531	3166.48	0.566303	0.065203	8.685169	3.78E-18	1.13E-16
CNAG_12064	38.18052	1.133265	0.334218	3.390799	0.000697	0.003486
CNAG_00532	1118.518	-0.52198	0.081229	-6.4261	1.31E-10	1.94E-09
CNAG_00533	5147.509	0.671883	0.06415	10.4737	1.14E-25	6.55E-24
CNAG_00534	4457.752	0.200872	0.071418	2.812635	0.004914	0.018543
CNAG_00537	428.7743	0.268993	0.126718	2.122768	0.033773	0.090916
CNAG_00539	547.6385	0.919586	0.117442	7.830145	4.87E-15	1.13E-13
CNAG_00543	1093.06	-0.31167	0.085291	-3.65423	0.000258	0.001448

CNAG_00546	1325.496	-0.21496	0.100884	-2.13076	0.033109	0.08956
CNAG_07955	381.9846	0.276029	0.11463	2.408006	0.01604	0.049791
CNAG_00550	506.3894	0.369591	0.109544	3.373894	0.000741	0.003668
CNAG_00551	5444.647	-0.23323	0.070148	-3.32481	0.000885	0.004273
CNAG_00554	967.2645	0.233493	0.085276	2.7381	0.00618	0.022504
CNAG_00557	1661.027	-0.18886	0.071771	-2.63138	0.008504	0.02964
CNAG_00563	1126.387	0.424014	0.09603	4.415426	1.01E-05	7.62E-05
CNAG_00564	820.5287	0.205167	0.090683	2.262461	0.023669	0.068285
CNAG_00565	5806.928	-0.22285	0.063666	-3.50029	0.000465	0.002449
CNAG_00566	1352.818	0.35866	0.082727	4.335449	1.45E-05	0.000107
CNAG_00573	2364.372	0.221272	0.083601	2.646764	0.008127	0.028671
CNAG_00575	2050.531	1.177389	0.2127	5.535444	3.10E-08	3.38E-07
CNAG_00577	796.546	0.234181	0.088833	2.636207	0.008384	0.029274
CNAG_00580	3576.142	-0.17545	0.084261	-2.08218	0.037326	0.098424
CNAG_00581	7037.32	0.335918	0.067263	4.994099	5.91E-07	5.35E-06
CNAG_00582	2266.199	0.399263	0.078253	5.102181	3.36E-07	3.17E-06
CNAG_00583	3497.438	-0.18178	0.091986	-1.97612	0.048141	0.12099
CNAG_00585	1463.196	-0.70509	0.081778	-8.62202	6.58E-18	1.90E-16
CNAG_00588	292.8255	0.789089	0.126231	6.251136	4.07E-10	5.70E-09
CNAG_00590	2703.997	-0.25477	0.075438	-3.37714	0.000732	0.003631
CNAG_00591	134.2618	0.482809	0.203105	2.377143	0.017447	0.053333
CNAG_12079	291.1179	0.523769	0.134516	3.893738	9.87E-05	0.000608
CNAG_00592	452.7693	-0.28727	0.116254	-2.47105	0.013472	0.043229
CNAG_00595	507.2928	0.736808	0.110246	6.683323	2.34E-11	3.83E-10
CNAG_00596	167.5394	0.498656	0.156825	3.179695	0.001474	0.006639
CNAG_00597	280.5505	-0.56604	0.149323	-3.79071	0.00015	0.000887
CNAG_00598	43.70662	0.77968	0.338428	2.30383	0.021232	0.062645
CNAG_00602	5018.921	-0.23056	0.074598	-3.09074	0.001997	0.008604
CNAG_00603	1405.753	-0.48424	0.083165	-5.8226	5.79E-09	7.00E-08
CNAG_00607	1650.294	-0.40338	0.075464	-5.34533	9.03E-08	9.21E-07
CNAG_07957	1831.576	-0.40635	0.075332	-5.39407	6.89E-08	7.12E-07
CNAG_00619	683.1582	-0.21592	0.09849	-2.19234	0.028355	0.079655
CNAG_07374	969.8705	-0.48408	0.091939	-5.26526	1.40E-07	1.38E-06
CNAG_00625	1886.941	0.174455	0.088455	1.972243	0.048582	0.121751
CNAG_00626	9321.154	0.394367	0.066618	5.919862	3.22E-09	4.03E-08
CNAG_00627	153.0265	0.705947	0.179771	3.92692	8.60E-05	0.00054
CNAG_07376	2418.857	-0.1776	0.070473	-2.52014	0.011731	0.038676
CNAG_00633	2572.42	-0.41439	0.083984	-4.93408	8.05E-07	7.09E-06
CNAG_00637	164.6526	0.815944	0.187966	4.340926	1.42E-05	0.000104
CNAG_00638	2734.528	0.787371	0.087064	9.043642	1.52E-19	5.30E-18
CNAG_00640	87776.28	-0.24537	0.072139	-3.40138	0.00067	0.003365
CNAG_00641	4401.029	-0.21226	0.083453	-2.54344	0.010977	0.036561
CNAG_00643	1053.121	-0.29807	0.082382	-3.61815	0.000297	0.001631
CNAG_00647	803.5786	-0.2379	0.106239	-2.23928	0.025138	0.072018
CNAG_00652	564.0581	0.211867	0.097038	2.183337	0.029011	0.081087
CNAG_00656	58586.95	-0.24208	0.070669	-3.42551	0.000614	0.003126
CNAG_00659	377.5149	-0.24284	0.115219	-2.10764	0.035062	0.093691
CNAG_00662	1880.432	0.213355	0.079122	2.696512	0.007007	0.025095

CNAG_00663	954.551	0.3663	0.092139	3.975516	7.02E-05	0.000451
CNAG_00666	3115.148	-0.25221	0.095624	-2.63752	0.008351	0.029213
CNAG_00669	791.5782	-0.57364	0.095279	-6.02066	1.74E-09	2.24E-08
CNAG_00672	46279.72	-0.30365	0.077173	-3.93467	8.33E-05	0.000526
CNAG_00673	2653.11	0.255633	0.06627	3.85747	0.000115	0.000695
CNAG_00674	1701.648	0.384805	0.108685	3.540561	0.000399	0.002131
CNAG_00675	914.4221	-0.38955	0.09076	-4.29213	1.77E-05	0.000128
CNAG_00680	1549.456	-0.19104	0.076634	-2.4929	0.01267	0.041234
CNAG_00686	16716.75	0.784895	0.081323	9.651574	4.84E-22	2.09E-20
CNAG_00688	476.0841	-0.36744	0.104251	-3.5246	0.000424	0.002249
CNAG_00696	727.6468	0.414075	0.097906	4.229324	2.34E-05	0.000163
CNAG_00697	5290.483	0.139692	0.06333	2.205793	0.027399	0.077498
CNAG_00698	2114.03	0.330379	0.076775	4.303233	1.68E-05	0.000122
CNAG_12092	158.9682	-0.48365	0.167204	-2.89256	0.003821	0.015084
CNAG_00700	11655.95	-0.2116	0.060259	-3.51152	0.000446	0.002357
CNAG_00701	2651.525	0.133724	0.065943	2.027878	0.042573	0.109831
CNAG_00702	3577.116	-0.31984	0.080921	-3.95247	7.73E-05	0.000492
CNAG_00703	41251.74	-0.16763	0.072209	-2.32151	0.02026	0.060231
CNAG_00706	1619.114	-0.49625	0.075918	-6.53667	6.29E-11	9.70E-10
CNAG_00707	2168.993	-0.644	0.078093	-8.24658	1.63E-16	4.21E-15
CNAG_00708	2177.472	-0.18849	0.078725	-2.39426	0.016654	0.051208
CNAG_00710	2020.283	0.669618	0.107256	6.243151	4.29E-10	5.99E-09
CNAG_00715	1739.219	0.276874	0.076694	3.610123	0.000306	0.001676
CNAG_00716	27174.34	0.670161	0.058908	11.37634	5.49E-30	3.89E-28
CNAG_00717	868.95	0.376683	0.089229	4.221508	2.43E-05	0.000169
CNAG_00718	951.3492	-0.24184	0.08921	-2.71092	0.00671	0.02423
CNAG_00719	2475.124	-0.31605	0.088406	-3.57503	0.00035	0.001897
CNAG_00720	1330.905	-0.23239	0.088709	-2.61969	0.008801	0.030566
CNAG_07381	1452.06	-0.53468	0.092524	-5.77884	7.52E-09	8.95E-08
CNAG_12095	39.37405	-0.77632	0.338531	-2.2932	0.021836	0.064032
CNAG_00727	1714.436	0.392633	0.078102	5.0272	4.98E-07	4.58E-06
CNAG_00728	200.4943	-0.81357	0.154043	-5.28144	1.28E-07	1.27E-06
CNAG_00730	3907.278	-0.53147	0.088445	-6.00911	1.87E-09	2.40E-08
CNAG_00732	3163.547	0.783679	0.091612	8.554311	1.19E-17	3.32E-16
CNAG_00733	1694.399	-0.5267	0.074388	-7.08046	1.44E-12	2.68E-11
CNAG_00738	93.14626	-0.62781	0.212019	-2.9611	0.003065	0.012466
CNAG_00739	1918.053	-0.17142	0.075675	-2.26516	0.023503	0.067881
CNAG_00741	12329.03	-0.22346	0.0665	-3.36036	0.000778	0.003818
CNAG_00743	3442.525	-0.23317	0.065307	-3.57035	0.000356	0.001923
CNAG_12103	46.93698	0.611588	0.285293	2.143718	0.032055	0.087438
CNAG_00745	2578.961	0.520137	0.088511	5.876549	4.19E-09	5.18E-08
CNAG_00747	11315.05	0.659842	0.064991	10.15278	3.22E-24	1.72E-22
CNAG_00750	1143.947	-0.28169	0.091276	-3.08617	0.002028	0.008703
CNAG_00752	421.9066	-0.46585	0.134905	-3.4532	0.000554	0.002855
CNAG_00755	689.5308	0.305855	0.099101	3.086291	0.002027	0.008703
CNAG_12110	136.2164	0.499738	0.189259	2.640502	0.008278	0.029039
CNAG_00760	1876.518	-0.22096	0.0738	-2.99409	0.002753	0.01139
CNAG_00762	687.4904	-0.68542	0.092159	-7.43735	1.03E-13	2.14E-12

CNAG_07960	367.2812	-0.257	0.114654	-2.24154	0.024991	0.071623
CNAG_00764	2707.502	-0.63216	0.070431	-8.97557	2.82E-19	9.48E-18
CNAG_00765	673.2026	0.397478	0.098539	4.033694	5.49E-05	0.000359
CNAG_00766	606.8175	0.205772	0.10192	2.018945	0.043493	0.111651
CNAG_00768	1027.272	0.296037	0.086605	3.418248	0.00063	0.003192
CNAG_00771	16463.81	-0.22942	0.072143	-3.18	0.001473	0.006636
CNAG_00772	2633.486	-0.17685	0.085523	-2.06785	0.038654	0.101432
CNAG_00773	619.876	0.443169	0.100319	4.417579	9.98E-06	7.56E-05
CNAG_00774	6271.495	-0.39134	0.074367	-5.26225	1.42E-07	1.41E-06
CNAG_00775	1935.152	-0.59989	0.075684	-7.92625	2.26E-15	5.37E-14
CNAG_00776	15678.65	0.328552	0.071428	4.599747	4.23E-06	3.39E-05
CNAG_00777	2272.424	-0.34109	0.080569	-4.23353	2.30E-05	0.000161
CNAG_00779	40203.54	-0.18771	0.071218	-2.63568	0.008397	0.029306
CNAG_00785	32625.59	-0.24324	0.071395	-3.40697	0.000657	0.003305
CNAG_00786	638.7581	0.595714	0.099094	6.011608	1.84E-09	2.37E-08
CNAG_00788	13613.58	-0.26998	0.070681	-3.81967	0.000134	0.000797
CNAG_00789	4.154357	2.56963	1.096795	2.342854	0.019137	NA
CNAG_00790	847.5436	0.211892	0.089023	2.380189	0.017304	0.052935
CNAG_00791	1869.798	0.763899	0.080454	9.494874	2.20E-21	9.01E-20
CNAG_00793	1873.215	-0.32834	0.075963	-4.32233	1.54E-05	0.000112
CNAG_00794	1282.257	-0.19165	0.076811	-2.49514	0.012591	0.041026
CNAG_00796	1485.204	0.328532	0.106946	3.07193	0.002127	0.009055
CNAG_00800	320.4779	0.469522	0.159256	2.948226	0.003196	0.012943
CNAG_00802	451.065	0.528489	0.109167	4.841087	1.29E-06	1.11E-05
CNAG_00803	1267.1	0.339537	0.080264	4.230226	2.33E-05	0.000163
CNAG_00807	917.2597	0.42145	0.093802	4.492988	7.02E-06	5.43E-05
CNAG_00809	4190.756	-0.5999	0.067839	-8.84292	9.33E-19	2.95E-17
CNAG_00810	1328.721	-0.18331	0.082587	-2.21956	0.026449	0.075111
CNAG_00815	1221.923	-0.35499	0.083522	-4.25021	2.14E-05	0.00015
CNAG_00819	12415.82	-0.22419	0.068333	-3.28083	0.001035	0.004898
CNAG_00821	25106.73	-0.21648	0.080202	-2.6992	0.006951	0.024928
CNAG_00822	4666.532	-0.35134	0.06862	-5.12008	3.05E-07	2.90E-06
CNAG_00834	1055.267	1.077333	0.099089	10.87238	1.56E-27	1.04E-25
CNAG_00836	504.3689	-0.30025	0.108282	-2.77287	0.005556	0.020541
CNAG_00838	258.1273	0.539385	0.139537	3.865548	0.000111	0.000675
CNAG_00841	380.5485	-0.30996	0.124754	-2.48454	0.012972	0.04204
CNAG_00843	254.9845	0.40244	0.140503	2.864289	0.004179	0.016251
CNAG_00845	1948.438	-0.52362	0.074027	-7.0734	1.51E-12	2.80E-11
CNAG_00846	799.1004	-0.52182	0.091097	-5.7282	1.02E-08	1.19E-07
CNAG_00848	3790.923	1.610045	0.200843	8.016449	1.09E-15	2.65E-14
CNAG_00851	171.343	0.834074	0.181028	4.607441	4.08E-06	3.27E-05
CNAG_00854	2887.576	-0.7953	0.087952	-9.0425	1.53E-19	5.31E-18
CNAG_00855	794.9392	0.300863	0.094326	3.189615	0.001425	0.006453
CNAG_07389	552.2446	-0.46916	0.106328	-4.41241	1.02E-05	7.70E-05
CNAG_07392	403.7308	0.350784	0.113193	3.098997	0.001942	0.008405
CNAG_07393	198.8998	0.391989	0.155841	2.515307	0.011893	0.039095
CNAG_06800	1362.278	1.186491	0.099666	11.9047	1.12E-32	9.49E-31
CNAG_06798	1952.386	-0.39069	0.073855	-5.28997	1.22E-07	1.22E-06

CNAG_06794	2128.189	0.536676	0.077791	6.89894	5.24E-12	9.13E-11
CNAG_06793	1335.432	0.343373	0.08042	4.269737	1.96E-05	0.000139
CNAG_06792	1130.182	0.184149	0.081121	2.270041	0.023205	0.067246
CNAG_06791	1764.578	1.280478	0.100935	12.68617	7.06E-37	8.10E-35
CNAG_06785	367.8878	-0.42888	0.126602	-3.3876	0.000705	0.003514
CNAG_06784	97.09756	-0.54893	0.207851	-2.64098	0.008267	0.029039
CNAG_06782	1726.689	0.253358	0.08599	2.946356	0.003215	0.013002
CNAG_06781	1308.625	0.758784	0.083194	9.120678	7.47E-20	2.67E-18
CNAG_06774	1213.706	-0.4784	0.078008	-6.13263	8.64E-10	1.17E-08
CNAG_06771	577.584	0.695258	0.112082	6.203124	5.54E-10	7.55E-09
CNAG_06770	32082.81	0.358193	0.069326	5.166781	2.38E-07	2.29E-06
CNAG_06769	1004.67	-0.34055	0.088114	-3.8649	0.000111	0.000677
CNAG_06766	2592.628	-0.6082	0.071355	-8.5236	1.55E-17	4.27E-16
CNAG_07962	110.0085	-0.7674	0.193782	-3.96013	7.49E-05	0.000478
CNAG_06765	1566.48	-0.34039	0.101108	-3.36665	0.000761	0.003751
CNAG_06764	2400.663	-0.19149	0.069249	-2.76523	0.005688	0.020929
CNAG_06763	1200.83	0.174076	0.080574	2.160447	0.030738	0.084644
CNAG_06762	2361.643	-0.59921	0.070295	-8.52411	1.54E-17	4.26E-16
CNAG_06761	193.1903	-0.70856	0.15235	-4.65088	3.31E-06	2.68E-05
CNAG_06760	1014.211	-0.67776	0.086651	-7.82174	5.21E-15	1.20E-13
CNAG_06759	829.0469	0.661085	0.095106	6.951044	3.63E-12	6.37E-11
CNAG_06758	614.7194	-1.12348	0.114969	-9.772	1.48E-22	6.66E-21
CNAG_06756	803.102	-0.44055	0.092702	-4.75236	2.01E-06	1.68E-05
CNAG_06753	5342.603	-0.13216	0.061831	-2.13736	0.032568	0.088374
CNAG_06751	3099.824	0.545243	0.07267	7.502995	6.24E-14	1.31E-12
CNAG_06749	731.5475	-0.27797	0.117257	-2.37057	0.017761	0.054037
CNAG_06748	2434.781	-0.55168	0.069975	-7.88406	3.17E-15	7.43E-14
CNAG_06747	33228.71	-0.20021	0.072067	-2.77805	0.005469	0.020264
CNAG_06746	33053.56	-0.2203	0.062922	-3.50114	0.000463	0.002444
CNAG_06745	29322.55	-0.32185	0.073616	-4.372	1.23E-05	9.17E-05
CNAG_06742	2443.88	-0.5829	0.073525	-7.92794	2.23E-15	5.32E-14
CNAG_06741	2380.036	-0.73248	0.088983	-8.23177	1.84E-16	4.75E-15
CNAG_06739	3620.945	0.760242	0.066751	11.38925	4.73E-30	3.39E-28
CNAG_06737	1663.72	0.333043	0.081243	4.099349	4.14E-05	0.000276
CNAG_06732	1641.157	0.378103	0.079203	4.773825	1.81E-06	1.52E-05
CNAG_06729	857.7877	0.803995	0.093705	8.58006	9.48E-18	2.69E-16
CNAG_06723	5976.41	0.647409	0.074706	8.66604	4.47E-18	1.32E-16
CNAG_07465	797.5654	-0.24309	0.102864	-2.36321	0.018117	0.054899
CNAG_06713	3324.841	-0.18878	0.066463	-2.84045	0.004505	0.017268
CNAG_06711	838.4334	0.269618	0.088588	3.043496	0.002338	0.009833
CNAG_06708	650.5178	-0.21794	0.096023	-2.26961	0.023231	0.067296
CNAG_06707	162.5951	-0.41487	0.166898	-2.48576	0.012927	0.041913
CNAG_06706	500.6528	0.70518	0.124923	5.644923	1.65E-08	1.88E-07
CNAG_06703	719.2016	-0.3001	0.096262	-3.11751	0.001824	0.007961
CNAG_03595	3022.792	0.331757	0.095017	3.491565	0.00048	0.00252
CNAG_03596	9622.514	0.787163	0.063648	12.3675	3.92E-35	3.87E-33
CNAG_12146	16.61192	-1.49499	0.50465	-2.96244	0.003052	0.012419
CNAG_03597	150.4832	-0.45708	0.182679	-2.50211	0.012345	0.040294

CNAG_03599	328.3147	-0.67456	0.128684	-5.24199	1.59E-07	1.56E-06
CNAG_03602	10830.03	-0.14842	0.064807	-2.29016	0.022012	0.064386
CNAG_03603	1963.293	-0.71351	0.075248	-9.48212	2.49E-21	1.01E-19
CNAG_03606	9268.047	-0.15562	0.068441	-2.27374	0.022982	0.066704
CNAG_03609	748.5521	0.441803	0.09453	4.673661	2.96E-06	2.42E-05
CNAG_03618	290.1191	-1.35723	0.136563	-9.93848	2.83E-23	1.37E-21
CNAG_03621	12584.74	-0.17882	0.067725	-2.64038	0.008281	0.029039
CNAG_03624	5735.493	-0.58752	0.065111	-9.02332	1.82E-19	6.25E-18
CNAG_03627	9724.413	-0.20169	0.070039	-2.87972	0.00398	0.015625
CNAG_03629	11434.26	0.159381	0.057699	2.762281	0.00574	0.021083
CNAG_03631	1236.268	0.492864	0.098797	4.98863	6.08E-07	5.48E-06
CNAG_03632	1309.275	0.478169	0.087208	5.483058	4.18E-08	4.48E-07
CNAG_03634	1105.446	-0.59253	0.089148	-6.64661	3.00E-11	4.78E-10
CNAG_03636	1243.738	-0.4053	0.082796	-4.89511	9.83E-07	8.54E-06
CNAG_03637	1132.807	0.308333	0.085561	3.603659	0.000314	0.001714
CNAG_03638	2047.506	0.260431	0.081415	3.198823	0.00138	0.006261
CNAG_03639	175.446	-0.59934	0.154904	-3.86908	0.000109	0.000667
CNAG_03645	9165.792	-0.31231	0.071824	-4.34833	1.37E-05	0.000101
CNAG_03646	1230.406	-0.18518	0.081032	-2.28524	0.022299	0.065103
CNAG_03647	950.3065	0.272456	0.085661	3.180636	0.00147	0.006625
CNAG_03654	976.2806	-0.18526	0.092241	-2.00844	0.044596	0.113922
CNAG_03655	3392.419	-0.36087	0.108572	-3.32373	0.000888	0.004281
CNAG_03661	551.7273	0.260743	0.099054	2.632317	0.00848	0.029572
CNAG_03663	1413.154	0.414405	0.075321	5.501876	3.76E-08	4.06E-07
CNAG_03664	2064.58	-0.16901	0.073915	-2.28649	0.022226	0.064914
CNAG_03666	363.6211	0.336129	0.127235	2.641784	0.008247	0.029008
CNAG_03667	3018.225	0.820822	0.076909	10.67268	1.37E-26	8.27E-25
CNAG_03671	349.1286	0.355573	0.133408	2.665307	0.007692	0.027248
CNAG_03673	1053.676	0.286075	0.098199	2.91322	0.003577	0.014309
CNAG_03674	5602.077	0.773577	0.061252	12.62951	1.45E-36	1.60E-34
CNAG_03675	3994.448	-0.66539	0.068652	-9.69218	3.26E-22	1.41E-20
CNAG_03676	930.4278	0.225923	0.083405	2.708744	0.006754	0.024356
CNAG_03679	379.6632	0.539081	0.117388	4.592309	4.38E-06	3.50E-05
CNAG_03681	2957.734	-0.17743	0.06715	-2.6423	0.008235	0.028986
CNAG_03683	477.2899	-0.74174	0.112937	-6.5677	5.11E-11	7.93E-10
CNAG_03684	4489.359	0.133542	0.066305	2.014043	0.044005	0.112817
CNAG_03688	2361.523	0.655	0.080177	8.169441	3.10E-16	7.84E-15
CNAG_03689	1850.58	0.678223	0.082008	8.270243	1.34E-16	3.49E-15
CNAG_07473	6013.493	0.123536	0.061584	2.005969	0.04486	0.114409
CNAG_03692	522.706	0.700947	0.129232	5.423949	5.83E-08	6.10E-07
CNAG_03697	316.899	0.388532	0.129137	3.008675	0.002624	0.010904
CNAG_03699	3274.756	-0.26829	0.070824	-3.7882	0.000152	0.000892
CNAG_03705	3030.614	0.408589	0.07836	5.214285	1.85E-07	1.80E-06
CNAG_03710	1593.907	-0.38558	0.091652	-4.20703	2.59E-05	0.000179
CNAG_03716	3656.425	-0.19773	0.069062	-2.86303	0.004196	0.016299
CNAG_03719	1335.766	-0.41199	0.105528	-3.90405	9.46E-05	0.000584
CNAG_03724	6181.539	-0.6464	0.070054	-9.22717	2.78E-20	1.03E-18
CNAG_03725	7463.028	0.311292	0.06402	4.862392	1.16E-06	1.00E-05

CNAG_03726	3376.766	-0.16112	0.076053	-2.11859	0.034125	0.091674
CNAG_12161	155.8249	0.526597	0.176201	2.988618	0.002802	0.011565
CNAG_03727	1676.123	0.26753	0.090837	2.945162	0.003228	0.013038
CNAG_03728	1059.908	0.403179	0.107386	3.7545	0.000174	0.001008
CNAG_03732	711.6112	0.195365	0.096494	2.024645	0.042904	0.110466
CNAG_03735	1178.029	-0.31639	0.081717	-3.87172	0.000108	0.00066
CNAG_03738	1574.718	0.573028	0.081889	6.997635	2.60E-12	4.64E-11
CNAG_03739	40818.5	-0.16876	0.069724	-2.42037	0.015505	0.048477
CNAG_03747	37968.51	-0.32467	0.078127	-4.15575	3.24E-05	0.00022
CNAG_03748	4431.341	-0.14169	0.065544	-2.16179	0.030634	0.084507
CNAG_03753	756.8126	-0.33953	0.088731	-3.82657	0.00013	0.000779
CNAG_03756	1339.068	0.167317	0.08374	1.998046	0.045712	0.116049
CNAG_03759	22.77619	1.991348	0.434318	4.585	4.54E-06	3.62E-05
CNAG_03762	4037.07	-0.60603	0.06692	-9.05603	1.35E-19	4.80E-18
CNAG_03763	3034.831	-0.31456	0.107894	-2.91545	0.003552	0.014221
CNAG_03765	7995.571	0.399589	0.060805	6.571674	4.98E-11	7.74E-10
CNAG_03766	993.9255	-0.37893	0.089478	-4.23494	2.29E-05	0.00016
CNAG_03769	10933.94	0.293718	0.063035	4.659575	3.17E-06	2.57E-05
CNAG_03770	151.1992	0.451938	0.175174	2.579945	0.009882	0.033543
CNAG_03771	17591.8	0.85273	0.075633	11.27453	1.75E-29	1.21E-27
CNAG_03772	10342.69	-0.44615	0.09375	-4.75897	1.95E-06	1.63E-05
CNAG_07965	15600.03	-0.54944	0.059812	-9.18608	4.07E-20	1.50E-18
CNAG_03776	769.4586	-0.28547	0.092667	-3.08058	0.002066	0.008844
CNAG_03777	370.284	-0.29614	0.123443	-2.399	0.01644	0.05077
CNAG_07481	674.5656	0.211585	0.106152	1.993231	0.046236	0.117038
CNAG_03780	45593.19	-0.19803	0.086831	-2.28059	0.022573	0.065681
CNAG_03781	1567.282	-0.18217	0.081438	-2.23695	0.02529	0.072426
CNAG_03782	24.86599	1.012364	0.453884	2.230446	0.025718	0.07341
CNAG_03785	2662.209	0.345125	0.066133	5.218665	1.80E-07	1.76E-06
CNAG_03786	779.7361	0.319038	0.091927	3.470577	0.000519	0.0027
CNAG_03788	687.7813	-0.24606	0.097463	-2.52464	0.011582	0.038298
CNAG_03789	2432.117	-0.63008	0.071222	-8.84663	9.02E-19	2.86E-17
CNAG_03794	1089.516	0.73261	0.095198	7.69568	1.41E-14	3.13E-13
CNAG_03805	2623.038	0.300821	0.070041	4.29492	1.75E-05	0.000126
CNAG_03806	930.6822	0.225441	0.084446	2.669642	0.007593	0.026911
CNAG_03811	1358.567	-0.17059	0.083001	-2.05528	0.039852	0.104086
CNAG_03812	4068.274	-0.13243	0.06574	-2.0144	0.043968	0.112759
CNAG_03816	2478.317	-0.13709	0.068858	-1.99084	0.046498	0.117472
CNAG_03817	2348.592	-0.48999	0.079335	-6.17622	6.57E-10	8.93E-09
CNAG_03819	8314.676	-0.99748	0.07627	-13.0784	4.38E-39	5.79E-37
CNAG_03820	4262.519	-0.45817	0.089033	-5.14611	2.66E-07	2.55E-06
CNAG_03822	1138.594	-0.1667	0.079086	-2.10777	0.035051	0.093691
CNAG_03824	3990.487	0.864979	0.073451	11.77625	5.17E-32	4.12E-30
CNAG_03832	2213.725	0.308546	0.072041	4.282898	1.84E-05	0.000132
CNAG_03837	292.1742	0.314903	0.137457	2.290912	0.021969	0.064295
CNAG_03839	2086.017	-0.26454	0.089875	-2.94346	0.003246	0.013104
CNAG_03840	1335.133	-0.17368	0.077775	-2.23314	0.02554	0.073008
CNAG_03844	620.2855	0.361203	0.099583	3.627171	0.000287	0.00159

CNAG_03847	2223.398	0.377316	0.074122	5.090492	3.57E-07	3.34E-06
CNAG_03849	1210.465	-0.27317	0.125908	-2.16964	0.030034	0.083145
CNAG_03856	7412.917	0.155485	0.06185	2.513915	0.01194	0.039216
CNAG_03857	3044.118	0.657157	0.06685	9.830326	8.33E-23	3.83E-21
CNAG_03858	2917.452	0.223348	0.065654	3.401915	0.000669	0.00336
CNAG_03861	12358.27	0.391206	0.064411	6.073603	1.25E-09	1.64E-08
CNAG_03864	2099.176	0.200032	0.075502	2.649365	0.008064	0.02849
CNAG_03865	2106.21	0.525276	0.078828	6.663558	2.67E-11	4.30E-10
CNAG_07966	234.5567	-0.28623	0.145656	-1.96514	0.049398	0.1234
CNAG_03872	1426.128	0.225958	0.075896	2.977186	0.002909	0.011936
CNAG_03873	4409.615	0.664223	0.097357	6.822547	8.94E-12	1.53E-10
CNAG_03874	732.5458	-0.18982	0.092362	-2.05513	0.039867	0.104088
CNAG_03875	1776.339	0.15213	0.073257	2.076653	0.037834	0.099579
CNAG_03876	3186.111	0.147583	0.070176	2.103039	0.035462	0.094543
CNAG_03878	1535.734	-0.38775	0.076486	-5.0696	3.99E-07	3.71E-06
CNAG_07482	1013.667	0.237412	0.09863	2.407093	0.01608	0.049837
CNAG_07483	1253.032	0.25992	0.096133	2.70376	0.006856	0.024668
CNAG_03881	1310.787	1.028894	0.089005	11.55991	6.58E-31	4.98E-29
CNAG_03882	905.2851	0.259041	0.084934	3.049918	0.002289	0.009656
CNAG_03890	1809.047	-0.18731	0.074313	-2.52057	0.011717	0.038646
CNAG_03891	23498.27	0.864233	0.075245	11.48551	1.56E-30	1.14E-28
CNAG_03892	13132.3	0.991397	0.063603	15.58723	8.89E-55	2.57E-52
CNAG_03893	726.5878	0.423286	0.092129	4.594504	4.34E-06	3.47E-05
CNAG_03896	286.2776	-0.31816	0.127732	-2.49082	0.012745	0.041407
CNAG_12183	75.13418	0.671566	0.233912	2.871015	0.004092	0.015981
CNAG_03897	1334.79	-0.49801	0.087613	-5.68421	1.31E-08	1.52E-07
CNAG_03899	3477.703	-0.44658	0.063712	-7.00934	2.39E-12	4.32E-11
CNAG_03900	1299.777	0.1803	0.086015	2.096148	0.036069	0.095873
CNAG_03901	749.5176	0.420775	0.094503	4.452518	8.49E-06	6.48E-05
CNAG_03902	3214.907	0.170186	0.06445	2.640583	0.008276	0.029039
CNAG_03904	2765.176	-0.2769	0.071957	-3.84815	0.000119	0.000719
CNAG_03909	470.5902	0.480626	0.113339	4.240617	2.23E-05	0.000156
CNAG_03910	2050.421	1.128724	0.243556	4.634351	3.58E-06	2.90E-05
CNAG_03911	144.0035	-0.57732	0.178421	-3.23572	0.001213	0.005603
CNAG_03912	318.7043	-0.40525	0.123499	-3.28138	0.001033	0.004892
CNAG_03915	377.9713	-0.73851	0.116136	-6.359	2.03E-10	2.96E-09
CNAG_03916	14646.56	0.579307	0.065374	8.861448	7.90E-19	2.54E-17
CNAG_03917	1801.355	-0.18748	0.075943	-2.46873	0.013559	0.043439
CNAG_03920	7099.025	-0.2809	0.061932	-4.53558	5.74E-06	4.49E-05
CNAG_03921	714.8555	-0.72656	0.105514	-6.88593	5.74E-12	9.96E-11
CNAG_03926	3776.781	-0.29599	0.067577	-4.38011	1.19E-05	8.86E-05
CNAG_03929	769.7246	0.381856	0.088106	4.334024	1.46E-05	0.000107
CNAG_03935	2927.916	0.413123	0.065605	6.297126	3.03E-10	4.34E-09
CNAG_03937	1100.464	0.38356	0.101203	3.789988	0.000151	0.000887
CNAG_03940	3536.671	-0.21615	0.068454	-3.15759	0.001591	0.007074
CNAG_03941	3787.776	-0.17157	0.070403	-2.43693	0.014813	0.046744
CNAG_03944	8080.587	-0.35794	0.062633	-5.71485	1.10E-08	1.28E-07
CNAG_03946	1110.741	0.365304	0.095543	3.82346	0.000132	0.000786

CNAG_07486	313.9964	-0.27267	0.135627	-2.0104	0.044388	0.113613
CNAG_03948	4663.777	-0.18006	0.077893	-2.31168	0.020795	0.061542
CNAG_03949	1212.055	-0.21306	0.077456	-2.75071	0.005947	0.021764
CNAG_03950	5031.526	-0.20797	0.086761	-2.39704	0.016528	0.050962
CNAG_03951	2001.498	-0.21758	0.071019	-3.06361	0.002187	0.00927
CNAG_03952	2028.987	-0.1688	0.080088	-2.10768	0.035059	0.093691
CNAG_03954	861.0056	-0.62881	0.099124	-6.3436	2.24E-10	3.26E-09
CNAG_03957	1928.837	-0.2343	0.082497	-2.8401	0.00451	0.017278
CNAG_03958	1204.863	0.251464	0.092947	2.705465	0.006821	0.024553
CNAG_03959	3808.838	0.172415	0.068514	2.516485	0.011853	0.038998
CNAG_03964	1293.078	-0.78294	0.079748	-9.81761	9.46E-23	4.32E-21
CNAG_03967	2719.355	0.239615	0.067945	3.52659	0.000421	0.002236
CNAG_03968	3705.262	-0.18282	0.063983	-2.85738	0.004272	0.016511
CNAG_07487	17159.41	0.166272	0.076506	2.173311	0.029757	0.082642
CNAG_03974	1226.37	0.541067	0.087004	6.218897	5.01E-10	6.90E-09
CNAG_03975	3228.631	-0.57435	0.082075	-6.99789	2.60E-12	4.64E-11
CNAG_03976	1174.509	-0.2538	0.105412	-2.40771	0.016053	0.049793
CNAG_03978	891.7131	0.245575	0.084817	2.895355	0.003787	0.014988
CNAG_03985	2602.199	0.297393	0.069379	4.286513	1.81E-05	0.000131
CNAG_03987	4781.514	0.26988	0.06427	4.199177	2.68E-05	0.000185
CNAG_12196	62.48028	0.663213	0.278399	2.382241	0.017208	0.052703
CNAG_03991	1189.065	0.449317	0.081123	5.538693	3.05E-08	3.33E-07
CNAG_03992	585.8678	0.242237	0.098077	2.469856	0.013517	0.043356
CNAG_03994	919.8753	0.381361	0.104544	3.647851	0.000264	0.001481
CNAG_03997	100.2087	0.426888	0.201801	2.115384	0.034397	0.092246
CNAG_04001	3267.217	-0.14825	0.075575	-1.96162	0.049807	0.124224
CNAG_04004	97320.54	-0.24133	0.070683	-3.41424	0.00064	0.003231
CNAG_04005	3047.628	-0.22061	0.071179	-3.09934	0.00194	0.008399
CNAG_04010	3889.719	-0.14412	0.062206	-2.31684	0.020513	0.060864
CNAG_04014	4185.401	-0.14613	0.067051	-2.17946	0.029297	0.081655
CNAG_04018	1331.148	0.260015	0.094883	2.740375	0.006137	0.022411
CNAG_07489	1033.5	0.248538	0.091335	2.721155	0.006505	0.023558
CNAG_04021	28426.89	-0.27104	0.072212	-3.75345	0.000174	0.00101
CNAG_04023	1806.917	-0.34537	0.083185	-4.1518	3.30E-05	0.000223
CNAG_04027	915.3423	2.668334	0.133734	19.95259	1.42E-88	1.59E-85
CNAG_04030	753.844	0.259721	0.103404	2.511719	0.012014	0.039395
CNAG_04032	6588.442	0.367678	0.058926	6.23964	4.39E-10	6.11E-09
CNAG_04033	2392.557	0.179906	0.067892	2.649878	0.008052	0.028459
CNAG_04035	1040.089	0.246028	0.087065	2.825788	0.004716	0.017955
CNAG_04037	1687.088	-0.31236	0.077325	-4.03956	5.36E-05	0.00035
CNAG_04038	309.81	0.573937	0.131632	4.360166	1.30E-05	9.64E-05
CNAG_04043	365.9154	0.756038	0.115997	6.517717	7.14E-11	1.10E-09
CNAG_04044	1706.885	-0.34511	0.08073	-4.2749	1.91E-05	0.000137
CNAG_04049	1639.928	-0.30213	0.076392	-3.95493	7.66E-05	0.000488
CNAG_04051	2312.351	-0.30155	0.100101	-3.01243	0.002592	0.010775
CNAG_04053	779.1944	-0.35753	0.090287	-3.95993	7.50E-05	0.000478
CNAG_07491	809.9762	0.769215	0.107359	7.164878	7.79E-13	1.49E-11
CNAG_04062	2345.018	-0.5626	0.069276	-8.1211	4.62E-16	1.15E-14

CNAG_04064	6695.034	0.397971	0.069606	5.717513	1.08E-08	1.26E-07
CNAG_04066	4044.871	0.181425	0.069942	2.593948	0.009488	0.032476
CNAG_04067	534.5013	0.677951	0.124086	5.46357	4.67E-08	4.96E-07
CNAG_04068	16110.71	-0.28912	0.092524	-3.1248	0.001779	0.007788
CNAG_04070	175.55	0.394393	0.186734	2.112055	0.034682	0.092881
CNAG_04071	2281.342	-0.17505	0.083698	-2.09146	0.036487	0.096653
CNAG_04072	1935.743	-0.5854	0.072795	-8.04173	8.86E-16	2.17E-14
CNAG_07494	2767.6	-0.43099	0.070259	-6.13425	8.56E-10	1.16E-08
CNAG_04076	946.4165	0.755389	0.095342	7.922981	2.32E-15	5.50E-14
CNAG_04077	597.4135	-0.25198	0.110363	-2.28324	0.022417	0.065276
CNAG_04082	7351.283	-0.14558	0.066838	-2.17809	0.029399	0.081904
CNAG_12208	123.9626	0.739389	0.184209	4.013857	5.97E-05	0.000388
CNAG_04090	3230.467	0.380047	0.066516	5.713597	1.11E-08	1.29E-07
CNAG_04094	289.1225	-0.87833	0.131739	-6.66715	2.61E-11	4.22E-10
CNAG_04096	41.59658	-1.13563	0.309782	-3.66591	0.000246	0.001388
CNAG_04098	844.9568	1.236488	0.107398	11.51311	1.13E-30	8.34E-29
CNAG_04099	431.0719	0.401212	0.108929	3.683239	0.00023	0.001304
CNAG_07499	1911.161	-0.15348	0.077444	-1.98184	0.047498	0.119572
CNAG_06963	11360.45	0.547769	0.07235	7.57113	3.70E-14	7.93E-13
CNAG_12214	86.44692	0.96741	0.238388	4.058126	4.95E-05	0.000326
CNAG_03083	254.0751	0.953703	0.13729	6.946613	3.74E-12	6.56E-11
CNAG_03082	110.0644	1.104808	0.241155	4.581322	4.62E-06	3.67E-05
CNAG_03081	3109.727	0.330667	0.067023	4.933651	8.07E-07	7.09E-06
CNAG_12216	60.24921	-0.82628	0.254768	-3.24326	0.001182	0.005479
CNAG_03079	889.5137	0.480338	0.089346	5.376168	7.61E-08	7.81E-07
CNAG_03069	856.3378	-0.17463	0.089033	-1.96138	0.049835	0.12425
CNAG_03068	343.8929	-2.44858	0.13848	-17.6818	5.79E-70	3.47E-67
CNAG_07502	7344.71	-0.33842	0.060091	-5.63187	1.78E-08	2.00E-07
CNAG_03064	1818.841	-0.53609	0.074335	-7.21183	5.52E-13	1.07E-11
CNAG_03060	1297.152	0.633781	0.11743	5.397092	6.77E-08	7.04E-07
CNAG_03059	888.7763	0.523066	0.087463	5.980423	2.23E-09	2.84E-08
CNAG_03058	19398.56	0.899914	0.0851	10.57476	3.90E-26	2.27E-24
CNAG_03057	301.2376	-0.43693	0.148882	-2.93475	0.003338	0.013456
CNAG_03055	1460.934	-0.36646	0.079365	-4.61747	3.88E-06	3.13E-05
CNAG_03053	30529.76	-0.24513	0.086886	-2.82133	0.004783	0.018148
CNAG_03051	742.2131	-0.23839	0.092676	-2.57233	0.010102	0.034142
CNAG_03048	1920.995	0.180463	0.073554	2.453478	0.014148	0.044993
CNAG_12222	71.70462	0.861097	0.242003	3.558206	0.000373	0.002001
CNAG_03046	823.6451	0.397051	0.095634	4.151785	3.30E-05	0.000223
CNAG_03045	958.8969	0.297925	0.097065	3.069336	0.002145	0.009114
CNAG_03044	385.5059	0.270674	0.122684	2.206275	0.027365	0.077447
CNAG_03041	102.8582	-0.45877	0.199236	-2.30267	0.021298	0.062814
CNAG_03040	3222.885	0.721749	0.072736	9.922851	3.31E-23	1.60E-21
CNAG_03034	393.3073	-0.34392	0.118814	-2.89463	0.003796	0.015007
CNAG_03024	1361.834	0.220438	0.110222	1.999942	0.045507	0.115792
CNAG_03021	34.35458	-1.10818	0.394593	-2.80842	0.004979	0.018751
CNAG_03020	746.6725	-0.23652	0.088545	-2.67117	0.007559	0.026813
CNAG_03017	1434.653	-0.17687	0.083255	-2.1245	0.033629	0.090559

CNAG_03012	53787.08	0.169298	0.078283	2.162634	0.030569	0.084387
CNAG_12229	44.35708	0.933307	0.301726	3.093224	0.00198	0.008546
CNAG_03011	100.6007	-0.60838	0.204895	-2.96924	0.002985	0.012204
CNAG_03009	674.3976	-0.22917	0.093182	-2.45935	0.013919	0.044372
CNAG_03007	2351.133	-0.80596	0.086408	-9.32733	1.09E-20	4.19E-19
CNAG_03006	123.2279	-0.44017	0.209907	-2.09696	0.035997	0.095746
CNAG_03005	140.2646	-0.59855	0.191433	-3.12668	0.001768	0.007756
CNAG_03004	1550.451	0.172176	0.080889	2.128549	0.033292	0.089961
CNAG_03002	908.3608	0.521199	0.093437	5.578084	2.43E-08	2.68E-07
CNAG_03001	2932.842	-0.37101	0.06648	-5.58077	2.39E-08	2.64E-07
CNAG_03000	53342.13	-0.28734	0.076674	-3.74751	0.000179	0.001032
CNAG_02994	10977.62	-0.29354	0.068859	-4.26285	2.02E-05	0.000142
CNAG_02993	1198.604	0.211299	0.080528	2.623923	0.008692	0.03027
CNAG_02989	1627.282	0.668088	0.074167	9.007839	2.10E-19	7.10E-18
CNAG_02986	1538.365	0.694969	0.09512	7.306226	2.75E-13	5.54E-12
CNAG_02983	1848.518	-0.38379	0.073525	-5.2199	1.79E-07	1.75E-06
CNAG_02982	2209.84	-0.67316	0.075688	-8.89388	5.90E-19	1.91E-17
CNAG_02978	229.1467	0.786054	0.1477	5.321964	1.03E-07	1.03E-06
CNAG_02977	2373.228	-0.19927	0.075466	-2.64058	0.008277	0.029039
CNAG_02975	1378.165	0.230427	0.079428	2.901098	0.003719	0.014761
CNAG_02973	976.6564	-0.21088	0.087852	-2.40041	0.016377	0.050596
CNAG_07506	1808.011	-0.27265	0.083929	-3.24864	0.00116	0.005391
CNAG_07507	868.9187	-0.24055	0.09986	-2.40884	0.016003	0.049717
CNAG_02969	679.8529	0.597766	0.103231	5.790587	7.01E-09	8.37E-08
CNAG_02968	420.3352	0.734289	0.122454	5.99646	2.02E-09	2.58E-08
CNAG_02967	1753.718	0.74947	0.090421	8.288695	1.14E-16	3.03E-15
CNAG_02961	2178.288	0.249783	0.088673	2.816893	0.004849	0.018325
CNAG_02960	404.0688	-0.72005	0.150438	-4.78633	1.70E-06	1.44E-05
CNAG_02959	1039.974	-0.47545	0.111342	-4.27018	1.95E-05	0.000139
CNAG_02958	623.3195	-0.30444	0.112617	-2.70334	0.006865	0.024687
CNAG_07509	2137.696	0.179646	0.083936	2.140275	0.032333	0.087979
CNAG_07510	4040.496	-0.20968	0.070478	-2.97509	0.002929	0.012007
CNAG_02955	659.7225	0.503867	0.112079	4.495662	6.94E-06	5.37E-05
CNAG_02953	2143.694	0.49588	0.100807	4.919121	8.69E-07	7.61E-06
CNAG_02952	1852.938	0.456471	0.075633	6.035327	1.59E-09	2.06E-08
CNAG_02951	793.6218	-0.22032	0.094074	-2.34198	0.019182	0.05753
CNAG_02948	9538.479	-0.29217	0.077469	-3.77149	0.000162	0.000946
CNAG_07511	5248.76	-0.25659	0.062493	-4.10584	4.03E-05	0.000269
CNAG_07512	610.1153	0.95452	0.115248	8.282301	1.21E-16	3.17E-15
CNAG_02944	1309.696	1.727391	0.106522	16.21633	3.87E-59	1.26E-56
CNAG_02943	19957.68	0.777394	0.065918	11.79332	4.23E-32	3.40E-30
CNAG_02942	2097.371	0.878572	0.105312	8.342596	7.27E-17	1.94E-15
CNAG_02938	8978.003	0.29043	0.066132	4.391673	1.12E-05	8.44E-05
CNAG_02937	3743.041	-0.14094	0.062678	-2.24858	0.024539	0.070484
CNAG_02935	1261.718	0.226015	0.081575	2.770641	0.005595	0.020643
CNAG_02933	700.1752	-0.53254	0.128097	-4.1573	3.22E-05	0.000219
CNAG_02928	53254.89	-0.27125	0.074905	-3.62121	0.000293	0.001617
CNAG_02927	1131.412	-0.19991	0.08096	-2.46928	0.013538	0.043402

CNAG_02925	5170.05	-0.34389	0.100503	-3.4217	0.000622	0.003162
CNAG_12238	121.4433	0.528872	0.190989	2.769125	0.005621	0.02072
CNAG_02918	10056.93	-0.68543	0.090102	-7.60726	2.80E-14	6.07E-13
CNAG_02914	1153.026	-0.22063	0.087578	-2.51927	0.01176	0.038747
CNAG_07513	1317.866	-0.3071	0.098069	-3.13147	0.001739	0.007651
CNAG_02907	1067.163	-0.26282	0.084231	-3.12018	0.001807	0.007893
CNAG_02906	1285.618	-0.17605	0.080639	-2.18321	0.029021	0.081087
CNAG_02905	2204.39	0.867928	0.071259	12.17999	3.97E-34	3.61E-32
CNAG_02903	2107.816	1.041823	0.085019	12.25403	1.60E-34	1.50E-32
CNAG_02901	2611.215	-0.16886	0.070824	-2.38429	0.017112	0.052459
CNAG_02896	4051.794	-1.09357	0.079321	-13.7868	3.06E-43	4.60E-41
CNAG_02895	1711.411	0.193412	0.074319	2.602457	0.009256	0.031817
CNAG_02894	628.9958	-0.30929	0.097404	-3.17531	0.001497	0.006717
CNAG_02892	491.3511	0.477304	0.108577	4.395982	1.10E-05	8.28E-05
CNAG_02891	2734.09	0.272945	0.073325	3.722391	0.000197	0.00113
CNAG_02890	1988.077	-0.22259	0.086084	-2.58568	0.009719	0.033091
CNAG_02889	90.63	0.513544	0.217507	2.361044	0.018224	0.055123
CNAG_07514	2744.256	-0.30125	0.081371	-3.70216	0.000214	0.001215
CNAG_02883	863.9885	0.261996	0.09456	2.770684	0.005594	0.020643
CNAG_02877	1595.014	0.856439	0.107863	7.940058	2.02E-15	4.85E-14
CNAG_02875	1227.912	-0.52332	0.082948	-6.30899	2.81E-10	4.04E-09
CNAG_02873	639.4204	0.285054	0.099315	2.870198	0.004102	0.015993
CNAG_02870	2595.684	-0.42865	0.073836	-5.80543	6.42E-09	7.70E-08
CNAG_12248	140.4698	-0.38445	0.183621	-2.09374	0.036283	0.096246
CNAG_02867	1497.7	-0.34009	0.079586	-4.27323	1.93E-05	0.000137
CNAG_12249	29.10372	0.91138	0.378507	2.407831	0.016048	0.049793
CNAG_02865	166.4979	0.497521	0.167665	2.967357	0.003004	0.012254
CNAG_02864	665.8394	1.184091	0.110266	10.73847	6.71E-27	4.23E-25
CNAG_12250	1324.977	0.308833	0.083602	3.694098	0.000221	0.001253
CNAG_02855	1669.761	-0.31404	0.087518	-3.58828	0.000333	0.00181
CNAG_07515	154.5172	0.456855	0.218023	2.09544	0.036132	0.096007
CNAG_02853	5659.685	-0.40567	0.068147	-5.95285	2.64E-09	3.34E-08
CNAG_02852	781.7599	0.55658	0.088781	6.269136	3.63E-10	5.11E-09
CNAG_12257	83.42431	-0.52539	0.223524	-2.35048	0.018749	0.056385
CNAG_02848	275.0223	-0.38728	0.132997	-2.91191	0.003592	0.014354
CNAG_02846	391.7429	0.387826	0.119295	3.250969	0.00115	0.005353
CNAG_02845	1804.597	-0.31674	0.07868	-4.02565	5.68E-05	0.00037
CNAG_12259	66.61398	1.082382	0.262735	4.119676	3.79E-05	0.000254
CNAG_02843	6126.195	0.481071	0.07069	6.805364	1.01E-11	1.71E-10
CNAG_02841	1056.314	-0.21081	0.080248	-2.62704	0.008613	0.030007
CNAG_02840	508.7701	-0.56364	0.116878	-4.82241	1.42E-06	1.21E-05
CNAG_02838	1378.483	-0.26257	0.083343	-3.15047	0.00163	0.007232
CNAG_07517	901.7415	0.32607	0.106432	3.063649	0.002187	0.00927
CNAG_07518	590.618	-0.28348	0.095499	-2.96842	0.002993	0.012224
CNAG_07519	681.5623	-0.73072	0.101668	-7.18732	6.61E-13	1.28E-11
CNAG_12261	35.95173	0.77385	0.326032	2.373543	0.017618	0.053729
CNAG_02834	793.3931	0.586138	0.091423	6.41125	1.44E-10	2.14E-09
CNAG_02833	2537.199	0.236889	0.07383	3.208577	0.001334	0.006088

CNAG_02830	5048.459	-0.84837	0.07948	-10.674	1.35E-26	8.21E-25
CNAG_02829	1300.954	-0.19209	0.080102	-2.39804	0.016483	0.050843
CNAG_02826	644.6544	-0.23571	0.095734	-2.46214	0.013811	0.044121
CNAG_02825	5341.608	-0.17289	0.064394	-2.68488	0.007256	0.025867
CNAG_07521	312.0581	0.632645	0.123354	5.128701	2.92E-07	2.78E-06
CNAG_02822	4830.221	-0.34276	0.083387	-4.1105	3.95E-05	0.000264
CNAG_02821	390.2564	-0.25819	0.113881	-2.2672	0.023378	0.067635
CNAG_02819	763.6513	0.366166	0.09005	4.066231	4.78E-05	0.000316
CNAG_02818	1612.381	0.279995	0.090917	3.079662	0.002072	0.008867
CNAG_02815	4826.023	1.178814	0.07989	14.75544	2.84E-49	5.40E-47
CNAG_02812	4180.661	0.209544	0.074931	2.796481	0.005166	0.019309
CNAG_02810	9721.295	-0.28542	0.07503	-3.80407	0.000142	0.000843
CNAG_02809	1715.239	-0.51965	0.079194	-6.56175	5.32E-11	8.22E-10
CNAG_07522	2229.698	0.507439	0.075505	6.720626	1.81E-11	3.00E-10
CNAG_02804	210.1616	-0.40956	0.144931	-2.82592	0.004715	0.017955
CNAG_02802	209.4344	0.311851	0.143019	2.180491	0.029221	0.08156
CNAG_02801	8599.758	0.188695	0.074106	2.546281	0.010888	0.036331
CNAG_02795	485.925	-0.48062	0.142833	-3.36488	0.000766	0.00377
CNAG_02792	1144.949	-0.27893	0.087764	-3.17817	0.001482	0.006666
CNAG_02791	687.8071	0.378806	0.104425	3.627539	0.000286	0.001589
CNAG_02790	1855.07	0.237734	0.081704	2.909686	0.003618	0.014427
CNAG_02783	1165.656	0.736411	0.081535	9.03184	1.69E-19	5.80E-18
CNAG_02782	1348.789	-0.18005	0.078517	-2.29311	0.021842	0.064032
CNAG_02777	12358.24	2.579655	0.184575	13.97623	2.18E-44	3.62E-42
CNAG_12269	192.7133	0.361174	0.153595	2.35147	0.018699	0.056279
CNAG_12271	24.17472	1.140298	0.447836	2.546241	0.010889	0.036331
CNAG_02775	3048.512	0.84803	0.078331	10.8262	2.59E-27	1.68E-25
CNAG_02773	2939.181	-0.49722	0.073326	-6.78088	1.19E-11	2.02E-10
CNAG_02771	991.2768	-0.22646	0.111122	-2.03795	0.041555	0.107837
CNAG_02768	163.9349	1.083247	0.177763	6.09377	1.10E-09	1.46E-08
CNAG_02765	32.49517	-0.79733	0.351548	-2.26806	0.023325	0.067544
CNAG_02763	4205.357	-0.14139	0.064071	-2.20681	0.027327	0.077381
CNAG_02758	216.0153	-0.86339	0.154549	-5.58648	2.32E-08	2.57E-07
CNAG_02757	696.4869	0.527825	0.096885	5.447968	5.09E-08	5.38E-07
CNAG_02753	4926.643	0.354819	0.075699	4.687256	2.77E-06	2.28E-05
CNAG_02752	1982.097	-0.2745	0.083205	-3.2991	0.00097	0.004621
CNAG_02751	2358.949	1.24405	0.091525	13.59245	4.44E-42	6.42E-40
CNAG_02744	699.989	-0.24488	0.10036	-2.44001	0.014687	0.046457
CNAG_02740	502.3593	-0.38184	0.109068	-3.50093	0.000464	0.002445
CNAG_02736	6492.628	-0.18074	0.062743	-2.8806	0.003969	0.015597
CNAG_02733	83.42826	0.701884	0.214435	3.273182	0.001063	0.005013
CNAG_07524	1170.987	-0.34971	0.082955	-4.2156	2.49E-05	0.000173
CNAG_02728	540.639	0.4717	0.103299	4.566341	4.96E-06	3.91E-05
CNAG_02726	4336.638	-0.2035	0.068401	-2.97505	0.002929	0.012007
CNAG_02725	3149.911	-0.25427	0.064756	-3.92661	8.62E-05	0.00054
CNAG_02721	2143.955	-0.20965	0.103338	-2.02883	0.042476	0.109617
CNAG_02720	2705.102	-0.49583	0.067747	-7.31877	2.50E-13	5.10E-12
CNAG_12278	29.67872	-0.92538	0.395796	-2.33801	0.019387	0.058078

CNAG_02718	1278.595	-0.34819	0.083359	-4.17701	2.95E-05	0.000203
CNAG_02715	956.8664	0.185622	0.087235	2.12785	0.03335	0.090033
CNAG_02712	2429.706	-0.27396	0.075571	-3.62525	0.000289	0.001598
CNAG_02710	8362.756	-0.16097	0.060476	-2.66163	0.007776	0.027515
CNAG_02708	636.4037	0.238607	0.093785	2.54419	0.010953	0.036498
CNAG_02705	179.9337	-0.6436	0.181786	-3.54044	0.000399	0.002131
CNAG_02703	2006.917	-0.28183	0.086264	-3.26707	0.001087	0.005099
CNAG_02699	1463.542	-0.35672	0.09073	-3.93163	8.44E-05	0.000532
CNAG_02698	1417.241	-0.20637	0.080111	-2.57604	0.009994	0.033821
CNAG_02697	848.0925	0.28575	0.094474	3.024633	0.002489	0.010417
CNAG_02693	797.7102	-0.58027	0.086965	-6.6725	2.51E-11	4.10E-10
CNAG_02692	3972.489	0.601417	0.084157	7.146407	8.91E-13	1.69E-11
CNAG_02691	604.6408	-0.42075	0.125009	-3.36577	0.000763	0.003761
CNAG_02690	1062.004	0.635777	0.084118	7.558121	4.09E-14	8.72E-13
CNAG_02687	2610.357	0.210328	0.068615	3.065339	0.002174	0.009227
CNAG_02686	3728.89	-0.15233	0.065248	-2.33464	0.019562	0.058471
CNAG_02685	1089.73	1.02615	0.123795	8.289131	1.14E-16	3.03E-15
CNAG_02682	1632.489	0.211906	0.101571	2.086271	0.036954	0.097627
CNAG_02679	763.5832	-0.19376	0.090628	-2.13793	0.032522	0.088322
CNAG_02678	568.0491	0.387637	0.101238	3.828977	0.000129	0.000773
CNAG_02677	3952.004	-0.14203	0.065719	-2.1611	0.030687	0.084563
CNAG_02675	3860.221	-0.2783	0.095036	-2.9284	0.003407	0.013692
CNAG_02673	6197.522	0.656687	0.073247	8.965344	3.09E-19	1.03E-17
CNAG_02672	3085.922	-0.5239	0.069463	-7.54215	4.62E-14	9.83E-13
CNAG_02667	453.824	0.515393	0.10684	4.823974	1.41E-06	1.20E-05
CNAG_02663	1195.626	0.706495	0.093895	7.524324	5.30E-14	1.12E-12
CNAG_02662	1113.138	0.35742	0.084298	4.239951	2.24E-05	0.000157
CNAG_12295	71.41533	1.195637	0.238266	5.018074	5.22E-07	4.79E-06
CNAG_12296	34.80168	0.951973	0.340768	2.793609	0.005212	0.019453
CNAG_02657	7702.972	-0.29933	0.072407	-4.13397	3.57E-05	0.00024
CNAG_02655	2185.557	0.457	0.089355	5.114461	3.15E-07	2.98E-06
CNAG_07536	971.1652	-0.64122	0.098994	-6.47741	9.33E-11	1.41E-09
CNAG_07537	687.4279	-0.22587	0.101152	-2.23299	0.02555	0.073009
CNAG_07538	1049.983	1.213079	0.093741	12.94072	2.65E-38	3.39E-36
CNAG_07540	532.2419	0.971177	0.117624	8.256624	1.50E-16	3.89E-15
CNAG_07541	1175.308	-0.19868	0.084542	-2.35002	0.018772	0.056432
CNAG_07542	175.5068	0.448396	0.15717	2.852938	0.004332	0.016702
CNAG_07544	54.50682	-0.94786	0.288343	-3.28726	0.001012	0.004805
CNAG_07545	891.41	-0.22961	0.094699	-2.4246	0.015325	0.048075
CNAG_07546	1326.666	0.18749	0.07854	2.387201	0.016977	0.052141
CNAG_07547	930.9774	0.199077	0.098119	2.028939	0.042465	0.109617
CNAG_07548	680.0482	-0.26956	0.093274	-2.88999	0.003853	0.0152
CNAG_07549	342.6896	0.446336	0.136364	3.273125	0.001064	0.005013
CNAG_07551	2476.24	0.47475	0.079589	5.965002	2.45E-09	3.10E-08
CNAG_07557	1092.645	0.203931	0.102165	1.996098	0.045923	0.116479
CNAG_07559	2932.935	0.879209	0.084507	10.40398	2.38E-25	1.34E-23
CNAG_07561	54988.21	0.384186	0.069419	5.534325	3.12E-08	3.40E-07
CNAG_07562	1461.472	-0.28548	0.087085	-3.27816	0.001045	0.004939

CNAG_07567	1287.427	-0.48943	0.086634	-5.64942	1.61E-08	1.84E-07
CNAG_07572	1177.495	-0.38373	0.083795	-4.57931	4.67E-06	3.70E-05
CNAG_02609	1522.678	0.159429	0.078489	2.03122	0.042233	0.10917
CNAG_07573	905.2858	0.167471	0.084371	1.984946	0.04715	0.118812
CNAG_02605	692.3926	-0.1947	0.094941	-2.05076	0.040291	0.104949
CNAG_02595	375.4471	-0.48823	0.129198	-3.77894	0.000157	0.000922
CNAG_02593	895.1216	0.4698	0.090236	5.206361	1.93E-07	1.87E-06
CNAG_02591	213.7167	0.767592	0.150772	5.091094	3.56E-07	3.33E-06
CNAG_02590	221.3735	-0.33921	0.139316	-2.43481	0.0149	0.047
CNAG_02588	483.3827	0.288086	0.110358	2.61048	0.009042	0.03118
CNAG_02587	801.4698	-0.88676	0.09678	-9.16263	5.06E-20	1.85E-18
CNAG_02585	2794.314	0.433447	0.075494	5.741464	9.39E-09	1.11E-07
CNAG_02583	558.5392	-0.51627	0.100417	-5.14123	2.73E-07	2.61E-06
CNAG_02581	1943.746	0.198378	0.080068	2.477631	0.013226	0.04265
CNAG_02580	1688.932	-0.19652	0.07302	-2.69127	0.007118	0.025423
CNAG_02578	992.0442	-0.19104	0.084838	-2.25183	0.024333	0.069969
CNAG_02577	3306.424	0.226025	0.074907	3.017418	0.002549	0.010628
CNAG_02576	1086.862	0.370075	0.087762	4.216826	2.48E-05	0.000172
CNAG_02573	481.5636	-0.34291	0.10664	-3.21561	0.001302	0.005958
CNAG_02568	3728.768	0.186761	0.068278	2.735288	0.006233	0.022686
CNAG_02564	979.5262	-0.31823	0.083161	-3.82673	0.00013	0.000779
CNAG_12315	63.92682	0.966102	0.266424	3.626189	0.000288	0.001594
CNAG_06880	1010.998	0.243049	0.090722	2.679044	0.007383	0.026238
CNAG_06881	1757.738	0.216571	0.078798	2.748428	0.005988	0.021899
CNAG_06882	899.716	-0.21338	0.088116	-2.42157	0.015454	0.048376
CNAG_07576	574.9583	0.576505	0.09924	5.809187	6.28E-09	7.54E-08
CNAG_06884	899.1461	0.406591	0.1192	3.411008	0.000647	0.003265
CNAG_07580	1235.278	-0.23211	0.082239	-2.82238	0.004767	0.018111
CNAG_06889	1647.547	-0.17414	0.080727	-2.15712	0.030996	0.085235
CNAG_06892	1364.027	0.175023	0.085896	2.037612	0.041589	0.107837
CNAG_06893	1485.213	0.421914	0.081149	5.19923	2.00E-07	1.94E-06
CNAG_06897	1146.162	-0.23013	0.082889	-2.77642	0.005496	0.020357
CNAG_06899	5235.737	-0.38454	0.072994	-5.2681	1.38E-07	1.37E-06
CNAG_06901	307.7398	-0.40429	0.129009	-3.13379	0.001726	0.007604
CNAG_06902	1114.303	-0.27005	0.088382	-3.05544	0.002247	0.009506
CNAG_06905	1769.345	0.21001	0.07942	2.644297	0.008186	0.028842
CNAG_06906	10758.95	-0.27705	0.066066	-4.1936	2.75E-05	0.000189
CNAG_06912	230.0502	-0.28567	0.143877	-1.98552	0.047086	0.118728
CNAG_06916	914.6969	-0.85542	0.091441	-9.35486	8.37E-21	3.27E-19
CNAG_06918	83.49238	0.592922	0.229153	2.587452	0.009669	0.032977
CNAG_06919	8685.02	-0.40314	0.071767	-5.6174	1.94E-08	2.16E-07
CNAG_06920	3953.243	-0.22756	0.071383	-3.18795	0.001433	0.006482
CNAG_06921	668.0039	-0.29569	0.113467	-2.60592	0.009163	0.03157
CNAG_06923	6632.694	0.627027	0.078173	8.021034	1.05E-15	2.56E-14
CNAG_06924	1545.042	0.720561	0.09307	7.742101	9.78E-15	2.21E-13
CNAG_06929	1749.845	-0.28082	0.083526	-3.36205	0.000774	0.003804
CNAG_06931	148.3578	-0.96055	0.168315	-5.70683	1.15E-08	1.34E-07
CNAG_07583	678.2891	0.223261	0.095577	2.335928	0.019495	0.058358

CNAG_06935	211.1937	0.50574	0.149225	3.389117	0.000701	0.003499
CNAG_06936	360.3186	1.103107	0.118647	9.297396	1.44E-20	5.48E-19
CNAG_04933	132.5097	-0.3955	0.18681	-2.1171	0.034251	0.091981
CNAG_04938	1779.796	0.804734	0.090351	8.90671	5.26E-19	1.72E-17
CNAG_07794	1710.201	0.208952	0.08797	2.375277	0.017536	0.053561
CNAG_04943	1399.81	0.500331	0.077664	6.442261	1.18E-10	1.76E-09
CNAG_04944	932.6575	0.427995	0.08399	5.095772	3.47E-07	3.27E-06
CNAG_04949	1461.06	0.185074	0.085587	2.162417	0.030586	0.084404
CNAG_04950	1895.153	-0.26128	0.077399	-3.37577	0.000736	0.003645
CNAG_04953	10708.26	0.242249	0.061308	3.951316	7.77E-05	0.000493
CNAG_04955	740.7077	-0.23939	0.109609	-2.184	0.028962	0.080983
CNAG_04956	298.4401	0.332725	0.135085	2.463081	0.013775	0.044039
CNAG_04958	2082.171	0.284582	0.091978	3.09401	0.001975	0.008528
CNAG_04959	1131.308	0.277764	0.083308	3.334176	0.000856	0.004147
CNAG_04960	161.015	0.358955	0.177182	2.025919	0.042773	0.110165
CNAG_04961	2640.223	-0.22829	0.07361	-3.10129	0.001927	0.008349
CNAG_04962	3992.073	-0.2412	0.066563	-3.62357	0.000291	0.001606
CNAG_04963	4424.22	0.155398	0.078384	1.982525	0.047421	0.119416
CNAG_07797	1333.802	-0.19263	0.08482	-2.27105	0.023144	0.067093
CNAG_04968	511.0898	0.219045	0.102419	2.138704	0.03246	0.088263
CNAG_04969	12240.71	-0.26371	0.062673	-4.20772	2.58E-05	0.000179
CNAG_04970	1582.158	0.782122	0.090343	8.657269	4.83E-18	1.42E-16
CNAG_12342	24.71968	0.906704	0.413563	2.192421	0.028349	0.079655
CNAG_04976	10771.84	-0.15914	0.07709	-2.06436	0.038984	0.102022
CNAG_04979	386.6205	-0.37906	0.117201	-3.23429	0.001219	0.005628
CNAG_04980	1125.67	-0.20522	0.082911	-2.47521	0.013316	0.04287
CNAG_04981	4314.743	1.10499	0.080112	13.79302	2.81E-43	4.30E-41
CNAG_04983	1030.255	-0.17944	0.090836	-1.97544	0.048218	0.121038
CNAG_04984	1961.876	-0.23321	0.115047	-2.02705	0.042657	0.109976
CNAG_04985	14805.23	-0.21693	0.075237	-2.88323	0.003936	0.015475
CNAG_04986	1160.86	0.198698	0.093956	2.114787	0.034448	0.092351
CNAG_04987	506.0312	0.364563	0.11626	3.135752	0.001714	0.007558
CNAG_04990	3116.71	-0.16197	0.075312	-2.15062	0.031507	0.086303
CNAG_07801	2567.609	0.266241	0.073481	3.62329	0.000291	0.001607
CNAG_04997	954.7278	-0.32651	0.087851	-3.71668	0.000202	0.001152
CNAG_04999	345.2777	0.331038	0.120596	2.745029	0.006051	0.022116
CNAG_05003	602.3496	0.304283	0.095056	3.201095	0.001369	0.006232
CNAG_05005	601.5458	0.754883	0.138987	5.431333	5.59E-08	5.87E-07
CNAG_05006	779.1432	-0.30264	0.095625	-3.16483	0.001552	0.006928
CNAG_05008	262.2668	0.395781	0.141353	2.799947	0.005111	0.01914
CNAG_05009	510.5186	-0.31075	0.134714	-2.30672	0.02107	0.062248
CNAG_05022	36.78081	1.006033	0.335633	2.997418	0.002723	0.01129
CNAG_05023	1130.735	0.179099	0.089654	1.997673	0.045752	0.116114
CNAG_05026	406.6229	-0.26275	0.111525	-2.35599	0.018473	0.05577
CNAG_05028	4300.713	-0.14665	0.069792	-2.10128	0.035616	0.094799
CNAG_05029	888.7235	0.35149	0.086998	4.040229	5.34E-05	0.00035
CNAG_05031	1955.264	1.119459	0.075742	14.78	1.97E-49	3.95E-47
CNAG_05035	999.952	0.47161	0.083401	5.654733	1.56E-08	1.79E-07

CNAG_05038	387.7382	0.34283	0.118881	2.883814	0.003929	0.015454
CNAG_05041	7307.114	0.302493	0.066094	4.57668	4.72E-06	3.73E-05
CNAG_05042	1044.369	0.480203	0.090539	5.303803	1.13E-07	1.13E-06
CNAG_05045	686.8902	-0.20894	0.092774	-2.25211	0.024315	0.069944
CNAG_07804	3129.051	0.205293	0.071308	2.878953	0.00399	0.015655
CNAG_07805	4404.804	0.291235	0.073099	3.984126	6.77E-05	0.000436
CNAG_05049	538.5547	-0.299	0.105373	-2.83753	0.004546	0.017409
CNAG_05050	3190.65	0.228671	0.07028	3.253701	0.001139	0.00531
CNAG_07807	31560.86	-0.17662	0.065535	-2.69502	0.007038	0.025185
CNAG_05055	709.963	-0.1912	0.097085	-1.96946	0.0489	0.122416
CNAG_05057	905.9663	-0.49695	0.090672	-5.48074	4.24E-08	4.53E-07
CNAG_05058	1411.315	0.179016	0.075754	2.363125	0.018122	0.054899
CNAG_05059	16533.38	0.576143	0.084864	6.789011	1.13E-11	1.92E-10
CNAG_05067	45.722	0.738967	0.29488	2.505995	0.012211	0.039938
CNAG_05069	4628.986	0.510283	0.076012	6.71321	1.90E-11	3.15E-10
CNAG_05070	9645.389	-0.32041	0.065341	-4.90369	9.41E-07	8.19E-06
CNAG_07808	2328.736	0.183596	0.081318	2.257742	0.023962	0.069028
CNAG_05076	972.9452	-0.28951	0.083795	-3.45495	0.00055	0.002841
CNAG_05077	2165.163	0.479163	0.068214	7.024408	2.15E-12	3.89E-11
CNAG_05079	90.41938	-0.74796	0.249935	-2.99263	0.002766	0.011432
CNAG_05080	4038.661	-0.16283	0.069219	-2.35247	0.018649	0.056215
CNAG_05085	1253.515	-0.5667	0.0879	-6.44715	1.14E-10	1.71E-09
CNAG_05086	679.9745	-0.40234	0.107949	-3.72711	0.000194	0.00111
CNAG_05087	38.00667	-1.08644	0.335665	-3.23668	0.001209	0.005594
CNAG_05090	2079.57	0.216985	0.071143	3.049989	0.002288	0.009656
CNAG_05095	2106.006	0.557242	0.090158	6.180745	6.38E-10	8.69E-09
CNAG_05096	1651.993	0.599581	0.075555	7.935706	2.09E-15	5.01E-14
CNAG_05097	1596.785	0.582263	0.079962	7.281793	3.29E-13	6.54E-12
CNAG_05098	1158.271	0.189519	0.08675	2.18465	0.028915	0.080907
CNAG_05101	3765.752	-0.43944	0.06651	-6.60721	3.92E-11	6.14E-10
CNAG_05105	8984.687	-0.1757	0.060593	-2.89974	0.003735	0.014802
CNAG_05106	337.8493	0.451889	0.124592	3.626952	0.000287	0.001591
CNAG_05109	3455.164	-0.23071	0.067536	-3.41612	0.000635	0.003213
CNAG_05111	3016.011	-0.2345	0.06823	-3.43682	0.000589	0.003022
CNAG_12368	104.6363	1.010448	0.197084	5.126994	2.94E-07	2.80E-06
CNAG_05118	1039.759	-0.2194	0.083097	-2.64031	0.008283	0.029039
CNAG_05119	425.0046	0.71783	0.114035	6.294831	3.08E-10	4.39E-09
CNAG_05120	2150.165	-0.18339	0.079377	-2.31032	0.02087	0.061718
CNAG_05122	1916.819	-0.21798	0.080776	-2.69861	0.006963	0.02496
CNAG_05125	2652.066	-0.3915	0.082	-4.77441	1.80E-06	1.52E-05
CNAG_07813	2001.37	-0.298	0.075152	-3.96533	7.33E-05	0.000468
CNAG_05132	10439.88	0.313847	0.08263	3.798233	0.000146	0.000861
CNAG_07815	665.1246	0.215523	0.107132	2.011754	0.044246	0.113297
CNAG_05134	1320.155	0.190163	0.076304	2.492172	0.012696	0.041285
CNAG_05138	2610.326	0.822543	0.073821	11.14238	7.80E-29	5.34E-27
CNAG_05139	1333.814	0.218846	0.095363	2.294872	0.02174	0.063783
CNAG_05140	7996.965	-0.49761	0.069558	-7.15392	8.43E-13	1.61E-11
CNAG_05145	1116.123	-0.25812	0.085998	-3.00145	0.002687	0.011148

CNAG_05147	132.2967	-0.41471	0.192826	-2.1507	0.0315	0.086303
CNAG_05150	2780.9	0.30656	0.06748	4.542965	5.55E-06	4.35E-05
CNAG_05151	1421.463	-0.39196	0.083647	-4.68586	2.79E-06	2.29E-05
CNAG_05152	2216.838	0.276439	0.070355	3.929218	8.52E-05	0.000535
CNAG_05153	1358.111	0.583145	0.087042	6.699598	2.09E-11	3.45E-10
CNAG_05155	2626.422	0.537101	0.091394	5.876752	4.18E-09	5.18E-08
CNAG_05156	1554.698	0.410592	0.076147	5.392115	6.96E-08	7.19E-07
CNAG_05157	129.6411	-0.69987	0.183549	-3.81297	0.000137	0.000816
CNAG_05158	555.116	-0.2667	0.107834	-2.4732	0.013391	0.043014
CNAG_12377	137.5893	0.482398	0.19475	2.477018	0.013249	0.042706
CNAG_05166	4477.525	-0.34849	0.082898	-4.20382	2.62E-05	0.000182
CNAG_05167	4308.248	1.195562	0.078739	15.18396	4.52E-52	1.04E-49
CNAG_05169	917.3416	0.405942	0.088455	4.589255	4.45E-06	3.55E-05
CNAG_12381	33.90345	0.736731	0.334591	2.201888	0.027673	0.078184
CNAG_05170	701.1259	-0.34382	0.097645	-3.52109	0.00043	0.002277
CNAG_05173	967.5685	0.390184	0.087157	4.476778	7.58E-06	5.83E-05
CNAG_07817	1768.445	-0.31097	0.085934	-3.61876	0.000296	0.001629
CNAG_05176	1605.701	0.214595	0.081988	2.617397	0.00886	0.030718
CNAG_05177	1103.534	-0.46094	0.09519	-4.8423	1.28E-06	1.10E-05
CNAG_05178	171.9958	0.527684	0.15309	3.446884	0.000567	0.002919
CNAG_05179	15571.52	0.215029	0.069061	3.113628	0.001848	0.008052
CNAG_07974	440.3412	0.41916	0.108622	3.858894	0.000114	0.000692
CNAG_05180	445.3923	0.279097	0.107666	2.592247	0.009535	0.032552
CNAG_05183	705.4336	0.552924	0.095415	5.794934	6.83E-09	8.17E-08
CNAG_05187	1232.865	-0.2463	0.083141	-2.96251	0.003051	0.012419
CNAG_05194	2611.688	0.443034	0.07122	6.22067	4.95E-10	6.84E-09
CNAG_05196	1457.837	-0.18187	0.077619	-2.34315	0.019122	0.057373
CNAG_05199	32174.55	0.942953	0.063371	14.87996	4.45E-50	9.64E-48
CNAG_05202	3252.115	-0.53674	0.073445	-7.30803	2.71E-13	5.48E-12
CNAG_05215	1343.298	0.198734	0.078888	2.519198	0.011762	0.038747
CNAG_05222	1382.961	-0.24682	0.084159	-2.93273	0.00336	0.013537
CNAG_05227	1093.793	-0.17473	0.087072	-2.00671	0.044781	0.114299
CNAG_05228	1450.917	-0.76461	0.090737	-8.42669	3.56E-17	9.64E-16
CNAG_05229	1002.915	0.566358	0.090151	6.282287	3.34E-10	4.72E-09
CNAG_05232	83113.67	-0.21754	0.07636	-2.84883	0.004388	0.016861
CNAG_05234	2703.301	-0.22927	0.081388	-2.81702	0.004847	0.018325
CNAG_05236	2184.484	0.370264	0.074399	4.976763	6.47E-07	5.80E-06
CNAG_05239	528.453	-0.22757	0.103877	-2.19073	0.028472	0.079954
CNAG_05242	3854.686	-0.13748	0.068909	-1.99511	0.046031	0.116557
CNAG_07975	241.2252	0.858883	0.139151	6.172307	6.73E-10	9.13E-09
CNAG_07976	57.89136	0.567793	0.273443	2.076455	0.037852	0.099594
CNAG_07822	765.0994	0.198484	0.094945	2.090515	0.036572	0.096813
CNAG_05251	793.7828	1.714329	0.113054	15.16379	6.14E-52	1.37E-49
CNAG_05252	1324.931	-0.52079	0.078328	-6.64881	2.95E-11	4.72E-10
CNAG_05253	578.8798	0.465322	0.117661	3.954749	7.66E-05	0.000488
CNAG_05254	1287.639	-0.52606	0.090196	-5.8324	5.46E-09	6.63E-08
CNAG_05258	42.82346	-0.76241	0.317877	-2.39843	0.016465	0.050809
CNAG_07823	170.1142	-0.63996	0.162189	-3.94577	7.95E-05	0.000504

CNAG_05260	3458.246	0.344833	0.069484	4.962784	6.95E-07	6.21E-06
CNAG_05262	2985.466	-0.21163	0.075104	-2.81789	0.004834	0.018287
CNAG_05263	85.43951	0.661248	0.227064	2.912163	0.003589	0.01435
CNAG_05264	208.7717	-0.58553	0.152236	-3.8462	0.00012	0.000724
CNAG_05267	2793.939	0.262249	0.07333	3.576283	0.000349	0.00189
CNAG_05268	66.95014	-0.59696	0.252316	-2.36594	0.017984	0.05461
CNAG_05269	4351.715	-0.13795	0.064161	-2.15009	0.031548	0.086326
CNAG_05270	2349.649	-0.39659	0.074748	-5.30566	1.12E-07	1.12E-06
CNAG_05273	1996.68	0.250466	0.090666	2.762504	0.005736	0.021083
CNAG_05274	903.4998	-0.36689	0.097129	-3.7774	0.000158	0.000927
CNAG_05285	3684.935	0.171999	0.073038	2.354925	0.018526	0.055887
CNAG_05286	440.0067	0.281441	0.107741	2.612191	0.008996	0.031038
CNAG_05287	658.2214	0.283389	0.097723	2.899938	0.003732	0.0148
CNAG_05290	948.066	1.377274	0.108365	12.70956	5.23E-37	6.09E-35
CNAG_05291	1420.816	-0.54233	0.109299	-4.96193	6.98E-07	6.23E-06
CNAG_05292	5265.282	0.372219	0.066895	5.564226	2.63E-08	2.89E-07
CNAG_05294	940.6769	0.218433	0.093684	2.331585	0.019723	0.058903
CNAG_05297	476.1889	0.310614	0.125181	2.481307	0.01309	0.042283
CNAG_05298	318.0931	-0.27647	0.125775	-2.19812	0.027941	0.078803
CNAG_05299	90.14263	0.628823	0.253559	2.479987	0.013139	0.042422
CNAG_05303	126.176	0.367653	0.183003	2.009006	0.044536	0.113806
CNAG_12402	23.95934	1.380445	0.405055	3.408045	0.000654	0.003294
CNAG_05304	166.5381	0.414854	0.180245	2.301604	0.021358	0.062901
CNAG_05309	3810.435	0.536049	0.073655	7.277805	3.39E-13	6.72E-12
CNAG_05310	56.92236	-1.44712	0.291776	-4.95969	7.06E-07	6.30E-06
CNAG_05312	3033.231	-0.66176	0.083825	-7.89457	2.91E-15	6.85E-14
CNAG_05313	932.0297	-0.53524	0.112027	-4.77778	1.77E-06	1.50E-05
CNAG_05315	276.4301	0.88012	0.149371	5.89219	3.81E-09	4.74E-08
CNAG_05317	521.6301	-0.60925	0.102431	-5.94791	2.72E-09	3.43E-08
CNAG_05318	594.3719	-0.30178	0.108599	-2.77882	0.005456	0.020226
CNAG_05321	170.7561	-0.38788	0.155426	-2.4956	0.012575	0.040991
CNAG_07830	714.148	0.215423	0.091183	2.362542	0.01815	0.054943
CNAG_05324	39.15003	-1.00705	0.330442	-3.04758	0.002307	0.009726
CNAG_05325	182.443	-0.53678	0.152256	-3.52554	0.000423	0.002242
CNAG_06876	461.1527	0.293192	0.104126	2.815731	0.004867	0.018374
CNAG_06875	645.1746	0.593469	0.097811	6.067484	1.30E-09	1.70E-08
CNAG_06874	698.1442	0.43823	0.097088	4.513739	6.37E-06	4.95E-05
CNAG_06873	87.74281	1.114458	0.224993	4.953298	7.30E-07	6.48E-06
CNAG_06872	48.83584	0.789081	0.281852	2.799629	0.005116	0.019149
CNAG_06871	1300.032	-0.27366	0.082608	-3.31278	0.000924	0.004433
CNAG_12410	146.3223	-0.41754	0.181535	-2.30007	0.021444	0.06308
CNAG_06868	1899.638	0.834133	0.077345	10.78463	4.07E-27	2.60E-25
CNAG_06867	668.9084	0.304458	0.109854	2.771468	0.00558	0.02061
CNAG_06866	2108.361	0.34367	0.075681	4.54103	5.60E-06	4.38E-05
CNAG_06865	971.9362	-0.46505	0.08489	-5.4782	4.30E-08	4.59E-07
CNAG_06864	2379.576	-0.2594	0.071726	-3.61654	0.000299	0.00164
CNAG_06860	2454.22	-0.18314	0.083494	-2.19349	0.028272	0.079566
CNAG_07979	1145.398	-0.18524	0.083191	-2.22674	0.025965	0.073979

CNAG_06853	218.799	0.299338	0.144412	2.072805	0.03819	0.100383
CNAG_07401	217.5578	0.323623	0.148446	2.180064	0.029253	0.081618
CNAG_06846	448.4575	0.270946	0.114163	2.373328	0.017629	0.053736
CNAG_12415	230.7369	-0.29922	0.145722	-2.0534	0.040034	0.104385
CNAG_06838	1260.051	-0.30658	0.082075	-3.73543	0.000187	0.001077
CNAG_06835	606.3847	0.751151	0.103992	7.223193	5.08E-13	9.91E-12
CNAG_06832	919.6752	-0.75861	0.084116	-9.01859	1.91E-19	6.49E-18
CNAG_06831	159.6815	-0.51632	0.168208	-3.06955	0.002144	0.009112
CNAG_06830	4672.78	-0.26563	0.062398	-4.25709	2.07E-05	0.000146
CNAG_06828	1534.709	0.385337	0.089986	4.282178	1.85E-05	0.000133
CNAG_06824	1325.068	-0.36618	0.096971	-3.77614	0.000159	0.000931
CNAG_06821	1175.33	-0.5673	0.077982	-7.27481	3.47E-13	6.85E-12
CNAG_06820	965.4402	-0.50577	0.089535	-5.64882	1.62E-08	1.84E-07
CNAG_07405	198.233	0.508251	0.149977	3.388853	0.000702	0.0035
CNAG_06818	443.0468	0.389004	0.10614	3.665007	0.000247	0.001392
CNAG_06817	546.4015	1.807398	0.129078	14.00241	1.51E-44	2.56E-42
CNAG_12420	105.0874	0.989222	0.197205	5.0162	5.27E-07	4.83E-06
CNAG_06811	21513.71	-0.20119	0.080384	-2.50287	0.012319	0.040225
CNAG_06807	289.627	-0.35184	0.134529	-2.61532	0.008914	0.030824
CNAG_06806	4596.488	0.203891	0.067561	3.017896	0.002545	0.010617
CNAG_06971	3264.594	-0.3404	0.090266	-3.7711	0.000163	0.000947
CNAG_06980	2562.227	-0.30437	0.069991	-4.34868	1.37E-05	0.000101
CNAG_07409	2796.087	-0.38015	0.078264	-4.85723	1.19E-06	1.02E-05
CNAG_07005	696.7689	0.271701	0.090722	2.994865	0.002746	0.011373
CNAG_07004	27309.86	0.861306	0.061898	13.91489	5.14E-44	8.36E-42
CNAG_07015	1317.905	-0.28916	0.092502	-3.12602	0.001772	0.007767
CNAG_07411	3388.1	-0.19513	0.093707	-2.08235	0.037311	0.098424
CNAG_01454	3590.459	-0.31048	0.090359	-3.43606	0.00059	0.003028
CNAG_01451	1164.839	0.31423	0.11599	2.709121	0.006746	0.02434
CNAG_07413	7999.756	-0.60708	0.062926	-9.64746	5.04E-22	2.16E-20
CNAG_01446	5879.527	0.969181	0.084784	11.43118	2.92E-30	2.11E-28
CNAG_01442	2350.075	-0.18259	0.078411	-2.32862	0.019879	0.059304
CNAG_01440	454.1115	0.286396	0.108412	2.641739	0.008248	0.029008
CNAG_01437	2663.192	-0.33679	0.068117	-4.9443	7.64E-07	6.77E-06
CNAG_01432	2614.233	-0.41613	0.070603	-5.89401	3.77E-09	4.69E-08
CNAG_01428	38911.13	-0.18774	0.07258	-2.58668	0.009691	0.033024
CNAG_12433	29.73471	1.211151	0.381193	3.177261	0.001487	0.006683
CNAG_01422	2086.58	0.632073	0.073785	8.566403	1.07E-17	3.01E-16
CNAG_01419	1328.89	0.488479	0.08365	5.839544	5.23E-09	6.36E-08
CNAG_01417	785.9455	0.452109	0.109453	4.130602	3.62E-05	0.000243
CNAG_01415	1449.127	-0.3351	0.109122	-3.07089	0.002134	0.009081
CNAG_01413	7738.643	-0.17888	0.061935	-2.88816	0.003875	0.015281
CNAG_01412	2156.698	0.420988	0.075786	5.554942	2.78E-08	3.04E-07
CNAG_01411	191.735	-0.67058	0.198548	-3.37743	0.000732	0.00363
CNAG_07417	118.0202	-0.66074	0.206274	-3.20322	0.001359	0.006199
CNAG_07418	819.9973	-0.20794	0.095619	-2.17466	0.029656	0.082407
CNAG_01408	1311.702	-0.38983	0.09882	-3.94481	7.99E-05	0.000506
CNAG_01404	9495.195	-0.16448	0.078239	-2.10227	0.03553	0.094601

CNAG_01403	1511.366	0.298866	0.074565	4.008131	6.12E-05	0.000397
CNAG_01399	986.8728	0.258445	0.100635	2.568131	0.010225	0.034447
CNAG_01398	3684.181	-0.42077	0.084221	-4.99601	5.85E-07	5.31E-06
CNAG_01397	4707.667	0.398275	0.074435	5.350617	8.77E-08	8.95E-07
CNAG_01387	2425.255	0.53798	0.092379	5.823617	5.76E-09	6.97E-08
CNAG_12434	198.4384	-0.48401	0.166474	-2.90744	0.003644	0.014487
CNAG_01380	893.7104	0.270456	0.08554	3.161761	0.001568	0.006985
CNAG_01379	976.4956	-0.29369	0.089012	-3.29948	0.000969	0.004618
CNAG_01377	2606.867	-0.16286	0.080491	-2.02338	0.043034	0.110765
CNAG_01376	6893.488	-0.19839	0.081833	-2.42429	0.015338	0.048075
CNAG_01375	1296.458	0.16784	0.083947	1.999354	0.04557	0.115903
CNAG_01365	936.4963	-0.2998	0.08718	-3.43885	0.000584	0.003001
CNAG_01361	6849.202	-0.20313	0.066205	-3.06816	0.002154	0.009145
CNAG_01356	663.9807	-0.25624	0.103203	-2.48287	0.013033	0.042203
CNAG_01354	4796.911	-1.14426	0.066213	-17.2814	6.50E-67	3.38E-64
CNAG_01353	479.1572	-0.7556	0.111984	-6.74745	1.50E-11	2.51E-10
CNAG_07423	696.2413	0.201605	0.094854	2.125417	0.033552	0.090445
CNAG_01352	1737.419	-0.43921	0.107449	-4.08762	4.36E-05	0.000289
CNAG_01348	1662.762	0.975076	0.092006	10.59801	3.04E-26	1.80E-24
CNAG_01344	868.5762	0.20522	0.090526	2.266981	0.023391	0.067635
CNAG_01341	1265.024	1.078515	0.099517	10.83745	2.29E-27	1.51E-25
CNAG_01340	3030.008	-0.1918	0.075263	-2.54835	0.010823	0.036174
CNAG_01337	1004.983	0.229865	0.089771	2.56058	0.01045	0.03509
CNAG_01334	2102.588	-0.32585	0.091522	-3.56039	0.00037	0.001989
CNAG_01332	39096.97	-0.19528	0.070814	-2.75768	0.005821	0.021359
CNAG_01323	10376.11	0.169097	0.077757	2.174693	0.029653	0.082407
CNAG_01317	1350.234	-0.17802	0.082106	-2.16819	0.030144	0.08342
CNAG_01310	1419.668	-0.45601	0.081903	-5.56765	2.58E-08	2.84E-07
CNAG_01307	4177.817	0.431031	0.069895	6.166866	6.97E-10	9.44E-09
CNAG_01306	565.385	-0.31318	0.124697	-2.51156	0.01202	0.039397
CNAG_01304	1328.214	-0.45384	0.082519	-5.49985	3.80E-08	4.09E-07
CNAG_01302	1434.1	0.773707	0.075543	10.2419	1.29E-24	6.97E-23
CNAG_01299	121.5552	-0.37865	0.186579	-2.02943	0.042414	0.109567
CNAG_01296	160.1438	-0.39022	0.165432	-2.35879	0.018334	0.055415
CNAG_01293	1503.151	0.208793	0.099845	2.091173	0.036513	0.09669
CNAG_01291	1141.067	0.586937	0.11007	5.332374	9.69E-08	9.82E-07
CNAG_01287	17949.38	0.215766	0.059692	3.61465	0.000301	0.001651
CNAG_01277	4437.698	-0.21901	0.066504	-3.29322	0.00099	0.004713
CNAG_01275	1414.158	0.296509	0.075401	3.932433	8.41E-05	0.000531
CNAG_01274	8243.447	-0.26276	0.064774	-4.05652	4.98E-05	0.000328
CNAG_01272	508.6443	0.725073	0.108431	6.686939	2.28E-11	3.74E-10
CNAG_01270	9149.655	-0.13088	0.060747	-2.15448	0.031203	0.085591
CNAG_07429	2017.008	-0.23533	0.073316	-3.20984	0.001328	0.006065
CNAG_01268	47.8784	1.050683	0.328436	3.199054	0.001379	0.00626
CNAG_01267	498.0027	0.447135	0.104763	4.268053	1.97E-05	0.00014
CNAG_01263	5797.257	0.335221	0.074128	4.522185	6.12E-06	4.76E-05
CNAG_01261	4968.263	0.440789	0.068142	6.46873	9.88E-11	1.49E-09
CNAG_06947	902.8547	-0.30161	0.084454	-3.57134	0.000355	0.001917

CNAG_06948	3405.662	-0.63633	0.072685	-8.7545	2.05E-18	6.27E-17
CNAG_06949	4464.795	0.246143	0.067526	3.64517	0.000267	0.001493
CNAG_01257	1014.395	-0.25853	0.091301	-2.83158	0.004632	0.017693
CNAG_01255	1639.587	0.281359	0.08301	3.389458	0.0007	0.003497
CNAG_01252	203.6246	0.584059	0.148225	3.940361	8.14E-05	0.000515
CNAG_01251	2206.67	-0.21129	0.100211	-2.10849	0.034989	0.093657
CNAG_01250	1564.179	-0.16713	0.083161	-2.00965	0.044468	0.113668
CNAG_01242	558.304	-0.42225	0.107719	-3.91996	8.86E-05	0.000552
CNAG_01236	4240.49	-0.50365	0.065985	-7.63273	2.30E-14	5.02E-13
CNAG_01234	1632.931	0.355808	0.100817	3.52924	0.000417	0.002217
CNAG_01229	1000.835	-0.43101	0.089448	-4.8185	1.45E-06	1.23E-05
CNAG_07432	163.3698	-0.48827	0.172273	-2.83429	0.004593	0.017561
CNAG_01224	53456.69	-0.22975	0.078996	-2.90841	0.003633	0.014474
CNAG_01223	325.4751	0.3865	0.130836	2.954078	0.003136	0.012727
CNAG_01221	781.4395	0.187313	0.090602	2.067427	0.038694	0.101502
CNAG_07433	1203.827	-0.28134	0.113765	-2.47298	0.013399	0.043014
CNAG_01217	2020.496	-0.22142	0.068948	-3.21139	0.001321	0.006035
CNAG_01212	1095.748	0.217836	0.08689	2.507018	0.012175	0.03984
CNAG_01208	1025.612	-0.3878	0.125881	-3.08072	0.002065	0.008844
CNAG_01207	404.6216	-0.459	0.111968	-4.0994	4.14E-05	0.000276
CNAG_07434	780.4192	0.431704	0.092161	4.684221	2.81E-06	2.31E-05
CNAG_01203	986.491	-0.1709	0.08649	-1.97601	0.048154	0.12099
CNAG_01202	673.2507	-0.3114	0.092155	-3.3791	0.000727	0.003613
CNAG_01194	24.55854	0.793559	0.395968	2.004098	0.04506	0.114804
CNAG_01189	2893.473	-0.62441	0.211271	-2.9555	0.003122	0.012675
CNAG_01187	2075.639	-0.47274	0.077241	-6.12042	9.33E-10	1.25E-08
CNAG_01185	2362.816	-0.21096	0.073688	-2.86289	0.004198	0.016299
CNAG_01184	1239.812	0.266348	0.081197	3.280273	0.001037	0.004905
CNAG_01183	1058.232	-0.32363	0.084392	-3.83481	0.000126	0.000757
CNAG_01182	8672.821	-0.14371	0.069977	-2.05365	0.04001	0.104358
CNAG_01179	2052.404	-0.50917	0.071242	-7.147	8.87E-13	1.68E-11
CNAG_01171	418.1112	-0.67232	0.112439	-5.97944	2.24E-09	2.85E-08
CNAG_01170	58945.63	-0.19475	0.073799	-2.63889	0.008318	0.029135
CNAG_01168	10321.05	-0.36972	0.067123	-5.50802	3.63E-08	3.93E-07
CNAG_01165	936.5873	-0.32118	0.112222	-2.86202	0.00421	0.016336
CNAG_01161	1306.261	-0.1822	0.079783	-2.28375	0.022386	0.065216
CNAG_07439	3329.705	-0.55115	0.067952	-8.11085	5.03E-16	1.25E-14
CNAG_01158	5811.8	-0.28735	0.061026	-4.70869	2.49E-06	2.06E-05
CNAG_01155	7051.981	0.455403	0.070505	6.459191	1.05E-10	1.59E-09
CNAG_01153	37096.71	-0.18728	0.065247	-2.87031	0.004101	0.015993
CNAG_01152	48269.75	-0.26948	0.087707	-3.07244	0.002123	0.009044
CNAG_01150	10180.07	0.339019	0.062082	5.460856	4.74E-08	5.03E-07
CNAG_01148	14453.08	-0.2388	0.076403	-3.1255	0.001775	0.007773
CNAG_01147	1923.37	0.222894	0.071037	3.137722	0.001703	0.007516
CNAG_01146	2984.077	-0.5255	0.078777	-6.67065	2.55E-11	4.14E-10
CNAG_01143	853.5342	-0.26498	0.090374	-2.93204	0.003367	0.013553
CNAG_01140	2688.374	-0.64567	0.082556	-7.82092	5.24E-15	1.20E-13
CNAG_12461	75.51489	-0.87176	0.269098	-3.23958	0.001197	0.005544

CNAG_01139	55.32781	-0.89995	0.278611	-3.23015	0.001237	0.005697
CNAG_01138	9575.89	0.360042	0.065491	5.497563	3.85E-08	4.14E-07
CNAG_01137	14100.46	1.139126	0.062562	18.20789	4.47E-74	3.17E-71
CNAG_01133	468.5856	0.226669	0.11096	2.042802	0.041072	0.106735
CNAG_01132	1379.892	-0.49117	0.083949	-5.85079	4.89E-09	6.00E-08
CNAG_01130	4017.855	-0.20316	0.073425	-2.76684	0.00566	0.020846
CNAG_01129	1598.254	-0.45719	0.080283	-5.69476	1.24E-08	1.43E-07
CNAG_01128	1587.335	0.175237	0.082969	2.112081	0.034679	0.092881
CNAG_01123	1391.663	-0.27205	0.079797	-3.40927	0.000651	0.003282
CNAG_01122	68.13906	-0.6849	0.268056	-2.55506	0.010617	0.035575
CNAG_01120	22275.08	0.605678	0.059217	10.22814	1.48E-24	7.98E-23
CNAG_12466	76.28466	-0.55472	0.228924	-2.42317	0.015386	0.048182
CNAG_01118	346.0133	1.619035	0.12624	12.82505	1.19E-37	1.45E-35
CNAG_01112	2496.325	0.341139	0.071016	4.803681	1.56E-06	1.32E-05
CNAG_01108	819.6716	-0.21547	0.098944	-2.1777	0.029429	0.081934
CNAG_01105	1401.804	0.18212	0.091525	1.989835	0.046609	0.11765
CNAG_01104	1449.88	-0.50122	0.076808	-6.52565	6.77E-11	1.04E-09
CNAG_01103	2360.435	-0.18878	0.06972	-2.70771	0.006775	0.024399
CNAG_01102	1865.046	1.035824	0.088832	11.66045	2.03E-31	1.58E-29
CNAG_01101	1323.795	-0.35135	0.120424	-2.91762	0.003527	0.01413
CNAG_01098	1954.045	-0.44244	0.078416	-5.64218	1.68E-08	1.90E-07
CNAG_01097	1526.62	0.230869	0.082438	2.800504	0.005102	0.019125
CNAG_01095	724.9723	0.24145	0.105108	2.297159	0.02161	0.063471
CNAG_01094	1828.121	-0.18631	0.073637	-2.53007	0.011404	0.037758
CNAG_01091	6582.737	-0.1296	0.060243	-2.15135	0.031449	0.086205
CNAG_01090	425.5922	1.105217	0.109837	10.06235	8.10E-24	4.08E-22
CNAG_01089	1594.199	0.54837	0.075145	7.297522	2.93E-13	5.90E-12
CNAG_01086	382.7424	0.667254	0.12308	5.421285	5.92E-08	6.18E-07
CNAG_01084	429.0033	-0.44279	0.13097	-3.38087	0.000723	0.003592
CNAG_01082	2193.215	0.373825	0.093523	3.997156	6.41E-05	0.000414
CNAG_12474	14.73703	-1.04942	0.519046	-2.02182	0.043195	0.111068
CNAG_01078	268.9968	0.454447	0.137479	3.30558	0.000948	0.004535
CNAG_01077	76.56694	1.25304	0.235394	5.323168	1.02E-07	1.03E-06
CNAG_12476	11.92371	1.213259	0.577112	2.102295	0.035527	0.094601
CNAG_01076	97.46243	1.040796	0.205695	5.059894	4.19E-07	3.89E-06
CNAG_01075	78.20411	0.686675	0.24571	2.794652	0.005196	0.0194
CNAG_01074	81.84852	1.201897	0.225798	5.322879	1.02E-07	1.03E-06
CNAG_01073	474.0832	0.224189	0.103953	2.156635	0.031034	0.085308
CNAG_01061	540.7059	1.235064	0.115498	10.69339	1.09E-26	6.72E-25
CNAG_01060	9345.746	-0.21054	0.070008	-3.00738	0.002635	0.010944
CNAG_01055	10026.59	0.715111	0.066593	10.73847	6.71E-27	4.23E-25
CNAG_01052	8512.53	1.277351	0.192214	6.645475	3.02E-11	4.80E-10
CNAG_01050	971.342	0.23034	0.090954	2.532507	0.011325	0.037513
CNAG_01047	313.0765	1.222045	0.144403	8.462724	2.61E-17	7.15E-16
CNAG_01044	1501.386	0.956956	0.075637	12.65198	1.09E-36	1.23E-34
CNAG_01043	10036.66	0.505203	0.070296	7.186779	6.63E-13	1.28E-11
CNAG_01041	247.8097	-0.32278	0.133841	-2.41167	0.01588	0.049392
CNAG_01037	528.7605	-0.21813	0.106442	-2.04927	0.040436	0.105292

CNAG_01036	579.6195	0.242048	0.102441	2.362807	0.018137	0.054925
CNAG_01035	4452.08	-0.16754	0.078017	-2.14745	0.031757	0.086807
CNAG_01032	340.0936	0.333049	0.139623	2.385348	0.017063	0.052363
CNAG_01031	3180.227	0.830144	0.090912	9.131292	6.77E-20	2.45E-18
CNAG_01029	1744.289	0.212082	0.083895	2.527957	0.011473	0.037954
CNAG_01028	599.363	0.284779	0.095405	2.984949	0.002836	0.01168
CNAG_01027	1042.955	0.441917	0.092534	4.775728	1.79E-06	1.51E-05
CNAG_01026	777.499	0.611136	0.087238	7.005376	2.46E-12	4.42E-11
CNAG_01022	690.0185	0.281495	0.098434	2.859739	0.00424	0.016413
CNAG_01021	3899.804	-0.29279	0.067952	-4.30883	1.64E-05	0.000119
CNAG_01019	26374.45	-0.35212	0.063455	-5.54918	2.87E-08	3.14E-07
CNAG_01016	1099.661	0.347691	0.083646	4.156719	3.23E-05	0.000219
CNAG_01011	485.5384	0.23987	0.11391	2.105782	0.035223	0.094041
CNAG_01010	2363.477	0.170828	0.079441	2.150377	0.031525	0.086324
CNAG_01007	1374.054	0.32507	0.09821	3.309952	0.000933	0.004476
CNAG_01005	3621.256	0.256048	0.066794	3.833381	0.000126	0.000761
CNAG_01004	1874.224	-0.95462	0.076712	-12.4442	1.50E-35	1.54E-33
CNAG_00999	918.8093	-0.24938	0.087458	-2.85136	0.004353	0.016777
CNAG_00995	4873.01	0.97974	0.089078	10.99863	3.88E-28	2.61E-26
CNAG_00993	739.256	-0.18387	0.092297	-1.99219	0.046351	0.117251
CNAG_00992	12496.18	-0.25812	0.065872	-3.91852	8.91E-05	0.000554
CNAG_00990	6563.646	0.184832	0.081606	2.264921	0.023518	0.067899
CNAG_00988	4157.636	-0.23235	0.082439	-2.81849	0.004825	0.018269
CNAG_00985	340.6848	0.242336	0.116967	2.07183	0.038281	0.100588
CNAG_00984	1273.931	1.262361	0.119527	10.56134	4.50E-26	2.60E-24
CNAG_00973	1004.577	0.191052	0.087824	2.175396	0.0296	0.082354
CNAG_00972	3669.323	0.202937	0.063917	3.175022	0.001498	0.00672
CNAG_00967	896.6043	0.358236	0.093073	3.848963	0.000119	0.000717
CNAG_07445	31526.75	0.238804	0.073927	3.230266	0.001237	0.005697
CNAG_07446	1605.492	0.248661	0.079921	3.111323	0.001863	0.008107
CNAG_00961	1397.393	0.960079	0.095299	10.07443	7.17E-24	3.68E-22
CNAG_00940	2882.418	-0.30701	0.069922	-4.39074	1.13E-05	8.46E-05
CNAG_00938	440.5637	0.44451	0.112081	3.965977	7.31E-05	0.000468
CNAG_00932	240.6116	0.376645	0.156606	2.40504	0.016171	0.050078
CNAG_00930	12852.53	-0.22808	0.064266	-3.54892	0.000387	0.002068
CNAG_00929	1214.159	0.514738	0.080431	6.399789	1.56E-10	2.30E-09
CNAG_00927	783.96	-0.1796	0.088518	-2.02893	0.042466	0.109617
CNAG_00922	832.4292	0.203199	0.088741	2.289794	0.022033	0.064408
CNAG_00921	369.137	0.352935	0.126847	2.782367	0.005396	0.020025
CNAG_00920	589.5963	0.379802	0.104386	3.638424	0.000274	0.001529
CNAG_00919	1815.226	1.109328	0.081753	13.56926	6.09E-42	8.65E-40
CNAG_00917	1229.498	0.259054	0.079686	3.250916	0.00115	0.005353
CNAG_00915	214.495	0.419831	0.166703	2.518433	0.011788	0.038815
CNAG_07448	795.1995	1.521994	0.128625	11.83281	2.64E-32	2.19E-30
CNAG_12498	209.7306	0.697903	0.152454	4.577794	4.70E-06	3.72E-05
CNAG_12499	38.56846	1.092343	0.339063	3.221654	0.001275	0.005851
CNAG_07450	162.0987	0.360924	0.169136	2.133925	0.032849	0.089012
CNAG_07982	1639.874	-0.22583	0.072931	-3.09645	0.001959	0.008472

CNAG_00906	3886.928	-0.5482	0.074984	-7.31084	2.65E-13	5.38E-12
CNAG_00905	234.0273	-0.40807	0.144559	-2.82286	0.00476	0.018093
CNAG_00904	572.1044	-0.47607	0.118137	-4.02978	5.58E-05	0.000365
CNAG_00903	608.3082	-0.64773	0.103183	-6.27751	3.44E-10	4.85E-09
CNAG_00899	1026.776	-0.73233	0.084651	-8.65112	5.10E-18	1.49E-16
CNAG_00897	3673.792	-0.17113	0.073866	-2.31671	0.020519	0.060864
CNAG_12505	9.594742	1.462319	0.663793	2.202974	0.027597	0.07803
CNAG_00895	1470.278	0.333367	0.096508	3.454297	0.000552	0.002846
CNAG_00893	1113.076	0.372729	0.093919	3.968619	7.23E-05	0.000463
CNAG_00892	308.9963	0.290372	0.121672	2.386518	0.017009	0.052217
CNAG_00891	9321.768	-0.1957	0.065429	-2.99096	0.002781	0.011489
CNAG_00886	33735.05	-0.45439	0.062919	-7.22192	5.13E-13	9.98E-12
CNAG_00884	1251.636	0.26062	0.079339	3.284897	0.00102	0.004837
CNAG_00883	447.4398	-0.31341	0.11377	-2.75477	0.005873	0.021529
CNAG_00877	1051.708	0.197667	0.079809	2.476754	0.013258	0.04272
CNAG_00875	801.6889	0.204976	0.093351	2.19575	0.02811	0.079166
CNAG_00874	4475.332	-0.23848	0.070169	-3.39869	0.000677	0.003396
CNAG_00873	474.4232	0.71811	0.114622	6.265001	3.73E-10	5.23E-09
CNAG_00871	128.2501	-0.81196	0.187214	-4.33707	1.44E-05	0.000106
CNAG_12511	30.86287	2.472362	0.403411	6.128639	8.86E-10	1.19E-08
CNAG_00866	1554.684	0.824885	0.08482	9.72512	2.36E-22	1.03E-20
CNAG_07456	132.9135	-0.65765	0.180302	-3.6475	0.000265	0.001481
CNAG_07457	59.55037	-0.62795	0.263029	-2.38737	0.01697	0.052138
CNAG_00863	96.9884	-0.70741	0.203098	-3.48312	0.000496	0.002591
CNAG_00862	774.9147	-0.24694	0.094166	-2.62234	0.008733	0.030383
CNAG_00861	249.8687	-0.32842	0.133701	-2.45639	0.014034	0.044685
CNAG_02554	388.9578	-0.2882	0.117657	-2.44948	0.014306	0.045386
CNAG_02552	330.9944	-0.43768	0.125875	-3.4771	0.000507	0.002641
CNAG_02549	764.5247	0.444945	0.090099	4.93842	7.88E-07	6.96E-06
CNAG_02548	1082.799	0.279565	0.118179	2.365618	0.018	0.054616
CNAG_02546	3496.789	-0.19871	0.076899	-2.58406	0.009765	0.033189
CNAG_02545	15013.96	0.146187	0.069131	2.11463	0.034462	0.092355
CNAG_02543	1032.139	0.410202	0.091119	4.501844	6.74E-06	5.23E-05
CNAG_02542	2618.683	1.48869	0.088325	16.85465	9.70E-64	4.20E-61
CNAG_02541	3409.298	0.623788	0.067017	9.307889	1.30E-20	4.99E-19
CNAG_02540	688.8213	-0.40105	0.093875	-4.27222	1.94E-05	0.000137
CNAG_02539	1227.624	0.258311	0.083762	3.083881	0.002043	0.008756
CNAG_02534	1246.734	-0.22123	0.079802	-2.77228	0.005567	0.020569
CNAG_02529	755.4265	0.198711	0.093932	2.115472	0.03439	0.092246
CNAG_02528	1567.652	0.60384	0.106171	5.687457	1.29E-08	1.50E-07
CNAG_02527	3830.576	0.673237	0.068603	9.813553	9.84E-23	4.47E-21
CNAG_02524	748.6166	0.484759	0.098247	4.934091	8.05E-07	7.09E-06
CNAG_02520	1426.413	-0.47371	0.07849	-6.0353	1.59E-09	2.06E-08
CNAG_02519	1640.31	0.33857	0.083235	4.067653	4.75E-05	0.000314
CNAG_02517	88.7999	0.558771	0.216857	2.576684	0.009975	0.033773
CNAG_02515	525.7487	0.706596	0.106012	6.665253	2.64E-11	4.27E-10
CNAG_02512	1030.751	-0.29785	0.087477	-3.40488	0.000662	0.003328
CNAG_02510	1276.392	0.215684	0.08896	2.42451	0.015329	0.048075

CNAG_02508	214.0494	-0.60189	0.155231	-3.87738	0.000106	0.000647
CNAG_02507	4320.903	-0.29216	0.078356	-3.72865	0.000193	0.001104
CNAG_02505	500.5902	0.62957	0.122064	5.157704	2.50E-07	2.40E-06
CNAG_02503	1409.788	-0.20811	0.084153	-2.47304	0.013397	0.043014
CNAG_02502	3183.098	-0.66038	0.067124	-9.83812	7.71E-23	3.58E-21
CNAG_02500	7144.12	-0.57215	0.065215	-8.77332	1.73E-18	5.39E-17
CNAG_02495	2249.649	-0.17485	0.069682	-2.50926	0.012099	0.039604
CNAG_02491	628.596	-0.2251	0.097067	-2.31907	0.020391	0.060543
CNAG_02490	764.3127	-0.23252	0.108508	-2.14286	0.032124	0.087542
CNAG_07625	2918.979	0.207713	0.066101	3.142365	0.001676	0.007406
CNAG_12527	38.11968	0.714934	0.318357	2.245699	0.024723	0.07096
CNAG_02479	387.6302	0.395404	0.124144	3.185056	0.001447	0.006536
CNAG_07626	170.6878	-0.35685	0.156821	-2.27552	0.022875	0.066487
CNAG_02473	776.1054	0.576334	0.0947	6.085863	1.16E-09	1.52E-08
CNAG_02469	1457.806	0.179309	0.085248	2.103373	0.035433	0.094505
CNAG_02468	2110.48	0.160393	0.070015	2.29084	0.021973	0.064295
CNAG_02467	2427.308	0.141501	0.070569	2.00514	0.044948	0.114595
CNAG_02464	621.0067	-0.27162	0.100308	-2.70783	0.006772	0.024399
CNAG_07627	1173.909	-0.2385	0.102152	-2.33475	0.019557	0.058471
CNAG_02460	469.1498	0.772833	0.13207	5.851678	4.87E-09	5.98E-08
CNAG_02457	4427.063	-0.29457	0.088208	-3.33955	0.000839	0.004078
CNAG_07628	11866.85	-0.14152	0.059913	-2.36206	0.018173	0.054993
CNAG_12536	10.18043	1.90617	0.68073	2.800183	0.005107	0.019135
CNAG_02455	793.5454	0.248978	0.102281	2.434247	0.014923	0.047035
CNAG_02454	1181.501	-0.7168	0.098023	-7.31255	2.62E-13	5.33E-12
CNAG_02453	1140.542	-0.26702	0.094743	-2.81836	0.004827	0.018269
CNAG_07629	1830.506	0.68814	0.072358	9.510201	1.90E-21	7.82E-20
CNAG_07630	1324.121	-0.71075	0.096408	-7.37233	1.68E-13	3.47E-12
CNAG_02447	1002.188	0.530851	0.081768	6.492178	8.46E-11	1.29E-09
CNAG_02445	2719.38	0.400722	0.070077	5.718354	1.08E-08	1.26E-07
CNAG_02444	521.7839	0.527213	0.115009	4.584114	4.56E-06	3.63E-05
CNAG_02443	3156.824	0.236095	0.06609	3.572313	0.000354	0.001911
CNAG_02442	534.0159	-0.40486	0.103169	-3.92429	8.70E-05	0.000544
CNAG_02438	241.9203	0.548556	0.148827	3.685868	0.000228	0.001293
CNAG_12538	51.72359	0.575237	0.274111	2.098552	0.035856	0.095405
CNAG_02437	1265.784	-0.56316	0.083497	-6.74466	1.53E-11	2.55E-10
CNAG_02435	4088.462	0.452242	0.075011	6.028979	1.65E-09	2.14E-08
CNAG_02433	923.149	0.287019	0.084008	3.416561	0.000634	0.00321
CNAG_02430	409.9636	0.29831	0.121768	2.449814	0.014293	0.045379
CNAG_02429	101.4344	-0.40988	0.20259	-2.02322	0.04305	0.11077
CNAG_02426	4298.731	-0.20724	0.065931	-3.14327	0.001671	0.00739
CNAG_02417	233.2614	0.927914	0.178974	5.184625	2.16E-07	2.10E-06
CNAG_02416	3826.16	0.191812	0.073708	2.602346	0.009259	0.031817
CNAG_02412	756.4628	-0.32822	0.092827	-3.53579	0.000407	0.002166
CNAG_07631	2467.764	0.549236	0.103921	5.285124	1.26E-07	1.25E-06
CNAG_02409	592.095	0.415205	0.103236	4.021892	5.77E-05	0.000376
CNAG_02407	1843.488	0.641388	0.078741	8.145514	3.78E-16	9.48E-15
CNAG_02406	1388.136	0.297981	0.087164	3.418624	0.000629	0.003189

CNAG_02404	6238.892	-0.28979	0.069898	-4.14593	3.38E-05	0.000229
CNAG_02403	3513.738	0.167382	0.073323	2.282787	0.022443	0.065328
CNAG_02400	2145.726	-0.21158	0.08569	-2.46918	0.013542	0.043402
CNAG_02399	6587.449	0.192491	0.068013	2.8302	0.004652	0.017744
CNAG_02398	1305.849	0.634399	0.112137	5.657341	1.54E-08	1.76E-07
CNAG_02395	1397.24	0.205302	0.078971	2.599728	0.00933	0.031991
CNAG_02392	231.3547	0.406115	0.143483	2.830406	0.004649	0.017741
CNAG_02389	287.502	-0.38398	0.132047	-2.90791	0.003639	0.014485
CNAG_02386	1190.539	1.121094	0.092663	12.09857	1.07E-33	9.53E-32
CNAG_02385	982.3982	0.490129	0.08927	5.490405	4.01E-08	4.30E-07
CNAG_02382	3245.718	-0.47687	0.070061	-6.80651	1.00E-11	1.70E-10
CNAG_07985	156.4244	-0.5488	0.183917	-2.98397	0.002845	0.011712
CNAG_07986	615.3856	-0.39604	0.1065	-3.71868	0.0002	0.001144
CNAG_02378	7860.043	-0.45091	0.067006	-6.7294	1.70E-11	2.83E-10
CNAG_02376	1396.824	0.182063	0.084092	2.165044	0.030384	0.083966
CNAG_02373	1285.016	0.215726	0.079063	2.728549	0.006361	0.02309
CNAG_02367	6050.739	-0.13208	0.063372	-2.08422	0.03714	0.098085
CNAG_02363	124.694	0.389948	0.186779	2.087754	0.03682	0.097306
CNAG_02362	844.6499	0.613772	0.08892	6.902519	5.11E-12	8.92E-11
CNAG_12554	129.1349	0.701446	0.175756	3.991032	6.58E-05	0.000424
CNAG_02361	1499.946	0.265926	0.104308	2.549432	0.01079	0.036077
CNAG_02360	1467.886	0.415243	0.076922	5.398243	6.73E-08	7.00E-07
CNAG_02356	1400.787	0.197067	0.081517	2.417489	0.015628	0.048765
CNAG_02355	799.9499	0.722103	0.096909	7.451384	9.24E-14	1.93E-12
CNAG_02351	2411.278	0.481625	0.081333	5.921629	3.19E-09	3.99E-08
CNAG_02349	337.121	0.443273	0.122817	3.609212	0.000307	0.001681
CNAG_07987	587.5234	0.377155	0.099062	3.807259	0.000141	0.000833
CNAG_02348	689.336	0.225981	0.09537	2.369527	0.017811	0.054147
CNAG_02347	175.3842	0.754826	0.169316	4.45808	8.27E-06	6.33E-05
CNAG_02343	1099.914	-0.25421	0.0794	-3.20158	0.001367	0.006227
CNAG_02338	23141.27	-0.1991	0.081629	-2.43914	0.014722	0.046515
CNAG_02337	1251.738	-0.39143	0.09609	-4.07353	4.63E-05	0.000307
CNAG_02336	414.1657	-0.38809	0.116708	-3.3253	0.000883	0.004268
CNAG_02335	12270.69	0.727238	0.075632	9.615479	6.88E-22	2.91E-20
CNAG_02334	20.53513	-0.97727	0.444569	-2.19825	0.027931	0.078803
CNAG_02331	27557.86	-0.30203	0.078983	-3.824	0.000131	0.000785
CNAG_02330	39405.38	-0.33263	0.067682	-4.91463	8.90E-07	7.77E-06
CNAG_02328	3281.981	-0.69135	0.086609	-7.9824	1.44E-15	3.46E-14
CNAG_02325	962.9515	0.437625	0.084369	5.187047	2.14E-07	2.07E-06
CNAG_02324	875.0951	-0.3561	0.095249	-3.73867	0.000185	0.001064
CNAG_02323	2329.587	-0.40796	0.081432	-5.00985	5.45E-07	4.98E-06
CNAG_12560	26.32973	-1.11759	0.429498	-2.60208	0.009266	0.031827
CNAG_02322	890.3847	-0.38513	0.102869	-3.74387	0.000181	0.001044
CNAG_02319	876.1289	0.201408	0.097736	2.060743	0.039328	0.102853
CNAG_02317	1594.343	-0.47438	0.077725	-6.10333	1.04E-09	1.38E-08
CNAG_02315	18264.68	0.283564	0.083096	3.412474	0.000644	0.00325
CNAG_02314	241.4348	0.740812	0.139894	5.295526	1.19E-07	1.18E-06
CNAG_02313	598.3791	0.480218	0.101411	4.735376	2.19E-06	1.82E-05

CNAG_02312	977.6016	0.517359	0.110769	4.670598	3.00E-06	2.45E-05
CNAG_02310	1748.718	-0.26057	0.076976	-3.38507	0.000712	0.003542
CNAG_02308	307.108	-0.39649	0.122049	-3.24859	0.00116	0.005391
CNAG_02307	648.7002	-0.25061	0.092817	-2.70003	0.006933	0.024877
CNAG_02301	8878.687	-0.51914	0.065692	-7.90269	2.73E-15	6.44E-14
CNAG_02299	858.3984	-0.34105	0.099259	-3.43591	0.000591	0.003028
CNAG_02298	2145.005	0.634459	0.100979	6.283081	3.32E-10	4.70E-09
CNAG_02292	2995.775	-0.18448	0.074981	-2.46039	0.013879	0.04428
CNAG_02290	1532.167	-0.20235	0.085041	-2.37944	0.017339	0.053022
CNAG_02286	2032.602	-0.36119	0.09267	-3.89764	9.71E-05	0.000599
CNAG_02282	1483.688	0.328436	0.078168	4.201657	2.65E-05	0.000183
CNAG_02281	993.742	-0.29164	0.087569	-3.33044	0.000867	0.004198
CNAG_07635	1906.737	-0.16002	0.077316	-2.06966	0.038485	0.10102
CNAG_07638	1254.045	0.288804	0.079765	3.620664	0.000294	0.001619
CNAG_07639	1757.702	0.459073	0.077566	5.918496	3.25E-09	4.06E-08
CNAG_07641	151.3432	0.325039	0.164311	1.978193	0.047907	0.120524
CNAG_07642	47.99989	0.581951	0.281603	2.066566	0.038775	0.101646
CNAG_02270	1605.871	-0.47015	0.075432	-6.23278	4.58E-10	6.36E-09
CNAG_02269	815.3567	0.205855	0.093784	2.194986	0.028165	0.079292
CNAG_07164	851.9811	0.716876	0.112052	6.397718	1.58E-10	2.33E-09
CNAG_02266	5065.643	0.307515	0.081328	3.781168	0.000156	0.000916
CNAG_02265	3050.842	-0.40542	0.100063	-4.05169	5.08E-05	0.000334
CNAG_02264	1250.742	0.682336	0.102856	6.633871	3.27E-11	5.18E-10
CNAG_02263	645.2181	0.906544	0.127975	7.083753	1.40E-12	2.62E-11
CNAG_02260	2144.418	-0.3099	0.071539	-4.33187	1.48E-05	0.000108
CNAG_02259	877.2086	-0.27893	0.10226	-2.72761	0.00638	0.023146
CNAG_02255	81.00185	-0.45342	0.220704	-2.05444	0.039933	0.104191
CNAG_02254	45.63986	-1.32662	0.306322	-4.3308	1.49E-05	0.000109
CNAG_12570	42.41651	-0.92062	0.309526	-2.97429	0.002937	0.01203
CNAG_02240	2407.165	-0.26435	0.092656	-2.85307	0.00433	0.016702
CNAG_02239	4481.58	-0.28644	0.063328	-4.52314	6.09E-06	4.75E-05
CNAG_02237	4512.826	-0.17896	0.074714	-2.39522	0.016611	0.051115
CNAG_02236	2216.072	-0.34822	0.079973	-4.35427	1.34E-05	9.89E-05
CNAG_02235	789.053	-0.2218	0.092571	-2.39597	0.016576	0.05103
CNAG_02234	51404.45	-0.21482	0.075252	-2.85464	0.004309	0.016637
CNAG_02230	4534.038	0.815188	0.092021	8.858697	8.10E-19	2.59E-17
CNAG_02226	194.7955	0.511968	0.14927	3.429815	0.000604	0.003083
CNAG_02222	1023.825	-0.18583	0.082838	-2.24331	0.024877	0.071349
CNAG_02220	1381.264	-0.57022	0.085553	-6.66504	2.65E-11	4.27E-10
CNAG_02215	580.9516	-0.55832	0.102436	-5.45044	5.02E-08	5.32E-07
CNAG_02214	256.1545	-0.50576	0.144579	-3.49817	0.000468	0.002467
CNAG_02212	13613.57	-0.16259	0.062368	-2.60693	0.009136	0.031491
CNAG_02211	482.441	0.403929	0.106817	3.781504	0.000156	0.000915
CNAG_02209	9034.855	-0.22752	0.067783	-3.35655	0.000789	0.003863
CNAG_02208	5545.647	0.287138	0.061444	4.673166	2.97E-06	2.42E-05
CNAG_02207	382.9895	-0.37845	0.120805	-3.1327	0.001732	0.007628
CNAG_02205	490.8291	0.336656	0.104019	3.236491	0.00121	0.005595
CNAG_02199	331.2246	0.327482	0.119518	2.740022	0.006143	0.022425

CNAG_02191	573.5862	-0.29754	0.101887	-2.9203	0.003497	0.014024
CNAG_02190	832.7288	0.848452	0.08616	9.847438	7.03E-23	3.31E-21
CNAG_02189	4535.793	0.221479	0.082246	2.692898	0.007083	0.025311
CNAG_02188	1220.254	0.976406	0.086195	11.32783	9.55E-30	6.72E-28
CNAG_02185	961.5596	-0.44042	0.08691	-5.06755	4.03E-07	3.74E-06
CNAG_02182	2522.66	1.296244	0.083332	15.55516	1.47E-54	3.95E-52
CNAG_02179	1817.523	0.263306	0.074087	3.554	0.000379	0.002031
CNAG_02176	1748.464	-0.35201	0.099044	-3.55409	0.000379	0.002031
CNAG_02169	254.5684	0.49318	0.170356	2.894988	0.003792	0.014998
CNAG_02165	1434.808	0.302959	0.081208	3.730652	0.000191	0.001096
CNAG_02164	1154.912	0.613449	0.092787	6.611393	3.81E-11	5.98E-10
CNAG_02163	394.0101	-0.35558	0.119261	-2.98156	0.002868	0.011785
CNAG_02162	878.3634	-0.29398	0.09517	-3.08905	0.002008	0.008639
CNAG_02159	378.379	-0.34213	0.121168	-2.82365	0.004748	0.018066
CNAG_07647	1290.323	0.318247	0.085082	3.740478	0.000184	0.001057
CNAG_12585	78.52839	-0.65609	0.231734	-2.83121	0.004637	0.017705
CNAG_02154	862.9533	-0.25585	0.085857	-2.97995	0.002883	0.011841
CNAG_02153	2404.365	-0.28241	0.072038	-3.92025	8.85E-05	0.000552
CNAG_02144	52882.16	-0.21858	0.063722	-3.43027	0.000603	0.003081
CNAG_02143	586.9729	-0.47727	0.102257	-4.66734	3.05E-06	2.48E-05
CNAG_02140	4476.387	0.132688	0.064217	2.066238	0.038806	0.101682
CNAG_12586	62.74024	0.698966	0.256938	2.720372	0.006521	0.023603
CNAG_02134	3223.929	-0.18719	0.072822	-2.57051	0.010155	0.034263
CNAG_02133	6386.399	0.285299	0.072338	3.943952	8.01E-05	0.000507
CNAG_02131	1111.878	0.328216	0.084465	3.885804	0.000102	0.000628
CNAG_02129	20051.59	1.156025	0.078279	14.76809	2.35E-49	4.59E-47
CNAG_02128	9576.167	-0.33658	0.06738	-4.99526	5.88E-07	5.32E-06
CNAG_02127	890.3864	0.229278	0.091935	2.493927	0.012634	0.041132
CNAG_02124	844.7909	0.27006	0.092802	2.910057	0.003614	0.014418
CNAG_02122	705.4632	-0.43482	0.093406	-4.65515	3.24E-06	2.62E-05
CNAG_02120	2583.417	-0.24213	0.075808	-3.19405	0.001403	0.006358
CNAG_02116	493.508	0.624455	0.108281	5.766987	8.07E-09	9.56E-08
CNAG_02115	1827.522	-0.34916	0.0727	-4.80279	1.56E-06	1.33E-05
CNAG_02111	1063.627	-0.2963	0.081363	-3.64173	0.000271	0.001511
CNAG_02110	2869.773	-0.18853	0.067731	-2.78359	0.005376	0.019969
CNAG_02107	598.8872	0.232506	0.101139	2.298866	0.021513	0.063233
CNAG_02106	1730.963	0.490804	0.078072	6.286573	3.25E-10	4.61E-09
CNAG_02105	260.293	0.315477	0.143879	2.192649	0.028333	0.079655
CNAG_02104	548.2765	-0.50604	0.105873	-4.77971	1.76E-06	1.48E-05
CNAG_12593	93.14421	0.536832	0.209723	2.559719	0.010476	0.035147
CNAG_02101	571.9663	-0.21498	0.102004	-2.10757	0.035068	0.093691
CNAG_02100	13098.71	0.181897	0.063206	2.877839	0.004004	0.015702
CNAG_02096	322.4905	0.288228	0.124428	2.31642	0.020535	0.060865
CNAG_02091	13537.5	-0.19878	0.069298	-2.86851	0.004124	0.01606
CNAG_02089	518.5622	0.380031	0.10973	3.46334	0.000534	0.002764
CNAG_02088	1260.637	-0.15876	0.076278	-2.08127	0.037409	0.098561
CNAG_02087	146.3367	0.38855	0.170845	2.274286	0.022949	0.066676
CNAG_02085	900.8593	0.371679	0.096996	3.831906	0.000127	0.000764

CNAG_02084	3980.294	-0.44648	0.066832	-6.68068	2.38E-11	3.89E-10
CNAG_02083	181.3465	-1.31559	0.158573	-8.29644	1.07E-16	2.86E-15
CNAG_02081	3145.666	-0.17396	0.074214	-2.34397	0.01908	0.057291
CNAG_02077	544.2306	-0.21067	0.105587	-1.99521	0.04602	0.116557
CNAG_02076	1558.739	-0.21411	0.079265	-2.70119	0.006909	0.024813
CNAG_02074	2447.975	0.369951	0.071511	5.173374	2.30E-07	2.22E-06
CNAG_02073	1260.055	0.213634	0.076185	2.804151	0.005045	0.018955
CNAG_02072	1003.271	0.51387	0.091463	5.618302	1.93E-08	2.16E-07
CNAG_02071	2370.468	0.16323	0.082637	1.975271	0.048237	0.121043
CNAG_02070	6391.391	1.069469	0.092667	11.54095	8.20E-31	6.10E-29
CNAG_02069	1774.233	0.710899	0.093195	7.628057	2.38E-14	5.20E-13
CNAG_02068	1083.777	-0.23458	0.084114	-2.78881	0.00529	0.019725
CNAG_02064	3383.505	-0.22568	0.094782	-2.38102	0.017265	0.052837
CNAG_02063	1867.336	0.234632	0.089596	2.618776	0.008825	0.030609
CNAG_02062	508.4946	-0.23445	0.109577	-2.13955	0.032391	0.088108
CNAG_02061	390.4245	0.269202	0.11644	2.311944	0.020781	0.061522
CNAG_12600	426.1867	0.742344	0.134852	5.504886	3.69E-08	4.00E-07
CNAG_02058	46.18096	0.865983	0.297491	2.910953	0.003603	0.014391
CNAG_02057	993.1108	0.340239	0.090248	3.770065	0.000163	0.00095
CNAG_02055	965.9074	0.316575	0.083084	3.810305	0.000139	0.000823
CNAG_02054	654.5754	-0.26816	0.104472	-2.56679	0.010265	0.034543
CNAG_02050	609.0284	-0.25018	0.110432	-2.26546	0.023484	0.067853
CNAG_02048	748.8377	0.322541	0.09063	3.558869	0.000372	0.001999
CNAG_02047	926.577	0.678949	0.088486	7.672913	1.68E-14	3.72E-13
CNAG_02045	93.70904	-0.63655	0.221839	-2.86945	0.004112	0.016021
CNAG_02043	165.6226	-0.92614	0.163644	-5.65947	1.52E-08	1.74E-07
CNAG_02041	2003.565	1.167169	0.100686	11.59221	4.51E-31	3.45E-29
CNAG_02040	140.898	-0.75932	0.196272	-3.86873	0.000109	0.000667
CNAG_02039	68.68623	0.623483	0.240503	2.59241	0.009531	0.032552
CNAG_02038	2950.996	0.65589	0.07336	8.940749	3.87E-19	1.28E-17
CNAG_02037	3112.938	0.162281	0.079737	2.03519	0.041832	0.108277
CNAG_02035	16786.99	0.35149	0.067187	5.231535	1.68E-07	1.65E-06
CNAG_02028	2011.45	0.713041	0.074016	9.633656	5.76E-22	2.46E-20
CNAG_02025	1423.796	-0.37794	0.075599	-4.99921	5.76E-07	5.24E-06
CNAG_02024	1600.42	-0.29299	0.097198	-3.01433	0.002575	0.01072
CNAG_02023	139.8836	0.631054	0.169625	3.720296	0.000199	0.001138
CNAG_02022	2089.792	0.218706	0.074626	2.930679	0.003382	0.013599
CNAG_02020	1570.867	0.218599	0.085186	2.566132	0.010284	0.034593
CNAG_07651	1009.974	-0.49463	0.084421	-5.85906	4.65E-09	5.73E-08
CNAG_06524	180.7634	-0.66581	0.204003	-3.26374	0.0011	0.005151
CNAG_06526	2500.607	0.14881	0.074962	1.985128	0.04713	0.1188
CNAG_06529	73.14392	-0.49765	0.230096	-2.16277	0.030559	0.084387
CNAG_06530	508.7547	-0.34517	0.111157	-3.10528	0.001901	0.008256
CNAG_06532	640.8504	0.328346	0.108075	3.03812	0.002381	0.009983
CNAG_06533	1493.405	0.597956	0.082608	7.238457	4.54E-13	8.92E-12
CNAG_06534	7160.439	-0.25024	0.066137	-3.78368	0.000155	0.000908
CNAG_06535	2392.762	-0.64552	0.079108	-8.15996	3.35E-16	8.44E-15
CNAG_06536	306.985	-0.42317	0.131592	-3.2158	0.001301	0.005958

CNAG_06540	125.8116	0.437386	0.180675	2.42084	0.015485	0.048434
CNAG_06541	7050.335	0.817709	0.078539	10.41154	2.20E-25	1.24E-23
CNAG_06542	1577.414	-0.15102	0.074412	-2.02948	0.042409	0.109567
CNAG_06545	4770.042	-0.24446	0.062953	-3.88323	0.000103	0.000634
CNAG_06546	42.99951	-0.71091	0.318439	-2.23248	0.025583	0.073079
CNAG_07657	1343.008	-0.77642	0.085857	-9.04318	1.52E-19	5.30E-18
CNAG_12613	9.183343	1.536503	0.693037	2.217056	0.026619	0.075541
CNAG_06550	2972.414	-0.20492	0.096618	-2.1209	0.03393	0.09125
CNAG_06552	1584.69	0.250894	0.078423	3.199245	0.001378	0.006259
CNAG_06553	1030.977	0.415336	0.083775	4.957759	7.13E-07	6.34E-06
CNAG_06554	630.7109	0.471259	0.099584	4.732268	2.22E-06	1.85E-05
CNAG_06555	1281.277	0.266281	0.076371	3.486678	0.000489	0.00256
CNAG_06556	61.63709	1.280426	0.28243	4.533599	5.80E-06	4.53E-05
CNAG_06557	56.89858	1.308075	0.267975	4.881327	1.05E-06	9.12E-06
CNAG_06559	642.0318	0.553941	0.102866	5.385078	7.24E-08	7.47E-07
CNAG_06560	1059.686	0.465648	0.090606	5.139245	2.76E-07	2.63E-06
CNAG_06567	1593.101	0.246721	0.087925	2.806048	0.005015	0.018862
CNAG_07658	30.5975	0.901755	0.362005	2.491	0.012738	0.041404
CNAG_12616	115.7941	0.586579	0.192035	3.054537	0.002254	0.009529
CNAG_06569	963.2499	0.402591	0.092512	4.351756	1.35E-05	1.00E-04
CNAG_06570	459.7296	0.231448	0.105118	2.201803	0.027679	0.078184
CNAG_07660	13900.43	0.661613	0.075768	8.732074	2.50E-18	7.62E-17
CNAG_06576	4231.534	1.991892	0.099067	20.10647	6.48E-90	1.01E-86
CNAG_06577	1210.814	1.862925	0.113585	16.40109	1.88E-60	6.66E-58
CNAG_06578	817.5768	0.807999	0.088044	9.177267	4.42E-20	1.62E-18
CNAG_07989	879.1577	-0.22981	0.090034	-2.55243	0.010698	0.035815
CNAG_12622	60.7824	0.957376	0.268531	3.565229	0.000364	0.001957
CNAG_06583	363.4116	1.109171	0.143626	7.722635	1.14E-14	2.56E-13
CNAG_06584	764.7529	0.180609	0.087572	2.062415	0.039168	0.10247
CNAG_12623	24.56038	0.878721	0.40867	2.150199	0.031539	0.086326
CNAG_06587	828.9179	-0.21741	0.093124	-2.33463	0.019563	0.058471
CNAG_06589	922.6184	0.330248	0.090533	3.647799	0.000264	0.001481
CNAG_06591	4236.808	-0.45181	0.112732	-4.00778	6.13E-05	0.000398
CNAG_06594	6487.325	0.261083	0.070293	3.714215	0.000204	0.001162
CNAG_06595	279.5733	-0.54976	0.180466	-3.04636	0.002316	0.009755
CNAG_06596	202.6582	-0.37299	0.162514	-2.2951	0.021727	0.06378
CNAG_06597	2087.503	-0.31054	0.070508	-4.40437	1.06E-05	7.98E-05
CNAG_06598	48.88767	-1.00183	0.323983	-3.09225	0.001986	0.008565
CNAG_06599	878.4672	-0.21987	0.094916	-2.31649	0.020532	0.060865
CNAG_12627	104.6776	0.460991	0.19851	2.322256	0.020219	0.060134
CNAG_06602	2031.225	0.783212	0.093287	8.395761	4.63E-17	1.25E-15
CNAG_06603	1881.633	-0.16713	0.084055	-1.98836	0.046772	0.118012
CNAG_06605	62280.5	-0.20584	0.077806	-2.6455	0.008157	0.028765
CNAG_06606	2124.936	0.169016	0.080334	2.103917	0.035386	0.09441
CNAG_06608	1850.697	0.304525	0.07353	4.141497	3.45E-05	0.000233
CNAG_12629	113.334	-0.52224	0.193266	-2.70219	0.006888	0.024762
CNAG_06610	186.9565	-0.35095	0.153635	-2.2843	0.022354	0.065165
CNAG_06611	2492.229	-0.49448	0.194323	-2.54461	0.01094	0.03647

CNAG_06612	1909.827	0.215038	0.077637	2.769769	0.00561	0.020689
CNAG_12632	53.28428	0.548853	0.266576	2.058899	0.039504	0.103279
CNAG_06616	1106.982	0.400016	0.091676	4.363372	1.28E-05	9.51E-05
CNAG_06621	6197.849	0.61271	0.070698	8.666628	4.45E-18	1.32E-16
CNAG_06622	179.2671	1.189856	0.163294	7.286606	3.18E-13	6.36E-12
CNAG_06624	1609.716	0.406305	0.083485	4.866787	1.13E-06	9.79E-06
CNAG_06626	4064.963	-0.44313	0.06686	-6.6278	3.41E-11	5.37E-10
CNAG_06628	2023.88	-0.2787	0.071297	-3.90898	9.27E-05	0.000574
CNAG_06629	21.76639	-0.89333	0.43144	-2.07058	0.038398	0.100828
CNAG_06630	4852.554	0.293614	0.066231	4.4332	9.28E-06	7.04E-05
CNAG_06632	1634.807	0.502873	0.078877	6.375445	1.82E-10	2.67E-09
CNAG_06633	42259.19	-0.26637	0.069608	-3.82671	0.00013	0.000779
CNAG_06635	3476.901	-0.18039	0.083269	-2.16633	0.030286	0.083723
CNAG_06638	4216.584	-0.54636	0.090661	-6.02642	1.68E-09	2.17E-08
CNAG_12642	41.11419	0.737555	0.305528	2.414033	0.015777	0.049114
CNAG_06640	3439.61	0.391469	0.067308	5.816055	6.03E-09	7.26E-08
CNAG_06641	1800.465	-0.39042	0.074287	-5.25556	1.48E-07	1.45E-06
CNAG_06643	557.3201	-0.2188	0.100075	-2.18639	0.028787	0.080637
CNAG_06644	3129.556	-0.45019	0.079921	-5.63292	1.77E-08	2.00E-07
CNAG_06645	2017.142	-0.41398	0.081848	-5.05786	4.24E-07	3.92E-06
CNAG_06646	15247.18	0.181557	0.058246	3.11705	0.001827	0.007968
CNAG_06648	2272.497	-0.13417	0.067998	-1.97317	0.048476	0.121563
CNAG_06656	1685.008	-0.26942	0.087588	-3.07596	0.002098	0.008958
CNAG_06657	406.992	0.545707	0.115254	4.734803	2.19E-06	1.82E-05
CNAG_06660	822.5904	-0.268	0.093792	-2.85737	0.004272	0.016511
CNAG_06661	1765.996	-0.15579	0.073186	-2.12861	0.033286	0.089961
CNAG_06663	8132.698	0.283107	0.073987	3.826431	0.00013	0.000779
CNAG_06664	807.7847	0.25703	0.087794	2.927639	0.003415	0.013711
CNAG_06668	2415.633	1.156094	0.087025	13.28469	2.84E-40	3.96E-38
CNAG_06675	1263.426	-0.28247	0.084863	-3.32853	0.000873	0.004224
CNAG_12649	39.81374	-0.64415	0.319045	-2.01898	0.043489	0.111651
CNAG_06679	2912.263	-0.19687	0.072696	-2.70817	0.006766	0.024387
CNAG_06680	854.3355	0.181293	0.086675	2.091635	0.036471	0.096645
CNAG_07665	4070.482	-0.19851	0.081023	-2.45008	0.014282	0.045364
CNAG_06684	503.1973	-0.76713	0.109381	-7.01331	2.33E-12	4.20E-11
CNAG_06688	4819.212	0.334444	0.061141	5.470035	4.50E-08	4.80E-07
CNAG_06689	2033.222	-0.37145	0.069535	-5.34195	9.20E-08	9.37E-07
CNAG_06695	430.2418	0.289109	0.117143	2.467994	0.013587	0.043493
CNAG_06697	965.8697	-0.36849	0.113971	-3.23316	0.001224	0.005647
CNAG_06698	1662.213	0.297861	0.087711	3.395927	0.000684	0.003428
CNAG_12655	106.0985	-0.62732	0.198874	-3.15435	0.001609	0.007145
CNAG_05673	1313.232	-0.31178	0.090812	-3.43327	0.000596	0.00305
CNAG_05675	443.1445	-0.35796	0.112368	-3.18563	0.001444	0.006527
CNAG_05676	334.6748	0.272464	0.119598	2.278167	0.022717	0.066051
CNAG_05678	780.6006	-0.31916	0.08961	-3.56162	0.000369	0.001981
CNAG_07667	5908.232	-0.24251	0.075762	-3.201	0.00137	0.006232
CNAG_05682	6552.293	0.600362	0.07164	8.38021	5.28E-17	1.42E-15
CNAG_05683	339.4589	0.886508	0.12174	7.281966	3.29E-13	6.54E-12

CNAG_05684	2047.71	0.294889	0.070433	4.186814	2.83E-05	0.000194
CNAG_05685	1035.257	0.183813	0.084676	2.170788	0.029947	0.082963
CNAG_05686	810.1377	0.822931	0.087797	9.373158	7.04E-21	2.79E-19
CNAG_05687	1395.599	-0.3542	0.097607	-3.62879	0.000285	0.001584
CNAG_05695	3412.075	-0.41094	0.094894	-4.33048	1.49E-05	0.000109
CNAG_05697	862.1686	-0.21351	0.090272	-2.36518	0.018021	0.054659
CNAG_05698	1043.18	0.303105	0.087029	3.482812	0.000496	0.002592
CNAG_05699	930.9575	0.321935	0.108546	2.965883	0.003018	0.0123
CNAG_05707	688.5251	0.34364	0.101459	3.38699	0.000707	0.003519
CNAG_05708	1449.01	-0.23002	0.090217	-2.5496	0.010785	0.036076
CNAG_05711	919.9199	0.205225	0.095227	2.155117	0.031153	0.085514
CNAG_05712	3642.975	-0.19964	0.090857	-2.19728	0.028001	0.078944
CNAG_05714	1441.683	0.211723	0.076283	2.775488	0.005512	0.020396
CNAG_05716	645.2976	-0.34399	0.102853	-3.34448	0.000824	0.004011
CNAG_05717	348.7784	-0.45674	0.126188	-3.61948	0.000295	0.001626
CNAG_05719	841.3232	-0.2563	0.093807	-2.73223	0.006291	0.022845
CNAG_05720	1524.443	-0.21153	0.076219	-2.7753	0.005515	0.020398
CNAG_05721	880.1921	-0.31032	0.095364	-3.25408	0.001138	0.005306
CNAG_05722	3275.097	-0.28838	0.112297	-2.56805	0.010227	0.034447
CNAG_05724	1113.083	0.362798	0.083868	4.325833	1.52E-05	0.000111
CNAG_05725	49189.8	-0.17046	0.066957	-2.54579	0.010903	0.036363
CNAG_05731	1154.365	-0.17617	0.081532	-2.1608	0.030711	0.084599
CNAG_05736	105.6983	0.511122	0.201102	2.541608	0.011034	0.036722
CNAG_05737	1555.348	0.261255	0.08008	3.262437	0.001105	0.005171
CNAG_05742	1400.167	-0.19125	0.079403	-2.40855	0.016016	0.049737
CNAG_05743	545.8994	0.262397	0.100331	2.615305	0.008915	0.030824
CNAG_05745	4698.539	-0.20805	0.079959	-2.60192	0.00927	0.031828
CNAG_05749	780.219	-0.20431	0.096149	-2.12492	0.033593	0.090526
CNAG_05750	75053.74	0.177402	0.066133	2.682493	0.007308	0.026028
CNAG_05752	2354.429	-0.22894	0.077677	-2.94728	0.003206	0.012976
CNAG_07671	569.9585	0.367868	0.12204	3.014332	0.002575	0.01072
CNAG_07672	1521.762	-0.5199	0.08484	-6.12804	8.90E-10	1.19E-08
CNAG_05755	2418.759	-0.54208	0.069273	-7.82525	5.07E-15	1.17E-13
CNAG_05756	1476.693	0.22628	0.0826	2.739486	0.006154	0.022446
CNAG_05757	839.0873	-0.96131	0.109702	-8.76293	1.90E-18	5.87E-17
CNAG_05759	12091.17	0.19096	0.061154	3.122631	0.001792	0.007836
CNAG_05760	16.6468	-1.09706	0.504495	-2.17458	0.029662	0.082407
CNAG_12676	301.5574	-0.39107	0.136734	-2.86005	0.004236	0.016405
CNAG_05762	62409.78	-0.22036	0.072903	-3.02266	0.002506	0.010462
CNAG_05763	344.9956	-0.52288	0.118212	-4.42322	9.72E-06	7.37E-05
CNAG_05765	1402.834	-0.2575	0.078782	-3.2685	0.001081	0.005077
CNAG_05768	1718.304	0.160578	0.073039	2.198533	0.027911	0.078777
CNAG_05769	1034.339	0.173059	0.080954	2.137741	0.032538	0.088322
CNAG_05770	3805.151	-0.17039	0.065633	-2.59611	0.009429	0.032289
CNAG_05771	933.132	-0.31202	0.122059	-2.55632	0.010579	0.035462
CNAG_05773	1141.857	0.190784	0.083496	2.284937	0.022317	0.065111
CNAG_12677	243.4698	-0.49929	0.143482	-3.47979	0.000502	0.002616
CNAG_05774	1021.767	0.503529	0.097135	5.183789	2.17E-07	2.10E-06

CNAG_05777	338.495	-0.26886	0.129117	-2.08228	0.037317	0.098424
CNAG_05778	482.1304	-0.57275	0.114486	-5.00283	5.65E-07	5.15E-06
CNAG_05781	574.492	-0.22026	0.103217	-2.13394	0.032848	0.089012
CNAG_05783	3840.613	0.197257	0.063066	3.127807	0.001761	0.00773
CNAG_05784	364.4681	0.406498	0.120635	3.36964	0.000753	0.003718
CNAG_05789	1615.363	-0.59886	0.079059	-7.57485	3.60E-14	7.73E-13
CNAG_05791	2117.21	-0.21111	0.095499	-2.21057	0.027065	0.076695
CNAG_05792	4279.627	-0.24557	0.070997	-3.45888	0.000542	0.002805
CNAG_05794	640.0735	0.311108	0.099622	3.122883	0.001791	0.007834
CNAG_05799	6204.926	0.317671	0.063096	5.034755	4.78E-07	4.42E-06
CNAG_05800	27594.15	-0.18685	0.077491	-2.41125	0.015898	0.04943
CNAG_05801	470.8109	-0.39402	0.110147	-3.57718	0.000347	0.001885
CNAG_05803	1454.944	-0.16002	0.074102	-2.1595	0.030812	0.084817
CNAG_05808	852.5732	0.395933	0.092533	4.27881	1.88E-05	0.000135
CNAG_05809	2798.106	0.431173	0.072478	5.949021	2.70E-09	3.41E-08
CNAG_05811	730.9499	-0.20873	0.094401	-2.21111	0.027028	0.076645
CNAG_05814	38075.78	-0.18453	0.081422	-2.26636	0.02343	0.06772
CNAG_05816	1364.318	-0.22115	0.074782	-2.95732	0.003103	0.012614
CNAG_05817	6055.073	-0.25628	0.066805	-3.83627	0.000125	0.000753
CNAG_05820	368.2741	0.395285	0.118168	3.34512	0.000822	0.004004
CNAG_05826	6422.358	-0.20393	0.062237	-3.27672	0.00105	0.004961
CNAG_05828	2179.862	-0.50232	0.082037	-6.12304	9.18E-10	1.23E-08
CNAG_05830	1374.453	-0.18569	0.088653	-2.09454	0.036211	0.096121
CNAG_12684	106.3528	-0.67981	0.201677	-3.37078	0.00075	0.003705
CNAG_05831	309.0704	-0.41642	0.123872	-3.36169	0.000775	0.003807
CNAG_05833	47.40753	-0.74629	0.302322	-2.46851	0.013568	0.043448
CNAG_05836	1725.035	0.491572	0.081716	6.015584	1.79E-09	2.31E-08
CNAG_05839	10373.87	0.422722	0.074918	5.642463	1.68E-08	1.90E-07
CNAG_05840	891.6908	0.379311	0.09553	3.970594	7.17E-05	0.00046
CNAG_05846	1220.475	-0.30869	0.086525	-3.56767	0.00036	0.001941
CNAG_05847	5982.212	-0.41871	0.074391	-5.62854	1.82E-08	2.04E-07
CNAG_05848	5640.607	-0.33048	0.061239	-5.3965	6.80E-08	7.05E-07
CNAG_07676	13395.66	-0.3174	0.071244	-4.45515	8.38E-06	6.41E-05
CNAG_07677	380.7608	0.527111	0.114233	4.614345	3.94E-06	3.17E-05
CNAG_05854	656.6178	-0.57537	0.112797	-5.10089	3.38E-07	3.19E-06
CNAG_07679	1802.942	-0.17642	0.071667	-2.46166	0.013829	0.044159
CNAG_05862	625.0716	0.214728	0.102128	2.102541	0.035506	0.094601
CNAG_05864	1656.687	0.465818	0.079618	5.850624	4.90E-09	6.00E-08
CNAG_05867	594.6275	0.312555	0.126046	2.479696	0.013149	0.042439
CNAG_05868	170.4907	0.534733	0.169612	3.152695	0.001618	0.007181
CNAG_05870	98.58754	-0.46705	0.207584	-2.24993	0.024453	0.070263
CNAG_05871	971.3132	0.495462	0.092902	5.333148	9.65E-08	9.80E-07
CNAG_05872	1992.376	1.145367	0.096866	11.82428	2.92E-32	2.40E-30
CNAG_05873	261.2707	0.72672	0.136459	5.325556	1.01E-07	1.02E-06
CNAG_12690	15.38342	1.032287	0.499935	2.064844	0.038938	0.101936
CNAG_05874	1540.501	1.279657	0.105751	12.10069	1.05E-33	9.39E-32
CNAG_05877	526.5045	0.293692	0.107484	2.732437	0.006287	0.022841
CNAG_05881	3390.181	0.202243	0.069538	2.908365	0.003633	0.014474

CNAG_05884	6809.555	-0.55105	0.064189	-8.58471	9.11E-18	2.60E-16
CNAG_05889	1101.081	-0.33759	0.098833	-3.41575	0.000636	0.003215
CNAG_12692	66.57408	-1.02494	0.34535	-2.96784	0.002999	0.012241
CNAG_05891	328.9875	0.535924	0.131011	4.090664	4.30E-05	0.000286
CNAG_05893	6660.696	0.148069	0.073678	2.009672	0.044466	0.113668
CNAG_05896	1343.622	-0.18966	0.077328	-2.45266	0.014181	0.045077
CNAG_05899	2360.263	-0.19686	0.08978	-2.19271	0.028329	0.079655
CNAG_05902	576.2938	0.232468	0.100098	2.322402	0.020211	0.060133
CNAG_12695	98.66002	-0.50924	0.237636	-2.14296	0.032116	0.087542
CNAG_05903	1108.543	0.19302	0.084675	2.279534	0.022635	0.065839
CNAG_05905	1057.148	-0.19287	0.080861	-2.38516	0.017072	0.052369
CNAG_05907	15252.22	0.2188	0.059718	3.663872	0.000248	0.001396
CNAG_05909	24037.26	0.440342	0.076707	5.740601	9.43E-09	1.11E-07
CNAG_05913	783.0792	-0.34777	0.094318	-3.68725	0.000227	0.001287
CNAG_05914	335.4824	0.849004	0.138523	6.128978	8.84E-10	1.19E-08
CNAG_05915	357.2785	0.420937	0.154787	2.719466	0.006539	0.023646
CNAG_05918	76000.39	0.169313	0.071308	2.374398	0.017578	0.053668
CNAG_12699	218.5523	0.532344	0.154975	3.435024	0.000593	0.003036
CNAG_05929	461.5085	0.523866	0.120532	4.346267	1.38E-05	0.000102
CNAG_05930	1270.701	0.253571	0.083841	3.024412	0.002491	0.010419
CNAG_05931	2777.129	0.38471	0.066713	5.766688	8.08E-09	9.56E-08
CNAG_05932	6684.503	-0.17859	0.066653	-2.67939	0.007376	0.026223
CNAG_05936	2579.487	-0.39777	0.087215	-4.56083	5.10E-06	4.01E-05
CNAG_05937	669.202	0.222971	0.100328	2.222413	0.026255	0.074644
CNAG_05939	521.1453	0.980643	0.111037	8.831659	1.03E-18	3.23E-17
CNAG_05940	3248.444	0.584621	0.088842	6.580472	4.69E-11	7.32E-10
CNAG_07684	166.6848	0.462873	0.175433	2.638455	0.008328	0.029159
CNAG_07685	362.9124	0.641813	0.118558	5.413487	6.18E-08	6.45E-07
CNAG_07688	1316.993	0.348446	0.083462	4.174884	2.98E-05	0.000204
CNAG_07691	118.7076	0.584254	0.20861	2.800698	0.005099	0.019123
CNAG_12707	16.02995	1.212051	0.487957	2.48393	0.012994	0.042095
CNAG_07692	892.3386	-0.36375	0.086923	-4.18473	2.85E-05	0.000196
CNAG_07694	2040.419	-0.20582	0.071367	-2.88394	0.003927	0.015454
CNAG_07695	8506.504	1.190026	0.065543	18.15633	1.14E-73	7.44E-71
CNAG_07699	1040.26	-0.19775	0.092835	-2.13015	0.033159	0.089666
CNAG_07701	675.5449	0.19257	0.095229	2.022187	0.043157	0.111008
CNAG_07702	171.6778	-0.75741	0.156688	-4.83389	1.34E-06	1.15E-05
CNAG_07703	279.2129	0.291954	0.134929	2.163751	0.030483	0.08421
CNAG_05962	179.8431	0.438807	0.167698	2.616643	0.00888	0.030771
CNAG_05964	100.0946	-0.52641	0.214019	-2.45964	0.013908	0.044355
CNAG_05965	363.2837	-0.37458	0.126039	-2.97198	0.002959	0.012102
CNAG_05968	409.0805	-0.44316	0.110007	-4.02844	5.61E-05	0.000366
CNAG_05970	453.5138	-0.28511	0.117253	-2.43157	0.015034	0.047308
CNAG_05972	92.74429	0.580615	0.224893	2.581738	0.00983	0.033384
CNAG_12711	14.61462	1.006789	0.504388	1.99606	0.045927	0.116479
CNAG_12712	435.7475	0.326127	0.11676	2.79315	0.00522	0.019472
CNAG_05974	179.9595	0.318622	0.158838	2.005959	0.044861	0.114409
CNAG_12714	110.1607	-0.8204	0.239947	-3.41908	0.000628	0.003186

CNAG_05975	3522.451	-0.1716	0.071616	-2.39605	0.016573	0.05103
CNAG_05976	4591.032	-0.27898	0.071959	-3.87687	0.000106	0.000648
CNAG_05977	3659.032	-0.29692	0.113186	-2.62328	0.008709	0.030313
CNAG_10090	4.449642	-2.37464	1.059046	-2.24224	0.024946	0.071519
CNAG_05979	921.1813	0.211762	0.08556	2.475003	0.013324	0.042877
CNAG_05982	120.928	0.614128	0.185068	3.318383	0.000905	0.004356
CNAG_03084	230.4362	1.352661	0.148667	9.0986	9.15E-20	3.26E-18
CNAG_03085	13.94484	1.431724	0.556718	2.57172	0.010119	0.034172
CNAG_12715	45.59801	-0.59103	0.30093	-1.96401	0.049529	0.123649
CNAG_03097	592.9814	0.241098	0.097153	2.481628	0.013078	0.042262
CNAG_03098	2152.345	0.344128	0.07863	4.376548	1.21E-05	9.00E-05
CNAG_03101	315.226	-0.29444	0.124936	-2.35676	0.018435	0.055676
CNAG_03103	1764.363	-0.19986	0.094064	-2.12477	0.033606	0.090529
CNAG_03108	707.4713	0.184901	0.094223	1.962379	0.049718	0.124081
CNAG_03109	3718.852	-0.2071	0.06814	-3.03931	0.002371	0.009949
CNAG_03113	3608.984	0.779742	0.097481	7.998881	1.26E-15	3.04E-14
CNAG_03114	2359.131	0.489224	0.098138	4.985063	6.19E-07	5.58E-06
CNAG_03115	624.1746	-0.41522	0.099754	-4.16245	3.15E-05	0.000215
CNAG_12720	88.83908	-0.56092	0.226597	-2.4754	0.013309	0.042865
CNAG_03118	750.1654	0.217123	0.089536	2.424984	0.015309	0.048058
CNAG_03122	158.8704	-0.32657	0.161802	-2.01832	0.043558	0.111781
CNAG_12721	128.3393	-0.56203	0.195539	-2.87426	0.00405	0.01585
CNAG_03124	3384.072	-0.54447	0.072216	-7.53952	4.72E-14	1.00E-12
CNAG_03127	45797.26	-0.17551	0.074575	-2.35345	0.0186	0.056087
CNAG_03128	771.9169	0.351668	0.093685	3.753742	0.000174	0.00101
CNAG_03129	242.4574	-0.49992	0.156251	-3.19948	0.001377	0.006258
CNAG_03136	2889.86	0.391609	0.068229	5.73964	9.49E-09	1.12E-07
CNAG_12722	49.45307	1.229821	0.282896	4.347257	1.38E-05	0.000102
CNAG_03139	4186.106	-0.34886	0.065057	-5.36236	8.21E-08	8.41E-07
CNAG_03141	54.5919	0.520671	0.264972	1.965006	0.049414	0.1234
CNAG_03142	12025.96	0.918456	0.077509	11.84964	2.16E-32	1.81E-30
CNAG_03143	9624.426	1.511001	0.107075	14.11166	3.22E-45	5.71E-43
CNAG_03144	1785.146	-0.25468	0.079263	-3.21308	0.001313	0.006004
CNAG_03146	11354.1	0.241367	0.073078	3.302857	0.000957	0.004574
CNAG_03147	1373.667	0.216369	0.084102	2.572698	0.010091	0.03412
CNAG_03150	1025.861	-0.26753	0.086682	-3.0864	0.002026	0.008703
CNAG_03152	1669.026	0.17581	0.076219	2.306662	0.021074	0.062248
CNAG_03153	7444.347	-0.29331	0.06442	-4.55304	5.29E-06	4.16E-05
CNAG_12727	172.5004	0.453246	0.162594	2.787592	0.00531	0.019762
CNAG_03155	1089.487	0.346313	0.085717	4.040203	5.34E-05	0.00035
CNAG_03156	1122.978	0.355188	0.083311	4.263377	2.01E-05	0.000142
CNAG_03158	1196.803	0.171803	0.077397	2.219778	0.026434	0.075096
CNAG_03159	1098.277	-0.44051	0.090723	-4.85558	1.20E-06	1.03E-05
CNAG_03162	781.3536	0.57918	0.106109	5.458356	4.81E-08	5.10E-07
CNAG_03167	1321.037	-0.18893	0.091845	-2.05703	0.039684	0.10368
CNAG_03168	9006.1	-0.36874	0.057909	-6.36754	1.92E-10	2.81E-09
CNAG_03170	765.1282	-0.31628	0.091783	-3.44592	0.000569	0.002928
CNAG_03171	1970.789	-0.2345	0.079974	-2.93225	0.003365	0.013551

CNAG_03173	1207.547	0.220952	0.084991	2.599719	0.00933	0.031991
CNAG_03175	675.3006	-0.30926	0.092031	-3.36038	0.000778	0.003818
CNAG_03176	1053.013	-0.92365	0.096258	-9.59555	8.35E-22	3.50E-20
CNAG_03178	1088.43	0.652354	0.096609	6.752548	1.45E-11	2.43E-10
CNAG_03183	2106.818	-0.19397	0.076568	-2.53325	0.011301	0.037465
CNAG_03184	1767.319	-0.30873	0.077036	-4.00758	6.13E-05	0.000398
CNAG_03185	787.8214	0.321259	0.099414	3.231512	0.001231	0.005676
CNAG_03187	446.5194	0.420075	0.11174	3.759412	0.00017	0.00099
CNAG_03188	1900.364	-0.4601	0.102606	-4.48417	7.32E-06	5.65E-05
CNAG_03191	1719.935	0.165774	0.07824	2.118787	0.034108	0.091674
CNAG_03192	391.0663	-0.38219	0.115657	-3.30448	0.000952	0.00455
CNAG_03198	53917.57	-0.2515	0.08138	-3.0904	0.001999	0.008604
CNAG_07717	2857.473	-0.23739	0.072684	-3.266	0.001091	0.005116
CNAG_03205	1669.066	-0.67825	0.07643	-8.87419	7.04E-19	2.27E-17
CNAG_07718	4006.788	0.194109	0.067742	2.865418	0.004165	0.01621
CNAG_03211	87.31884	-0.55408	0.219799	-2.52083	0.011708	0.038634
CNAG_03213	3625.265	0.652554	0.084045	7.76434	8.21E-15	1.86E-13
CNAG_03215	281.1569	-0.51876	0.142208	-3.64787	0.000264	0.001481
CNAG_07719	4926.314	-0.24171	0.069101	-3.49789	0.000469	0.002468
CNAG_07720	2116.664	0.343329	0.091419	3.755568	0.000173	0.001004
CNAG_03220	1791.554	0.341963	0.071537	4.780254	1.75E-06	1.48E-05
CNAG_03222	365.9484	-0.31098	0.146299	-2.12564	0.033534	0.090445
CNAG_03225	20022.78	0.305649	0.078418	3.89768	9.71E-05	0.000599
CNAG_03226	7919.541	1.076126	0.07382	14.57761	3.90E-48	7.25E-46
CNAG_12740	111.2189	0.615044	0.18989	3.238946	0.0012	0.005553
CNAG_03227	540.783	0.57364	0.120685	4.753203	2.00E-06	1.67E-05
CNAG_03228	1421.499	0.52796	0.088279	5.980579	2.22E-09	2.84E-08
CNAG_03231	374.3965	-0.62882	0.122978	-5.11331	3.17E-07	3.00E-06
CNAG_03232	1180.121	0.314651	0.081146	3.877593	0.000105	0.000647
CNAG_03235	2473.885	-0.17182	0.070741	-2.42883	0.015148	0.047647
CNAG_03246	1309.378	-0.53473	0.086046	-6.21442	5.15E-10	7.09E-09
CNAG_03249	5303.822	-0.22797	0.0642	-3.5509	0.000384	0.002053
CNAG_03250	1045.487	0.494509	0.083564	5.9177	3.26E-09	4.07E-08
CNAG_03258	1356.296	-0.32302	0.109613	-2.94689	0.00321	0.012986
CNAG_03262	1940.9	-0.305	0.076883	-3.96708	7.28E-05	0.000466
CNAG_03263	17074.96	0.77233	0.063372	12.18718	3.64E-34	3.34E-32
CNAG_03264	1474.553	-0.2926	0.074526	-3.92621	8.63E-05	0.00054
CNAG_03265	869.9082	-0.29941	0.087308	-3.4293	0.000605	0.003087
CNAG_03268	3360.956	0.199451	0.082425	2.419776	0.01553	0.048537
CNAG_03271	3258.937	-0.29334	0.085746	-3.42101	0.000624	0.003166
CNAG_03272	147.3311	0.573266	0.175609	3.264451	0.001097	0.005141
CNAG_03275	719.4191	-0.34369	0.096382	-3.56587	0.000363	0.001953
CNAG_03281	23715.43	-0.17301	0.082057	-2.10841	0.034995	0.093657
CNAG_03282	1526.421	0.18223	0.080147	2.273702	0.022984	0.066704
CNAG_03283	38384.47	-0.19422	0.081714	-2.37683	0.017462	0.053358
CNAG_03284	1166.398	-0.51197	0.079178	-6.46603	1.01E-10	1.52E-09
CNAG_03285	1983.742	-0.18874	0.070156	-2.69027	0.007139	0.025476
CNAG_03286	1122.141	-0.27195	0.088424	-3.07547	0.002102	0.008967

CNAG_03289	4736.878	-0.23775	0.06319	-3.76252	0.000168	0.000979
CNAG_03291	1452.769	0.168395	0.08147	2.066952	0.038739	0.101585
CNAG_03293	1469.12	-0.20316	0.088356	-2.29936	0.021485	0.063175
CNAG_03297	2283.684	-0.25635	0.076337	-3.35816	0.000785	0.003846
CNAG_03299	6114.225	-0.1682	0.064286	-2.61635	0.008888	0.030772
CNAG_03300	2172.228	-0.45539	0.074772	-6.09039	1.13E-09	1.49E-08
CNAG_03301	2776.169	0.263411	0.07055	3.733702	0.000189	0.001084
CNAG_03302	2227.025	-0.69484	0.07303	-9.5144	1.83E-21	7.55E-20
CNAG_03303	16885.29	-0.19073	0.071067	-2.68376	0.00728	0.025941
CNAG_03310	811.6137	-0.22741	0.091616	-2.48223	0.013056	0.042209
CNAG_03311	7408.077	-0.61784	0.06685	-9.24215	2.42E-20	9.02E-19
CNAG_03319	3157.007	-0.23132	0.072875	-3.17414	0.001503	0.006732
CNAG_03320	1525.257	-0.20518	0.089126	-2.30217	0.021326	0.062849
CNAG_03321	2484.842	0.365019	0.080475	4.535825	5.74E-06	4.49E-05
CNAG_03333	2240.014	0.978728	0.099448	9.841645	7.45E-23	3.48E-21
CNAG_03335	3483.162	-0.29308	0.067116	-4.36674	1.26E-05	9.38E-05
CNAG_03336	1948.461	-0.5576	0.078073	-7.14211	9.19E-13	1.74E-11
CNAG_03337	1260.194	0.254126	0.080494	3.157065	0.001594	0.007083
CNAG_03338	752.783	-0.19044	0.096243	-1.9788	0.047839	0.120391
CNAG_03342	3045.033	-0.32492	0.068947	-4.71255	2.45E-06	2.03E-05
CNAG_03345	3797.605	0.218648	0.106981	2.043802	0.040973	0.106526
CNAG_03346	727.4381	0.457022	0.10199	4.48106	7.43E-06	5.73E-05
CNAG_12757	25.25078	0.895506	0.412346	2.171736	0.029876	0.082853
CNAG_03347	2133.654	0.868191	0.10107	8.590008	8.70E-18	2.50E-16
CNAG_03349	3916.181	-0.17342	0.070944	-2.44448	0.014506	0.045925
CNAG_07724	1782.631	-0.3056	0.10199	-2.99638	0.002732	0.011323
CNAG_03352	903.6938	0.199547	0.094304	2.115995	0.034345	0.09217
CNAG_03354	655.8682	-0.42946	0.096531	-4.44893	8.63E-06	6.58E-05
CNAG_03358	21706.9	0.175629	0.070582	2.488277	0.012836	0.04167
CNAG_03359	2609.13	0.525799	0.089242	5.891813	3.82E-09	4.74E-08
CNAG_03365	2072.541	0.247885	0.073834	3.357343	0.000787	0.003855
CNAG_12760	17.4431	1.099065	0.501315	2.192363	0.028353	0.079655
CNAG_12761	120.7631	-0.5945	0.187516	-3.17038	0.001522	0.006816
CNAG_03372	1764.344	0.363244	0.078911	4.603212	4.16E-06	3.34E-05
CNAG_03375	9205.893	0.684575	0.077751	8.804675	1.31E-18	4.10E-17
CNAG_03376	1424.108	0.207167	0.078553	2.637284	0.008357	0.029221
CNAG_03378	2145.273	-0.14873	0.072465	-2.05246	0.040125	0.104588
CNAG_03381	452.0506	0.507562	0.110072	4.611184	4.00E-06	3.22E-05
CNAG_03383	1142.764	-0.22374	0.086311	-2.59224	0.009535	0.032552
CNAG_03385	1436.328	0.226726	0.082733	2.740462	0.006135	0.022411
CNAG_12764	41.81585	-0.86595	0.309855	-2.7947	0.005195	0.0194
CNAG_03389	5022.74	-0.19916	0.070593	-2.8212	0.004784	0.018148
CNAG_03390	1084.149	-0.49591	0.08147	-6.08707	1.15E-09	1.52E-08
CNAG_03392	428.595	0.324731	0.118761	2.734324	0.006251	0.022731
CNAG_03394	387.543	0.446259	0.113738	3.923578	8.72E-05	0.000545
CNAG_03395	1768.274	0.329063	0.072279	4.552695	5.30E-06	4.16E-05
CNAG_03396	2166.482	0.14432	0.071921	2.006637	0.044788	0.114299
CNAG_03399	4610.012	-0.31705	0.070768	-4.48019	7.46E-06	5.75E-05

CNAG_03402	1335.834	0.177622	0.075544	2.351237	0.018711	0.056292
CNAG_03403	760.1537	0.400253	0.093542	4.278846	1.88E-05	0.000135
CNAG_12766	107.374	1.24672	0.213121	5.849811	4.92E-09	6.02E-08
CNAG_03408	4329.769	0.885112	0.108674	8.144621	3.80E-16	9.52E-15
CNAG_03409	1193.2	-0.25688	0.126315	-2.03367	0.041985	0.108637
CNAG_03411	656.4367	-0.19481	0.096474	-2.01928	0.043458	0.111635
CNAG_03414	431.6995	0.352495	0.111402	3.164187	0.001555	0.006939
CNAG_03415	313.8606	0.268704	0.125397	2.142827	0.032127	0.087542
CNAG_03423	1459.786	-0.19103	0.091489	-2.08806	0.036793	0.097266
CNAG_03438	18468.68	0.29153	0.058266	5.003461	5.63E-07	5.14E-06
CNAG_03445	391.0651	0.345614	0.117888	2.931712	0.003371	0.01356
CNAG_03446	774.449	0.220154	0.094672	2.325437	0.020049	0.059717
CNAG_03447	1242.315	-0.20404	0.085952	-2.37384	0.017604	0.053707
CNAG_03448	1868.853	-0.19526	0.076947	-2.53753	0.011164	0.037073
CNAG_03450	1606.537	0.191679	0.096064	1.995335	0.046006	0.116557
CNAG_03452	1516.824	0.327662	0.082696	3.962231	7.43E-05	0.000474
CNAG_03453	1313.888	-0.32866	0.108618	-3.02588	0.002479	0.010385
CNAG_03454	2173.141	-0.98384	0.089349	-11.0112	3.37E-28	2.29E-26
CNAG_12773	118.5074	0.723301	0.230084	3.143646	0.001669	0.007386
CNAG_03457	8811.615	-0.23057	0.06267	-3.67911	0.000234	0.001324
CNAG_03465	687.4842	-0.60906	0.096249	-6.32795	2.48E-10	3.59E-09
CNAG_03466	1874.843	-0.25234	0.097088	-2.59915	0.009346	0.03203
CNAG_03467	337.6966	-0.31676	0.11976	-2.64494	0.00817	0.028799
CNAG_07735	2151.244	-0.61106	0.079221	-7.71333	1.23E-14	2.74E-13
CNAG_07736	988.2488	-0.24953	0.083933	-2.97301	0.002949	0.012068
CNAG_12779	170.5291	-0.44846	0.159561	-2.8106	0.004945	0.018652
CNAG_12780	887.8317	-0.45792	0.109045	-4.19937	2.68E-05	0.000185
CNAG_03473	475.3949	0.484501	0.102842	4.71112	2.46E-06	2.04E-05
CNAG_03474	87.98224	-0.89707	0.239599	-3.74406	0.000181	0.001044
CNAG_03475	1702.471	0.275007	0.079673	3.451699	0.000557	0.00287
CNAG_03476	10400.98	-0.1378	0.068488	-2.01202	0.044218	0.113288
CNAG_12781	216.1675	0.504716	0.178032	2.834975	0.004583	0.017532
CNAG_03478	279.7529	-0.35967	0.127479	-2.82144	0.004781	0.018148
CNAG_03479	2361.818	-0.17537	0.084895	-2.06566	0.03886	0.101767
CNAG_03481	816.8103	-0.3365	0.085603	-3.93093	8.46E-05	0.000533
CNAG_03482	14375.19	0.26664	0.0697	3.825541	0.00013	0.000781
CNAG_03483	1578.742	-0.235	0.085435	-2.75062	0.005948	0.021764
CNAG_03484	1610.125	-0.16577	0.082939	-1.99864	0.045647	0.115923
CNAG_03485	1299.74	-0.29095	0.092883	-3.13243	0.001734	0.007631
CNAG_03487	1061.947	0.272293	0.088276	3.084563	0.002039	0.008741
CNAG_03489	897.4476	-0.228	0.088872	-2.56551	0.010302	0.03464
CNAG_03492	2855.724	1.085883	0.107477	10.10337	5.34E-24	2.78E-22
CNAG_03493	474.7629	0.243401	0.105772	2.301181	0.021381	0.062919
CNAG_03494	1493.794	-0.15659	0.079516	-1.96923	0.048926	0.122416
CNAG_12786	500.2453	0.577607	0.135369	4.2669	1.98E-05	0.00014
CNAG_03498	477.0979	-0.36773	0.116382	-3.15965	0.00158	0.007028
CNAG_03502	3100.245	0.400835	0.075355	5.319285	1.04E-07	1.05E-06
CNAG_03503	846.7162	-0.51116	0.121669	-4.20123	2.65E-05	0.000183

CNAG_03507	13324.65	0.233606	0.09204	2.538092	0.011146	0.037049
CNAG_03509	6462.621	0.652835	0.07332	8.903865	5.39E-19	1.75E-17
CNAG_03510	26805.38	-0.13397	0.063232	-2.11869	0.034116	0.091674
CNAG_03511	251.8151	0.640272	0.143964	4.447458	8.69E-06	6.62E-05
CNAG_03513	1343.611	-0.41865	0.089494	-4.67792	2.90E-06	2.38E-05
CNAG_07740	4835.611	-0.13341	0.066257	-2.01347	0.044065	0.112935
CNAG_07741	1974.809	0.195119	0.07331	2.661571	0.007778	0.027515
CNAG_03517	4996.552	0.640271	0.068221	9.385203	6.28E-21	2.50E-19
CNAG_03518	86.32415	-0.51491	0.218976	-2.35146	0.0187	0.056279
CNAG_03525	1153.455	0.356751	0.096246	3.706667	0.00021	0.001195
CNAG_03527	2047.145	0.239945	0.075362	3.183908	0.001453	0.006558
CNAG_07995	1587.186	0.160675	0.075357	2.132174	0.032993	0.089277
CNAG_03534	308.5861	0.524143	0.124281	4.217398	2.47E-05	0.000172
CNAG_07745	3835.375	1.415196	0.100665	14.05847	6.83E-45	1.19E-42
CNAG_03543	1739.478	0.343697	0.077141	4.455443	8.37E-06	6.41E-05
CNAG_03552	896.6971	0.873576	0.087422	9.992669	1.64E-23	8.16E-22
CNAG_03553	5065.735	0.276191	0.06502	4.247796	2.16E-05	0.000152
CNAG_03554	10174.61	-0.20714	0.067294	-3.07815	0.002083	0.008907
CNAG_03555	487.8303	0.404396	0.122736	3.294856	0.000985	0.004689
CNAG_03557	868.4628	0.242426	0.093382	2.596079	0.009429	0.032289
CNAG_03559	516.2912	0.262108	0.102014	2.569343	0.010189	0.034348
CNAG_03561	847.8344	0.290532	0.097281	2.986531	0.002822	0.011626
CNAG_03563	3377.05	0.76039	0.073818	10.30095	6.98E-25	3.83E-23
CNAG_03564	1578.123	0.886484	0.087701	10.10803	5.09E-24	2.67E-22
CNAG_03565	3266.017	0.838819	0.072167	11.62323	3.14E-31	2.43E-29
CNAG_03566	4987.727	0.974966	0.090958	10.71891	8.30E-27	5.18E-25
CNAG_03567	2479.539	0.169069	0.07279	2.322698	0.020195	0.060109
CNAG_03570	83.68475	0.531441	0.223173	2.381293	0.017252	0.052819
CNAG_03572	2680.889	1.574159	0.081656	19.27783	8.25E-83	7.15E-80
CNAG_03577	51973.66	-0.16562	0.073176	-2.26327	0.023619	0.068168
CNAG_07748	494.8356	-0.31141	0.102731	-3.03134	0.002435	0.010204
CNAG_03583	934.8854	0.199175	0.089052	2.236616	0.025311	0.072462
CNAG_03592	3129.466	0.379825	0.069765	5.44431	5.20E-08	5.48E-07
CNAG_06993	4571.13	0.378651	0.076288	4.963455	6.93E-07	6.20E-06
CNAG_07752	5908.589	0.179042	0.060955	2.937295	0.003311	0.01336
CNAG_06967	2694.469	0.134227	0.065875	2.037607	0.041589	0.107837
CNAG_04102	364.5348	-0.36153	0.11775	-3.07036	0.002138	0.009093
CNAG_04105	3897.213	1.85249	0.107871	17.17318	4.22E-66	2.06E-63
CNAG_07755	172.836	0.973597	0.17445	5.580948	2.39E-08	2.64E-07
CNAG_04106	1888.223	1.025835	0.101095	10.14728	3.41E-24	1.80E-22
CNAG_04107	427.8002	0.564905	0.127966	4.414474	1.01E-05	7.64E-05
CNAG_04108	1300.788	0.239801	0.084554	2.836078	0.004567	0.01748
CNAG_04110	493.2954	0.231675	0.106277	2.179928	0.029263	0.081618
CNAG_12812	81.53193	0.635349	0.227918	2.787621	0.00531	0.019762
CNAG_04112	643.1146	0.658316	0.107745	6.109969	9.97E-10	1.32E-08
CNAG_07756	1758.341	-0.26486	0.080274	-3.29945	0.000969	0.004618
CNAG_04119	1534.979	-0.32815	0.111381	-2.94616	0.003217	0.013003
CNAG_04120	2299.496	0.208022	0.071587	2.905854	0.003663	0.014553

CNAG_04121	2340.709	0.151728	0.072456	2.094075	0.036253	0.096199
CNAG_04124	214.0491	-0.35742	0.144517	-2.47323	0.01339	0.043014
CNAG_12817	44.64556	0.702596	0.296636	2.368549	0.017858	0.054269
CNAG_07757	1129.705	-0.21303	0.09562	-2.22787	0.02589	0.073821
CNAG_04136	1890.39	-0.37871	0.073597	-5.14577	2.66E-07	2.55E-06
CNAG_12820	68.41902	-0.57543	0.238093	-2.41683	0.015656	0.048834
CNAG_04141	3292.224	-0.32613	0.068263	-4.77753	1.77E-06	1.50E-05
CNAG_07758	393.0913	-0.30793	0.11429	-2.69431	0.007053	0.025215
CNAG_04147	1013	-0.65077	0.090574	-7.18493	6.72E-13	1.29E-11
CNAG_04150	1751.566	-0.24493	0.073742	-3.32142	0.000896	0.004312
CNAG_04160	28.86395	0.796288	0.37429	2.127464	0.033382	0.090079
CNAG_04163	3961.601	1.131521	0.074326	15.22378	2.46E-52	5.82E-50
CNAG_04168	7976.459	-0.26973	0.074029	-3.64361	0.000269	0.001501
CNAG_04173	1603.926	0.336772	0.076293	4.414193	1.01E-05	7.64E-05
CNAG_04175	2332.032	-0.7	0.071785	-9.75136	1.82E-22	8.07E-21
CNAG_04177	1024.03	0.221008	0.085491	2.585165	0.009733	0.033112
CNAG_04184	581.5363	-0.22721	0.106256	-2.13833	0.03249	0.088316
CNAG_04185	819.394	0.971775	0.11728	8.285913	1.17E-16	3.09E-15
CNAG_04186	852.9232	0.905827	0.088299	10.25867	1.08E-24	5.90E-23
CNAG_04188	1025.983	0.220977	0.085729	2.577635	0.009948	0.033695
CNAG_04189	17358.69	1.060227	0.063457	16.70772	1.15E-62	4.49E-60
CNAG_04190	5526.788	-0.1827	0.066144	-2.76222	0.005741	0.021083
CNAG_04192	7087.585	-0.31515	0.064282	-4.90266	9.45E-07	8.23E-06
CNAG_04193	1078.696	0.193536	0.084089	2.301575	0.021359	0.062901
CNAG_04195	857.7131	0.248843	0.091412	2.722218	0.006485	0.023515
CNAG_04200	549.3563	0.599701	0.122606	4.891277	1.00E-06	8.69E-06
CNAG_04205	1704.441	-0.27574	0.080517	-3.4246	0.000616	0.003134
CNAG_04206	435.6872	0.54534	0.110558	4.932612	8.11E-07	7.12E-06
CNAG_12830	57.28478	0.679021	0.273511	2.482614	0.013042	0.042209
CNAG_04208	1423.488	0.674541	0.079117	8.525819	1.52E-17	4.21E-16
CNAG_04209	2623.497	-0.67281	0.077127	-8.72343	2.70E-18	8.16E-17
CNAG_04216	6.095447	-1.93457	0.869879	-2.22395	0.026152	0.074457
CNAG_04219	3708.765	0.276874	0.079537	3.481068	0.000499	0.002607
CNAG_04221	1682.419	0.218341	0.076294	2.861855	0.004212	0.016336
CNAG_04222	3696.999	-0.70336	0.070605	-9.96178	2.24E-23	1.10E-21
CNAG_04224	2540.975	-0.1697	0.077535	-2.18868	0.02862	0.080333
CNAG_04230	314.366	-0.53675	0.163818	-3.27651	0.001051	0.004962
CNAG_04236	3578.347	-0.25819	0.072208	-3.57559	0.000349	0.001894
CNAG_04242	568.6742	-0.28217	0.09929	-2.84186	0.004485	0.0172
CNAG_04243	1878.639	-0.34161	0.107531	-3.17687	0.001489	0.006689
CNAG_04246	104.2605	-0.48401	0.19858	-2.43737	0.014794	0.046724
CNAG_04248	727.7557	0.663496	0.093931	7.063655	1.62E-12	3.00E-11
CNAG_04253	666.0195	0.216625	0.10629	2.038057	0.041544	0.107837
CNAG_04256	262.9423	0.317239	0.129816	2.443762	0.014535	0.045998
CNAG_04259	6415.73	-0.3363	0.070635	-4.76102	1.93E-06	1.61E-05
CNAG_04263	504.5128	-0.32849	0.114256	-2.875	0.00404	0.01582
CNAG_12837	10.31796	1.676616	0.644651	2.600813	0.0093	0.031917
CNAG_04265	3254.494	0.348682	0.082385	4.232371	2.31E-05	0.000162

CNAG_04267	1141.577	0.313493	0.08141	3.850766	0.000118	0.000712
CNAG_04268	580.8018	0.242908	0.098162	2.474559	0.01334	0.042913
CNAG_04269	1284.945	0.302748	0.088434	3.423444	0.000618	0.003146
CNAG_04270	562.5114	0.373341	0.10675	3.49734	0.00047	0.002471
CNAG_04271	624.7144	-0.39137	0.102633	-3.81324	0.000137	0.000816
CNAG_04273	2366.068	0.248423	0.078571	3.161778	0.001568	0.006985
CNAG_04275	1191.529	0.835533	0.099783	8.373478	5.59E-17	1.50E-15
CNAG_04276	2472.97	0.280179	0.072384	3.870721	0.000109	0.000663
CNAG_04280	58.64069	-0.64866	0.265073	-2.44709	0.014402	0.04565
CNAG_04281	2629.925	0.182316	0.070596	2.582525	0.009808	0.033323
CNAG_12840	251.9319	0.313763	0.152939	2.051559	0.040212	0.104781
CNAG_04287	3008.396	0.38254	0.077154	4.958159	7.12E-07	6.33E-06
CNAG_04288	813.0117	0.217723	0.100261	2.171556	0.029889	0.082861
CNAG_04291	1361.06	0.215709	0.083245	2.591241	0.009563	0.032632
CNAG_04293	1225.32	0.236416	0.095414	2.477774	0.01322	0.04265
CNAG_04297	618.331	0.219255	0.100975	2.171372	0.029903	0.082871
CNAG_04298	3441.175	-0.83045	0.092174	-9.00961	2.07E-19	7.02E-18
CNAG_04299	630.4653	-0.21489	0.094787	-2.26706	0.023387	0.067635
CNAG_04308	884.4189	-0.69944	0.095322	-7.33766	2.17E-13	4.46E-12
CNAG_04309	321.4219	0.502751	0.134077	3.749708	0.000177	0.001024
CNAG_04311	579.776	-0.30305	0.099077	-3.05868	0.002223	0.009414
CNAG_04313	273.1472	-0.31718	0.136336	-2.32644	0.019995	0.059603
CNAG_04314	3470.192	0.731261	0.082607	8.852294	8.57E-19	2.73E-17
CNAG_04315	2592.851	0.540211	0.079714	6.77689	1.23E-11	2.07E-10
CNAG_04316	2129.187	0.212655	0.088405	2.405456	0.016152	0.050041
CNAG_04322	1702.391	1.517201	0.096963	15.64727	3.47E-55	1.04E-52
CNAG_04326	1095.346	0.174193	0.079708	2.185391	0.02886	0.080784
CNAG_04328	1131.136	-0.18836	0.088137	-2.13709	0.032591	0.088404
CNAG_04329	757.0146	0.233245	0.100415	2.32282	0.020189	0.060109
CNAG_04334	2306.024	-0.29113	0.083027	-3.50649	0.000454	0.002399
CNAG_04338	583.6387	0.327018	0.107079	3.053999	0.002258	0.009541
CNAG_04339	1660.374	0.219067	0.076688	2.856611	0.004282	0.016543
CNAG_04340	2114.811	0.157155	0.072779	2.15934	0.030824	0.08482
CNAG_04341	1669.834	-0.27996	0.109648	-2.55331	0.01067	0.035739
CNAG_04343	151.8524	-0.54419	0.173447	-3.13748	0.001704	0.007517
CNAG_04344	268.6597	-0.44038	0.145779	-3.02085	0.002521	0.01052
CNAG_04346	249.3646	-0.3416	0.140168	-2.43708	0.014806	0.046743
CNAG_04347	4789.554	-0.39289	0.064984	-6.046	1.48E-09	1.93E-08
CNAG_04348	2013.069	-0.6791	0.072492	-9.36796	7.40E-21	2.90E-19
CNAG_12847	8.249275	1.851945	0.706672	2.620659	0.008776	0.030507
CNAG_04351	418.6929	0.494468	0.120082	4.117772	3.83E-05	0.000256
CNAG_04353	800.5143	0.456403	0.089011	5.127506	2.94E-07	2.79E-06
CNAG_04354	1011.752	0.555614	0.086298	6.438334	1.21E-10	1.80E-09
CNAG_04355	221.1769	-0.54168	0.161443	-3.35527	0.000793	0.003875
CNAG_04356	1820.819	-0.41887	0.0717	-5.84206	5.16E-09	6.29E-08
CNAG_04358	2922.877	-0.27373	0.082391	-3.3223	0.000893	0.004301
CNAG_04359	1406.781	0.204756	0.103517	1.977996	0.047929	0.120541
CNAG_04360	1557.203	-0.30923	0.077396	-3.99541	6.46E-05	0.000417

CNAG_04361	5873.562	0.340643	0.074532	4.570423	4.87E-06	3.84E-05
CNAG_04364	3578.922	-0.16579	0.067336	-2.46212	0.013812	0.044121
CNAG_04365	5407.368	-0.56644	0.065016	-8.7123	2.98E-18	8.94E-17
CNAG_04367	507.5728	-0.54298	0.109718	-4.94891	7.46E-07	6.62E-06
CNAG_04370	3497.556	-0.45147	0.096619	-4.67266	2.97E-06	2.43E-05
CNAG_04371	926.1894	0.24368	0.093164	2.615604	0.008907	0.030824
CNAG_04372	1744.171	-0.27068	0.082778	-3.26994	0.001076	0.00506
CNAG_04373	6932.488	0.155662	0.062177	2.503536	0.012296	0.040177
CNAG_04375	2840.964	0.187631	0.071163	2.636654	0.008373	0.029262
CNAG_04376	2877.448	-0.4191	0.073919	-5.66968	1.43E-08	1.64E-07
CNAG_04386	1034.295	1.005374	0.099733	10.08067	6.73E-24	3.48E-22
CNAG_04388	3433.417	0.231325	0.069539	3.326575	0.000879	0.004251
CNAG_04389	600.3918	-0.25939	0.095997	-2.70203	0.006892	0.024762
CNAG_07765	276.9015	1.171023	0.160514	7.295443	2.98E-13	5.97E-12
CNAG_04395	1772.96	-0.4965	0.077756	-6.38538	1.71E-10	2.52E-09
CNAG_04396	362.1463	0.340287	0.118328	2.875795	0.00403	0.015789
CNAG_04398	1172.634	-0.18551	0.081625	-2.27273	0.023042	0.066823
CNAG_04401	2717.892	0.148486	0.075405	1.969179	0.048933	0.122416
CNAG_04404	174.349	0.50183	0.161759	3.102336	0.00192	0.008324
CNAG_04408	1440.646	-0.29104	0.07754	-3.75338	0.000174	0.00101
CNAG_04409	448.8622	0.316738	0.135707	2.333986	0.019596	0.058549
CNAG_04412	632.1938	0.25968	0.101932	2.547579	0.010847	0.036223
CNAG_04414	973.2687	0.268209	0.095072	2.821104	0.004786	0.018148
CNAG_04416	32.78958	-0.84891	0.351381	-2.41594	0.015695	0.048914
CNAG_04417	669.3445	0.65466	0.100497	6.514202	7.31E-11	1.12E-09
CNAG_12852	191.0924	-0.36779	0.160625	-2.28975	0.022036	0.064408
CNAG_07769	2290.137	-0.2386	0.117231	-2.03528	0.041823	0.108277
CNAG_04434	978.3457	-0.20923	0.095798	-2.18412	0.028954	0.080983
CNAG_04440	575.3355	-0.50124	0.10152	-4.93735	7.92E-07	6.98E-06
CNAG_04443	1364.237	0.319956	0.08143	3.929206	8.52E-05	0.000535
CNAG_04445	41820.04	-0.22353	0.071416	-3.12997	0.001748	0.007678
CNAG_04446	260.5916	-0.48355	0.142414	-3.39536	0.000685	0.003433
CNAG_04448	65106.93	-0.24288	0.07073	-3.43388	0.000595	0.003047
CNAG_04451	1197.258	-0.30365	0.077447	-3.92079	8.83E-05	0.000551
CNAG_04452	1211.155	-0.1895	0.090033	-2.10483	0.035306	0.09423
CNAG_04456	632.2115	0.298368	0.103832	2.87357	0.004059	0.015874
CNAG_04459	44.42189	1.429047	0.340127	4.201513	2.65E-05	0.000183
CNAG_12862	95.57403	0.425554	0.207939	2.046528	0.040704	0.105957
CNAG_04466	469.6712	0.255146	0.112797	2.261992	0.023698	0.068293
CNAG_04468	303.3887	0.481285	0.126236	3.812581	0.000138	0.000817
CNAG_12863	16.52672	1.423029	0.494081	2.880153	0.003975	0.015611
CNAG_04470	297.4702	0.750965	0.137789	5.450101	5.03E-08	5.32E-07
CNAG_04471	538.7432	0.683563	0.126716	5.394428	6.87E-08	7.11E-07
CNAG_04472	97.78827	1.165065	0.206193	5.650366	1.60E-08	1.83E-07
CNAG_04473	1073.411	0.336787	0.081341	4.140431	3.47E-05	0.000234
CNAG_04475	2040.914	0.284018	0.070295	4.040382	5.34E-05	0.00035
CNAG_04476	139.2986	0.626923	0.19306	3.247306	0.001165	0.005412
CNAG_07771	2221.947	0.364935	0.069843	5.225107	1.74E-07	1.71E-06

CNAG_04485	6669.458	0.265913	0.062499	4.254657	2.09E-05	0.000147
CNAG_07775	962.4264	0.974307	0.113836	8.558837	1.14E-17	3.20E-16
CNAG_04495	181.3039	-0.4007	0.161005	-2.48877	0.012819	0.04163
CNAG_04497	574.4487	-0.19566	0.097784	-2.00096	0.045397	0.115589
CNAG_04498	1453.286	0.195981	0.083465	2.34807	0.018871	0.056706
CNAG_04499	3639.866	-0.18921	0.074191	-2.55028	0.010764	0.03602
CNAG_04501	3449.385	-0.28112	0.065943	-4.26308	2.02E-05	0.000142
CNAG_04503	695.2615	-0.1975	0.098806	-1.99885	0.045625	0.115903
CNAG_04505	2085.026	-0.43283	0.077252	-5.60274	2.11E-08	2.35E-07
CNAG_04506	86.47989	0.622139	0.216299	2.876295	0.004024	0.015772
CNAG_04511	404.7046	0.458712	0.117065	3.91845	8.91E-05	0.000554
CNAG_04513	2413.342	-0.52222	0.078814	-6.62607	3.45E-11	5.42E-10
CNAG_04514	1516.943	-0.22238	0.078036	-2.84975	0.004375	0.016845
CNAG_04515	2645.963	-0.48035	0.081973	-5.85984	4.63E-09	5.71E-08
CNAG_12869	176.4357	-0.39919	0.158839	-2.51315	0.011966	0.039252
CNAG_04521	70.9804	-0.5043	0.252665	-1.99594	0.045941	0.116479
CNAG_04523	278.1895	4.959781	0.223265	22.2148	2.47E-109	4.82E-106
CNAG_04524	1105.208	0.280368	0.096822	2.895721	0.003783	0.014978
CNAG_04528	279.089	-0.37989	0.170599	-2.22678	0.025962	0.073979
CNAG_04529	391.8999	0.317746	0.115006	2.762863	0.00573	0.021072
CNAG_04531	1514.554	0.175641	0.075392	2.329712	0.019821	0.059153
CNAG_04536	28.41788	-1.62755	0.414679	-3.92486	8.68E-05	0.000543
CNAG_04539	1169.59	0.76801	0.082981	9.255288	2.14E-20	8.02E-19
CNAG_04541	1429.239	-0.18666	0.081016	-2.30393	0.021226	0.062645
CNAG_07782	691.4998	0.415641	0.090898	4.572637	4.82E-06	3.80E-05
CNAG_12874	59.51824	-1.125	0.263311	-4.27249	1.93E-05	0.000137
CNAG_06997	358.5513	-0.78325	0.167738	-4.66946	3.02E-06	2.46E-05
CNAG_06999	580.1715	0.871532	0.101445	8.591206	8.61E-18	2.48E-16
CNAG_12875	38.64572	0.947718	0.325922	2.907805	0.00364	0.014485
CNAG_07784	32.0604	-0.72027	0.353757	-2.03605	0.041745	0.108124
CNAG_04927	98.49088	0.558263	0.224903	2.482237	0.013056	0.042209
CNAG_04926	182.7015	0.873954	0.159861	5.466954	4.58E-08	4.87E-07
CNAG_04920	1336.674	1.000739	0.076792	13.03175	8.07E-39	1.05E-36
CNAG_04919	285.9382	-0.2681	0.124702	-2.14993	0.031561	0.086331
CNAG_07839	49184.56	-0.17418	0.070398	-2.47423	0.013352	0.042935
CNAG_04913	1583.295	-0.25353	0.075729	-3.34789	0.000814	0.003969
CNAG_04911	479.5338	0.487783	0.113049	4.314773	1.60E-05	0.000116
CNAG_04908	921.806	0.632659	0.085871	7.367573	1.74E-13	3.59E-12
CNAG_04906	2477.7	-0.22431	0.070038	-3.20267	0.001362	0.006207
CNAG_07842	151.5463	0.880531	0.173368	5.078977	3.79E-07	3.54E-06
CNAG_04903	1044.609	1.08429	0.103812	10.44477	1.55E-25	8.82E-24
CNAG_04902	1624.476	0.214577	0.074149	2.893843	0.003806	0.01503
CNAG_04899	1348.06	-0.59525	0.07782	-7.64915	2.02E-14	4.43E-13
CNAG_04896	1670.207	-0.15274	0.07332	-2.08313	0.037239	0.098313
CNAG_04894	309.5473	0.589139	0.143894	4.094251	4.24E-05	0.000282
CNAG_04889	666.3531	0.53923	0.112914	4.775564	1.79E-06	1.51E-05
CNAG_04886	537.5576	0.664967	0.108711	6.116854	9.54E-10	1.27E-08
CNAG_12883	38.89672	0.69366	0.324448	2.13797	0.032519	0.088322

CNAG_04883	37370.24	-0.15532	0.072407	-2.14508	0.031946	0.08718
CNAG_04879	5310.114	0.436238	0.08084	5.396341	6.80E-08	7.05E-07
CNAG_04874	271.818	-1.02941	0.145496	-7.07519	1.49E-12	2.77E-11
CNAG_04873	164.4219	-0.54624	0.161663	-3.3789	0.000728	0.003613
CNAG_04872	1912.884	-0.27875	0.077507	-3.59644	0.000323	0.001757
CNAG_04869	1611.108	-0.56418	0.0805	-7.00844	2.41E-12	4.33E-11
CNAG_04868	1062.769	-0.29169	0.086047	-3.38993	0.000699	0.003493
CNAG_04865	80.03711	0.724653	0.267098	2.713059	0.006667	0.02409
CNAG_04864	3335.516	0.401224	0.078487	5.111975	3.19E-07	3.02E-06
CNAG_04862	2841.019	0.819458	0.085833	9.547106	1.33E-21	5.57E-20
CNAG_12890	30.76266	1.343195	0.36449	3.685135	0.000229	0.001295
CNAG_04861	1803.628	0.544993	0.079778	6.831403	8.41E-12	1.45E-10
CNAG_04859	970.5557	-0.20964	0.088337	-2.37321	0.017634	0.053736
CNAG_04855	929.2033	-0.2216	0.09063	-2.44515	0.014479	0.045858
CNAG_04853	604.5131	-0.21941	0.097626	-2.24749	0.024609	0.070658
CNAG_04851	10397.74	-0.36871	0.058448	-6.30835	2.82E-10	4.05E-09
CNAG_04849	494.1704	0.255282	0.122607	2.082122	0.037331	0.098424
CNAG_04843	1103.258	-0.32972	0.079263	-4.15983	3.18E-05	0.000217
CNAG_04837	595.3749	2.727641	0.254583	10.71414	8.74E-27	5.41E-25
CNAG_04836	496.7626	-0.42363	0.128902	-3.28641	0.001015	0.004814
CNAG_04833	1429.36	0.746369	0.105224	7.093151	1.31E-12	2.46E-11
CNAG_04832	1170.947	-0.27395	0.084025	-3.2604	0.001113	0.005205
CNAG_04830	927.7016	0.21999	0.093882	2.343254	0.019116	0.057373
CNAG_12895	132.1852	-0.92472	0.195424	-4.73186	2.22E-06	1.85E-05
CNAG_04828	8260.451	-0.2046	0.059775	-3.42284	0.00062	0.003151
CNAG_04827	280.146	0.789368	0.135159	5.840301	5.21E-09	6.34E-08
CNAG_04822	1231.983	-0.32548	0.086249	-3.77377	0.000161	0.000939
CNAG_04820	1706.01	-0.19917	0.081109	-2.45559	0.014065	0.044766
CNAG_04819	511.2956	-0.39187	0.103419	-3.78917	0.000151	0.00089
CNAG_04817	1007.72	-0.28924	0.08909	-3.24655	0.001168	0.005421
CNAG_04816	504.2049	0.547128	0.107469	5.091015	3.56E-07	3.33E-06
CNAG_07845	150.8946	0.384969	0.188938	2.037538	0.041596	0.107837
CNAG_12898	351.4718	0.382976	0.120838	3.169348	0.001528	0.006837
CNAG_12900	86.92575	0.502919	0.220118	2.284767	0.022326	0.065111
CNAG_04807	2294.996	0.526383	0.093788	5.612507	1.99E-08	2.22E-07
CNAG_04805	1446.821	-0.15472	0.075975	-2.03647	0.041703	0.108052
CNAG_12901	63.40217	-0.7154	0.258498	-2.76752	0.005649	0.020813
CNAG_04804	1715.13	-0.74996	0.076968	-9.74381	1.96E-22	8.64E-21
CNAG_04802	1575.69	-0.51882	0.073825	-7.02765	2.10E-12	3.82E-11
CNAG_04799	44372.09	-0.21261	0.074578	-2.85084	0.00436	0.016796
CNAG_04794	1673.157	0.717147	0.097619	7.346392	2.04E-13	4.19E-12
CNAG_04791	1405.802	-0.35768	0.081789	-4.37325	1.22E-05	9.12E-05
CNAG_04790	1010.288	-0.21062	0.089962	-2.34117	0.019223	0.057633
CNAG_04789	1604.377	0.464479	0.092384	5.027677	4.96E-07	4.57E-06
CNAG_04785	591.4588	0.221004	0.095021	2.325851	0.020027	0.059674
CNAG_04783	1661.859	-0.37653	0.079916	-4.71159	2.46E-06	2.04E-05
CNAG_07851	12957.27	1.262657	0.068475	18.43978	6.30E-76	4.92E-73
CNAG_04777	769.4957	-0.19129	0.093529	-2.04528	0.040827	0.106241

CNAG_04774	1382.284	-0.31871	0.092125	-3.45954	0.000541	0.0028
CNAG_04772	2386.619	-0.24348	0.077305	-3.14961	0.001635	0.007245
CNAG_04770	2271.623	-0.50426	0.074474	-6.77096	1.28E-11	2.15E-10
CNAG_04769	1054.581	-0.22895	0.087864	-2.60567	0.009169	0.031579
CNAG_04768	948.2273	-0.34692	0.091938	-3.77344	0.000161	0.00094
CNAG_04766	36.47392	0.701087	0.344103	2.037435	0.041606	0.107837
CNAG_04762	47143.77	-0.24238	0.083945	-2.8873	0.003886	0.015315
CNAG_04760	2535.004	0.381612	0.105058	3.63239	0.000281	0.001563
CNAG_12910	228.4891	1.996554	0.161624	12.35308	4.69E-35	4.57E-33
CNAG_04758	1745.311	1.833638	0.209159	8.766714	1.84E-18	5.70E-17
CNAG_04757	1524.157	0.827992	0.079811	10.37443	3.24E-25	1.81E-23
CNAG_04756	795.7556	0.474387	0.106145	4.469255	7.85E-06	6.03E-05
CNAG_12911	26.20503	1.138112	0.405934	2.803685	0.005052	0.018974
CNAG_04751	735.9765	0.349026	0.089367	3.905549	9.40E-05	0.000581
CNAG_04748	881.1241	0.427502	0.087077	4.909481	9.13E-07	7.97E-06
CNAG_04747	642.97	0.626376	0.09503	6.591319	4.36E-11	6.82E-10
CNAG_04746	1775.861	0.672114	0.079687	8.434417	3.33E-17	9.08E-16
CNAG_04744	1309.805	0.948435	0.096236	9.855338	6.50E-23	3.08E-21
CNAG_04742	743.8265	0.276863	0.095131	2.910332	0.00361	0.014412
CNAG_04739	735.702	0.197276	0.099927	1.974191	0.04836	0.121312
CNAG_04737	6578.799	0.981463	0.076145	12.88942	5.16E-38	6.40E-36
CNAG_12917	66.87135	0.580911	0.248553	2.337171	0.01943	0.058186
CNAG_04736	487.2422	0.508366	0.118951	4.273733	1.92E-05	0.000137
CNAG_04735	3048.101	1.066921	0.07197	14.82455	1.02E-49	2.09E-47
CNAG_04734	1176.237	0.281046	0.079204	3.548362	0.000388	0.002071
CNAG_04733	1515.459	-0.20058	0.091317	-2.19653	0.028054	0.079037
CNAG_04730	1561.226	0.414708	0.086908	4.771785	1.83E-06	1.53E-05
CNAG_04729	2480.682	-0.41417	0.068346	-6.05989	1.36E-09	1.78E-08
CNAG_04727	442.8037	0.62595	0.125616	4.983056	6.26E-07	5.63E-06
CNAG_04726	35539.77	-0.20268	0.070275	-2.88411	0.003925	0.015454
CNAG_04713	1201.178	-0.1585	0.080657	-1.96514	0.049398	0.1234
CNAG_04712	1034.523	-0.32916	0.082528	-3.98844	6.65E-05	0.000429
CNAG_04711	72.9111	-0.79709	0.236033	-3.37702	0.000733	0.003631
CNAG_04705	1641.232	0.229998	0.073483	3.129961	0.001748	0.007678
CNAG_04704	32.36871	0.809912	0.342156	2.367088	0.017929	0.054463
CNAG_12926	65.07278	0.654229	0.250039	2.616509	0.008883	0.030771
CNAG_04696	643.0998	0.725799	0.10287	7.055526	1.72E-12	3.17E-11
CNAG_04694	4420.793	-0.56608	0.06493	-8.71831	2.82E-18	8.51E-17
CNAG_04690	982.1498	1.101745	0.088831	12.40277	2.52E-35	2.53E-33
CNAG_04689	486.1518	0.438031	0.105015	4.171121	3.03E-05	0.000207
CNAG_04688	405.1434	0.24547	0.109898	2.233621	0.025508	0.072971
CNAG_04687	15516.44	0.745205	0.076561	9.733527	2.17E-22	9.51E-21
CNAG_07856	1792.413	1.029614	0.081686	12.60458	1.99E-36	2.16E-34
CNAG_04684	3516.273	-0.18071	0.065657	-2.75236	0.005917	0.021678
CNAG_04680	1089.643	1.01637	0.093804	10.83506	2.35E-27	1.54E-25
CNAG_04677	698.5664	-0.91735	0.105686	-8.67995	3.96E-18	1.17E-16
CNAG_04676	7561.579	0.162997	0.069082	2.359477	0.018301	0.055335
CNAG_07999	474.2018	0.287963	0.10896	2.642825	0.008222	0.028954

CNAG_04667	689.1706	0.285661	0.09574	2.983709	0.002848	0.011715
CNAG_04666	4502.247	-0.13835	0.064428	-2.14729	0.03177	0.086812
CNAG_04664	921.9707	0.214543	0.088214	2.43207	0.015013	0.047261
CNAG_04659	15387.64	1.131069	0.082954	13.63481	2.49E-42	3.66E-40
CNAG_12933	114.2058	0.989407	0.198034	4.996156	5.85E-07	5.31E-06
CNAG_04658	574.7633	0.514468	0.095576	5.382809	7.33E-08	7.54E-07
CNAG_04657	362.3469	-0.28646	0.128829	-2.22353	0.02618	0.074511
CNAG_04654	1063.095	-0.18915	0.083782	-2.25759	0.023971	0.069029
CNAG_04640	33379.6	-0.33552	0.058824	-5.70384	1.17E-08	1.36E-07
CNAG_04639	2878.66	-0.22414	0.067752	-3.30825	0.000939	0.004497
CNAG_04636	1743.766	0.249872	0.071326	3.503262	0.00046	0.002427
CNAG_04635	2091.765	0.25345	0.070672	3.586269	0.000335	0.001822
CNAG_04634	834.0277	0.340766	0.089384	3.812371	0.000138	0.000817
CNAG_04632	320.965	1.935172	0.33353	5.802086	6.55E-09	7.84E-08
CNAG_12938	98.00533	1.106445	0.205494	5.384324	7.27E-08	7.49E-07
CNAG_04631	1110.055	-0.44537	0.0887	-5.02115	5.14E-07	4.72E-06
CNAG_04630	1657.543	0.422312	0.096403	4.380697	1.18E-05	8.84E-05
CNAG_04628	3115.489	-0.24509	0.077208	-3.17435	0.001502	0.006732
CNAG_04626	237.5972	0.317017	0.148675	2.132278	0.032984	0.089277
CNAG_04625	2885.583	0.669971	0.077459	8.649319	5.18E-18	1.50E-16
CNAG_04621	5769.563	-0.19014	0.076214	-2.49484	0.012601	0.041044
CNAG_04620	1156.64	0.214379	0.085287	2.513621	0.01195	0.039216
CNAG_04617	444.1712	0.557625	0.116803	4.774045	1.81E-06	1.52E-05
CNAG_04616	261.2833	-0.3357	0.146764	-2.28736	0.022175	0.064789
CNAG_04615	357.2966	-0.59028	0.118522	-4.9804	6.35E-07	5.70E-06
CNAG_04614	315.3463	0.306571	0.127567	2.403216	0.016252	0.050249
CNAG_04613	3032.253	-0.24741	0.068256	-3.62479	0.000289	0.001599
CNAG_04611	1076.935	0.600647	0.093198	6.444854	1.16E-10	1.73E-09
CNAG_07858	8.716145	1.892256	0.708315	2.67149	0.007552	0.026812
CNAG_04605	5599.391	-0.21772	0.066593	-3.26936	0.001078	0.005068
CNAG_04602	253.8439	0.302634	0.140957	2.146996	0.031794	0.086815
CNAG_04599	1079.649	-0.19135	0.094434	-2.02624	0.04274	0.110153
CNAG_04589	468.6882	0.516438	0.129553	3.986306	6.71E-05	0.000432
CNAG_04588	1739.816	-0.30792	0.091933	-3.34942	0.00081	0.00395
CNAG_04587	3695.574	1.17103	0.0745	15.71863	1.13E-55	3.52E-53
CNAG_04585	154.5088	0.674636	0.174782	3.85988	0.000113	0.00069
CNAG_04584	5054.068	-0.2007	0.080402	-2.49619	0.012554	0.040939
CNAG_04581	836.0134	-0.23166	0.088443	-2.61933	0.00881	0.030585
CNAG_04577	4951.614	0.267102	0.079703	3.351196	0.000805	0.003927
CNAG_07862	689.5208	0.204964	0.090604	2.2622	0.023685	0.068285
CNAG_07863	7902.03	-0.13535	0.062145	-2.17798	0.029407	0.081904
CNAG_04571	1138.344	-0.40946	0.082867	-4.94123	7.76E-07	6.87E-06
CNAG_04570	5456.346	-0.32619	0.080551	-4.04948	5.13E-05	0.000337
CNAG_07864	2116.824	-0.44242	0.074538	-5.93552	2.93E-09	3.68E-08
CNAG_07866	359.707	0.353407	0.143969	2.454746	0.014098	0.044853
CNAG_04565	1179.4	0.210818	0.083129	2.536024	0.011212	0.037201
CNAG_04564	435.2407	0.401767	0.11126	3.611059	0.000305	0.001671
CNAG_08002	2727.153	-0.16836	0.077244	-2.17961	0.029287	0.081655

CNAG_01464	25755.58	2.542097	0.091581	27.75783	1.40E-169	3.65E-166
CNAG_12951	102.8894	0.914801	0.221253	4.134639	3.56E-05	0.00024
CNAG_01470	5921.94	0.255134	0.068673	3.715212	0.000203	0.001158
CNAG_01471	1847.962	0.353625	0.071857	4.92123	8.60E-07	7.53E-06
CNAG_01472	981.0237	0.267522	0.084164	3.178575	0.00148	0.006661
CNAG_01474	272.2896	-0.34904	0.13014	-2.68207	0.007317	0.026049
CNAG_01475	7658.858	-0.27411	0.062419	-4.39144	1.13E-05	8.44E-05
CNAG_01480	39813.78	-0.21974	0.076899	-2.85744	0.004271	0.016511
CNAG_01481	238.477	0.269911	0.13769	1.96028	0.049963	0.124453
CNAG_01485	3531.438	0.159148	0.072718	2.188552	0.028629	0.080333
CNAG_01486	56246.86	-0.21054	0.075552	-2.78669	0.005325	0.019798
CNAG_07587	80.15747	-0.56185	0.233938	-2.40171	0.016319	0.050436
CNAG_01489	188.2388	-0.4036	0.150712	-2.67795	0.007408	0.026312
CNAG_01490	375.9664	-0.64958	0.117362	-5.53483	3.12E-08	3.39E-07
CNAG_01491	1637.925	0.266614	0.074851	3.561927	0.000368	0.00198
CNAG_01493	468.9406	1.121695	0.125014	8.972525	2.90E-19	9.71E-18
CNAG_12958	31.2037	1.255061	0.393947	3.185864	0.001443	0.006525
CNAG_01495	685.4053	-0.60288	0.099457	-6.06176	1.35E-09	1.76E-08
CNAG_01498	643.5506	-0.2333	0.094934	-2.45752	0.01399	0.044562
CNAG_01501	351.7066	0.295301	0.122663	2.407416	0.016066	0.049813
CNAG_01500	5.39868	-1.91447	0.971157	-1.97133	0.048687	0.121974
CNAG_01504	1281.122	0.295539	0.082712	3.57312	0.000353	0.001906
CNAG_08003	563.5664	-0.29133	0.104069	-2.79936	0.00512	0.019156
CNAG_01508	1619.176	-0.23597	0.07558	-3.12212	0.001796	0.007846
CNAG_01509	644.5968	-0.31695	0.099038	-3.20024	0.001373	0.006245
CNAG_01510	2918.21	0.169571	0.067345	2.517944	0.011804	0.038853
CNAG_07589	55.93681	-1.24418	0.28622	-4.34694	1.38E-05	0.000102
CNAG_07591	1540.376	0.426191	0.07833	5.440953	5.30E-08	5.57E-07
CNAG_01519	349.2261	0.258633	0.119056	2.172359	0.029829	0.082811
CNAG_01520	1307.303	-0.21236	0.076205	-2.78675	0.005324	0.019798
CNAG_01521	672.1847	0.365598	0.102316	3.573217	0.000353	0.001906
CNAG_01522	1703.212	0.456551	0.092456	4.938018	7.89E-07	6.97E-06
CNAG_01524	1621.697	-0.17436	0.075144	-2.32035	0.020322	0.060394
CNAG_01526	2993.557	0.201372	0.071982	2.797516	0.00515	0.019257
CNAG_12968	38.93687	0.792526	0.328302	2.414016	0.015778	0.049114
CNAG_01528	1029.392	-0.36473	0.08359	-4.36331	1.28E-05	9.51E-05
CNAG_01529	1506.053	0.554425	0.084367	6.571561	4.98E-11	7.74E-10
CNAG_01530	2666.866	-0.19533	0.08083	-2.41656	0.015668	0.04885
CNAG_01534	100.4065	-0.59693	0.21306	-2.80171	0.005083	0.019072
CNAG_01538	192.5798	-0.38752	0.158872	-2.43923	0.014719	0.046515
CNAG_01539	11338.02	0.34469	0.080877	4.261875	2.03E-05	0.000143
CNAG_01540	556.3178	-0.3035	0.105188	-2.88528	0.003911	0.015406
CNAG_01543	1399.701	0.290322	0.086083	3.372591	0.000745	0.003683
CNAG_01545	316.6877	-0.52011	0.128604	-4.04427	5.25E-05	0.000344
CNAG_01546	368.9167	-0.32924	0.124885	-2.63633	0.008381	0.029274
CNAG_01547	2059.195	-0.19613	0.072723	-2.69699	0.006997	0.025071
CNAG_01548	5624.871	-0.19313	0.062117	-3.10911	0.001877	0.008163
CNAG_01549	3066.667	-0.13648	0.068548	-1.99094	0.046487	0.117472

CNAG_01550	1361.35	-0.27747	0.076525	-3.62581	0.000288	0.001595
CNAG_01553	1072.925	-0.40477	0.084104	-4.81275	1.49E-06	1.27E-05
CNAG_01557	8954.141	0.157364	0.073799	2.132324	0.03298	0.089277
CNAG_01558	21547.27	1.310809	0.080132	16.35822	3.80E-60	1.29E-57
CNAG_01559	1905.852	-0.19367	0.076822	-2.52101	0.011702	0.038634
CNAG_01562	30829.86	0.238742	0.098066	2.434514	0.014912	0.047019
CNAG_01564	2995.909	-0.58615	0.074692	-7.84747	4.24E-15	9.83E-14
CNAG_01565	2762.011	0.173466	0.069757	2.486729	0.012892	0.04183
CNAG_01567	181.7357	0.631003	0.153854	4.101314	4.11E-05	0.000274
CNAG_01569	928.4466	0.38417	0.085876	4.473521	7.69E-06	5.92E-05
CNAG_01571	944.5061	0.166335	0.084478	1.968967	0.048957	0.122416
CNAG_01572	571.746	0.425996	0.110563	3.852965	0.000117	0.000707
CNAG_01573	1897.377	0.238432	0.07992	2.9834	0.002851	0.011721
CNAG_01574	3611.734	0.661125	0.067698	9.76585	1.58E-22	7.04E-21
CNAG_01576	1632.187	0.175605	0.076632	2.291535	0.021933	0.064226
CNAG_01577	39339.86	0.26001	0.068126	3.816604	0.000135	0.000806
CNAG_01578	2203.538	0.22059	0.085094	2.592312	0.009533	0.032552
CNAG_01584	1008.751	0.731425	0.090304	8.099614	5.51E-16	1.36E-14
CNAG_01585	662.5258	0.847679	0.099663	8.505409	1.81E-17	4.97E-16
CNAG_01586	16727.67	0.257681	0.078077	3.300336	0.000966	0.004611
CNAG_01588	5010.773	0.465005	0.073631	6.315326	2.70E-10	3.88E-09
CNAG_01590	972.7519	0.275106	0.081824	3.362155	0.000773	0.003804
CNAG_01593	3054.851	-0.26142	0.079517	-3.28755	0.001011	0.004803
CNAG_01594	4847.249	0.452744	0.074769	6.055277	1.40E-09	1.83E-08
CNAG_01597	1084.675	0.408187	0.086892	4.697651	2.63E-06	2.17E-05
CNAG_01600	1562.321	-0.60853	0.096817	-6.28542	3.27E-10	4.64E-09
CNAG_01604	546.8502	0.681981	0.102424	6.658436	2.77E-11	4.43E-10
CNAG_01605	350.8729	0.411365	0.134877	3.049929	0.002289	0.009656
CNAG_01608	1415.545	-0.45966	0.081736	-5.62378	1.87E-08	2.09E-07
CNAG_01615	808.8551	0.21434	0.10262	2.088682	0.036736	0.09716
CNAG_01616	2769.715	-0.18927	0.078009	-2.42621	0.015257	0.047934
CNAG_07594	1186.031	0.398681	0.078422	5.083791	3.70E-07	3.45E-06
CNAG_01619	624.5736	0.322104	0.0979	3.290126	0.001001	0.004762
CNAG_12978	93.47274	1.157261	0.229895	5.033869	4.81E-07	4.43E-06
CNAG_01621	11391.06	3.284435	0.085464	38.43044	0	0
CNAG_01622	800.4136	-0.28607	0.088741	-3.22361	0.001266	0.005814
CNAG_01623	989.0109	-0.42285	0.097764	-4.32517	1.52E-05	0.000111
CNAG_01629	684.7445	-0.27944	0.099061	-2.82088	0.004789	0.018152
CNAG_01631	1543.259	-0.14556	0.073681	-1.97549	0.048212	0.121038
CNAG_01632	582.0845	0.267685	0.097873	2.735026	0.006238	0.022694
CNAG_01634	3512.732	-0.36307	0.06542	-5.54993	2.86E-08	3.13E-07
CNAG_01636	1184.568	-0.16443	0.083499	-1.96924	0.048925	0.122416
CNAG_01638	824.0546	-0.42226	0.094148	-4.4851	7.29E-06	5.63E-05
CNAG_01639	7009.463	0.510994	0.07673	6.659644	2.74E-11	4.41E-10
CNAG_01641	4541.884	-0.38388	0.074854	-5.12833	2.92E-07	2.79E-06
CNAG_01643	973.5021	-0.34989	0.086078	-4.06477	4.81E-05	0.000317
CNAG_01644	2172.336	-0.23013	0.072745	-3.16345	0.001559	0.006953
CNAG_01646	850.3099	-0.19103	0.088383	-2.16144	0.030662	0.084523

CNAG_01653	142.7356	-0.45098	0.199355	-2.26218	0.023686	0.068285
CNAG_01654	1131.069	-0.33037	0.090001	-3.67077	0.000242	0.001364
CNAG_01656	541.6764	0.487224	0.10644	4.577442	4.71E-06	3.72E-05
CNAG_01657	8907.886	0.474733	0.06697	7.088769	1.35E-12	2.53E-11
CNAG_08004	125.4834	0.417223	0.19549	2.134248	0.032822	0.089002
CNAG_01665	727.6429	-0.28014	0.091136	-3.07386	0.002113	0.009006
CNAG_01673	1662.99	-0.23454	0.09226	-2.54211	0.011018	0.036684
CNAG_01674	59.21388	-0.53167	0.256699	-2.07119	0.038341	0.100711
CNAG_01675	880.1141	-0.25388	0.091264	-2.7818	0.005406	0.020051
CNAG_01676	1695.894	-0.48224	0.087624	-5.50355	3.72E-08	4.02E-07
CNAG_01679	4062.123	-0.18814	0.066734	-2.81922	0.004814	0.018237
CNAG_12984	26.08708	0.838062	0.392029	2.137754	0.032537	0.088322
CNAG_12985	1169.715	0.299003	0.091758	3.258585	0.00112	0.005232
CNAG_01681	6447.551	-0.20779	0.061689	-3.36836	0.000756	0.003732
CNAG_01682	11774.08	0.199351	0.057562	3.463206	0.000534	0.002764
CNAG_12988	249.2817	-0.43093	0.136362	-3.16017	0.001577	0.00702
CNAG_01683	10569.82	0.276428	0.0647	4.272427	1.93E-05	0.000137
CNAG_01686	2477.27	0.559411	0.079507	7.035975	1.98E-12	3.62E-11
CNAG_01687	1977.129	-0.44919	0.077807	-5.7731	7.78E-09	9.24E-08
CNAG_01691	1193.256	0.622453	0.089174	6.980228	2.95E-12	5.22E-11
CNAG_12992	137.0998	0.432918	0.176614	2.451213	0.014238	0.04524
CNAG_01694	870.1026	-0.3416	0.131239	-2.60289	0.009244	0.031817
CNAG_01696	4301.246	0.294663	0.071977	4.093853	4.24E-05	0.000282
CNAG_08005	149.0841	0.574309	0.175018	3.281435	0.001033	0.004892
CNAG_01708	1078.916	-0.30623	0.100673	-3.04187	0.002351	0.009875
CNAG_01709	4242.506	-0.24413	0.066502	-3.67101	0.000242	0.001364
CNAG_01711	4273.93	0.17461	0.062212	2.806682	0.005005	0.018834
CNAG_01712	1115.619	-0.35244	0.088544	-3.98039	6.88E-05	0.000442
CNAG_01713	1661.867	0.679268	0.086268	7.873951	3.44E-15	8.03E-14
CNAG_01715	3709.991	-0.55784	0.086629	-6.43943	1.20E-10	1.79E-09
CNAG_01721	3512.759	0.352225	0.079247	4.444636	8.80E-06	6.70E-05
CNAG_01726	5335.235	-0.18007	0.074298	-2.42365	0.015366	0.048138
CNAG_01727	44008.05	-0.25773	0.070331	-3.66452	0.000248	0.001393
CNAG_01728	1207.656	0.378471	0.078943	4.794214	1.63E-06	1.39E-05
CNAG_01729	1473.408	-0.71571	0.093362	-7.66594	1.78E-14	3.91E-13
CNAG_01730	621.8661	0.242259	0.100349	2.414167	0.015771	0.049114
CNAG_12998	20.62416	0.933476	0.434769	2.147061	0.031788	0.086815
CNAG_01732	1675.243	0.23106	0.078251	2.952796	0.003149	0.012767
CNAG_01735	1902.65	0.440252	0.077944	5.648279	1.62E-08	1.84E-07
CNAG_01737	3657.343	-0.70925	0.078345	-9.05284	1.39E-19	4.92E-18
CNAG_01738	456.36	-0.3229	0.11288	-2.86059	0.004229	0.016385
CNAG_01742	180.1257	1.018947	0.156347	6.517201	7.16E-11	1.10E-09
CNAG_01744	11131.68	0.298231	0.069577	4.28636	1.82E-05	0.000131
CNAG_01745	11322.59	0.431255	0.061949	6.961432	3.37E-12	5.95E-11
CNAG_01746	296.4644	-0.38812	0.136214	-2.84934	0.004381	0.01685
CNAG_07599	1133.941	-0.2092	0.088699	-2.35857	0.018346	0.055428
CNAG_01749	693.0057	-0.78368	0.103099	-7.60125	2.93E-14	6.34E-13
CNAG_01751	1044.635	0.784926	0.106985	7.336771	2.19E-13	4.48E-12

CNAG_01752	7352.948	0.147432	0.059904	2.461153	0.013849	0.044204
CNAG_01753	5899.009	0.538409	0.07246	7.430427	1.08E-13	2.25E-12
CNAG_01754	1714.531	-0.24577	0.075186	-3.26885	0.00108	0.005073
CNAG_01755	3037.444	-0.4547	0.07177	-6.33552	2.37E-10	3.42E-09
CNAG_01756	738.5464	-0.25821	0.092609	-2.78814	0.005301	0.019747
CNAG_08006	229.2531	-0.3999	0.142323	-2.80981	0.004957	0.018688
CNAG_07601	497.2994	-0.32337	0.113703	-2.84395	0.004456	0.017104
CNAG_01761	20527.06	0.826015	0.066959	12.33621	5.78E-35	5.57E-33
CNAG_01764	813.3889	-0.29634	0.099656	-2.97365	0.002943	0.012049
CNAG_01765	1147.517	-0.32645	0.084158	-3.87901	0.000105	0.000644
CNAG_01769	3220.512	0.395722	0.073874	5.356735	8.47E-08	8.67E-07
CNAG_01772	3395.297	0.152768	0.066351	2.302428	0.021311	0.06283
CNAG_01777	1885.56	0.389714	0.081774	4.765726	1.88E-06	1.58E-05
CNAG_01779	771.385	0.248113	0.092885	2.671193	0.007558	0.026813
CNAG_01780	4801.815	-0.27229	0.079858	-3.40965	0.00065	0.003279
CNAG_01788	1912.935	-0.29128	0.080281	-3.62829	0.000285	0.001586
CNAG_01790	846.9417	-0.26025	0.086056	-3.0242	0.002493	0.01042
CNAG_01791	1989.866	-0.51649	0.076541	-6.74782	1.50E-11	2.51E-10
CNAG_01792	1428.294	-0.33154	0.094041	-3.52553	0.000423	0.002242
CNAG_13006	128.4006	-0.785	0.187963	-4.17636	2.96E-05	0.000203
CNAG_01793	652.1761	-0.3888	0.096497	-4.02916	5.60E-05	0.000365
CNAG_01794	368.8807	-1.86192	0.141017	-13.2035	8.37E-40	1.13E-37
CNAG_01795	385.4634	0.269304	0.117923	2.283725	0.022388	0.065216
CNAG_01799	10182.6	0.16224	0.074864	2.167115	0.030226	0.083617
CNAG_01800	1917.121	0.306008	0.087766	3.486634	0.000489	0.00256
CNAG_01803	450.5587	-0.29256	0.126372	-2.31508	0.020608	0.061058
CNAG_01807	860.1585	-0.42285	0.118827	-3.55852	0.000373	0.002
CNAG_01808	2420.485	0.214891	0.070611	3.043294	0.00234	0.009834
CNAG_01810	547.2964	-0.56517	0.102477	-5.51511	3.49E-08	3.78E-07
CNAG_01812	3593.092	-0.34271	0.076757	-4.46488	8.01E-06	6.14E-05
CNAG_01813	11597.53	-0.15733	0.071937	-2.18708	0.028737	0.080533
CNAG_01818	1740.336	0.202945	0.078912	2.571793	0.010117	0.034172
CNAG_01821	933.6063	0.386085	0.103058	3.746286	0.000179	0.001037
CNAG_01822	1937.025	0.477328	0.075342	6.335521	2.37E-10	3.42E-09
CNAG_01823	2922.339	0.191292	0.071085	2.691051	0.007123	0.025428
CNAG_13008	157.0175	-0.8543	0.172298	-4.95828	7.11E-07	6.33E-06
CNAG_07608	1510.373	-0.26995	0.080147	-3.36822	0.000757	0.003732
CNAG_07609	1547.856	-0.5874	0.074711	-7.86229	3.77E-15	8.76E-14
CNAG_01833	3874.384	-0.26453	0.067325	-3.92919	8.52E-05	0.000535
CNAG_01836	128.4075	-0.3816	0.193943	-1.96758	0.049117	0.122776
CNAG_13010	151.9142	0.377381	0.162735	2.31899	0.020396	0.060543
CNAG_01839	2905.109	0.153927	0.070866	2.172072	0.02985	0.082842
CNAG_01842	1861.129	-0.52645	0.077023	-6.83494	8.20E-12	1.41E-10
CNAG_01843	2674.274	0.607146	0.077167	7.867943	3.61E-15	8.40E-14
CNAG_01844	304.8015	-0.34071	0.131686	-2.58732	0.009673	0.032977
CNAG_01846	6351.753	1.151701	0.073945	15.57516	1.07E-54	2.99E-52
CNAG_01850	2789.441	-0.19943	0.08237	-2.42117	0.01547	0.048409
CNAG_01851	37.67881	1.108748	0.319911	3.4658	0.000529	0.002744

CNAG_01860	759.0953	0.195398	0.090662	2.155238	0.031143	0.085514
CNAG_13013	191.5037	0.465943	0.156942	2.968886	0.002989	0.012212
CNAG_01865	492.2717	-0.72559	0.108824	-6.66757	2.60E-11	4.22E-10
CNAG_01866	517.9023	-0.37504	0.118111	-3.17533	0.001497	0.006717
CNAG_01867	2263.843	0.218293	0.070982	3.075328	0.002103	0.008967
CNAG_01870	4867.491	0.333375	0.076049	4.383667	1.17E-05	8.73E-05
CNAG_01874	1428.134	0.235389	0.107829	2.182977	0.029037	0.081105
CNAG_01876	1426.028	0.473367	0.086995	5.441308	5.29E-08	5.57E-07
CNAG_01877	9215.08	-0.42314	0.075094	-5.63488	1.75E-08	1.98E-07
CNAG_01880	1412.552	-0.19579	0.086977	-2.25111	0.024379	0.070074
CNAG_01881	5100.012	1.048402	0.068014	15.41443	1.31E-53	3.30E-51
CNAG_01883	91.90509	-0.91467	0.220553	-4.14716	3.37E-05	0.000228
CNAG_01884	65613.32	-0.16259	0.080197	-2.02734	0.042628	0.109937
CNAG_01885	1353.718	-0.39783	0.081532	-4.87947	1.06E-06	9.19E-06
CNAG_01890	54408.34	-0.22875	0.058472	-3.91223	9.14E-05	0.000567
CNAG_01892	1851.335	0.371512	0.097253	3.820044	0.000133	0.000796
CNAG_01893	1800.506	0.254586	0.079166	3.215859	0.001301	0.005958
CNAG_01894	2784.456	-0.14813	0.070339	-2.10587	0.035216	0.094041
CNAG_01898	2250.626	-0.4757	0.071272	-6.67448	2.48E-11	4.05E-10
CNAG_01900	1123.55	0.361555	0.08439	4.284347	1.83E-05	0.000132
CNAG_01902	2931.502	-0.31572	0.068882	-4.58344	4.57E-06	3.64E-05
CNAG_01907	1914.979	-0.30538	0.120363	-2.53716	0.011176	0.037097
CNAG_13021	375.7217	0.548075	0.134377	4.078654	4.53E-05	0.0003
CNAG_01914	1552.272	0.330539	0.078153	4.229384	2.34E-05	0.000163
CNAG_01915	9261.154	0.49094	0.074043	6.630458	3.35E-11	5.29E-10
CNAG_01919	791.2906	0.242586	0.096901	2.503445	0.012299	0.040177
CNAG_01920	13995.14	-0.39367	0.067559	-5.82705	5.64E-09	6.84E-08
CNAG_01921	94.28011	0.634562	0.203934	3.111597	0.001861	0.008104
CNAG_01922	1148.569	0.336943	0.080044	4.209481	2.56E-05	0.000178
CNAG_01923	2339.581	-0.21964	0.068673	-3.19841	0.001382	0.006266
CNAG_01924	1193	0.665782	0.081418	8.177359	2.90E-16	7.40E-15
CNAG_01925	1027.715	1.383639	0.130282	10.62038	2.40E-26	1.43E-24
CNAG_01926	473.4197	0.323604	0.112735	2.870479	0.004099	0.015993
CNAG_01929	245.6949	-0.58558	0.14062	-4.16428	3.12E-05	0.000213
CNAG_01937	91.63702	-0.47434	0.220313	-2.15301	0.031318	0.085877
CNAG_01939	178.2005	0.521978	0.183413	2.845924	0.004428	0.017007
CNAG_01941	1677.622	-0.15869	0.079384	-1.99903	0.045605	0.115903
CNAG_01942	403.6559	0.707366	0.118564	5.966126	2.43E-09	3.09E-08
CNAG_01943	2714.954	-0.19751	0.075608	-2.61232	0.008993	0.031038
CNAG_01945	85.64949	0.437455	0.219691	1.99123	0.046456	0.117441
CNAG_01946	1366.639	1.388791	0.079043	17.57016	4.17E-69	2.32E-66
CNAG_01947	570.4618	-0.9859	0.114881	-8.58192	9.33E-18	2.66E-16
CNAG_01948	1001.905	-0.35268	0.093309	-3.77975	0.000157	0.00092
CNAG_01949	1236.523	-1.23587	0.089574	-13.7971	2.65E-43	4.14E-41
CNAG_01952	446.3083	0.449765	0.116034	3.876161	0.000106	0.000649
CNAG_01953	230.1651	-0.87162	0.163434	-5.33318	9.65E-08	9.80E-07
CNAG_13035	32.48194	-0.86781	0.361102	-2.40322	0.016251	0.050249
CNAG_13036	50.38923	-0.88544	0.281158	-3.14926	0.001637	0.00725

CNAG_01957	3160.701	-0.28977	0.072414	-4.00166	6.29E-05	0.000407
CNAG_01958	2893.187	-0.21604	0.066763	-3.23593	0.001212	0.005602
CNAG_01959	2285.706	-0.30881	0.098036	-3.14999	0.001633	0.00724
CNAG_01961	6069.674	0.156788	0.067777	2.313274	0.020708	0.061329
CNAG_01962	996.1835	0.27524	0.091056	3.022748	0.002505	0.010462
CNAG_13037	50.24673	1.556525	0.318214	4.891449	1.00E-06	8.69E-06
CNAG_01970	1897.529	0.433518	0.072158	6.007931	1.88E-09	2.41E-08
CNAG_01974	1040.867	0.215211	0.086352	2.492262	0.012693	0.041285
CNAG_01976	34288	-0.18854	0.073133	-2.57797	0.009938	0.033683
CNAG_01981	6480.797	-0.34368	0.066618	-5.15897	2.48E-07	2.39E-06
CNAG_01982	573.7327	0.366735	0.110335	3.32383	0.000888	0.004281
CNAG_01984	52940.18	0.556718	0.060398	9.217553	3.04E-20	1.12E-18
CNAG_01985	5473.857	0.160272	0.065857	2.433612	0.014949	0.047098
CNAG_01986	1161.598	0.252836	0.084637	2.987315	0.002814	0.011603
CNAG_13042	78.31475	0.813608	0.236198	3.444602	0.000572	0.00294
CNAG_01987	1802.388	0.203431	0.083807	2.427365	0.015209	0.047801
CNAG_01989	251.2613	0.31817	0.145898	2.180765	0.029201	0.081532
CNAG_01990	51349.43	-0.19812	0.078075	-2.53758	0.011162	0.037073
CNAG_01991	11756.27	0.268798	0.073189	3.672671	0.00024	0.001356
CNAG_01993	153.0393	0.399556	0.166212	2.403898	0.016221	0.050195
CNAG_01996	441.3278	-0.3615	0.134082	-2.69609	0.007016	0.025116
CNAG_01997	2986.696	-0.27083	0.074442	-3.63817	0.000275	0.00153
CNAG_02000	1958.509	0.308122	0.080639	3.821017	0.000133	0.000794
CNAG_02004	2797.411	0.182919	0.068821	2.657905	0.007863	0.027803
CNAG_02006	1323.295	0.332319	0.084965	3.911227	9.18E-05	0.000569
CNAG_02008	2291.148	0.454025	0.101042	4.493429	7.01E-06	5.43E-05
CNAG_02009	1023.195	0.380962	0.098199	3.879507	0.000105	0.000643
CNAG_02011	281.6375	-0.31181	0.128453	-2.42744	0.015206	0.047801
CNAG_07619	1039.805	0.419151	0.094511	4.434925	9.21E-06	6.99E-05
CNAG_02016	12.24353	-2.01169	0.63478	-3.16911	0.001529	0.006838
CNAG_07026	45.85985	1.036309	0.299635	3.458574	0.000543	0.002807
CNAG_05986	52.6927	0.531706	0.265982	1.99903	0.045605	0.115903
CNAG_05989	777.6945	0.254947	0.08912	2.860715	0.004227	0.016385
CNAG_05990	396.0123	0.266481	0.119984	2.220968	0.026353	0.074894
CNAG_05992	149.1987	0.405095	0.171231	2.365785	0.017992	0.054613
CNAG_05993	886.0099	0.820564	0.087828	9.34282	9.38E-21	3.64E-19
CNAG_05994	2147.641	0.943503	0.095737	9.855132	6.51E-23	3.08E-21
CNAG_06000	2493.801	0.656138	0.069652	9.420263	4.50E-21	1.80E-19
CNAG_06001	1070.688	-0.36073	0.083815	-4.30389	1.68E-05	0.000121
CNAG_06005	1991.485	-0.69154	0.074654	-9.26332	1.98E-20	7.47E-19
CNAG_06006	1280.428	-0.18436	0.087668	-2.10293	0.035472	0.094543
CNAG_06007	1103.346	-0.60894	0.084236	-7.22902	4.87E-13	9.54E-12
CNAG_06009	88.1688	0.723231	0.234025	3.090397	0.001999	0.008604
CNAG_06011	85.50082	-0.61631	0.24785	-2.48662	0.012896	0.04183
CNAG_06012	4908.301	0.216859	0.073867	2.935808	0.003327	0.013417
CNAG_06013	638.8945	0.231728	0.104234	2.223147	0.026206	0.074557
CNAG_06018	1336.097	0.182546	0.090812	2.010151	0.044415	0.113645
CNAG_06022	511.8131	0.268015	0.115178	2.326952	0.019968	0.059545

CNAG_06026	1621.651	-0.16087	0.080614	-1.99551	0.045987	0.116557
CNAG_06029	411.3766	-0.42895	0.108888	-3.93935	8.17E-05	0.000516
CNAG_06030	561.3421	0.229972	0.103292	2.226435	0.025985	0.07401
CNAG_06031	274.8192	-0.38307	0.129816	-2.95083	0.003169	0.012842
CNAG_06032	582.2685	0.227487	0.105498	2.156312	0.031059	0.085347
CNAG_06033	1065.05	-0.88204	0.098811	-8.92655	4.39E-19	1.44E-17
CNAG_06048	1186.222	0.221256	0.083763	2.641461	0.008255	0.029019
CNAG_13055	78.66051	0.802679	0.229554	3.496686	0.000471	0.002474
CNAG_06050	187.2083	0.968027	0.173735	5.571874	2.52E-08	2.77E-07
CNAG_06051	1012.147	0.46369	0.086396	5.367014	8.01E-08	8.21E-07
CNAG_06052	1164.869	0.580289	0.093548	6.203099	5.54E-10	7.55E-09
CNAG_07897	109.483	0.484705	0.217566	2.227852	0.02589	0.073821
CNAG_13058	12.0326	-1.29071	0.574992	-2.24474	0.024785	0.07111
CNAG_06061	5724.819	-0.19758	0.064537	-3.06144	0.002203	0.009332
CNAG_06063	2636.711	1.407243	0.072405	19.43576	3.85E-84	3.75E-81
CNAG_06064	951.1739	0.490513	0.090332	5.430086	5.63E-08	5.90E-07
CNAG_06068	1958.584	-0.30653	0.079469	-3.85727	0.000115	0.000695
CNAG_07902	1358.902	0.53374	0.086012	6.205438	5.45E-10	7.48E-09
CNAG_06071	568.5196	-0.23066	0.104344	-2.21059	0.027064	0.076695
CNAG_06073	57.15661	-0.76528	0.267212	-2.86393	0.004184	0.016262
CNAG_06074	1214.771	1.048819	0.103313	10.15183	3.25E-24	1.73E-22
CNAG_06075	11593.76	0.945746	0.078229	12.08942	1.20E-33	1.05E-31
CNAG_06076	2462.182	-0.20406	0.081439	-2.50566	0.012222	0.039959
CNAG_06078	888.082	0.320566	0.092334	3.471811	0.000517	0.002691
CNAG_06081	7688.584	-0.40803	0.08126	-5.02128	5.13E-07	4.72E-06
CNAG_13062	124.5244	-3.29447	0.257742	-12.782	2.07E-37	2.48E-35
CNAG_06082	82.42895	-4.42006	0.374413	-11.8053	3.66E-32	2.98E-30
CNAG_06085	766.6134	0.510334	0.097809	5.217655	1.81E-07	1.77E-06
CNAG_06087	1609.556	-0.15334	0.077063	-1.98979	0.046614	0.11765
CNAG_06088	4246.607	0.15404	0.073469	2.096662	0.036024	0.095785
CNAG_06090	2778.83	-0.35172	0.070405	-4.9956	5.87E-07	5.32E-06
CNAG_06095	90047.36	-0.24887	0.068255	-3.64624	0.000266	0.001488
CNAG_06096	11786.51	-0.25344	0.06331	-4.0031	6.25E-05	0.000405
CNAG_06097	504.1273	-0.25768	0.118485	-2.17482	0.029644	0.082407
CNAG_06098	722.5889	0.43259	0.103819	4.166786	3.09E-05	0.000211
CNAG_06100	874.1988	0.189365	0.095992	1.972722	0.048527	0.121653
CNAG_06101	136546.9	0.330018	0.070778	4.662708	3.12E-06	2.54E-05
CNAG_13067	110.8122	-0.88568	0.204022	-4.34108	1.42E-05	0.000104
CNAG_06102	89.8804	-0.46024	0.210441	-2.18704	0.02874	0.080533
CNAG_06105	116.8514	-0.56878	0.237315	-2.39673	0.016542	0.050985
CNAG_06107	855.5587	-0.23485	0.096255	-2.43989	0.014692	0.046457
CNAG_06109	13557.05	1.137073	0.09475	12.00071	3.52E-33	3.05E-31
CNAG_07903	931.9005	0.217712	0.111007	1.961251	0.04985	0.12425
CNAG_06112	13817.14	0.203873	0.064036	3.183741	0.001454	0.006558
CNAG_06113	69847.18	-0.24009	0.076386	-3.14317	0.001671	0.00739
CNAG_06114	2963.808	-0.25631	0.069849	-3.66948	0.000243	0.001369
CNAG_06115	374.5759	0.303876	0.118515	2.564036	0.010346	0.034773
CNAG_08016	1619.872	0.218175	0.099694	2.188444	0.028637	0.080333

CNAG_06119	173.05	-0.36494	0.171054	-2.13347	0.032886	0.089081
CNAG_06121	1248.668	0.192872	0.096398	2.000786	0.045415	0.115598
CNAG_06122	1011.282	-0.24736	0.098341	-2.51532	0.011893	0.039095
CNAG_06125	316427.1	-0.17056	0.066126	-2.57932	0.009899	0.033575
CNAG_06126	1360.571	-0.55715	0.076083	-7.32295	2.43E-13	4.96E-12
CNAG_06127	3246.753	-0.45207	0.072476	-6.23746	4.45E-10	6.19E-09
CNAG_06132	88.51604	-0.6215	0.230672	-2.69431	0.007053	0.025215
CNAG_06134	4924.965	-0.79272	0.063212	-12.5407	4.47E-36	4.72E-34
CNAG_06136	557.5783	-0.31375	0.120563	-2.60237	0.009258	0.031817
CNAG_06138	2634.7	0.205791	0.079575	2.586116	0.009706	0.033064
CNAG_06139	154.712	0.831071	0.167384	4.965051	6.87E-07	6.15E-06
CNAG_06144	28232.05	-0.14508	0.064376	-2.25356	0.024224	0.069706
CNAG_06149	466.1712	-0.28567	0.106954	-2.671	0.007563	0.026814
CNAG_06150	10023.67	-0.199	0.068752	-2.89447	0.003798	0.015007
CNAG_06152	408.8714	0.437907	0.116401	3.762065	0.000169	0.00098
CNAG_06153	4285.008	-0.32126	0.065803	-4.8822	1.05E-06	9.09E-06
CNAG_06154	708.168	0.292048	0.094347	3.095463	0.001965	0.008491
CNAG_06156	918.2017	-0.46189	0.128309	-3.59984	0.000318	0.001736
CNAG_06157	1140.34	-0.47109	0.084361	-5.58423	2.35E-08	2.60E-07
CNAG_06158	2140.65	0.276757	0.103854	2.664857	0.007702	0.027272
CNAG_06163	60.69805	0.848811	0.255374	3.323799	0.000888	0.004281
CNAG_06169	964.34	1.248855	0.098736	12.64847	1.14E-36	1.27E-34
CNAG_06173	740.5733	-0.32959	0.101301	-3.25355	0.00114	0.00531
CNAG_06174	2383.159	-0.34305	0.096934	-3.53903	0.000402	0.002141
CNAG_06175	4974.033	-0.22975	0.06933	-3.3139	0.00092	0.004418
CNAG_07908	5721.097	-0.15363	0.065643	-2.34038	0.019264	0.057733
CNAG_06186	559.7971	0.279737	0.102839	2.720154	0.006525	0.023608
CNAG_07911	293.8715	0.83017	0.127792	6.496286	8.23E-11	1.25E-09
CNAG_06188	702.2964	0.502166	0.100412	5.001033	5.70E-07	5.19E-06
CNAG_06189	34.85785	0.6827	0.329179	2.073946	0.038084	0.100138
CNAG_06190	1327.807	0.758286	0.083841	9.044347	1.51E-19	5.29E-18
CNAG_06192	1728.255	-0.17776	0.08185	-2.17174	0.029875	0.082853
CNAG_06197	1037.257	-0.19015	0.088832	-2.14059	0.032307	0.08794
CNAG_06200	901.8551	-0.25678	0.090956	-2.82313	0.004756	0.018087
CNAG_06201	1430.694	0.569053	0.097805	5.818242	5.95E-09	7.17E-08
CNAG_06203	168.5899	0.445211	0.162522	2.739398	0.006155	0.022446
CNAG_06204	4644.678	0.178333	0.082175	2.170168	0.029994	0.083064
CNAG_06205	3783.48	-0.285	0.063079	-4.51809	6.24E-06	4.85E-05
CNAG_06206	2559.782	-0.49224	0.07073	-6.95944	3.42E-12	6.02E-11
CNAG_13083	76.92001	0.502735	0.236269	2.127808	0.033353	0.090033
CNAG_06207	1598.042	1.648497	0.106244	15.51612	2.70E-54	7.02E-52
CNAG_06213	1475.504	0.358886	0.083464	4.299909	1.71E-05	0.000123
CNAG_06214	1724.896	0.261922	0.074508	3.515348	0.000439	0.002325
CNAG_06215	665.4307	-0.24864	0.095888	-2.59307	0.009512	0.03253
CNAG_06216	1006.652	0.450732	0.097985	4.600014	4.22E-06	3.38E-05
CNAG_06218	1053.773	0.240224	0.087189	2.755215	0.005865	0.02151
CNAG_06220	11784.82	1.062331	0.069599	15.26354	1.34E-52	3.26E-50
CNAG_06221	1115.738	-0.34228	0.085615	-3.99788	6.39E-05	0.000413

CNAG_06224	1332.479	-0.37776	0.090859	-4.15764	3.22E-05	0.000219
CNAG_06226	4588.918	0.251341	0.077204	3.255552	0.001132	0.005285
CNAG_06227	1295.691	0.234617	0.080885	2.900608	0.003724	0.014776
CNAG_06228	2205.571	-0.52355	0.08316	-6.29567	3.06E-10	4.37E-09
CNAG_06229	1483.291	-0.18561	0.076277	-2.43337	0.014959	0.04711
CNAG_13087	31.64338	-1.61694	0.374378	-4.31901	1.57E-05	0.000114
CNAG_06231	50082.39	-0.23805	0.073713	-3.22936	0.001241	0.005709
CNAG_06232	840.5581	0.594693	0.102266	5.815163	6.06E-09	7.28E-08
CNAG_06235	2389.741	-0.27421	0.07235	-3.79002	0.000151	0.000887
CNAG_06236	1608.781	-0.19616	0.080877	-2.42544	0.01529	0.048016
CNAG_06237	1657.692	-0.16191	0.080167	-2.01963	0.043422	0.111616
CNAG_06238	245.5819	1.235562	0.175542	7.038566	1.94E-12	3.56E-11
CNAG_06240	5751.625	-0.50866	0.066368	-7.66425	1.80E-14	3.95E-13
CNAG_06241	1661.084	-0.45258	0.086657	-5.22262	1.76E-07	1.73E-06
CNAG_06242	3592.687	-0.61871	0.073388	-8.4307	3.44E-17	9.34E-16
CNAG_06243	79.37952	-0.6116	0.224723	-2.72156	0.006497	0.023552
CNAG_06246	22603.71	-0.21664	0.064989	-3.3335	0.000858	0.004154
CNAG_06247	231.8637	0.401639	0.144181	2.785667	0.005342	0.019851
CNAG_06248	8624.878	0.84651	0.070616	11.98751	4.13E-33	3.54E-31
CNAG_06252	459.2874	-0.68277	0.112009	-6.09561	1.09E-09	1.44E-08
CNAG_07917	705.5617	-0.28963	0.101658	-2.84904	0.004385	0.016858
CNAG_06986	492.7433	-0.38321	0.108487	-3.5323	0.000412	0.002193
CNAG_06259	112.2623	-1.89654	0.207917	-9.12163	7.40E-20	2.66E-18
CNAG_06260	1596.2	-0.92961	0.07411	-12.5438	4.30E-36	4.60E-34
CNAG_13097	407.7283	0.346563	0.113769	3.046198	0.002318	0.009755
CNAG_07922	936.0917	-0.26871	0.08841	-3.03937	0.002371	0.009949
CNAG_06267	1609.643	1.223782	0.115725	10.57488	3.90E-26	2.27E-24
CNAG_13099	466.0541	0.259113	0.107143	2.418396	0.015589	0.048673
CNAG_06273	2332.404	-0.37719	0.099278	-3.79933	0.000145	0.000858
CNAG_06274	11105.42	0.486823	0.069019	7.053475	1.75E-12	3.21E-11
CNAG_13104	94.13849	-0.57145	0.244705	-2.33525	0.01953	0.058441
CNAG_06277	3416.323	-0.64518	0.068307	-9.44518	3.55E-21	1.43E-19
CNAG_06278	2507.48	-0.19063	0.091766	-2.07736	0.037768	0.09944
CNAG_06279	3500.593	-0.16088	0.066661	-2.41346	0.015802	0.04917
CNAG_06280	1454.933	-0.17152	0.083346	-2.05786	0.039603	0.103504
CNAG_06282	1559.427	0.282754	0.084122	3.361254	0.000776	0.003811
CNAG_06283	1175.223	-0.23482	0.085738	-2.73878	0.006167	0.022467
CNAG_06284	586.8316	-0.48568	0.117796	-4.12304	3.74E-05	0.000251
CNAG_06286	361.2818	0.758656	0.118952	6.377822	1.80E-10	2.63E-09
CNAG_06290	475.4346	0.666713	0.115503	5.772261	7.82E-09	9.28E-08
CNAG_06291	242.6414	0.383498	0.161628	2.372724	0.017657	0.053786
CNAG_06293	391.9879	0.273082	0.115029	2.374024	0.017595	0.053701
CNAG_06294	140.8121	0.56513	0.173393	3.259243	0.001117	0.005223
CNAG_06296	64.22347	0.728254	0.271759	2.679773	0.007367	0.026205
CNAG_06297	709.4637	1.756757	0.241136	7.285329	3.21E-13	6.40E-12
CNAG_06298	2969.625	1.833788	0.109343	16.77102	3.98E-63	1.63E-60
CNAG_06299	881.2592	-0.20856	0.087479	-2.3841	0.017121	0.052459
CNAG_06300	2092.847	-0.2513	0.081468	-3.0847	0.002038	0.008741

CNAG_06301	5405.068	0.238303	0.064274	3.707633	0.000209	0.001191
CNAG_06302	117.3824	0.501688	0.194057	2.58526	0.009731	0.033112
CNAG_06314	10198.85	-0.2569	0.065002	-3.95225	7.74E-05	0.000492
CNAG_06318	2363.295	-0.46592	0.074814	-6.22767	4.73E-10	6.56E-09
CNAG_06320	2769.489	-0.15231	0.076769	-1.984	0.047256	0.119039
CNAG_06323	411.2227	0.273783	0.11549	2.370615	0.017759	0.054037
CNAG_06329	196.0641	0.817278	0.163657	4.993846	5.92E-07	5.35E-06
CNAG_06332	1653.148	0.361897	0.08705	4.157346	3.22E-05	0.000219
CNAG_06336	811.6631	-0.20912	0.086432	-2.41943	0.015545	0.048563
CNAG_13117	24.03939	-1.16744	0.409707	-2.84944	0.00438	0.01685
CNAG_06338	341.6506	-0.33853	0.137183	-2.46774	0.013597	0.043505
CNAG_06340	2205.663	-0.406	0.079466	-5.10911	3.24E-07	3.06E-06
CNAG_06341	907.8548	-0.71792	0.084113	-8.53514	1.40E-17	3.90E-16
CNAG_06345	1023.695	-0.21419	0.092688	-2.31082	0.020843	0.061659
CNAG_13120	285.242	0.433005	0.134132	3.228203	0.001246	0.005729
CNAG_06347	13564.62	0.804341	0.098121	8.197419	2.46E-16	6.30E-15
CNAG_06348	1342.076	-0.70335	0.093578	-7.51622	5.64E-14	1.19E-12
CNAG_06350	1575.143	-0.49552	0.076412	-6.48478	8.89E-11	1.35E-09
CNAG_06351	2427.681	-0.18832	0.072044	-2.614	0.008949	0.030929
CNAG_06353	3134.282	-0.18813	0.065548	-2.87014	0.004103	0.015993
CNAG_06356	1561.39	0.27664	0.075169	3.680249	0.000233	0.001319
CNAG_06360	1179.93	0.21203	0.083717	2.532698	0.011319	0.037508
CNAG_06361	3442.365	-0.14728	0.068269	-2.15738	0.030976	0.085209
CNAG_06363	1224.099	-0.52922	0.081964	-6.45672	1.07E-10	1.61E-09
CNAG_06364	5004.111	0.260834	0.089961	2.899394	0.003739	0.014811
CNAG_06367	3503.42	-0.55759	0.068808	-8.10361	5.34E-16	1.32E-14
CNAG_06371	311.091	0.315129	0.124624	2.528641	0.011451	0.037896
CNAG_06372	204.4283	0.704117	0.157674	4.465639	7.98E-06	6.13E-05
CNAG_06374	1749.606	0.912364	0.111684	8.169187	3.10E-16	7.84E-15
CNAG_06377	45024.72	0.515119	0.074791	6.887421	5.68E-12	9.87E-11
CNAG_06378	527.0394	-0.24976	0.106214	-2.35148	0.018699	0.056279
CNAG_06379	3283.255	-0.63155	0.068932	-9.16201	5.09E-20	1.85E-18
CNAG_06381	8887.266	0.536153	0.066632	8.046421	8.53E-16	2.09E-14
CNAG_06385	810.0598	-0.2766	0.099658	-2.77551	0.005512	0.020396
CNAG_06388	5350.99	1.433442	0.087276	16.42428	1.28E-60	4.76E-58
CNAG_06389	141.1413	-0.4023	0.173412	-2.31992	0.020345	0.060439
CNAG_06392	1123.729	0.170406	0.079315	2.148465	0.031677	0.086617
CNAG_13125	22.19235	1.065925	0.414642	2.570711	0.010149	0.034257
CNAG_06396	354.7988	1.05265	0.128826	8.171091	3.06E-16	7.77E-15
CNAG_06399	658.5004	0.317261	0.094522	3.356466	0.000789	0.003863
CNAG_06400	84469.46	0.413276	0.060643	6.814864	9.44E-12	1.61E-10
CNAG_13126	60.27702	-0.63786	0.285936	-2.23078	0.025696	0.073374
CNAG_06402	1748.651	-0.24659	0.087036	-2.83322	0.004608	0.017611
CNAG_06403	1624.624	0.187683	0.079186	2.370157	0.017781	0.054076
CNAG_06404	5447.968	1.422334	0.084064	16.91972	3.22E-64	1.48E-61
CNAG_06405	925.36	-0.25524	0.092304	-2.76525	0.005688	0.020929
CNAG_06407	3363.323	0.342728	0.082988	4.129857	3.63E-05	0.000244
CNAG_06409	574.1689	0.753548	0.118075	6.38195	1.75E-10	2.57E-09

CNAG_06410	1203.231	0.308531	0.081405	3.790082	0.000151	0.000887
CNAG_06412	1005.102	-0.71569	0.082006	-8.72726	2.61E-18	7.92E-17
CNAG_06420	3477.073	-0.19894	0.070356	-2.82767	0.004689	0.017867
CNAG_06422	3301.558	0.226218	0.092356	2.449403	0.014309	0.045386
CNAG_06424	927.1685	-0.29124	0.090554	-3.21621	0.001299	0.005956
CNAG_06428	1077.459	0.387472	0.099905	3.878419	0.000105	0.000645
CNAG_06432	4149.821	1.063227	0.086531	12.2872	1.06E-34	1.01E-32
CNAG_07939	11131.59	0.898885	0.095933	9.369955	7.26E-21	2.86E-19
CNAG_06443	20009.07	-0.44287	0.064519	-6.86415	6.69E-12	1.15E-10
CNAG_06446	2915.909	0.782239	0.067683	11.55744	6.77E-31	5.08E-29
CNAG_06447	35537.99	-0.22553	0.080304	-2.80848	0.004978	0.018751
CNAG_06449	93.13515	-0.46238	0.218448	-2.11665	0.03429	0.092053
CNAG_06452	1294.129	0.328427	0.079674	4.122131	3.75E-05	0.000252
CNAG_06453	4001.456	1.035563	0.088039	11.76251	6.09E-32	4.80E-30
CNAG_06454	1286.043	-0.22218	0.079834	-2.783	0.005386	0.019996
CNAG_06459	1112.217	0.603435	0.089296	6.757686	1.40E-11	2.35E-10
CNAG_06460	1757.963	-0.16698	0.074744	-2.23398	0.025484	0.07293
CNAG_07942	1659.858	-0.28073	0.088644	-3.16692	0.001541	0.006882
CNAG_06466	459.9842	-0.24622	0.109259	-2.25358	0.024222	0.069706
CNAG_06468	3881.948	-0.62655	0.069873	-8.96693	3.05E-19	1.02E-17
CNAG_06472	4788.553	-0.53372	0.066817	-7.98789	1.37E-15	3.32E-14
CNAG_06474	5771.033	-0.23696	0.071014	-3.33682	0.000847	0.00411
CNAG_06475	8269.977	-0.17443	0.079062	-2.2062	0.02737	0.077447
CNAG_13132	151.7017	-0.4423	0.169216	-2.61384	0.008953	0.030929
CNAG_06482	627.2793	0.453139	0.102644	4.414646	1.01E-05	7.64E-05
CNAG_06484	121.7986	1.058184	0.189104	5.59578	2.20E-08	2.44E-07
CNAG_06486	2505.615	-0.24431	0.074643	-3.27312	0.001064	0.005013
CNAG_07943	141.5177	-1.76491	0.186399	-9.46845	2.84E-21	1.15E-19
CNAG_06491	159.2812	-0.6016	0.164929	-3.64765	0.000265	0.001481
CNAG_06493	1577.369	0.612585	0.108976	5.621271	1.90E-08	2.12E-07
CNAG_06494	1162.252	0.393532	0.088445	4.449455	8.61E-06	6.57E-05
CNAG_06497	812.6171	0.413793	0.096451	4.290208	1.79E-05	0.000129
CNAG_06498	922.0555	0.68738	0.099522	6.906828	4.96E-12	8.67E-11
CNAG_06499	1107.691	0.500012	0.080507	6.21083	5.27E-10	7.24E-09
CNAG_06500	745.5015	0.311238	0.109503	2.842274	0.004479	0.017186
CNAG_06505	1072.706	0.178463	0.082773	2.156062	0.031079	0.085371
CNAG_06506	660.6532	0.184285	0.092511	1.992031	0.046368	0.117256
CNAG_06512	512.5173	-0.49446	0.10919	-4.52847	5.94E-06	4.63E-05
CNAG_13141	168.2074	-0.55846	0.184604	-3.02516	0.002485	0.010404
CNAG_06517	3284.006	-0.71275	0.092552	-7.70112	1.35E-14	3.01E-13
CNAG_13142	439.5184	0.613769	0.127062	4.830466	1.36E-06	1.17E-05
CNAG_07043	360.4921	0.511198	0.12408	4.119891	3.79E-05	0.000254
CNAG_05333	11.14682	2.583006	0.676941	3.815703	0.000136	0.000808
CNAG_05336	1067.761	0.36054	0.089921	4.009534	6.08E-05	0.000395
CNAG_05337	1002.436	0.181102	0.085112	2.127811	0.033353	0.090033
CNAG_05340	215.6441	-1.39521	0.168698	-8.27046	1.33E-16	3.49E-15
CNAG_05341	212.8876	0.423002	0.167801	2.520857	0.011707	0.038634
CNAG_05343	1331.89	-0.29826	0.096327	-3.09631	0.001959	0.008472

CNAG_05344	2576.945	0.238342	0.067553	3.528211	0.000418	0.002224
CNAG_07032	4062.051	0.212741	0.083001	2.563113	0.010374	0.03485
CNAG_05349	5078.27	0.214195	0.069349	3.088652	0.002011	0.008645
CNAG_13146	55.8919	0.556846	0.26951	2.066146	0.038815	0.101682
CNAG_05350	2044.732	0.477868	0.08478	5.636589	1.73E-08	1.96E-07
CNAG_05351	7594.852	-0.19347	0.067385	-2.87117	0.00409	0.015981
CNAG_05352	1259.958	-0.30925	0.078922	-3.91844	8.91E-05	0.000554
CNAG_05356	55.71849	0.523739	0.261273	2.004561	0.04501	0.114715
CNAG_05357	17.93195	1.164725	0.481625	2.418322	0.015592	0.048673
CNAG_13147	136.3623	0.635044	0.203876	3.114856	0.00184	0.008024
CNAG_05360	1963.874	-0.54287	0.07213	-7.52628	5.22E-14	1.10E-12
CNAG_08021	109.9231	0.389149	0.195254	1.993042	0.046257	0.117052
CNAG_05363	2563.725	-0.19403	0.076156	-2.54786	0.010839	0.036209
CNAG_05365	1952.526	-0.24913	0.08055	-3.09282	0.001983	0.008553
CNAG_05370	567.1978	-0.32861	0.09916	-3.31397	0.00092	0.004418
CNAG_05371	1883.429	-0.46383	0.080222	-5.78187	7.39E-09	8.80E-08
CNAG_05375	25727.86	0.17379	0.059879	2.90235	0.003704	0.014709
CNAG_05379	1391.16	-0.16177	0.077767	-2.08021	0.037507	0.098785
CNAG_05381	220.8777	1.512279	0.384751	3.930535	8.48E-05	0.000533
CNAG_07874	35.36483	0.829767	0.334255	2.482439	0.013049	0.042209
CNAG_05383	921.7177	1.116757	0.111887	9.981116	1.84E-23	9.11E-22
CNAG_05386	638.9377	-0.24493	0.097563	-2.51046	0.012057	0.039486
CNAG_13158	42.29634	1.812589	0.340558	5.322408	1.02E-07	1.03E-06
CNAG_05388	1396.068	0.67952	0.085948	7.906166	2.65E-15	6.28E-14
CNAG_05392	175.9939	0.425172	0.177437	2.396187	0.016567	0.05103
CNAG_05394	615.3845	0.21603	0.099938	2.161639	0.030646	0.084509
CNAG_05401	1103.304	-0.70482	0.277702	-2.53806	0.011147	0.037049
CNAG_05402	2680.022	-0.4376	0.093428	-4.68386	2.82E-06	2.31E-05
CNAG_05410	745.9114	1.02892	0.102289	10.05893	8.39E-24	4.20E-22
CNAG_05411	1266.516	0.475969	0.080376	5.921745	3.19E-09	3.99E-08
CNAG_05413	707.938	-0.20351	0.099771	-2.03973	0.041377	0.107457
CNAG_05415	1936.173	-0.61226	0.074113	-8.26118	1.44E-16	3.75E-15
CNAG_13165	24.27277	-0.87575	0.428333	-2.04456	0.040898	0.10639
CNAG_05422	1199.226	0.283288	0.091262	3.104121	0.001908	0.008283
CNAG_08024	358.329	-0.29779	0.142291	-2.09283	0.036364	0.096427
CNAG_05430	776.3244	0.593954	0.097062	6.119323	9.40E-10	1.25E-08
CNAG_05431	1385.483	0.648673	0.080219	8.086325	6.15E-16	1.51E-14
CNAG_05434	3459.741	0.24438	0.064651	3.779973	0.000157	0.00092
CNAG_05436	124.1802	-0.66485	0.185348	-3.58707	0.000334	0.001817
CNAG_05437	17511.93	-0.27975	0.067445	-4.14779	3.36E-05	0.000227
CNAG_05438	649.5541	-0.65222	0.095637	-6.81977	9.12E-12	1.56E-10
CNAG_05439	761.807	0.405476	0.094103	4.308878	1.64E-05	0.000119
CNAG_05441	319.6465	-0.37692	0.121283	-3.10778	0.001885	0.008191
CNAG_05442	1675.287	-0.17835	0.077826	-2.29159	0.021929	0.064226
CNAG_05453	169.6498	-0.51767	0.160954	-3.21623	0.001299	0.005956
CNAG_05457	4309.838	0.545266	0.073483	7.420284	1.17E-13	2.43E-12
CNAG_05459	4417.873	0.418367	0.071246	5.872123	4.30E-09	5.31E-08
CNAG_05460	308.406	-0.83131	0.126648	-6.56392	5.24E-11	8.12E-10

CNAG_05461	56.86511	-0.97255	0.268767	-3.61855	0.000296	0.001629
CNAG_05462	10767.52	0.331674	0.068967	4.809162	1.52E-06	1.29E-05
CNAG_05463	1709.763	-0.25113	0.075713	-3.31683	0.00091	0.004378
CNAG_05465	38844.54	-0.16644	0.077593	-2.14504	0.031949	0.08718
CNAG_05470	694.3285	-0.21866	0.098374	-2.22274	0.026233	0.074608
CNAG_13180	21.01911	-1.25142	0.446372	-2.80353	0.005055	0.018974
CNAG_07878	3112.887	-0.88052	0.099631	-8.83773	9.77E-19	3.07E-17
CNAG_05473	619.7838	-0.54174	0.096872	-5.59227	2.24E-08	2.49E-07
CNAG_13181	92.25756	0.452651	0.204422	2.214293	0.026809	0.07605
CNAG_05475	2779.07	-0.31814	0.068835	-4.6218	3.80E-06	3.07E-05
CNAG_05478	1583.486	-0.21135	0.077988	-2.70997	0.006729	0.024289
CNAG_05479	634.3322	-0.64963	0.10274	-6.32307	2.56E-10	3.70E-09
CNAG_05480	7277.191	-0.21943	0.063311	-3.46587	0.000529	0.002744
CNAG_13183	17.61844	-1.26769	0.491751	-2.57791	0.00994	0.033683
CNAG_05485	280.7604	-0.64257	0.150386	-4.2728	1.93E-05	0.000137
CNAG_13185	108.9366	-0.50988	0.198584	-2.56755	0.010242	0.034482
CNAG_05486	2730.489	-0.27702	0.088212	-3.14043	0.001687	0.007451
CNAG_08026	220.4027	-0.33305	0.144521	-2.30451	0.021194	0.06258
CNAG_05497	16844.42	0.300575	0.059008	5.093797	3.51E-07	3.30E-06
CNAG_05504	2557.718	0.302967	0.069807	4.340044	1.42E-05	0.000105
CNAG_07883	1118.136	-0.1973	0.092134	-2.14149	0.032235	0.087805
CNAG_05509	2173.969	-0.1806	0.079455	-2.27296	0.023029	0.066809
CNAG_05516	1043.768	-0.19649	0.083545	-2.3519	0.018678	0.056279
CNAG_05521	2271.968	0.325383	0.078819	4.12825	3.66E-05	0.000245
CNAG_05522	5060.169	0.252632	0.073843	3.421207	0.000623	0.003165
CNAG_05523	80.12682	-0.85703	0.230858	-3.71237	0.000205	0.00117
CNAG_05524	1242.942	0.219596	0.083258	2.637532	0.008351	0.029213
CNAG_05525	46035.7	-0.16517	0.079618	-2.0745	0.038033	0.100036
CNAG_05528	33.77831	0.752629	0.340607	2.209673	0.027128	0.076844
CNAG_05529	847.5263	-0.33718	0.093735	-3.59713	0.000322	0.001753
CNAG_05531	1471.731	0.320059	0.087192	3.670741	0.000242	0.001364
CNAG_05532	165.2444	-0.42935	0.164308	-2.61307	0.008973	0.030986
CNAG_05533	584.3482	0.633861	0.099839	6.348848	2.17E-10	3.16E-09
CNAG_05534	521.6441	-0.37395	0.109018	-3.43019	0.000603	0.003081
CNAG_05535	1758.351	-0.48176	0.089189	-5.40156	6.61E-08	6.88E-07
CNAG_05536	255.5293	0.532179	0.142091	3.745347	0.00018	0.00104
CNAG_05537	993.151	-0.34243	0.084469	-4.0539	5.04E-05	0.000331
CNAG_05538	1634.368	-0.15862	0.077191	-2.05494	0.039884	0.1041
CNAG_05540	8024.021	0.141114	0.064557	2.1859	0.028823	0.080708
CNAG_13191	19.74802	-1.13644	0.450207	-2.52426	0.011594	0.038323
CNAG_05544	357.4832	0.37602	0.124793	3.013162	0.002585	0.010755
CNAG_05547	293.4871	0.919138	0.133816	6.868697	6.48E-12	1.12E-10
CNAG_05548	162.9897	0.863386	0.164238	5.256915	1.46E-07	1.45E-06
CNAG_05551	790.1423	-0.23794	0.094658	-2.51364	0.011949	0.039216
CNAG_05552	5925.468	-0.20374	0.077339	-2.63434	0.00843	0.02941
CNAG_05553	584.9947	0.341089	0.103353	3.300241	0.000966	0.004611
CNAG_05555	54969.4	-0.20956	0.074052	-2.82992	0.004656	0.01775
CNAG_05556	16303.97	-0.20065	0.073735	-2.72124	0.006504	0.023558

CNAG_05559	2263.718	0.251428	0.074166	3.390054	0.000699	0.003493
CNAG_05566	515.3151	0.338061	0.104296	3.241369	0.00119	0.005513
CNAG_05567	849.2166	0.193933	0.086841	2.233185	0.025537	0.073008
CNAG_05569	694.6259	0.570822	0.092003	6.204382	5.49E-10	7.52E-09
CNAG_05571	3167.92	-0.60058	0.078788	-7.62273	2.48E-14	5.40E-13
CNAG_05573	1429.28	1.220881	0.085311	14.3109	1.87E-46	3.40E-44
CNAG_05575	1373.762	0.641629	0.078364	8.187778	2.66E-16	6.81E-15
CNAG_05578	1889.236	0.210962	0.090532	2.33025	0.019793	0.059091
CNAG_05580	48.78851	1.443774	0.327518	4.408232	1.04E-05	7.84E-05
CNAG_05584	1223.909	-0.2201	0.085175	-2.58407	0.009764	0.033189
CNAG_05586	805.335	0.500079	0.088719	5.636636	1.73E-08	1.96E-07
CNAG_05590	1875.431	0.314941	0.09385	3.355787	0.000791	0.00387
CNAG_05591	645.3286	0.290146	0.101697	2.853035	0.00433	0.016702
CNAG_05592	4025.959	-0.81144	0.066565	-12.1902	3.50E-34	3.25E-32
CNAG_05595	1247.934	0.285436	0.081756	3.491309	0.000481	0.002521
CNAG_05597	894.0709	-0.24045	0.089719	-2.68001	0.007362	0.026198
CNAG_05598	1286.545	0.376294	0.076655	4.908902	9.16E-07	7.99E-06
CNAG_05599	372.3624	0.829327	0.123977	6.689352	2.24E-11	3.69E-10
CNAG_05600	1764.932	-0.30434	0.077761	-3.91384	9.08E-05	0.000564
CNAG_05601	1121.376	-0.26229	0.085231	-3.07743	0.002088	0.008923
CNAG_05603	1104.435	-0.57181	0.088901	-6.43206	1.26E-10	1.87E-09
CNAG_05605	706.8289	0.280004	0.099829	2.804843	0.005034	0.018924
CNAG_05606	802.56	-0.22691	0.112004	-2.02594	0.042771	0.110165
CNAG_05607	2450.173	0.333019	0.079539	4.186869	2.83E-05	0.000194
CNAG_05608	452.6187	0.404444	0.126816	3.189212	0.001427	0.006458
CNAG_07888	25676.14	0.381057	0.075094	5.074382	3.89E-07	3.62E-06
CNAG_07889	1618.008	-0.41775	0.075949	-5.50041	3.79E-08	4.08E-07
CNAG_05614	1848.436	0.418497	0.083536	5.0098	5.45E-07	4.98E-06
CNAG_05615	5670.37	-0.22899	0.075198	-3.0452	0.002325	0.009782
CNAG_05616	1935.621	0.305044	0.093271	3.270514	0.001074	0.005053
CNAG_05617	1258.271	-0.29812	0.089294	-3.33867	0.000842	0.004088
CNAG_05618	1994.73	-0.34972	0.074722	-4.68026	2.87E-06	2.35E-05
CNAG_05619	1203.288	-0.39233	0.079719	-4.92143	8.59E-07	7.53E-06
CNAG_05620	1123.636	0.44498	0.083485	5.330075	9.82E-08	9.94E-07
CNAG_05621	1187.869	-0.41324	0.08208	-5.03457	4.79E-07	4.42E-06
CNAG_05622	772.0948	-0.26288	0.120141	-2.18809	0.028663	0.080376
CNAG_05624	889.3339	-0.25518	0.094058	-2.713	0.006668	0.02409
CNAG_05626	1729.89	1.516959	0.075854	19.99849	5.68E-89	7.38E-86
CNAG_05627	1143.623	-0.56662	0.079431	-7.13351	9.78E-13	1.84E-11
CNAG_05628	819.237	0.311831	0.08918	3.496638	0.000471	0.002474
CNAG_05631	11536.66	0.187489	0.060316	3.108473	0.001881	0.008176
CNAG_05632	257.513	0.583712	0.136458	4.277599	1.89E-05	0.000135
CNAG_05633	4242.542	0.340525	0.072833	4.675443	2.93E-06	2.40E-05
CNAG_05637	1043.625	0.298556	0.091265	3.271315	0.00107	0.005042
CNAG_05639	1509.545	0.743432	0.084888	8.757849	1.99E-18	6.11E-17
CNAG_05640	1292.576	-0.17476	0.079408	-2.20079	0.027751	0.078353
CNAG_05642	1308.364	-0.37359	0.103761	-3.60046	0.000318	0.001734
CNAG_05643	939.0149	0.481986	0.102463	4.703988	2.55E-06	2.11E-05

CNAG_05644	358.3596	-0.63079	0.119606	-5.27392	1.34E-07	1.32E-06
CNAG_05645	1521.354	0.325999	0.082487	3.952113	7.75E-05	0.000492
CNAG_05651	495.9795	-0.55453	0.108913	-5.09144	3.55E-07	3.33E-06
CNAG_05652	1516.392	0.491594	0.078337	6.275382	3.49E-10	4.91E-09
CNAG_05653	1365.684	0.93224	0.107073	8.706618	3.13E-18	9.36E-17
CNAG_05654	471.8605	0.919785	0.118458	7.764624	8.19E-15	1.86E-13
CNAG_05656	162.635	0.618778	0.162831	3.800127	0.000145	0.000856
CNAG_05657	907.3743	-1.36806	0.109962	-12.4412	1.56E-35	1.58E-33
CNAG_05660	2160.632	-0.23905	0.072315	-3.30573	0.000947	0.004535
CNAG_13203	154.2388	0.545554	0.174526	3.125921	0.001772	0.007767
CNAG_05662	4979.296	2.120062	0.247142	8.578314	9.63E-18	2.72E-16
CNAG_05663	3068.972	-0.22473	0.110556	-2.03275	0.042077	0.108841
CNAG_05665	2115.273	0.505974	0.076097	6.649087	2.95E-11	4.72E-10
CNAG_05667	535.5497	-0.58103	0.102202	-5.68517	1.31E-08	1.51E-07
CNAG_13206	289.9507	0.800981	0.14092	5.683929	1.32E-08	1.52E-07

Table S7. Data sheets of the RNA-seq data for L-DOPA treatment.

	baseMean	log2FoldCh	lfcSE	stat	pvalue	padj
CNAG_07303	5.215809	-2.41691	1.117363	-2.16305	0.030538	0.063839
CNAG_00002	777.2105	-0.56059	0.209632	-2.67417	0.007491	0.019153
CNAG_12001	44.45174	-1.27195	0.403439	-3.15276	0.001617	0.005066
CNAG_00007	3549.556	-0.48982	0.129262	-3.78937	0.000151	0.000628
CNAG_00009	838.3747	-0.35288	0.142968	-2.46826	0.013577	0.031938
CNAG_00011	1116.719	0.603541	0.197712	3.052629	0.002268	0.006831
CNAG_00014	409.6928	0.731429	0.204096	3.583751	0.000339	0.001285
CNAG_00015	274.6089	0.961797	0.186631	5.153461	2.56E-07	1.99E-06
CNAG_00016	2990.749	-0.31834	0.125818	-2.53017	0.011401	0.027527
CNAG_00017	492.0273	1.1063	0.259079	4.270125	1.95E-05	0.000101
CNAG_12002	34.00088	2.14414	0.433779	4.942936	7.70E-07	5.47E-06
CNAG_00018	1707.721	-0.69918	0.149693	-4.67075	3.00E-06	1.90E-05
CNAG_00020	1417.702	0.511232	0.198609	2.574065	0.010051	0.024711
CNAG_12003	48.53692	1.33116	0.326525	4.076747	4.57E-05	0.000216
CNAG_00021	834.3204	-0.98412	0.145397	-6.76855	1.30E-11	2.24E-10
CNAG_00022	1163.718	-1.30674	0.237988	-5.49078	4.00E-08	3.76E-07
CNAG_00023	164.8357	0.762866	0.180711	4.22147	2.43E-05	0.000123
CNAG_00025	1364.991	0.499258	0.139323	3.583456	0.000339	0.001285
CNAG_00026	6056.017	-0.62891	0.173395	-3.62703	0.000287	0.001114
CNAG_00030	238.4392	-1.06034	0.207624	-5.10702	3.27E-07	2.50E-06
CNAG_00031	668.2287	-0.29402	0.125038	-2.35141	0.018702	0.042027
CNAG_00034	37138.83	-0.88395	0.192721	-4.5867	4.50E-06	2.74E-05
CNAG_00036	837.4774	1.7747	0.229261	7.740969	9.87E-15	2.60E-13
CNAG_07307	51.56041	0.906035	0.365949	2.475853	0.013292	0.03134
CNAG_00038	1309.458	1.1296	0.126323	8.942136	3.82E-19	1.63E-17
CNAG_12005	24.19063	2.002427	0.488325	4.100601	4.12E-05	0.000197
CNAG_00039	1175.732	0.474415	0.217846	2.177754	0.029424	0.061821
CNAG_00040	3242.292	-0.99312	0.181439	-5.4736	4.41E-08	4.09E-07
CNAG_07308	278.461	2.091331	0.310244	6.740923	1.57E-11	2.65E-10
CNAG_12006	191.1548	1.25509	0.23486	5.343998	9.09E-08	7.90E-07
CNAG_00045	635.6095	1.771358	0.15073	11.75184	6.91E-32	7.42E-30
CNAG_00046	7114.898	0.376785	0.189046	1.99309	0.046252	0.090992
CNAG_00047	576.7314	0.521276	0.169322	3.078605	0.00208	0.006336
CNAG_00048	97.61766	0.680045	0.245766	2.767047	0.005657	0.015134
CNAG_12007	3.074766	5.024041	1.715337	2.928894	0.003402	0.009678
CNAG_00052	1228.553	1.577625	0.286861	5.499618	3.81E-08	3.62E-07
CNAG_00053	321.0993	-0.67494	0.168467	-4.00636	6.17E-05	0.000283
CNAG_00054	646.8075	2.963946	0.216598	13.68409	1.26E-42	2.39E-40
CNAG_00055	1294.639	-0.5708	0.165501	-3.44894	0.000563	0.002012
CNAG_00056	1227.067	0.783793	0.184364	4.251328	2.13E-05	0.000109
CNAG_00057	10384.94	1.297369	0.203895	6.362942	1.98E-10	2.81E-09
CNAG_00059	370.2805	-1.07682	0.313899	-3.43046	0.000603	0.002136
CNAG_00061	25478.83	-0.37916	0.129509	-2.92768	0.003415	0.009712
CNAG_00062	5608.088	0.66064	0.125676	5.256701	1.47E-07	1.21E-06
CNAG_00063	1249.139	-0.98638	0.190524	-5.17717	2.25E-07	1.78E-06
CNAG_12009	40.04248	1.049318	0.372366	2.817973	0.004833	0.013151

CNAG_12010	4.554955	-2.60329	1.083202	-2.40333	0.016246	0.037301
CNAG_12011	10.06063	-1.40166	0.690794	-2.02905	0.042453	0.084544
CNAG_00068	831.8577	-1.39997	0.264058	-5.30175	1.15E-07	9.72E-07
CNAG_00070	1276.14	0.846871	0.145687	5.812936	6.14E-09	6.65E-08
CNAG_00074	4812.398	0.482252	0.134519	3.585009	0.000337	0.001281
CNAG_00076	1795.05	0.634464	0.128404	4.94114	7.77E-07	5.52E-06
CNAG_07947	100.7648	1.144475	0.242348	4.722448	2.33E-06	1.51E-05
CNAG_00077	5001.771	0.654687	0.129326	5.062288	4.14E-07	3.10E-06
CNAG_00078	6241.714	-0.87753	0.158769	-5.52708	3.26E-08	3.16E-07
CNAG_00079	635.3978	-0.66939	0.221578	-3.02101	0.002519	0.007492
CNAG_00080	65.93933	0.786324	0.304342	2.583683	0.009775	0.024137
CNAG_00081	3148.853	-0.31255	0.132647	-2.35625	0.01846	0.041601
CNAG_00082	1441.44	0.326891	0.141434	2.311258	0.020819	0.046079
CNAG_00084	3609.458	-0.37701	0.130199	-2.89567	0.003784	0.0106
CNAG_00085	758.4885	-0.4327	0.19308	-2.24102	0.025025	0.05385
CNAG_00086	1689.186	-0.89102	0.133265	-6.68613	2.29E-11	3.73E-10
CNAG_00087	1704.526	0.365054	0.147416	2.476358	0.013273	0.031305
CNAG_00088	4773.617	0.401168	0.127545	3.145311	0.001659	0.005184
CNAG_00089	1337.913	-0.38356	0.121135	-3.16638	0.001543	0.004853
CNAG_00090	1568.459	-0.63676	0.17753	-3.58677	0.000335	0.001274
CNAG_00091	14286.99	3.104183	0.202213	15.35107	3.48E-53	9.67E-51
CNAG_00093	5461.285	2.049661	0.185212	11.06655	1.82E-28	1.49E-26
CNAG_00094	244.4781	0.70399	0.174012	4.045637	5.22E-05	0.000243
CNAG_00097	459.3118	0.750498	0.163349	4.594436	4.34E-06	2.66E-05
CNAG_12015	8.8246	2.139166	0.874208	2.446976	0.014406	0.033688
CNAG_00098	1259.797	1.294253	0.172457	7.504799	6.15E-14	1.47E-12
CNAG_00099	1806.048	-0.62265	0.157527	-3.95266	7.73E-05	0.000347
CNAG_00101	1759.549	-0.91647	0.230344	-3.97871	6.93E-05	0.000315
CNAG_07309	2292.427	0.392738	0.181844	2.159747	0.030792	0.064253
CNAG_07310	1562.447	-0.85472	0.228065	-3.74769	0.000178	0.000733
CNAG_00104	6612.922	-0.44688	0.128769	-3.47037	0.00052	0.001876
CNAG_00107	2632.773	1.761914	0.179591	9.810721	1.01E-22	6.24E-21
CNAG_00108	4585.241	-0.40571	0.14858	-2.73061	0.006322	0.016644
CNAG_00109	1673.456	-0.56566	0.214412	-2.63822	0.008334	0.020997
CNAG_00110	929.4177	-1.02045	0.250304	-4.07684	4.57E-05	0.000216
CNAG_00111	3121.308	-0.37624	0.178588	-2.10673	0.035141	0.072022
CNAG_07311	513.2772	-0.86627	0.152065	-5.69671	1.22E-08	1.26E-07
CNAG_00116	36529.47	-1.04897	0.258906	-4.05156	5.09E-05	0.000238
CNAG_00117	3549.399	-0.35682	0.158814	-2.24677	0.024655	0.053235
CNAG_00120	753.3568	-0.47347	0.185105	-2.55786	0.010532	0.025746
CNAG_00123	549.7308	1.201895	0.218959	5.489143	4.04E-08	3.79E-07
CNAG_00124	987.6208	0.322449	0.156317	2.062792	0.039132	0.07878
CNAG_00125	188.4452	1.280127	0.248106	5.159595	2.47E-07	1.93E-06
CNAG_00126	2209.449	-0.98138	0.207588	-4.72754	2.27E-06	1.48E-05
CNAG_00127	601.2443	-0.70177	0.269544	-2.60356	0.009226	0.022931
CNAG_12018	293.7365	-0.68443	0.208831	-3.27743	0.001048	0.00345
CNAG_12019	198.6296	1.482378	0.315942	4.691933	2.71E-06	1.73E-05

CNAG_00130	12416.55	1.064882	0.186699	5.703732	1.17E-08	1.22E-07
CNAG_00132	189.9648	1.519539	0.247385	6.142399	8.13E-10	1.05E-08
CNAG_00133	709.8663	0.947792	0.187527	5.054177	4.32E-07	3.23E-06
CNAG_00134	813.6532	0.891723	0.257872	3.45801	0.000544	0.001954
CNAG_00135	734.6138	-0.54899	0.17597	-3.11982	0.00181	0.005571
CNAG_00136	7211.854	0.388748	0.146921	2.645965	0.008146	0.020601
CNAG_00137	3113.142	1.105503	0.148136	7.462772	8.47E-14	1.97E-12
CNAG_00138	2557.596	-0.37254	0.113496	-3.28244	0.001029	0.003402
CNAG_00139	4942.07	1.207475	0.13957	8.651413	5.09E-18	1.88E-16
CNAG_00141	1725.569	1.214476	0.1725	7.040427	1.92E-12	3.83E-11
CNAG_12022	93.80807	-1.23878	0.335096	-3.69678	0.000218	0.000873
CNAG_00143	4362.839	-0.8286	0.154357	-5.36811	7.96E-08	6.98E-07
CNAG_00144	558.9319	0.847815	0.13114	6.464961	1.01E-10	1.50E-09
CNAG_00145	1418.768	-0.27406	0.139705	-1.96169	0.049799	0.096915
CNAG_00148	4541.728	0.59969	0.176997	3.388132	0.000704	0.002434
CNAG_00149	7923.333	-1.17129	0.270483	-4.33036	1.49E-05	7.96E-05
CNAG_00150	2958.692	0.33739	0.14616	2.308363	0.020979	0.046408
CNAG_00151	2975.435	-0.59753	0.128791	-4.63953	3.49E-06	2.18E-05
CNAG_00152	851.0682	-0.65053	0.136832	-4.75425	1.99E-06	1.30E-05
CNAG_00154	1713.772	1.281942	0.153969	8.325978	8.36E-17	2.80E-15
CNAG_12025	16.77819	1.80778	0.635173	2.846121	0.004426	0.012189
CNAG_00156	1665.291	0.821659	0.171408	4.793582	1.64E-06	1.09E-05
CNAG_00159	323.5826	-0.44749	0.207405	-2.15755	0.030963	0.064559
CNAG_00161	425.5586	-0.45306	0.162103	-2.79491	0.005191	0.014045
CNAG_00165	2767.733	-0.64915	0.15371	-4.22319	2.41E-05	0.000122
CNAG_00166	974.1452	-0.44888	0.180402	-2.48819	0.012839	0.030491
CNAG_00167	1037.24	0.522787	0.179942	2.905303	0.003669	0.010335
CNAG_07314	649.9343	0.811257	0.156883	5.171093	2.33E-07	1.83E-06
CNAG_07315	1696.62	0.379184	0.149292	2.539875	0.011089	0.026881
CNAG_00172	707.6524	-1.13431	0.305855	-3.70866	0.000208	0.000839
CNAG_00174	1233.859	0.537232	0.161076	3.335276	0.000852	0.002873
CNAG_00175	875.7931	0.562366	0.208011	2.703539	0.006861	0.017815
CNAG_00176	3652.366	0.412308	0.127799	3.226214	0.001254	0.004057
CNAG_00177	108.1619	0.956787	0.286948	3.334356	0.000855	0.00288
CNAG_00178	1589.974	0.874014	0.170862	5.115311	3.13E-07	2.40E-06
CNAG_12030	27.81252	-2.19372	0.487803	-4.49715	6.89E-06	4.00E-05
CNAG_00181	1129.181	0.628476	0.221718	2.834578	0.004589	0.012577
CNAG_00182	1450.013	0.511795	0.133372	3.837357	0.000124	0.000528
CNAG_00187	1865.022	0.28222	0.124136	2.273479	0.022997	0.050313
CNAG_00189	881.7175	0.494136	0.142938	3.456984	0.000546	0.00196
CNAG_00190	3281.729	1.488178	0.148575	10.01634	1.29E-23	8.21E-22
CNAG_00191	496.8995	-0.31002	0.149115	-2.07909	0.037609	0.076411
CNAG_00193	4120.448	-0.87587	0.203722	-4.29937	1.71E-05	9.06E-05
CNAG_00194	2313.64	-0.33574	0.124561	-2.69534	0.007032	0.0182
CNAG_07317	2171.182	0.536153	0.150522	3.56196	0.000368	0.00138
CNAG_07319	3399.381	1.390531	0.207704	6.694763	2.16E-11	3.54E-10
CNAG_07320	1140.437	-0.28744	0.124476	-2.30923	0.020931	0.046315

CNAG_07322	3392.788	-0.53606	0.138001	-3.88447	0.000103	0.000445
CNAG_07324	1473.221	0.332143	0.157094	2.114301	0.03449	0.070833
CNAG_07326	3061.892	-0.64429	0.180575	-3.56798	0.00036	0.001352
CNAG_07334	647.8098	-0.43076	0.199341	-2.16094	0.0307	0.064112
CNAG_07335	245.9256	-0.61012	0.175878	-3.46901	0.000522	0.001885
CNAG_07030	497.9399	-0.36398	0.161112	-2.25919	0.023871	0.051911
CNAG_07028	5156.24	0.743669	0.15496	4.799089	1.59E-06	1.06E-05
CNAG_07338	951.7108	0.5898	0.152302	3.87258	0.000108	0.000465
CNAG_07339	1666.75	0.386976	0.162224	2.385434	0.017059	0.038873
CNAG_07342	1391.359	0.275253	0.132994	2.069657	0.038484	0.077772
CNAG_00229	410.9604	1.247786	0.187084	6.66967	2.56E-11	4.14E-10
CNAG_00231	577.895	0.520052	0.177711	2.926389	0.003429	0.009746
CNAG_00232	25147.26	-0.74439	0.252548	-2.94752	0.003203	0.009166
CNAG_00236	994.6797	-0.56467	0.145485	-3.8813	0.000104	0.00045
CNAG_00237	9591.266	-1.39178	0.152997	-9.09675	9.31E-20	4.18E-18
CNAG_00238	15158.99	-0.6626	0.137551	-4.81714	1.46E-06	9.75E-06
CNAG_00239	628.0259	0.802601	0.166361	4.824451	1.40E-06	9.43E-06
CNAG_00242	22.87801	0.966224	0.433158	2.23065	0.025704	0.055098
CNAG_00243	431.6726	-0.51517	0.181533	-2.8379	0.004541	0.012469
CNAG_07344	439.4747	1.27287	0.209603	6.07277	1.26E-09	1.57E-08
CNAG_12039	113.9581	-0.55323	0.224917	-2.4597	0.013905	0.032651
CNAG_00250	685.9511	-1.18707	0.174565	-6.80019	1.04E-11	1.83E-10
CNAG_00251	27.47024	2.170126	0.444511	4.882054	1.05E-06	7.23E-06
CNAG_00253	1181.997	0.691804	0.260619	2.654469	0.007943	0.020133
CNAG_00254	1315.897	0.951537	0.241274	3.94381	8.02E-05	0.000359
CNAG_00259	1832.175	-0.54706	0.165686	-3.30176	0.000961	0.003205
CNAG_00260	2757.953	-0.2572	0.117082	-2.19672	0.028041	0.059353
CNAG_00261	11436.57	0.441735	0.152337	2.899732	0.003735	0.010487
CNAG_00263	740.8116	0.372474	0.142133	2.620592	0.008778	0.021934
CNAG_00264	5324.956	0.339499	0.167947	2.021467	0.043231	0.085944
CNAG_00265	1635.862	-0.83652	0.145732	-5.74014	9.46E-09	9.94E-08
CNAG_00268	8108.835	-0.74876	0.133856	-5.59379	2.22E-08	2.21E-07
CNAG_00270	4682.228	-0.75682	0.278354	-2.7189	0.00655	0.017121
CNAG_00273	238.1654	-0.70333	0.237539	-2.96092	0.003067	0.00883
CNAG_00279	5276.313	-0.66306	0.213005	-3.11288	0.001853	0.005695
CNAG_00284	320.6185	0.766744	0.277129	2.76674	0.005662	0.015142
CNAG_00285	1151.644	0.769233	0.251406	3.059726	0.002215	0.006696
CNAG_00288	1486.843	-0.71308	0.232913	-3.06159	0.002202	0.006659
CNAG_00292	4627.535	-0.26956	0.10342	-2.60645	0.009148	0.022767
CNAG_00294	2326.582	-0.52403	0.12307	-4.25795	2.06E-05	0.000106
CNAG_07347	10861.33	1.176981	0.236137	4.984323	6.22E-07	4.51E-06
CNAG_07349	1865.677	-0.72739	0.237192	-3.06665	0.002165	0.006558
CNAG_00301	1590.467	2.079908	0.190198	10.93551	7.80E-28	6.26E-26
CNAG_00305	11356.29	0.262526	0.117006	2.243704	0.024851	0.053544
CNAG_00308	1452.702	0.442324	0.19864	2.226763	0.025963	0.055547
CNAG_00309	2132.22	0.631585	0.118795	5.316583	1.06E-07	9.04E-07
CNAG_00313	1274.025	0.878312	0.201749	4.353477	1.34E-05	7.30E-05

CNAG_00314	728.5662	0.54522	0.131555	4.14442	3.41E-05	0.000167
CNAG_07350	864.5517	1.251464	0.226683	5.520777	3.38E-08	3.26E-07
CNAG_12047	26.21323	1.075715	0.436095	2.466701	0.013636	0.032067
CNAG_00319	3616.281	-0.59726	0.226206	-2.64032	0.008283	0.0209
CNAG_00320	448.795	-0.54287	0.219716	-2.47077	0.013482	0.03175
CNAG_00321	83.07583	0.844062	0.285537	2.956055	0.003116	0.008958
CNAG_00322	825.0981	0.289027	0.135744	2.129213	0.033237	0.068561
CNAG_12048	114.7781	0.701396	0.26227	2.674323	0.007488	0.01915
CNAG_00328	1493.481	0.852181	0.200807	4.243775	2.20E-05	0.000112
CNAG_12049	7.944476	1.739555	0.80779	2.153474	0.031281	0.065137
CNAG_00329	2764.446	-1.05642	0.286507	-3.68723	0.000227	0.000903
CNAG_00330	1027.014	-0.8926	0.154205	-5.78843	7.10E-09	7.63E-08
CNAG_00332	1854.704	0.424935	0.201023	2.113865	0.034527	0.070891
CNAG_00334	62424.55	-0.79585	0.114057	-6.97769	3.00E-12	5.66E-11
CNAG_00337	2484.078	1.342745	0.203699	6.591815	4.34E-11	6.74E-10
CNAG_07352	6135.99	-0.77036	0.254714	-3.0244	0.002491	0.007417
CNAG_00345	2395.399	-0.30179	0.151864	-1.98723	0.046897	0.092102
CNAG_00347	1395.836	0.689529	0.323611	2.130732	0.033111	0.068356
CNAG_00348	289.4319	0.856706	0.17021	5.03323	4.82E-07	3.57E-06
CNAG_07356	2632.784	-1.45821	0.211173	-6.90527	5.01E-12	9.22E-11
CNAG_00354	4947.166	-0.32932	0.125734	-2.61918	0.008814	0.022011
CNAG_00357	441.0244	0.699336	0.245064	2.853686	0.004322	0.011931
CNAG_12051	68.1	0.725297	0.267371	2.712706	0.006674	0.017404
CNAG_00361	3846.398	0.265099	0.110954	2.389274	0.016882	0.038524
CNAG_00363	5273.139	0.827581	0.186402	4.439774	9.01E-06	5.07E-05
CNAG_00364	1312.879	-0.5447	0.151184	-3.60289	0.000315	0.001205
CNAG_00365	2011.985	-0.49688	0.127696	-3.89112	9.98E-05	0.000435
CNAG_00366	53.95228	1.073	0.293202	3.65959	0.000253	0.000993
CNAG_00370	28965.89	-0.90037	0.265248	-3.39445	0.000688	0.00239
CNAG_00374	1063.474	0.750069	0.200543	3.740193	0.000184	0.000752
CNAG_00377	4131.759	-0.73579	0.221158	-3.32696	0.000878	0.002949
CNAG_00384	524.8199	0.459938	0.169933	2.706595	0.006798	0.017675
CNAG_00385	4026.385	0.517489	0.162268	3.189096	0.001427	0.004529
CNAG_00386	5977.145	-0.55275	0.147521	-3.74689	0.000179	0.000735
CNAG_00387	570.2841	-0.40728	0.186684	-2.18166	0.029135	0.061318
CNAG_00389	1368.954	-0.46817	0.132724	-3.52742	0.00042	0.001549
CNAG_07951	834.2274	-0.61716	0.137359	-4.49307	7.02E-06	4.07E-05
CNAG_00392	334.8071	-0.38986	0.196477	-1.98424	0.047229	0.092616
CNAG_00394	509.638	-0.41283	0.209916	-1.96664	0.049224	0.095914
CNAG_00399	1908.943	1.560331	0.115634	13.49368	1.70E-41	3.15E-39
CNAG_00400	2473.152	0.367127	0.142307	2.579818	0.009885	0.024379
CNAG_00401	1139.729	0.646883	0.167891	3.852995	0.000117	0.0005
CNAG_00403	844.4651	-0.53771	0.200823	-2.67755	0.007416	0.019004
CNAG_00404	238.1282	0.368672	0.178699	2.063087	0.039104	0.078744
CNAG_00405	713.3259	0.556939	0.162452	3.428324	0.000607	0.002146
CNAG_00406	375.9941	-1.1434	0.150807	-7.58188	3.41E-14	8.43E-13
CNAG_00407	8382.057	0.947475	0.218768	4.330957	1.48E-05	7.94E-05

CNAG_00409	1859.441	0.621118	0.18244	3.404511	0.000663	0.002318
CNAG_00412	38.71099	0.705333	0.318516	2.214434	0.026799	0.05709
CNAG_00413	3401.675	-0.37355	0.137431	-2.71807	0.006566	0.017158
CNAG_00414	480.0561	-0.7562	0.168098	-4.49858	6.84E-06	3.98E-05
CNAG_00415	573.3371	-0.54172	0.172982	-3.13165	0.001738	0.005387
CNAG_00417	30819.58	-0.57406	0.191532	-2.99723	0.002724	0.008018
CNAG_00418	17170.23	-0.86134	0.10676	-8.06801	7.15E-16	2.18E-14
CNAG_12057	141.53	0.831665	0.227835	3.650292	0.000262	0.001027
CNAG_00419	107.2509	1.342832	0.245581	5.467991	4.55E-08	4.21E-07
CNAG_00423	519.2387	-1.06393	0.238946	-4.45257	8.48E-06	4.81E-05
CNAG_00426	1410.388	0.431255	0.180449	2.389893	0.016853	0.038471
CNAG_00428	736.1313	-0.5367	0.204686	-2.62208	0.00874	0.021866
CNAG_00430	148.769	-1.23285	0.217951	-5.65654	1.54E-08	1.57E-07
CNAG_07359	3215.254	1.010364	0.294636	3.429187	0.000605	0.002141
CNAG_00434	1279.78	-0.40817	0.195348	-2.08944	0.036669	0.074767
CNAG_00441	15276.6	-0.34816	0.111589	-3.11999	0.001809	0.00557
CNAG_00442	1687.081	-1.4616	0.158789	-9.20466	3.43E-20	1.60E-18
CNAG_00443	195.9744	0.944121	0.221005	4.271948	1.94E-05	0.000101
CNAG_00444	374.1887	-0.58261	0.257596	-2.26171	0.023715	0.0516
CNAG_00449	1555.141	-0.87763	0.124882	-7.02769	2.10E-12	4.14E-11
CNAG_00450	8291.289	-0.33364	0.155834	-2.14098	0.032276	0.066957
CNAG_00451	438.8633	-0.67721	0.211576	-3.20079	0.001371	0.004375
CNAG_00456	10621.23	-1.03292	0.117983	-8.75485	2.04E-18	8.01E-17
CNAG_00457	33987.9	-0.89177	0.20648	-4.31893	1.57E-05	8.36E-05
CNAG_00458	2147.702	-0.27054	0.1129	-2.39624	0.016564	0.037891
CNAG_00460	368.4203	-0.94022	0.187458	-5.01567	5.28E-07	3.89E-06
CNAG_00461	5301.509	0.485483	0.155795	3.116162	0.001832	0.005634
CNAG_00462	3139.424	-1.86503	0.198874	-9.37798	6.72E-21	3.38E-19
CNAG_12060	375.9207	1.193931	0.158996	7.509206	5.95E-14	1.43E-12
CNAG_00465	2815.914	1.508162	0.279552	5.394926	6.86E-08	6.11E-07
CNAG_00466	866.7568	0.493875	0.196124	2.518185	0.011796	0.028309
CNAG_00469	1897.183	0.418876	0.12819	3.267614	0.001085	0.003552
CNAG_00474	3035.954	5.202829	0.178005	29.22863	8.39E-188	6.67E-184
CNAG_07361	1902.057	-0.79692	0.272947	-2.9197	0.003504	0.009929
CNAG_07362	8625.232	-1.03553	0.200278	-5.17047	2.34E-07	1.84E-06
CNAG_07363	12011.51	-0.37257	0.156416	-2.38191	0.017223	0.039191
CNAG_12061	6.308757	2.665066	0.902421	2.95324	0.003145	0.009024
CNAG_00480	1943.8	1.508771	0.218651	6.900365	5.19E-12	9.52E-11
CNAG_00485	2068.754	2.223513	0.198207	11.21812	3.32E-29	2.90E-27
CNAG_00486	1839.694	-0.63146	0.239495	-2.63664	0.008373	0.021082
CNAG_00487	115.5791	1.129111	0.371907	3.036007	0.002397	0.007167
CNAG_00488	334.4608	0.478804	0.229522	2.086093	0.03697	0.075296
CNAG_00492	64.54477	1.397992	0.295164	4.736325	2.18E-06	1.42E-05
CNAG_00493	757.0207	0.606549	0.307596	1.971901	0.048621	0.094971
CNAG_07364	835.2002	0.537956	0.1241	4.334857	1.46E-05	7.85E-05
CNAG_00497	4198.742	1.585721	0.175049	9.058741	1.32E-19	5.79E-18
CNAG_00498	739.1602	0.668485	0.163563	4.087024	4.37E-05	0.000208

CNAG_00499	854.8278	-0.47743	0.210409	-2.26907	0.023264	0.050813
CNAG_00504	1346.898	-0.43231	0.163926	-2.6372	0.008359	0.021053
CNAG_00506	762.0238	-0.60657	0.144302	-4.2035	2.63E-05	0.000132
CNAG_00508	1214.985	0.484478	0.199636	2.426809	0.015232	0.035339
CNAG_00509	8488.024	-0.65097	0.179143	-3.63379	0.000279	0.001088
CNAG_00510	715.1129	-0.63505	0.168557	-3.76759	0.000165	0.000683
CNAG_00511	105.2976	0.486928	0.223209	2.181491	0.029147	0.061319
CNAG_00512	2857.96	-0.49842	0.109464	-4.5533	5.28E-06	3.16E-05
CNAG_00513	2453.897	-0.80181	0.141103	-5.6824	1.33E-08	1.36E-07
CNAG_00515	331.2852	1.459113	0.177151	8.236537	1.77E-16	5.77E-15
CNAG_00516	229.6356	-0.42307	0.189599	-2.2314	0.025655	0.05502
CNAG_07365	1196.379	0.284344	0.140689	2.021085	0.043271	0.086001
CNAG_00519	2621.611	-0.57179	0.146447	-3.90442	9.45E-05	0.000415
CNAG_07953	964.5317	-0.88131	0.22327	-3.9473	7.90E-05	0.000354
CNAG_00521	2038.79	1.134141	0.159963	7.090044	1.34E-12	2.73E-11
CNAG_00522	5695.671	1.641786	0.187698	8.746979	2.19E-18	8.49E-17
CNAG_00524	1012.266	0.532561	0.146915	3.624959	0.000289	0.001121
CNAG_00526	43.72438	0.999037	0.344234	2.9022	0.003706	0.010426
CNAG_00527	3668.856	-0.58866	0.126112	-4.66774	3.05E-06	1.93E-05
CNAG_00528	819.3239	0.471988	0.147739	3.194744	0.0014	0.004454
CNAG_00531	6984.988	0.387301	0.188301	2.056813	0.039704	0.07983
CNAG_12064	41.5078	0.812343	0.37944	2.140901	0.032282	0.066957
CNAG_00532	1082.25	-0.78898	0.161686	-4.87969	1.06E-06	7.31E-06
CNAG_00534	5030.711	-0.7103	0.267734	-2.65301	0.007978	0.020206
CNAG_00535	2350.055	-0.48966	0.186588	-2.6243	0.008683	0.021744
CNAG_00536	2352.343	-1.24491	0.277256	-4.49009	7.12E-06	4.12E-05
CNAG_00537	1413.67	-0.73012	0.275013	-2.65488	0.007934	0.020122
CNAG_00538	1783.586	-0.81843	0.205467	-3.98328	6.80E-05	0.000309
CNAG_00543	3015.819	1.015438	0.209241	4.852957	1.22E-06	8.25E-06
CNAG_00544	235.548	0.571139	0.213864	2.670574	0.007572	0.019322
CNAG_00546	1709.191	1.039473	0.189042	5.498637	3.83E-08	3.63E-07
CNAG_00554	1195.205	0.84635	0.226658	3.734043	0.000188	0.000769
CNAG_00556	3027.28	-0.35113	0.169803	-2.06785	0.038654	0.078055
CNAG_00557	1884.558	-0.60157	0.154331	-3.89788	9.70E-05	0.000424
CNAG_12074	36.52627	-1.13837	0.542557	-2.09815	0.035892	0.073466
CNAG_00559	2094.89	0.81063	0.19693	4.116341	3.85E-05	0.000186
CNAG_00561	3168.076	-0.31759	0.110409	-2.87648	0.004021	0.011173
CNAG_00562	1518.15	-0.39446	0.127164	-3.10195	0.001922	0.005887
CNAG_00563	1170.534	0.687669	0.146411	4.696824	2.64E-06	1.70E-05
CNAG_00564	636.9833	0.566827	0.138831	4.082842	4.45E-05	0.000211
CNAG_00565	10392.76	0.552555	0.12724	4.342613	1.41E-05	7.61E-05
CNAG_00566	1298.05	0.714141	0.233579	3.057381	0.002233	0.006741
CNAG_00568	59.20934	0.653916	0.295952	2.209533	0.027138	0.057734
CNAG_00569	2327.381	0.31534	0.132205	2.385231	0.017068	0.038883
CNAG_00573	1818.162	-1.046	0.197563	-5.2945	1.19E-07	1.00E-06
CNAG_00575	2186.642	2.311931	0.197147	11.72692	9.28E-32	9.45E-30
CNAG_07369	48.68429	0.799991	0.307578	2.600936	0.009297	0.023082

CNAG_00581	14259.77	0.371226	0.171635	2.162878	0.030551	0.063849
CNAG_00582	2000.016	0.519137	0.17396	2.984237	0.002843	0.008308
CNAG_00586	203.2791	1.503659	0.202505	7.425282	1.13E-13	2.58E-12
CNAG_00587	90.04277	0.672925	0.251147	2.679406	0.007375	0.018917
CNAG_00588	80.30999	0.618519	0.302191	2.046783	0.040679	0.081605
CNAG_00589	1226.122	-0.69266	0.114787	-6.03431	1.60E-09	1.97E-08
CNAG_00590	2101.532	-0.46798	0.166052	-2.81828	0.004828	0.013143
CNAG_00591	83.84999	1.0963	0.255278	4.29453	1.75E-05	9.24E-05
CNAG_00592	1810.156	-0.67537	0.272421	-2.47914	0.01317	0.031125
CNAG_00595	565.2274	1.457975	0.201112	7.24957	4.18E-13	9.10E-12
CNAG_00596	114.5575	0.944597	0.297654	3.173473	0.001506	0.00475
CNAG_00597	450.4421	-0.42371	0.203065	-2.08655	0.036929	0.07524
CNAG_00598	148.3201	-1.51194	0.251418	-6.01364	1.81E-09	2.22E-08
CNAG_00600	4314.558	-0.46052	0.119983	-3.83821	0.000124	0.000527
CNAG_00603	1179.079	-0.53867	0.142362	-3.7838	0.000154	0.000641
CNAG_00605	2361.089	1.75609	0.249968	7.025263	2.14E-12	4.20E-11
CNAG_00607	1253.43	-0.90089	0.186803	-4.82267	1.42E-06	9.51E-06
CNAG_00608	2067.548	-0.85243	0.152107	-5.60415	2.09E-08	2.08E-07
CNAG_07957	1590.415	-0.43196	0.11914	-3.62561	0.000288	0.00112
CNAG_00612	819.9033	0.611119	0.280823	2.176169	0.029543	0.062029
CNAG_00613	960.8369	-0.74588	0.216672	-3.44244	0.000576	0.002053
CNAG_07374	963.4661	-0.97917	0.178073	-5.49871	3.83E-08	3.63E-07
CNAG_00622	4231.882	-0.63595	0.109023	-5.83313	5.44E-09	5.95E-08
CNAG_00623	1382.955	0.448206	0.119876	3.7389	0.000185	0.000755
CNAG_00625	2522.288	0.504401	0.217429	2.319845	0.020349	0.045229
CNAG_00627	153.2503	1.571544	0.245975	6.389049	1.67E-10	2.39E-09
CNAG_00628	442.1569	-0.81361	0.213451	-3.81168	0.000138	0.000582
CNAG_07375	2682.367	0.537626	0.273979	1.962292	0.049728	0.096801
CNAG_00637	602.9653	0.835237	0.236536	3.53112	0.000414	0.001532
CNAG_00638	3592.542	1.029417	0.175524	5.864834	4.50E-09	5.01E-08
CNAG_00639	805.6647	-0.69988	0.242572	-2.88523	0.003911	0.010894
CNAG_00640	73231.75	-0.96675	0.194834	-4.96193	6.98E-07	5.01E-06
CNAG_07958	151.6408	-0.59542	0.273713	-2.17534	0.029605	0.062118
CNAG_00642	851.6809	1.495653	0.221677	6.746996	1.51E-11	2.55E-10
CNAG_12086	43.15723	-0.86826	0.345535	-2.5128	0.011978	0.028684
CNAG_00643	828.8099	-0.53936	0.222746	-2.42143	0.01546	0.035792
CNAG_07959	1494.993	-0.44349	0.179398	-2.47208	0.013433	0.031654
CNAG_00648	1158.281	0.770941	0.144811	5.323767	1.02E-07	8.72E-07
CNAG_00649	8261.979	-0.61816	0.10522	-5.87491	4.23E-09	4.74E-08
CNAG_00651	388.1655	0.432265	0.220526	1.960152	0.049978	0.097216
CNAG_00652	930.9735	1.132603	0.123458	9.17403	4.56E-20	2.08E-18
CNAG_00653	176.784	1.496026	0.305537	4.896387	9.76E-07	6.75E-06
CNAG_00655	36149.24	-1.2312	0.296462	-4.15297	3.28E-05	0.000161
CNAG_00656	45901.42	-0.93131	0.158403	-5.87939	4.12E-09	4.63E-08
CNAG_00663	1087.836	0.770821	0.150565	5.119521	3.06E-07	2.35E-06
CNAG_00664	40.81223	1.115947	0.395157	2.82406	0.004742	0.012939
CNAG_00665	3908.93	-0.3124	0.146491	-2.13257	0.03296	0.068115

CNAG_00667	1186.672	-0.27831	0.133776	-2.08042	0.037487	0.076273
CNAG_00668	3439.944	0.654524	0.126817	5.161159	2.45E-07	1.92E-06
CNAG_00669	796.3758	-0.79586	0.192464	-4.13511	3.55E-05	0.000173
CNAG_00672	35968.67	-0.93162	0.245625	-3.79285	0.000149	0.000621
CNAG_00677	797.4365	-1.02962	0.162935	-6.31919	2.63E-10	3.66E-09
CNAG_00679	205.1835	1.618185	0.215531	7.507893	6.01E-14	1.44E-12
CNAG_00680	1233.165	-1.11868	0.14207	-7.87412	3.43E-15	9.60E-14
CNAG_00681	1162.679	-0.97032	0.309376	-3.1364	0.00171	0.005311
CNAG_00682	4184.914	0.765038	0.196197	3.899324	9.65E-05	0.000422
CNAG_00686	18784.4	0.800413	0.229019	3.494963	0.000474	0.001723
CNAG_00690	2087.902	-0.48554	0.126443	-3.83996	0.000123	0.000524
CNAG_00692	478.1794	0.574901	0.223469	2.572623	0.010093	0.024799
CNAG_00694	6284.553	-0.35426	0.110283	-3.21229	0.001317	0.004234
CNAG_00695	567.9167	0.516141	0.221586	2.329303	0.019843	0.04424
CNAG_00696	1888.03	1.259401	0.169367	7.43594	1.04E-13	2.40E-12
CNAG_00697	4093.38	-0.52852	0.105592	-5.00532	5.58E-07	4.09E-06
CNAG_00699	889.2494	-0.39915	0.143503	-2.78151	0.005411	0.01454
CNAG_00700	11990.85	-0.30103	0.103698	-2.90293	0.003697	0.010406
CNAG_00701	2948.798	-0.53429	0.106782	-5.00356	5.63E-07	4.12E-06
CNAG_12093	256.6175	-1.15402	0.287278	-4.01708	5.89E-05	0.000271
CNAG_00702	6628.18	-1.47623	0.232532	-6.34852	2.17E-10	3.07E-09
CNAG_00703	36034.78	-1.11436	0.284919	-3.91116	9.19E-05	0.000405
CNAG_00705	3845.603	-0.35867	0.138586	-2.58807	0.009651	0.023854
CNAG_00706	1397.196	-0.8161	0.163855	-4.98066	6.34E-07	4.59E-06
CNAG_00709	1453.971	-0.62783	0.15245	-4.11826	3.82E-05	0.000185
CNAG_00711	770.885	0.993451	0.170346	5.831963	5.48E-09	5.99E-08
CNAG_00714	706.6952	-0.36064	0.168999	-2.134	0.032843	0.067919
CNAG_00715	1373.828	-1.16246	0.236604	-4.91309	8.97E-07	6.27E-06
CNAG_00716	11839.18	-1.67838	0.252929	-6.63578	3.23E-11	5.14E-10
CNAG_00717	1318.785	0.999995	0.133367	7.498092	6.48E-14	1.54E-12
CNAG_00719	2205.397	-0.42079	0.118725	-3.54421	0.000394	0.001465
CNAG_00720	2106.588	0.503796	0.123786	4.069898	4.70E-05	0.000222
CNAG_00721	4042.611	-0.80442	0.165505	-4.86036	1.17E-06	7.97E-06
CNAG_00722	1899.656	-1.01347	0.194077	-5.222	1.77E-07	1.44E-06
CNAG_07381	1036.483	-1.03137	0.24447	-4.21878	2.46E-05	0.000124
CNAG_07382	3684.423	-0.65637	0.120743	-5.43605	5.45E-08	4.96E-07
CNAG_00727	1879.884	0.293696	0.128055	2.293518	0.021818	0.047989
CNAG_00728	479.5886	1.044602	0.14817	7.050017	1.79E-12	3.59E-11
CNAG_12097	19.67878	-0.9273	0.438919	-2.11269	0.034628	0.071062
CNAG_00730	6302.859	1.74464	0.263937	6.610066	3.84E-11	6.00E-10
CNAG_00732	2740.43	1.323729	0.199766	6.626381	3.44E-11	5.43E-10
CNAG_12100	48.427	-1.02568	0.337068	-3.04294	0.002343	0.007023
CNAG_00735	145.8135	-0.57495	0.230573	-2.49355	0.012647	0.030089
CNAG_00737	1027.735	0.62525	0.234861	2.662212	0.007763	0.019739
CNAG_12101	59.48457	-0.75753	0.335571	-2.25744	0.02398	0.052048
CNAG_00742	1886.503	-0.3203	0.107555	-2.97799	0.002901	0.008442
CNAG_00743	3224.951	-1.2946	0.127571	-10.1481	3.38E-24	2.26E-22

CNAG_00744	2590.119	0.712539	0.129604	5.497803	3.85E-08	3.65E-07
CNAG_00745	2981.53	0.614272	0.233352	2.63239	0.008479	0.021334
CNAG_00746	6755.083	-0.51318	0.113466	-4.52278	6.10E-06	3.60E-05
CNAG_00747	11163.83	-0.31737	0.149272	-2.12615	0.033491	0.068996
CNAG_12105	35.3136	0.6871	0.336741	2.040443	0.041306	0.082702
CNAG_00750	1238.757	-1.51607	0.260529	-5.8192	5.91E-09	6.43E-08
CNAG_12108	42.23534	1.470334	0.355183	4.139656	3.48E-05	0.00017
CNAG_00751	18.48476	1.210608	0.593991	2.03809	0.041541	0.08304
CNAG_12109	50.58834	-2.18414	0.382908	-5.70408	1.17E-08	1.22E-07
CNAG_00754	4155.842	-1.03913	0.162601	-6.39069	1.65E-10	2.38E-09
CNAG_00756	667.002	-0.47278	0.202767	-2.33165	0.019719	0.044026
CNAG_12110	105.4465	0.927726	0.241896	3.835223	0.000125	0.000533
CNAG_00758	556.7042	0.919075	0.155654	5.904615	3.53E-09	4.05E-08
CNAG_00762	706.3651	-0.70183	0.128689	-5.45373	4.93E-08	4.53E-07
CNAG_07960	599.0552	0.720213	0.15979	4.507252	6.57E-06	3.84E-05
CNAG_00764	2565.711	-0.88695	0.149174	-5.94574	2.75E-09	3.23E-08
CNAG_00765	629.6993	0.799692	0.162543	4.919868	8.66E-07	6.08E-06
CNAG_00766	1480.123	1.494147	0.186834	7.997186	1.27E-15	3.80E-14
CNAG_00769	1418.106	0.906879	0.321053	2.824703	0.004732	0.012918
CNAG_00771	14687.79	-1.02302	0.150084	-6.81631	9.34E-12	1.65E-10
CNAG_00774	7346.359	-0.75307	0.224616	-3.35269	0.0008	0.002727
CNAG_00775	1653.57	-0.33986	0.125244	-2.71355	0.006657	0.017365
CNAG_12113	40.39968	1.009595	0.423213	2.385548	0.017054	0.038872
CNAG_00776	15106.37	0.593483	0.196883	3.014399	0.002575	0.007632
CNAG_00778	1813.946	0.445248	0.125528	3.546993	0.00039	0.001454
CNAG_00779	33054.15	-0.91307	0.236482	-3.86105	0.000113	0.000485
CNAG_00785	28035.37	-0.67499	0.13563	-4.9767	6.47E-07	4.68E-06
CNAG_00788	14516.38	-1.31548	0.158918	-8.27773	1.26E-16	4.17E-15
CNAG_12114	202.5559	-1.27556	0.250049	-5.10122	3.37E-07	2.57E-06
CNAG_00791	2291.568	0.66852	0.259044	2.580717	0.00986	0.024323
CNAG_00792	2549.496	0.628568	0.318802	1.971654	0.048649	0.095003
CNAG_00793	1959.694	-0.77639	0.166514	-4.66259	3.12E-06	1.97E-05
CNAG_00794	2048.05	-0.34423	0.164139	-2.09721	0.035975	0.07356
CNAG_12117	165.028	1.646461	0.246291	6.685023	2.31E-11	3.75E-10
CNAG_00796	2259.353	1.85166	0.228321	8.109898	5.07E-16	1.58E-14
CNAG_00797	6413.662	-0.92175	0.107974	-8.53674	1.38E-17	4.96E-16
CNAG_07961	73.00434	0.820332	0.281481	2.914344	0.003564	0.010079
CNAG_00798	166.5638	2.421987	0.389981	6.210519	5.28E-10	6.95E-09
CNAG_00799	14150.75	0.923197	0.174425	5.292798	1.20E-07	1.01E-06
CNAG_00801	194.2578	1.028147	0.238279	4.314884	1.60E-05	8.49E-05
CNAG_00802	648.0187	1.136253	0.196788	5.77401	7.74E-09	8.27E-08
CNAG_00803	1573.296	0.29569	0.134835	2.19298	0.028309	0.059809
CNAG_00804	2269.403	-0.51762	0.148193	-3.49287	0.000478	0.001735
CNAG_00807	1271.495	0.841293	0.240093	3.504035	0.000458	0.00167
CNAG_00808	3899.354	-0.70982	0.303966	-2.3352	0.019533	0.043697
CNAG_00810	1251.889	-0.47452	0.133004	-3.56771	0.00036	0.001353
CNAG_00811	3173.971	-1.02033	0.226371	-4.50735	6.56E-06	3.84E-05

CNAG_00814	117.9764	1.157623	0.291911	3.965668	7.32E-05	0.000331
CNAG_00813	11.22133	3.117156	0.811153	3.84287	0.000122	0.000519
CNAG_00816	3125.245	0.53238	0.170245	3.127152	0.001765	0.005463
CNAG_00819	12244.56	-0.92934	0.321421	-2.89135	0.003836	0.010714
CNAG_00820	789.3654	-1.31749	0.147298	-8.94438	3.74E-19	1.61E-17
CNAG_00821	21710.82	-0.99752	0.290762	-3.43072	0.000602	0.002134
CNAG_00822	4017.661	-0.62876	0.121061	-5.19373	2.06E-07	1.64E-06
CNAG_12119	55.77802	1.593348	0.30321	5.254931	1.48E-07	1.22E-06
CNAG_00826	305.1559	2.349705	0.178765	13.14409	1.84E-39	3.05E-37
CNAG_12120	5.511598	2.740832	1.097299	2.497798	0.012497	0.029775
CNAG_00827	71.5498	1.266973	0.345197	3.67029	0.000242	0.000956
CNAG_00830	884.198	0.879699	0.224857	3.912263	9.14E-05	0.000403
CNAG_00831	925.1566	0.431376	0.143395	3.008298	0.002627	0.007772
CNAG_12122	131.3051	0.56759	0.259511	2.187151	0.028732	0.060573
CNAG_00833	79.39345	0.785137	0.26568	2.9552	0.003125	0.008973
CNAG_00834	2391.731	2.359302	0.15568	15.15481	7.04E-52	1.80E-49
CNAG_00835	554.9271	0.37775	0.178942	2.111016	0.034771	0.071337
CNAG_00836	484.7087	-0.665	0.170232	-3.90645	9.37E-05	0.000412
CNAG_00838	253.3647	-0.7519	0.316678	-2.37433	0.017581	0.03989
CNAG_00841	291.1984	-0.59088	0.242563	-2.43596	0.014852	0.034564
CNAG_07386	115.9505	-0.90739	0.232454	-3.90353	9.48E-05	0.000416
CNAG_00843	175.4271	0.750513	0.236234	3.176991	0.001488	0.0047
CNAG_00845	1573.907	-0.9103	0.114892	-7.92314	2.32E-15	6.60E-14
CNAG_00846	681.4978	-0.6246	0.137049	-4.5575	5.18E-06	3.11E-05
CNAG_00848	20564.91	4.218003	0.190524	22.13899	1.33E-108	2.65E-105
CNAG_00849	150.0275	1.545251	0.214515	7.203464	5.87E-13	1.25E-11
CNAG_00850	188.9952	0.556859	0.262098	2.124626	0.033618	0.06919
CNAG_00851	179.6787	1.491693	0.417955	3.569025	0.000358	0.001347
CNAG_00853	1256.51	-0.56954	0.192056	-2.96548	0.003022	0.008732
CNAG_00854	2773.346	-0.63336	0.21175	-2.99107	0.00278	0.008154
CNAG_07389	698.214	0.349427	0.134588	2.596267	0.009424	0.023372
CNAG_00859	142.5939	-0.94902	0.192072	-4.94093	7.78E-07	5.52E-06
CNAG_07459	221.5472	0.376822	0.181468	2.076522	0.037846	0.076813
CNAG_12128	12.17184	1.575551	0.581165	2.71102	0.006708	0.017481
CNAG_06800	3342.958	2.422315	0.251135	9.645482	5.14E-22	2.92E-20
CNAG_06799	3695.602	-0.69172	0.25751	-2.6862	0.007227	0.018609
CNAG_06798	1749.281	-0.62876	0.139958	-4.49246	7.04E-06	4.08E-05
CNAG_06796	1265.656	-0.31926	0.133235	-2.39621	0.016566	0.037891
CNAG_06792	1482.308	0.39574	0.175909	2.249678	0.024469	0.052936
CNAG_06791	2571.634	1.44387	0.242137	5.963033	2.48E-09	2.95E-08
CNAG_06790	3661.484	0.458326	0.154663	2.963385	0.003043	0.008779
CNAG_07460	1302.189	-0.34567	0.175527	-1.96933	0.048915	0.095381
CNAG_06787	64.40528	0.932621	0.27379	3.406335	0.000658	0.002306
CNAG_06785	2379.803	-0.71091	0.294074	-2.41744	0.01563	0.036114
CNAG_06782	2556.466	0.496698	0.17835	2.784958	0.005353	0.014415
CNAG_12130	42.9361	0.733125	0.3647	2.010211	0.044409	0.087801
CNAG_06779	2610.732	-0.65222	0.237729	-2.74353	0.006078	0.016089

CNAG_06778	2916.798	0.276298	0.115663	2.388819	0.016903	0.038561
CNAG_06777	120.4397	-1.17059	0.244312	-4.79138	1.66E-06	1.10E-05
CNAG_06775	2551.616	-0.80441	0.191542	-4.19965	2.67E-05	0.000134
CNAG_06774	887.8696	-1.01449	0.148645	-6.8249	8.80E-12	1.56E-10
CNAG_06771	853.3748	1.428093	0.213093	6.701744	2.06E-11	3.40E-10
CNAG_06770	33719.17	0.340329	0.1418	2.400062	0.016392	0.03758
CNAG_06767	12023.13	-0.64169	0.293754	-2.18444	0.02893	0.060943
CNAG_06766	2630.628	-0.57048	0.147271	-3.87366	0.000107	0.000464
CNAG_12131	15.04918	-1.08116	0.544174	-1.9868	0.046945	0.092173
CNAG_07962	157.9886	-1.34328	0.316961	-4.23798	2.26E-05	0.000115
CNAG_06763	896.0637	-0.51381	0.156642	-3.28015	0.001038	0.003425
CNAG_06762	4569.677	-1.43245	0.17625	-8.1274	4.39E-16	1.38E-14
CNAG_06760	906.7778	-0.5504	0.119879	-4.59127	4.41E-06	2.69E-05
CNAG_12135	79.54064	1.241022	0.264849	4.68577	2.79E-06	1.78E-05
CNAG_06759	1371.778	1.441176	0.184765	7.800034	6.19E-15	1.68E-13
CNAG_06757	1039.381	0.479623	0.140635	3.410399	0.000649	0.002275
CNAG_06756	812.8476	-0.98188	0.254841	-3.85289	0.000117	0.0005
CNAG_06755	6350.936	-0.79589	0.111649	-7.12857	1.01E-12	2.10E-11
CNAG_06754	5989.917	-0.45398	0.105226	-4.31432	1.60E-05	8.50E-05
CNAG_06753	4739.921	-0.47694	0.139706	-3.41391	0.00064	0.00225
CNAG_06752	559.7419	-0.47455	0.187332	-2.53318	0.011303	0.027308
CNAG_06751	1980.53	-0.3689	0.17084	-2.15935	0.030823	0.0643
CNAG_12139	35.07909	-0.72802	0.343902	-2.11695	0.034264	0.070428
CNAG_06748	1965.999	-0.42778	0.114728	-3.72868	0.000192	0.000782
CNAG_06747	22157.36	-1.26896	0.197765	-6.41651	1.39E-10	2.03E-09
CNAG_06746	20052.37	-0.86071	0.157617	-5.46077	4.74E-08	4.38E-07
CNAG_06745	16926.67	-0.90702	0.169603	-5.34787	8.90E-08	7.75E-07
CNAG_06744	1131.1	-0.62056	0.169213	-3.66734	0.000245	0.000966
CNAG_06742	2048.459	-0.43268	0.125609	-3.4447	0.000572	0.002041
CNAG_06741	1923.738	-0.73759	0.252947	-2.916	0.003545	0.010029
CNAG_06740	2169.923	0.538888	0.273538	1.970063	0.048831	0.095272
CNAG_06737	2238.535	0.349369	0.173266	2.016377	0.043761	0.086822
CNAG_06734	670.5169	-1.12963	0.282877	-3.99335	6.51E-05	0.000298
CNAG_06733	2025.878	1.045084	0.175146	5.966918	2.42E-09	2.89E-08
CNAG_06732	2793.155	0.704098	0.188728	3.730747	0.000191	0.000777
CNAG_06729	645.0415	-0.55886	0.17123	-3.26379	0.001099	0.003596
CNAG_06727	382.0397	1.092433	0.191245	5.712216	1.12E-08	1.16E-07
CNAG_06726	520.3828	0.677607	0.196831	3.44258	0.000576	0.002053
CNAG_06725	969.974	-0.40852	0.139871	-2.92067	0.003493	0.009905
CNAG_06724	748.2513	0.668713	0.19419	3.443607	0.000574	0.002046
CNAG_06723	3053.912	-1.5817	0.199999	-7.90855	2.60E-15	7.34E-14
CNAG_06722	145.2193	1.342227	0.236464	5.676248	1.38E-08	1.41E-07
CNAG_07465	635.6865	-0.85724	0.15478	-5.53848	3.05E-08	2.97E-07
CNAG_06717	2075.769	-0.56913	0.207966	-2.73667	0.006207	0.016374
CNAG_06716	2566.753	-0.79593	0.22562	-3.52773	0.000419	0.001548
CNAG_06713	3338.57	-0.73461	0.14301	-5.13678	2.79E-07	2.16E-06
CNAG_06711	1173.468	-0.54238	0.227702	-2.38196	0.017221	0.039191

CNAG_06710	2110.955	-0.52014	0.245045	-2.12264	0.033784	0.06951
CNAG_06708	611.9189	-0.50618	0.157197	-3.22001	0.001282	0.004135
CNAG_06707	110.815	-0.4951	0.239901	-2.06378	0.039038	0.078687
CNAG_06706	280.3592	0.579372	0.17638	3.284786	0.001021	0.00338
CNAG_10500	766813.5	2.191363	1.066599	2.054533	0.039924	0.080232
CNAG_10501	16003.16	2.356122	1.069601	2.202804	0.027609	0.058563
CNAG_10502	1053222	2.089125	1.056856	1.976735	0.048072	0.094014
CNAG_10503	32.30304	1.8866	0.799828	2.358758	0.018336	0.041368
CNAG_03595	3890.392	0.995966	0.2521	3.950678	7.79E-05	0.00035
CNAG_12146	31.31839	1.184088	0.387957	3.052114	0.002272	0.006839
CNAG_03597	174.5933	0.963831	0.191318	5.037859	4.71E-07	3.49E-06
CNAG_03600	276.7009	1.318739	0.291087	4.53039	5.89E-06	3.48E-05
CNAG_07964	1344.118	-0.46184	0.197537	-2.33798	0.019388	0.04341
CNAG_03602	8768.433	-1.15018	0.178528	-6.4426	1.17E-10	1.73E-09
CNAG_03603	1674.141	-0.94273	0.138728	-6.79554	1.08E-11	1.88E-10
CNAG_03605	711.552	-0.59243	0.268627	-2.20541	0.027425	0.058237
CNAG_03606	9286.377	-0.39155	0.172431	-2.27078	0.02316	0.050627
CNAG_03607	378.0422	0.543255	0.187286	2.900673	0.003724	0.010462
CNAG_03609	1015.614	0.739404	0.142932	5.173118	2.30E-07	1.81E-06
CNAG_03612	7967.785	-0.8168	0.237715	-3.43603	0.00059	0.002099
CNAG_03619	494.5676	0.630327	0.141867	4.443088	8.87E-06	5.00E-05
CNAG_03621	15599.67	0.490691	0.193071	2.541497	0.011038	0.026773
CNAG_03624	4634.178	-0.95444	0.156996	-6.07939	1.21E-09	1.51E-08
CNAG_03627	16505.56	0.956087	0.188316	5.077046	3.83E-07	2.88E-06
CNAG_03628	2530.206	0.510491	0.155065	3.292108	0.000994	0.003303
CNAG_03629	7428	-1.33232	0.139612	-9.54306	1.39E-21	7.45E-20
CNAG_03630	66.53463	-0.60474	0.27872	-2.16969	0.030031	0.062895
CNAG_03631	1813.638	0.891433	0.198737	4.485486	7.27E-06	4.20E-05
CNAG_03632	1198.482	-0.47278	0.157938	-2.99342	0.002759	0.008108
CNAG_03634	1050.151	-1.03716	0.158793	-6.53152	6.51E-11	9.89E-10
CNAG_03636	1254.098	-0.79755	0.229453	-3.47588	0.000509	0.001842
CNAG_03637	2258.537	0.684388	0.168724	4.056253	4.99E-05	0.000234
CNAG_03638	3157.53	0.54782	0.257801	2.124969	0.033589	0.069163
CNAG_03639	417.1732	0.620836	0.276917	2.241958	0.024964	0.053743
CNAG_07471	60.26821	1.053324	0.343348	3.067806	0.002156	0.006535
CNAG_07472	761.1659	-0.42611	0.165626	-2.57272	0.01009	0.024799
CNAG_03641	7056.314	-0.59743	0.111804	-5.34356	9.11E-08	7.91E-07
CNAG_03642	259.3869	1.261896	0.399573	3.158112	0.001588	0.004983
CNAG_03643	189.2327	0.603655	0.248578	2.428428	0.015164	0.035212
CNAG_03644	5135.088	-1.27648	0.176924	-7.21487	5.40E-13	1.16E-11
CNAG_03645	6874.52	-0.40955	0.192445	-2.12812	0.033327	0.068695
CNAG_03646	1257.023	-0.56735	0.125071	-4.53621	5.73E-06	3.39E-05
CNAG_03647	699.7721	-0.6639	0.171075	-3.88077	0.000104	0.000451
CNAG_03648	4989.587	0.631354	0.162793	3.878264	0.000105	0.000455
CNAG_03649	695.7367	-1.42768	0.160105	-8.91713	4.79E-19	1.99E-17
CNAG_03651	1751.97	-0.42893	0.158161	-2.712	0.006688	0.017435
CNAG_03654	1291.498	0.407957	0.164229	2.484072	0.012989	0.030782

CNAG_03655	2649.577	-0.7322	0.302344	-2.42174	0.015446	0.035773
CNAG_03658	634.4197	-0.49484	0.140381	-3.52501	0.000423	0.001561
CNAG_03661	618.8784	-0.3602	0.176593	-2.03972	0.041379	0.082799
CNAG_03662	2139.38	-0.55138	0.268501	-2.05355	0.040019	0.080383
CNAG_03663	1150.063	-0.78663	0.164341	-4.78659	1.70E-06	1.12E-05
CNAG_03666	384.0848	-1.1016	0.201792	-5.4591	4.79E-08	4.41E-07
CNAG_03667	4109.944	0.74326	0.231658	3.20843	0.001335	0.004278
CNAG_03669	2372.469	1.686099	0.231051	7.297517	2.93E-13	6.45E-12
CNAG_03671	291.5966	-0.71306	0.201261	-3.54298	0.000396	0.00147
CNAG_03673	937.5964	0.32104	0.15663	2.049677	0.040396	0.081098
CNAG_03675	3328.283	-0.89656	0.121679	-7.36825	1.73E-13	3.86E-12
CNAG_03676	1084.534	-0.36281	0.124578	-2.91232	0.003588	0.010134
CNAG_03679	678.3557	1.730977	0.265552	6.518405	7.11E-11	1.07E-09
CNAG_03680	1166.737	1.022799	0.135957	7.522977	5.35E-14	1.30E-12
CNAG_03683	1155.698	-0.30024	0.120058	-2.50075	0.012393	0.029563
CNAG_03685	837.5046	1.124191	0.190493	5.901481	3.60E-09	4.11E-08
CNAG_03688	4247.856	1.820354	0.227943	7.986007	1.39E-15	4.12E-14
CNAG_03689	2771.664	1.28328	0.198299	6.471429	9.71E-11	1.44E-09
CNAG_07473	5690.096	-0.41344	0.111159	-3.71933	0.0002	0.000807
CNAG_03692	788.8265	1.306756	0.186194	7.01826	2.25E-12	4.41E-11
CNAG_03693	1602.085	-0.61788	0.207412	-2.97899	0.002892	0.008424
CNAG_03695	595.3856	-0.55994	0.141921	-3.94548	7.96E-05	0.000357
CNAG_03697	423.8554	0.654175	0.216269	3.024827	0.002488	0.007412
CNAG_03698	558.3351	-0.30052	0.140062	-2.14564	0.031901	0.066289
CNAG_03702	832.161	-0.47915	0.221455	-2.16362	0.030493	0.063763
CNAG_03705	4684.418	0.623166	0.205343	3.034749	0.002407	0.007191
CNAG_03707	536.9054	-0.43411	0.150094	-2.89228	0.003825	0.010689
CNAG_03708	1657.736	-0.36722	0.133729	-2.74601	0.006033	0.015978
CNAG_03709	398.0028	0.630182	0.268583	2.346326	0.01896	0.042509
CNAG_03712	322.3442	-0.56124	0.172065	-3.26179	0.001107	0.00362
CNAG_03713	292.7512	1.643787	0.441313	3.72476	0.000196	0.000792
CNAG_07475	27.43798	2.017515	0.483834	4.16985	3.05E-05	0.000151
CNAG_03715	1069.591	-1.11407	0.159668	-6.9774	3.01E-12	5.66E-11
CNAG_03716	1943.136	-0.57726	0.219939	-2.62465	0.008674	0.021742
CNAG_03718	1405.213	0.620384	0.162646	3.814323	0.000137	0.000577
CNAG_03719	1830.65	1.105627	0.330295	3.347391	0.000816	0.002772
CNAG_03720	1067.888	-0.28489	0.139453	-2.04288	0.041065	0.082253
CNAG_03721	3525.506	0.514569	0.13569	3.792243	0.000149	0.000622
CNAG_03724	4890.066	-0.66362	0.164844	-4.02575	5.68E-05	0.000263
CNAG_03725	7681.052	-0.41246	0.15485	-2.66365	0.00773	0.019667
CNAG_12161	241.1053	1.396747	0.253606	5.507539	3.64E-08	3.48E-07
CNAG_03727	2654.979	1.108018	0.271896	4.07516	4.60E-05	0.000217
CNAG_03728	1154.579	0.909706	0.306784	2.9653	0.003024	0.008734
CNAG_03729	499.4965	0.759487	0.159567	4.759664	1.94E-06	1.27E-05
CNAG_03731	739.9512	-0.48094	0.142761	-3.36884	0.000755	0.00259
CNAG_03732	856.3511	0.488374	0.185829	2.628085	0.008587	0.021558
CNAG_03733	123.7435	1.2162	0.279743	4.347564	1.38E-05	7.47E-05

CNAG_03735	1713.6	-0.71116	0.155989	-4.55903	5.14E-06	3.09E-05
CNAG_07476	253.6873	1.146517	0.160981	7.122075	1.06E-12	2.19E-11
CNAG_03738	2139.802	0.562943	0.181159	3.107456	0.001887	0.005789
CNAG_03739	34901.92	-0.68834	0.151609	-4.54023	5.62E-06	3.33E-05
CNAG_03743	737.4167	-0.61341	0.141527	-4.33424	1.46E-05	7.86E-05
CNAG_03746	982.2656	1.26251	0.132858	9.502723	2.04E-21	1.07E-19
CNAG_03747	31935.27	-1.00985	0.230711	-4.37713	1.20E-05	6.61E-05
CNAG_03748	4810.522	-0.30244	0.146643	-2.06244	0.039166	0.078828
CNAG_07477	893.2449	0.77518	0.337345	2.297884	0.021568	0.047527
CNAG_12163	9.046741	1.393889	0.665265	2.09524	0.03615	0.07388
CNAG_03753	1101.647	0.503292	0.172211	2.92253	0.003472	0.00985
CNAG_03754	7.993026	2.821973	0.877872	3.214561	0.001306	0.004206
CNAG_07479	436.8169	1.389528	0.36555	3.801198	0.000144	0.000604
CNAG_07480	49.01367	1.518689	0.315117	4.819449	1.44E-06	9.65E-06
CNAG_03759	89.01567	3.996731	0.460552	8.678129	4.02E-18	1.53E-16
CNAG_03762	3605.467	-1.02338	0.144731	-7.0709	1.54E-12	3.12E-11
CNAG_03764	90.25136	1.025567	0.265372	3.864642	0.000111	0.000478
CNAG_12165	7.460608	1.784258	0.735506	2.425892	0.015271	0.035418
CNAG_03765	14656.8	1.090572	0.136561	7.985996	1.39E-15	4.12E-14
CNAG_03766	1056.352	-0.43371	0.169412	-2.56008	0.010465	0.025617
CNAG_12166	63.53157	-1.03179	0.425909	-2.42256	0.015412	0.035713
CNAG_12168	68.34374	-1.21299	0.407242	-2.97855	0.002896	0.008433
CNAG_03771	19092.9	1.359351	0.153196	8.873269	7.10E-19	2.91E-17
CNAG_03772	17029.28	-2.62859	0.245405	-10.7113	9.01E-27	6.76E-25
CNAG_03775	4994.291	0.679101	0.223269	3.041631	0.002353	0.00705
CNAG_03779	1603.138	-0.69438	0.21224	-3.27167	0.001069	0.003509
CNAG_03780	42384.41	-1.29188	0.306797	-4.21086	2.54E-05	0.000129
CNAG_03781	1362.487	-0.45531	0.204806	-2.22312	0.026208	0.056011
CNAG_03782	79.8312	4.202111	0.371887	11.29944	1.32E-29	1.22E-27
CNAG_03783	75.39281	2.136087	0.288396	7.406779	1.29E-13	2.93E-12
CNAG_03785	3479.562	0.763732	0.162475	4.700614	2.59E-06	1.67E-05
CNAG_03786	841.9498	-0.34827	0.143171	-2.43251	0.014994	0.034858
CNAG_03787	20601.09	-0.69058	0.256488	-2.69245	0.007093	0.018323
CNAG_03789	2036.087	-0.78416	0.183953	-4.26282	2.02E-05	0.000104
CNAG_03790	4322.542	-0.33449	0.166375	-2.01046	0.044382	0.087784
CNAG_03791	2247.831	0.974111	0.267425	3.642561	0.00027	0.001056
CNAG_03792	992.3894	0.416018	0.127787	3.255553	0.001132	0.003695
CNAG_03793	2236.854	-0.76297	0.263496	-2.89558	0.003785	0.0106
CNAG_03794	1910.242	1.658232	0.312224	5.311025	1.09E-07	9.28E-07
CNAG_03806	1484.044	1.12614	0.138751	8.116239	4.81E-16	1.50E-14
CNAG_03808	1395.833	1.084177	0.125233	8.657306	4.83E-18	1.80E-16
CNAG_03810	2200.032	-0.7666	0.261931	-2.92671	0.003426	0.009739
CNAG_03812	5484.087	-0.82352	0.137813	-5.97558	2.29E-09	2.75E-08
CNAG_03813	2399.907	0.337534	0.16138	2.091549	0.036479	0.074457
CNAG_03814	3528.402	-0.68072	0.268282	-2.53734	0.01117	0.027045
CNAG_03816	4803.432	0.661098	0.243009	2.720462	0.006519	0.017062
CNAG_03817	2014.74	-0.79017	0.2339	-3.37823	0.00073	0.002511

CNAG_03819	6827.004	-1.43667	0.187943	-7.64418	2.10E-14	5.33E-13
CNAG_03823	1314.307	0.492094	0.219611	2.240747	0.025042	0.053868
CNAG_03824	6880.333	1.441437	0.11979	12.03306	2.38E-33	2.82E-31
CNAG_12173	68.99309	1.408278	0.293428	4.799404	1.59E-06	1.06E-05
CNAG_03827	1098.971	0.435501	0.143852	3.027434	0.002466	0.007354
CNAG_03828	398.3656	1.58126	0.177016	8.932852	4.15E-19	1.75E-17
CNAG_03833	107.7961	1.001556	0.27585	3.630797	0.000283	0.001099
CNAG_12175	216.1735	0.575993	0.223313	2.579303	0.0099	0.024407
CNAG_03837	495.6285	0.78472	0.231513	3.389531	0.0007	0.002423
CNAG_03838	92.67424	1.815616	0.276796	6.559412	5.40E-11	8.29E-10
CNAG_03840	1899.866	-0.45699	0.205018	-2.22903	0.025812	0.055299
CNAG_03841	3621.305	0.642855	0.296985	2.164603	0.030418	0.063639
CNAG_03842	60.85488	1.071987	0.30238	3.545163	0.000392	0.001462
CNAG_03844	1414.377	1.083268	0.157534	6.876409	6.14E-12	1.12E-10
CNAG_03845	3966.599	-0.53757	0.138051	-3.894	9.86E-05	0.00043
CNAG_03848	2521.138	-1.54831	0.241875	-6.40128	1.54E-10	2.23E-09
CNAG_03849	2031.795	1.317981	0.325722	4.04634	5.20E-05	0.000243
CNAG_03855	463.5988	0.592608	0.196516	3.015565	0.002565	0.007611
CNAG_03856	15866.54	1.837633	0.114867	15.99787	1.32E-57	4.70E-55
CNAG_03857	4896.543	1.24024	0.154753	8.014321	1.11E-15	3.32E-14
CNAG_03858	3267.384	0.64322	0.154603	4.160455	3.18E-05	0.000157
CNAG_03859	1388.971	-0.77859	0.265201	-2.93585	0.003326	0.009484
CNAG_03861	9120.149	-0.81563	0.175556	-4.64596	3.38E-06	2.12E-05
CNAG_03863	4094.821	-0.35092	0.120201	-2.91946	0.003506	0.009933
CNAG_03864	2576.301	0.455671	0.132546	3.437839	0.000586	0.002086
CNAG_03865	2358.253	0.62858	0.183406	3.427261	0.00061	0.002152
CNAG_03868	1239.735	-0.44839	0.154757	-2.89737	0.003763	0.010555
CNAG_03870	1471.175	0.286275	0.140401	2.038975	0.041453	0.082909
CNAG_03871	1151.474	-0.65307	0.203345	-3.21164	0.00132	0.004242
CNAG_03873	8124.169	1.317306	0.295812	4.453181	8.46E-06	4.80E-05
CNAG_03875	1583.03	0.378947	0.135924	2.787935	0.005305	0.014298
CNAG_03876	3205.819	0.277485	0.123623	2.244602	0.024794	0.053463
CNAG_03877	2094.031	0.482011	0.119169	4.044764	5.24E-05	0.000244
CNAG_03878	1322.652	-0.68596	0.175366	-3.91159	9.17E-05	0.000404
CNAG_07482	1797.412	1.689076	0.251109	6.726465	1.74E-11	2.90E-10
CNAG_03881	2966.649	2.061097	0.210735	9.780521	1.37E-22	8.16E-21
CNAG_03885	2667.022	-0.71193	0.219548	-3.24272	0.001184	0.003852
CNAG_03886	2022.77	-0.27325	0.123425	-2.21388	0.026837	0.057156
CNAG_03890	1494.648	-0.54723	0.120093	-4.55668	5.20E-06	3.12E-05
CNAG_03892	11415.44	0.441398	0.204713	2.156178	0.03107	0.064747
CNAG_03893	527.2719	-0.69777	0.197875	-3.52632	0.000421	0.001555
CNAG_03894	98.08811	-0.97222	0.311114	-3.12496	0.001778	0.005496
CNAG_12183	36.86216	2.500714	0.534643	4.677352	2.91E-06	1.85E-05
CNAG_03897	1334.117	-0.42362	0.173876	-2.43631	0.014838	0.034545
CNAG_03898	1864.18	-0.66013	0.134279	-4.9161	8.83E-07	6.20E-06
CNAG_03899	7793.163	0.913699	0.18652	4.898671	9.65E-07	6.71E-06
CNAG_03901	718.8125	0.334439	0.151537	2.206972	0.027316	0.058051

CNAG_12185	29.19804	1.28475	0.508157	2.528255	0.011463	0.027635
CNAG_03904	4459.868	0.292104	0.137278	2.127826	0.033351	0.068727
CNAG_03906	537.1179	1.510137	0.170437	8.860364	7.98E-19	3.23E-17
CNAG_03905	552.418	-0.44489	0.226363	-1.96537	0.049371	0.096176
CNAG_03907	386.4624	-0.69663	0.187663	-3.71211	0.000206	0.000828
CNAG_03911	123.8869	0.800524	0.28768	2.782693	0.005391	0.014501
CNAG_03912	138.7883	-0.42334	0.202486	-2.09073	0.036552	0.074588
CNAG_03913	596.3397	1.320132	0.18882	6.991476	2.72E-12	5.25E-11
CNAG_03918	1702.366	0.633813	0.241737	2.621911	0.008744	0.021869
CNAG_03919	842.8093	-0.81405	0.272454	-2.98783	0.00281	0.008226
CNAG_03920	11070.11	-0.43442	0.116267	-3.73641	0.000187	0.000762
CNAG_03921	532.0101	-0.60571	0.219034	-2.76536	0.005686	0.015187
CNAG_03922	709.0913	3.435778	0.217055	15.82903	1.96E-56	6.24E-54
CNAG_03925	391.4479	-0.63954	0.160986	-3.97264	7.11E-05	0.000322
CNAG_03926	3070.962	-0.66558	0.110759	-6.00931	1.86E-09	2.28E-08
CNAG_03927	637.4183	-0.38112	0.187966	-2.02758	0.042603	0.084822
CNAG_03931	9311.152	-0.45813	0.170594	-2.68551	0.007242	0.018641
CNAG_03935	4863.826	1.031015	0.115485	8.927671	4.35E-19	1.82E-17
CNAG_03936	6471.408	0.942506	0.201696	4.672907	2.97E-06	1.88E-05
CNAG_03937	1390.804	1.158204	0.274846	4.214015	2.51E-05	0.000127
CNAG_03938	97.68533	1.595819	0.255858	6.237132	4.46E-10	5.92E-09
CNAG_03939	2114.836	-0.75812	0.130187	-5.82331	5.77E-09	6.29E-08
CNAG_03940	2626.931	-0.75756	0.166749	-4.54312	5.54E-06	3.30E-05
CNAG_03941	6470.774	0.489465	0.234685	2.085623	0.037013	0.075353
CNAG_03944	10904.01	0.276413	0.118947	2.323832	0.020134	0.044829
CNAG_03946	1363.254	0.94345	0.181252	5.205185	1.94E-07	1.57E-06
CNAG_07486	976.2676	0.728243	0.17486	4.16473	3.12E-05	0.000154
CNAG_12191	16.18894	1.244076	0.57803	2.152269	0.031376	0.065283
CNAG_03949	1070.894	-0.9293	0.156536	-5.9367	2.91E-09	3.38E-08
CNAG_03951	1624.873	-1.03976	0.193102	-5.3845	7.26E-08	6.42E-07
CNAG_03952	3918.141	-0.73368	0.281076	-2.61024	0.009048	0.02253
CNAG_03957	2874.014	-0.37891	0.118247	-3.20442	0.001353	0.004326
CNAG_03958	2466.05	1.346427	0.239752	5.615911	1.96E-08	1.95E-07
CNAG_03959	5591.975	0.656647	0.193644	3.39101	0.000696	0.002413
CNAG_03961	1634.495	-0.67902	0.187525	-3.62093	0.000294	0.001136
CNAG_03964	965.2964	-1.21273	0.172493	-7.03062	2.06E-12	4.06E-11
CNAG_03965	1645.909	-0.63881	0.25325	-2.52246	0.011654	0.028027
CNAG_03966	973.6215	-1.04975	0.234129	-4.48364	7.34E-06	4.23E-05
CNAG_03967	3245.457	0.315494	0.126334	2.4973	0.012514	0.029808
CNAG_03968	3347.248	-0.39701	0.117286	-3.38497	0.000712	0.00246
CNAG_07487	17131.04	-0.53415	0.239171	-2.23332	0.025528	0.054808
CNAG_07488	3911.138	-0.84779	0.209484	-4.04703	5.19E-05	0.000242
CNAG_03974	4014.632	2.56055	0.17877	14.32312	1.57E-46	3.67E-44
CNAG_03976	1481.588	-0.57837	0.127875	-4.52293	6.10E-06	3.60E-05
CNAG_03977	1093.747	-1.12191	0.154433	-7.26471	3.74E-13	8.18E-12
CNAG_03978	1411.669	0.613354	0.140266	4.372805	1.23E-05	6.73E-05
CNAG_03979	769.4953	-0.43194	0.158474	-2.72563	0.006418	0.016847

CNAG_03982	1534.046	-0.33764	0.133789	-2.52366	0.011614	0.02794
CNAG_03984	926.8847	-0.77827	0.258174	-3.0145	0.002574	0.007632
CNAG_03986	2503.965	-0.73191	0.129976	-5.63113	1.79E-08	1.80E-07
CNAG_03987	5941.957	0.317733	0.13187	2.40944	0.015977	0.036783
CNAG_03988	219.3306	0.877371	0.412714	2.125854	0.033515	0.069029
CNAG_12196	42.77324	2.521474	0.368162	6.848809	7.45E-12	1.34E-10
CNAG_03991	2108.092	0.825965	0.144814	5.703612	1.17E-08	1.22E-07
CNAG_03992	860.5595	0.368535	0.176667	2.08604	0.036975	0.075296
CNAG_03993	1563.657	-1.21016	0.240926	-5.02297	5.09E-07	3.75E-06
CNAG_03995	911.5747	0.837327	0.338004	2.47727	0.013239	0.031246
CNAG_03996	9445.861	-0.97995	0.21101	-4.6441	3.42E-06	2.14E-05
CNAG_03997	57.21172	-1.31065	0.397271	-3.29914	0.00097	0.003228
CNAG_03998	1170.607	0.423725	0.146386	2.894581	0.003797	0.010619
CNAG_03999	643.1724	3.280671	0.205092	15.99607	1.36E-57	4.70E-55
CNAG_04000	788.7899	-0.53317	0.168302	-3.16796	0.001535	0.004835
CNAG_04003	667.692	0.83601	0.142224	5.878144	4.15E-09	4.66E-08
CNAG_04004	79157.61	-1.01089	0.21898	-4.61638	3.90E-06	2.41E-05
CNAG_04006	20.1764	1.788476	0.621864	2.875992	0.004028	0.011186
CNAG_12197	99.18685	-0.66247	0.293755	-2.25518	0.024122	0.052313
CNAG_04010	4326.807	-0.24487	0.112323	-2.18006	0.029253	0.06151
CNAG_04011	21682.22	-1.10706	0.314623	-3.5187	0.000434	0.001591
CNAG_04014	7233.727	0.865712	0.141539	6.11641	9.57E-10	1.22E-08
CNAG_12198	64.69789	0.967346	0.263937	3.66507	0.000247	0.000975
CNAG_12199	33.19671	1.39927	0.377605	3.705647	0.000211	0.000848
CNAG_04017	2373.833	1.231888	0.186933	6.59001	4.40E-11	6.77E-10
CNAG_07489	1320.746	1.429743	0.162848	8.779616	1.64E-18	6.48E-17
CNAG_04021	24313.18	-1.05771	0.216915	-4.87614	1.08E-06	7.42E-06
CNAG_04023	1790.279	-0.77235	0.18913	-4.0837	4.43E-05	0.000211
CNAG_04024	197.6213	-0.67807	0.176009	-3.85249	0.000117	0.000501
CNAG_04025	73.37504	1.293398	0.43212	2.993145	0.002761	0.008108
CNAG_04030	697.307	-0.7837	0.223465	-3.50703	0.000453	0.001655
CNAG_04032	8159.853	0.393009	0.128178	3.066121	0.002169	0.006567
CNAG_04033	3261	0.366669	0.148015	2.477251	0.01324	0.031246
CNAG_04036	243.8133	0.584818	0.169352	3.453265	0.000554	0.001984
CNAG_04037	1661.991	-0.7397	0.262623	-2.8166	0.004854	0.013203
CNAG_04039	3880.424	0.458696	0.182034	2.519839	0.011741	0.028188
CNAG_04041	1026.488	-0.31333	0.148555	-2.10917	0.03493	0.071626
CNAG_04043	570.7421	1.543255	0.215212	7.170871	7.45E-13	1.57E-11
CNAG_04044	1316.292	-0.70286	0.117814	-5.96588	2.43E-09	2.90E-08
CNAG_04045	1438.096	-0.70568	0.160492	-4.397	1.10E-05	6.06E-05
CNAG_04048	2798.193	-0.47942	0.120719	-3.97141	7.14E-05	0.000323
CNAG_04049	1391.898	-0.43338	0.125828	-3.44423	0.000573	0.002043
CNAG_04050	5403.05	-0.51192	0.248374	-2.0611	0.039293	0.079044
CNAG_04055	768.6993	0.555268	0.132413	4.193468	2.75E-05	0.000137
CNAG_04056	1480.399	-0.58227	0.187971	-3.09767	0.00195	0.005963
CNAG_07491	858.5335	0.939814	0.127154	7.391123	1.46E-13	3.27E-12
CNAG_07492	126.5635	2.739471	0.411451	6.658073	2.77E-11	4.46E-10

CNAG_07493	500.5136	3.273028	0.213224	15.35022	3.53E-53	9.67E-51
CNAG_04062	2071.828	-0.50241	0.108434	-4.63331	3.60E-06	2.24E-05
CNAG_04064	6505.292	0.534943	0.206165	2.594728	0.009467	0.023457
CNAG_04067	1060.961	1.298878	0.249813	5.199398	2.00E-07	1.61E-06
CNAG_04068	14342.69	-1.07501	0.261035	-4.11828	3.82E-05	0.000185
CNAG_04070	235.0909	1.261339	0.41474	3.041273	0.002356	0.007053
CNAG_04071	4369.343	0.572949	0.212367	2.697922	0.006977	0.018071
CNAG_04072	1693.007	-0.83628	0.270849	-3.08764	0.002018	0.006154
CNAG_04073	1719.128	-0.53295	0.177987	-2.99432	0.002751	0.008092
CNAG_12203	178.6501	0.965785	0.274427	3.519279	0.000433	0.001589
CNAG_07494	2204.372	-0.60874	0.176641	-3.4462	0.000569	0.00203
CNAG_04076	1481.663	1.470222	0.29482	4.986853	6.14E-07	4.46E-06
CNAG_12204	17.59715	-1.28435	0.467991	-2.74439	0.006062	0.016052
CNAG_04079	1597.165	-0.49028	0.147442	-3.32521	0.000884	0.002966
CNAG_12206	47.07021	-1.78492	0.480385	-3.7156	0.000203	0.000819
CNAG_04082	7185.914	-0.70586	0.128634	-5.48733	4.08E-08	3.82E-07
CNAG_04083	1906.445	0.915583	0.236699	3.868126	0.00011	0.000472
CNAG_12208	96.18157	-1.42777	0.414203	-3.44703	0.000567	0.002025
CNAG_12209	180.6448	1.373764	0.201288	6.824854	8.80E-12	1.56E-10
CNAG_04085	504.3608	2.577574	0.157995	16.31432	7.81E-60	3.10E-57
CNAG_12210	582.1128	0.998912	0.190499	5.243666	1.57E-07	1.29E-06
CNAG_04086	68.43611	1.052548	0.293945	3.580763	0.000343	0.001297
CNAG_04090	4267.285	0.611787	0.21171	2.889743	0.003856	0.010761
CNAG_04091	115.9764	0.886536	0.315424	2.810615	0.004945	0.013433
CNAG_04092	63.31573	1.238282	0.275242	4.498882	6.83E-06	3.98E-05
CNAG_04093	74.05258	-0.94365	0.292783	-3.22302	0.001268	0.004097
CNAG_04095	338.455	-0.69327	0.157772	-4.39413	1.11E-05	6.14E-05
CNAG_04096	186.6398	-0.81975	0.223937	-3.66061	0.000252	0.000989
CNAG_04098	2432.848	2.688304	0.21962	12.24073	1.88E-34	2.34E-32
CNAG_12212	496.5224	-0.76664	0.185333	-4.13656	3.53E-05	0.000172
CNAG_07498	1123.102	1.23341	0.22854	5.396906	6.78E-08	6.07E-07
CNAG_06973	112.8975	1.638118	0.27825	5.887225	3.93E-09	4.44E-08
CNAG_07499	2181.588	0.272548	0.12966	2.10202	0.035552	0.072789
CNAG_06963	5671.707	-0.61885	0.155036	-3.99169	6.56E-05	0.0003
CNAG_06962	624.3542	1.538626	0.180623	8.518428	1.62E-17	5.74E-16
CNAG_03083	349.7504	1.429386	0.215452	6.634367	3.26E-11	5.17E-10
CNAG_03082	101.5333	1.042358	0.321739	3.239758	0.001196	0.003888
CNAG_03081	2926.184	-0.43818	0.105539	-4.15186	3.30E-05	0.000162
CNAG_03080	7403.984	-0.56124	0.109074	-5.14548	2.67E-07	2.06E-06
CNAG_03078	3399.48	0.559177	0.142548	3.922733	8.76E-05	0.000389
CNAG_03076	253.1973	1.078713	0.285632	3.776578	0.000159	0.000659
CNAG_12217	652.5695	0.588814	0.229332	2.567523	0.010243	0.025136
CNAG_03072	48691.77	-0.23356	0.102832	-2.27125	0.023132	0.05058
CNAG_12218	143.8114	0.642828	0.258614	2.485667	0.012931	0.030662
CNAG_03068	1199.001	1.413355	0.226582	6.237726	4.44E-10	5.91E-09
CNAG_03067	406.3606	-0.32683	0.157886	-2.07004	0.038449	0.077739
CNAG_07502	6302.66	-0.71851	0.117261	-6.12744	8.93E-10	1.14E-08

CNAG_03065	5639.071	-0.72263	0.182698	-3.95534	7.64E-05	0.000344
CNAG_03064	1473.885	-1.16134	0.213425	-5.44143	5.29E-08	4.83E-07
CNAG_03060	960.9781	1.339665	0.25745	5.203595	1.95E-07	1.57E-06
CNAG_03058	24428.23	1.094	0.271527	4.029065	5.60E-05	0.00026
CNAG_03057	177.6324	-1.21496	0.39861	-3.04799	0.002304	0.006926
CNAG_12220	9.802576	1.323062	0.660114	2.004293	0.045039	0.088869
CNAG_03055	1425.361	-0.73279	0.231978	-3.15889	0.001584	0.004974
CNAG_03054	233.6019	1.398166	0.222188	6.292723	3.12E-10	4.30E-09
CNAG_03053	26070.47	-1.05087	0.268478	-3.91417	9.07E-05	0.000401
CNAG_03051	983.3837	-0.33514	0.149976	-2.23465	0.02544	0.054635
CNAG_03049	3103.495	-0.61181	0.259636	-2.35643	0.018452	0.041593
CNAG_03048	2097.945	-0.63097	0.188744	-3.34301	0.000829	0.002802
CNAG_12222	49.90305	-1.83476	0.643146	-2.85279	0.004334	0.011961
CNAG_03046	1568.243	-2.33915	0.438184	-5.33828	9.38E-08	8.11E-07
CNAG_03044	364.4503	-0.85156	0.276764	-3.07686	0.002092	0.006364
CNAG_03040	5387.601	1.708951	0.156711	10.90514	1.09E-27	8.57E-26
CNAG_03039	1876.44	0.698014	0.166086	4.202741	2.64E-05	0.000133
CNAG_03037	1122.099	-0.95721	0.157698	-6.06992	1.28E-09	1.59E-08
CNAG_03036	1187.142	0.498125	0.150081	3.319041	0.000903	0.003022
CNAG_03034	428.1564	-0.43409	0.187304	-2.31759	0.020472	0.045451
CNAG_03033	586.7091	-0.80763	0.198636	-4.0659	4.78E-05	0.000225
CNAG_03032	1629.896	0.350115	0.134881	2.595725	0.009439	0.023402
CNAG_03031	668.8494	0.281958	0.143131	1.969924	0.048847	0.095272
CNAG_07505	33.07265	1.311276	0.351663	3.728784	0.000192	0.000782
CNAG_03027	3246.029	-0.5992	0.112725	-5.3156	1.06E-07	9.07E-07
CNAG_03026	1091.544	0.439727	0.133199	3.301277	0.000962	0.003209
CNAG_03024	2087.577	1.028507	0.300072	3.427527	0.000609	0.002151
CNAG_03022	835.318	-0.46511	0.156475	-2.97244	0.002954	0.00858
CNAG_03021	60.36728	1.503802	0.292661	5.138381	2.77E-07	2.14E-06
CNAG_03018	1182.563	-0.62045	0.218448	-2.84027	0.004508	0.012385
CNAG_03017	1645.709	-0.50749	0.158613	-3.19953	0.001377	0.004391
CNAG_03015	20738.36	-1.05859	0.254382	-4.16141	3.16E-05	0.000156
CNAG_03012	46842.82	1.281211	0.21621	5.925763	3.11E-09	3.61E-08
CNAG_12228	23.42699	1.464692	0.444066	3.298362	0.000973	0.003235
CNAG_12229	22.24302	1.605134	0.521584	3.07742	0.002088	0.006357
CNAG_03011	887.7272	0.826896	0.187818	4.402646	1.07E-05	5.92E-05
CNAG_03009	616.5646	-0.75688	0.191181	-3.95898	7.53E-05	0.000339
CNAG_03008	731.7684	-0.32081	0.147692	-2.17214	0.029845	0.062573
CNAG_03007	5558.311	2.819252	0.17687	15.93971	3.36E-57	1.11E-54
CNAG_12230	116.0084	1.302608	0.304647	4.2758	1.90E-05	9.93E-05
CNAG_03004	1729.117	-0.3749	0.1782	-2.10379	0.035397	0.072509
CNAG_03002	1850.748	1.647615	0.353737	4.657741	3.20E-06	2.01E-05
CNAG_03001	2767.415	-0.68715	0.122125	-5.62663	1.84E-08	1.84E-07
CNAG_03000	40961.08	-0.95177	0.257677	-3.69365	0.000221	0.000883
CNAG_02999	716.3345	-0.51164	0.182489	-2.8037	0.005052	0.013696
CNAG_02996	716.2926	1.588712	0.167074	9.509031	1.92E-21	1.01E-19
CNAG_02994	10030.43	0.289191	0.146456	1.974594	0.048314	0.094418

CNAG_02993	1611.704	0.433491	0.122171	3.548229	0.000388	0.001447
CNAG_02990	5390.63	1.391225	0.213216	6.524953	6.80E-11	1.03E-09
CNAG_02989	2440.342	0.901569	0.159148	5.664975	1.47E-08	1.50E-07
CNAG_02987	3.438367	3.223536	1.431188	2.25235	0.0243	0.052627
CNAG_02986	3251.2	0.786755	0.176313	4.462271	8.11E-06	4.62E-05
CNAG_02984	61.01366	-0.93326	0.323582	-2.88416	0.003925	0.010923
CNAG_02983	2043.821	-0.68253	0.133114	-5.1274	2.94E-07	2.26E-06
CNAG_02982	1963.28	-0.88615	0.150929	-5.87127	4.32E-09	4.84E-08
CNAG_02981	3952.885	-0.30004	0.136019	-2.20586	0.027393	0.058185
CNAG_02980	722.1652	0.351431	0.152993	2.297043	0.021616	0.047619
CNAG_02979	470.9472	0.880427	0.170397	5.166908	2.38E-07	1.87E-06
CNAG_02977	3418.834	0.472933	0.216977	2.179651	0.029283	0.061557
CNAG_02976	1534.599	0.316176	0.148554	2.128367	0.033307	0.06867
CNAG_02973	992.6873	-0.2965	0.142731	-2.07736	0.037769	0.076695
CNAG_02968	516.1453	0.725926	0.174891	4.150735	3.31E-05	0.000163
CNAG_02967	2095.615	0.859091	0.185795	4.623874	3.77E-06	2.33E-05
CNAG_02966	299.0179	1.660056	0.231466	7.171912	7.40E-13	1.57E-11
CNAG_02965	659.9125	0.313553	0.122455	2.56056	0.01045	0.02559
CNAG_02964	687.7168	0.543421	0.146757	3.702859	0.000213	0.000854
CNAG_02963	628.3774	-1.33469	0.191639	-6.96461	3.29E-12	6.17E-11
CNAG_02962	1861.789	-0.56848	0.215968	-2.63224	0.008482	0.021334
CNAG_02961	2465.175	-0.94426	0.296638	-3.18321	0.001457	0.004607
CNAG_02959	5528.795	0.586771	0.109736	5.3471	8.94E-08	7.78E-07
CNAG_02958	3161.113	0.528239	0.163192	3.236919	0.001208	0.003922
CNAG_07508	648.8403	0.602899	0.143801	4.192581	2.76E-05	0.000138
CNAG_07510	4426.359	-0.62523	0.120258	-5.19911	2.00E-07	1.61E-06
CNAG_02955	622.3308	-0.49667	0.232747	-2.13393	0.032848	0.067919
CNAG_02953	2926.022	0.850823	0.250071	3.402328	0.000668	0.002331
CNAG_02951	619.7492	-0.60607	0.192938	-3.14124	0.001682	0.00524
CNAG_07511	4716.698	-0.58644	0.141396	-4.14751	3.36E-05	0.000165
CNAG_07512	1080.285	1.715347	0.221566	7.741918	9.79E-15	2.59E-13
CNAG_02944	539.2676	0.736906	0.253432	2.907705	0.003641	0.01027
CNAG_02943	19752.92	1.218724	0.150014	8.124094	4.51E-16	1.42E-14
CNAG_02942	2970.822	2.70153	0.201064	13.43618	3.71E-41	6.55E-39
CNAG_02941	1727.092	0.681619	0.197027	3.459522	0.000541	0.001945
CNAG_02940	1392.038	0.580565	0.248719	2.334216	0.019584	0.043787
CNAG_02938	7136.613	-0.66556	0.17783	-3.74268	0.000182	0.000746
CNAG_02935	2430.445	-0.90926	0.227087	-4.00403	6.23E-05	0.000285
CNAG_02934	528.1121	1.461875	0.167717	8.716326	2.87E-18	1.10E-16
CNAG_02931	461.4004	-0.70109	0.177336	-3.95344	7.70E-05	0.000346
CNAG_02928	42011.42	-0.8461	0.184622	-4.58288	4.59E-06	2.78E-05
CNAG_02925	8195.317	0.426778	0.150589	2.834062	0.004596	0.012584
CNAG_02923	2637.019	-0.68809	0.24181	-2.84559	0.004433	0.012203
CNAG_02922	2054.061	-0.52363	0.172438	-3.03664	0.002392	0.007154
CNAG_02921	3337.009	0.277703	0.115682	2.400568	0.01637	0.037539
CNAG_02920	1040.894	-0.49414	0.167583	-2.94865	0.003192	0.009139
CNAG_02918	7935.077	-1.04978	0.233671	-4.49257	7.04E-06	4.08E-05

CNAG_02917	2514.823	-0.75889	0.323363	-2.34687	0.018932	0.042483
CNAG_02914	958.0922	-0.91014	0.190976	-4.76574	1.88E-06	1.24E-05
CNAG_02913	800.5761	-0.36742	0.127842	-2.87404	0.004053	0.011247
CNAG_02910	857.84	-0.44631	0.189264	-2.35812	0.018368	0.041428
CNAG_02907	893.4517	-0.43091	0.139493	-3.08914	0.002007	0.006128
CNAG_02904	437.927	0.747806	0.174878	4.276155	1.90E-05	9.92E-05
CNAG_02901	2394.629	-0.46017	0.170341	-2.70147	0.006903	0.017908
CNAG_02900	224.1865	1.370075	0.248617	5.510779	3.57E-08	3.42E-07
CNAG_12243	50.2755	1.931336	0.368142	5.246178	1.55E-07	1.28E-06
CNAG_02899	46.36448	2.224405	0.420076	5.295242	1.19E-07	1.00E-06
CNAG_02898	2844.45	-0.47887	0.190501	-2.51375	0.011945	0.028616
CNAG_02896	4598.41	-0.85324	0.170758	-4.99682	5.83E-07	4.24E-06
CNAG_02895	2976.394	1.080475	0.16688	6.474566	9.51E-11	1.42E-09
CNAG_02893	337.2322	-1.20313	0.173527	-6.93341	4.11E-12	7.61E-11
CNAG_02892	566.043	0.781442	0.14799	5.280364	1.29E-07	1.08E-06
CNAG_02890	1525.567	-0.48105	0.143834	-3.3445	0.000824	0.002791
CNAG_02888	328.351	0.630603	0.217794	2.89541	0.003787	0.010602
CNAG_02886	4091.228	0.60445	0.204823	2.951089	0.003167	0.009074
CNAG_02885	5783.469	-0.56761	0.170246	-3.33406	0.000856	0.002881
CNAG_02883	1127.274	0.357835	0.182426	1.961536	0.049816	0.096925
CNAG_12244	40.97456	1.248116	0.346149	3.605717	0.000311	0.001197
CNAG_02880	18795.32	-0.70256	0.199525	-3.52117	0.00043	0.001581
CNAG_02879	1880.945	-0.6077	0.269045	-2.25872	0.023901	0.051944
CNAG_02878	1472.194	-0.44967	0.133028	-3.38022	0.000724	0.002498
CNAG_02877	4433.044	2.614918	0.272725	9.588115	8.97E-22	4.92E-20
CNAG_02874	1336.503	0.464528	0.186362	2.49261	0.012681	0.030159
CNAG_02871	611.3273	0.295727	0.134366	2.200904	0.027743	0.058817
CNAG_02870	1946.692	-0.94142	0.170278	-5.52872	3.23E-08	3.13E-07
CNAG_02866	1419.547	-0.49661	0.150879	-3.29147	0.000997	0.003308
CNAG_02865	127.3472	0.811173	0.253537	3.199424	0.001377	0.004391
CNAG_02864	346.432	2.635024	0.203204	12.96737	1.87E-38	3.04E-36
CNAG_12250	1216.835	-0.90295	0.210489	-4.28977	1.79E-05	9.40E-05
CNAG_02862	409.317	0.806033	0.152325	5.291527	1.21E-07	1.02E-06
CNAG_02860	9916.991	0.425236	0.144993	2.932812	0.003359	0.009574
CNAG_02859	727.1346	0.583329	0.142536	4.092493	4.27E-05	0.000204
CNAG_02858	19972.4	-0.83452	0.15005	-5.56159	2.67E-08	2.63E-07
CNAG_02857	3081.084	0.76243	0.231019	3.300293	0.000966	0.003218
CNAG_07515	200.7903	-2.16919	0.421309	-5.1487	2.62E-07	2.04E-06
CNAG_02854	1837.09	-0.65423	0.141357	-4.62819	3.69E-06	2.29E-05
CNAG_02853	5309.001	-0.57184	0.117423	-4.86991	1.12E-06	7.63E-06
CNAG_02851	1241.125	-0.39245	0.144529	-2.71538	0.00662	0.017281
CNAG_02850	9688.018	-0.68942	0.221123	-3.11779	0.001822	0.005605
CNAG_02849	37.74201	-3.58482	0.664649	-5.39355	6.91E-08	6.14E-07
CNAG_02848	333.5999	0.672061	0.182574	3.681031	0.000232	0.000922
CNAG_02847	508.7487	-0.43438	0.157453	-2.75881	0.005801	0.015448
CNAG_12259	105.873	0.810698	0.290456	2.791125	0.005253	0.014177
CNAG_02843	9417.613	0.77698	0.130501	5.953833	2.62E-09	3.08E-08

CNAG_02842	2762.473	-0.79329	0.202097	-3.92527	8.66E-05	0.000385
CNAG_02841	1689.185	0.540147	0.149871	3.604072	0.000313	0.001202
CNAG_02840	1524.234	1.781204	0.19603	9.086384	1.02E-19	4.57E-18
CNAG_02839	1292.676	4.07386	0.307047	13.26786	3.56E-40	6.01E-38
CNAG_07517	1057.664	-0.41396	0.161801	-2.55844	0.010514	0.025714
CNAG_07518	675.2958	-0.43833	0.188141	-2.32979	0.019817	0.044207
CNAG_07519	3089.786	0.270474	0.126301	2.141509	0.032233	0.066891
CNAG_12261	33.39499	1.087109	0.361948	3.003498	0.002669	0.007875
CNAG_02834	1522.529	0.981315	0.181776	5.398474	6.72E-08	6.02E-07
CNAG_02833	2360.422	-1.05418	0.236997	-4.44807	8.66E-06	4.90E-05
CNAG_07520	150.6333	0.725215	0.212335	3.415427	0.000637	0.002241
CNAG_02830	4577.475	-0.68442	0.152148	-4.49837	6.85E-06	3.98E-05
CNAG_02828	144.5676	1.503434	0.327641	4.588659	4.46E-06	2.72E-05
CNAG_02826	1069.439	-0.84443	0.158845	-5.31605	1.06E-07	9.06E-07
CNAG_02825	5241.294	-1.0474	0.103076	-10.1614	2.95E-24	1.98E-22
CNAG_07521	342.4462	0.803617	0.201385	3.990445	6.59E-05	0.000301
CNAG_02824	1227.826	-0.32949	0.140819	-2.33981	0.019293	0.043209
CNAG_12262	26.67334	1.102478	0.433404	2.543764	0.010967	0.026632
CNAG_02822	4159.255	0.622311	0.286689	2.17068	0.029955	0.062769
CNAG_02821	319.5758	-0.93665	0.243079	-3.85326	0.000117	0.0005
CNAG_02819	861.5538	-0.248	0.119224	-2.08009	0.037517	0.076282
CNAG_02818	2319.438	-0.48239	0.157697	-3.05897	0.002221	0.00671
CNAG_02815	12755.86	2.236638	0.159737	14.00199	1.52E-44	3.17E-42
CNAG_02814	11553.09	0.244998	0.112585	2.176111	0.029547	0.062029
CNAG_02813	1433.249	-0.69106	0.190788	-3.62215	0.000292	0.001132
CNAG_02812	4538.788	-1.0625	0.179965	-5.90389	3.55E-09	4.06E-08
CNAG_02811	10808.63	-1.09959	0.316346	-3.47591	0.000509	0.001842
CNAG_02810	6882.128	-0.4664	0.186803	-2.49676	0.012533	0.029844
CNAG_02809	1596.105	-0.50212	0.151138	-3.32222	0.000893	0.002995
CNAG_07522	2645.683	1.038473	0.165415	6.277982	3.43E-10	4.67E-09
CNAG_02806	3301.093	0.353	0.128726	2.742266	0.006102	0.016124
CNAG_02802	144.9544	-1.01378	0.199521	-5.08107	3.75E-07	2.83E-06
CNAG_12267	27.31584	0.756386	0.371787	2.034461	0.041905	0.083579
CNAG_02799	1076.301	-0.59826	0.15751	-3.79823	0.000146	0.000611
CNAG_02798	1204.724	-0.48604	0.216274	-2.24733	0.024619	0.053201
CNAG_02796	2107.179	-0.44324	0.120661	-3.67341	0.000239	0.000947
CNAG_02794	3515.355	-0.4333	0.160452	-2.7005	0.006923	0.017953
CNAG_02793	1854.851	-0.38782	0.117524	-3.29994	0.000967	0.003221
CNAG_02791	512.6711	-0.40682	0.173604	-2.34338	0.01911	0.042834
CNAG_12268	28.50297	-3.23055	0.566705	-5.70059	1.19E-08	1.24E-07
CNAG_02790	2303.748	-0.53802	0.143023	-3.76181	0.000169	0.000697
CNAG_02788	1134.89	-0.89751	0.190271	-4.71699	2.39E-06	1.55E-05
CNAG_02785	1044.882	-0.45412	0.228942	-1.98356	0.047305	0.092743
CNAG_02782	1298.479	-0.58767	0.139999	-4.19768	2.70E-05	0.000135
CNAG_02780	2659.691	-0.39487	0.111469	-3.54243	0.000396	0.001473
CNAG_02777	3551.266	0.835662	0.248225	3.36655	0.000761	0.002608
CNAG_07523	332.6356	-0.47187	0.221668	-2.12872	0.033278	0.068628

CNAG_02775	1518.525	-0.44517	0.18929	-2.35177	0.018684	0.041999
CNAG_02771	1591.653	0.877328	0.256552	3.419693	0.000627	0.002209
CNAG_02769	1737.841	-0.35265	0.12955	-2.72208	0.006487	0.01699
CNAG_02768	366.2413	2.273503	0.217399	10.45776	1.35E-25	9.49E-24
CNAG_02767	594.1038	-0.41197	0.153215	-2.68887	0.00717	0.018496
CNAG_02766	648.5144	-0.65568	0.138541	-4.73275	2.22E-06	1.44E-05
CNAG_02764	344.7261	0.366219	0.152161	2.40678	0.016094	0.037014
CNAG_02763	4481.056	-0.60795	0.169591	-3.58479	0.000337	0.001281
CNAG_02762	11855.41	-0.78576	0.264886	-2.96642	0.003013	0.008715
CNAG_12273	9.274607	1.583934	0.778835	2.033722	0.04198	0.083673
CNAG_02757	948.4909	0.427719	0.148147	2.887133	0.003888	0.010839
CNAG_02755	1281.802	-0.48042	0.161705	-2.97095	0.002969	0.008616
CNAG_02754	48150.09	-1.1077	0.327737	-3.37985	0.000725	0.002498
CNAG_02752	3543.645	-0.69332	0.209267	-3.31307	0.000923	0.003083
CNAG_02751	4979.727	2.555496	0.159702	16.00165	1.24E-57	4.70E-55
CNAG_02747	764.1172	0.258639	0.124954	2.069879	0.038464	0.077749
CNAG_02741	912.987	-0.34079	0.138727	-2.45658	0.014027	0.032907
CNAG_02740	427.8542	-0.73053	0.160225	-4.55942	5.13E-06	3.09E-05
CNAG_02739	1529.54	-0.6611	0.136056	-4.85904	1.18E-06	8.02E-06
CNAG_02735	384.2393	2.919442	0.208893	13.97577	2.19E-44	4.46E-42
CNAG_02733	54.08943	-0.72708	0.332048	-2.18969	0.028547	0.060232
CNAG_02725	5112.248	0.809219	0.145344	5.567628	2.58E-08	2.55E-07
CNAG_02724	628.5831	-1.07954	0.26061	-4.14235	3.44E-05	0.000168
CNAG_12276	2.761079	-2.93572	1.440756	-2.03763	0.041587	0.0831
CNAG_12277	16.56335	1.24459	0.606068	2.053548	0.040019	0.080383
CNAG_02721	1541.053	-0.78352	0.282759	-2.77099	0.005589	0.014987
CNAG_02720	2162.974	-0.67761	0.151267	-4.47959	7.48E-06	4.31E-05
CNAG_02719	332.2698	0.637503	0.202777	3.143862	0.001667	0.005204
CNAG_07969	123.5481	2.79704	0.374424	7.470242	8.00E-14	1.89E-12
CNAG_02714	29665.88	-0.87941	0.281812	-3.12057	0.001805	0.005564
CNAG_02712	2058.773	-0.49856	0.149341	-3.33844	0.000843	0.002846
CNAG_02709	659.4875	-0.84025	0.239721	-3.50513	0.000456	0.001665
CNAG_02708	923.5684	0.609415	0.125944	4.838772	1.31E-06	8.81E-06
CNAG_02705	121.1736	-0.53623	0.221928	-2.41622	0.015683	0.036225
CNAG_07525	7.771584	1.87882	0.776399	2.419917	0.015524	0.035901
CNAG_02704	2086.057	-0.55497	0.210312	-2.63877	0.008321	0.02097
CNAG_02702	2701.431	-0.43563	0.214209	-2.03368	0.041984	0.083673
CNAG_02701	9068.686	4.480936	0.209123	21.42732	7.43E-102	9.85E-99
CNAG_12282	52.99754	3.067565	0.372864	8.227036	1.92E-16	6.20E-15
CNAG_02700	2650.068	0.383685	0.192998	1.988026	0.046809	0.091951
CNAG_02698	1492.459	-0.40285	0.200307	-2.01116	0.044308	0.087736
CNAG_12285	370.7742	1.13832	0.318649	3.572331	0.000354	0.001336
CNAG_02692	9327.339	2.858336	0.243902	11.7192	1.02E-31	1.02E-29
CNAG_02691	1096.495	1.402451	0.28684	4.889308	1.01E-06	6.98E-06
CNAG_02690	872.3762	0.864289	0.131136	6.59079	4.37E-11	6.77E-10
CNAG_07527	55.88064	1.620577	0.326313	4.966322	6.82E-07	4.91E-06
CNAG_02687	1943.468	-1.0462	0.13811	-7.57507	3.59E-14	8.86E-13

CNAG_02686	5691.914	-0.45019	0.129808	-3.46815	0.000524	0.001889
CNAG_02685	2080.854	3.543326	0.160846	22.02925	1.51E-107	2.40E-104
CNAG_12289	37.21106	3.870756	0.501398	7.719924	1.16E-14	3.03E-13
CNAG_02684	2136.499	1.521627	0.312113	4.875241	1.09E-06	7.44E-06
CNAG_02683	1731.62	0.352129	0.114605	3.072539	0.002122	0.006444
CNAG_02682	1785.189	0.579136	0.280134	2.067353	0.038701	0.078129
CNAG_02681	562.8935	0.816917	0.14899	5.483031	4.18E-08	3.89E-07
CNAG_02679	749.3242	0.452503	0.144956	3.121662	0.001798	0.005549
CNAG_02678	758.2865	0.439977	0.197145	2.231742	0.025632	0.055002
CNAG_02677	3714.976	-0.40358	0.156998	-2.57061	0.010152	0.024928
CNAG_02675	2912.36	-0.60838	0.300852	-2.02219	0.043157	0.085817
CNAG_02674	949.4581	0.431774	0.172581	2.501858	0.012354	0.02948
CNAG_02673	8201.156	1.195332	0.155424	7.690778	1.46E-14	3.75E-13
CNAG_02672	3094.45	-0.63072	0.153654	-4.1048	4.05E-05	0.000195
CNAG_02670	1164.37	-0.56482	0.123734	-4.56483	5.00E-06	3.01E-05
CNAG_12290	32.13826	0.988374	0.372521	2.653205	0.007973	0.020202
CNAG_02667	636.5693	0.637368	0.155875	4.088966	4.33E-05	0.000207
CNAG_02666	770.0752	0.411991	0.133896	3.076943	0.002091	0.006364
CNAG_02664	3768.098	-0.70881	0.106574	-6.65087	2.91E-11	4.66E-10
CNAG_02663	1571.456	1.208424	0.138087	8.751152	2.11E-18	8.23E-17
CNAG_12292	31.4581	1.049372	0.365481	2.871207	0.004089	0.011341
CNAG_02662	1506.387	0.487317	0.162242	3.003647	0.002668	0.007874
CNAG_02661	407.7249	0.487055	0.178806	2.723925	0.006451	0.016918
CNAG_12293	180.8233	0.629877	0.179503	3.509007	0.00045	0.001645
CNAG_12294	226.1881	0.688996	0.175152	3.933699	8.36E-05	0.000374
CNAG_12295	79.28726	0.987802	0.378063	2.612796	0.00898	0.022377
CNAG_12298	278.0393	0.965142	0.251343	3.839939	0.000123	0.000524
CNAG_02657	7491.174	-0.71535	0.196471	-3.64096	0.000272	0.001061
CNAG_02656	2852.936	-0.44553	0.192216	-2.31786	0.020457	0.045431
CNAG_02655	3118.244	0.807182	0.154089	5.238404	1.62E-07	1.33E-06
CNAG_07529	1787.853	-0.62014	0.182926	-3.39008	0.000699	0.002419
CNAG_07531	966.7218	-1.08115	0.235768	-4.58566	4.53E-06	2.75E-05
CNAG_07532	1019.214	-0.47467	0.17799	-2.66684	0.007657	0.0195
CNAG_07533	138.9516	0.728428	0.350347	2.07916	0.037603	0.076411
CNAG_07535	900.8105	-0.89652	0.22188	-4.04056	5.33E-05	0.000248
CNAG_07536	708.9059	-0.57991	0.158997	-3.64731	0.000265	0.001037
CNAG_07537	535.0589	-0.56105	0.159396	-3.51984	0.000432	0.001586
CNAG_07538	3589.722	3.017498	0.14365	21.00593	5.79E-98	5.75E-95
CNAG_07162	408.0014	-0.63573	0.174183	-3.64978	0.000262	0.001028
CNAG_07540	672.3561	-0.73433	0.242275	-3.03099	0.002437	0.00727
CNAG_07541	2944.711	1.230695	0.174937	7.035067	1.99E-12	3.96E-11
CNAG_07542	247.8117	0.91386	0.169637	5.387137	7.16E-08	6.34E-07
CNAG_07544	38.3238	2.096287	0.444319	4.717975	2.38E-06	1.54E-05
CNAG_07545	791.0112	-0.64793	0.234192	-2.76665	0.005664	0.015142
CNAG_07546	1151.195	-0.39619	0.145483	-2.72328	0.006464	0.016945
CNAG_07547	1796.979	-0.57821	0.172165	-3.35844	0.000784	0.002675
CNAG_07548	906.9124	0.50091	0.126517	3.959234	7.52E-05	0.000339

CNAG_07549	144.7439	-0.99605	0.244071	-4.08098	4.48E-05	0.000213
CNAG_12301	7.134677	-2.39053	0.890897	-2.68329	0.00729	0.018735
CNAG_07551	3282.074	1.381997	0.279963	4.936361	7.96E-07	5.63E-06
CNAG_07552	1754.346	1.328016	0.326471	4.067789	4.75E-05	0.000223
CNAG_07554	6254.372	-0.88964	0.114386	-7.77755	7.39E-15	1.99E-13
CNAG_07555	503.2376	0.854255	0.177589	4.810296	1.51E-06	1.01E-05
CNAG_07558	22052.64	0.442109	0.18311	2.414451	0.015759	0.036372
CNAG_07559	4161.621	0.660292	0.204511	3.228639	0.001244	0.004024
CNAG_07560	689.7071	-0.84942	0.147933	-5.74194	9.36E-09	9.85E-08
CNAG_07561	52251.76	-0.4105	0.137193	-2.99214	0.00277	0.008129
CNAG_07563	965.6417	-0.65312	0.136803	-4.77418	1.80E-06	1.19E-05
CNAG_07564	773.3833	0.714848	0.187563	3.81124	0.000138	0.000582
CNAG_07566	626.1745	0.320626	0.137025	2.339914	0.019288	0.043209
CNAG_07567	1206.716	-0.82187	0.18889	-4.35107	1.35E-05	7.37E-05
CNAG_07572	1117.166	-1.08157	0.238105	-4.54243	5.56E-06	3.30E-05
CNAG_02609	1531.094	-0.37795	0.168576	-2.24199	0.024962	0.053743
CNAG_07573	1401.882	0.939287	0.172109	5.457529	4.83E-08	4.44E-07
CNAG_02606	771.1443	0.685793	0.187611	3.655391	0.000257	0.001009
CNAG_02605	1236.565	0.51679	0.137683	3.753477	0.000174	0.000718
CNAG_02603	189.7319	-1.54576	0.233483	-6.62043	3.58E-11	5.62E-10
CNAG_02602	25.20495	1.365423	0.435925	3.132245	0.001735	0.005378
CNAG_02600	265.6228	-0.90032	0.163952	-5.49136	3.99E-08	3.75E-07
CNAG_02599	27.91824	0.861957	0.365377	2.359092	0.01832	0.041348
CNAG_02598	95.35641	0.605187	0.227985	2.6545	0.007943	0.020133
CNAG_02597	1757.734	-0.59188	0.180756	-3.27448	0.001059	0.003479
CNAG_02593	1393.024	0.881211	0.156302	5.637862	1.72E-08	1.73E-07
CNAG_02592	379.2939	-0.95349	0.384619	-2.47906	0.013173	0.031125
CNAG_12305	29.77101	1.51929	0.453169	3.352594	0.000801	0.002727
CNAG_02591	571.8709	1.919582	0.333514	5.755621	8.63E-09	9.13E-08
CNAG_02590	454.4151	0.511274	0.15861	3.223458	0.001267	0.004093
CNAG_02589	646.5203	0.681153	0.159895	4.260014	2.04E-05	0.000105
CNAG_02588	264.6722	-0.86995	0.250837	-3.46819	0.000524	0.001889
CNAG_02587	711.5187	-0.58383	0.231138	-2.52591	0.01154	0.027787
CNAG_02585	4623.952	1.12089	0.135838	8.251654	1.56E-16	5.13E-15
CNAG_02584	574.4212	0.83887	0.158708	5.285612	1.25E-07	1.05E-06
CNAG_02580	1278.262	-0.42028	0.17265	-2.43428	0.014921	0.034699
CNAG_02578	886.571	-0.58788	0.181524	-3.23859	0.001201	0.003902
CNAG_02577	3602.693	1.127075	0.22536	5.001217	5.70E-07	4.16E-06
CNAG_02576	1345.297	0.631881	0.166514	3.794766	0.000148	0.000618
CNAG_02575	3968.368	0.347584	0.175937	1.975619	0.048198	0.094214
CNAG_07574	124.0243	2.57994	0.288849	8.931806	4.19E-19	1.76E-17
CNAG_12313	104.3997	1.779641	0.266153	6.686531	2.29E-11	3.73E-10
CNAG_12314	68.29004	1.26507	0.419951	3.012425	0.002592	0.007676
CNAG_02569	835.2963	-0.362	0.118149	-3.0639	0.002185	0.006613
CNAG_02568	5323.032	0.579336	0.170876	3.390384	0.000698	0.002418
CNAG_02566	1610.458	0.559629	0.253193	2.210282	0.027086	0.057654
CNAG_02565	3103.754	-1.39262	0.203613	-6.83953	7.95E-12	1.42E-10

CNAG_02564	802.6593	-0.86267	0.20714	-4.16465	3.12E-05	0.000154
CNAG_12315	159.6614	2.96547	0.341744	8.677472	4.05E-18	1.53E-16
CNAG_07972	528.0212	1.470814	0.192827	7.627648	2.39E-14	6.03E-13
CNAG_06879	1191.895	0.810503	0.1906	4.252365	2.12E-05	0.000109
CNAG_06880	1940.25	0.622398	0.215254	2.891455	0.003835	0.010714
CNAG_06881	2482.756	0.545726	0.190301	2.867703	0.004135	0.011455
CNAG_06884	745.7562	1.483689	0.184737	8.031364	9.64E-16	2.92E-14
CNAG_07579	621.5265	-0.52861	0.214758	-2.46143	0.013839	0.032504
CNAG_07580	1275.785	-0.3652	0.133372	-2.73819	0.006178	0.016303
CNAG_06890	2588.244	-0.55077	0.217177	-2.53605	0.011211	0.027127
CNAG_06891	1266.943	-0.50642	0.234466	-2.15987	0.030783	0.06425
CNAG_06897	1520.708	-0.82744	0.246444	-3.35753	0.000786	0.002682
CNAG_12320	428.7933	-1.70032	0.322482	-5.2726	1.35E-07	1.12E-06
CNAG_06899	9551.413	0.566537	0.119796	4.729171	2.25E-06	1.46E-05
CNAG_06900	20075.56	-0.37363	0.103498	-3.61004	0.000306	0.001179
CNAG_06901	679.7173	0.550008	0.128107	4.293364	1.76E-05	9.27E-05
CNAG_06902	1074.299	-1.01186	0.14858	-6.8102	9.75E-12	1.72E-10
CNAG_06905	2362.491	1.550027	0.17226	8.998164	2.30E-19	9.97E-18
CNAG_06906	9681.462	-0.65584	0.107758	-6.08627	1.16E-09	1.45E-08
CNAG_06908	15497.89	-1.11233	0.224023	-4.96524	6.86E-07	4.93E-06
CNAG_12328	127.9838	0.833942	0.232816	3.581985	0.000341	0.001292
CNAG_06913	661.8887	0.508044	0.187949	2.703093	0.00687	0.017833
CNAG_06915	66.02042	0.657691	0.282804	2.325608	0.020039	0.044666
CNAG_06916	871.0569	-0.5023	0.182326	-2.75494	0.00587	0.015606
CNAG_06917	6961.791	-1.12096	0.307733	-3.64263	0.00027	0.001056
CNAG_06918	102.4588	1.687641	0.248186	6.799901	1.05E-11	1.83E-10
CNAG_06922	2177.399	0.589613	0.213686	2.75925	0.005793	0.015432
CNAG_06923	16007.53	-0.97013	0.385923	-2.5138	0.011944	0.028616
CNAG_06924	1157.415	1.014191	0.171731	5.905682	3.51E-09	4.04E-08
CNAG_06925	1023.8	-0.34885	0.1686	-2.0691	0.038537	0.077838
CNAG_06926	271.116	-0.32442	0.157804	-2.05588	0.039794	0.079992
CNAG_06927	1822.756	-0.61504	0.143637	-4.28192	1.85E-05	9.70E-05
CNAG_06930	2152.661	-1.09566	0.332757	-3.29268	0.000992	0.003298
CNAG_06932	105.3718	-0.67296	0.297985	-2.25837	0.023923	0.051965
CNAG_07583	730.3172	0.668698	0.195411	3.422012	0.000622	0.002191
CNAG_06935	552.5127	2.1694	0.271785	7.982046	1.44E-15	4.22E-14
CNAG_04934	4481.081	2.286163	0.178266	12.82443	1.20E-37	1.73E-35
CNAG_04935	989.5021	-0.43135	0.156908	-2.74904	0.005977	0.015852
CNAG_04936	2753.703	-0.72574	0.126438	-5.7399	9.47E-09	9.94E-08
CNAG_04937	307.039	-0.42486	0.183561	-2.31455	0.020638	0.045742
CNAG_04938	3291.144	2.352202	0.182531	12.88659	5.36E-38	8.34E-36
CNAG_04939	1204.022	-0.40644	0.204172	-1.99066	0.046519	0.091449
CNAG_07794	2677.834	0.885675	0.197287	4.489279	7.15E-06	4.13E-05
CNAG_07796	818.8453	-0.92876	0.172918	-5.37108	7.83E-08	6.88E-07
CNAG_04943	1832.192	1.295042	0.151647	8.539823	1.34E-17	4.86E-16
CNAG_04944	1626.321	0.894732	0.12612	7.094284	1.30E-12	2.66E-11
CNAG_04945	295.971	0.459818	0.172175	2.670649	0.00757	0.019322

CNAG_04946	257.4237	-0.4669	0.199345	-2.34216	0.019172	0.042962
CNAG_12335	90.04055	3.230261	0.306487	10.53965	5.67E-26	4.10E-24
CNAG_04948	3410.808	-0.48662	0.118448	-4.10827	3.99E-05	0.000192
CNAG_04949	1831.938	-1.07106	0.166922	-6.41658	1.39E-10	2.03E-09
CNAG_04950	3059.952	-0.59332	0.222888	-2.66194	0.007769	0.019748
CNAG_04951	5103.962	-1.52992	0.191936	-7.97097	1.57E-15	4.57E-14
CNAG_04952	2088.336	-0.42712	0.161821	-2.63948	0.008303	0.020939
CNAG_04953	11785.78	0.878678	0.128781	6.823022	8.91E-12	1.58E-10
CNAG_04957	1757.589	-0.82925	0.200891	-4.12787	3.66E-05	0.000178
CNAG_04958	3486.944	0.631946	0.265685	2.378553	0.017381	0.039482
CNAG_04961	2550.167	-0.42387	0.171615	-2.46986	0.013516	0.031813
CNAG_04962	3671.794	-0.61075	0.185662	-3.28956	0.001003	0.003329
CNAG_04963	4383.648	0.672979	0.1648	4.083613	4.43E-05	0.000211
CNAG_07797	1110.224	-1.84471	0.203223	-9.07727	1.11E-19	4.94E-18
CNAG_04968	415.7163	-0.58214	0.18548	-3.13855	0.001698	0.005276
CNAG_04969	18071.11	-0.389	0.123702	-3.14463	0.001663	0.005194
CNAG_04970	1625.676	0.851988	0.223413	3.813512	0.000137	0.000578
CNAG_07800	1289.075	0.552596	0.215779	2.56094	0.010439	0.02557
CNAG_12342	48.84211	-1.03938	0.299363	-3.47196	0.000517	0.001868
CNAG_12343	8.051266	2.1124	0.746234	2.830746	0.004644	0.012698
CNAG_04976	9543.384	-1.04769	0.233245	-4.4918	7.06E-06	4.09E-05
CNAG_04980	1254.751	-0.91552	0.230371	-3.97412	7.06E-05	0.000321
CNAG_04981	5932.724	1.321052	0.174569	7.567489	3.81E-14	9.36E-13
CNAG_04982	223.0487	-0.82245	0.241175	-3.41019	0.000649	0.002275
CNAG_04983	1362.389	-0.81644	0.215079	-3.79599	0.000147	0.000616
CNAG_04984	3118.645	0.713396	0.164257	4.34317	1.40E-05	7.60E-05
CNAG_04985	12303.31	-0.46665	0.197811	-2.35905	0.018322	0.041348
CNAG_04987	691.7229	0.879076	0.155183	5.664768	1.47E-08	1.50E-07
CNAG_12349	42.60027	1.695879	0.353768	4.793755	1.64E-06	1.09E-05
CNAG_04990	3352.517	-0.52763	0.125538	-4.20293	2.63E-05	0.000133
CNAG_12351	62.35611	-0.90803	0.43865	-2.07005	0.038448	0.077739
CNAG_04992	733.3083	0.550675	0.174937	3.14784	0.001645	0.005146
CNAG_04993	79.02306	1.131954	0.264519	4.279299	1.87E-05	9.80E-05
CNAG_04994	1055.587	-0.59618	0.198024	-3.01065	0.002607	0.007715
CNAG_07802	457.1086	1.193215	0.20029	5.957426	2.56E-09	3.03E-08
CNAG_05001	785.1959	-0.58034	0.259548	-2.23598	0.025353	0.054492
CNAG_05003	637.0037	0.697905	0.16851	4.141616	3.45E-05	0.000169
CNAG_05004	1111.84	-0.44885	0.196854	-2.28014	0.022599	0.049537
CNAG_05005	1031.464	1.544471	0.329938	4.681095	2.85E-06	1.82E-05
CNAG_05006	712.9969	-0.46634	0.173496	-2.68787	0.007191	0.018546
CNAG_05007	1309.005	-0.86562	0.163556	-5.29251	1.21E-07	1.01E-06
CNAG_05009	658.2243	1.043002	0.208723	4.997054	5.82E-07	4.24E-06
CNAG_12357	66.9697	1.210127	0.27418	4.413624	1.02E-05	5.66E-05
CNAG_05010	26.16939	1.913308	0.46503	4.114373	3.88E-05	0.000188
CNAG_05011	982.8566	-0.52655	0.198143	-2.65743	0.007874	0.019995
CNAG_05012	760.4045	0.673356	0.15483	4.348991	1.37E-05	7.43E-05
CNAG_05013	9673.587	-0.50259	0.213663	-2.35224	0.018661	0.041969

CNAG_05016	33.05743	-1.12614	0.505643	-2.22714	0.025938	0.055523
CNAG_05019	364.9416	-0.4955	0.194728	-2.54458	0.010941	0.026586
CNAG_05024	262.0588	0.93234	0.225047	4.142877	3.43E-05	0.000168
CNAG_07803	326.6997	-0.7842	0.252572	-3.10487	0.001904	0.005838
CNAG_05028	4465.034	-0.45421	0.170365	-2.66607	0.007674	0.019539
CNAG_05030	960.3277	-0.30814	0.123637	-2.49231	0.012691	0.030175
CNAG_05032	375.8386	2.220347	0.165393	13.42471	4.33E-41	7.48E-39
CNAG_05035	1192.451	-0.56384	0.174035	-3.23979	0.001196	0.003888
CNAG_05038	738.9182	1.418666	0.196799	7.208698	5.65E-13	1.21E-11
CNAG_05039	1026.051	-0.73604	0.210099	-3.5033	0.00046	0.001673
CNAG_05041	4252.486	-1.30851	0.160566	-8.14939	3.66E-16	1.16E-14
CNAG_05045	849.9884	-0.75528	0.171719	-4.39832	1.09E-05	6.03E-05
CNAG_07805	6320.459	0.732037	0.173047	4.230275	2.33E-05	0.000119
CNAG_05050	4008.081	-0.82499	0.191206	-4.31465	1.60E-05	8.49E-05
CNAG_07807	23712.03	-0.95761	0.20301	-4.71709	2.39E-06	1.55E-05
CNAG_05053	630.0395	-0.70794	0.259127	-2.73203	0.006295	0.016595
CNAG_12360	201.0564	-1.45679	0.304226	-4.78851	1.68E-06	1.11E-05
CNAG_05055	777.7433	-0.33738	0.124031	-2.72009	0.006526	0.017076
CNAG_05057	1027.387	1.076	0.207027	5.197401	2.02E-07	1.61E-06
CNAG_05059	12922.68	-1.03666	0.213836	-4.8479	1.25E-06	8.44E-06
CNAG_05060	1308.945	0.475529	0.204577	2.324452	0.020101	0.044778
CNAG_05062	1621.172	-0.39915	0.148745	-2.68345	0.007287	0.018735
CNAG_05067	51.65972	2.753318	0.396302	6.94753	3.72E-12	6.92E-11
CNAG_05069	3809.295	-1.14161	0.291622	-3.91468	9.05E-05	0.000401
CNAG_05070	6619.736	-1.11725	0.188107	-5.93943	2.86E-09	3.34E-08
CNAG_12363	26.82372	1.767828	0.440973	4.008924	6.10E-05	0.00028
CNAG_07808	3085.218	-0.29547	0.118163	-2.50053	0.012401	0.029573
CNAG_05074	2044.49	0.813686	0.245825	3.310026	0.000933	0.003115
CNAG_12365	29.00326	1.227389	0.381119	3.220486	0.00128	0.00413
CNAG_05077	2297.277	1.277823	0.149716	8.534987	1.40E-17	5.02E-16
CNAG_05079	729.1761	-3.21489	0.248525	-12.9359	2.82E-38	4.49E-36
CNAG_05084	4215.496	0.419653	0.123976	3.384938	0.000712	0.00246
CNAG_05085	1670.991	-0.71238	0.203589	-3.49909	0.000467	0.001699
CNAG_05086	982.2872	-0.91552	0.306096	-2.99095	0.002781	0.008155
CNAG_07810	10228.97	-0.35353	0.122435	-2.88752	0.003883	0.010834
CNAG_05090	3397.982	0.39743	0.112065	3.546422	0.000391	0.001455
CNAG_05091	1665.328	-1.0165	0.223525	-4.54758	5.43E-06	3.23E-05
CNAG_05092	2.115566	3.406217	1.619428	2.103345	0.035436	0.07257
CNAG_07973	276.3132	0.428826	0.19785	2.167426	0.030202	0.063205
CNAG_05095	4252.535	1.805081	0.204856	8.81146	1.24E-18	4.93E-17
CNAG_05097	2061.137	0.94513	0.203059	4.654458	3.25E-06	2.04E-05
CNAG_05101	3170.88	-0.58437	0.117853	-4.9585	7.10E-07	5.09E-06
CNAG_12367	78.91984	-0.83024	0.38698	-2.14543	0.031919	0.066307
CNAG_05102	1187.54	-0.3139	0.136472	-2.30013	0.021441	0.047298
CNAG_05103	824.5066	-0.26885	0.123317	-2.18017	0.029245	0.061508
CNAG_05105	7567.1	-0.58405	0.121437	-4.80949	1.51E-06	1.01E-05
CNAG_05107	1770.311	-0.64981	0.254782	-2.55045	0.010758	0.026182

CNAG_05109	3576.909	-0.41754	0.115873	-3.60346	0.000314	0.001204
CNAG_05110	1424.496	-0.83832	0.130262	-6.43565	1.23E-10	1.81E-09
CNAG_05111	2636.817	-0.85497	0.140887	-6.06847	1.29E-09	1.61E-08
CNAG_12368	188.6126	-1.40288	0.430432	-3.25925	0.001117	0.003651
CNAG_05114	357.8961	-0.96379	0.181471	-5.311	1.09E-07	9.28E-07
CNAG_05115	940.1698	-0.7311	0.166915	-4.38009	1.19E-05	6.53E-05
CNAG_05118	1125.551	-0.74174	0.231182	-3.20845	0.001335	0.004278
CNAG_05119	1661.656	0.590085	0.139171	4.240013	2.24E-05	0.000114
CNAG_05120	2818.373	-0.30241	0.116704	-2.59126	0.009563	0.023679
CNAG_05121	1843.74	-0.67436	0.158694	-4.24946	2.14E-05	0.00011
CNAG_05122	2476.478	-0.74404	0.112903	-6.5901	4.40E-11	6.77E-10
CNAG_05124	2553.421	-0.57553	0.129383	-4.44827	8.66E-06	4.90E-05
CNAG_05125	2182.139	-0.94833	0.162381	-5.84017	5.21E-09	5.73E-08
CNAG_07814	1008.394	-0.47662	0.13224	-3.6042	0.000313	0.001202
CNAG_05132	8417.017	-1.19048	0.261641	-4.55006	5.36E-06	3.20E-05
CNAG_05137	2734.029	0.929386	0.219698	4.230287	2.33E-05	0.000119
CNAG_05139	1588.431	0.650188	0.217223	2.993182	0.002761	0.008108
CNAG_05140	7575.076	-0.52031	0.211236	-2.46316	0.013772	0.032357
CNAG_05144	8507.47	-0.41271	0.141518	-2.91631	0.003542	0.010023
CNAG_05145	1062.671	-0.4006	0.177094	-2.2621	0.023691	0.051576
CNAG_05147	370.8285	-1.08255	0.305412	-3.54457	0.000393	0.001464
CNAG_05148	3286.341	-0.33735	0.116728	-2.89005	0.003852	0.010754
CNAG_05149	1004.987	-0.42556	0.139641	-3.04749	0.002308	0.006935
CNAG_05150	2119.976	-0.40028	0.122857	-3.25815	0.001121	0.003662
CNAG_05151	1450.83	-0.76783	0.190275	-4.03538	5.45E-05	0.000253
CNAG_05152	2903.483	0.362346	0.157961	2.293902	0.021796	0.047962
CNAG_05154	1544.633	-1.21319	0.238019	-5.09703	3.45E-07	2.62E-06
CNAG_05155	3972.795	1.371637	0.277726	4.938818	7.86E-07	5.57E-06
CNAG_05156	1411.722	0.547354	0.125295	4.368538	1.25E-05	6.85E-05
CNAG_12377	247.5288	0.935158	0.317497	2.945408	0.003225	0.009225
CNAG_05162	411.9671	-0.49729	0.241033	-2.06317	0.039097	0.078744
CNAG_05164	2428.378	-0.32012	0.128683	-2.48767	0.012858	0.030526
CNAG_12379	25.48405	1.043473	0.430949	2.421336	0.015464	0.035792
CNAG_05167	6773.914	1.771977	0.228666	7.749208	9.25E-15	2.46E-13
CNAG_05168	31.53438	1.588414	0.410243	3.871889	0.000108	0.000466
CNAG_12380	5.481053	-2.90852	1.126391	-2.58216	0.009819	0.024237
CNAG_05169	489.6052	-0.60676	0.15503	-3.91383	9.08E-05	0.000401
CNAG_05170	1069.94	0.782551	0.193026	4.054126	5.03E-05	0.000236
CNAG_05173	1158.755	0.793664	0.142958	5.551713	2.83E-08	2.76E-07
CNAG_07817	1322.096	-1.0051	0.23164	-4.33907	1.43E-05	7.72E-05
CNAG_07818	786.0447	-0.83243	0.151757	-5.48526	4.13E-08	3.85E-07
CNAG_05178	291.8705	1.500961	0.191376	7.84298	4.40E-15	1.21E-13
CNAG_05179	11609.53	-1.07292	0.156222	-6.86794	6.51E-12	1.18E-10
CNAG_05180	1075.52	0.882212	0.196978	4.478727	7.51E-06	4.32E-05
CNAG_05183	975.7988	0.479634	0.148356	3.233005	0.001225	0.003973
CNAG_05184	48.05538	2.150366	0.420414	5.11487	3.14E-07	2.40E-06
CNAG_05185	127.6718	-0.81823	0.287136	-2.84961	0.004377	0.012073

CNAG_05186	569.3169	-0.60199	0.19005	-3.16753	0.001537	0.004838
CNAG_05189	924.5358	-0.53446	0.136963	-3.90218	9.53E-05	0.000418
CNAG_05190	3034.876	-0.59414	0.11451	-5.18855	2.12E-07	1.68E-06
CNAG_05192	151.6214	0.711996	0.278775	2.554019	0.010649	0.025956
CNAG_05193	1765.219	0.629307	0.241494	2.605891	0.009164	0.022797
CNAG_05194	2672.61	-0.71762	0.228294	-3.14339	0.00167	0.00521
CNAG_05195	2946.843	0.61519	0.240428	2.55873	0.010506	0.025701
CNAG_05196	1347.815	-0.7336	0.182496	-4.0198	5.82E-05	0.000269
CNAG_05197	4504.46	-0.96942	0.263724	-3.67588	0.000237	0.000939
CNAG_05198	1166.816	0.300798	0.135698	2.216674	0.026645	0.056839
CNAG_05199	34192.58	0.624193	0.13975	4.466503	7.95E-06	4.53E-05
CNAG_05200	954.8009	-0.28704	0.118331	-2.42573	0.015278	0.035424
CNAG_05201	300.3775	0.866656	0.194573	4.454144	8.42E-06	4.78E-05
CNAG_07819	35.73355	1.174066	0.464363	2.528339	0.01146	0.027635
CNAG_05215	1985.354	0.333	0.107503	3.097591	0.001951	0.005963
CNAG_05216	2443.422	0.820534	0.227572	3.605605	0.000311	0.001197
CNAG_05217	384.3323	-0.92852	0.169764	-5.4695	4.51E-08	4.17E-07
CNAG_05218	4145.776	0.268019	0.106364	2.519822	0.011741	0.028188
CNAG_05220	177.1624	1.424295	0.226499	6.288296	3.21E-10	4.41E-09
CNAG_05221	2982.812	-1.06748	0.256527	-4.16129	3.16E-05	0.000156
CNAG_05224	189.8762	-0.63047	0.174305	-3.61705	0.000298	0.001152
CNAG_05225	1371.202	-0.76105	0.233513	-3.25915	0.001117	0.003651
CNAG_05226	2026.803	-0.58564	0.172611	-3.39285	0.000692	0.002399
CNAG_05228	1486.275	-1.2202	0.250612	-4.86887	1.12E-06	7.66E-06
CNAG_05229	1025.524	-1.51637	0.19523	-7.76713	8.03E-15	2.16E-13
CNAG_05230	623.8783	-0.46308	0.151804	-3.05054	0.002284	0.00687
CNAG_05231	1689.949	-0.58186	0.222428	-2.61595	0.008898	0.022206
CNAG_05232	62055.23	-0.91527	0.241357	-3.7922	0.000149	0.000622
CNAG_05234	2123.627	-0.54475	0.20534	-2.65293	0.00798	0.020206
CNAG_05239	450.9411	-0.70803	0.192241	-3.68307	0.00023	0.000916
CNAG_05240	2759.885	-0.61824	0.145101	-4.26073	2.04E-05	0.000105
CNAG_05243	917.8231	-0.52833	0.138056	-3.82695	0.00013	0.000549
CNAG_12387	98.91283	-0.66843	0.302746	-2.20789	0.027252	0.057942
CNAG_12388	33.372	-0.83892	0.404204	-2.07549	0.037941	0.076965
CNAG_05245	267.091	-0.7868	0.284038	-2.77004	0.005605	0.015017
CNAG_07976	44.23319	1.144745	0.300246	3.812696	0.000137	0.00058
CNAG_07822	649.6818	-0.33117	0.144394	-2.29348	0.02182	0.047989
CNAG_12389	32.79039	0.93945	0.378612	2.481301	0.01309	0.030976
CNAG_05252	3053.545	0.592845	0.167262	3.544414	0.000393	0.001464
CNAG_05253	451.4195	0.395084	0.167848	2.353817	0.018582	0.041819
CNAG_05256	1441.332	1.18759	0.149472	7.945253	1.94E-15	5.58E-14
CNAG_05258	429.5345	1.616381	0.279305	5.787146	7.16E-09	7.68E-08
CNAG_07823	164.9203	1.000216	0.202038	4.950639	7.40E-07	5.28E-06
CNAG_05259	76.91036	0.605621	0.248466	2.437445	0.014791	0.034467
CNAG_05260	5551.717	0.339793	0.169231	2.007872	0.044657	0.088226
CNAG_05261	1481.234	-0.30682	0.135624	-2.26227	0.023681	0.051566
CNAG_05264	519.7254	0.826868	0.205898	4.015902	5.92E-05	0.000272

CNAG_12392	2273.668	3.490258	0.28777	12.12863	7.45E-34	9.11E-32
CNAG_05267	2470.888	-1.26844	0.230975	-5.49168	3.98E-08	3.75E-07
CNAG_05268	51.14231	-1.30039	0.414777	-3.13514	0.001718	0.005332
CNAG_05269	7218.402	0.486494	0.208563	2.332604	0.019669	0.043951
CNAG_05270	2074.312	-0.41793	0.171167	-2.44165	0.01462	0.034109
CNAG_05273	1731.8	-0.80159	0.262298	-3.05601	0.002243	0.006764
CNAG_05274	839.8903	-0.76856	0.207557	-3.7029	0.000213	0.000854
CNAG_05276	1406.115	-0.47816	0.135942	-3.51742	0.000436	0.001597
CNAG_05281	154.7907	-0.56981	0.236487	-2.40948	0.015975	0.036783
CNAG_05283	581.5332	-0.96558	0.128635	-7.50637	6.08E-14	1.45E-12
CNAG_05284	555.8987	-0.54224	0.16446	-3.29712	0.000977	0.003248
CNAG_05286	574.7402	0.432514	0.211571	2.044299	0.040924	0.081999
CNAG_05288	1104.95	1.112602	0.158999	6.997549	2.60E-12	5.06E-11
CNAG_12397	58.03647	1.535927	0.29932	5.131385	2.88E-07	2.21E-06
CNAG_12399	16.71343	1.114243	0.527994	2.110331	0.03483	0.07144
CNAG_05290	798.3017	-0.43596	0.164362	-2.65244	0.007991	0.020229
CNAG_05292	8684.86	1.040839	0.17815	5.84249	5.14E-09	5.67E-08
CNAG_05293	3249.927	-0.99946	0.145886	-6.85096	7.34E-12	1.32E-10
CNAG_05294	1574.95	0.697408	0.149043	4.679251	2.88E-06	1.83E-05
CNAG_05296	857.2664	0.465686	0.193823	2.402629	0.016278	0.037361
CNAG_05298	431.166	0.87883	0.166799	5.268791	1.37E-07	1.14E-06
CNAG_05299	420.4938	2.346117	0.164906	14.22703	6.23E-46	1.37E-43
CNAG_05300	1073.725	0.963088	0.132765	7.254102	4.04E-13	8.83E-12
CNAG_05301	4495.53	0.260866	0.123415	2.113728	0.034539	0.070897
CNAG_05302	1567.99	-0.94415	0.140486	-6.7206	1.81E-11	3.00E-10
CNAG_12402	48.21376	1.539331	0.376518	4.088332	4.34E-05	0.000207
CNAG_05304	114.9343	0.849491	0.21757	3.904448	9.44E-05	0.000415
CNAG_12403	16.3743	-1.92758	0.71554	-2.69388	0.007063	0.018268
CNAG_05307	2239.893	-0.57361	0.116338	-4.93059	8.20E-07	5.78E-06
CNAG_05308	1939.234	0.428756	0.214851	1.995595	0.045978	0.090543
CNAG_05312	5912.553	-1.88202	0.129911	-14.4869	1.47E-47	3.53E-45
CNAG_05313	1543.945	-1.10007	0.282918	-3.88828	0.000101	0.00044
CNAG_05314	2989.467	-0.54049	0.197162	-2.74134	0.006119	0.016159
CNAG_05315	214.6364	1.447727	0.182668	7.92546	2.27E-15	6.50E-14
CNAG_12405	158.547	0.620861	0.182356	3.40467	0.000662	0.002318
CNAG_05316	341.8642	-1.07153	0.172727	-6.20358	5.52E-10	7.24E-09
CNAG_05317	484.4409	0.721171	0.175017	4.120574	3.78E-05	0.000183
CNAG_05320	195.6438	-1.18867	0.192809	-6.16503	7.05E-10	9.13E-09
CNAG_12407	247.1847	-1.93082	0.305037	-6.3298	2.45E-10	3.43E-09
CNAG_05321	144.5405	-0.87217	0.191949	-4.54377	5.53E-06	3.29E-05
CNAG_07830	498.3555	-0.52379	0.178063	-2.9416	0.003265	0.009326
CNAG_05324	36.28767	-1.53026	0.364575	-4.19737	2.70E-05	0.000135
CNAG_05329	39.28908	-1.28613	0.4223	-3.04553	0.002323	0.006975
CNAG_05331	40.54319	-0.79034	0.354398	-2.2301	0.025741	0.05516
CNAG_06876	835.9471	-0.82855	0.195026	-4.24842	2.15E-05	0.00011
CNAG_06875	683.8827	0.712558	0.182031	3.914494	9.06E-05	0.000401
CNAG_06873	63.28879	1.250246	0.300718	4.157539	3.22E-05	0.000158

CNAG_12409	42.02049	-1.44668	0.425123	-3.40298	0.000667	0.002326
CNAG_06870	163.3202	0.699063	0.22404	3.120256	0.001807	0.005567
CNAG_06868	4349.777	2.238505	0.196416	11.39674	4.34E-30	4.11E-28
CNAG_06867	932.1456	1.455529	0.212912	6.836294	8.13E-12	1.45E-10
CNAG_06865	774.8699	-0.46687	0.193499	-2.41279	0.015831	0.036525
CNAG_06864	2448.013	-0.52682	0.15359	-3.43004	0.000603	0.002137
CNAG_06863	660.9447	-0.58483	0.172883	-3.38278	0.000718	0.002477
CNAG_07979	1018.758	-0.41274	0.123235	-3.3492	0.00081	0.002758
CNAG_06853	300.1004	1.107217	0.18303	6.049364	1.45E-09	1.80E-08
CNAG_06852	379.6632	-0.49644	0.198373	-2.50254	0.012331	0.02945
CNAG_12414	121.5628	-0.60333	0.292249	-2.06444	0.038976	0.078584
CNAG_07400	10871.93	-0.46499	0.171699	-2.70818	0.006765	0.017608
CNAG_06849	6019.272	-1.12343	0.222016	-5.06014	4.19E-07	3.13E-06
CNAG_06848	2.61401	-2.86569	1.448265	-1.97871	0.047849	0.093647
CNAG_06847	16882.78	-1.18178	0.286157	-4.12984	3.63E-05	0.000177
CNAG_07401	395.5979	0.634766	0.22101	2.872114	0.004077	0.011312
CNAG_06846	652.5874	-0.54683	0.2766	-1.97696	0.048046	0.093987
CNAG_07402	1351.531	0.643336	0.210811	3.051714	0.002275	0.006846
CNAG_06840	54410.56	-0.80762	0.125734	-6.4232	1.33E-10	1.95E-09
CNAG_06839	3645.829	-0.89784	0.235281	-3.81604	0.000136	0.000573
CNAG_06835	678.4215	0.977943	0.18567	5.267098	1.39E-07	1.15E-06
CNAG_06834	3922.616	0.704376	0.144773	4.865378	1.14E-06	7.78E-06
CNAG_06832	441.4761	-1.37551	0.281921	-4.87905	1.07E-06	7.32E-06
CNAG_06831	200.2275	0.915852	0.209442	4.372827	1.23E-05	6.73E-05
CNAG_06830	4922.376	-0.52582	0.123197	-4.26809	1.97E-05	0.000102
CNAG_06829	1884.589	-0.84456	0.134497	-6.27938	3.40E-10	4.65E-09
CNAG_06827	36.1985	0.8526	0.359872	2.369175	0.017828	0.040382
CNAG_06825	839.1502	-0.53256	0.266326	-1.99965	0.045538	0.089765
CNAG_06824	1429.35	-0.43516	0.179983	-2.41779	0.015615	0.03609
CNAG_07404	85.13791	0.715938	0.286359	2.500142	0.012414	0.029587
CNAG_06818	764.7306	-0.58031	0.190709	-3.04291	0.002343	0.007023
CNAG_12420	252.3418	-1.21585	0.250296	-4.85765	1.19E-06	8.07E-06
CNAG_06815	147.2827	-0.79842	0.374612	-2.13131	0.033063	0.068301
CNAG_12422	7.45228	-1.84332	0.79112	-2.33001	0.019806	0.044194
CNAG_06813	3484.895	-0.85814	0.158815	-5.4034	6.54E-08	5.88E-07
CNAG_12423	32.38781	-1.52982	0.481741	-3.17561	0.001495	0.00472
CNAG_06811	18846.97	-1.12709	0.239918	-4.69782	2.63E-06	1.69E-05
CNAG_06807	233.4056	-0.7617	0.287866	-2.646	0.008145	0.020601
CNAG_07406	80.70142	1.513906	0.36598	4.136578	3.53E-05	0.000172
CNAG_07407	80.94351	1.497833	0.326091	4.593301	4.36E-06	2.67E-05
CNAG_07005	1054.757	0.504321	0.144002	3.502186	0.000461	0.00168
CNAG_01455	12402.48	-0.95721	0.256426	-3.73288	0.000189	0.000771
CNAG_12429	84.66806	-0.74444	0.328648	-2.26516	0.023503	0.05125
CNAG_01451	1344.372	-0.46956	0.238173	-1.97153	0.048664	0.095008
CNAG_07413	6028.778	-0.93945	0.136293	-6.89282	5.47E-12	1.00E-10
CNAG_01446	12203.12	2.01732	0.224944	8.968086	3.02E-19	1.30E-17
CNAG_01444	974.7183	-0.70486	0.236283	-2.98312	0.002853	0.008335

CNAG_01441	1522.868	0.789731	0.124987	6.318521	2.64E-10	3.67E-09
CNAG_01439	2380.578	-0.451	0.114878	-3.92588	8.64E-05	0.000384
CNAG_01437	2062.458	-0.97457	0.125062	-7.79268	6.56E-15	1.77E-13
CNAG_01435	8951.149	-0.58757	0.141298	-4.15839	3.20E-05	0.000158
CNAG_01432	2056.004	-0.69128	0.132842	-5.2038	1.95E-07	1.57E-06
CNAG_01430	1042.513	-0.53403	0.145395	-3.67297	0.00024	0.000948
CNAG_01428	30569.31	-0.73825	0.170272	-4.33572	1.45E-05	7.83E-05
CNAG_01425	215.3935	-0.99407	0.270674	-3.67256	0.00024	0.000949
CNAG_01424	1502.333	0.785798	0.302627	2.596585	0.009416	0.023358
CNAG_01422	2873.964	0.828983	0.136111	6.090478	1.13E-09	1.42E-08
CNAG_01421	5111.573	0.694933	0.129532	5.364966	8.10E-08	7.09E-07
CNAG_07416	1084.558	-0.43804	0.202916	-2.15871	0.030872	0.064386
CNAG_01417	1827.65	1.121181	0.198632	5.644507	1.66E-08	1.67E-07
CNAG_01412	3119.712	0.460684	0.129266	3.563836	0.000365	0.001371
CNAG_07418	952.1605	-0.45421	0.196111	-2.3161	0.020553	0.045592
CNAG_01404	8477.965	-0.88046	0.21662	-4.06453	4.81E-05	0.000226
CNAG_01402	6222.176	0.347216	0.106096	3.27266	0.001065	0.003498
CNAG_01401	69.06397	1.190341	0.296222	4.018417	5.86E-05	0.00027
CNAG_01400	7327.763	-1.08566	0.156475	-6.93824	3.97E-12	7.37E-11
CNAG_01399	1178.941	-0.69427	0.2176	-3.19057	0.00142	0.004512
CNAG_01398	3187.976	-0.44612	0.213457	-2.09	0.036618	0.07469
CNAG_01395	3173.359	-0.51579	0.219144	-2.35368	0.018589	0.041819
CNAG_07420	520.213	-0.4139	0.178112	-2.32382	0.020135	0.044829
CNAG_01392	703.257	-0.34423	0.140149	-2.4562	0.014041	0.032919
CNAG_01390	2985.287	-0.92091	0.25387	-3.62747	0.000286	0.001113
CNAG_01389	633.7015	-1.09475	0.156727	-6.98505	2.85E-12	5.44E-11
CNAG_01387	3537.926	0.59399	0.243015	2.444252	0.014515	0.033893
CNAG_01385	2225.135	0.329895	0.129344	2.550528	0.010756	0.026182
CNAG_01384	295.5677	1.040897	0.17003	6.121837	9.25E-10	1.18E-08
CNAG_01381	1573.89	-0.69166	0.22321	-3.09868	0.001944	0.005948
CNAG_01375	2088.011	0.887126	0.143697	6.173599	6.68E-10	8.68E-09
CNAG_01372	1463.122	-1.09384	0.170842	-6.40263	1.53E-10	2.21E-09
CNAG_12437	64.60372	-1.16214	0.471323	-2.46569	0.013675	0.032148
CNAG_01369	128.3092	-0.59746	0.235468	-2.53732	0.01117	0.027045
CNAG_01367	175.4867	1.055496	0.349977	3.015903	0.002562	0.007605
CNAG_01368	11.34998	3.643044	0.863472	4.219065	2.45E-05	0.000124
CNAG_01365	891.744	-1.07739	0.162825	-6.61683	3.67E-11	5.74E-10
CNAG_01364	3895.677	-0.96068	0.247126	-3.88742	0.000101	0.000441
CNAG_01361	6593.432	-0.31969	0.10885	-2.93701	0.003314	0.009455
CNAG_01360	2531.152	0.733869	0.247301	2.967517	0.003002	0.00869
CNAG_01359	3365.318	-0.42756	0.170862	-2.50235	0.012337	0.029453
CNAG_01356	957.9734	0.765417	0.236855	3.23158	0.001231	0.003989
CNAG_01354	2822.475	-1.64968	0.196508	-8.39497	4.66E-17	1.60E-15
CNAG_01351	1707.517	-0.60641	0.22572	-2.68657	0.007219	0.018602
CNAG_01350	1320.952	0.492231	0.163641	3.008003	0.00263	0.007777
CNAG_01348	2822.84	1.811996	0.214701	8.439632	3.18E-17	1.10E-15
CNAG_01347	4235.182	0.934156	0.121328	7.699414	1.37E-14	3.54E-13

CNAG_01345	851.2059	-0.92277	0.231989	-3.97765	6.96E-05	0.000316
CNAG_01344	1376.336	1.554287	0.197605	7.865618	3.67E-15	1.02E-13
CNAG_01341	2834.516	2.353623	0.242935	9.688289	3.38E-22	1.93E-20
CNAG_01339	3412.018	-0.37532	0.116923	-3.20994	0.001328	0.004262
CNAG_01338	1058.171	0.49014	0.223074	2.197213	0.028005	0.05931
CNAG_01334	1901.451	-0.72825	0.269267	-2.70457	0.006839	0.017766
CNAG_01333	727.8299	-0.60476	0.295485	-2.04668	0.04069	0.081605
CNAG_01332	34317.43	-0.97593	0.2791	-3.49672	0.000471	0.001713
CNAG_01331	522.8417	0.467455	0.152961	3.056035	0.002243	0.006764
CNAG_07425	45.46107	1.071247	0.369545	2.898828	0.003746	0.010513
CNAG_01324	569.5881	0.376131	0.149955	2.508291	0.012132	0.029036
CNAG_01323	7481.49	-1.11925	0.231197	-4.84112	1.29E-06	8.72E-06
CNAG_01321	6558.709	-0.47246	0.235176	-2.00898	0.04454	0.088016
CNAG_01320	2406.446	-0.62809	0.218494	-2.87463	0.004045	0.011231
CNAG_01318	3458.25	-0.41187	0.148335	-2.77663	0.005493	0.014745
CNAG_01317	1347.215	-0.89534	0.149005	-6.00882	1.87E-09	2.28E-08
CNAG_01314	2047.558	-0.46676	0.135893	-3.43477	0.000593	0.002106
CNAG_07426	1776.679	0.666885	0.171421	3.890329	0.0001	0.000436
CNAG_07427	14043.19	0.455997	0.105233	4.33322	1.47E-05	7.89E-05
CNAG_01310	1022.595	-0.82374	0.195761	-4.2079	2.58E-05	0.00013
CNAG_01308	1139.805	0.408338	0.178538	2.287125	0.022189	0.048704
CNAG_01307	4651.193	0.466898	0.181914	2.566592	0.01027	0.025195
CNAG_01305	6782.045	-0.64145	0.110459	-5.80714	6.35E-09	6.85E-08
CNAG_01304	1204.083	-0.644	0.134494	-4.78835	1.68E-06	1.11E-05
CNAG_01300	14240.16	-1.13883	0.273606	-4.16229	3.15E-05	0.000156
CNAG_01299	51.31781	1.456659	0.311973	4.669186	3.02E-06	1.91E-05
CNAG_01297	842.3865	0.428366	0.184642	2.319981	0.020342	0.045226
CNAG_01287	11527.44	-1.25545	0.129387	-9.70304	2.93E-22	1.69E-20
CNAG_01285	1612.784	-0.7953	0.161405	-4.92736	8.33E-07	5.87E-06
CNAG_01283	1756.409	-0.90877	0.197673	-4.59736	4.28E-06	2.63E-05
CNAG_01281	671.7877	-0.43338	0.135052	-3.20897	0.001332	0.004275
CNAG_01279	1164.295	-0.77972	0.168729	-4.62113	3.82E-06	2.36E-05
CNAG_07428	2719.083	0.339531	0.168589	2.013961	0.044014	0.087235
CNAG_01278	2323.38	0.557466	0.198728	2.805175	0.005029	0.013638
CNAG_01277	7966.421	0.496007	0.134682	3.682786	0.000231	0.000917
CNAG_12450	30.91744	2.581459	0.525788	4.909699	9.12E-07	6.37E-06
CNAG_01276	3508.749	-0.52871	0.205708	-2.57018	0.010165	0.024952
CNAG_01275	1521.553	1.453022	0.148422	9.789828	1.25E-22	7.55E-21
CNAG_01273	8410.601	-0.84432	0.312663	-2.70042	0.006925	0.017953
CNAG_01272	811.9559	2.149304	0.172838	12.43535	1.68E-35	2.22E-33
CNAG_01271	2960.455	-0.52431	0.154184	-3.40057	0.000672	0.002344
CNAG_07429	2254.546	-0.41498	0.150469	-2.75792	0.005817	0.01548
CNAG_01267	435.0874	-0.86452	0.212251	-4.07309	4.64E-05	0.000219
CNAG_01266	4423.174	0.441842	0.2213	1.996575	0.045871	0.090355
CNAG_01264	9531.564	-0.77968	0.182525	-4.27164	1.94E-05	0.000101
CNAG_01261	7301.176	0.905493	0.169855	5.33097	9.77E-08	8.42E-07
CNAG_06946	2091.037	-1.35988	0.168392	-8.07569	6.71E-16	2.07E-14

CNAG_06948	2910.131	-0.75714	0.111326	-6.80106	1.04E-11	1.83E-10
CNAG_06949	4129.724	-0.62643	0.236626	-2.64734	0.008113	0.02053
CNAG_01258	2800.366	0.479847	0.235842	2.034614	0.04189	0.08357
CNAG_01256	245.8161	-0.47334	0.177429	-2.66779	0.007635	0.019458
CNAG_01255	2767.316	1.174349	0.126677	9.270396	1.85E-20	9.10E-19
CNAG_01254	472.5706	0.496581	0.149583	3.319772	0.000901	0.003015
CNAG_01252	551.3967	0.547945	0.200666	2.730638	0.006321	0.016644
CNAG_01251	3650.645	0.997763	0.336958	2.961086	0.003066	0.00883
CNAG_01247	1054.803	-0.44664	0.185664	-2.40561	0.016145	0.037111
CNAG_01245	1260.525	-0.51131	0.215796	-2.3694	0.017817	0.040381
CNAG_01244	137.6083	3.64265	0.302709	12.03349	2.37E-33	2.82E-31
CNAG_01242	2926.845	0.904733	0.265802	3.40378	0.000665	0.002321
CNAG_01240	768.5472	-0.51905	0.162796	-3.18838	0.001431	0.004537
CNAG_01239	1323.188	-0.58113	0.213271	-2.72487	0.006433	0.016875
CNAG_07981	77.24545	0.911314	0.271404	3.357777	0.000786	0.002681
CNAG_01236	3647.467	-0.79378	0.128199	-6.19181	5.95E-10	7.77E-09
CNAG_01234	2144.726	0.80674	0.28885	2.792935	0.005223	0.014117
CNAG_01232	2567.965	0.851511	0.301865	2.820832	0.00479	0.013061
CNAG_12456	7.555182	2.474006	0.905804	2.731282	0.006309	0.016621
CNAG_01230	5626.209	0.885567	0.162348	5.454755	4.90E-08	4.50E-07
CNAG_01229	734.7625	-0.84061	0.212848	-3.94934	7.84E-05	0.000352
CNAG_07432	150.0049	0.739985	0.303384	2.439101	0.014724	0.03433
CNAG_01224	45598.54	-1.06444	0.216552	-4.91539	8.86E-07	6.21E-06
CNAG_01223	422.3358	2.485368	0.179369	13.85621	1.17E-43	2.32E-41
CNAG_01222	2774.251	-0.6141	0.222629	-2.75839	0.005809	0.015463
CNAG_01221	1100.042	-0.63033	0.160427	-3.92905	8.53E-05	0.00038
CNAG_01213	2081.782	-0.49036	0.162393	-3.01961	0.002531	0.007524
CNAG_01212	1225.418	0.442571	0.186508	2.372932	0.017648	0.040019
CNAG_12458	23.54695	0.979155	0.424822	2.304859	0.021174	0.046789
CNAG_01208	1410.039	0.586501	0.297672	1.970295	0.048805	0.095259
CNAG_01207	601.4402	0.414475	0.161144	2.572079	0.010109	0.02483
CNAG_01204	13514.44	-0.61497	0.261481	-2.35186	0.01868	0.041999
CNAG_01203	1049.632	-0.27065	0.126577	-2.13823	0.032498	0.067369
CNAG_01202	523.0778	-0.43116	0.181896	-2.37035	0.017771	0.040288
CNAG_01201	583.628	-0.38942	0.171995	-2.26414	0.023566	0.051358
CNAG_01200	630.9937	-0.55205	0.206876	-2.66852	0.007619	0.019428
CNAG_01197	2126.857	-0.96829	0.154729	-6.25799	3.90E-10	5.26E-09
CNAG_01192	386.5002	-0.66309	0.20866	-3.17787	0.001484	0.004687
CNAG_01191	656.0215	0.502152	0.136603	3.675985	0.000237	0.000939
CNAG_01188	1077.967	-0.44398	0.131992	-3.36373	0.000769	0.00263
CNAG_01187	1997.506	-0.60126	0.147212	-4.08435	4.42E-05	0.00021
CNAG_01186	2053.705	0.411712	0.139293	2.955731	0.003119	0.008964
CNAG_01184	1578.007	0.732487	0.16643	4.401166	1.08E-05	5.96E-05
CNAG_01183	1911.839	0.265726	0.117537	2.260778	0.023773	0.051711
CNAG_07436	17.79165	1.034969	0.521389	1.985022	0.047142	0.092492
CNAG_01182	8492.668	-0.56732	0.16443	-3.45021	0.00056	0.002004
CNAG_01181	37353.62	-0.94622	0.217375	-4.35296	1.34E-05	7.31E-05

CNAG_01180	518.5021	-0.79308	0.186578	-4.25065	2.13E-05	0.000109
CNAG_01179	1892.955	-0.82571	0.194643	-4.24215	2.21E-05	0.000113
CNAG_07437	730.9099	-0.41053	0.176039	-2.33205	0.019698	0.043991
CNAG_07438	678.3816	-0.31169	0.138292	-2.25386	0.024205	0.052479
CNAG_01174	3329.836	0.768185	0.179725	4.27422	1.92E-05	9.99E-05
CNAG_01171	501.7157	-0.52612	0.265614	-1.98078	0.047616	0.093283
CNAG_01170	52758.91	-1.00914	0.271109	-3.72227	0.000197	0.000799
CNAG_01168	10109.32	-0.91836	0.198484	-4.62687	3.71E-06	2.30E-05
CNAG_01163	1194.297	0.6272	0.187472	3.34556	0.000821	0.002785
CNAG_01161	2508.138	0.438383	0.15129	2.897624	0.00376	0.01055
CNAG_07439	3202.443	-0.34148	0.122556	-2.78634	0.005331	0.014363
CNAG_01159	1883.669	-0.26568	0.11672	-2.27619	0.022835	0.050026
CNAG_01158	5972.228	-0.37412	0.116433	-3.21315	0.001313	0.004225
CNAG_01155	8682.798	0.567096	0.135112	4.197236	2.70E-05	0.000135
CNAG_01154	280.3676	0.414988	0.200264	2.072203	0.038247	0.077429
CNAG_01153	25745.85	-0.55427	0.202109	-2.74244	0.006099	0.016124
CNAG_01152	42005.45	-1.02937	0.233376	-4.41077	1.03E-05	5.73E-05
CNAG_01148	11770.59	-0.65054	0.159951	-4.06714	4.76E-05	0.000224
CNAG_01146	2538.954	-0.49426	0.23845	-2.0728	0.038191	0.077374
CNAG_01145	832.1088	-1.10819	0.146957	-7.54092	4.67E-14	1.14E-12
CNAG_01144	4055.192	0.700363	0.190671	3.673146	0.00024	0.000948
CNAG_01142	2118.601	0.882134	0.268678	3.283234	0.001026	0.003395
CNAG_01141	1000.333	-0.56633	0.242829	-2.33221	0.019689	0.043984
CNAG_01140	2217.491	-0.69629	0.222784	-3.12541	0.001776	0.00549
CNAG_01138	10071.32	-2.09868	0.182862	-11.4768	1.73E-30	1.67E-28
CNAG_01137	9140.795	-0.39625	0.181632	-2.18161	0.029139	0.061318
CNAG_01136	2839.992	-0.56301	0.135054	-4.16879	3.06E-05	0.000152
CNAG_01134	808.052	-0.51306	0.151067	-3.39627	0.000683	0.002377
CNAG_01133	457.9812	-0.50102	0.181794	-2.75596	0.005852	0.015562
CNAG_01132	1169.976	-0.78923	0.13379	-5.899	3.66E-09	4.16E-08
CNAG_01131	430.6303	0.953074	0.165771	5.749342	8.96E-09	9.47E-08
CNAG_12463	13.57317	1.593759	0.567364	2.809059	0.004969	0.013493
CNAG_01130	3405.705	-0.42792	0.183385	-2.33345	0.019625	0.043864
CNAG_01128	2322.918	0.422385	0.166988	2.529426	0.011425	0.02756
CNAG_01127	1233.686	0.426746	0.133267	3.202182	0.001364	0.004356
CNAG_01126	4467.578	0.335282	0.170705	1.964099	0.049519	0.09644
CNAG_12465	10.51798	2.740964	0.740281	3.702601	0.000213	0.000855
CNAG_01124	205.7291	-0.93332	0.289899	-3.21945	0.001284	0.004142
CNAG_01123	1182.266	-0.61858	0.14316	-4.32093	1.55E-05	8.29E-05
CNAG_01121	185.0343	1.549087	0.205595	7.53467	4.90E-14	1.19E-12
CNAG_01120	16577.04	-0.55756	0.11074	-5.03488	4.78E-07	3.54E-06
CNAG_01119	429.6923	-0.70735	0.232579	-3.04133	0.002355	0.007053
CNAG_01118	7592.33	-0.42742	0.195657	-2.18453	0.028923	0.060943
CNAG_12469	61.0564	-2.41013	0.387624	-6.21771	5.04E-10	6.65E-09
CNAG_01117	45869.88	-0.89295	0.21487	-4.15577	3.24E-05	0.00016
CNAG_01116	372.6243	-0.34464	0.171989	-2.00386	0.045085	0.08894
CNAG_01115	2943.645	0.928371	0.371901	2.496287	0.01255	0.029875

CNAG_01113	1460.944	-1.02807	0.229537	-4.47889	7.50E-06	4.32E-05
CNAG_01112	5188.532	1.261425	0.137323	9.185791	4.09E-20	1.88E-18
CNAG_01111	4203.993	-0.71481	0.193039	-3.70294	0.000213	0.000854
CNAG_12470	22.40824	-1.18769	0.537499	-2.20966	0.027129	0.05773
CNAG_01108	1082.132	-1.11265	0.182093	-6.11035	9.94E-10	1.26E-08
CNAG_01107	1781.246	-0.32259	0.147615	-2.18533	0.028865	0.060838
CNAG_01104	1317.804	-0.46537	0.143184	-3.25014	0.001153	0.003759
CNAG_01102	6345.792	2.508447	0.234456	10.69902	1.03E-26	7.64E-25
CNAG_12472	17.47865	2.411074	0.566043	4.259524	2.05E-05	0.000105
CNAG_01097	2439.086	0.765813	0.142941	5.35753	8.44E-08	7.37E-07
CNAG_01095	1201.049	0.960905	0.13089	7.341291	2.12E-13	4.71E-12
CNAG_01092	1740.827	-0.65722	0.150623	-4.36335	1.28E-05	7.00E-05
CNAG_01090	793.6839	1.82608	0.16426	11.117	1.04E-28	8.77E-27
CNAG_01089	1512.707	-0.77238	0.210961	-3.66124	0.000251	0.000987
CNAG_01088	1250.149	-0.37467	0.136618	-2.74245	0.006098	0.016124
CNAG_12473	17.95653	-1.10277	0.493368	-2.23519	0.025405	0.054574
CNAG_01083	7631.149	0.593022	0.13127	4.517556	6.26E-06	3.68E-05
CNAG_01082	1604.731	0.650913	0.323695	2.010882	0.044338	0.087748
CNAG_01081	200.2262	0.921369	0.279344	3.298327	0.000973	0.003235
CNAG_01080	195.162	-0.90216	0.32783	-2.75192	0.005925	0.01574
CNAG_01077	40.22078	-0.91583	0.341104	-2.68491	0.007255	0.018668
CNAG_01075	46.52481	-1.00837	0.395706	-2.54829	0.010825	0.026329
CNAG_01072	269.0297	-0.4604	0.165012	-2.79008	0.00527	0.014218
CNAG_01070	28.46529	-1.09141	0.472962	-2.30761	0.021021	0.046488
CNAG_07443	54.30638	2.431494	0.3612	6.731717	1.68E-11	2.80E-10
CNAG_01060	9964.917	-0.64906	0.263421	-2.46395	0.013742	0.032295
CNAG_01059	612.2416	1.255645	0.13146	9.55153	1.28E-21	6.91E-20
CNAG_01058	1323.25	-0.3102	0.133539	-2.32292	0.020183	0.044898
CNAG_01057	2354.965	0.379611	0.169317	2.242015	0.02496	0.053743
CNAG_01056	49.78544	1.232127	0.323552	3.808126	0.00014	0.000589
CNAG_07444	118.17	0.747083	0.249583	2.993328	0.00276	0.008108
CNAG_12480	4.273506	-2.94385	1.151	-2.55764	0.010538	0.02575
CNAG_01055	10589.16	-0.49137	0.134926	-3.64178	0.000271	0.001058
CNAG_01054	887.0286	-0.52424	0.189453	-2.76715	0.005655	0.015134
CNAG_01052	13353.36	1.601719	0.277105	5.780198	7.46E-09	7.99E-08
CNAG_01050	1145.59	-0.50184	0.181404	-2.76643	0.005667	0.015147
CNAG_01048	185.3823	1.780798	0.24966	7.132886	9.83E-13	2.04E-11
CNAG_01047	851.5696	2.736308	0.260722	10.49513	9.10E-26	6.51E-24
CNAG_01045	2670.062	-0.24268	0.120219	-2.01867	0.043522	0.086412
CNAG_01044	1149.048	0.330972	0.149158	2.218936	0.026491	0.05657
CNAG_01043	11855.32	0.673947	0.190984	3.528815	0.000417	0.001543
CNAG_12483	31.93909	-0.75746	0.376734	-2.01059	0.044369	0.087784
CNAG_01041	269.3645	0.426581	0.180663	2.361201	0.018216	0.041179
CNAG_01039	5474.518	0.448671	0.184455	2.432415	0.014999	0.034858
CNAG_01037	762.9138	0.451239	0.212878	2.11971	0.03403	0.069999
CNAG_01035	7603.36	0.7816	0.16483	4.741868	2.12E-06	1.38E-05
CNAG_01034	71.09951	0.684421	0.259061	2.641926	0.008244	0.020808

CNAG_01032	394.7294	0.794994	0.324973	2.44634	0.014431	0.033737
CNAG_01031	5077.946	2.196739	0.209864	10.46744	1.22E-25	8.65E-24
CNAG_01027	1222.536	0.759537	0.125942	6.030867	1.63E-09	2.01E-08
CNAG_01026	974.8293	0.834537	0.160285	5.206592	1.92E-07	1.55E-06
CNAG_01022	1033.31	0.928076	0.154869	5.992654	2.06E-09	2.50E-08
CNAG_01021	3293.014	-0.60363	0.134182	-4.49856	6.84E-06	3.98E-05
CNAG_01020	1818.633	-0.6964	0.233039	-2.98837	0.002805	0.008218
CNAG_01018	6792.381	-0.57299	0.131119	-4.36997	1.24E-05	6.81E-05
CNAG_01010	3346.328	0.61928	0.215751	2.870344	0.0041	0.011364
CNAG_01009	1044.253	-0.55357	0.185469	-2.9847	0.002839	0.008302
CNAG_01008	845.1685	0.819034	0.133814	6.120684	9.32E-10	1.19E-08
CNAG_01007	1753.303	1.247057	0.181816	6.858908	6.94E-12	1.26E-10
CNAG_01004	3127.796	0.851083	0.182633	4.660058	3.16E-06	1.99E-05
CNAG_01003	3317.472	-0.27851	0.110174	-2.5279	0.011475	0.027655
CNAG_01001	181.6225	1.571655	0.233408	6.733498	1.66E-11	2.77E-10
CNAG_01000	3525.955	-0.39799	0.118482	-3.35906	0.000782	0.002671
CNAG_00997	813.3189	-1.28632	0.348697	-3.68894	0.000225	0.000897
CNAG_00996	4335.951	0.213025	0.107774	1.97659	0.048088	0.094022
CNAG_00995	8995.299	2.264207	0.176367	12.83804	1.00E-37	1.48E-35
CNAG_00994	2338.781	-0.72423	0.248589	-2.91335	0.003576	0.010108
CNAG_00992	12689.84	-0.52827	0.169409	-3.11834	0.001819	0.005597
CNAG_00991	2246.505	-0.36669	0.11933	-3.07291	0.00212	0.006439
CNAG_00990	6275.034	-0.7564	0.26723	-2.83052	0.004647	0.012703
CNAG_00987	1610.519	-0.60727	0.2132	-2.84837	0.004394	0.012116
CNAG_00986	1006.423	-0.42531	0.145386	-2.92539	0.00344	0.00977
CNAG_00984	7784.757	4.129282	0.18335	22.52131	2.57E-112	6.80E-109
CNAG_00982	1719.478	-0.61238	0.172394	-3.55222	0.000382	0.001428
CNAG_00981	25.0946	1.45473	0.438718	3.315861	0.000914	0.003054
CNAG_12488	118.4809	-1.71338	0.358243	-4.78275	1.73E-06	1.14E-05
CNAG_00979	492.6959	-1.62161	0.239982	-6.7572	1.41E-11	2.40E-10
CNAG_00978	2501.663	-1.03281	0.272278	-3.79322	0.000149	0.000621
CNAG_00976	9162.302	-0.82655	0.117441	-7.03797	1.95E-12	3.88E-11
CNAG_00975	250.1801	-0.71555	0.244148	-2.93079	0.003381	0.009626
CNAG_00972	2095.117	-0.7422	0.127705	-5.81184	6.18E-09	6.68E-08
CNAG_12489	322.0428	-1.09388	0.256086	-4.27155	1.94E-05	0.000101
CNAG_00968	310.8055	0.536186	0.236516	2.267016	0.023389	0.051044
CNAG_07445	32493.45	-0.3891	0.126797	-3.06868	0.00215	0.006521
CNAG_00961	2504.188	1.989484	0.185562	10.72138	8.08E-27	6.11E-25
CNAG_12491	2237.242	3.495378	0.290957	12.01339	3.02E-33	3.53E-31
CNAG_12492	596.3629	0.542971	0.172894	3.140491	0.001687	0.005252
CNAG_00938	527.536	0.514189	0.155497	3.306756	0.000944	0.00315
CNAG_00935	10735.37	-0.40526	0.196376	-2.0637	0.039046	0.078687
CNAG_00933	510.4658	0.568159	0.214904	2.643785	0.008198	0.020707
CNAG_00932	1145.775	2.868044	0.241871	11.85776	1.96E-32	2.20E-30
CNAG_00930	12863.68	-0.81405	0.124756	-6.52515	6.79E-11	1.03E-09
CNAG_00929	1155.627	-0.56635	0.203584	-2.7819	0.005404	0.014527
CNAG_00925	297.3377	0.933497	0.167904	5.559691	2.70E-08	2.66E-07

CNAG_00923	620.0048	0.950306	0.244457	3.887407	0.000101	0.000441
CNAG_00921	852.4307	1.080286	0.208251	5.187417	2.13E-07	1.69E-06
CNAG_00920	1182.618	0.978094	0.226582	4.316738	1.58E-05	8.43E-05
CNAG_00919	3222.597	-2.01026	0.421883	-4.76497	1.89E-06	1.24E-05
CNAG_00917	1274.964	-0.36465	0.161018	-2.26466	0.023534	0.051303
CNAG_00915	312.272	1.890209	0.1659	11.39367	4.50E-30	4.20E-28
CNAG_12499	128.9628	-0.72707	0.318779	-2.28081	0.02256	0.049465
CNAG_12500	7.512711	2.46614	0.831197	2.966974	0.003007	0.008703
CNAG_07450	219.9396	0.559016	0.234114	2.387796	0.01695	0.038646
CNAG_07982	964.1729	-1.3568	0.137277	-9.88363	4.90E-23	3.04E-21
CNAG_00908	37.28126	0.812642	0.360254	2.25575	0.024086	0.052249
CNAG_00906	3379.571	-0.43099	0.148077	-2.91058	0.003608	0.010183
CNAG_00905	290.9473	1.565361	0.1604	9.759092	1.69E-22	1.00E-20
CNAG_00904	662.2655	2.627919	0.173703	15.12878	1.05E-51	2.60E-49
CNAG_00903	651.6811	-0.48052	0.146201	-3.2867	0.001014	0.003359
CNAG_00901	360.3833	0.726414	0.209745	3.463313	0.000534	0.00192
CNAG_00900	208.3901	-0.39627	0.189084	-2.09572	0.036107	0.073812
CNAG_00899	861.3523	-0.75004	0.219105	-3.42318	0.000619	0.002183
CNAG_12503	22.64817	1.036354	0.479448	2.161559	0.030652	0.064028
CNAG_00896	560.8143	-1.77247	0.316959	-5.59211	2.24E-08	2.23E-07
CNAG_12505	31.39564	-3.6998	0.754824	-4.90154	9.51E-07	6.63E-06
CNAG_12506	43.06085	-2.63612	0.478791	-5.50579	3.68E-08	3.51E-07
CNAG_00895	20602.87	-4.43032	0.832515	-5.32161	1.03E-07	8.82E-07
CNAG_00894	4504.303	-0.92187	0.27833	-3.31215	0.000926	0.003092
CNAG_00886	26381.14	-0.49376	0.201031	-2.45613	0.014044	0.032919
CNAG_10063	2.091282	3.392781	1.635475	2.074492	0.038034	0.077115
CNAG_00883	840.1967	-1.51658	0.292238	-5.18952	2.11E-07	1.67E-06
CNAG_12510	14.25785	-1.90039	0.662013	-2.87062	0.004097	0.011358
CNAG_07453	1946.168	-0.46689	0.115772	-4.0328	5.51E-05	0.000256
CNAG_00877	1204.232	-0.80774	0.223491	-3.61417	0.000301	0.001163
CNAG_00876	302.8472	0.903026	0.183949	4.909117	9.15E-07	6.39E-06
CNAG_00873	1006.54	2.098118	0.403669	5.197617	2.02E-07	1.61E-06
CNAG_00870	48.29618	1.084342	0.374295	2.897027	0.003767	0.010562
CNAG_12511	43.31428	2.866071	0.487513	5.878966	4.13E-09	4.64E-08
CNAG_00869	624.9839	-0.60321	0.202465	-2.97931	0.002889	0.008421
CNAG_00866	3115.928	1.848901	0.199893	9.249442	2.26E-20	1.10E-18
CNAG_07456	301.021	0.76315	0.181316	4.208958	2.57E-05	0.00013
CNAG_07458	203.6846	1.2102	0.220571	5.486682	4.10E-08	3.83E-07
CNAG_00863	100.2857	0.470646	0.239236	1.967283	0.049151	0.095794
CNAG_00862	592.4033	-0.73861	0.248267	-2.97506	0.002929	0.008514
CNAG_00861	145.8064	-0.72797	0.203107	-3.58415	0.000338	0.001284
CNAG_00860	297.2162	-0.49336	0.222549	-2.21687	0.026632	0.056826
CNAG_02554	484.9447	-0.43971	0.143545	-3.06321	0.00219	0.006626
CNAG_02553	137.8463	-1.93815	0.292062	-6.63608	3.22E-11	5.14E-10
CNAG_02552	321.6267	0.555464	0.245571	2.261932	0.023702	0.051584
CNAG_02551	183.2026	-1.01492	0.309969	-3.27426	0.001059	0.00348
CNAG_02550	981.2239	1.628567	0.250165	6.509963	7.52E-11	1.13E-09

CNAG_02549	1079.368	0.670289	0.198334	3.379606	0.000726	0.002499
CNAG_02548	21595.46	-5.1498	1.206536	-4.26825	1.97E-05	0.000102
CNAG_02546	3238.391	-0.61125	0.179237	-3.41029	0.000649	0.002275
CNAG_02545	14092.76	-0.57459	0.170827	-3.36357	0.000769	0.002631
CNAG_02544	64.72639	1.298355	0.366389	3.543648	0.000395	0.001467
CNAG_02543	1199.098	0.650297	0.145466	4.470428	7.81E-06	4.46E-05
CNAG_02542	3656.266	0.758947	0.203731	3.725248	0.000195	0.000791
CNAG_02540	897.2199	-1.10932	0.165631	-6.69755	2.12E-11	3.48E-10
CNAG_12515	132.8764	1.760657	0.233197	7.550099	4.35E-14	1.07E-12
CNAG_02537	10.62093	1.593957	0.70904	2.248049	0.024573	0.053131
CNAG_02536	897.5579	-0.65501	0.193032	-3.39325	0.000691	0.002399
CNAG_02534	1102.769	-0.33429	0.143886	-2.3233	0.020163	0.044865
CNAG_12517	141.4276	-0.87761	0.389031	-2.25588	0.024078	0.052246
CNAG_02531	338.1798	1.070845	0.157139	6.814632	9.45E-12	1.67E-10
CNAG_02530	3119.827	-0.30717	0.132676	-2.31521	0.020601	0.045674
CNAG_02528	1283.877	0.769717	0.186062	4.136885	3.52E-05	0.000172
CNAG_02527	3899.208	1.367791	0.148719	9.197136	3.68E-20	1.70E-18
CNAG_12518	70.89163	1.031904	0.252279	4.090322	4.31E-05	0.000205
CNAG_02526	24.51124	1.092718	0.45416	2.406018	0.016127	0.03708
CNAG_02524	714.992	0.527936	0.259293	2.036061	0.041744	0.083362
CNAG_02523	1285.364	1.674869	0.164442	10.18515	2.31E-24	1.57E-22
CNAG_02520	1442.593	-0.56323	0.130034	-4.33146	1.48E-05	7.94E-05
CNAG_02519	2530.095	0.648928	0.23478	2.76398	0.00571	0.015241
CNAG_02516	581.8195	0.501647	0.146902	3.414831	0.000638	0.002245
CNAG_07622	2032.305	0.3081	0.120387	2.559242	0.01049	0.025671
CNAG_02512	2222.651	0.914821	0.129538	7.06217	1.64E-12	3.31E-11
CNAG_12522	46.66488	2.137973	0.351815	6.076989	1.22E-09	1.53E-08
CNAG_02510	1370.621	0.757951	0.14379	5.27123	1.36E-07	1.13E-06
CNAG_02509	83.07836	0.501109	0.245976	2.037228	0.041627	0.08315
CNAG_02508	532.4794	-2.17372	0.235405	-9.234	2.61E-20	1.25E-18
CNAG_02507	4399.846	-0.52606	0.168528	-3.12147	0.001799	0.005549
CNAG_02506	1783.275	-0.79967	0.223899	-3.57156	0.000355	0.00134
CNAG_02505	1426.216	1.728921	0.155026	11.15245	6.97E-29	6.02E-27
CNAG_02504	651.2643	-0.9085	0.196406	-4.62561	3.73E-06	2.31E-05
CNAG_02502	2594.279	-0.68269	0.124288	-5.49278	3.96E-08	3.73E-07
CNAG_02500	19482.61	0.489001	0.208385	2.346626	0.018944	0.042499
CNAG_02497	157.3386	1.666218	0.208641	7.986037	1.39E-15	4.12E-14
CNAG_02495	2050.98	-0.30065	0.135522	-2.21843	0.026525	0.056629
CNAG_12523	170.9632	-1.64226	0.405014	-4.05481	5.02E-05	0.000235
CNAG_02491	590.1597	-1.11185	0.168463	-6.59993	4.11E-11	6.40E-10
CNAG_02488	698.5069	1.919247	0.33834	5.672548	1.41E-08	1.44E-07
CNAG_02486	4892.117	-0.68075	0.250379	-2.71888	0.00655	0.017121
CNAG_02485	17182.01	-0.42887	0.171392	-2.50229	0.012339	0.029453
CNAG_07625	2537.67	-0.27327	0.137874	-1.98206	0.047473	0.093026
CNAG_12526	25.60875	1.01378	0.425768	2.381063	0.017263	0.03927
CNAG_02482	1489.252	-0.41595	0.184162	-2.25863	0.023906	0.051944
CNAG_12527	22.37138	1.510621	0.427217	3.535956	0.000406	0.001507

CNAG_02479	268.4159	-0.85492	0.331614	-2.57806	0.009936	0.024488
CNAG_02475	673.6301	0.447124	0.125219	3.570728	0.000356	0.001342
CNAG_07626	248.8011	0.68691	0.161087	4.264205	2.01E-05	0.000104
CNAG_02473	2201.585	1.345487	0.164943	8.157308	3.43E-16	1.09E-14
CNAG_12531	59.99577	1.034245	0.26814	3.857105	0.000115	0.000492
CNAG_02471	802.2683	-0.50763	0.207662	-2.4445	0.014505	0.033881
CNAG_02470	1353.816	0.464487	0.151382	3.068306	0.002153	0.006526
CNAG_02469	1150.107	-0.25433	0.121254	-2.09753	0.035947	0.073541
CNAG_02463	72.43378	-1.02803	0.268489	-3.82896	0.000129	0.000545
CNAG_02460	627.668	-1.10161	0.398813	-2.76222	0.005741	0.015318
CNAG_12535	38.14218	-1.77764	0.44839	-3.9645	7.35E-05	0.000332
CNAG_02459	365.4704	0.765285	0.344059	2.224286	0.026129	0.055873
CNAG_02458	2759.442	0.470073	0.20402	2.304051	0.02122	0.046876
CNAG_07628	10821.46	-0.46731	0.153987	-3.03475	0.002407	0.007191
CNAG_07629	2754.872	0.804082	0.180121	4.464132	8.04E-06	4.58E-05
CNAG_02449	1324.71	-0.74566	0.110369	-6.75604	1.42E-11	2.41E-10
CNAG_12537	70.30083	1.208229	0.263682	4.582154	4.60E-06	2.79E-05
CNAG_02448	24.27759	0.895426	0.430657	2.079211	0.037598	0.076411
CNAG_02444	1185.14	1.241799	0.176269	7.044891	1.86E-12	3.71E-11
CNAG_02443	2669.771	-0.87774	0.242408	-3.62091	0.000294	0.001136
CNAG_02442	429.6388	-0.66268	0.161904	-4.09307	4.26E-05	0.000203
CNAG_02441	18.57564	2.662636	0.68131	3.90811	9.30E-05	0.000409
CNAG_02440	5948.611	0.741794	0.110944	6.686198	2.29E-11	3.73E-10
CNAG_02438	391.7374	1.616148	0.181585	8.900206	5.57E-19	2.31E-17
CNAG_12538	51.52562	2.280461	0.38293	5.955296	2.60E-09	3.07E-08
CNAG_02437	1063.421	-0.74026	0.15013	-4.93078	8.19E-07	5.77E-06
CNAG_12540	49.40316	1.895029	0.316186	5.9934	2.05E-09	2.49E-08
CNAG_02435	3826.964	0.376244	0.155501	2.419555	0.01554	0.035926
CNAG_02433	1354.579	0.732102	0.140659	5.204808	1.94E-07	1.57E-06
CNAG_02430	461.4022	1.045821	0.319373	3.274606	0.001058	0.003479
CNAG_02427	901.3469	1.232428	0.159861	7.709354	1.26E-14	3.28E-13
CNAG_02426	4479.382	-0.40724	0.168869	-2.4116	0.015883	0.036634
CNAG_02424	880.8036	-0.78329	0.143501	-5.45843	4.80E-08	4.42E-07
CNAG_02421	4073.376	-0.74448	0.236359	-3.14979	0.001634	0.005113
CNAG_02418	12032.1	-1.12482	0.128482	-8.75466	2.05E-18	8.01E-17
CNAG_02417	259.3887	1.073767	0.498812	2.152649	0.031346	0.065238
CNAG_12541	148.549	0.700784	0.266149	2.633052	0.008462	0.021299
CNAG_02416	3893.818	-0.57556	0.262499	-2.19263	0.028334	0.059847
CNAG_02413	4610.474	-0.38304	0.133838	-2.86195	0.00421	0.011653
CNAG_02412	617.9803	-1.25174	0.210441	-5.94817	2.71E-09	3.19E-08
CNAG_12542	8.645919	1.654296	0.743529	2.224925	0.026086	0.055796
CNAG_12543	40.42945	-0.79203	0.383236	-2.06668	0.038764	0.078178
CNAG_07631	2645.862	1.572647	0.317924	4.946618	7.55E-07	5.38E-06
CNAG_02409	884.8568	0.415587	0.164776	2.522137	0.011664	0.028044
CNAG_02407	3119.648	0.630741	0.193869	3.253444	0.00114	0.003721
CNAG_02406	1014.971	0.4251	0.189291	2.245754	0.02472	0.053347
CNAG_02405	920.5734	-0.36787	0.163687	-2.24741	0.024614	0.053201

CNAG_02403	2956.793	-0.88401	0.251	-3.52197	0.000428	0.001577
CNAG_02400	3840.155	0.423171	0.204713	2.067147	0.03872	0.078149
CNAG_02398	1754.49	1.671938	0.255324	6.548295	5.82E-11	8.88E-10
CNAG_02394	733.678	-0.30674	0.15494	-1.97973	0.047733	0.093467
CNAG_02389	307.0505	0.756312	0.317967	2.378586	0.017379	0.039482
CNAG_12549	57.85145	0.656692	0.262331	2.503297	0.012304	0.029395
CNAG_02388	661.3557	-0.5505	0.203917	-2.69963	0.006942	0.017984
CNAG_02387	1347.371	0.604277	0.182137	3.317706	0.000908	0.003035
CNAG_02385	681.904	-0.29786	0.146094	-2.03885	0.041465	0.082909
CNAG_02383	499.025	-0.51068	0.168073	-3.03844	0.002378	0.007117
CNAG_02382	2973.953	-0.68062	0.191169	-3.56031	0.00037	0.001388
CNAG_07986	679.0184	-0.62473	0.137783	-4.53415	5.78E-06	3.42E-05
CNAG_02378	6853.396	-0.67708	0.209878	-3.22606	0.001255	0.004057
CNAG_02376	1143.599	-0.98	0.193107	-5.07489	3.88E-07	2.91E-06
CNAG_02373	1666.898	0.575831	0.189167	3.04403	0.002334	0.007007
CNAG_02372	2900.802	0.286262	0.118262	2.420571	0.015496	0.035846
CNAG_02371	376.4102	-0.54292	0.153441	-3.5383	0.000403	0.001495
CNAG_07633	270.0906	0.56295	0.167696	3.356968	0.000788	0.002686
CNAG_02366	600.4916	-0.82282	0.170351	-4.83014	1.36E-06	9.17E-06
CNAG_02365	596.9787	-1.13304	0.181511	-6.24229	4.31E-10	5.79E-09
CNAG_02362	1255.387	1.488455	0.126791	11.73944	8.00E-32	8.26E-30
CNAG_02361	1986.333	-0.77998	0.302885	-2.57518	0.010019	0.024654
CNAG_12555	19.91922	-1.76742	0.529587	-3.33736	0.000846	0.002855
CNAG_02360	1937.897	0.510059	0.130307	3.914281	9.07E-05	0.000401
CNAG_02359	34516.57	-1.06406	0.30116	-3.5332	0.000411	0.001521
CNAG_02357	1092.657	0.703143	0.255243	2.754801	0.005873	0.015607
CNAG_02355	1289.456	1.06255	0.166837	6.3688	1.91E-10	2.71E-09
CNAG_02354	2760.404	-0.69173	0.161888	-4.2729	1.93E-05	0.0001
CNAG_02351	2461.688	1.377846	0.132355	10.41022	2.23E-25	1.55E-23
CNAG_02350	904.4119	-0.27358	0.134179	-2.03889	0.041461	0.082909
CNAG_02348	728.7601	0.373972	0.187957	1.989671	0.046627	0.09164
CNAG_12557	4.547671	2.184156	1.043246	2.093615	0.036294	0.074119
CNAG_02343	1635.364	0.610846	0.123781	4.934889	8.02E-07	5.67E-06
CNAG_02342	577.2899	0.645677	0.151132	4.272268	1.93E-05	0.000101
CNAG_02338	16258.8	-0.59445	0.234996	-2.5296	0.011419	0.027555
CNAG_02337	2934.459	-2.17658	0.669226	-3.25238	0.001144	0.003733
CNAG_02335	19224.54	1.096374	0.118666	9.239186	2.48E-20	1.20E-18
CNAG_02334	13.97116	1.126913	0.565847	1.991552	0.04642	0.091278
CNAG_02333	493.5205	-0.45305	0.222851	-2.03298	0.042055	0.083793
CNAG_02331	23681.16	-1.20159	0.301039	-3.99148	6.57E-05	0.0003
CNAG_02330	27762.45	-0.81704	0.200144	-4.08228	4.46E-05	0.000212
CNAG_02325	1235.074	0.506459	0.145027	3.492171	0.000479	0.001738
CNAG_02323	5698.284	-0.65729	0.226127	-2.90673	0.003652	0.010295
CNAG_12560	153.0386	-1.31931	0.351896	-3.74914	0.000177	0.00073
CNAG_02322	1953.031	-0.88273	0.378911	-2.32964	0.019825	0.044213
CNAG_02319	597.1772	-0.81802	0.264619	-3.0913	0.001993	0.006086
CNAG_02317	1108.203	-0.96521	0.140928	-6.84892	7.44E-12	1.34E-10

CNAG_02316	1700.197	-0.66115	0.198068	-3.33798	0.000844	0.00285
CNAG_02315	13695.72	-1.13208	0.252882	-4.4767	7.58E-06	4.35E-05
CNAG_12562	41.78188	1.215694	0.321612	3.780005	0.000157	0.00065
CNAG_02313	710.6406	1.089325	0.208998	5.212122	1.87E-07	1.52E-06
CNAG_02312	1645.703	1.176353	0.238444	4.933445	8.08E-07	5.70E-06
CNAG_02309	1576.349	0.443344	0.187125	2.369241	0.017825	0.040382
CNAG_02305	491.1276	-0.44367	0.208368	-2.12927	0.033232	0.068561
CNAG_02301	7518.334	-1.13874	0.182087	-6.25378	4.01E-10	5.39E-09
CNAG_02300	14987.51	-0.70894	0.125342	-5.65606	1.55E-08	1.57E-07
CNAG_02299	819.6658	-0.32986	0.154317	-2.13751	0.032556	0.067438
CNAG_02298	3518.851	1.096804	0.253206	4.331666	1.48E-05	7.94E-05
CNAG_02297	622.944	0.741868	0.198839	3.73099	0.000191	0.000776
CNAG_02294	3631.611	-0.43218	0.17686	-2.4436	0.014542	0.033945
CNAG_02293	1454.336	-0.55672	0.142706	-3.90119	9.57E-05	0.000419
CNAG_02292	2852.996	-0.58143	0.164526	-3.53397	0.000409	0.001517
CNAG_02291	836.5653	-0.68782	0.242668	-2.8344	0.004591	0.01258
CNAG_02289	821.1812	0.78986	0.171511	4.605313	4.12E-06	2.54E-05
CNAG_02285	19521.86	-0.47569	0.238496	-1.99453	0.046094	0.090726
CNAG_02284	336.3327	0.89418	0.173347	5.158325	2.49E-07	1.94E-06
CNAG_02283	138.092	1.630683	0.420544	3.877554	0.000106	0.000456
CNAG_02282	2180.947	0.90342	0.161058	5.609285	2.03E-08	2.03E-07
CNAG_02281	972.7496	-0.41647	0.139134	-2.99328	0.00276	0.008108
CNAG_07635	1625.824	-1.03776	0.176242	-5.88826	3.90E-09	4.42E-08
CNAG_07636	1616.638	-0.37502	0.140683	-2.66575	0.007682	0.019551
CNAG_07637	3041.593	-0.84073	0.229014	-3.6711	0.000242	0.000953
CNAG_07638	1468.265	1.385617	0.165783	8.358	6.38E-17	2.17E-15
CNAG_07640	887.6153	-0.72025	0.238787	-3.01631	0.002559	0.007599
CNAG_07641	133.6074	0.77654	0.202953	3.826206	0.00013	0.00055
CNAG_02270	1959.924	-0.45812	0.134701	-3.40097	0.000671	0.002341
CNAG_02267	1417.507	-0.52022	0.163503	-3.18174	0.001464	0.004629
CNAG_07164	1187.43	1.487262	0.310965	4.782729	1.73E-06	1.14E-05
CNAG_07163	1333.15	-1.01139	0.20207	-5.00512	5.58E-07	4.09E-06
CNAG_02266	3680.719	-1.42816	0.275262	-5.18835	2.12E-07	1.68E-06
CNAG_02264	2448.956	1.017122	0.22392	4.542356	5.56E-06	3.30E-05
CNAG_02263	792.2673	1.831491	0.714111	2.564713	0.010326	0.025317
CNAG_02262	3249.859	0.591398	0.289051	2.045998	0.040757	0.081719
CNAG_02257	17292.18	-0.45639	0.176213	-2.58997	0.009598	0.023742
CNAG_12570	97.68648	1.037631	0.36622	2.833354	0.004606	0.012608
CNAG_12571	281.6466	1.180731	0.300681	3.926859	8.61E-05	0.000383
CNAG_02240	3346.142	0.621537	0.249705	2.489084	0.012807	0.030423
CNAG_02239	7299.192	0.654498	0.125975	5.195448	2.04E-07	1.63E-06
CNAG_02235	544.7813	-0.62393	0.178618	-3.49308	0.000477	0.001735
CNAG_02234	43410.99	-1.06252	0.226058	-4.70022	2.60E-06	1.67E-05
CNAG_02232	2079.396	-0.34295	0.135867	-2.52414	0.011598	0.02791
CNAG_02230	10315.51	2.434234	0.249616	9.751925	1.81E-22	1.07E-20
CNAG_07646	189.8158	0.591967	0.195698	3.024899	0.002487	0.007412
CNAG_02227	450.262	0.41965	0.1606	2.613021	0.008975	0.022369

CNAG_02226	1101.193	2.500566	0.194557	12.8526	8.32E-38	1.25E-35
CNAG_02225	318.9154	1.790607	0.21777	8.222474	1.99E-16	6.41E-15
CNAG_02223	3935.732	0.398157	0.166169	2.39609	0.016571	0.037892
CNAG_02220	1379	0.339934	0.167076	2.03461	0.04189	0.08357
CNAG_02219	810.3084	0.686428	0.123497	5.558248	2.72E-08	2.68E-07
CNAG_02217	224.1793	1.225371	0.202226	6.059416	1.37E-09	1.70E-08
CNAG_12573	611.94	3.047967	1.211893	2.515046	0.011902	0.028537
CNAG_02216	2475.416	0.680595	0.21571	3.155145	0.001604	0.00503
CNAG_02212	12175.79	-0.65012	0.111752	-5.81753	5.97E-09	6.48E-08
CNAG_02211	662.1022	1.296059	0.214246	6.049404	1.45E-09	1.80E-08
CNAG_02209	8240.496	-0.90882	0.192045	-4.73231	2.22E-06	1.44E-05
CNAG_02207	674.6024	0.503331	0.14592	3.44935	0.000562	0.00201
CNAG_02206	16.1647	3.43735	0.660119	5.207167	1.92E-07	1.55E-06
CNAG_02205	534.2471	0.359369	0.176126	2.04041	0.041309	0.082702
CNAG_02204	1233.913	-0.66023	0.150818	-4.37764	1.20E-05	6.60E-05
CNAG_02203	1106.119	-0.70304	0.119574	-5.87957	4.11E-09	4.63E-08
CNAG_02202	3602.464	-0.83133	0.151253	-5.49628	3.88E-08	3.67E-07
CNAG_02198	458.8196	-0.3897	0.136164	-2.86201	0.00421	0.011653
CNAG_02197	2119.243	-0.54993	0.193954	-2.83534	0.004578	0.012551
CNAG_02196	1953.402	-0.39548	0.165892	-2.38397	0.017127	0.039006
CNAG_02195	396.2707	-0.99207	0.189481	-5.23573	1.64E-07	1.35E-06
CNAG_02194	974.6854	-1.40477	0.177356	-7.92065	2.36E-15	6.71E-14
CNAG_02193	721.5058	-1.35278	0.209581	-6.45469	1.08E-10	1.61E-09
CNAG_02192	29.38148	1.560358	0.407816	3.82613	0.00013	0.00055
CNAG_02190	722.6417	-0.51721	0.256827	-2.01384	0.044027	0.087235
CNAG_02189	3808.713	0.938761	0.203807	4.606119	4.10E-06	2.53E-05
CNAG_12579	15.31994	1.499026	0.523853	2.86154	0.004216	0.011664
CNAG_02188	839.7304	1.137473	0.182328	6.238604	4.41E-10	5.89E-09
CNAG_02185	841.7553	-0.36919	0.16497	-2.2379	0.025228	0.054237
CNAG_12580	12.37219	1.4508	0.592661	2.447943	0.014367	0.033617
CNAG_02182	5848.313	2.066435	0.125927	16.40977	1.63E-60	6.81E-58
CNAG_02176	3254.198	0.72452	0.27815	2.604778	0.009193	0.022864
CNAG_02174	5247.117	-0.34648	0.108352	-3.19777	0.001385	0.004414
CNAG_02173	801.128	0.597045	0.198697	3.004804	0.002658	0.007847
CNAG_02170	170.5962	1.038706	0.254619	4.079446	4.51E-05	0.000214
CNAG_02169	140.87	2.128318	0.274546	7.752139	9.04E-15	2.41E-13
CNAG_02168	1697.359	0.870665	0.280523	3.103723	0.001911	0.005856
CNAG_02167	1127.518	0.539811	0.252516	2.137734	0.032538	0.067436
CNAG_02165	1922.333	0.330121	0.137562	2.399802	0.016404	0.037585
CNAG_02164	3123.693	1.597887	0.14208	11.24642	2.41E-29	2.15E-27
CNAG_02163	378.8315	-0.86499	0.155751	-5.55371	2.80E-08	2.74E-07
CNAG_02159	470.0899	0.498814	0.229996	2.168791	0.030099	0.063009
CNAG_07647	2223.3	1.00748	0.206681	4.874571	1.09E-06	7.46E-06
CNAG_02157	482.8242	-0.55206	0.185483	-2.97631	0.002917	0.008482
CNAG_02156	518.0397	1.381812	0.173092	7.983109	1.43E-15	4.20E-14
CNAG_02154	852.1378	-0.60835	0.245599	-2.47701	0.013249	0.031258
CNAG_02153	2942.735	-0.44479	0.106784	-4.1653	3.11E-05	0.000154

CNAG_02147	234.1744	-1.06861	0.196776	-5.43056	5.62E-08	5.10E-07
CNAG_02145	1685.283	-0.88335	0.206427	-4.27925	1.88E-05	9.80E-05
CNAG_02144	38037.47	-0.70306	0.125428	-5.60534	2.08E-08	2.07E-07
CNAG_02143	619.6033	-0.78088	0.163118	-4.78719	1.69E-06	1.12E-05
CNAG_02141	335.3481	-0.63937	0.21474	-2.97743	0.002907	0.008454
CNAG_02140	3549.763	-1.25784	0.182142	-6.90584	4.99E-12	9.20E-11
CNAG_02139	3431.901	0.495153	0.128128	3.864502	0.000111	0.000478
CNAG_12586	77.31952	0.629676	0.258671	2.434275	0.014922	0.034699
CNAG_02136	2226.857	-0.61599	0.217941	-2.82641	0.004707	0.012858
CNAG_02134	3343.11	-1.06952	0.167358	-6.39059	1.65E-10	2.38E-09
CNAG_02131	739.5518	-0.65474	0.191468	-3.41958	0.000627	0.002209
CNAG_02130	4271.552	-0.54453	0.180531	-3.01625	0.002559	0.007599
CNAG_02129	30364.43	1.887286	0.163443	11.54704	7.64E-31	7.50E-29
CNAG_02128	9814.781	-0.64884	0.218375	-2.97122	0.002966	0.008611
CNAG_02126	1069.11	0.532002	0.189101	2.813326	0.004903	0.013334
CNAG_02125	66.10956	0.697692	0.268891	2.594705	0.009467	0.023457
CNAG_02124	938.2335	0.466996	0.149349	3.126868	0.001767	0.005465
CNAG_02123	1294.12	-0.71186	0.140313	-5.07342	3.91E-07	2.93E-06
CNAG_02118	114.3025	1.730991	0.313543	5.520741	3.38E-08	3.26E-07
CNAG_02117	992.4913	0.343537	0.157792	2.177145	0.02947	0.0619
CNAG_02115	1577.534	-0.97388	0.201394	-4.83568	1.33E-06	8.94E-06
CNAG_12591	100.447	-1.12023	0.245982	-4.55411	5.26E-06	3.15E-05
CNAG_02114	2306.002	0.505791	0.220448	2.294383	0.021769	0.047915
CNAG_02109	2028.998	0.571505	0.132679	4.307422	1.65E-05	8.75E-05
CNAG_12593	281.4918	-2.1725	0.362965	-5.98543	2.16E-09	2.60E-08
CNAG_02103	857.0484	1.095099	0.293136	3.735811	0.000187	0.000764
CNAG_02102	91.24779	1.52971	0.51701	2.958765	0.003089	0.008886
CNAG_02101	495.2417	-0.49852	0.17907	-2.78396	0.00537	0.014455
CNAG_02100	13001.24	-0.70819	0.118642	-5.96909	2.39E-09	2.86E-08
CNAG_02099	12655.11	-0.37137	0.187122	-1.98465	0.047183	0.092549
CNAG_02096	340.6817	-0.49185	0.194067	-2.53443	0.011263	0.027227
CNAG_12594	37.18875	1.38309	0.385457	3.588184	0.000333	0.001269
CNAG_02094	3965.946	0.404212	0.117475	3.440829	0.00058	0.002064
CNAG_02093	1203.056	1.337419	0.167598	7.979932	1.46E-15	4.26E-14
CNAG_02089	853.5958	0.790814	0.161697	4.890714	1.00E-06	6.94E-06
CNAG_02086	2108.471	-0.75115	0.120979	-6.20897	5.33E-10	7.00E-09
CNAG_02084	3328.523	-1.15566	0.131173	-8.8102	1.25E-18	4.96E-17
CNAG_02083	1241.225	-1.35107	0.21722	-6.21981	4.98E-10	6.57E-09
CNAG_12598	47.7155	-0.88209	0.3981	-2.21574	0.026709	0.056929
CNAG_02082	508.6001	-1.14643	0.305162	-3.75679	0.000172	0.00071
CNAG_02079	12334.02	0.641063	0.160103	4.004058	6.23E-05	0.000285
CNAG_02076	2099.775	-0.54988	0.143191	-3.84021	0.000123	0.000524
CNAG_02075	904.1707	-0.63219	0.142145	-4.44748	8.69E-06	4.91E-05
CNAG_02073	1616.291	1.164868	0.202249	5.759571	8.43E-09	8.95E-08
CNAG_02072	1624.603	1.12569	0.192497	5.847816	4.98E-09	5.51E-08
CNAG_02070	10052.03	1.855101	0.256894	7.221265	5.15E-13	1.11E-11
CNAG_02069	2402.619	1.470178	0.243883	6.028212	1.66E-09	2.04E-08

CNAG_02067	1955.987	-0.68248	0.126232	-5.40656	6.42E-08	5.79E-07
CNAG_02061	293.1793	-0.56551	0.152979	-3.69666	0.000218	0.000873
CNAG_02060	1519.093	-2.73908	0.192216	-14.25	4.48E-46	1.02E-43
CNAG_12600	1157.295	2.15482	0.188181	11.45077	2.33E-30	2.23E-28
CNAG_02058	123.0927	1.640953	0.276088	5.943592	2.79E-09	3.26E-08
CNAG_02055	1183.595	0.967533	0.158259	6.113619	9.74E-10	1.24E-08
CNAG_02054	583.2989	-0.46519	0.139085	-3.34464	0.000824	0.002791
CNAG_02051	1506.924	-1.07552	0.159012	-6.76375	1.34E-11	2.31E-10
CNAG_02049	21.92868	1.003993	0.501763	2.000932	0.0454	0.089515
CNAG_02048	664.7199	-0.99806	0.147121	-6.78393	1.17E-11	2.02E-10
CNAG_02047	1248.582	1.561835	0.134066	11.64976	2.30E-31	2.29E-29
CNAG_02045	156.01	-0.58986	0.213071	-2.76836	0.005634	0.015088
CNAG_02043	220.3889	-0.64541	0.212056	-3.04359	0.002338	0.007015
CNAG_02041	1975.727	0.775523	0.198753	3.901949	9.54E-05	0.000418
CNAG_02040	322.1702	-0.67054	0.29167	-2.29896	0.021507	0.047419
CNAG_02038	2851.61	0.664251	0.119789	5.545195	2.94E-08	2.86E-07
CNAG_02037	2442.728	-0.57288	0.189527	-3.02266	0.002506	0.007454
CNAG_02034	782.8573	-0.80238	0.134025	-5.98678	2.14E-09	2.58E-08
CNAG_12605	116.0697	0.616949	0.228317	2.702159	0.006889	0.017877
CNAG_12606	34.4284	0.983077	0.396271	2.480822	0.013108	0.031008
CNAG_02029	1628.714	0.422914	0.1481	2.855598	0.004296	0.011868
CNAG_02028	3740.211	-0.7119	0.164666	-4.32327	1.54E-05	8.20E-05
CNAG_02026	361.3028	1.344719	0.203746	6.599967	4.11E-11	6.40E-10
CNAG_02025	1323.112	-0.73499	0.145819	-5.04042	4.65E-07	3.45E-06
CNAG_02024	1286.003	-1.12631	0.21884	-5.14672	2.65E-07	2.05E-06
CNAG_02023	299.6301	1.184342	0.219092	5.405678	6.46E-08	5.81E-07
CNAG_02020	1528.197	-0.66147	0.206004	-3.21096	0.001323	0.004249
CNAG_06525	138.0058	0.601639	0.258934	2.323527	0.020151	0.044851
CNAG_06533	2277.985	0.720263	0.131415	5.480817	4.23E-08	3.94E-07
CNAG_06534	5648.096	-0.80497	0.127873	-6.29504	3.07E-10	4.24E-09
CNAG_06535	2104.88	-0.73556	0.136688	-5.38131	7.39E-08	6.51E-07
CNAG_06539	45.95181	0.830966	0.311354	2.668878	0.007611	0.019414
CNAG_06541	6250.652	1.707607	0.174558	9.782438	1.34E-22	8.06E-21
CNAG_06544	12488.7	-0.7722	0.228847	-3.3743	0.00074	0.002544
CNAG_06545	3911.633	-1.22205	0.113451	-10.7716	4.69E-27	3.62E-25
CNAG_06549	893.5401	-0.35191	0.169815	-2.07234	0.038233	0.077422
CNAG_06553	1053.669	0.61535	0.192002	3.204914	0.001351	0.004322
CNAG_06554	835.9303	-0.74284	0.163211	-4.55144	5.33E-06	3.18E-05
CNAG_06555	1174.69	-0.81714	0.125048	-6.53458	6.38E-11	9.71E-10
CNAG_06556	818.9171	-0.82859	0.140606	-5.89302	3.79E-09	4.30E-08
CNAG_06558	1275.21	-0.47237	0.139226	-3.39282	0.000692	0.002399
CNAG_06559	843.6	1.114792	0.213059	5.232324	1.67E-07	1.37E-06
CNAG_06560	1065.645	0.709916	0.239188	2.968021	0.002997	0.008679
CNAG_06563	5061.062	-0.66883	0.190611	-3.5089	0.00045	0.001645
CNAG_06567	1757.241	1.152917	0.273175	4.220437	2.44E-05	0.000124
CNAG_06568	1734.082	-0.53015	0.235773	-2.24856	0.024541	0.053076
CNAG_07659	2609.657	0.625338	0.144375	4.331348	1.48E-05	7.94E-05

CNAG_07660	12015.25	-0.67051	0.188647	-3.55431	0.000379	0.001418
CNAG_06573	1543.178	0.600739	0.203123	2.957515	0.003101	0.008919
CNAG_06574	1063.601	1.335655	0.219069	6.09697	1.08E-09	1.37E-08
CNAG_12619	48.48895	0.815496	0.354561	2.300014	0.021447	0.0473
CNAG_06576	12160.93	2.675031	0.226822	11.79353	4.22E-32	4.59E-30
CNAG_06577	2208.615	2.990652	0.22218	13.46048	2.67E-41	4.82E-39
CNAG_06578	6823.399	0.982989	0.22966	4.280192	1.87E-05	9.77E-05
CNAG_07661	384.9758	0.874437	0.290028	3.015008	0.00257	0.007622
CNAG_12622	72.69106	1.794986	0.285891	6.278558	3.42E-10	4.67E-09
CNAG_06583	265.9428	-0.95048	0.180969	-5.25216	1.50E-07	1.24E-06
CNAG_06588	831.724	0.529223	0.221113	2.393448	0.016691	0.038155
CNAG_06589	1257.602	0.758542	0.207712	3.651897	0.00026	0.001021
CNAG_06593	535.9083	0.431694	0.155236	2.780888	0.005421	0.014562
CNAG_06598	90.20163	-3.15859	0.503254	-6.27633	3.47E-10	4.72E-09
CNAG_06599	792.8167	-0.40856	0.172888	-2.36317	0.018119	0.040996
CNAG_06600	4545.657	0.413603	0.132438	3.123003	0.00179	0.005531
CNAG_06602	3161.653	1.734175	0.260136	6.666421	2.62E-11	4.22E-10
CNAG_06604	816.0168	-0.54644	0.192538	-2.83809	0.004539	0.012466
CNAG_06605	49064.45	-0.89769	0.250977	-3.57677	0.000348	0.001316
CNAG_06606	2626.282	0.582829	0.199251	2.925104	0.003443	0.009775
CNAG_06608	1496.219	-0.4993	0.15234	-3.27753	0.001047	0.00345
CNAG_12629	209.9164	1.275261	0.280421	4.547674	5.42E-06	3.23E-05
CNAG_06609	37.87022	1.463612	0.396972	3.686938	0.000227	0.000903
CNAG_06610	235.6492	0.590453	0.217108	2.719626	0.006536	0.017094
CNAG_06614	811.4306	0.741527	0.183122	4.049362	5.14E-05	0.00024
CNAG_12632	80.80964	0.94997	0.297568	3.192444	0.001411	0.004487
CNAG_12633	31.6983	0.821325	0.364524	2.253145	0.02425	0.052547
CNAG_06616	1340.753	1.135175	0.190516	5.958419	2.55E-09	3.02E-08
CNAG_07664	2125.752	-0.42966	0.114183	-3.76288	0.000168	0.000694
CNAG_06621	5299.953	-0.75897	0.194696	-3.89824	9.69E-05	0.000424
CNAG_06623	903.034	0.720448	0.211569	3.405266	0.000661	0.002314
CNAG_06625	4335.564	0.64537	0.231165	2.791816	0.005241	0.014156
CNAG_06626	3986.662	-0.52071	0.155529	-3.348	0.000814	0.002769
CNAG_06627	2628.882	-0.40628	0.109654	-3.70509	0.000211	0.00085
CNAG_12636	21.96318	1.443999	0.447046	3.230093	0.001238	0.004005
CNAG_06629	31.28149	1.298195	0.388715	3.339708	0.000839	0.002835
CNAG_06630	4019.899	-0.75112	0.158109	-4.75067	2.03E-06	1.32E-05
CNAG_06631	2254.419	-0.31943	0.15182	-2.10402	0.035377	0.072487
CNAG_06632	2278.841	0.573575	0.122035	4.70007	2.60E-06	1.67E-05
CNAG_06633	37055.71	-0.91589	0.255578	-3.58358	0.000339	0.001285
CNAG_06634	917.6936	-0.24668	0.115034	-2.14438	0.032002	0.066446
CNAG_06635	3687.13	-0.45754	0.206448	-2.21627	0.026673	0.056883
CNAG_06636	219.9576	1.117521	0.183271	6.097641	1.08E-09	1.36E-08
CNAG_06638	10232.22	-0.99084	0.130593	-7.58725	3.27E-14	8.11E-13
CNAG_06641	1826.298	-0.97267	0.20117	-4.83506	1.33E-06	8.96E-06
CNAG_06643	548.5152	-0.64514	0.143731	-4.48856	7.17E-06	4.14E-05
CNAG_06644	3536.07	-0.83989	0.195817	-4.28916	1.79E-05	9.42E-05

CNAG_06645	2241.223	-0.96616	0.175548	-5.50369	3.72E-08	3.55E-07
CNAG_06648	2286.01	-0.35217	0.136002	-2.58948	0.009612	0.023764
CNAG_06650	209.1591	0.764112	0.177819	4.29714	1.73E-05	9.14E-05
CNAG_06652	206.044	0.679326	0.219349	3.097011	0.001955	0.005972
CNAG_06654	549.8244	-0.5877	0.205685	-2.85727	0.004273	0.011814
CNAG_06655	525.1391	0.795347	0.171242	4.644573	3.41E-06	2.13E-05
CNAG_06656	1613.589	-0.92023	0.221174	-4.16067	3.17E-05	0.000157
CNAG_06657	653.0325	0.44412	0.214451	2.070965	0.038362	0.077615
CNAG_06658	503.8034	1.297803	0.160117	8.105365	5.26E-16	1.63E-14
CNAG_06659	794.0542	0.662032	0.127867	5.177501	2.25E-07	1.78E-06
CNAG_06661	1712.887	-0.57354	0.142863	-4.01461	5.95E-05	0.000274
CNAG_12644	24.68528	1.232236	0.485793	2.536548	0.011195	0.027096
CNAG_06663	6462.542	-1.39128	0.315524	-4.40945	1.04E-05	5.75E-05
CNAG_06664	1153.357	0.539464	0.144082	3.744147	0.000181	0.000742
CNAG_06666	7406.11	1.051079	0.150597	6.979431	2.96E-12	5.63E-11
CNAG_06668	5591.367	1.863303	0.174523	10.67657	1.31E-26	9.64E-25
CNAG_06669	1859.369	-0.7123	0.246056	-2.89488	0.003793	0.010612
CNAG_06671	2649.705	0.457074	0.182584	2.503357	0.012302	0.029395
CNAG_12647	150.0606	0.874639	0.193926	4.510172	6.48E-06	3.80E-05
CNAG_06672	22.12899	1.380918	0.546772	2.525583	0.011551	0.027804
CNAG_06673	3097.601	0.558278	0.126087	4.427709	9.52E-06	5.35E-05
CNAG_06674	669.1995	-0.908	0.203043	-4.47197	7.75E-06	4.43E-05
CNAG_06679	3161.354	-0.73326	0.138215	-5.3052	1.13E-07	9.54E-07
CNAG_07177	2840.555	-1.2644	0.294167	-4.29825	1.72E-05	9.10E-05
CNAG_06684	467.4622	-0.91706	0.182924	-5.01335	5.35E-07	3.93E-06
CNAG_06685	2315.012	-0.3629	0.117041	-3.1006	0.001931	0.005911
CNAG_06690	2691.246	-0.71598	0.173603	-4.12425	3.72E-05	0.000181
CNAG_06692	806.4776	-0.52069	0.203741	-2.55566	0.010599	0.025866
CNAG_06693	689.4349	1.16279	0.156682	7.421348	1.16E-13	2.65E-12
CNAG_06694	640.5801	0.620171	0.193456	3.20575	0.001347	0.004311
CNAG_06695	486.9776	1.187783	0.17021	6.978325	2.99E-12	5.66E-11
CNAG_07990	995.296	-1.61292	0.156755	-10.2894	7.87E-25	5.43E-23
CNAG_12653	14.37475	-1.45651	0.52211	-2.78966	0.005276	0.014227
CNAG_06698	792.8623	0.993662	0.130697	7.602782	2.90E-14	7.24E-13
CNAG_06699	88531.13	-0.61364	0.125812	-4.87747	1.07E-06	7.37E-06
CNAG_05671	896.2535	0.689252	0.130446	5.28382	1.27E-07	1.06E-06
CNAG_05672	2264.693	-0.44684	0.160575	-2.78275	0.00539	0.014501
CNAG_05673	1422.148	0.320183	0.159204	2.011151	0.044309	0.087736
CNAG_07666	21.37283	1.226775	0.443843	2.763987	0.00571	0.015241
CNAG_05676	1061.783	2.833818	0.153334	18.48139	2.92E-76	1.36E-73
CNAG_07668	1097.274	0.593368	0.146648	4.04619	5.21E-05	0.000243
CNAG_05682	6975.13	0.912235	0.141348	6.453842	1.09E-10	1.61E-09
CNAG_05683	829.4698	3.093695	0.321528	9.621847	6.47E-22	3.64E-20
CNAG_05684	2305.541	0.76066	0.16607	4.580356	4.64E-06	2.81E-05
CNAG_12663	227.7349	1.596839	0.333036	4.794796	1.63E-06	1.08E-05
CNAG_05686	1161.365	1.228228	0.195009	6.298318	3.01E-10	4.16E-09
CNAG_05689	1397.393	-0.24997	0.120443	-2.0754	0.03795	0.076965

CNAG_05694	4523.308	-0.48749	0.122691	-3.97333	7.09E-05	0.000321
CNAG_12666	45.40922	0.839539	0.332929	2.521672	0.01168	0.028073
CNAG_05697	574.0339	0.619471	0.170213	3.639382	0.000273	0.001067
CNAG_05699	726.5972	-0.93414	0.227174	-4.112	3.92E-05	0.000189
CNAG_05700	2037.641	-0.31649	0.135425	-2.33698	0.01944	0.043513
CNAG_12667	129.5731	0.763325	0.37503	2.035367	0.041814	0.083481
CNAG_05702	1207.022	-0.29547	0.131517	-2.24661	0.024665	0.053242
CNAG_05705	428.3794	-0.72621	0.184681	-3.93224	8.42E-05	0.000375
CNAG_05707	978.2395	0.705181	0.170151	4.144451	3.41E-05	0.000167
CNAG_05711	711.4776	-0.56565	0.221305	-2.55597	0.010589	0.025858
CNAG_05718	577.7874	1.379094	0.161117	8.559566	1.13E-17	4.13E-16
CNAG_05719	1190.015	-0.87392	0.145573	-6.00329	1.93E-09	2.35E-08
CNAG_05723	709.8298	1.508511	0.154864	9.740904	2.02E-22	1.17E-20
CNAG_05724	996.8716	0.789852	0.177502	4.449822	8.59E-06	4.87E-05
CNAG_05725	49620.6	-0.82967	0.141401	-5.86749	4.42E-09	4.94E-08
CNAG_05726	1613.877	-0.64483	0.173649	-3.71344	0.000204	0.000825
CNAG_12671	30.61619	1.467149	0.389592	3.76586	0.000166	0.000687
CNAG_05732	2714.287	0.793934	0.255064	3.112689	0.001854	0.005697
CNAG_05734	2274.185	0.36934	0.173361	2.130467	0.033133	0.068383
CNAG_05735	223.4558	1.26619	0.202438	6.254703	3.98E-10	5.36E-09
CNAG_05736	67.64624	1.019388	0.29672	3.435521	0.000591	0.002101
CNAG_05737	1784.313	0.575939	0.155895	3.694406	0.00022	0.00088
CNAG_05738	108.4428	1.85424	0.378501	4.898901	9.64E-07	6.71E-06
CNAG_05739	115.6057	1.530752	0.276034	5.545522	2.93E-08	2.86E-07
CNAG_12673	66.88003	1.034155	0.308656	3.350508	0.000807	0.002746
CNAG_05742	2550.557	-1.29775	0.170482	-7.61224	2.69E-14	6.77E-13
CNAG_05745	3890.283	-0.50092	0.197301	-2.53887	0.011121	0.02695
CNAG_05746	124.5189	0.525657	0.251515	2.089963	0.036621	0.07469
CNAG_05747	996.7486	0.312099	0.119997	2.600883	0.009298	0.023082
CNAG_05748	1175.795	-0.47112	0.21953	-2.14605	0.031869	0.066238
CNAG_05749	684.0578	-0.62797	0.215976	-2.90757	0.003642	0.010271
CNAG_05750	71367.37	-0.45615	0.14269	-3.1968	0.00139	0.004426
CNAG_05751	1110.443	-0.45907	0.152036	-3.0195	0.002532	0.007524
CNAG_05752	1793.976	-1.24042	0.182757	-6.78728	1.14E-11	1.98E-10
CNAG_05753	10381.83	-0.57151	0.110066	-5.1924	2.08E-07	1.65E-06
CNAG_07671	582.2324	0.512644	0.225678	2.271567	0.023113	0.050551
CNAG_07672	1142.64	-0.64871	0.167783	-3.86637	0.00011	0.000475
CNAG_05755	1925.592	-1.22071	0.21922	-5.56845	2.57E-08	2.55E-07
CNAG_05756	1403.154	-0.65303	0.157346	-4.15028	3.32E-05	0.000163
CNAG_07673	21.33842	1.344967	0.530589	2.534856	0.011249	0.027203
CNAG_05757	518.5253	-0.78016	0.228475	-3.41465	0.000639	0.002245
CNAG_05759	11603.57	-0.31532	0.138909	-2.26996	0.02321	0.050722
CNAG_12676	197.1311	1.054742	0.243727	4.327553	1.51E-05	8.05E-05
CNAG_05762	44869.22	-0.96207	0.276061	-3.485	0.000492	0.001785
CNAG_05763	247.2849	-0.71018	0.166867	-4.25595	2.08E-05	0.000107
CNAG_05764	2738.582	-1.04641	0.227749	-4.59458	4.34E-06	2.66E-05
CNAG_05768	2381.426	0.396641	0.12536	3.16401	0.001556	0.004891

CNAG_05770	6812.55	0.608717	0.181931	3.345869	0.00082	0.002783
CNAG_05771	1756.242	0.988803	0.293755	3.366085	0.000762	0.002609
CNAG_05772	1024.101	0.302545	0.133382	2.268252	0.023314	0.050907
CNAG_05775	1023.196	0.477029	0.168837	2.825376	0.004723	0.012895
CNAG_05777	366.6272	-0.80624	0.223936	-3.60034	0.000318	0.001215
CNAG_05778	573.8333	0.632098	0.152443	4.146454	3.38E-05	0.000166
CNAG_05782	3080.889	-0.47253	0.141633	-3.33631	0.000849	0.002863
CNAG_05784	480.3418	0.340863	0.155031	2.198677	0.027901	0.059104
CNAG_12679	29.63195	-1.04188	0.400575	-2.60097	0.009296	0.023082
CNAG_05786	408.9752	0.337818	0.170637	1.979744	0.047732	0.093467
CNAG_05787	2180.14	-0.4049	0.182108	-2.22344	0.026186	0.05598
CNAG_05789	1680.004	-0.76997	0.168413	-4.57192	4.83E-06	2.92E-05
CNAG_05792	7339.643	0.706654	0.196541	3.595458	0.000324	0.001237
CNAG_07675	1068.365	0.566502	0.210656	2.689229	0.007162	0.018482
CNAG_05798	988.2102	-0.40085	0.158135	-2.53485	0.011249	0.027203
CNAG_05799	6904.03	0.576675	0.184694	3.122331	0.001794	0.005539
CNAG_05800	22237.37	-0.99061	0.240608	-4.1171	3.84E-05	0.000186
CNAG_05801	332.5277	-1.10036	0.163395	-6.73439	1.65E-11	2.76E-10
CNAG_05803	643.5944	-0.51298	0.147442	-3.47921	0.000503	0.001823
CNAG_05805	917.2634	-0.55627	0.184622	-3.01304	0.002586	0.007663
CNAG_05807	508.105	0.734728	0.147911	4.967367	6.79E-07	4.89E-06
CNAG_05810	468.2518	-0.87237	0.187415	-4.65475	3.24E-06	2.04E-05
CNAG_05811	780.2214	-0.75793	0.230807	-3.28381	0.001024	0.003391
CNAG_05813	702.9794	-1.23314	0.247486	-4.98269	6.27E-07	4.54E-06
CNAG_05814	29874.78	-0.9916	0.269806	-3.67525	0.000238	0.000941
CNAG_05815	205.4164	0.860898	0.217996	3.949149	7.84E-05	0.000352
CNAG_05816	1208.274	-0.53204	0.123949	-4.2924	1.77E-05	9.30E-05
CNAG_05818	1532.758	0.80636	0.234691	3.435841	0.000591	0.002099
CNAG_05819	766.1738	-0.59128	0.270004	-2.18991	0.028531	0.060214
CNAG_05820	704.8476	-0.77479	0.303418	-2.55355	0.010663	0.025983
CNAG_05823	352.8805	-0.58374	0.163508	-3.57007	0.000357	0.001344
CNAG_05824	791.2992	0.61726	0.134428	4.59176	4.40E-06	2.69E-05
CNAG_05825	3277.826	-0.66435	0.166415	-3.99216	6.55E-05	0.000299
CNAG_05827	4510.908	-0.71601	0.212379	-3.37136	0.000748	0.00257
CNAG_05833	78.3389	0.658734	0.284273	2.317256	0.02049	0.045478
CNAG_05836	1288.602	0.64645	0.130571	4.950956	7.38E-07	5.28E-06
CNAG_05837	5.521661	2.800809	0.992255	2.82267	0.004763	0.012991
CNAG_05839	8361.748	-1.12102	0.241319	-4.64539	3.39E-06	2.13E-05
CNAG_05840	783.1102	-0.32574	0.135141	-2.41035	0.015937	0.036735
CNAG_05847	8089.914	-0.4488	0.151534	-2.9617	0.003059	0.008818
CNAG_05848	5485.102	-0.59038	0.126268	-4.67563	2.93E-06	1.86E-05
CNAG_07676	12657.43	-0.56323	0.129356	-4.35413	1.34E-05	7.28E-05
CNAG_07677	529.5817	1.189864	0.159346	7.467154	8.19E-14	1.92E-12
CNAG_05853	443.8658	-1.59704	0.184213	-8.66956	4.34E-18	1.63E-16
CNAG_05854	553.2839	-0.58773	0.195487	-3.00651	0.002643	0.007809
CNAG_07678	131.2789	0.883691	0.231815	3.812058	0.000138	0.000581
CNAG_07679	1685.851	-0.88812	0.170516	-5.20842	1.90E-07	1.54E-06

CNAG_05858	836.1699	-0.72571	0.191135	-3.79683	0.000147	0.000614
CNAG_05859	751.6144	-0.43151	0.192183	-2.2453	0.024749	0.053395
CNAG_05860	897.5911	1.016498	0.19682	5.164606	2.41E-07	1.89E-06
CNAG_05861	179.3079	1.508648	0.288728	5.22516	1.74E-07	1.42E-06
CNAG_05862	820.3352	1.136888	0.162209	7.008765	2.40E-12	4.69E-11
CNAG_05863	767.6634	-1.51959	0.179971	-8.44354	3.08E-17	1.07E-15
CNAG_05864	4189.695	1.915438	0.153813	12.45306	1.35E-35	1.81E-33
CNAG_05866	471.2296	0.889058	0.252185	3.525422	0.000423	0.00156
CNAG_05867	336.8493	-1.8867	0.274465	-6.87411	6.24E-12	1.14E-10
CNAG_05870	546.9335	-0.97659	0.308152	-3.16918	0.001529	0.004818
CNAG_05871	1394.684	0.912337	0.298125	3.060248	0.002212	0.006687
CNAG_05872	4322.131	2.002612	0.252981	7.916049	2.45E-15	6.93E-14
CNAG_05873	197.8644	1.374271	0.271012	5.070891	3.96E-07	2.97E-06
CNAG_05874	708.0415	-0.37048	0.179746	-2.0611	0.039293	0.079044
CNAG_05875	1217.385	-1.1973	0.21029	-5.69357	1.24E-08	1.28E-07
CNAG_05876	293.8776	-1.22022	0.174677	-6.98556	2.84E-12	5.43E-11
CNAG_05881	3465.473	-0.37568	0.184729	-2.03369	0.041982	0.083673
CNAG_07680	728.8998	-0.70509	0.271752	-2.59459	0.00947	0.023458
CNAG_07681	1149.835	-0.4782	0.14869	-3.21606	0.0013	0.004188
CNAG_05884	5745.553	-0.42657	0.18584	-2.29539	0.021711	0.047801
CNAG_05886	2645.593	-0.40348	0.197778	-2.04004	0.041346	0.082755
CNAG_05887	1861.739	-0.42454	0.127569	-3.3279	0.000875	0.00294
CNAG_05888	3156.855	-0.57475	0.292893	-1.96233	0.049724	0.096801
CNAG_05889	1647.405	0.377942	0.12086	3.127092	0.001765	0.005463
CNAG_05891	518.9264	0.798258	0.173759	4.594057	4.35E-06	2.66E-05
CNAG_05892	3305.484	-0.80977	0.208518	-3.88343	0.000103	0.000447
CNAG_05894	2973.847	0.849778	0.235857	3.602929	0.000315	0.001205
CNAG_05896	1214.002	-0.75022	0.148972	-5.03598	4.75E-07	3.52E-06
CNAG_05899	3777.029	-1.27359	0.225855	-5.63896	1.71E-08	1.72E-07
CNAG_05900	8672.836	-0.62063	0.102147	-6.07585	1.23E-09	1.54E-08
CNAG_05901	507.3845	-0.51971	0.243134	-2.13756	0.032553	0.067438
CNAG_12695	66.51062	-1.33985	0.374097	-3.58156	0.000342	0.001294
CNAG_05904	25813.45	-1.07073	0.303401	-3.52909	0.000417	0.001543
CNAG_05905	1239.052	-0.2829	0.142486	-1.98543	0.047096	0.092425
CNAG_05907	20057.24	-0.757	0.143247	-5.28461	1.26E-07	1.05E-06
CNAG_05909	16315.97	-1.47587	0.22785	-6.47736	9.33E-11	1.40E-09
CNAG_05910	154.2908	0.457663	0.202003	2.265627	0.023474	0.051215
CNAG_05911	84.0884	-0.97455	0.468508	-2.08011	0.037516	0.076282
CNAG_12698	186.6623	-1.63071	0.278022	-5.86539	4.48E-09	5.00E-08
CNAG_05912	879.8823	0.614772	0.204829	3.001396	0.002687	0.007922
CNAG_05913	3922.837	-0.79648	0.147586	-5.39668	6.79E-08	6.07E-07
CNAG_05914	500.67	-1.81918	0.18357	-9.91001	3.77E-23	2.36E-21
CNAG_05915	662.662	1.956508	0.289378	6.761077	1.37E-11	2.34E-10
CNAG_05917	1385.218	-0.45389	0.151992	-2.98624	0.002824	0.008263
CNAG_05919	1076.041	-0.65868	0.183029	-3.59877	0.00032	0.001222
CNAG_05920	543.2724	-0.44754	0.142221	-3.14681	0.001651	0.005161
CNAG_05921	1165.947	0.663886	0.141997	4.675343	2.93E-06	1.86E-05

CNAG_12699	157.8095	1.442113	0.227758	6.331765	2.42E-10	3.40E-09
CNAG_12700	25.56107	1.411432	0.469284	3.00763	0.002633	0.007783
CNAG_05924	84.62874	1.190869	0.249182	4.779107	1.76E-06	1.16E-05
CNAG_05926	2051.95	-0.54632	0.148411	-3.68111	0.000232	0.000922
CNAG_05927	1388.556	-0.33707	0.114508	-2.94362	0.003244	0.009272
CNAG_05930	1651.716	0.41121	0.122085	3.368226	0.000757	0.002594
CNAG_05935	3426.141	-0.85002	0.289261	-2.93859	0.003297	0.00941
CNAG_05937	1007.262	0.483434	0.204902	2.359339	0.018308	0.041339
CNAG_05938	50.3443	0.861619	0.331746	2.597224	0.009398	0.023322
CNAG_05939	1280.762	3.534858	0.191091	18.49827	2.13E-76	1.06E-73
CNAG_12702	35.72721	1.508245	0.359325	4.197446	2.70E-05	0.000135
CNAG_05940	4212.066	1.66149	0.214	7.763958	8.23E-15	2.20E-13
CNAG_12703	138.8485	-0.68096	0.288618	-2.35938	0.018305	0.041339
CNAG_05941	178.7865	-0.64876	0.237896	-2.72707	0.00639	0.016791
CNAG_12704	619.4443	0.570927	0.273423	2.088074	0.036791	0.074998
CNAG_07683	328.2446	1.151944	0.412935	2.789651	0.005276	0.014227
CNAG_07684	228.6168	0.474767	0.229057	2.0727	0.0382	0.077374
CNAG_07687	92.05715	0.934988	0.22779	4.104601	4.05E-05	0.000195
CNAG_07688	1814.93	0.575288	0.15819	3.636697	0.000276	0.001077
CNAG_07689	3681.849	-0.51135	0.114849	-4.45239	8.49E-06	4.81E-05
CNAG_07690	312.3993	1.025034	0.412898	2.482533	0.013045	0.030887
CNAG_12707	15.69795	2.483605	0.562785	4.413061	1.02E-05	5.68E-05
CNAG_07188	222.9442	0.660622	0.195784	3.374232	0.00074	0.002544
CNAG_07693	12.81441	1.461383	0.639618	2.284777	0.022326	0.048992
CNAG_07701	573.3986	-0.48943	0.176384	-2.7748	0.005524	0.014823
CNAG_07702	187.639	-0.91709	0.199425	-4.59864	4.25E-06	2.62E-05
CNAG_05963	1246.938	0.395515	0.185263	2.134877	0.032771	0.067795
CNAG_05965	680.4448	0.59292	0.15259	3.885708	0.000102	0.000444
CNAG_05968	293.7265	-0.79369	0.197232	-4.02416	5.72E-05	0.000264
CNAG_05972	46.99	-1.62195	0.367798	-4.40991	1.03E-05	5.75E-05
CNAG_05973	244.3749	0.456523	0.218728	2.087173	0.036873	0.075145
CNAG_05976	4151.618	-0.63666	0.14397	-4.42216	9.77E-06	5.47E-05
CNAG_05977	6505.447	1.100081	0.318192	3.457284	0.000546	0.001958
CNAG_05978	7853.24	-0.4633	0.10487	-4.4179	9.97E-06	5.58E-05
CNAG_05979	946.052	-0.79063	0.162036	-4.87935	1.06E-06	7.32E-06
CNAG_05980	3739.746	-0.79182	0.205809	-3.84736	0.000119	0.00051
CNAG_05981	447.1017	-1.04977	0.201901	-5.19945	2.00E-07	1.61E-06
CNAG_05982	127.3196	0.830734	0.24478	3.393805	0.000689	0.002395
CNAG_06944	87.07363	-0.96067	0.273846	-3.50806	0.000451	0.001649
CNAG_03084	194.9222	1.232774	0.228628	5.392056	6.97E-08	6.18E-07
CNAG_03086	986.6133	-0.51267	0.153152	-3.34745	0.000816	0.002772
CNAG_03091	9.678931	1.491658	0.747642	1.995149	0.046027	0.090616
CNAG_07708	91.28168	1.265009	0.286401	4.416908	1.00E-05	5.59E-05
CNAG_03097	461.6247	-0.34729	0.137433	-2.527	0.011504	0.027718
CNAG_03098	2024.649	-0.66249	0.219066	-3.02414	0.002493	0.00742
CNAG_03100	1611.869	0.329997	0.132577	2.489094	0.012807	0.030423
CNAG_03106	1626.576	-1.13585	0.177764	-6.38967	1.66E-10	2.39E-09

CNAG_03107	16.54807	1.93974	0.523749	3.703568	0.000213	0.000854
CNAG_12716	42.75924	0.855477	0.319103	2.680886	0.007343	0.018845
CNAG_03108	956.3275	0.771746	0.143056	5.394712	6.86E-08	6.11E-07
CNAG_03109	5951.02	0.596946	0.110909	5.382293	7.35E-08	6.49E-07
CNAG_03110	5006.545	1.34442	0.171267	7.849841	4.17E-15	1.15E-13
CNAG_03113	9172.344	1.380596	0.219988	6.275774	3.48E-10	4.73E-09
CNAG_03114	4130.271	1.660831	0.211104	7.867355	3.62E-15	1.01E-13
CNAG_03115	883.0503	0.504531	0.129882	3.88453	0.000103	0.000445
CNAG_12720	73.50889	0.765119	0.314101	2.435898	0.014855	0.034564
CNAG_03116	2015.574	-0.42261	0.125535	-3.36648	0.000761	0.002608
CNAG_03122	318.9179	-1.12432	0.312017	-3.60339	0.000314	0.001204
CNAG_03125	420.212	-0.82926	0.304004	-2.7278	0.006376	0.01677
CNAG_03127	42700.78	-1.2065	0.308441	-3.91162	9.17E-05	0.000404
CNAG_03134	308.1252	-0.41525	0.188476	-2.20319	0.027581	0.058529
CNAG_03135	1163.408	0.568546	0.226348	2.51182	0.012011	0.028756
CNAG_03136	2622.319	-0.4516	0.172372	-2.61991	0.008795	0.02197
CNAG_12722	42.528	0.796191	0.322393	2.469624	0.013526	0.031825
CNAG_03138	1889.003	0.702133	0.20625	3.404286	0.000663	0.002319
CNAG_03139	6653.178	-0.24349	0.105263	-2.31315	0.020715	0.045884
CNAG_03140	13083.5	-0.24181	0.115971	-2.08509	0.037061	0.075432
CNAG_03141	195.1698	-0.53811	0.266613	-2.01831	0.043559	0.086466
CNAG_03142	18126	1.722636	0.230645	7.468762	8.10E-14	1.90E-12
CNAG_03143	16821.56	2.206082	0.29854	7.389562	1.47E-13	3.30E-12
CNAG_03145	44.9838	0.831118	0.292516	2.841272	0.004493	0.01235
CNAG_03148	2031.341	-0.84556	0.326459	-2.5901	0.009595	0.023742
CNAG_03150	967.2869	-0.49803	0.143156	-3.47896	0.000503	0.001824
CNAG_03154	626.2224	0.775359	0.214631	3.612513	0.000303	0.00117
CNAG_03159	945.1001	-0.49758	0.205004	-2.4272	0.015216	0.035322
CNAG_03160	473.466	0.934022	0.35009	2.667944	0.007632	0.019455
CNAG_03162	1476.38	0.932629	0.232968	4.003253	6.25E-05	0.000286
CNAG_03163	601.7208	0.683728	0.166243	4.112828	3.91E-05	0.000189
CNAG_03165	2427.872	-0.80428	0.179184	-4.48858	7.17E-06	4.14E-05
CNAG_03168	9085.733	-1.30395	0.157779	-8.26438	1.40E-16	4.65E-15
CNAG_12730	15.94837	1.306289	0.554717	2.354875	0.018529	0.041732
CNAG_03170	611.1578	-0.4091	0.156388	-2.61596	0.008898	0.022206
CNAG_03171	2270.114	0.354436	0.128414	2.760097	0.005778	0.015403
CNAG_03172	500.719	0.429305	0.155547	2.759971	0.005781	0.015403
CNAG_03173	2003.1	0.659773	0.180066	3.664062	0.000248	0.000978
CNAG_03176	6628.778	2.114982	0.138234	15.30002	7.64E-53	2.02E-50
CNAG_12732	73.3114	0.840403	0.289204	2.905918	0.003662	0.010318
CNAG_12733	156.7534	0.377571	0.187907	2.009349	0.0445	0.08796
CNAG_03178	1587.853	0.648737	0.29446	2.203139	0.027585	0.058529
CNAG_12734	311.2131	0.962388	0.214888	4.478556	7.51E-06	4.32E-05
CNAG_03181	37.58068	1.257834	0.358212	3.51142	0.000446	0.001632
CNAG_03183	1468.786	-0.95334	0.215543	-4.42297	9.74E-06	5.46E-05
CNAG_03184	1480.246	-1.39642	0.187977	-7.42869	1.10E-13	2.52E-12
CNAG_03185	1167.406	0.745977	0.229609	3.248903	0.001159	0.003773

CNAG_03186	2497.081	-0.53104	0.22784	-2.33075	0.019767	0.04412
CNAG_03187	497.6416	1.26592	0.308754	4.100094	4.13E-05	0.000198
CNAG_03189	1496.506	-0.40133	0.167877	-2.39062	0.01682	0.038406
CNAG_03193	43.31226	2.39601	0.453972	5.277881	1.31E-07	1.09E-06
CNAG_03195	222.8374	1.285436	0.166126	7.737721	1.01E-14	2.65E-13
CNAG_03196	2510.271	-1.00366	0.210351	-4.77136	1.83E-06	1.20E-05
CNAG_03197	172.349	0.506107	0.230588	2.194849	0.028174	0.059557
CNAG_03198	46401	-1.07352	0.247175	-4.34317	1.40E-05	7.60E-05
CNAG_07717	4518.096	0.459414	0.185433	2.477521	0.01323	0.031241
CNAG_03202	2188.555	0.348692	0.136493	2.554642	0.01063	0.025917
CNAG_03204	491.303	0.985813	0.181737	5.424389	5.82E-08	5.27E-07
CNAG_03205	1478.291	-1.00239	0.160788	-6.23421	4.54E-10	6.02E-09
CNAG_03206	670.7081	-0.82691	0.18691	-4.42413	9.68E-06	5.43E-05
CNAG_03211	48.76455	0.692569	0.333985	2.073649	0.038112	0.077235
CNAG_03212	680.1161	-1.46457	0.244993	-5.978	2.26E-09	2.71E-08
CNAG_03213	4390.977	0.936178	0.207511	4.511456	6.44E-06	3.78E-05
CNAG_03215	352.0165	1.108621	0.165341	6.705051	2.01E-11	3.33E-10
CNAG_07719	8083.101	0.630485	0.118349	5.327358	9.97E-08	8.57E-07
CNAG_03221	9105.899	-1.01772	0.262984	-3.86989	0.000109	0.000469
CNAG_03226	3516.585	-1.69422	0.263592	-6.42743	1.30E-10	1.90E-09
CNAG_12740	54.05898	-1.47756	0.409138	-3.6114	0.000305	0.001174
CNAG_03230	160.9057	2.396291	0.215687	11.11004	1.12E-28	9.28E-27
CNAG_03232	1884.784	1.346777	0.1646	8.182121	2.79E-16	8.90E-15
CNAG_03233	146.6599	0.669894	0.238723	2.806161	0.005014	0.013606
CNAG_03234	96.22928	1.355778	0.252439	5.370721	7.84E-08	6.89E-07
CNAG_03238	155.1646	0.553139	0.253455	2.182392	0.029081	0.061228
CNAG_03239	597.5709	-1.11764	0.176651	-6.32682	2.50E-10	3.49E-09
CNAG_03240	3185.605	0.53258	0.184463	2.887186	0.003887	0.010839
CNAG_03242	335.2912	0.405952	0.162082	2.504612	0.012259	0.029313
CNAG_03246	1294.117	-0.35095	0.127118	-2.76082	0.005766	0.015374
CNAG_03247	3325.49	0.492898	0.224399	2.196531	0.028054	0.059365
CNAG_03248	3014.735	0.310761	0.11469	2.709582	0.006737	0.017545
CNAG_03250	1003.96	0.935963	0.173674	5.389186	7.08E-08	6.28E-07
CNAG_03259	111.1711	1.167877	0.237602	4.915259	8.87E-07	6.21E-06
CNAG_03260	1340.438	-0.69761	0.15033	-4.64054	3.48E-06	2.17E-05
CNAG_03261	3317.734	-0.46292	0.156359	-2.96064	0.00307	0.008835
CNAG_03264	1635.896	-0.47601	0.13489	-3.5289	0.000417	0.001543
CNAG_03266	7108.337	-0.3429	0.149828	-2.28866	0.022099	0.048535
CNAG_03267	4121.853	-0.64344	0.164412	-3.91361	9.09E-05	0.000402
CNAG_03268	5014.449	0.650187	0.217295	2.992191	0.00277	0.008129
CNAG_03270	4170.582	-0.70933	0.119529	-5.93443	2.95E-09	3.43E-08
CNAG_03275	748.3796	-0.36344	0.148575	-2.44617	0.014438	0.033743
CNAG_03276	328.6787	-0.56748	0.250516	-2.26524	0.023498	0.05125
CNAG_03277	221.7017	-0.58879	0.247829	-2.37578	0.017512	0.039746
CNAG_12750	60.05136	0.724836	0.307932	2.353881	0.018579	0.041819
CNAG_03279	418.8433	-0.39825	0.16259	-2.44944	0.014308	0.033488
CNAG_03280	834.2109	-0.6985	0.142548	-4.90012	9.58E-07	6.67E-06

CNAG_03282	1877.523	0.477581	0.176472	2.706275	0.006804	0.017686
CNAG_03283	33321.77	-1.07029	0.295215	-3.62545	0.000288	0.00112
CNAG_03284	970.171	-0.95449	0.128884	-7.40574	1.30E-13	2.94E-12
CNAG_03285	1893.317	-0.54955	0.127977	-4.29413	1.75E-05	9.25E-05
CNAG_03290	1494.604	0.409568	0.156161	2.622735	0.008723	0.02183
CNAG_03291	1323.558	-0.43621	0.154739	-2.81897	0.004818	0.013119
CNAG_12751	32.30105	1.07117	0.428418	2.500292	0.012409	0.029584
CNAG_07722	4.98819	2.500707	1.194505	2.093509	0.036304	0.074119
CNAG_03292	17.394	1.707265	0.487084	3.50507	0.000456	0.001665
CNAG_03293	1379.121	-0.88134	0.216636	-4.06828	4.74E-05	0.000223
CNAG_03294	287.1748	0.536385	0.161097	3.329581	0.00087	0.002926
CNAG_03296	680.6673	-0.86526	0.148559	-5.82437	5.73E-09	6.26E-08
CNAG_03298	4225.239	-0.8225	0.261945	-3.13996	0.00169	0.005257
CNAG_03300	1687.363	-0.67809	0.127044	-5.33749	9.42E-08	8.14E-07
CNAG_03302	1800.577	-0.75726	0.129556	-5.84502	5.07E-09	5.59E-08
CNAG_03303	13591.11	-0.83202	0.187366	-4.44062	8.97E-06	5.06E-05
CNAG_12753	155.757	0.829738	0.230418	3.601017	0.000317	0.001213
CNAG_03307	29.96698	1.730749	0.47851	3.616956	0.000298	0.001152
CNAG_03308	1087.438	0.602788	0.212492	2.836756	0.004557	0.012505
CNAG_03310	711.3058	-0.62413	0.147921	-4.21935	2.45E-05	0.000124
CNAG_03311	6246.677	-1.22135	0.157658	-7.74687	9.42E-15	2.49E-13
CNAG_03314	1089.046	0.44878	0.193914	2.314324	0.02065	0.045757
CNAG_03316	2437.492	0.739548	0.207486	3.564332	0.000365	0.001369
CNAG_03318	204.1021	0.502257	0.18311	2.742934	0.006089	0.016112
CNAG_03319	3489.558	-0.35895	0.162496	-2.20897	0.027177	0.057802
CNAG_03321	2644.047	0.260022	0.122963	2.114634	0.034461	0.070793
CNAG_03322	8583.657	-1.03479	0.234388	-4.41486	1.01E-05	5.64E-05
CNAG_03326	2478.631	1.493089	0.119059	12.54072	4.47E-36	6.23E-34
CNAG_03327	47.98833	0.734747	0.309472	2.374199	0.017587	0.039894
CNAG_03328	2303.059	0.740424	0.126658	5.845846	5.04E-09	5.57E-08
CNAG_03330	414.7591	0.438693	0.221191	1.983326	0.047331	0.092771
CNAG_03331	2633.287	-0.37597	0.108334	-3.4705	0.000519	0.001876
CNAG_03333	2336.615	1.400019	0.17786	7.87145	3.51E-15	9.77E-14
CNAG_03335	2638.873	-0.56613	0.134903	-4.19657	2.71E-05	0.000135
CNAG_03336	2988.563	0.502879	0.186524	2.696052	0.007017	0.018167
CNAG_03337	1357.564	0.687643	0.157727	4.359704	1.30E-05	7.11E-05
CNAG_03338	795.2477	-0.58664	0.18793	-3.12156	0.001799	0.005549
CNAG_03340	62.92256	0.667747	0.263952	2.529805	0.011413	0.027547
CNAG_03341	1970.636	-0.52535	0.192613	-2.72749	0.006382	0.01678
CNAG_03342	2463.4	-0.66767	0.125958	-5.30077	1.15E-07	9.76E-07
CNAG_03343	1032.082	-0.50579	0.148935	-3.39607	0.000684	0.002377
CNAG_03345	6340.71	1.197844	0.371726	3.222382	0.001271	0.004105
CNAG_07723	41.77314	1.60818	0.465981	3.451173	0.000558	0.001998
CNAG_03347	4478.361	0.916507	0.12368	7.410287	1.26E-13	2.86E-12
CNAG_03349	3958.776	-0.3399	0.1554	-2.18726	0.028723	0.060572
CNAG_03353	2203.111	-0.72258	0.197685	-3.65521	0.000257	0.001009
CNAG_03355	5624.413	0.537707	0.191305	2.81073	0.004943	0.013432

CNAG_03357	1126.707	-0.52189	0.135992	-3.83763	0.000124	0.000528
CNAG_03359	2186.011	-0.90018	0.302072	-2.98001	0.002882	0.008408
CNAG_03360	1801.494	-0.45779	0.229735	-1.99267	0.046297	0.091059
CNAG_03363	829.6426	-0.39662	0.120653	-3.28727	0.001012	0.003354
CNAG_03364	750.9439	-0.45363	0.139985	-3.24056	0.001193	0.00388
CNAG_03365	3015.714	0.270526	0.121243	2.231268	0.025663	0.055025
CNAG_03366	431.7754	1.167151	0.167051	6.986802	2.81E-12	5.40E-11
CNAG_12761	153.8575	-0.66142	0.320538	-2.06347	0.039068	0.078711
CNAG_12762	47.30185	1.045067	0.305739	3.418173	0.00063	0.002219
CNAG_03372	2788.886	0.777917	0.215907	3.603014	0.000315	0.001205
CNAG_03374	127.8355	0.736601	0.250488	2.940664	0.003275	0.009351
CNAG_03376	1530.637	0.517641	0.240437	2.152921	0.031325	0.06521
CNAG_03377	755.3989	-0.63137	0.21388	-2.95197	0.003158	0.009057
CNAG_03378	2280.813	-0.48867	0.162613	-3.00513	0.002655	0.007842
CNAG_03381	536.7999	0.599228	0.183304	3.269039	0.001079	0.003539
CNAG_03382	3061.649	0.885213	0.210739	4.200526	2.66E-05	0.000134
CNAG_03384	40.61589	1.123566	0.337563	3.328463	0.000873	0.002937
CNAG_03385	1974.469	1.823443	0.221115	8.246592	1.63E-16	5.33E-15
CNAG_03386	3627.81	-0.74577	0.200811	-3.7138	0.000204	0.000824
CNAG_03387	373.1175	-0.4122	0.171821	-2.399	0.01644	0.037646
CNAG_03389	7905.537	-0.59143	0.142535	-4.14932	3.33E-05	0.000164
CNAG_03390	1128.894	-0.286	0.123966	-2.30704	0.021052	0.046545
CNAG_03391	818.1959	-0.48163	0.138874	-3.46811	0.000524	0.001889
CNAG_03392	715.327	0.915702	0.202673	4.518119	6.24E-06	3.67E-05
CNAG_03394	407.5347	-0.65208	0.218894	-2.97897	0.002892	0.008424
CNAG_03395	1517.391	-0.83008	0.224961	-3.68989	0.000224	0.000894
CNAG_03397	4552.307	0.645432	0.126397	5.106376	3.28E-07	2.50E-06
CNAG_03398	61.96981	-2.19039	0.441406	-4.96231	6.97E-07	5.00E-06
CNAG_03400	1137.878	1.555945	0.162705	9.56296	1.14E-21	6.23E-20
CNAG_03401	1434.525	-0.33945	0.145264	-2.33677	0.019451	0.043526
CNAG_03403	832.8278	-0.4805	0.162777	-2.95188	0.003158	0.009057
CNAG_03404	354.4324	0.665562	0.215664	3.086103	0.002028	0.006184
CNAG_03405	2972.283	0.824776	0.107899	7.643989	2.11E-14	5.33E-13
CNAG_03406	574.6689	-0.57509	0.175868	-3.27001	0.001075	0.003528
CNAG_12767	97.78523	1.057574	0.41347	2.557805	0.010534	0.025746
CNAG_03408	8196.069	2.248837	0.261348	8.604768	7.65E-18	2.81E-16
CNAG_03412	1379.517	0.680646	0.151126	4.503842	6.67E-06	3.90E-05
CNAG_03413	1270.619	-0.27543	0.139211	-1.9785	0.047872	0.09367
CNAG_03416	1765.824	-0.40817	0.167311	-2.43959	0.014704	0.034293
CNAG_03417	655.7264	0.401035	0.140891	2.846416	0.004421	0.012182
CNAG_03420	1851.292	0.582348	0.18349	3.173727	0.001505	0.004747
CNAG_03424	149.1596	-0.9608	0.427636	-2.24678	0.024654	0.053235
CNAG_03426	1352.439	-1.67728	0.178989	-9.37086	7.19E-21	3.60E-19
CNAG_07732	743.0702	0.473711	0.15947	2.970537	0.002973	0.008624
CNAG_03432	836.4701	0.551455	0.124673	4.423199	9.73E-06	5.45E-05
CNAG_03433	2971.971	-0.52129	0.206878	-2.5198	0.011742	0.028188
CNAG_03435	6578.224	-0.61321	0.205676	-2.98142	0.002869	0.008375

CNAG_03436	3480.687	-0.45541	0.136173	-3.34437	0.000825	0.002791
CNAG_12769	73.32976	0.899393	0.348333	2.581993	0.009823	0.024241
CNAG_03437	155.9288	0.78383	0.270237	2.900527	0.003725	0.010464
CNAG_12770	1069.224	-0.91882	0.198991	-4.6174	3.89E-06	2.40E-05
CNAG_03439	8045.292	-0.35666	0.165436	-2.15589	0.031092	0.064777
CNAG_03441	1291.924	-0.68961	0.130148	-5.29866	1.17E-07	9.85E-07
CNAG_03442	583.0863	-0.61398	0.157354	-3.90193	9.54E-05	0.000418
CNAG_03445	720.6091	1.379318	0.158211	8.718206	2.83E-18	1.08E-16
CNAG_03447	1062.443	-0.77133	0.287657	-2.68143	0.007331	0.018827
CNAG_03448	1964.906	-0.43219	0.164922	-2.62058	0.008778	0.021934
CNAG_03452	1220.699	-0.41596	0.124288	-3.34677	0.000818	0.002777
CNAG_03453	954.0932	-1.1471	0.249658	-4.59467	4.33E-06	2.66E-05
CNAG_12772	23.34025	1.353314	0.486408	2.782259	0.005398	0.014516
CNAG_03454	2303.418	0.59105	0.221035	2.674015	0.007495	0.019156
CNAG_12773	135.504	1.765257	0.279611	6.31327	2.73E-10	3.79E-09
CNAG_03455	330.0205	1.14507	0.225428	5.079537	3.78E-07	2.86E-06
CNAG_03456	1848.68	-0.91765	0.208637	-4.39833	1.09E-05	6.03E-05
CNAG_03457	7994.009	-0.65972	0.130741	-5.04604	4.51E-07	3.36E-06
CNAG_03458	1891.118	-0.38881	0.145342	-2.67514	0.00747	0.019115
CNAG_03463	3419.655	0.313994	0.117379	2.675045	0.007472	0.019115
CNAG_12776	234.7642	-0.67783	0.281444	-2.40839	0.016023	0.036872
CNAG_07736	447.9161	-1.57114	0.181105	-8.67527	4.13E-18	1.55E-16
CNAG_12780	936.1566	-0.59896	0.21396	-2.79942	0.005119	0.01386
CNAG_03473	358.8328	-0.59397	0.199133	-2.98279	0.002856	0.008341
CNAG_03475	2277.265	0.72279	0.176033	4.105987	4.03E-05	0.000194
CNAG_03476	8094.926	-0.63483	0.114647	-5.53722	3.07E-08	2.99E-07
CNAG_03477	183.3122	-0.90841	0.277076	-3.27855	0.001043	0.003442
CNAG_12781	139.6914	-1.46978	0.328639	-4.47231	7.74E-06	4.43E-05
CNAG_12782	45.94104	0.810058	0.30222	2.680356	0.007354	0.018869
CNAG_03480	715.042	1.407007	0.138116	10.18713	2.26E-24	1.55E-22
CNAG_03481	559.0382	-1.25338	0.176379	-7.10622	1.19E-12	2.45E-11
CNAG_03483	1807.146	-0.90756	0.194253	-4.67206	2.98E-06	1.89E-05
CNAG_03484	1109.723	-1.21442	0.272269	-4.46036	8.18E-06	4.65E-05
CNAG_03485	2065.263	0.594415	0.117497	5.05898	4.22E-07	3.15E-06
CNAG_03492	8366.198	3.513742	0.186522	18.8382	3.67E-79	2.08E-76
CNAG_03493	710.559	0.385213	0.132647	2.904036	0.003684	0.010373
CNAG_03494	1859.015	-0.33817	0.128543	-2.63076	0.008519	0.02141
CNAG_12785	19.49327	2.079844	0.540322	3.849266	0.000118	0.000507
CNAG_12786	584.8094	1.995378	0.285257	6.995012	2.65E-12	5.14E-11
CNAG_03498	3537.951	1.221782	0.290926	4.199633	2.67E-05	0.000134
CNAG_03499	414.4909	0.986516	0.148868	6.62678	3.43E-11	5.43E-10
CNAG_12788	54.37729	1.695886	0.309183	5.485059	4.13E-08	3.85E-07
CNAG_03500	356.0445	-0.40914	0.16083	-2.54394	0.010961	0.026627
CNAG_12789	50.52344	1.345752	0.354803	3.792961	0.000149	0.000621
CNAG_03502	3126.712	0.652816	0.14815	4.406462	1.05E-05	5.83E-05
CNAG_03503	1232.537	0.837828	0.231554	3.61828	0.000297	0.001147
CNAG_03504	235.9506	1.077616	0.171499	6.283495	3.31E-10	4.54E-09

CNAG_03507	11897.22	-0.97373	0.227166	-4.28644	1.82E-05	9.52E-05
CNAG_03509	6840.05	-0.61874	0.220876	-2.80128	0.00509	0.01379
CNAG_03510	20067.33	-0.6658	0.22013	-3.02459	0.00249	0.007415
CNAG_03512	1466.19	-0.57048	0.191377	-2.98094	0.002874	0.008386
CNAG_03513	1292.831	-1.25861	0.237491	-5.29959	1.16E-07	9.81E-07
CNAG_07740	4909.034	-0.2737	0.130672	-2.09457	0.036209	0.073983
CNAG_07741	2287.757	0.263738	0.130612	2.019251	0.043461	0.086314
CNAG_03517	6071.724	0.789251	0.151432	5.211907	1.87E-07	1.52E-06
CNAG_12792	20.94023	-2.00167	0.636694	-3.14384	0.001667	0.005204
CNAG_03519	30.89492	0.838084	0.355636	2.356579	0.018444	0.041588
CNAG_03521	124.7561	-1.13629	0.298072	-3.81214	0.000138	0.000581
CNAG_03524	1841.778	-0.96381	0.234804	-4.10472	4.05E-05	0.000195
CNAG_03525	1345.852	1.370477	0.172095	7.963483	1.67E-15	4.83E-14
CNAG_03526	197.396	0.953192	0.242649	3.928276	8.56E-05	0.000381
CNAG_07995	2349.143	0.801077	0.120727	6.635419	3.24E-11	5.14E-10
CNAG_03534	593.1612	0.664315	0.222448	2.98639	0.002823	0.008262
CNAG_07745	10582.03	0.680746	0.207674	3.277964	0.001046	0.003447
CNAG_07746	19935.57	-1.87158	0.226604	-8.25923	1.47E-16	4.83E-15
CNAG_12794	409.5044	-0.83391	0.305592	-2.72884	0.006356	0.016723
CNAG_03538	322.7807	-1.45338	0.389396	-3.7324	0.00019	0.000772
CNAG_03539	738.4286	-1.00851	0.228647	-4.41078	1.03E-05	5.73E-05
CNAG_03540	1187.125	0.561682	0.129187	4.347829	1.37E-05	7.46E-05
CNAG_03542	1605.488	-0.37421	0.141499	-2.6446	0.008179	0.020677
CNAG_12795	172.4731	0.434493	0.176804	2.457489	0.013991	0.032833
CNAG_03543	1854.749	0.520872	0.185458	2.808569	0.004976	0.013509
CNAG_03544	5098.413	-0.90498	0.167606	-5.39943	6.69E-08	6.00E-07
CNAG_03548	36.72053	1.032344	0.372967	2.767924	0.005641	0.015103
CNAG_03550	294.6315	-0.64718	0.149234	-4.33669	1.45E-05	7.80E-05
CNAG_03552	627.5558	-0.58559	0.237017	-2.47068	0.013486	0.03175
CNAG_03553	6016.494	0.344715	0.144878	2.379342	0.017344	0.039425
CNAG_03557	1195.447	0.501505	0.181326	2.765766	0.005679	0.015173
CNAG_03560	244.7795	0.330974	0.165579	1.998889	0.04562	0.089883
CNAG_03562	425.166	-0.8499	0.219548	-3.87112	0.000108	0.000467
CNAG_03563	5482.062	1.818982	0.18143	10.02579	1.17E-23	7.52E-22
CNAG_03564	2016.574	1.598746	0.215462	7.420089	1.17E-13	2.66E-12
CNAG_03565	5681.452	2.371709	0.234706	10.10503	5.25E-24	3.48E-22
CNAG_03566	11820	2.075669	0.234203	8.862674	7.81E-19	3.18E-17
CNAG_12800	131.6413	0.516543	0.240339	2.149226	0.031617	0.065731
CNAG_03569	23.21226	1.572984	0.595779	2.640214	0.008285	0.0209
CNAG_03570	64.20211	1.404117	0.297257	4.723581	2.32E-06	1.50E-05
CNAG_12801	42.61394	2.167359	0.369827	5.860468	4.62E-09	5.12E-08
CNAG_03572	6252.863	2.67118	0.190028	14.05677	7.00E-45	1.50E-42
CNAG_03573	750.7462	0.478272	0.182223	2.624643	0.008674	0.021742
CNAG_12804	21.46813	2.636907	0.589468	4.473364	7.70E-06	4.41E-05
CNAG_03575	804.5943	0.581226	0.160891	3.612538	0.000303	0.00117
CNAG_03577	41013.46	-0.93654	0.183156	-5.11334	3.17E-07	2.42E-06
CNAG_03578	785.6439	-0.92984	0.15777	-5.89361	3.78E-09	4.29E-08

CNAG_07749	951.0989	-0.45228	0.160414	-2.81947	0.00481	0.013108
CNAG_03584	2300.639	-0.36483	0.128601	-2.83689	0.004556	0.012504
CNAG_03587	647.9181	-0.65548	0.250828	-2.61326	0.008968	0.02236
CNAG_03588	8573.218	-0.80582	0.124122	-6.49215	8.46E-11	1.27E-09
CNAG_03589	1374.964	-0.54755	0.160591	-3.40961	0.000651	0.002279
CNAG_03590	2805.792	-0.50381	0.141215	-3.56766	0.00036	0.001353
CNAG_03592	3809.196	-0.79859	0.128366	-6.22123	4.93E-10	6.52E-09
CNAG_12806	22.25701	2.720156	0.54382	5.001944	5.68E-07	4.15E-06
CNAG_12807	41.79633	1.347596	0.390325	3.452497	0.000555	0.001989
CNAG_06977	75.66666	-0.69354	0.302559	-2.29226	0.021891	0.048104
CNAG_06993	3820.488	0.498323	0.123405	4.038106	5.39E-05	0.00025
CNAG_06994	1170.964	0.568114	0.130193	4.363623	1.28E-05	7.00E-05
CNAG_06966	62.89035	1.388564	0.288842	4.807341	1.53E-06	1.02E-05
CNAG_06968	2180.263	-0.6289	0.262774	-2.3933	0.016698	0.038159
CNAG_04101	2290.372	-0.88139	0.158292	-5.56813	2.57E-08	2.55E-07
CNAG_12810	22.6395	6.032422	1.084286	5.563498	2.64E-08	2.61E-07
CNAG_04103	452.5193	-1.59826	0.163121	-9.79799	1.15E-22	7.02E-21
CNAG_04104	1001.829	-0.59287	0.148494	-3.99254	6.54E-05	0.000299
CNAG_04105	7130.739	2.418903	0.276806	8.738632	2.36E-18	9.10E-17
CNAG_07755	161.0579	1.448299	0.2428	5.964992	2.45E-09	2.91E-08
CNAG_04106	4118.491	3.035452	0.247146	12.28204	1.13E-34	1.45E-32
CNAG_04107	506.5762	1.2234	0.224618	5.446572	5.13E-08	4.70E-07
CNAG_04110	851.5525	0.670481	0.174564	3.840899	0.000123	0.000523
CNAG_04111	2410.893	-0.30167	0.121431	-2.48428	0.012981	0.030772
CNAG_12812	88.08804	1.500562	0.27392	5.478111	4.30E-08	4.00E-07
CNAG_04112	815.9357	0.953621	0.161192	5.916074	3.30E-09	3.81E-08
CNAG_04114	27651.76	-1.11877	0.283575	-3.94524	7.97E-05	0.000357
CNAG_07756	1369.656	-1.20999	0.162262	-7.457	8.85E-14	2.05E-12
CNAG_04115	928.2815	-0.64483	0.153111	-4.21151	2.54E-05	0.000128
CNAG_04117	803.5507	0.905755	0.158147	5.727283	1.02E-08	1.07E-07
CNAG_12814	24.38939	1.272439	0.400242	3.17917	0.001477	0.004668
CNAG_04120	2406.598	-0.33613	0.124826	-2.69277	0.007086	0.018317
CNAG_04122	336.6849	-0.34089	0.153843	-2.21583	0.026703	0.056929
CNAG_12816	94.28141	0.851252	0.248251	3.428991	0.000606	0.002141
CNAG_04129	113.2616	0.696495	0.217225	3.206325	0.001344	0.004304
CNAG_04130	2072.306	-0.68684	0.208108	-3.3004	0.000965	0.003218
CNAG_04132	2824.204	-0.62893	0.150708	-4.17317	3.00E-05	0.000149
CNAG_07757	1573.024	0.666467	0.262617	2.537794	0.011155	0.027025
CNAG_04136	1782.163	-0.81859	0.129227	-6.33447	2.38E-10	3.35E-09
CNAG_12820	64.16834	-1.5564	0.286809	-5.42662	5.74E-08	5.21E-07
CNAG_04141	3675.421	-0.57224	0.127204	-4.49863	6.84E-06	3.98E-05
CNAG_04142	1378.345	-0.72593	0.198876	-3.65018	0.000262	0.001027
CNAG_07759	22.71913	-3.30293	0.655429	-5.03934	4.67E-07	3.47E-06
CNAG_04146	2139.206	-0.5847	0.168734	-3.46523	0.00053	0.001907
CNAG_04147	1009.633	-0.53433	0.136429	-3.91653	8.98E-05	0.000399
CNAG_04148	1427.623	-0.70677	0.225213	-3.13822	0.0017	0.00528
CNAG_04149	2016.607	-0.5898	0.114824	-5.13658	2.80E-07	2.16E-06

CNAG_12822	19.02869	1.320499	0.519193	2.543368	0.010979	0.026654
CNAG_04156	2729.947	-0.85295	0.212195	-4.01963	5.83E-05	0.000269
CNAG_04157	785.542	-0.68941	0.158836	-4.34042	1.42E-05	7.68E-05
CNAG_04159	1647.857	0.728104	0.322359	2.258671	0.023904	0.051944
CNAG_04163	7519.364	2.141726	0.251636	8.511199	1.72E-17	6.08E-16
CNAG_04167	229.4743	0.882497	0.16974	5.1991	2.00E-07	1.61E-06
CNAG_04168	4803.182	-1.3609	0.195327	-6.96729	3.23E-12	6.07E-11
CNAG_04175	2011.076	-1.19563	0.17719	-6.74772	1.50E-11	2.54E-10
CNAG_04177	1380.155	0.327963	0.122077	2.686535	0.00722	0.018602
CNAG_04178	493.1591	-0.93322	0.171148	-5.45272	4.96E-08	4.55E-07
CNAG_04179	8429.776	-0.69311	0.122365	-5.66427	1.48E-08	1.50E-07
CNAG_04180	2238.544	-0.60666	0.16998	-3.56902	0.000358	0.001347
CNAG_04181	1209.874	-0.90553	0.210911	-4.29341	1.76E-05	9.27E-05
CNAG_12827	1275.048	-0.9495	0.299751	-3.16763	0.001537	0.004838
CNAG_04182	1817.888	-0.70017	0.212949	-3.28798	0.001009	0.003347
CNAG_04183	19507.13	-0.50443	0.165203	-3.05342	0.002262	0.006818
CNAG_07997	74.0655	0.932458	0.309606	3.011759	0.002597	0.00769
CNAG_04185	1700.335	0.59424	0.228155	2.604547	0.0092	0.022872
CNAG_04186	1569.486	-0.88755	0.206962	-4.28847	1.80E-05	9.44E-05
CNAG_04187	2068.828	0.886482	0.133823	6.624267	3.49E-11	5.50E-10
CNAG_12829	66.00899	1.798567	0.381135	4.718981	2.37E-06	1.53E-05
CNAG_04188	1701.805	0.681196	0.216967	3.139629	0.001692	0.005261
CNAG_04189	7187.697	-1.46683	0.155352	-9.44198	3.66E-21	1.88E-19
CNAG_04190	5041.428	-0.75882	0.133516	-5.68334	1.32E-08	1.36E-07
CNAG_04192	5918.783	-0.61805	0.124551	-4.96219	6.97E-07	5.00E-06
CNAG_04195	2370.614	2.044103	0.165571	12.3458	5.13E-35	6.69E-33
CNAG_04197	2022.645	0.645661	0.230345	2.803018	0.005063	0.01372
CNAG_04199	326.8415	0.818004	0.260612	3.138777	0.001697	0.005274
CNAG_04200	878.344	1.233418	0.321398	3.837669	0.000124	0.000528
CNAG_04201	90.11558	0.610962	0.242923	2.515041	0.011902	0.028537
CNAG_04203	1835.164	0.40515	0.188882	2.144995	0.031953	0.066362
CNAG_04204	1218.2	-0.935	0.218754	-4.27421	1.92E-05	9.99E-05
CNAG_04205	1563.633	-0.57466	0.130201	-4.41362	1.02E-05	5.66E-05
CNAG_04206	1557.928	1.184503	0.199514	5.936942	2.90E-09	3.38E-08
CNAG_12830	65.74125	1.310281	0.338268	3.873499	0.000107	0.000464
CNAG_04208	1938.175	0.513381	0.124941	4.108981	3.97E-05	0.000191
CNAG_04210	2237.148	-0.41044	0.168351	-2.43797	0.01477	0.034427
CNAG_04212	2078.43	-0.51165	0.229271	-2.23163	0.025639	0.055002
CNAG_04215	15206.26	-0.93301	0.162675	-5.73542	9.73E-09	1.02E-07
CNAG_04219	3894.344	-0.41894	0.182727	-2.2927	0.021865	0.048061
CNAG_04220	1562.149	0.408667	0.133147	3.069281	0.002146	0.00651
CNAG_04222	3031.586	-0.94866	0.143251	-6.62235	3.54E-11	5.56E-10
CNAG_04224	2288.838	-0.64028	0.238828	-2.68092	0.007342	0.018845
CNAG_04225	145.1467	0.916036	0.202931	4.514024	6.36E-06	3.74E-05
CNAG_04227	1126.67	-0.92446	0.255087	-3.62408	0.00029	0.001124
CNAG_04228	460.1911	1.127675	0.157906	7.141432	9.24E-13	1.94E-11
CNAG_04231	349.5866	-0.71753	0.20469	-3.50544	0.000456	0.001664

CNAG_04235	1553.226	-0.59405	0.188516	-3.15119	0.001626	0.005091
CNAG_04236	2767.568	-0.91383	0.13516	-6.76106	1.37E-11	2.34E-10
CNAG_04239	326.0677	-0.81032	0.163946	-4.94262	7.71E-07	5.48E-06
CNAG_04242	502.6002	-0.7782	0.208742	-3.72804	0.000193	0.000784
CNAG_12834	74.55906	1.246645	0.273199	4.563139	5.04E-06	3.03E-05
CNAG_04244	1231.29	0.619617	0.225413	2.748801	0.005981	0.015859
CNAG_04245	7021.923	-0.9591	0.112151	-8.55191	1.21E-17	4.39E-16
CNAG_04248	1192.516	0.862232	0.171929	5.01506	5.30E-07	3.90E-06
CNAG_07763	894.5105	0.992244	0.148082	6.700618	2.08E-11	3.41E-10
CNAG_04253	669.0648	0.438009	0.176088	2.487449	0.012866	0.030536
CNAG_04255	337.8184	-0.50346	0.210446	-2.39235	0.016741	0.038247
CNAG_04256	529.8846	1.478979	0.183185	8.073683	6.82E-16	2.09E-14
CNAG_04257	4242.46	-0.53483	0.139503	-3.83379	0.000126	0.000536
CNAG_04259	5143.838	-0.50215	0.18686	-2.68732	0.007203	0.01857
CNAG_04260	2046.563	-0.41074	0.156405	-2.62612	0.008636	0.021662
CNAG_04261	276.948	-0.76721	0.225833	-3.39722	0.000681	0.002369
CNAG_04262	326.1466	0.469873	0.175732	2.673804	0.0075	0.019161
CNAG_04263	533.9701	-1.27946	0.199604	-6.41	1.46E-10	2.11E-09
CNAG_04266	1045.185	-0.6186	0.133308	-4.64035	3.48E-06	2.17E-05
CNAG_04268	845.9737	0.88338	0.19761	4.470311	7.81E-06	4.46E-05
CNAG_04269	1867.26	0.470735	0.136779	3.441563	0.000578	0.002059
CNAG_04270	824.0834	0.685367	0.149088	4.597055	4.29E-06	2.63E-05
CNAG_04276	3368.559	-0.72582	0.10599	-6.84801	7.49E-12	1.34E-10
CNAG_04278	1305.875	-0.41192	0.18755	-2.19634	0.028067	0.059378
CNAG_04280	130.3603	0.566753	0.267822	2.116154	0.034332	0.070546
CNAG_04283	2729.47	0.814737	0.130596	6.238618	4.41E-10	5.89E-09
CNAG_12840	235.142	1.704955	0.214979	7.930812	2.18E-15	6.25E-14
CNAG_04284	4466.405	-0.27723	0.116667	-2.37622	0.017491	0.03971
CNAG_04288	1379.689	1.068911	0.288643	3.703226	0.000213	0.000854
CNAG_04289	3703.571	-0.61982	0.14544	-4.26166	2.03E-05	0.000105
CNAG_04290	3681.986	-0.61226	0.295845	-2.06951	0.038498	0.077779
CNAG_04292	2752.343	-0.58497	0.214592	-2.72595	0.006412	0.016837
CNAG_04293	1649.173	0.789414	0.289914	2.72293	0.006471	0.016952
CNAG_04294	401.5228	0.51679	0.161111	3.207668	0.001338	0.004286
CNAG_04295	771.1678	-0.46464	0.138319	-3.3592	0.000782	0.00267
CNAG_04297	481.5277	-0.80336	0.195829	-4.10233	4.09E-05	0.000196
CNAG_04307	450.5155	-0.40281	0.178968	-2.25077	0.0244	0.052815
CNAG_04309	315.8449	0.416757	0.191578	2.175392	0.029601	0.062118
CNAG_04310	1371.064	-0.92515	0.122835	-7.53164	5.01E-14	1.22E-12
CNAG_04313	524.5698	1.694864	0.176581	9.598212	8.13E-22	4.55E-20
CNAG_04314	4321.687	1.473956	0.146563	10.0568	8.57E-24	5.63E-22
CNAG_04315	3142.176	0.69	0.148959	4.63215	3.62E-06	2.25E-05
CNAG_04317	83.14973	0.596892	0.246548	2.420993	0.015478	0.035815
CNAG_04320	3157.98	0.658592	0.180831	3.642036	0.00027	0.001057
CNAG_04321	2534.753	0.58931	0.174348	3.380077	0.000725	0.002498
CNAG_04322	4593.431	2.411687	0.221143	10.90555	1.08E-27	8.57E-26
CNAG_04323	3202.761	0.474797	0.20833	2.279068	0.022663	0.049664

CNAG_04325	3095.169	3.478768	0.146233	23.78924	4.32E-125	1.71E-121
CNAG_04326	1483.119	0.766839	0.144512	5.306407	1.12E-07	9.49E-07
CNAG_12843	24.03689	1.271629	0.48456	2.624295	0.008683	0.021744
CNAG_04331	44.74372	1.548874	0.368981	4.19771	2.70E-05	0.000135
CNAG_04332	907.713	-0.4111	0.153631	-2.67592	0.007452	0.019084
CNAG_04333	73.36433	1.164522	0.322754	3.608079	0.000308	0.001186
CNAG_04334	2030.927	-0.63836	0.129925	-4.91334	8.95E-07	6.27E-06
CNAG_04338	917.4034	1.165952	0.195654	5.959244	2.53E-09	3.01E-08
CNAG_04339	2162.929	0.520372	0.170166	3.05802	0.002228	0.006729
CNAG_04340	2011.129	-0.36547	0.137589	-2.65625	0.007902	0.020047
CNAG_04343	251.4318	0.624419	0.238778	2.615058	0.008921	0.02225
CNAG_04345	817.3422	-0.44936	0.181159	-2.48049	0.01312	0.031027
CNAG_04347	5367.18	-1.01086	0.133176	-7.59035	3.19E-14	7.95E-13
CNAG_04348	1919.825	-0.91646	0.183019	-5.00746	5.52E-07	4.05E-06
CNAG_12847	6.407591	2.428589	0.86124	2.819875	0.004804	0.013096
CNAG_04350	845.3031	0.690584	0.138784	4.975968	6.49E-07	4.69E-06
CNAG_04351	556.6744	0.994588	0.141405	7.033609	2.01E-12	3.99E-11
CNAG_04353	1162.174	0.867409	0.158472	5.473582	4.41E-08	4.09E-07
CNAG_04354	1355.85	0.501194	0.147722	3.392826	0.000692	0.002399
CNAG_04356	1952.985	-0.32229	0.126073	-2.55635	0.010578	0.025838
CNAG_04361	6388.428	0.7448	0.183812	4.051961	5.08E-05	0.000238
CNAG_04362	4037.028	-0.29395	0.12255	-2.39865	0.016456	0.037671
CNAG_04363	69.05449	1.576803	0.300212	5.25229	1.50E-07	1.24E-06
CNAG_04364	6487.994	0.351248	0.105253	3.337191	0.000846	0.002855
CNAG_04365	4974.926	-0.63985	0.116326	-5.5005	3.79E-08	3.61E-07
CNAG_04367	366.3704	-0.74158	0.150188	-4.93767	7.91E-07	5.60E-06
CNAG_04368	1336.038	-0.37559	0.131334	-2.85982	0.004239	0.011723
CNAG_04371	1029.603	0.687212	0.18344	3.746246	0.00018	0.000737
CNAG_04372	1908.259	-0.78045	0.278194	-2.80542	0.005025	0.013633
CNAG_12849	10.2376	2.470761	0.733379	3.36901	0.000754	0.002589
CNAG_04375	2308.956	-0.56945	0.176274	-3.23051	0.001236	0.004003
CNAG_04376	2740.168	-0.89834	0.174468	-5.14906	2.62E-07	2.04E-06
CNAG_04378	3367.027	-0.586	0.230284	-2.54467	0.010938	0.026586
CNAG_04380	1157.844	0.591378	0.164708	3.590462	0.00033	0.00126
CNAG_04383	1328.643	-0.46367	0.196343	-2.36151	0.018201	0.041156
CNAG_04386	1412.366	1.944664	0.208905	9.308861	1.29E-20	6.38E-19
CNAG_04388	4301.498	0.912477	0.170379	5.355569	8.53E-08	7.45E-07
CNAG_07766	1010.517	1.224383	0.216375	5.658621	1.53E-08	1.55E-07
CNAG_07765	858.9353	3.822831	0.228971	16.69569	1.41E-62	6.22E-60
CNAG_04392	538.9923	0.850214	0.169023	5.030174	4.90E-07	3.62E-06
CNAG_04394	41.13837	1.311003	0.409338	3.202736	0.001361	0.004349
CNAG_04395	1592.628	-1.16024	0.200392	-5.78984	7.05E-09	7.58E-08
CNAG_04397	629.6212	-0.44685	0.170118	-2.62673	0.008621	0.02163
CNAG_04399	1107.207	-0.4429	0.171011	-2.58991	0.0096	0.023742
CNAG_04400	1258.815	-1.12374	0.243965	-4.60614	4.10E-06	2.53E-05
CNAG_04402	1749.823	-0.91714	0.16857	-5.44067	5.31E-08	4.84E-07
CNAG_04404	94.79903	0.598062	0.282514	2.11693	0.034266	0.070428

CNAG_04405	1289.449	0.389692	0.131263	2.968781	0.00299	0.008664
CNAG_04408	1758.719	-0.46947	0.110022	-4.26706	1.98E-05	0.000102
CNAG_04412	862.2095	1.11865	0.225036	4.970978	6.66E-07	4.81E-06
CNAG_04413	36.72698	3.022681	0.44955	6.723791	1.77E-11	2.94E-10
CNAG_04414	1845.424	0.886403	0.119303	7.429832	1.09E-13	2.50E-12
CNAG_04415	198.236	1.238673	0.249903	4.956612	7.17E-07	5.13E-06
CNAG_04416	519.3414	4.496399	0.223893	20.08279	1.04E-89	9.21E-87
CNAG_07767	54.53371	1.540597	0.337576	4.563701	5.03E-06	3.03E-05
CNAG_04417	491.1069	0.629406	0.134332	4.685442	2.79E-06	1.78E-05
CNAG_12852	173.1047	0.754919	0.274559	2.749565	0.005967	0.015837
CNAG_07769	3808.89	0.969518	0.328881	2.94793	0.003199	0.009157
CNAG_04433	1606.335	0.536722	0.228302	2.350931	0.018727	0.04207
CNAG_04434	888.1219	-1.27716	0.182604	-6.99417	2.67E-12	5.16E-11
CNAG_04436	998.0949	-0.59039	0.177722	-3.32201	0.000894	0.002996
CNAG_04437	805.8678	-0.66791	0.20952	-3.1878	0.001434	0.004542
CNAG_04438	1018.546	-0.49817	0.130949	-3.8043	0.000142	0.000598
CNAG_04440	647.5042	-0.50811	0.207237	-2.45183	0.014213	0.033305
CNAG_04441	18171.47	-0.72587	0.154704	-4.69202	2.71E-06	1.73E-05
CNAG_04443	2396.583	1.289846	0.161625	7.980495	1.46E-15	4.26E-14
CNAG_04445	34970.77	-0.74505	0.196298	-3.79548	0.000147	0.000617
CNAG_04446	157.5928	0.669466	0.232421	2.880408	0.003972	0.011042
CNAG_04447	730.1736	-0.57513	0.156992	-3.66345	0.000249	0.00098
CNAG_04448	53001.49	-0.91345	0.210896	-4.33131	1.48E-05	7.94E-05
CNAG_04451	1165.073	-0.52559	0.128817	-4.08013	4.50E-05	0.000213
CNAG_04452	1048.456	-0.81472	0.227141	-3.58685	0.000335	0.001274
CNAG_04453	844.8054	-0.34484	0.134972	-2.55491	0.010622	0.025905
CNAG_04454	13.35447	1.46579	0.534502	2.742349	0.0061	0.016124
CNAG_04455	309.9925	0.542509	0.183575	2.955244	0.003124	0.008973
CNAG_04456	812.9594	0.334405	0.168957	1.979234	0.04779	0.093554
CNAG_04457	60.58762	1.692044	0.306876	5.513778	3.51E-08	3.37E-07
CNAG_04459	54.84426	2.732116	0.405142	6.743608	1.55E-11	2.61E-10
CNAG_04460	2892.787	-0.4533	0.108936	-4.16117	3.17E-05	0.000156
CNAG_04462	374.7069	0.369521	0.166678	2.216979	0.026625	0.056825
CNAG_04465	2954.858	-0.59607	0.203418	-2.93026	0.003387	0.009639
CNAG_04466	463.5234	0.981358	0.200764	4.888108	1.02E-06	7.02E-06
CNAG_04469	365.2721	-0.6715	0.24052	-2.79186	0.005241	0.014156
CNAG_04470	1013.564	-1.16756	0.237207	-4.92212	8.56E-07	6.02E-06
CNAG_04471	689.5265	-0.50296	0.177012	-2.84141	0.004491	0.012349
CNAG_04473	1148.426	-0.63126	0.15703	-4.01999	5.82E-05	0.000269
CNAG_04474	214.3697	0.844546	0.172473	4.896685	9.75E-07	6.75E-06
CNAG_04476	101.1503	1.044857	0.293618	3.55856	0.000373	0.001396
CNAG_07771	2497.865	0.273504	0.124995	2.188115	0.028661	0.060457
CNAG_04478	3449.722	0.333258	0.151693	2.196922	0.028026	0.059338
CNAG_04479	471.6854	-1.21838	0.175367	-6.94758	3.72E-12	6.92E-11
CNAG_07772	144.7322	0.781972	0.192756	4.056794	4.98E-05	0.000234
CNAG_04485	7240.922	0.327474	0.156094	2.09793	0.035911	0.073487
CNAG_04486	61.02837	0.686836	0.303535	2.262789	0.023649	0.051511

CNAG_07775	1606.814	1.712925	0.285157	6.00696	1.89E-09	2.30E-08
CNAG_07777	238.357	-0.59528	0.181425	-3.28116	0.001034	0.003414
CNAG_04493	747.459	-0.50873	0.146544	-3.47151	0.000518	0.00187
CNAG_04494	1842.541	-0.46988	0.21731	-2.16225	0.030599	0.063934
CNAG_04496	793.9003	-1.26891	0.21651	-5.86078	4.61E-09	5.12E-08
CNAG_04497	665.1585	-0.52085	0.149144	-3.49227	0.000479	0.001738
CNAG_04501	3818.458	-0.49984	0.134988	-3.70288	0.000213	0.000854
CNAG_04503	742.5382	-0.45719	0.219764	-2.08035	0.037493	0.076273
CNAG_04505	2311.657	0.601186	0.241315	2.491295	0.012728	0.030253
CNAG_12867	36.69069	2.36022	0.402643	5.86182	4.58E-09	5.10E-08
CNAG_04506	68.12763	1.392824	0.279279	4.987216	6.13E-07	4.45E-06
CNAG_04507	728.038	1.118541	0.226033	4.948571	7.48E-07	5.33E-06
CNAG_04512	451.0593	-0.38883	0.197624	-1.9675	0.049125	0.095768
CNAG_04513	2402.751	-0.35561	0.176838	-2.01092	0.044333	0.087748
CNAG_04515	1727.997	-0.55613	0.146752	-3.78957	0.000151	0.000627
CNAG_12869	155.5881	-1.91896	0.321853	-5.96223	2.49E-09	2.96E-08
CNAG_04518	663.8676	-0.53301	0.154336	-3.45357	0.000553	0.001983
CNAG_04519	334.3014	-0.52879	0.200893	-2.63217	0.008484	0.021334
CNAG_04520	427.4157	-0.31019	0.137654	-2.25342	0.024232	0.052523
CNAG_04521	375.7199	1.37311	0.177477	7.736847	1.02E-14	2.66E-13
CNAG_04522	1293.56	-0.66766	0.186798	-3.57421	0.000351	0.001328
CNAG_04523	108.5481	0.747968	0.285015	2.624308	0.008683	0.021744
CNAG_04524	1392.208	0.803397	0.214612	3.743494	0.000181	0.000744
CNAG_04528	194.7188	-1.10077	0.260503	-4.22558	2.38E-05	0.000121
CNAG_04529	427.8862	-1.02063	0.252412	-4.0435	5.27E-05	0.000245
CNAG_07778	7406.647	-0.56113	0.105623	-5.31259	1.08E-07	9.21E-07
CNAG_04534	201.998	-1.09929	0.222777	-4.93451	8.04E-07	5.68E-06
CNAG_04535	78.15657	-0.76105	0.267584	-2.84415	0.004453	0.012252
CNAG_04536	36.6286	-1.15119	0.365099	-3.1531	0.001615	0.005062
CNAG_04539	1079.957	0.357022	0.173422	2.05869	0.039524	0.079488
CNAG_04541	1232.917	-0.39857	0.155957	-2.55565	0.010599	0.025866
CNAG_04546	62.03755	1.520756	0.298584	5.093227	3.52E-07	2.67E-06
CNAG_12873	118.7335	1.41054	0.221231	6.375862	1.82E-10	2.59E-09
CNAG_07780	941.9218	0.864166	0.143657	6.015489	1.79E-09	2.20E-08
CNAG_07781	1097.806	0.971869	0.276232	3.518312	0.000434	0.001593
CNAG_06998	13337.54	-0.37455	0.169739	-2.20662	0.02734	0.058087
CNAG_06999	1286.754	1.664299	0.176353	9.437293	3.83E-21	1.95E-19
CNAG_12876	476.986	-0.61206	0.211617	-2.8923	0.003824	0.010689
CNAG_07784	735.872	1.398085	0.221746	6.304909	2.88E-10	3.99E-09
CNAG_07002	144.1876	0.707921	0.288867	2.450686	0.014258	0.033392
CNAG_04931	2.241939	3.505295	1.584259	2.212577	0.026927	0.057331
CNAG_04926	102.8055	0.607875	0.244549	2.485695	0.01293	0.030662
CNAG_04925	1410.134	-0.40851	0.168145	-2.42953	0.015118	0.035126
CNAG_04922	257.2537	0.882087	0.165103	5.342634	9.16E-08	7.94E-07
CNAG_04921	47.5342	1.480119	0.56591	2.615466	0.008911	0.02223
CNAG_04920	2373.494	2.132758	0.198571	10.74053	6.57E-27	5.02E-25
CNAG_07839	43385.38	-1.00518	0.268778	-3.73981	0.000184	0.000753

CNAG_04914	6049.696	-0.66808	0.302597	-2.20782	0.027257	0.057942
CNAG_04911	543.5153	0.617895	0.183016	3.376174	0.000735	0.002528
CNAG_07840	28.82336	1.478802	0.535591	2.761066	0.005761	0.015367
CNAG_04908	736.1696	0.421523	0.152174	2.770007	0.005606	0.015017
CNAG_04907	89.91186	0.789338	0.266487	2.96201	0.003056	0.008812
CNAG_04906	4401.702	0.321462	0.142713	2.252509	0.02429	0.05262
CNAG_04905	41.09815	-1.82023	0.385169	-4.72579	2.29E-06	1.49E-05
CNAG_04904	13509.41	0.263919	0.109729	2.405183	0.016164	0.037143
CNAG_04903	3289.99	4.016241	0.212593	18.89167	1.34E-79	8.16E-77
CNAG_04902	2776.11	0.496636	0.173896	2.855937	0.004291	0.01186
CNAG_04901	360.0345	-1.02283	0.173221	-5.90477	3.53E-09	4.05E-08
CNAG_04900	1629.873	-0.71636	0.132497	-5.40664	6.42E-08	5.79E-07
CNAG_04899	1524.979	-0.7241	0.162868	-4.44591	8.75E-06	4.94E-05
CNAG_04898	145.4048	1.061039	0.207636	5.110103	3.22E-07	2.46E-06
CNAG_04896	1257.197	-0.40533	0.120415	-3.3661	0.000762	0.002609
CNAG_04894	182.0871	1.489238	0.207483	7.177651	7.09E-13	1.51E-11
CNAG_04891	86.3423	2.161455	0.302923	7.13533	9.66E-13	2.01E-11
CNAG_04889	1047.584	1.031278	0.185064	5.57255	2.51E-08	2.49E-07
CNAG_04887	159.4706	0.683424	0.292751	2.334491	0.01957	0.043767
CNAG_04886	1319.138	1.424848	0.153038	9.31039	1.27E-20	6.33E-19
CNAG_12883	56.6491	1.86334	0.343987	5.416895	6.06E-08	5.48E-07
CNAG_04884	28130.58	-1.1492	0.310874	-3.69668	0.000218	0.000873
CNAG_04883	27845.7	-0.7357	0.236452	-3.1114	0.001862	0.005719
CNAG_12884	28.7377	0.995551	0.463179	2.149388	0.031604	0.065722
CNAG_04882	1359.947	-0.32079	0.139442	-2.30052	0.021419	0.047263
CNAG_04880	429.7505	0.605965	0.240984	2.514548	0.011919	0.028568
CNAG_04879	6961.109	0.923634	0.255848	3.610091	0.000306	0.001179
CNAG_12885	122.0404	-2.6812	0.903044	-2.96907	0.002987	0.008659
CNAG_04877	1424.001	-5.98492	1.321154	-4.53007	5.90E-06	3.48E-05
CNAG_04876	2144.667	-0.42155	0.187589	-2.24721	0.024627	0.053204
CNAG_04875	992.1489	-0.66694	0.187378	-3.55936	0.000372	0.001393
CNAG_04874	863.8176	-1.11336	0.202522	-5.49748	3.85E-08	3.65E-07
CNAG_04873	203.0658	-0.57667	0.180816	-3.18927	0.001426	0.004528
CNAG_04872	1777.077	-0.80255	0.139849	-5.73871	9.54E-09	1.00E-07
CNAG_04871	436.7681	-0.4653	0.178448	-2.60746	0.009122	0.022707
CNAG_12886	86.59126	-0.7778	0.24838	-3.13149	0.001739	0.005388
CNAG_04869	2021.02	-1.61064	0.167833	-9.59672	8.25E-22	4.59E-20
CNAG_04868	913.7871	-0.33405	0.134685	-2.48023	0.01313	0.031041
CNAG_04867	701.1543	1.008302	0.217913	4.627083	3.71E-06	2.30E-05
CNAG_04865	76.59406	0.621422	0.264692	2.347712	0.018889	0.042399
CNAG_04864	3599.543	-0.6048	0.155889	-3.87966	0.000105	0.000453
CNAG_12889	95.56136	-1.51286	0.33477	-4.51909	6.21E-06	3.66E-05
CNAG_04862	5522.086	-1.10038	0.324784	-3.38802	0.000704	0.002434
CNAG_12890	15.03917	-1.30059	0.53379	-2.43653	0.014829	0.034544
CNAG_04861	1575.512	1.369571	0.138103	9.917001	3.51E-23	2.21E-21
CNAG_04860	2485.265	0.923273	0.145897	6.328234	2.48E-10	3.46E-09
CNAG_04859	930.3518	-0.85463	0.160329	-5.33045	9.80E-08	8.43E-07

CNAG_04857	3326.293	0.58734	0.184088	3.19053	0.00142	0.004512
CNAG_04853	1042.25	0.518293	0.21084	2.458229	0.013962	0.032776
CNAG_04851	26576.07	1.173573	0.12412	9.455129	3.23E-21	1.66E-19
CNAG_12892	197.1012	1.11471	0.238151	4.68068	2.86E-06	1.82E-05
CNAG_04849	705.9836	1.011578	0.283407	3.569346	0.000358	0.001347
CNAG_04845	1389.625	-0.37771	0.172407	-2.19078	0.028468	0.060097
CNAG_04843	981.2917	-0.60725	0.160296	-3.78832	0.000152	0.00063
CNAG_04840	6444.59	-0.57153	0.279203	-2.047	0.040659	0.081584
CNAG_04839	848.3073	-0.96597	0.20048	-4.81828	1.45E-06	9.70E-06
CNAG_04837	173.0774	-1.38375	0.19741	-7.00952	2.39E-12	4.68E-11
CNAG_04835	155.1204	0.537067	0.221301	2.426864	0.01523	0.035339
CNAG_04833	1772.232	1.260234	0.174777	7.210524	5.57E-13	1.20E-11
CNAG_04831	259.9463	1.05037	0.18197	5.772231	7.82E-09	8.33E-08
CNAG_04830	761.4839	-0.66146	0.153411	-4.31166	1.62E-05	8.59E-05
CNAG_04828	9849.897	0.427795	0.206546	2.071185	0.038342	0.077601
CNAG_04824	644.7034	-0.97066	0.280429	-3.46136	0.000537	0.001933
CNAG_04817	1251.888	-0.69646	0.223929	-3.11019	0.00187	0.005738
CNAG_04816	800.9765	1.146789	0.190846	6.008962	1.87E-09	2.28E-08
CNAG_07845	89.51658	-1.11435	0.29932	-3.72293	0.000197	0.000797
CNAG_04815	1826.904	0.55146	0.203573	2.708899	0.006751	0.017576
CNAG_12898	561.3834	-0.99761	0.276692	-3.60549	0.000312	0.001197
CNAG_04809	1405.428	0.354045	0.131493	2.692501	0.007092	0.018323
CNAG_12900	198.6281	-0.82281	0.323673	-2.54209	0.011019	0.026735
CNAG_04805	1488.592	-0.45113	0.121293	-3.71939	0.0002	0.000807
CNAG_04803	7488.824	0.54711	0.24562	2.227464	0.025916	0.055492
CNAG_04802	1218.362	-0.54436	0.132971	-4.0938	4.24E-05	0.000203
CNAG_04800	6828.782	-0.51388	0.098867	-5.1977	2.02E-07	1.61E-06
CNAG_04799	41178.63	-1.22683	0.28887	-4.24701	2.17E-05	0.000111
CNAG_04798	3932.397	-0.61175	0.163608	-3.73915	0.000185	0.000755
CNAG_04797	2591.185	-0.43026	0.107841	-3.9898	6.61E-05	0.000301
CNAG_04794	2474.587	2.075434	0.245324	8.459957	2.67E-17	9.36E-16
CNAG_04793	35.80674	-1.43152	0.469802	-3.04706	0.002311	0.006942
CNAG_04792	2434.457	0.660484	0.217857	3.031735	0.002432	0.007255
CNAG_04791	1272.794	-0.61799	0.112937	-5.47201	4.45E-08	4.12E-07
CNAG_04789	1775.409	0.971872	0.183539	5.295176	1.19E-07	1.00E-06
CNAG_04788	1910.073	-0.76084	0.237129	-3.20854	0.001334	0.004278
CNAG_04787	401.0619	0.776941	0.166546	4.665034	3.09E-06	1.95E-05
CNAG_12903	74.12723	0.829651	0.27263	3.043139	0.002341	0.007023
CNAG_04785	626.5591	-0.66495	0.234808	-2.83188	0.004628	0.012658
CNAG_04783	1505.076	-0.4951	0.124288	-3.9835	6.79E-05	0.000309
CNAG_04777	931.6976	-0.45553	0.136114	-3.34666	0.000818	0.002777
CNAG_04773	63.59954	0.564584	0.280363	2.013762	0.044035	0.087235
CNAG_12906	66.11613	1.450088	0.305901	4.740381	2.13E-06	1.39E-05
CNAG_04770	2267.278	-0.92586	0.182713	-5.0673	4.03E-07	3.02E-06
CNAG_04769	1193.374	-0.30764	0.130174	-2.36329	0.018114	0.040994
CNAG_04768	1220.495	-0.68257	0.165159	-4.13278	3.58E-05	0.000174
CNAG_04763	2848.652	0.621002	0.306919	2.023343	0.043038	0.085602

CNAG_04762	39867.25	-0.98879	0.239338	-4.13138	3.61E-05	0.000175
CNAG_04761	415.5378	-0.40257	0.173637	-2.31846	0.020424	0.045371
CNAG_04760	33099.45	-0.64947	0.211092	-3.07674	0.002093	0.006364
CNAG_12910	559.6893	-1.40452	0.259163	-5.41946	5.98E-08	5.40E-07
CNAG_04758	19874.37	-0.90575	0.209918	-4.31478	1.60E-05	8.49E-05
CNAG_04753	555.9771	-0.62766	0.141731	-4.42852	9.49E-06	5.33E-05
CNAG_04751	1375.934	1.230806	0.224182	5.490208	4.01E-08	3.77E-07
CNAG_07853	51.3055	2.116019	0.356187	5.940747	2.84E-09	3.32E-08
CNAG_12916	67.50749	0.795703	0.29569	2.691009	0.007124	0.01839
CNAG_04750	333.1582	1.52685	0.261346	5.842264	5.15E-09	5.67E-08
CNAG_04747	969.2673	1.200467	0.157043	7.644205	2.10E-14	5.33E-13
CNAG_04746	2062.11	0.377328	0.171367	2.201874	0.027674	0.058687
CNAG_04744	3302.21	2.69929	0.245163	11.01021	3.41E-28	2.77E-26
CNAG_04741	2522.639	0.503863	0.20122	2.504037	0.012279	0.029352
CNAG_04737	11278.11	1.218588	0.291679	4.177833	2.94E-05	0.000146
CNAG_04736	699.6732	-0.93403	0.2306	-4.05045	5.11E-05	0.000239
CNAG_04732	1215.114	-0.36083	0.160764	-2.24448	0.024802	0.053466
CNAG_04730	1679.757	0.614014	0.1215	5.0536	4.34E-07	3.23E-06
CNAG_12918	30.43895	-1.13547	0.450568	-2.52008	0.011733	0.028188
CNAG_04729	2268.456	-0.86347	0.146279	-5.90287	3.57E-09	4.08E-08
CNAG_04728	1335.764	-0.5046	0.211747	-2.38304	0.01717	0.039093
CNAG_04727	299.0197	1.015026	0.202296	5.017539	5.23E-07	3.85E-06
CNAG_04726	29706.31	-1.00864	0.174013	-5.79635	6.78E-09	7.30E-08
CNAG_04725	1158.212	-0.3985	0.173361	-2.29864	0.021525	0.047446
CNAG_04724	3052.692	1.008351	0.209893	4.804106	1.55E-06	1.04E-05
CNAG_04721	1108.847	-0.36101	0.142457	-2.53416	0.011272	0.02724
CNAG_12921	134.4506	0.57018	0.225651	2.526825	0.01151	0.027723
CNAG_04717	28.25016	0.821932	0.357986	2.295988	0.021677	0.047739
CNAG_04716	4205.379	-0.40802	0.130183	-3.13421	0.001723	0.005344
CNAG_04714	798.1972	0.671298	0.130056	5.161622	2.45E-07	1.92E-06
CNAG_04712	984.5449	-0.3457	0.145293	-2.3793	0.017346	0.039425
CNAG_04711	33.13132	1.084142	0.394238	2.749971	0.00596	0.015828
CNAG_04710	2215.097	-0.25386	0.113897	-2.22886	0.025823	0.055307
CNAG_04709	3099.776	-0.66071	0.113237	-5.83474	5.39E-09	5.90E-08
CNAG_04707	284.4198	0.621541	0.189352	3.282464	0.001029	0.003402
CNAG_04705	1844.286	0.539623	0.158779	3.398582	0.000677	0.00236
CNAG_04704	43.32203	-1.04239	0.350032	-2.97798	0.002902	0.008442
CNAG_04700	237.357	-0.45493	0.224553	-2.02595	0.04277	0.085112
CNAG_04697	1083.274	-1.05134	0.210974	-4.9833	6.25E-07	4.53E-06
CNAG_04695	50.63395	1.49797	0.349577	4.285097	1.83E-05	9.57E-05
CNAG_04694	4021.925	-0.65949	0.129633	-5.08736	3.63E-07	2.75E-06
CNAG_04692	1272.793	-0.86641	0.147213	-5.88542	3.97E-09	4.48E-08
CNAG_04691	68.97791	1.177253	0.428474	2.747545	0.006004	0.015914
CNAG_04690	794.3843	-0.4378	0.133633	-3.27613	0.001052	0.003463
CNAG_04689	827.1002	-0.34123	0.170227	-2.00454	0.045012	0.08884
CNAG_04688	649.9956	0.401621	0.184803	2.173236	0.029763	0.062416
CNAG_04687	16840.14	0.921633	0.139846	6.590349	4.39E-11	6.77E-10

CNAG_04686	525.5554	-0.82215	0.281833	-2.91716	0.003532	0.009999
CNAG_07856	840.4385	0.953624	0.175832	5.423511	5.84E-08	5.29E-07
CNAG_04683	1334.815	-0.95405	0.223519	-4.26831	1.97E-05	0.000102
CNAG_04681	229.7749	1.517405	0.473514	3.204562	0.001353	0.004325
CNAG_04680	1164.302	1.014468	0.208358	4.868876	1.12E-06	7.66E-06
CNAG_04679	1880.099	-0.70236	0.26083	-2.69277	0.007086	0.018317
CNAG_04675	1069.071	1.133092	0.304317	3.723395	0.000197	0.000796
CNAG_12929	267.8376	2.387415	0.214239	11.1437	7.69E-29	6.57E-27
CNAG_04673	162.3214	0.966864	0.185248	5.219302	1.80E-07	1.46E-06
CNAG_12930	32.34344	1.164544	0.435612	2.673354	0.00751	0.019175
CNAG_04671	1418.333	-0.52698	0.142425	-3.70008	0.000216	0.000863
CNAG_04669	947.5293	-0.33685	0.14102	-2.3887	0.016908	0.038562
CNAG_04668	953.6871	0.427581	0.180961	2.362832	0.018136	0.041021
CNAG_04666	7206.183	0.751054	0.136141	5.516759	3.45E-08	3.32E-07
CNAG_04664	890.6925	-0.44749	0.150675	-2.96988	0.002979	0.00864
CNAG_04663	526.1937	1.043223	0.178068	5.858582	4.67E-09	5.17E-08
CNAG_08000	64.14481	1.608939	0.278586	5.775375	7.68E-09	8.21E-08
CNAG_12933	30.20256	-0.93286	0.395291	-2.35994	0.018278	0.041295
CNAG_04658	565.7853	0.826265	0.27237	3.033614	0.002416	0.007213
CNAG_04657	2043.343	2.007048	0.240739	8.337025	7.62E-17	2.57E-15
CNAG_04656	1379.29	0.335514	0.13339	2.515289	0.011893	0.028534
CNAG_04655	1163.806	-1.2694	0.1867	-6.79914	1.05E-11	1.84E-10
CNAG_04653	281.7125	-0.68758	0.195833	-3.51103	0.000446	0.001634
CNAG_04652	2520.48	0.661963	0.232454	2.847715	0.004403	0.012137
CNAG_04651	4251.675	0.981136	0.159646	6.145692	7.96E-10	1.03E-08
CNAG_04649	512.3606	0.577671	0.143424	4.027729	5.63E-05	0.000261
CNAG_04646	562.9894	-0.72818	0.231673	-3.14314	0.001671	0.005212
CNAG_07857	3978.559	-0.52594	0.124554	-4.22258	2.42E-05	0.000123
CNAG_04638	297.7611	1.555568	0.229348	6.782574	1.18E-11	2.04E-10
CNAG_04636	1950.657	0.387564	0.143545	2.699956	0.006935	0.017973
CNAG_04635	4102.406	1.672148	0.21861	7.648995	2.03E-14	5.18E-13
CNAG_04634	1160.639	0.960776	0.156004	6.158663	7.34E-10	9.49E-09
CNAG_12937	26.7104	1.130024	0.47875	2.360366	0.018257	0.04126
CNAG_04632	3988.155	-0.68153	0.336679	-2.02427	0.042942	0.085432
CNAG_04630	2940.085	1.348506	0.258484	5.216972	1.82E-07	1.48E-06
CNAG_04628	3006.52	-0.45575	0.213841	-2.13127	0.033067	0.068301
CNAG_04626	181.4537	1.141911	0.239733	4.763266	1.90E-06	1.25E-05
CNAG_04625	5731.083	0.915592	0.158756	5.767293	8.06E-09	8.56E-08
CNAG_04622	411.3502	1.429033	0.250946	5.694575	1.24E-08	1.27E-07
CNAG_04618	763.8307	-1.13675	0.208573	-5.45015	5.03E-08	4.61E-07
CNAG_04617	221.3967	-0.57542	0.196834	-2.92337	0.003463	0.009827
CNAG_04615	266.5174	-0.78846	0.199206	-3.95799	7.56E-05	0.00034
CNAG_04614	251.7462	0.570986	0.168065	3.397406	0.00068	0.002369
CNAG_04613	3011.917	-0.38484	0.195049	-1.97306	0.048489	0.094737
CNAG_04612	2212.719	-0.99444	0.216805	-4.58677	4.50E-06	2.74E-05
CNAG_04611	2109.229	0.631719	0.235831	2.678688	0.007391	0.018945
CNAG_07858	19.91401	1.040118	0.460582	2.258268	0.023929	0.051965

CNAG_04610	21.50288	2.005314	0.457803	4.380301	1.19E-05	6.53E-05
CNAG_04606	517.4225	1.859704	0.195268	9.523859	1.67E-21	8.90E-20
CNAG_04605	5101.638	-0.80411	0.185698	-4.3302	1.49E-05	7.96E-05
CNAG_04604	3990.749	-0.7704	0.17115	-4.50131	6.75E-06	3.94E-05
CNAG_04603	434.0193	-0.4545	0.1854	-2.45142	0.014229	0.033333
CNAG_04601	19395.27	-0.74379	0.121731	-6.11008	9.96E-10	1.26E-08
CNAG_04600	1059.925	0.604237	0.234664	2.5749	0.010027	0.024667
CNAG_04598	275.0662	0.934499	0.160112	5.836539	5.33E-09	5.85E-08
CNAG_04589	939.2177	1.940622	0.163341	11.88078	1.49E-32	1.69E-30
CNAG_04588	1532.838	-0.5618	0.215047	-2.61244	0.00899	0.022393
CNAG_04587	5083.775	1.577124	0.222926	7.074668	1.50E-12	3.04E-11
CNAG_04585	243.9033	1.696977	0.238001	7.130119	1.00E-12	2.08E-11
CNAG_04584	5187.429	-0.74985	0.211972	-3.53751	0.000404	0.001498
CNAG_04582	254.7639	0.70133	0.171159	4.097538	4.18E-05	0.0002
CNAG_04576	310.6479	1.035681	0.246642	4.199118	2.68E-05	0.000135
CNAG_12946	69.79461	1.117678	0.305898	3.653759	0.000258	0.001014
CNAG_07862	650.7369	-0.84039	0.14843	-5.66184	1.50E-08	1.52E-07
CNAG_07863	12337.62	0.733073	0.201774	3.633138	0.00028	0.001091
CNAG_04571	890.6342	-1.1452	0.152504	-7.50932	5.94E-14	1.43E-12
CNAG_07865	2833.672	0.636232	0.144411	4.405707	1.05E-05	5.85E-05
CNAG_04567	564.1076	-0.35454	0.153952	-2.30291	0.021284	0.046992
CNAG_04566	9218.959	0.402842	0.13422	3.00136	0.002688	0.007922
CNAG_07866	426.2796	-0.5883	0.184069	-3.19606	0.001393	0.004435
CNAG_04564	434.112	0.499439	0.17607	2.836593	0.00456	0.012507
CNAG_04561	512.4147	0.471382	0.218591	2.156453	0.031048	0.064719
CNAG_04553	61.51869	2.01119	0.306743	6.556596	5.50E-11	8.41E-10
CNAG_04552	13.66016	1.36528	0.568663	2.400861	0.016357	0.03752
CNAG_07868	291.3529	-0.71336	0.208914	-3.41461	0.000639	0.002245
CNAG_08001	74.55993	-0.77265	0.26237	-2.94487	0.003231	0.009238
CNAG_01464	4946.695	-1.20855	0.204593	-5.9071	3.48E-09	4.01E-08
CNAG_12950	10.50614	-3.60016	0.825779	-4.35972	1.30E-05	7.11E-05
CNAG_01465	347.5617	0.875752	0.364976	2.399476	0.016419	0.037608
CNAG_01466	773.9926	0.958524	0.186467	5.140438	2.74E-07	2.12E-06
CNAG_01467	521.6224	-0.28667	0.133993	-2.13943	0.032401	0.067186
CNAG_01468	142.7591	-0.76044	0.370801	-2.05082	0.040285	0.080895
CNAG_12951	87.52462	1.046848	0.260338	4.021105	5.79E-05	0.000268
CNAG_01470	3853.7	-1.56367	0.203234	-7.69391	1.43E-14	3.68E-13
CNAG_01471	1215.944	-0.87407	0.163948	-5.33141	9.75E-08	8.41E-07
CNAG_01473	181.159	1.100167	0.212276	5.182708	2.19E-07	1.73E-06
CNAG_12954	116.67	-0.93883	0.239793	-3.91517	9.03E-05	0.0004
CNAG_01475	10269.54	-0.3445	0.128421	-2.68256	0.007306	0.01877
CNAG_01476	2691.637	-0.46515	0.123919	-3.75362	0.000174	0.000718
CNAG_01480	32206.08	-0.84953	0.239943	-3.54055	0.000399	0.001483
CNAG_01481	270.8942	1.1674	0.203292	5.742472	9.33E-09	9.83E-08
CNAG_01486	45072.38	-0.95148	0.254868	-3.73324	0.000189	0.000771
CNAG_01487	26.08257	1.058653	0.465525	2.274108	0.022959	0.050258
CNAG_01489	150.505	-1.24095	0.296519	-4.18505	2.85E-05	0.000142

CNAG_01490	472.6067	0.410589	0.170412	2.409385	0.015979	0.036783
CNAG_01493	1058.859	1.237131	0.232621	5.318224	1.05E-07	8.97E-07
CNAG_12957	16.83516	-1.04886	0.48145	-2.17854	0.029366	0.061714
CNAG_01495	1087.304	1.126939	0.181765	6.199963	5.65E-10	7.39E-09
CNAG_01498	461.0215	-0.74071	0.195355	-3.79161	0.00015	0.000623
CNAG_08003	538.8346	-0.58502	0.212783	-2.74936	0.005971	0.015842
CNAG_01506	98.72248	1.333009	0.22586	5.901928	3.59E-09	4.10E-08
CNAG_01507	567.5718	-0.33325	0.164486	-2.02598	0.042767	0.085112
CNAG_01508	2111.373	-0.62915	0.139825	-4.49956	6.81E-06	3.97E-05
CNAG_01509	544.6401	-0.68329	0.214393	-3.18709	0.001437	0.004551
CNAG_01511	38.13081	1.913081	0.371672	5.147225	2.64E-07	2.05E-06
CNAG_01512	471.8925	1.524159	0.200353	7.607383	2.80E-14	7.01E-13
CNAG_07589	89.20854	-0.72951	0.27099	-2.69202	0.007102	0.01834
CNAG_07591	2266.289	1.119483	0.160316	6.982993	2.89E-12	5.51E-11
CNAG_01518	1526.5	-0.46834	0.171732	-2.72717	0.006388	0.016791
CNAG_01519	595.5433	0.868876	0.156206	5.562359	2.66E-08	2.62E-07
CNAG_01520	1300.224	-0.30392	0.149156	-2.03758	0.041592	0.0831
CNAG_01522	1461.073	-0.48513	0.149184	-3.25191	0.001146	0.003738
CNAG_01523	5297.082	-0.32988	0.167454	-1.96998	0.048841	0.095272
CNAG_01524	2645.229	0.860279	0.167632	5.131937	2.87E-07	2.21E-06
CNAG_01525	311.831	0.430054	0.177058	2.42889	0.015145	0.035178
CNAG_12967	44.56283	1.264563	0.317385	3.984321	6.77E-05	0.000308
CNAG_12968	77.04696	1.972437	0.273675	7.207218	5.71E-13	1.22E-11
CNAG_01528	975.7016	-0.7543	0.126667	-5.95502	2.60E-09	3.07E-08
CNAG_01529	1099.234	-0.46385	0.170681	-2.71766	0.006575	0.017173
CNAG_01531	928.6669	-0.66397	0.181286	-3.66257	0.00025	0.000983
CNAG_01534	174.4485	1.147795	0.256279	4.478697	7.51E-06	4.32E-05
CNAG_01538	200.5905	0.971876	0.28297	3.43455	0.000594	0.002106
CNAG_01539	25635.48	-0.92866	0.168328	-5.51698	3.45E-08	3.32E-07
CNAG_01542	912.0421	-0.91175	0.199168	-4.57779	4.70E-06	2.84E-05
CNAG_01544	5738.517	-0.95376	0.216561	-4.40413	1.06E-05	5.89E-05
CNAG_01545	588.1984	-0.43593	0.171546	-2.54118	0.011048	0.026789
CNAG_01549	2882.854	-0.54599	0.121271	-4.50221	6.72E-06	3.93E-05
CNAG_01550	1271.396	-0.63782	0.163538	-3.90016	9.61E-05	0.000421
CNAG_01551	1225.814	-0.96371	0.205194	-4.69659	2.65E-06	1.70E-05
CNAG_01552	1371.376	-0.62327	0.269007	-2.31693	0.020507	0.045504
CNAG_01555	86.27468	0.570557	0.268548	2.124603	0.03362	0.06919
CNAG_01556	1290.581	0.654427	0.166408	3.932676	8.40E-05	0.000375
CNAG_01558	26931.68	1.419912	0.149809	9.478168	2.59E-21	1.34E-19
CNAG_01559	1583.15	-0.74316	0.198296	-3.74774	0.000178	0.000733
CNAG_01560	99.67169	1.054133	0.265849	3.96516	7.33E-05	0.000332
CNAG_01561	2667.63	0.599335	0.248231	2.414427	0.01576	0.036372
CNAG_01562	26070.5	0.812391	0.137327	5.915742	3.30E-09	3.82E-08
CNAG_01564	2884.236	-0.8468	0.159033	-5.32467	1.01E-07	8.69E-07
CNAG_01566	796.5241	-0.97133	0.253471	-3.83213	0.000127	0.000539
CNAG_01569	964.2754	-0.39234	0.172451	-2.27506	0.022902	0.05016
CNAG_01577	61396.07	-0.53854	0.161529	-3.33405	0.000856	0.002881

CNAG_01580	2661.964	0.250747	0.120811	2.075524	0.037938	0.076965
CNAG_01583	2865.318	-0.37133	0.184952	-2.0077	0.044676	0.088241
CNAG_01584	1381.585	1.267793	0.214592	5.907914	3.46E-09	4.00E-08
CNAG_01585	1384.189	2.578735	0.231998	11.11533	1.06E-28	8.84E-27
CNAG_01586	16102.7	-0.69916	0.235868	-2.96418	0.003035	0.008763
CNAG_01587	2217.384	-0.54869	0.163978	-3.34612	0.00082	0.002782
CNAG_12974	58.37785	2.43877	0.442467	5.511757	3.55E-08	3.41E-07
CNAG_01588	6183.824	0.884502	0.197517	4.478104	7.53E-06	4.32E-05
CNAG_01590	801.3	-0.67565	0.165491	-4.0827	4.45E-05	0.000211
CNAG_01592	192.9267	-0.68533	0.20486	-3.34536	0.000822	0.002786
CNAG_01593	2798.235	-0.86504	0.24113	-3.58747	0.000334	0.001272
CNAG_01594	7670.545	-0.58256	0.163129	-3.57114	0.000355	0.001341
CNAG_01595	654.2025	-0.9789	0.21123	-4.63426	3.58E-06	2.23E-05
CNAG_01596	1207.046	-0.9603	0.234646	-4.09255	4.27E-05	0.000204
CNAG_01598	4279.535	-1.11925	0.280855	-3.98515	6.74E-05	0.000307
CNAG_01599	854.0656	-1.12463	0.383527	-2.93234	0.003364	0.009585
CNAG_01600	1531.399	-0.77509	0.239788	-3.23238	0.001228	0.00398
CNAG_01601	327.0946	0.531089	0.17143	3.097997	0.001948	0.005959
CNAG_01603	6919.337	-0.50726	0.221243	-2.29275	0.021862	0.048061
CNAG_01605	638.5882	1.14895	0.189821	6.052815	1.42E-09	1.76E-08
CNAG_01607	1355.565	-0.61921	0.206378	-3.00039	0.002696	0.007944
CNAG_01608	1355.248	-0.65975	0.116379	-5.66897	1.44E-08	1.47E-07
CNAG_01609	2620.534	-0.99123	0.126163	-7.85679	3.94E-15	1.09E-13
CNAG_01610	1717.436	0.804667	0.31236	2.576093	0.009992	0.024612
CNAG_01611	732.3153	1.017557	0.180238	5.645635	1.65E-08	1.66E-07
CNAG_01613	2233.516	0.690656	0.255065	2.707767	0.006774	0.017618
CNAG_01614	1626.214	-0.2596	0.116819	-2.22226	0.026265	0.056119
CNAG_01615	1104.75	0.541965	0.195544	2.771579	0.005579	0.014965
CNAG_07594	894.3915	0.359392	0.161848	2.220551	0.026381	0.056351
CNAG_01619	868.4353	0.454585	0.139268	3.2641	0.001098	0.003594
CNAG_01620	839.5024	-0.99857	0.19591	-5.09708	3.45E-07	2.62E-06
CNAG_12978	162.4187	1.208983	0.354505	3.410338	0.000649	0.002275
CNAG_01621	7336.807	1.780353	0.246453	7.223892	5.05E-13	1.09E-11
CNAG_01625	563.4569	-0.78731	0.162133	-4.85592	1.20E-06	8.13E-06
CNAG_01626	2693.83	-0.51489	0.125557	-4.10083	4.12E-05	0.000197
CNAG_01628	21678.15	-0.95414	0.232552	-4.10292	4.08E-05	0.000196
CNAG_01629	675.7061	-1.20717	0.259365	-4.65434	3.25E-06	2.04E-05
CNAG_01631	1527.536	-0.34975	0.116966	-2.99018	0.002788	0.008172
CNAG_01632	851.6002	0.439262	0.142711	3.077991	0.002084	0.006347
CNAG_01634	3288.995	-0.35294	0.110705	-3.18807	0.001432	0.00454
CNAG_01638	1069.536	-0.35915	0.149438	-2.40332	0.016247	0.037301
CNAG_01640	3996.508	0.914862	0.263856	3.467273	0.000526	0.001894
CNAG_01644	1818.637	-1.045	0.157017	-6.65531	2.83E-11	4.54E-10
CNAG_01645	97.83634	1.273249	0.236687	5.379464	7.47E-08	6.57E-07
CNAG_01648	19735.94	-1.31832	0.230806	-5.71182	1.12E-08	1.16E-07
CNAG_01650	1203.659	-0.63234	0.1455	-4.34595	1.39E-05	7.52E-05
CNAG_01653	3161.175	1.635958	0.359031	4.556592	5.20E-06	3.12E-05

CNAG_01655	5403.541	0.697862	0.245452	2.843176	0.004467	0.012285
CNAG_01660	1168.888	-0.27276	0.130284	-2.09359	0.036296	0.074119
CNAG_12982	6.124772	2.394698	0.964276	2.483416	0.013013	0.03082
CNAG_01665	532.6417	-0.71028	0.187239	-3.79343	0.000149	0.00062
CNAG_01666	1655.507	-0.82463	0.283371	-2.91007	0.003613	0.010196
CNAG_01667	938.2104	-0.50227	0.168292	-2.98453	0.00284	0.008303
CNAG_01668	18.47569	1.166468	0.513949	2.269617	0.023231	0.050754
CNAG_01669	1001.512	-0.32943	0.152198	-2.16447	0.030428	0.063644
CNAG_01671	150.1181	0.620281	0.183268	3.384556	0.000713	0.002462
CNAG_01674	66.85076	-0.96065	0.257908	-3.72479	0.000195	0.000792
CNAG_01679	3228.969	-0.44077	0.183568	-2.40112	0.016345	0.037504
CNAG_01680	80.32814	0.710074	0.24124	2.943431	0.003246	0.009274
CNAG_12985	1380.675	-0.67367	0.231355	-2.91185	0.003593	0.010146
CNAG_01681	9303.489	-0.96155	0.15586	-6.16934	6.86E-10	8.90E-09
CNAG_07596	191.8114	2.114417	0.231033	9.152	5.59E-20	2.54E-18
CNAG_01683	5584.181	0.317564	0.135172	2.349334	0.018807	0.042227
CNAG_01686	4591.883	1.014989	0.184969	5.487359	4.08E-08	3.82E-07
CNAG_01687	1638.789	-0.49074	0.197703	-2.48223	0.013056	0.030904
CNAG_01688	5215.015	-0.36223	0.177121	-2.04508	0.040847	0.08188
CNAG_12991	97.32666	0.62253	0.228003	2.730359	0.006327	0.016651
CNAG_01690	856.2562	0.876936	0.153859	5.699598	1.20E-08	1.24E-07
CNAG_01691	1682.381	1.33037	0.18753	7.094167	1.30E-12	2.66E-11
CNAG_12992	90.84264	1.999035	0.286476	6.978012	2.99E-12	5.66E-11
CNAG_01692	2027.528	0.288371	0.129216	2.231696	0.025635	0.055002
CNAG_01693	1069.247	-0.61127	0.150149	-4.07107	4.68E-05	0.000221
CNAG_12993	253.8494	4.10606	1.406564	2.919213	0.003509	0.009937
CNAG_01695	163.9948	1.785078	0.197031	9.059902	1.31E-19	5.76E-18
CNAG_01699	728.6099	0.463486	0.189766	2.442406	0.01459	0.034047
CNAG_01702	440.5116	0.391982	0.158334	2.47567	0.013299	0.031347
CNAG_01703	280.1995	0.565661	0.159483	3.546831	0.00039	0.001454
CNAG_01704	5098.194	0.499408	0.176215	2.834087	0.004596	0.012584
CNAG_01713	1812.328	0.792241	0.24823	3.191567	0.001415	0.004499
CNAG_01714	26.83944	1.017415	0.382663	2.658772	0.007843	0.019922
CNAG_01715	3411.854	-0.60995	0.205993	-2.96102	0.003066	0.00883
CNAG_01716	1327.673	-0.76756	0.127338	-6.02771	1.66E-09	2.04E-08
CNAG_01719	130.4212	0.70054	0.221458	3.163315	0.00156	0.004901
CNAG_01721	1859.677	-1.6264	0.189259	-8.5935	8.44E-18	3.09E-16
CNAG_01722	7190.533	0.727328	0.275129	2.643584	0.008203	0.020713
CNAG_01723	1405.706	0.42312	0.152707	2.770797	0.005592	0.014991
CNAG_12996	10.51626	1.397013	0.620283	2.25222	0.024308	0.052631
CNAG_01727	78863.58	0.660382	0.14117	4.677928	2.90E-06	1.85E-05
CNAG_12998	21.77811	-1.44942	0.584931	-2.47793	0.013215	0.031214
CNAG_01732	2673.698	0.588404	0.149989	3.922968	8.75E-05	0.000388
CNAG_01733	6998.083	-0.51785	0.223025	-2.32192	0.020237	0.045006
CNAG_01735	3066.854	2.13781	0.189363	11.2895	1.48E-29	1.35E-27
CNAG_01737	3761.762	-0.86482	0.138566	-6.24125	4.34E-10	5.82E-09
CNAG_01739	1025.3	0.91073	0.200929	4.532602	5.83E-06	3.44E-05

CNAG_01742	333.4892	1.485815	0.174803	8.499939	1.90E-17	6.67E-16
CNAG_01743	832.315	1.753221	0.18996	9.22942	2.72E-20	1.29E-18
CNAG_01744	9733.519	0.390838	0.128035	3.052596	0.002269	0.006831
CNAG_01745	10666.05	0.646276	0.118892	5.435841	5.45E-08	4.96E-07
CNAG_01746	494.7675	1.100219	0.166221	6.619034	3.62E-11	5.67E-10
CNAG_07600	727.086	1.067186	0.247765	4.307259	1.65E-05	8.75E-05
CNAG_01750	14499.68	2.373868	0.173414	13.68904	1.18E-42	2.29E-40
CNAG_01751	2789.388	2.211216	0.255517	8.653888	4.98E-18	1.85E-16
CNAG_01752	6609.959	-0.80258	0.173	-4.63918	3.50E-06	2.18E-05
CNAG_01753	6922.828	0.972594	0.15144	6.422292	1.34E-10	1.96E-09
CNAG_01755	2840.915	-0.61002	0.12456	-4.89736	9.71E-07	6.74E-06
CNAG_01758	107.285	0.480047	0.212091	2.263398	0.023611	0.051444
CNAG_13000	12.45636	1.733108	0.598628	2.895133	0.00379	0.010608
CNAG_08008	685.3608	0.792925	0.275413	2.879038	0.003989	0.011086
CNAG_01764	1179.533	0.424805	0.177007	2.399932	0.016398	0.037583
CNAG_01765	1275.854	0.371633	0.178779	2.078725	0.037643	0.076459
CNAG_07604	357.6251	0.647836	0.168487	3.845023	0.000121	0.000515
CNAG_01771	1620.398	0.505034	0.224408	2.250515	0.024416	0.052835
CNAG_01772	2525.562	-0.92568	0.124076	-7.46059	8.61E-14	2.00E-12
CNAG_01773	719.9459	0.283204	0.133754	2.117357	0.03423	0.07039
CNAG_13002	11.52858	1.767611	0.612514	2.885832	0.003904	0.010876
CNAG_01776	217.7817	0.495744	0.18783	2.639331	0.008307	0.020941
CNAG_01779	660.5379	0.328817	0.1397	2.353743	0.018585	0.041819
CNAG_13004	31.03489	0.77596	0.384096	2.020225	0.04336	0.086135
CNAG_01789	2806.358	-0.67168	0.204039	-3.2919	0.000995	0.003304
CNAG_01791	2103.062	-0.41484	0.131833	-3.14671	0.001651	0.005161
CNAG_01796	408.2626	0.843763	0.149223	5.654364	1.56E-08	1.58E-07
CNAG_01797	1659.395	-0.81055	0.186606	-4.34365	1.40E-05	7.59E-05
CNAG_01799	7086.034	-0.74724	0.231329	-3.2302	0.001237	0.004005
CNAG_01800	1780.139	-1.63441	0.316308	-5.16715	2.38E-07	1.87E-06
CNAG_01803	559.9347	0.406334	0.169567	2.3963	0.016562	0.037891
CNAG_13007	173.4295	0.811536	0.26435	3.069928	0.002141	0.006499
CNAG_01806	27.58516	1.175958	0.420068	2.799451	0.005119	0.01386
CNAG_01808	2015.949	-0.54532	0.123454	-4.41716	1.00E-05	5.59E-05
CNAG_01809	1359.588	-0.57154	0.170918	-3.34393	0.000826	0.002794
CNAG_01810	549.7051	-0.82989	0.194061	-4.27643	1.90E-05	9.92E-05
CNAG_01812	3096.691	-0.83624	0.192904	-4.33503	1.46E-05	7.85E-05
CNAG_01813	15920.01	-0.59307	0.205895	-2.88044	0.003971	0.011042
CNAG_01816	148.0092	-0.39232	0.193055	-2.03218	0.042135	0.083933
CNAG_01818	1599.59	-0.45267	0.131432	-3.44414	0.000573	0.002043
CNAG_01820	24220.26	-0.67195	0.138703	-4.84452	1.27E-06	8.58E-06
CNAG_01821	1403.276	1.70679	0.20802	8.204938	2.31E-16	7.39E-15
CNAG_01822	1487.571	-0.30794	0.130223	-2.36471	0.018044	0.040848
CNAG_01823	3675.666	0.457989	0.178564	2.564854	0.010322	0.025314
CNAG_13008	154.172	-0.68029	0.287455	-2.36661	0.017952	0.040651
CNAG_01824	4581.56	0.563584	0.210667	2.675232	0.007468	0.019115
CNAG_01828	1009.336	-0.59187	0.153086	-3.86627	0.000111	0.000475

CNAG_07607	1308.38	0.454883	0.133669	3.403053	0.000666	0.002326
CNAG_07609	1602.109	-0.77116	0.13878	-5.5567	2.75E-08	2.70E-07
CNAG_01832	711.4152	0.338037	0.142019	2.380232	0.017302	0.039347
CNAG_01836	41.2134	-1.22568	0.348172	-3.52031	0.000431	0.001584
CNAG_13010	267.1419	1.448356	0.228177	6.34751	2.19E-10	3.09E-09
CNAG_01840	23260.23	-0.53023	0.112723	-4.70383	2.55E-06	1.65E-05
CNAG_01841	75.28866	0.873464	0.298016	2.930926	0.00338	0.009625
CNAG_01842	1559.691	-0.87187	0.226611	-3.84742	0.000119	0.00051
CNAG_01844	523.4775	0.386498	0.157913	2.447547	0.014383	0.033644
CNAG_01847	284.4879	2.006729	0.25015	8.022097	1.04E-15	3.14E-14
CNAG_01848	3872.629	0.809486	0.278935	2.902059	0.003707	0.010427
CNAG_01854	13944.62	0.519616	0.141136	3.681654	0.000232	0.000921
CNAG_01856	334.7915	1.648435	0.260198	6.335304	2.37E-10	3.34E-09
CNAG_01858	64.53579	0.585369	0.260855	2.244043	0.02483	0.053511
CNAG_01861	2184.606	0.978123	0.231406	4.226865	2.37E-05	0.000121
CNAG_07610	12.14255	-1.29375	0.592469	-2.18365	0.028988	0.061049
CNAG_01863	4289.887	-0.55871	0.20454	-2.73156	0.006304	0.016613
CNAG_13013	134.3118	-1.34215	0.313132	-4.28619	1.82E-05	9.53E-05
CNAG_01864	1262	-0.78374	0.149033	-5.25886	1.45E-07	1.20E-06
CNAG_01865	1086.812	-1.36307	0.152548	-8.93536	4.06E-19	1.72E-17
CNAG_01866	934.1104	1.053207	0.269009	3.915142	9.04E-05	0.0004
CNAG_01867	2676.19	0.352997	0.157227	2.245148	0.024759	0.053402
CNAG_01871	1191.987	-0.48104	0.172991	-2.78071	0.005424	0.014565
CNAG_01873	6.458666	3.007629	1.018949	2.951696	0.00316	0.009059
CNAG_01874	2789.952	0.833658	0.219473	3.798453	0.000146	0.000611
CNAG_01876	1465.299	0.571032	0.177757	3.212427	0.001316	0.004234
CNAG_01877	8343.073	-0.75724	0.19069	-3.97106	7.16E-05	0.000324
CNAG_01878	1464.657	0.451236	0.131542	3.430345	0.000603	0.002136
CNAG_01880	1202.518	-0.29179	0.144725	-2.01615	0.043784	0.086846
CNAG_01882	583.0035	-0.90794	0.194273	-4.6735	2.96E-06	1.88E-05
CNAG_07611	28.76741	1.488991	0.401575	3.707881	0.000209	0.000841
CNAG_01883	121.0179	2.136228	0.530532	4.026579	5.66E-05	0.000262
CNAG_01884	55254.05	-0.90679	0.158377	-5.72556	1.03E-08	1.08E-07
CNAG_01885	1125.998	-0.77588	0.166886	-4.64919	3.33E-06	2.09E-05
CNAG_13015	97.00212	-1.18064	0.272221	-4.33705	1.44E-05	7.79E-05
CNAG_01889	4133.842	3.595972	0.189135	19.01269	1.34E-80	9.67E-78
CNAG_01890	47371.17	-0.37225	0.179248	-2.07673	0.037827	0.076794
CNAG_01891	749.5437	0.433661	0.142923	3.034226	0.002412	0.007201
CNAG_01892	2442.323	0.740204	0.269977	2.741725	0.006112	0.016145
CNAG_01893	2625.111	0.902206	0.141945	6.356044	2.07E-10	2.93E-09
CNAG_01894	2376.308	-0.43186	0.125939	-3.42917	0.000605	0.002141
CNAG_01896	16099.69	0.935159	0.135355	6.908958	4.88E-12	9.02E-11
CNAG_01897	5467.897	-1.01294	0.128428	-7.8872	3.09E-15	8.68E-14
CNAG_01898	1631.079	-0.71232	0.132011	-5.39596	6.82E-08	6.08E-07
CNAG_01902	2341.736	-0.7022	0.174446	-4.02533	5.69E-05	0.000263
CNAG_01903	406.0368	-1.3776	0.205187	-6.71388	1.90E-11	3.14E-10
CNAG_01904	891.5203	-0.40044	0.200803	-1.99422	0.046128	0.090771

CNAG_01906	576.2489	-0.33033	0.150168	-2.19974	0.027825	0.058976
CNAG_01907	1199.082	-1.08865	0.325523	-3.34432	0.000825	0.002791
CNAG_01908	363.9971	-0.68862	0.192854	-3.57069	0.000356	0.001342
CNAG_13021	489.8642	0.796157	0.164223	4.848034	1.25E-06	8.44E-06
CNAG_01912	6198.959	0.543924	0.182559	2.979439	0.002888	0.008421
CNAG_01913	284.9271	0.7321	0.171389	4.271558	1.94E-05	0.000101
CNAG_01914	1445.626	-0.37798	0.157223	-2.4041	0.016212	0.037242
CNAG_01915	10591.02	0.424675	0.141621	2.998672	0.002712	0.007983
CNAG_13022	29.45576	-0.76089	0.368145	-2.06681	0.038752	0.078173
CNAG_01917	293.7818	-1.03109	0.182902	-5.63743	1.73E-08	1.73E-07
CNAG_01919	1271.192	1.138027	0.175447	6.486449	8.79E-11	1.32E-09
CNAG_01920	22290.49	0.496355	0.130828	3.793951	0.000148	0.00062
CNAG_01922	987.4754	0.574619	0.249662	2.301581	0.021359	0.047144
CNAG_01924	1425.983	0.596423	0.193516	3.082041	0.002056	0.006266
CNAG_01925	486.6026	0.977735	0.246882	3.960335	7.48E-05	0.000338
CNAG_01934	68.42027	0.821358	0.25059	3.277701	0.001047	0.003449
CNAG_08011	220.0807	0.684038	0.194131	3.523589	0.000426	0.001569
CNAG_01942	634.3924	1.057753	0.293136	3.608403	0.000308	0.001186
CNAG_01943	2575.731	-0.50625	0.168782	-2.99943	0.002705	0.007966
CNAG_01947	472.3497	-1.36318	0.298049	-4.57368	4.79E-06	2.89E-05
CNAG_01949	1769.866	-1.67437	0.322076	-5.19868	2.01E-07	1.61E-06
CNAG_01951	25965.07	-1.08772	0.243033	-4.47559	7.62E-06	4.37E-05
CNAG_13034	6.381678	1.864393	0.791568	2.355317	0.018507	0.041694
CNAG_01953	544.7344	1.058467	0.149714	7.069937	1.55E-12	3.13E-11
CNAG_13035	69.23935	1.400165	0.372961	3.75419	0.000174	0.000717
CNAG_01954	978.3304	5.19529	0.270402	19.21323	2.87E-82	2.28E-79
CNAG_01957	5500.312	0.615605	0.177901	3.460377	0.000539	0.001939
CNAG_01959	1655.048	-1.13362	0.248887	-4.55476	5.24E-06	3.14E-05
CNAG_01960	180.1647	1.501248	0.231894	6.473853	9.55E-11	1.42E-09
CNAG_01963	368.1061	-0.50313	0.182541	-2.75627	0.005847	0.015553
CNAG_01965	76.10516	-1.33219	0.256245	-5.19889	2.00E-07	1.61E-06
CNAG_01969	215.1733	0.483695	0.224993	2.149819	0.03157	0.065668
CNAG_01970	2508.646	0.879952	0.159282	5.524507	3.30E-08	3.19E-07
CNAG_01972	1949.693	0.80987	0.215859	3.75185	0.000176	0.000723
CNAG_01974	847.4947	-0.83513	0.205031	-4.07319	4.64E-05	0.000219
CNAG_01976	30345.25	-1.14359	0.26988	-4.2374	2.26E-05	0.000115
CNAG_01981	8259.497	-0.4354	0.125865	-3.4593	0.000542	0.001945
CNAG_01982	617.1063	0.624306	0.166214	3.756041	0.000173	0.000712
CNAG_01983	1721.746	-0.86168	0.226778	-3.79968	0.000145	0.000608
CNAG_01985	4977.067	-0.2949	0.12064	-2.44449	0.014506	0.033881
CNAG_01986	1524.111	1.121601	0.190117	5.899529	3.65E-09	4.15E-08
CNAG_13042	44.64373	1.583411	0.400436	3.954213	7.68E-05	0.000345
CNAG_01987	2215.157	0.560214	0.157446	3.558139	0.000373	0.001398
CNAG_01988	3597.959	1.138726	0.277015	4.110696	3.94E-05	0.00019
CNAG_01989	212.3376	1.868547	0.202812	9.21322	3.16E-20	1.50E-18
CNAG_01990	41431.64	-1.01443	0.184233	-5.5062	3.67E-08	3.51E-07
CNAG_01991	8598.857	-0.86493	0.178941	-4.83358	1.34E-06	9.02E-06

CNAG_01992	101.0048	1.580479	0.26688	5.92206	3.18E-09	3.68E-08
CNAG_01993	404.8912	0.659634	0.205607	3.208223	0.001336	0.004279
CNAG_01994	40.91381	1.170926	0.352536	3.321437	0.000896	0.003001
CNAG_01997	3031.046	-1.10182	0.177964	-6.19126	5.97E-10	7.79E-09
CNAG_01999	559.4627	-0.3944	0.1771	-2.22698	0.025948	0.055531
CNAG_02000	2552.424	0.997136	0.194624	5.123395	3.00E-07	2.30E-06
CNAG_02001	2074.059	0.823407	0.18238	4.514797	6.34E-06	3.72E-05
CNAG_02002	2828.588	-0.82664	0.20397	-4.05274	5.06E-05	0.000237
CNAG_08013	874.0688	-0.27752	0.137613	-2.01666	0.043731	0.086784
CNAG_02004	3595.788	0.771645	0.123041	6.27144	3.58E-10	4.84E-09
CNAG_02005	405.6325	-4.33272	0.940399	-4.60732	4.08E-06	2.52E-05
CNAG_02006	1505.006	0.90215	0.184222	4.897071	9.73E-07	6.74E-06
CNAG_02007	7367.185	-0.49326	0.1138	-4.33442	1.46E-05	7.86E-05
CNAG_02008	3226.368	1.334063	0.20883	6.388272	1.68E-10	2.40E-09
CNAG_02009	1497.572	0.601754	0.175367	3.431394	0.0006	0.00213
CNAG_02010	580.1039	-0.48205	0.16477	-2.92558	0.003438	0.009767
CNAG_02011	731.9068	-0.48033	0.181653	-2.64424	0.008188	0.020686
CNAG_07619	1285.899	1.023764	0.17102	5.986218	2.15E-09	2.59E-08
CNAG_13043	110.2366	-0.49403	0.227324	-2.17324	0.029762	0.062416
CNAG_07026	41.50189	0.86733	0.340631	2.546242	0.010889	0.026476
CNAG_05990	461.0397	0.486391	0.163874	2.968077	0.002997	0.008679
CNAG_05993	4174.95	-1.32505	0.417333	-3.17504	0.001498	0.004728
CNAG_05994	4863.652	0.993318	0.26394	3.763418	0.000168	0.000693
CNAG_05995	1704.016	-0.92006	0.131605	-6.99108	2.73E-12	5.25E-11
CNAG_05997	4106.404	-1.01959	0.198097	-5.14696	2.65E-07	2.05E-06
CNAG_05999	1033.788	0.47088	0.171251	2.749656	0.005966	0.015837
CNAG_06001	1113.154	-0.75149	0.198087	-3.79377	0.000148	0.00062
CNAG_06002	548.4	-0.60919	0.173042	-3.52048	0.000431	0.001584
CNAG_06004	507.9144	-0.27691	0.129959	-2.13073	0.033111	0.068356
CNAG_06005	1625.32	-0.70666	0.17113	-4.12938	3.64E-05	0.000177
CNAG_06006	1348.049	-0.38044	0.146885	-2.59005	0.009596	0.023742
CNAG_06007	926.6096	-1.01907	0.221655	-4.59756	4.27E-06	2.63E-05
CNAG_06008	2822.206	-1.20324	0.149062	-8.07205	6.91E-16	2.11E-14
CNAG_06009	974.153	1.278125	0.198814	6.428749	1.29E-10	1.89E-09
CNAG_13046	177.324	3.914676	0.407975	9.595378	8.36E-22	4.61E-20
CNAG_06012	5773.027	-0.59112	0.193537	-3.05431	0.002256	0.0068
CNAG_06018	884.4276	0.406414	0.201172	2.020226	0.04336	0.086135
CNAG_06021	5606.902	1.3166	0.168713	7.803803	6.01E-15	1.63E-13
CNAG_06026	2268.959	-0.45756	0.143418	-3.19043	0.001421	0.004512
CNAG_06027	118.6324	2.962348	0.263495	11.24251	2.52E-29	2.23E-27
CNAG_06028	483.4542	-0.52988	0.147753	-3.58627	0.000335	0.001276
CNAG_06029	389.0015	-0.61168	0.154287	-3.9646	7.35E-05	0.000332
CNAG_06030	590.1055	0.301238	0.131919	2.283507	0.022401	0.049143
CNAG_06033	1032.29	-0.59213	0.211569	-2.79876	0.00513	0.013883
CNAG_06034	1999.188	0.351722	0.174952	2.010396	0.044389	0.087784
CNAG_06035	712.4227	1.040001	0.160609	6.475346	9.46E-11	1.42E-09
CNAG_06036	4069.298	0.548749	0.175749	3.122354	0.001794	0.005539

CNAG_13055	50.33539	1.163234	0.316404	3.676421	0.000237	0.000938
CNAG_06052	1028.865	0.446799	0.164994	2.707972	0.00677	0.017613
CNAG_06055	205.428	0.666557	0.197139	3.381152	0.000722	0.00249
CNAG_06060	3593.177	1.16544	0.256894	4.536651	5.72E-06	3.39E-05
CNAG_06061	4954.102	-0.44078	0.125262	-3.51889	0.000433	0.00159
CNAG_06063	1343.425	-0.64854	0.133776	-4.84798	1.25E-06	8.44E-06
CNAG_06064	1237.816	0.971046	0.252277	3.849123	0.000119	0.000507
CNAG_06065	1831.974	0.568031	0.235866	2.408274	0.016028	0.036873
CNAG_06066	59.35672	1.741251	0.482246	3.61071	0.000305	0.001177
CNAG_07901	2755.316	0.912177	0.273571	3.334332	0.000855	0.00288
CNAG_07902	9801.368	-0.57205	0.147846	-3.86921	0.000109	0.000471
CNAG_06072	1525.955	-0.59269	0.182516	-3.24731	0.001165	0.003792
CNAG_13060	20.03477	1.046552	0.452451	2.313073	0.020719	0.045884
CNAG_13061	232.5635	-0.62171	0.269635	-2.30573	0.021126	0.046694
CNAG_06074	1968.784	1.837625	0.280224	6.557712	5.46E-11	8.37E-10
CNAG_06075	14178.65	0.857111	0.198809	4.31122	1.62E-05	8.61E-05
CNAG_06079	4814.985	-0.3569	0.131064	-2.72313	0.006467	0.016947
CNAG_06080	4216.965	0.38471	0.129425	2.972454	0.002954	0.00858
CNAG_06082	10.37644	-1.97194	0.72471	-2.72101	0.006508	0.017039
CNAG_13063	1211.315	0.441412	0.173596	2.542746	0.010999	0.026693
CNAG_06085	1017.177	0.897253	0.177624	5.051421	4.39E-07	3.27E-06
CNAG_06086	837.6739	-0.54445	0.163601	-3.32792	0.000875	0.00294
CNAG_06087	1206.537	-1.44384	0.212909	-6.78146	1.19E-11	2.05E-10
CNAG_06095	70698.83	-0.90795	0.242139	-3.74969	0.000177	0.000729
CNAG_06096	11341.02	-1.36592	0.135958	-10.0466	9.51E-24	6.19E-22
CNAG_06097	464.0876	-0.48253	0.194104	-2.48592	0.012922	0.030659
CNAG_06100	819.9915	0.689177	0.155203	4.44049	8.98E-06	5.06E-05
CNAG_06101	119193.9	-0.77819	0.153217	-5.07901	3.79E-07	2.86E-06
CNAG_13067	155.2584	0.481375	0.221772	2.170585	0.029963	0.062769
CNAG_06102	172.2447	1.173725	0.20384	5.758066	8.51E-09	9.01E-08
CNAG_06103	4783.081	0.780022	0.198807	3.923513	8.73E-05	0.000388
CNAG_06104	665.1195	-1.89673	0.226816	-8.3624	6.15E-17	2.10E-15
CNAG_13069	20.53869	-2.89087	0.611122	-4.73044	2.24E-06	1.46E-05
CNAG_06105	105.2853	1.708945	0.372514	4.587594	4.48E-06	2.73E-05
CNAG_06106	7914.293	0.495945	0.187548	2.644369	0.008184	0.020685
CNAG_06109	21560.74	1.90181	0.271521	7.00428	2.48E-12	4.83E-11
CNAG_06112	11978.07	-0.53993	0.123501	-4.37188	1.23E-05	6.75E-05
CNAG_06113	61073.26	-0.80816	0.223583	-3.61459	0.000301	0.001162
CNAG_06118	1228.658	-0.89058	0.235472	-3.7821	0.000156	0.000645
CNAG_06119	205.5649	1.782989	0.193081	9.234401	2.60E-20	1.25E-18
CNAG_06121	3424.663	1.691985	0.225965	7.48783	7.00E-14	1.66E-12
CNAG_06122	1175.721	-0.89458	0.182831	-4.89292	9.94E-07	6.86E-06
CNAG_06123	8264.354	-0.75287	0.172788	-4.35717	1.32E-05	7.19E-05
CNAG_06125	303615.7	-0.44973	0.115528	-3.89286	9.91E-05	0.000432
CNAG_06126	1014.206	-1.32129	0.201035	-6.57247	4.95E-11	7.61E-10
CNAG_06127	3099.008	-0.4338	0.15213	-2.85153	0.004351	0.012004
CNAG_06134	10528.66	0.363824	0.141279	2.57521	0.010018	0.024654

CNAG_07905	67.29487	1.06393	0.381111	2.791655	0.005244	0.014158
CNAG_06136	732.1771	1.055573	0.215534	4.897474	9.71E-07	6.74E-06
CNAG_06138	1776.579	-1.24752	0.240079	-5.19629	2.03E-07	1.62E-06
CNAG_06140	6148.501	-0.67284	0.300244	-2.24098	0.025027	0.05385
CNAG_06141	946.6629	-0.65122	0.223024	-2.91996	0.003501	0.009924
CNAG_06144	21051.57	-0.61666	0.194692	-3.16736	0.001538	0.004839
CNAG_06145	915.3392	0.348083	0.154177	2.257688	0.023965	0.052029
CNAG_06147	2455.513	-0.98659	0.317125	-3.11104	0.001864	0.005724
CNAG_06149	518.4282	-0.53977	0.159704	-3.37982	0.000725	0.002498
CNAG_06152	612.7142	1.130706	0.185968	6.080107	1.20E-09	1.51E-08
CNAG_06153	7399.439	0.736556	0.133324	5.524536	3.30E-08	3.19E-07
CNAG_06154	889.3086	0.493577	0.18024	2.738437	0.006173	0.016296
CNAG_06157	1171.873	-0.29795	0.145465	-2.04825	0.040536	0.081358
CNAG_06159	132.1555	1.006549	0.227981	4.415063	1.01E-05	5.64E-05
CNAG_06161	1459.184	0.821581	0.252866	3.249079	0.001158	0.003772
CNAG_06162	626.9209	-0.50777	0.255701	-1.98581	0.047055	0.092366
CNAG_06164	376.5253	0.352364	0.175765	2.004748	0.04499	0.088818
CNAG_06166	1547.86	0.672257	0.268199	2.506558	0.012191	0.02917
CNAG_08019	444.289	0.668652	0.2447	2.732536	0.006285	0.016575
CNAG_06167	4801.227	0.500575	0.134285	3.7277	0.000193	0.000784
CNAG_06168	4016.548	-0.80062	0.137687	-5.81475	6.07E-09	6.58E-08
CNAG_06169	3498.42	3.461289	0.18711	18.4987	2.12E-76	1.06E-73
CNAG_06170	3057.937	0.346565	0.162228	2.136285	0.032656	0.06761
CNAG_06175	7789.522	0.777484	0.250457	3.104261	0.001908	0.005848
CNAG_07908	2804.403	-1.89791	0.147455	-12.8712	6.54E-38	1.00E-35
CNAG_07909	88.58309	-1.34707	0.250223	-5.38346	7.31E-08	6.45E-07
CNAG_06180	2365.493	-1.35638	0.270036	-5.02295	5.09E-07	3.75E-06
CNAG_13075	35.35055	0.848194	0.360985	2.349665	0.01879	0.042201
CNAG_07910	5.882076	2.31634	0.950724	2.436395	0.014834	0.034545
CNAG_06183	934.3235	-0.44384	0.124988	-3.55107	0.000384	0.001433
CNAG_06184	585.2704	-0.48484	0.237172	-2.04427	0.040927	0.081999
CNAG_06186	1106.734	-0.52671	0.125657	-4.1916	2.77E-05	0.000138
CNAG_07911	212.5932	-1.42409	0.302941	-4.70089	2.59E-06	1.67E-05
CNAG_06188	877.4999	-0.89488	0.226961	-3.94288	8.05E-05	0.00036
CNAG_06190	1334.095	0.919909	0.181215	5.076347	3.85E-07	2.89E-06
CNAG_06191	586.5711	-0.60358	0.179464	-3.36324	0.00077	0.002633
CNAG_06193	1159.174	0.735561	0.361503	2.034729	0.041878	0.08357
CNAG_13077	20.19787	2.701082	0.543792	4.967122	6.80E-07	4.90E-06
CNAG_13078	6.798204	2.884407	1.07677	2.678758	0.00739	0.018945
CNAG_06200	1659.278	0.776619	0.188857	4.112201	3.92E-05	0.000189
CNAG_13080	140.3594	0.785872	0.316429	2.483568	0.013007	0.030816
CNAG_06201	2472.475	1.441931	0.159304	9.051466	1.41E-19	6.16E-18
CNAG_06202	90.30429	0.556949	0.260065	2.141572	0.032228	0.066891
CNAG_06203	256.5733	-0.68115	0.253815	-2.68366	0.007282	0.018732
CNAG_13082	700.1597	-1.73031	0.353364	-4.89669	9.75E-07	6.75E-06
CNAG_06204	5071.004	-2.05251	0.173423	-11.8353	2.56E-32	2.83E-30
CNAG_06205	4761.677	-0.3529	0.120193	-2.93613	0.003323	0.009479

CNAG_06206	2249.13	-0.79787	0.138334	-5.76768	8.04E-09	8.55E-08
CNAG_06207	1791.376	-1.35555	0.220985	-6.13413	8.56E-10	1.10E-08
CNAG_06208	37432.18	0.48886	0.106469	4.591577	4.40E-06	2.69E-05
CNAG_06213	1709.843	1.325273	0.249643	5.308672	1.10E-07	9.38E-07
CNAG_06214	2045.407	0.460413	0.173294	2.656837	0.007888	0.020024
CNAG_13084	46.06722	1.388079	0.349317	3.973695	7.08E-05	0.000321
CNAG_06216	1728.867	0.987316	0.182976	5.395868	6.82E-08	6.08E-07
CNAG_06217	200.6591	0.469319	0.172235	2.724872	0.006433	0.016875
CNAG_06220	12864.59	2.105051	0.136374	15.43592	9.39E-54	2.76E-51
CNAG_06221	914.1366	-0.79711	0.152358	-5.23184	1.68E-07	1.37E-06
CNAG_06222	35383.36	-1.06794	0.283588	-3.76582	0.000166	0.000687
CNAG_06226	3702.119	-2.01692	0.3153	-6.39681	1.59E-10	2.29E-09
CNAG_06227	1732.178	0.966422	0.184572	5.23601	1.64E-07	1.35E-06
CNAG_06228	1920.62	-0.49667	0.145015	-3.42493	0.000615	0.00217
CNAG_13087	26.0759	-2.12825	0.487208	-4.36827	1.25E-05	6.85E-05
CNAG_06231	37350.46	-0.85743	0.213499	-4.01609	5.92E-05	0.000272
CNAG_06232	870.0846	0.433535	0.186481	2.324823	0.020081	0.044747
CNAG_13089	229.8415	1.041675	0.171103	6.088011	1.14E-09	1.44E-08
CNAG_06235	2154.112	-0.42341	0.133619	-3.16882	0.001531	0.004822
CNAG_06237	2380.129	0.450442	0.16523	2.72615	0.006408	0.016832
CNAG_06238	646.6783	3.131871	0.266533	11.75039	7.03E-32	7.45E-30
CNAG_06239	240.7518	0.431963	0.179218	2.410273	0.015941	0.036735
CNAG_06240	14961.17	0.904018	0.133196	6.787123	1.14E-11	1.98E-10
CNAG_06244	1618.165	0.583383	0.152404	3.827874	0.000129	0.000547
CNAG_06247	165.1945	0.401317	0.179171	2.239856	0.0251	0.053977
CNAG_06249	413.2646	0.37827	0.169186	2.235819	0.025364	0.0545
CNAG_06252	442.9206	-0.61124	0.175753	-3.47782	0.000506	0.00183
CNAG_06259	319.8805	-0.76724	0.201759	-3.80278	0.000143	0.000601
CNAG_06260	1718.149	-0.66465	0.12309	-5.39971	6.67E-08	5.99E-07
CNAG_07921	1005.524	-0.31947	0.140477	-2.27418	0.022955	0.050258
CNAG_13097	435.6443	-0.59664	0.201148	-2.96617	0.003015	0.008719
CNAG_06263	23.39551	1.035494	0.419021	2.471223	0.013465	0.031721
CNAG_06265	541.8821	0.390913	0.139963	2.792976	0.005223	0.014117
CNAG_06267	3292.705	3.365344	0.177185	18.99334	1.94E-80	1.28E-77
CNAG_13099	788.1813	1.418284	0.203671	6.963599	3.32E-12	6.20E-11
CNAG_07923	20.77869	1.451521	0.469942	3.08872	0.00201	0.006134
CNAG_13100	91.06707	-0.69781	0.239542	-2.91308	0.003579	0.010113
CNAG_07924	1434.057	-0.42164	0.212004	-1.98883	0.04672	0.0918
CNAG_06272	612.3673	0.568778	0.192517	2.954427	0.003132	0.008992
CNAG_06273	2168.118	-0.58699	0.291287	-2.01517	0.043887	0.087029
CNAG_06274	8273.175	-0.85132	0.238435	-3.57043	0.000356	0.001343
CNAG_06277	3054.206	-0.36876	0.167931	-2.19592	0.028098	0.059426
CNAG_06279	2921.811	-0.46623	0.161815	-2.88128	0.003961	0.011019
CNAG_06283	1128.035	-0.59031	0.160692	-3.67357	0.000239	0.000947
CNAG_06285	533.7644	1.296339	0.20314	6.381512	1.75E-10	2.50E-09
CNAG_06286	349.499	1.158729	0.157894	7.338638	2.16E-13	4.79E-12
CNAG_06288	2157.494	-0.73245	0.187197	-3.91271	9.13E-05	0.000403

CNAG_06289	2277.747	0.484451	0.133483	3.629314	0.000284	0.001105
CNAG_13106	60.40101	1.533679	0.281956	5.439419	5.35E-08	4.87E-07
CNAG_06290	411.6428	1.695978	0.21664	7.828571	4.93E-15	1.35E-13
CNAG_06291	148.4142	1.381843	0.195752	7.059161	1.68E-12	3.37E-11
CNAG_06292	57.38041	0.82824	0.308655	2.683383	0.007288	0.018735
CNAG_06293	415.6124	-0.59243	0.147877	-4.00624	6.17E-05	0.000283
CNAG_06296	63.86876	2.278038	0.317954	7.164689	7.80E-13	1.64E-11
CNAG_06297	932.2337	-1.92132	0.264218	-7.27172	3.55E-13	7.79E-12
CNAG_06298	3401.578	-2.08395	0.323475	-6.44237	1.18E-10	1.73E-09
CNAG_13108	57.32374	1.317113	0.433538	3.038056	0.002381	0.007124
CNAG_07928	68.68854	-1.57782	0.35239	-4.47749	7.55E-06	4.33E-05
CNAG_06301	6322.571	0.443652	0.135735	3.268512	0.001081	0.003543
CNAG_06302	137.8962	1.284116	0.244095	5.260733	1.43E-07	1.19E-06
CNAG_06308	420.3452	-0.54735	0.169154	-3.23583	0.001213	0.003935
CNAG_06309	142.0079	0.924794	0.239301	3.864559	0.000111	0.000478
CNAG_06310	789.182	0.87386	0.130976	6.671922	2.52E-11	4.09E-10
CNAG_06311	1112.997	0.356964	0.178523	1.999534	0.045551	0.089768
CNAG_06314	8698.309	-0.59132	0.119453	-4.95021	7.41E-07	5.29E-06
CNAG_06315	2795.986	-0.68052	0.191594	-3.55191	0.000382	0.001429
CNAG_06316	3328.23	-0.55621	0.177848	-3.12746	0.001763	0.00546
CNAG_06318	2126.87	-0.86375	0.227016	-3.80478	0.000142	0.000597
CNAG_06320	3235.558	0.532709	0.142233	3.745322	0.00018	0.000739
CNAG_06321	106.8702	1.19764	0.329706	3.632444	0.000281	0.001093
CNAG_06322	601.2305	-0.94998	0.152268	-6.23884	4.41E-10	5.89E-09
CNAG_06324	542.2937	-1.24923	0.248597	-5.02512	5.03E-07	3.72E-06
CNAG_06325	2251.212	-0.63112	0.132015	-4.78066	1.75E-06	1.15E-05
CNAG_06326	1033.987	0.641688	0.154323	4.158078	3.21E-05	0.000158
CNAG_06330	295.86	-0.53105	0.179171	-2.96395	0.003037	0.008766
CNAG_06332	2038.55	0.315509	0.11802	2.673354	0.00751	0.019175
CNAG_06334	2301.863	1.347586	0.214537	6.281366	3.36E-10	4.60E-09
CNAG_06335	1238.854	-0.79591	0.211146	-3.76949	0.000164	0.000678
CNAG_13118	42.36409	2.039625	0.363064	5.617819	1.93E-08	1.94E-07
CNAG_06339	637.4768	0.928483	0.220091	4.218625	2.46E-05	0.000124
CNAG_06340	2042.357	-0.80534	0.225198	-3.57615	0.000349	0.001319
CNAG_06341	825.5964	-1.1517	0.264872	-4.34812	1.37E-05	7.46E-05
CNAG_06345	897.3282	-0.62078	0.184374	-3.36696	0.00076	0.002605
CNAG_06346	6430.218	-2.01435	0.330282	-6.09887	1.07E-09	1.35E-08
CNAG_06347	12077.16	3.038419	0.195229	15.56338	1.29E-54	3.95E-52
CNAG_06352	1625.174	-0.8465	0.236066	-3.58586	0.000336	0.001277
CNAG_06353	2554.499	-0.55379	0.121932	-4.54183	5.58E-06	3.31E-05
CNAG_06355	606.064	0.583958	0.266376	2.192229	0.028363	0.059892
CNAG_06357	547.6756	-0.72249	0.280348	-2.57711	0.009963	0.024548
CNAG_06358	380.7226	-0.68776	0.258889	-2.6566	0.007893	0.020032
CNAG_06359	483.8197	0.404945	0.188033	2.15359	0.031272	0.065135
CNAG_06361	6105.973	0.762812	0.114658	6.652931	2.87E-11	4.60E-10
CNAG_06362	1346.303	0.672	0.255441	2.630749	0.00852	0.02141
CNAG_06363	1021.016	-1.04835	0.167147	-6.272	3.56E-10	4.83E-09

CNAG_06365	1019.452	0.839769	0.145452	5.773511	7.76E-09	8.28E-08
CNAG_06367	3024.776	-0.73384	0.130135	-5.63905	1.71E-08	1.72E-07
CNAG_06368	640.3864	-0.77468	0.173437	-4.46665	7.95E-06	4.53E-05
CNAG_06369	754.0376	-0.43042	0.145876	-2.95056	0.003172	0.009086
CNAG_06370	3457.632	-1.19297	0.164717	-7.24257	4.40E-13	9.56E-12
CNAG_06372	354.9425	0.982051	0.264499	3.712869	0.000205	0.000826
CNAG_06374	6527.991	-1.28057	0.155636	-8.22797	1.90E-16	6.18E-15
CNAG_06377	38960.21	-0.40878	0.194896	-2.09742	0.035956	0.073542
CNAG_06378	501.3514	-0.48618	0.174566	-2.78509	0.005351	0.014414
CNAG_06379	2866.844	-0.44546	0.175921	-2.53217	0.011336	0.027379
CNAG_06381	8933.18	0.834342	0.136146	6.128289	8.88E-10	1.14E-08
CNAG_06382	500.731	0.481396	0.152411	3.158534	0.001586	0.004978
CNAG_06385	623.5707	-0.77878	0.166137	-4.6876	2.76E-06	1.77E-05
CNAG_06386	1053.831	-0.63848	0.184874	-3.45362	0.000553	0.001983
CNAG_06388	4302.2	-1.54726	0.27969	-5.53207	3.16E-08	3.07E-07
CNAG_13123	23.26738	-1.96325	0.534751	-3.67133	0.000241	0.000953
CNAG_06389	1362.178	-0.74832	0.264451	-2.82971	0.004659	0.012731
CNAG_13124	94.62026	1.110744	0.264588	4.198018	2.69E-05	0.000135
CNAG_06391	94.05762	-0.72245	0.268947	-2.68622	0.007227	0.018609
CNAG_06392	1019.187	-0.83708	0.199061	-4.20516	2.61E-05	0.000132
CNAG_06396	1048.257	3.173816	0.266843	11.89393	1.27E-32	1.47E-30
CNAG_07936	2102.048	0.818465	0.263792	3.10269	0.001918	0.005874
CNAG_06399	897.9589	1.138161	0.32306	3.523063	0.000427	0.001571
CNAG_06400	56227.19	-0.52802	0.205023	-2.57541	0.010012	0.024653
CNAG_13126	19.4759	-1.4196	0.526736	-2.6951	0.007037	0.018207
CNAG_13128	106.3671	3.387127	1.352238	2.50483	0.012251	0.029304
CNAG_06404	1999.299	-1.18866	0.22808	-5.21159	1.87E-07	1.52E-06
CNAG_06406	874.7334	-0.65306	0.164746	-3.96405	7.37E-05	0.000332
CNAG_06407	2842.926	-0.85069	0.255902	-3.3243	0.000886	0.002974
CNAG_06411	195.1836	0.730979	0.19089	3.829322	0.000128	0.000545
CNAG_06412	981.6674	-0.54322	0.165447	-3.28335	0.001026	0.003395
CNAG_06413	181.7171	0.408808	0.176771	2.312647	0.020742	0.045923
CNAG_06414	476.2503	-0.60841	0.224833	-2.70606	0.006809	0.017692
CNAG_06415	1528.64	0.285204	0.124951	2.282524	0.022458	0.049256
CNAG_06421	3831.404	-1.09497	0.255159	-4.29132	1.78E-05	9.34E-05
CNAG_06425	711.4704	-0.66686	0.166158	-4.01339	5.99E-05	0.000275
CNAG_06426	985.6855	-0.80201	0.262348	-3.05706	0.002235	0.006745
CNAG_06427	880.1445	1.376919	0.241496	5.701624	1.19E-08	1.23E-07
CNAG_06428	1757.216	1.301173	0.138211	9.414419	4.76E-21	2.41E-19
CNAG_07937	2802.608	-0.32997	0.128188	-2.57414	0.010049	0.024711
CNAG_07938	1366.399	1.159973	0.314127	3.692695	0.000222	0.000885
CNAG_06431	812.7307	0.400616	0.150482	2.662214	0.007763	0.019739
CNAG_06432	7099.155	1.219491	0.125194	9.740839	2.02E-22	1.17E-20
CNAG_07939	13828.06	1.858162	0.338151	5.495066	3.91E-08	3.69E-07
CNAG_13131	59.94245	0.637594	0.264047	2.414697	0.015748	0.036366
CNAG_06439	2942.163	-0.41347	0.18849	-2.19362	0.028263	0.059727
CNAG_07940	386.5895	0.807379	0.235411	3.429662	0.000604	0.002139

CNAG_06443	56821.23	1.160644	0.107421	10.80463	3.27E-27	2.55E-25
CNAG_06446	2315.881	-0.32149	0.110798	-2.90164	0.003712	0.010438
CNAG_06447	29575.62	-0.91816	0.219904	-4.17526	2.98E-05	0.000148
CNAG_06449	133.3932	0.774026	0.204063	3.793076	0.000149	0.000621
CNAG_06452	1855.223	0.852943	0.136154	6.264543	3.74E-10	5.05E-09
CNAG_06453	7541.479	2.730502	0.129343	21.11051	6.37E-99	7.23E-96
CNAG_06456	2472.182	-0.86845	0.121664	-7.13811	9.46E-13	1.98E-11
CNAG_07941	2535.349	-0.38748	0.186825	-2.07403	0.038076	0.077182
CNAG_06459	2226.76	1.19563	0.155421	7.692856	1.44E-14	3.70E-13
CNAG_06460	3299.548	0.946699	0.129437	7.313998	2.59E-13	5.74E-12
CNAG_06464	1173.49	-0.5	0.193363	-2.58581	0.009715	0.024004
CNAG_06466	361.8598	-0.75581	0.198522	-3.80716	0.000141	0.000591
CNAG_06468	3674.305	-0.82347	0.164783	-4.99731	5.81E-07	4.24E-06
CNAG_06471	1242.061	-0.76278	0.209756	-3.63653	0.000276	0.001077
CNAG_06472	3990.732	-0.63595	0.139719	-4.55166	5.32E-06	3.18E-05
CNAG_06473	1551.469	0.489417	0.110366	4.434512	9.23E-06	5.19E-05
CNAG_06474	6189.762	-0.48215	0.146917	-3.28178	0.001032	0.003408
CNAG_13133	37.47929	0.970884	0.362653	2.677169	0.007425	0.019019
CNAG_06482	586.764	0.573898	0.143326	4.004139	6.22E-05	0.000285
CNAG_06485	294.5617	-0.64365	0.183384	-3.50985	0.000448	0.00164
CNAG_06487	1065.241	-1.04448	0.227664	-4.5878	4.48E-06	2.73E-05
CNAG_07943	1329.717	2.145612	0.213901	10.03086	1.12E-23	7.21E-22
CNAG_06490	2314.595	0.455175	0.178314	2.552663	0.01069	0.026041
CNAG_06491	153.1315	-0.69598	0.283343	-2.45633	0.014037	0.032919
CNAG_06492	539.8058	-0.81979	0.18169	-4.51203	6.42E-06	3.77E-05
CNAG_06493	1790.371	1.254108	0.311529	4.025649	5.68E-05	0.000263
CNAG_06494	1830.774	1.405078	0.167806	8.373223	5.61E-17	1.92E-15
CNAG_06497	930.6993	0.835848	0.132021	6.331189	2.43E-10	3.41E-09
CNAG_06498	1432.193	2.17816	0.185407	11.74798	7.23E-32	7.56E-30
CNAG_06499	999.7608	0.292129	0.13451	2.171811	0.02987	0.062608
CNAG_06500	719.1125	-0.89882	0.167981	-5.35072	8.76E-08	7.64E-07
CNAG_06503	65.74907	0.758158	0.327381	2.31583	0.020568	0.045613
CNAG_06506	582.4857	-0.28605	0.129756	-2.20454	0.027486	0.05835
CNAG_06510	780.8659	-0.31709	0.148683	-2.13265	0.032953	0.068115
CNAG_06511	1996.767	1.153711	0.22686	5.085555	3.67E-07	2.77E-06
CNAG_06512	879.3628	0.956637	0.177665	5.384505	7.26E-08	6.42E-07
CNAG_06515	379.1845	0.620689	0.189492	3.275542	0.001055	0.003468
CNAG_13141	217.0418	-1.33392	0.266881	-4.99818	5.79E-07	4.22E-06
CNAG_06517	4082.058	-1.95726	0.212586	-9.20689	3.36E-20	1.58E-18
CNAG_06520	482.4641	-1.07502	0.234513	-4.58405	4.56E-06	2.77E-05
CNAG_06521	170.6628	0.599952	0.207118	2.896659	0.003772	0.010571
CNAG_07043	391.3022	0.603479	0.153672	3.927062	8.60E-05	0.000383
CNAG_05336	1234.458	0.400282	0.125079	3.200225	0.001373	0.004382
CNAG_05337	1176.836	0.342231	0.155862	2.195727	0.028111	0.059439
CNAG_05339	2355.017	0.70564	0.189213	3.729335	0.000192	0.000781
CNAG_05340	460.7888	-0.7862	0.185109	-4.24723	2.16E-05	0.000111
CNAG_05341	285.3404	1.7288	0.194595	8.884094	6.44E-19	2.65E-17

CNAG_13144	21.71922	1.453459	0.558763	2.601209	0.00929	0.023082
CNAG_05345	8.196203	1.955611	0.744452	2.626915	0.008616	0.021625
CNAG_07869	18.85935	-3.60089	0.673683	-5.34508	9.04E-08	7.86E-07
CNAG_05348	5560.847	-0.52439	0.192962	-2.71755	0.006577	0.017173
CNAG_05349	5341.014	0.539085	0.159963	3.370062	0.000752	0.002581
CNAG_13146	48.12884	0.987861	0.310202	3.184576	0.00145	0.004587
CNAG_05351	14355.84	0.408462	0.203563	2.006559	0.044797	0.088458
CNAG_05352	2695.088	1.110914	0.13381	8.302159	1.02E-16	3.41E-15
CNAG_05354	1012.783	-0.79962	0.168086	-4.7572	1.96E-06	1.28E-05
CNAG_05355	5677.648	0.307023	0.123087	2.494358	0.012619	0.030029
CNAG_13147	29.38503	-1.17994	0.422328	-2.79388	0.005208	0.014085
CNAG_05358	64.57686	-1.89891	0.43404	-4.37496	1.21E-05	6.67E-05
CNAG_05359	985.9689	-0.41759	0.127908	-3.26476	0.001096	0.003587
CNAG_05360	1777.286	-0.73964	0.127321	-5.80926	6.28E-09	6.77E-08
CNAG_05365	2018.331	-0.53226	0.187051	-2.84554	0.004434	0.012203
CNAG_05366	6785.339	-0.65673	0.129229	-5.08194	3.74E-07	2.82E-06
CNAG_05367	2255.964	-1.18798	0.275188	-4.31696	1.58E-05	8.43E-05
CNAG_07873	1501.848	-0.73823	0.197259	-3.74243	0.000182	0.000746
CNAG_05369	476.8578	-0.34596	0.147445	-2.3464	0.018956	0.042509
CNAG_05370	782.886	0.567144	0.12199	4.649105	3.33E-06	2.09E-05
CNAG_05371	1575.282	-0.71182	0.14877	-4.7847	1.71E-06	1.13E-05
CNAG_05372	3332.508	-0.37412	0.121717	-3.07372	0.002114	0.006424
CNAG_05377	52.6942	1.484371	0.372296	3.987072	6.69E-05	0.000305
CNAG_05383	1829.157	2.825741	0.226704	12.46444	1.17E-35	1.60E-33
CNAG_13157	21.93735	1.627507	0.455399	3.573808	0.000352	0.001329
CNAG_05387	7806.013	-0.78381	0.136408	-5.74606	9.13E-09	9.64E-08
CNAG_13158	9.839205	2.278478	0.708272	3.216953	0.001296	0.004176
CNAG_05388	2323.508	0.759412	0.165958	4.575929	4.74E-06	2.86E-05
CNAG_05390	718.6779	0.383561	0.136394	2.812151	0.004921	0.013378
CNAG_05391	1535.427	-1.25282	0.141906	-8.82854	1.06E-18	4.26E-17
CNAG_13160	15.0444	-1.94619	0.741738	-2.62383	0.008695	0.021767
CNAG_05392	1151.504	-2.90686	0.315878	-9.20248	3.50E-20	1.63E-18
CNAG_05393	1028.198	-0.37608	0.136892	-2.74726	0.00601	0.015923
CNAG_05395	1206.728	0.737694	0.181139	4.072526	4.65E-05	0.000219
CNAG_05398	4469.195	-1.1441	0.192429	-5.94558	2.75E-09	3.23E-08
CNAG_05402	2417.497	-1.20139	0.225	-5.33949	9.32E-08	8.07E-07
CNAG_05406	305.1098	-0.75317	0.169043	-4.45548	8.37E-06	4.76E-05
CNAG_05408	4268.553	-0.29063	0.119983	-2.42225	0.015425	0.035733
CNAG_05410	1473.846	1.659787	0.233519	7.107722	1.18E-12	2.43E-11
CNAG_05411	2035.898	0.651267	0.123031	5.293524	1.20E-07	1.01E-06
CNAG_05412	50.85807	3.338141	0.44713	7.465711	8.29E-14	1.94E-12
CNAG_05414	260.1327	-0.37299	0.156005	-2.39092	0.016806	0.038385
CNAG_05415	1412.379	-1.42596	0.211097	-6.755	1.43E-11	2.43E-10
CNAG_05416	4239.082	-0.45033	0.140864	-3.19687	0.001389	0.004426
CNAG_05419	2480.536	1.071717	0.209885	5.106199	3.29E-07	2.50E-06
CNAG_05420	401.7238	-0.72895	0.242854	-3.00161	0.002686	0.007921
CNAG_13168	114.9943	0.667412	0.250212	2.667382	0.007644	0.019475

CNAG_05423	372.4674	-0.51336	0.144653	-3.54891	0.000387	0.001444
CNAG_05425	14780.77	-1.19959	0.160555	-7.4715	7.93E-14	1.87E-12
CNAG_05426	128.6912	1.153377	0.281234	4.101129	4.11E-05	0.000197
CNAG_13172	76.14692	-0.8265	0.314183	-2.63063	0.008523	0.02141
CNAG_05430	838.4851	0.726947	0.187209	3.883079	0.000103	0.000447
CNAG_05431	1306.178	-0.50484	0.15409	-3.27627	0.001052	0.003462
CNAG_05434	2424.538	-0.8864	0.193376	-4.58384	4.57E-06	2.77E-05
CNAG_05435	875.4889	-0.59459	0.152569	-3.89721	9.73E-05	0.000425
CNAG_05436	121.2127	-0.57433	0.247691	-2.31875	0.020409	0.045349
CNAG_05437	14317.64	-0.58714	0.168778	-3.47878	0.000504	0.001824
CNAG_05438	597.1646	-1.41719	0.236837	-5.98381	2.18E-09	2.62E-08
CNAG_05439	749.8201	0.566315	0.12925	4.381556	1.18E-05	6.50E-05
CNAG_05440	1261.769	-0.56587	0.195059	-2.90101	0.00372	0.010455
CNAG_05441	493.9045	0.730794	0.138612	5.272232	1.35E-07	1.12E-06
CNAG_05442	1919.28	-0.33986	0.159036	-2.13702	0.032596	0.067503
CNAG_05443	160.2872	0.53179	0.230902	2.303097	0.021273	0.046981
CNAG_05446	2860.153	-0.30323	0.115284	-2.63028	0.008531	0.021426
CNAG_07876	90.68279	0.908639	0.278003	3.268453	0.001081	0.003543
CNAG_05449	10619.71	0.85196	0.23067	3.693418	0.000221	0.000883
CNAG_05450	336.9453	0.630339	0.199618	3.157731	0.00159	0.004988
CNAG_05454	58.16226	1.637859	0.548155	2.987946	0.002809	0.008226
CNAG_05455	8739.483	-0.85008	0.332683	-2.55522	0.010612	0.02589
CNAG_05456	745.882	-0.29129	0.127316	-2.28791	0.022143	0.048618
CNAG_05457	5402.298	1.245138	0.207297	6.006551	1.90E-09	2.30E-08
CNAG_05458	241.5273	2.113513	0.22222	9.510893	1.89E-21	1.00E-19
CNAG_05460	466.3211	0.432763	0.15101	2.865795	0.00416	0.011521
CNAG_05463	2138.325	0.366908	0.177522	2.066837	0.03875	0.078173
CNAG_05465	30504.12	-0.85463	0.172303	-4.96003	7.05E-07	5.05E-06
CNAG_08025	1826.996	-0.51079	0.140905	-3.62503	0.000289	0.001121
CNAG_05468	1088.738	-0.39832	0.141283	-2.81933	0.004812	0.013109
CNAG_05469	2444.482	0.35664	0.113566	3.140381	0.001687	0.005252
CNAG_05473	469.8033	-0.64312	0.165023	-3.89717	9.73E-05	0.000425
CNAG_13181	99.30572	0.621687	0.256491	2.423822	0.015358	0.0356
CNAG_05475	4689.589	0.721184	0.178105	4.049207	5.14E-05	0.00024
CNAG_05476	344.2489	-0.72573	0.280886	-2.58373	0.009774	0.024137
CNAG_05477	1964.382	-0.34154	0.143692	-2.3769	0.017459	0.039648
CNAG_05478	1949.054	-0.35756	0.157708	-2.26723	0.023376	0.05103
CNAG_05482	437.1089	0.423493	0.146786	2.885106	0.003913	0.010894
CNAG_08026	218.4035	-0.69486	0.214657	-3.23707	0.001208	0.003922
CNAG_05496	6737.925	-0.47808	0.140455	-3.4038	0.000665	0.002321
CNAG_05497	11447.4	-1.43375	0.156897	-9.13814	6.35E-20	2.87E-18
CNAG_05499	3377.748	-0.88185	0.164542	-5.35939	8.35E-08	7.31E-07
CNAG_05500	519.6682	0.603422	0.163597	3.688462	0.000226	0.000899
CNAG_05505	744.9845	0.396284	0.137291	2.886455	0.003896	0.010859
CNAG_07884	4060.901	-0.84215	0.151634	-5.55382	2.79E-08	2.74E-07
CNAG_05507	2089.043	-0.5068	0.197775	-2.5625	0.010392	0.025463
CNAG_05509	1970.154	-1.45514	0.212238	-6.85618	7.07E-12	1.28E-10

CNAG_05512	1658.04	-0.62618	0.119697	-5.23139	1.68E-07	1.37E-06
CNAG_05515	3416.793	0.692848	0.136467	5.077033	3.83E-07	2.88E-06
CNAG_05516	927.9199	-0.672	0.158997	-4.22649	2.37E-05	0.000121
CNAG_05521	3706.543	0.867959	0.165852	5.233334	1.66E-07	1.36E-06
CNAG_13188	142.1512	1.731098	0.202999	8.527626	1.49E-17	5.32E-16
CNAG_05525	40304.53	-1.09579	0.280453	-3.90721	9.34E-05	0.000411
CNAG_05528	18.51503	1.331854	0.470082	2.83324	0.004608	0.012608
CNAG_05531	1941.923	0.728363	0.268745	2.71024	0.006723	0.017516
CNAG_05534	1079.795	0.562667	0.175007	3.215115	0.001304	0.0042
CNAG_05535	1554.425	-0.50938	0.234873	-2.16876	0.030101	0.063009
CNAG_05537	963.9415	-1.09571	0.164222	-6.67213	2.52E-11	4.09E-10
CNAG_05543	125.647	1.069929	0.235523	4.54278	5.55E-06	3.30E-05
CNAG_05544	1477.376	2.895072	0.235966	12.26904	1.33E-34	1.68E-32
CNAG_05546	989.0788	-0.47309	0.219027	-2.15997	0.030775	0.06425
CNAG_05547	1003.038	3.325793	0.261485	12.71889	4.64E-37	6.59E-35
CNAG_05548	390.3206	1.091659	0.224335	4.866193	1.14E-06	7.75E-06
CNAG_13192	31.5853	1.573643	0.445794	3.529977	0.000416	0.001538
CNAG_05553	586.0468	-0.49881	0.15533	-3.21131	0.001321	0.004245
CNAG_05554	2458.293	-0.96187	0.301664	-3.18854	0.00143	0.004536
CNAG_05555	43697.33	-0.95473	0.184867	-5.16443	2.41E-07	1.89E-06
CNAG_05556	14241.7	-0.98946	0.282359	-3.50425	0.000458	0.001669
CNAG_08027	805.1203	0.361971	0.122052	2.965724	0.00302	0.008729
CNAG_05563	602.0247	-0.80455	0.167251	-4.81041	1.51E-06	1.01E-05
CNAG_05565	228.9488	-2.11382	0.296098	-7.13894	9.41E-13	1.97E-11
CNAG_05572	1028.649	0.405396	0.184332	2.19927	0.027859	0.059031
CNAG_05573	1110.477	0.763613	0.212626	3.591346	0.000329	0.001256
CNAG_05576	697.9617	-1.28764	0.17608	-7.31284	2.62E-13	5.77E-12
CNAG_05578	1660.659	-0.78208	0.24548	-3.18591	0.001443	0.004568
CNAG_05581	4458.413	0.849074	0.270241	3.141907	0.001679	0.005232
CNAG_05583	870.7321	-0.55562	0.149444	-3.71794	0.000201	0.000811
CNAG_13195	15.29305	-1.48905	0.568213	-2.62059	0.008778	0.021934
CNAG_05584	969.3965	-0.25854	0.129157	-2.00178	0.045309	0.089358
CNAG_05586	573.658	0.306531	0.143562	2.135183	0.032746	0.067764
CNAG_07887	2231.705	0.776978	0.252663	3.075148	0.002104	0.006396
CNAG_05590	3438.415	0.499968	0.195881	2.552406	0.010698	0.026052
CNAG_05592	12731.55	1.292441	0.152932	8.451063	2.89E-17	1.01E-15
CNAG_08028	742.1318	-0.55734	0.133392	-4.17821	2.94E-05	0.000146
CNAG_05595	1253.771	0.84394	0.163921	5.148472	2.63E-07	2.04E-06
CNAG_05596	3583.947	-0.66314	0.179026	-3.70419	0.000212	0.000852
CNAG_05598	1708.42	0.787082	0.152835	5.149891	2.61E-07	2.03E-06
CNAG_05599	575.9312	0.561899	0.169216	3.320599	0.000898	0.003008
CNAG_05600	1887.788	-0.45613	0.145509	-3.13469	0.00172	0.005338
CNAG_05602	716.9023	-0.36951	0.166777	-2.21559	0.02672	0.056936
CNAG_05603	865.2206	-0.8822	0.143829	-6.13366	8.59E-10	1.10E-08
CNAG_05606	699.5317	-0.70113	0.258224	-2.71518	0.006624	0.017285
CNAG_05607	3311.086	1.047489	0.204112	5.131936	2.87E-07	2.21E-06
CNAG_05608	807.4946	1.644594	0.197268	8.336836	7.63E-17	2.57E-15

CNAG_05609	1423.344	-0.46356	0.230092	-2.01467	0.04394	0.087112
CNAG_05610	506.6208	-0.48104	0.228112	-2.10879	0.034963	0.071676
CNAG_07889	1414.737	-0.67104	0.160229	-4.18799	2.81E-05	0.00014
CNAG_05613	3409.191	0.535114	0.156846	3.41171	0.000646	0.002267
CNAG_05614	1770.891	-0.46526	0.193103	-2.40938	0.01598	0.036783
CNAG_05616	2910.782	1.254246	0.257611	4.868766	1.12E-06	7.66E-06
CNAG_05618	2642.67	-0.49837	0.12225	-4.07665	4.57E-05	0.000216
CNAG_05619	1200.143	-0.25698	0.12409	-2.0709	0.038368	0.077615
CNAG_05621	1194.684	-0.50567	0.140874	-3.58951	0.000331	0.001263
CNAG_05623	5429.848	-0.826	0.168279	-4.90853	9.18E-07	6.40E-06
CNAG_05624	967.305	-1.02392	0.160417	-6.38289	1.74E-10	2.48E-09
CNAG_08029	617.3068	-0.42228	0.147957	-2.85407	0.004316	0.011921
CNAG_05627	978.1637	-1.00693	0.161693	-6.22742	4.74E-10	6.28E-09
CNAG_05630	1318.4	-0.47269	0.139382	-3.39131	0.000696	0.002411
CNAG_05631	8593.795	-1.10523	0.125015	-8.84082	9.50E-19	3.83E-17
CNAG_05632	365.2155	-0.49669	0.151459	-3.27938	0.00104	0.003433
CNAG_13197	149.4965	0.5822	0.22706	2.564081	0.010345	0.025355
CNAG_05633	2976.404	-0.92936	0.209469	-4.43673	9.13E-06	5.14E-05
CNAG_05635	516.9609	-0.82675	0.32426	-2.54966	0.010783	0.026234
CNAG_05638	6296.089	0.724908	0.201922	3.590041	0.000331	0.001261
CNAG_05639	1732.984	0.53986	0.153545	3.515968	0.000438	0.001605
CNAG_05640	2019.904	-0.5167	0.163871	-3.15307	0.001616	0.005062
CNAG_05641	41.41962	2.965132	0.479479	6.184065	6.25E-10	8.14E-09
CNAG_13200	40.93158	1.760762	0.380462	4.627954	3.69E-06	2.29E-05
CNAG_05647	942.9512	-0.64101	0.149239	-4.29521	1.75E-05	9.21E-05
CNAG_05649	440.28	0.484941	0.197955	2.449758	0.014295	0.033468
CNAG_05652	2589.538	1.081696	0.146228	7.397343	1.39E-13	3.13E-12
CNAG_05653	13908.59	-1.34262	0.167447	-8.01817	1.07E-15	3.23E-14
CNAG_05654	345.4224	1.955974	0.241228	8.108411	5.13E-16	1.59E-14
CNAG_05655	776.1542	-0.79613	0.268676	-2.96314	0.003045	0.008783
CNAG_05657	1537.307	-0.72231	0.217526	-3.32057	0.000898	0.003008
CNAG_05658	114.9674	-2.88894	0.271267	-10.6498	1.75E-26	1.27E-24
CNAG_05660	2354.96	-0.48436	0.137509	-3.52235	0.000428	0.001575
CNAG_13202	15.79204	1.862923	0.772894	2.410323	0.015938	0.036735
CNAG_13203	214.6857	1.192433	0.302236	3.945372	7.97E-05	0.000357
CNAG_05662	3319.274	-2.74454	0.243948	-11.2505	2.30E-29	2.08E-27
CNAG_05664	3707.795	-0.57878	0.140434	-4.12138	3.77E-05	0.000183
CNAG_13204	317.5297	0.767405	0.244262	3.141723	0.00168	0.005234
CNAG_05665	2832.545	0.748514	0.128603	5.820336	5.87E-09	6.39E-08
CNAG_13206	395.6514	1.351681	0.200623	6.737403	1.61E-11	2.71E-10
CNAG_07892	217.6739	-0.9129	0.343188	-2.66005	0.007813	0.019853
CNAG_11003	3.85239	4.316338	2.021551	2.135162	0.032748	0.067764
CNAG_11005	5.992957	4.010114	1.749958	2.291549	0.021932	0.048181
CNAG_11016	5.231253	3.906606	1.718029	2.273888	0.022973	0.050273

Table S8. Data sheets of the RNA-seq data for the WT vs. *cir1Δ* mutant.

	baseMean	log2FoldCh	lfcSE	stat	pvalue	padj
CNAG_04548	29.49797	1.849894	0.413161	4.477413	7.56E-06	3.42E-05
CNAG_07304	568.791	-0.72459	0.119374	-6.06994	1.28E-09	9.29E-09
CNAG_07305	59.90067	-0.70012	0.310414	-2.25544	0.024106	0.049907
CNAG_00002	708.4145	-0.77868	0.09605	-8.10701	5.19E-16	6.05E-15
CNAG_00003	491.0802	-0.64586	0.10644	-6.06786	1.30E-09	9.40E-09
CNAG_00004	832.1575	-0.25856	0.094413	-2.73863	0.00617	0.015028
CNAG_12001	99.62981	2.13244	0.273079	7.808872	5.77E-15	6.32E-14
CNAG_00006	3855.291	-0.27477	0.067321	-4.08146	4.48E-05	0.000175
CNAG_00007	4144.377	-0.29968	0.061216	-4.89547	9.81E-07	5.04E-06
CNAG_00009	891.8573	-0.34468	0.091836	-3.75323	0.000175	0.000608
CNAG_00010	4925.036	1.257745	0.067484	18.6376	1.59E-77	8.51E-76
CNAG_00011	1064.399	1.458651	0.09982	14.61282	2.33E-48	7.63E-47
CNAG_00012	1785.724	-0.22546	0.079456	-2.83754	0.004546	0.011476
CNAG_00013	397.1122	-0.28382	0.115604	-2.45515	0.014082	0.03124
CNAG_00015	232.4405	0.315939	0.148986	2.1206	0.033956	0.067069
CNAG_00016	2912.48	-0.47658	0.0676	-7.05004	1.79E-12	1.68E-11
CNAG_00018	2384.28	0.415973	0.088753	4.686838	2.77E-06	1.34E-05
CNAG_00021	1051.99	-0.30558	0.097012	-3.14992	0.001633	0.00458
CNAG_00022	2120.207	1.200887	0.099259	12.09849	1.08E-33	2.44E-32
CNAG_00023	207.3176	0.563018	0.16586	3.394536	0.000687	0.002108
CNAG_00024	13749.08	-0.13196	0.062593	-2.10824	0.03501	0.068868
CNAG_00025	1299.259	0.324855	0.078509	4.137783	3.51E-05	0.00014
CNAG_00026	5765.296	-0.22484	0.057657	-3.89966	9.63E-05	0.000355
CNAG_00027	589.8108	-0.30737	0.106373	-2.88959	0.003857	0.009916
CNAG_00028	570.5793	-0.66634	0.107252	-6.21284	5.20E-10	3.91E-09
CNAG_00029	198.7606	0.448392	0.164949	2.718365	0.006561	0.015869
CNAG_00030	163.5758	-0.59647	0.181523	-3.28594	0.001016	0.002986
CNAG_07306	14.5706	1.343968	0.626473	2.145292	0.03193	0.063698
CNAG_00034	47674.08	-0.22085	0.067831	-3.25582	0.001131	0.003285
CNAG_00038	845.3812	-0.22695	0.091667	-2.47584	0.013292	0.029731
CNAG_00039	1490.853	0.225677	0.083242	2.7111	0.006706	0.016189
CNAG_07308	149.473	1.395493	0.240913	5.792511	6.93E-09	4.66E-08
CNAG_00043	1028.574	-0.49895	0.083373	-5.98453	2.17E-09	1.54E-08
CNAG_00048	57.43961	-1.01436	0.296107	-3.42567	0.000613	0.001903
CNAG_00050	391.0857	-0.23247	0.115573	-2.01143	0.04428	0.084605
CNAG_00052	277.7986	-1.21882	0.183493	-6.64228	3.09E-11	2.61E-10
CNAG_00053	704.9547	0.901339	0.108483	8.3086	9.68E-17	1.17E-15
CNAG_00054	236.5174	2.089649	0.197578	10.57633	3.84E-26	6.85E-25
CNAG_00055	980.8145	-0.67618	0.09508	-7.11172	1.15E-12	1.09E-11
CNAG_00057	3856.094	-0.71537	0.237657	-3.01009	0.002612	0.006992
CNAG_00062	4281.298	0.287662	0.060325	4.768535	1.86E-06	9.19E-06
CNAG_00063	1333.084	-0.45584	0.078897	-5.77766	7.57E-09	5.05E-08
CNAG_12011	13.68901	-1.6806	0.672175	-2.50025	0.012411	0.027966
CNAG_00068	874.0638	-1.60241	0.112267	-14.2732	3.22E-46	1.02E-44
CNAG_12013	22.37455	-1.14781	0.454163	-2.52731	0.011494	0.026169
CNAG_00070	1011.075	-0.19192	0.092241	-2.08066	0.037465	0.073166

CNAG_00072	2291.302	-0.16792	0.07304	-2.29896	0.021507	0.045112
CNAG_00073	3611.763	-0.26481	0.082931	-3.19318	0.001407	0.003999
CNAG_00074	3891.007	-0.58606	0.078982	-7.42016	1.17E-13	1.19E-12
CNAG_00075	1471.069	-0.8536	0.078917	-10.8164	2.88E-27	5.38E-26
CNAG_00076	1594.614	0.216994	0.084124	2.579457	0.009896	0.022889
CNAG_00077	3281.64	0.302706	0.083127	3.64151	0.000271	0.000905
CNAG_00078	2493.755	0.399832	0.065043	6.14724	7.88E-10	5.83E-09
CNAG_00079	441.5475	-0.50879	0.14605	-3.48364	0.000495	0.001559
CNAG_00081	2944.419	-0.2418	0.070197	-3.44464	0.000572	0.001784
CNAG_00082	1153.761	0.179384	0.081027	2.213879	0.026837	0.05489
CNAG_00083	1479.53	-0.51998	0.07628	-6.81675	9.31E-12	8.29E-11
CNAG_00088	3316.172	-0.27078	0.064833	-4.17656	2.96E-05	0.000119
CNAG_00090	1465.254	-0.29482	0.077082	-3.82475	0.000131	0.000468
CNAG_00092	3343.798	-0.48381	0.080975	-5.97476	2.30E-09	1.63E-08
CNAG_00094	393.8972	0.842823	0.171632	4.910652	9.08E-07	4.68E-06
CNAG_00096	545.6639	-0.27298	0.111937	-2.4387	0.01474	0.032477
CNAG_00099	2019.318	0.181536	0.072552	2.502163	0.012344	0.027854
CNAG_00100	7410.898	-0.16154	0.0733	-2.20381	0.027538	0.056098
CNAG_00101	1453.87	-0.36379	0.095495	-3.80948	0.000139	0.000495
CNAG_07309	2930.754	0.901329	0.080636	11.17773	5.24E-29	1.04E-27
CNAG_07310	3196.16	1.175353	0.069464	16.92037	3.18E-64	1.46E-62
CNAG_00104	9504.794	0.448211	0.063514	7.056886	1.70E-12	1.60E-11
CNAG_00106	3010.925	-0.42885	0.07538	-5.68921	1.28E-08	8.27E-08
CNAG_00107	1641.667	-0.49952	0.130431	-3.8298	0.000128	0.000459
CNAG_00108	4450.198	-0.21703	0.06636	-3.27056	0.001073	0.003129
CNAG_00109	1728.724	-0.29498	0.088245	-3.34272	0.00083	0.002493
CNAG_07312	738.4456	-0.24131	0.092161	-2.61837	0.008835	0.020731
CNAG_00115	4979.398	-0.16437	0.076921	-2.13689	0.032607	0.064815
CNAG_00117	3807.06	-0.58444	0.079504	-7.35118	1.96E-13	1.98E-12
CNAG_00118	347.8509	-0.36328	0.139292	-2.60805	0.009106	0.02133
CNAG_00121	5292.164	-0.24393	0.057187	-4.26556	1.99E-05	8.27E-05
CNAG_00123	457.9927	0.459547	0.158018	2.908199	0.003635	0.009407
CNAG_00126	3120.373	0.583712	0.104429	5.589581	2.28E-08	1.43E-07
CNAG_00127	608.9325	-0.4026	0.105065	-3.83189	0.000127	0.000456
CNAG_12019	170.7909	0.569405	0.178094	3.197226	0.001388	0.003953
CNAG_00129	976.8929	0.502262	0.093223	5.387728	7.14E-08	4.27E-07
CNAG_00131	175.9531	-0.58332	0.170915	-3.41292	0.000643	0.00198
CNAG_00135	746.4342	-0.47778	0.100058	-4.77498	1.80E-06	8.90E-06
CNAG_00137	1826.05	0.260384	0.077258	3.370332	0.000751	0.002285
CNAG_00139	2298.778	-0.50922	0.094288	-5.40064	6.64E-08	3.99E-07
CNAG_00140	4641.241	-0.20984	0.079151	-2.65113	0.008022	0.018977
CNAG_00141	386.0639	-0.58995	0.121832	-4.84233	1.28E-06	6.48E-06
CNAG_00144	371.0994	0.43494	0.121224	3.587912	0.000333	0.001096
CNAG_00146	915.9672	-0.37004	0.111283	-3.32524	0.000883	0.002634
CNAG_00150	3427.811	0.477085	0.08793	5.425736	5.77E-08	3.49E-07
CNAG_00152	911.5428	-0.20704	0.087702	-2.36076	0.018238	0.039112
CNAG_00156	1859.066	0.917971	0.085528	10.73303	7.12E-27	1.31E-25

CNAG_00158	260.0033	0.542024	0.145231	3.732155	0.00019	0.000657
CNAG_00159	463.9156	0.423706	0.137822	3.074304	0.00211	0.005774
CNAG_00161	767.7702	1.929976	0.113446	17.01227	6.66E-65	3.11E-63
CNAG_00162	17637.63	2.126017	0.075813	28.04282	4.89E-173	7.94E-171
CNAG_00163	1667.74	0.693215	0.082102	8.443372	3.08E-17	3.81E-16
CNAG_12026	45.75919	1.344526	0.320136	4.199854	2.67E-05	0.000108
CNAG_00164	603.8415	-3.60357	0.131206	-27.4649	4.61E-166	6.81E-164
CNAG_00165	3036.546	0.185414	0.068705	2.698709	0.006961	0.016714
CNAG_12028	10.83929	2.25756	0.721982	3.126892	0.001767	0.004921
CNAG_07314	954.9889	1.669225	0.100694	16.57726	1.02E-61	4.42E-60
CNAG_00171	620.691	-0.35717	0.099668	-3.58363	0.000339	0.001111
CNAG_00172	1089.929	-0.40547	0.099957	-4.05644	4.98E-05	0.000193
CNAG_00176	2787.186	0.501832	0.067567	7.427149	1.11E-13	1.13E-12
CNAG_00177	100.7193	0.556307	0.21587	2.577043	0.009965	0.02303
CNAG_00180	859.325	-0.41258	0.095984	-4.29843	1.72E-05	7.23E-05
CNAG_00181	581.0464	-0.45898	0.107208	-4.28123	1.86E-05	7.75E-05
CNAG_00185	1314.42	-0.20278	0.075441	-2.68788	0.007191	0.017225
CNAG_00188	640.2396	-0.62494	0.099962	-6.25179	4.06E-10	3.09E-09
CNAG_00189	754.5839	-0.54902	0.109968	-4.99257	5.96E-07	3.15E-06
CNAG_00190	1258.689	-0.28476	0.083217	-3.42191	0.000622	0.001928
CNAG_00191	845.1393	0.473869	0.093883	5.047436	4.48E-07	2.41E-06
CNAG_00192	1957.658	-1.85439	0.096759	-19.165	7.25E-82	4.09E-80
CNAG_00193	4466.256	-0.30939	0.062902	-4.91853	8.72E-07	4.51E-06
CNAG_07316	497.2665	-1.68548	0.118481	-14.2257	6.34E-46	1.99E-44
CNAG_07317	1386.882	-0.5073	0.105336	-4.81596	1.46E-06	7.33E-06
CNAG_07318	2644.015	-0.27764	0.074403	-3.73162	0.00019	0.000658
CNAG_07319	1603.902	-0.7575	0.136615	-5.5448	2.94E-08	1.83E-07
CNAG_07320	1138.428	-0.21628	0.080975	-2.67099	0.007563	0.018026
CNAG_07321	934.0709	0.501585	0.085362	5.87595	4.20E-09	2.88E-08
CNAG_07322	3589.606	-0.15028	0.066293	-2.26698	0.023392	0.048601
CNAG_07323	2429.867	-0.21969	0.068459	-3.2091	0.001331	0.003804
CNAG_07324	1083.838	0.402805	0.091673	4.393918	1.11E-05	4.85E-05
CNAG_07326	4631.039	0.601767	0.090055	6.682238	2.35E-11	2.00E-10
CNAG_07327	846.603	1.05406	0.115307	9.141304	6.17E-20	8.71E-19
CNAG_07331	174.1312	-0.34249	0.172054	-1.9906	0.046525	0.088169
CNAG_07334	964.8688	0.752081	0.085535	8.792702	1.46E-18	1.91E-17
CNAG_07335	244.8595	0.807044	0.144605	5.581008	2.39E-08	1.50E-07
CNAG_07336	167.5221	-0.48613	0.176039	-2.76147	0.005754	0.01416
CNAG_07029	678.3519	-0.28125	0.118427	-2.37491	0.017553	0.037824
CNAG_07028	3203.825	0.167583	0.070742	2.368921	0.01784	0.038375
CNAG_07027	1082.56	0.200654	0.089115	2.25163	0.024346	0.050335
CNAG_00231	395.7034	-0.42439	0.140146	-3.02822	0.00246	0.00663
CNAG_00232	32233.65	-0.32832	0.066346	-4.94863	7.47E-07	3.90E-06
CNAG_00234	697.0856	-0.30803	0.094752	-3.2509	0.00115	0.003336
CNAG_00235	4031.02	0.488376	0.077109	6.333606	2.39E-10	1.87E-09
CNAG_00236	778.1946	0.515922	0.092567	5.573477	2.50E-08	1.57E-07
CNAG_00237	25159.71	1.407787	0.066907	21.04088	2.77E-98	1.99E-96

CNAG_00238	18429.5	-0.25063	0.057932	-4.32622	1.52E-05	6.43E-05
CNAG_00240	1033.702	-0.26806	0.09428	-2.84325	0.004466	0.01129
CNAG_07344	446.3303	0.333974	0.123487	2.70452	0.00684	0.016454
CNAG_00249	774.9446	0.316285	0.09823	3.219841	0.001283	0.003676
CNAG_00250	1469.311	-0.73473	0.104731	-7.01536	2.29E-12	2.13E-11
CNAG_00253	823.5459	-0.38743	0.100934	-3.83845	0.000124	0.000445
CNAG_00254	895.4873	-0.33391	0.109607	-3.04648	0.002315	0.006277
CNAG_00257	3880.453	0.25097	0.068416	3.668293	0.000244	0.000825
CNAG_00260	3079.778	-0.28	0.064216	-4.36027	1.30E-05	5.58E-05
CNAG_00261	15646.08	1.492057	0.079227	18.83262	4.08E-79	2.21E-77
CNAG_00264	1559.264	-0.48914	0.125607	-3.89421	9.85E-05	0.000362
CNAG_00266	848.7154	-0.45518	0.09315	-4.88647	1.03E-06	5.26E-06
CNAG_00269	72.12152	2.219789	0.423411	5.242631	1.58E-07	9.04E-07
CNAG_12041	33.55763	2.438777	0.470824	5.17981	2.22E-07	1.24E-06
CNAG_00270	3458.129	-0.68702	0.078	-8.80796	1.27E-18	1.67E-17
CNAG_00274	539.8007	0.393719	0.106122	3.710072	0.000207	0.000711
CNAG_00275	1074.694	2.512146	0.129613	19.38189	1.10E-83	6.42E-82
CNAG_00276	462.9181	1.211699	0.118214	10.25006	1.18E-24	2.05E-23
CNAG_00278	1169.177	-0.19177	0.089195	-2.15	0.031555	0.063094
CNAG_00279	3909.761	-0.37746	0.085966	-4.39079	1.13E-05	4.90E-05
CNAG_07345	284.3202	-0.46274	0.159192	-2.90682	0.003651	0.009445
CNAG_07114	1715.285	-0.39697	0.081285	-4.88366	1.04E-06	5.33E-06
CNAG_07346	9104.222	-0.12184	0.059503	-2.04758	0.040601	0.078479
CNAG_00283	369.3849	-0.84881	0.123038	-6.8987	5.25E-12	4.73E-11
CNAG_00287	5233.665	0.143974	0.068429	2.103991	0.035379	0.069476
CNAG_00288	1282.363	0.325391	0.082531	3.942641	8.06E-05	0.000301
CNAG_00289	554.155	-0.52428	0.106714	-4.91297	8.97E-07	4.63E-06
CNAG_00290	1183.636	-0.30316	0.081154	-3.73559	0.000187	0.000649
CNAG_12042	26.50789	1.429164	0.43932	3.25313	0.001141	0.003312
CNAG_00291	1554.841	0.491664	0.077543	6.340523	2.29E-10	1.79E-09
CNAG_00293	3062.979	-0.33564	0.072021	-4.66025	3.16E-06	1.51E-05
CNAG_00294	2670.59	0.168382	0.084409	1.994835	0.046061	0.087432
CNAG_07348	507.4209	-0.5679	0.112368	-5.05391	4.33E-07	2.34E-06
CNAG_07349	1711.653	-0.50288	0.071924	-6.99179	2.71E-12	2.50E-11
CNAG_00299	1662.095	0.258891	0.077149	3.355744	0.000792	0.002389
CNAG_00301	1502.092	1.670724	0.106327	15.71312	1.23E-55	4.67E-54
CNAG_00302	244.0802	-1.21142	0.173097	-6.99852	2.59E-12	2.39E-11
CNAG_00304	567.529	-0.91174	0.114145	-7.98755	1.38E-15	1.57E-14
CNAG_12043	32.45517	-0.76572	0.370654	-2.06586	0.038842	0.07551
CNAG_00306	7319.368	-1.69562	0.103512	-16.381	2.62E-60	1.11E-58
CNAG_00307	1607.181	0.372285	0.092821	4.010798	6.05E-05	0.000231
CNAG_00308	1005.474	0.384492	0.09046	4.250426	2.13E-05	8.80E-05
CNAG_00309	1643.543	0.487359	0.080595	6.047029	1.48E-09	1.06E-08
CNAG_00312	573.1096	-0.28815	0.104424	-2.7594	0.005791	0.014224
CNAG_12046	543.8169	1.883244	0.190384	9.891795	4.52E-23	7.43E-22
CNAG_07350	586.941	-0.60177	0.117618	-5.11627	3.12E-07	1.71E-06
CNAG_00317	967.3674	-0.26886	0.10399	-2.58544	0.009725	0.02254

CNAG_00318	1677.626	-0.37539	0.075747	-4.95591	7.20E-07	3.77E-06
CNAG_00319	3166.92	-0.25125	0.082791	-3.03473	0.002408	0.006508
CNAG_07351	2129.983	0.516039	0.080709	6.393842	1.62E-10	1.28E-09
CNAG_00329	3787.338	0.352853	0.076697	4.600608	4.21E-06	1.98E-05
CNAG_00331	267.9383	1.936404	0.149836	12.92348	3.32E-38	8.59E-37
CNAG_00332	1342.748	0.306041	0.077587	3.944506	8.00E-05	0.000299
CNAG_00333	1339.826	0.219197	0.084934	2.580795	0.009857	0.022813
CNAG_00335	1606.665	-0.2146	0.072793	-2.94805	0.003198	0.008387
CNAG_00336	681.5817	-0.34635	0.099088	-3.49534	0.000473	0.0015
CNAG_00337	1287.521	-0.35218	0.084645	-4.16068	3.17E-05	0.000127
CNAG_07352	5102.126	-0.44976	0.0778	-5.78101	7.43E-09	4.96E-08
CNAG_07354	1491.087	-0.45076	0.084282	-5.34827	8.88E-08	5.24E-07
CNAG_00343	1315.124	-0.48105	0.084487	-5.69379	1.24E-08	8.07E-08
CNAG_00345	2773.018	-0.323	0.06995	-4.61752	3.88E-06	1.84E-05
CNAG_00347	1422.419	0.198119	0.075932	2.609169	0.009076	0.021272
CNAG_00349	1258.754	4.475888	0.114404	39.12353	0	0
CNAG_07356	6043.167	1.20662	0.059146	20.40072	1.65E-92	1.07E-90
CNAG_00352	996.6743	0.367653	0.095149	3.863986	0.000112	0.000405
CNAG_00353	516.0946	-0.40369	0.110154	-3.6648	0.000248	0.000836
CNAG_00354	3572.122	-0.44521	0.077608	-5.73662	9.66E-09	6.36E-08
CNAG_00355	2726.163	-0.27004	0.087658	-3.08058	0.002066	0.005662
CNAG_00358	1138.8	-0.18785	0.081711	-2.29898	0.021506	0.045112
CNAG_00359	551.6016	-0.55444	0.115855	-4.78558	1.70E-06	8.50E-06
CNAG_00360	322.9154	1.175396	0.162052	7.25322	4.07E-13	4.04E-12
CNAG_00361	2392.303	-0.1788	0.087742	-2.03783	0.041567	0.079984
CNAG_00362	1927.222	-0.29123	0.082092	-3.54759	0.000389	0.001257
CNAG_00363	3897.218	-0.37258	0.064704	-5.75829	8.50E-09	5.64E-08
CNAG_00364	1421.158	-0.24834	0.08641	-2.87396	0.004054	0.010361
CNAG_00365	1708.41	-0.40573	0.077874	-5.21004	1.89E-07	1.07E-06
CNAG_00368	1072.182	-0.17686	0.084683	-2.08849	0.036753	0.071927
CNAG_00370	36343.35	-0.18611	0.069479	-2.67865	0.007392	0.017666
CNAG_00371	248.8602	-0.35499	0.141276	-2.51271	0.011981	0.027148
CNAG_00372	2794.784	-0.42938	0.080269	-5.34925	8.83E-08	5.22E-07
CNAG_00373	625.326	-0.45444	0.100594	-4.51759	6.25E-06	2.87E-05
CNAG_00374	671.0683	0.323293	0.12207	2.648423	0.008087	0.019119
CNAG_00375	2208.617	-0.2834	0.072648	-3.90104	9.58E-05	0.000353
CNAG_12053	8.611709	-2.16937	0.802799	-2.70226	0.006887	0.016557
CNAG_00378	201.713	-1.48459	0.161083	-9.2163	3.08E-20	4.42E-19
CNAG_07950	87.31114	0.94674	0.283343	3.341318	0.000834	0.002503
CNAG_00383	2640.508	-0.26905	0.072536	-3.70924	0.000208	0.000713
CNAG_00387	491.3496	-0.265	0.112693	-2.3515	0.018698	0.039984
CNAG_00388	1067.757	-0.17334	0.086608	-2.00146	0.045343	0.086311
CNAG_00389	1497.867	0.236196	0.073516	3.212833	0.001314	0.003759
CNAG_07951	1109.648	-0.42398	0.102633	-4.13102	3.61E-05	0.000143
CNAG_00392	336.678	-0.54234	0.124787	-4.34613	1.39E-05	5.92E-05
CNAG_00393	6109.625	-0.60659	0.115138	-5.26838	1.38E-07	7.91E-07
CNAG_00395	1544.313	0.764838	0.076314	10.02224	1.22E-23	2.02E-22

CNAG_00396	848.9417	-0.29071	0.086437	-3.36319	0.00077	0.002334
CNAG_00398	1016.897	0.661659	0.092193	7.176861	7.13E-13	6.89E-12
CNAG_00399	1442.959	-0.5089	0.104401	-4.87446	1.09E-06	5.56E-06
CNAG_00400	3723.099	0.751581	0.077446	9.704548	2.88E-22	4.54E-21
CNAG_00402	6098.712	0.232356	0.070442	3.298523	0.000972	0.00287
CNAG_00406	499.022	-0.43474	0.146292	-2.97174	0.002961	0.007822
CNAG_00407	11033.21	1.027028	0.084877	12.10025	1.05E-33	2.39E-32
CNAG_00408	878.8752	-0.31274	0.093633	-3.34012	0.000837	0.002513
CNAG_00409	1792.342	0.72892	0.082149	8.873158	7.11E-19	9.46E-18
CNAG_00410	4149.686	-0.23704	0.062162	-3.81335	0.000137	0.000487
CNAG_00414	429.6272	0.650301	0.11253	5.778895	7.52E-09	5.02E-08
CNAG_00415	562.8747	-0.47074	0.108085	-4.35523	1.33E-05	5.69E-05
CNAG_00418	21632.93	-0.23418	0.05717	-4.0961	4.20E-05	0.000165
CNAG_00420	437.4556	-0.37314	0.113218	-3.29579	0.000981	0.002896
CNAG_00421	616.1406	-0.46605	0.104026	-4.48009	7.46E-06	3.37E-05
CNAG_00423	479.8652	-0.5263	0.112611	-4.67364	2.96E-06	1.42E-05
CNAG_00424	1846.882	-0.50427	0.088678	-5.68648	1.30E-08	8.39E-08
CNAG_07359	2392.586	0.170681	0.073887	2.31002	0.020887	0.043921
CNAG_00433	453.401	0.77062	0.139319	5.531354	3.18E-08	1.97E-07
CNAG_00434	1226.007	-0.42863	0.079938	-5.36197	8.23E-08	4.89E-07
CNAG_00437	261.1519	-0.61739	0.163976	-3.76511	0.000166	0.000583
CNAG_00440	416.6591	-0.41592	0.13086	-3.17837	0.001481	0.004184
CNAG_00441	18084.14	0.256071	0.055918	4.57937	4.66E-06	2.18E-05
CNAG_00442	1901.416	-0.19537	0.072413	-2.69801	0.006976	0.016745
CNAG_00443	408.0111	0.724308	0.143463	5.048739	4.45E-07	2.40E-06
CNAG_00444	1029.412	2.294119	0.10376	22.10994	2.54E-108	2.04E-106
CNAG_00445	2858.051	-0.30361	0.086072	-3.52735	0.00042	0.001348
CNAG_00447	8001.673	-0.23629	0.0566	-4.17484	2.98E-05	0.00012
CNAG_00448	3090.96	-0.33547	0.064473	-5.20326	1.96E-07	1.10E-06
CNAG_00449	2400.232	0.314718	0.078332	4.017764	5.88E-05	0.000225
CNAG_00450	5800.685	0.224862	0.06744	3.334244	0.000855	0.002563
CNAG_00451	1609.288	2.278371	0.083577	27.26075	1.24E-163	1.77E-161
CNAG_00452	68.0323	1.320508	0.2634	5.013317	5.35E-07	2.85E-06
CNAG_00453	2295.846	-0.19629	0.074305	-2.64172	0.008249	0.019478
CNAG_00455	2597.829	0.145135	0.065941	2.200998	0.027736	0.056445
CNAG_00456	4601.064	0.569186	0.082193	6.924978	4.36E-12	3.94E-11
CNAG_00457	26848.99	0.438094	0.062057	7.059516	1.67E-12	1.57E-11
CNAG_00460	315.9912	-1.09893	0.161666	-6.7975	1.06E-11	9.38E-11
CNAG_00462	12045.24	1.596675	0.066334	24.07022	5.13E-128	4.90E-126
CNAG_00464	5468.769	-0.34139	0.066018	-5.17119	2.33E-07	1.30E-06
CNAG_00465	1284.544	-0.62488	0.151443	-4.12617	3.69E-05	0.000146
CNAG_00469	1174.848	0.19307	0.084894	2.274258	0.022951	0.047782
CNAG_00473	956.9382	-0.26876	0.097792	-2.74825	0.005992	0.014645
CNAG_00474	159.3296	-0.49295	0.2173	-2.26852	0.023298	0.048433
CNAG_00475	1994.49	0.551431	0.071612	7.700286	1.36E-14	1.46E-13
CNAG_00476	46.60672	0.937106	0.361154	2.594755	0.009466	0.022042
CNAG_07361	2611.263	1.297101	0.070487	18.40205	1.26E-75	6.68E-74

CNAG_07362	12772.62	0.184521	0.056824	3.247217	0.001165	0.003375
CNAG_07363	6291.932	-0.51746	0.062879	-8.2294	1.88E-16	2.23E-15
CNAG_00484	395.2795	-0.27588	0.126903	-2.17395	0.029709	0.059875
CNAG_00486	1546.511	-0.37544	0.088015	-4.26567	1.99E-05	8.27E-05
CNAG_00490	464.4588	-0.50892	0.115411	-4.40967	1.04E-05	4.54E-05
CNAG_00493	842.9338	0.232622	0.111259	2.09082	0.036544	0.071591
CNAG_00496	828.2752	-0.20329	0.092567	-2.19619	0.028079	0.057042
CNAG_00498	805.003	0.713388	0.120729	5.909022	3.44E-09	2.40E-08
CNAG_00500	2123.466	-0.20137	0.082087	-2.45315	0.014161	0.031406
CNAG_00501	178.7423	0.492549	0.161315	3.053346	0.002263	0.006154
CNAG_00503	1502.394	1.104758	0.085466	12.92634	3.20E-38	8.30E-37
CNAG_00504	1311.587	0.3003	0.102729	2.923239	0.003464	0.009016
CNAG_00505	537.3144	0.602227	0.111005	5.425238	5.79E-08	3.50E-07
CNAG_00506	767.2382	-0.29783	0.093455	-3.18686	0.001438	0.004075
CNAG_00509	9555.016	-0.29933	0.064222	-4.66085	3.15E-06	1.51E-05
CNAG_00512	3263.203	-0.21856	0.066811	-3.27137	0.00107	0.003124
CNAG_00514	1375.026	-0.18315	0.089087	-2.0558	0.039802	0.077082
CNAG_07365	947.9411	0.615277	0.09453	6.508774	7.58E-11	6.18E-10
CNAG_00519	2632.464	-0.7969	0.073957	-10.7752	4.51E-27	8.35E-26
CNAG_07953	951.222	-0.51224	0.115333	-4.4414	8.94E-06	3.97E-05
CNAG_00520	6741.521	0.528282	0.092337	5.721258	1.06E-08	6.93E-08
CNAG_00521	1025.576	-0.28315	0.097443	-2.90584	0.003663	0.009468
CNAG_00522	2223.189	-0.55694	0.090165	-6.17692	6.54E-10	4.88E-09
CNAG_00523	878.9278	-0.50101	0.087286	-5.73988	9.47E-09	6.26E-08
CNAG_00524	671.0548	-0.401	0.101398	-3.9547	7.66E-05	0.000288
CNAG_00528	556.6911	-0.38888	0.106137	-3.66399	0.000248	0.000838
CNAG_00529	1150.863	-0.41287	0.10063	-4.10281	4.08E-05	0.000161
CNAG_00531	1786.825	-1.58196	0.115757	-13.6662	1.62E-42	4.69E-41
CNAG_12064	52.39721	1.674691	0.332478	5.037001	4.73E-07	2.54E-06
CNAG_00535	2618.95	-0.42755	0.07738	-5.52535	3.29E-08	2.04E-07
CNAG_00536	3617.03	0.728365	0.090945	8.008858	1.16E-15	1.33E-14
CNAG_00539	707.5348	1.355498	0.126597	10.70718	9.42E-27	1.72E-25
CNAG_12067	100.9061	0.795711	0.249852	3.184723	0.001449	0.004101
CNAG_00540	480.5287	5.720876	0.231848	24.67516	1.98E-134	2.06E-132
CNAG_12068	19.73429	2.709537	0.569049	4.761515	1.92E-06	9.48E-06
CNAG_00541	407.734	0.582777	0.124584	4.677798	2.90E-06	1.40E-05
CNAG_00542	392.8636	2.0925	0.141438	14.79447	1.59E-49	5.34E-48
CNAG_00543	1649.37	0.674828	0.084029	8.030874	9.68E-16	1.12E-14
CNAG_00545	1412.688	0.320925	0.077038	4.165791	3.10E-05	0.000124
CNAG_00546	1796.824	0.491266	0.094713	5.186902	2.14E-07	1.20E-06
CNAG_00547	3036.527	-0.29194	0.069608	-4.19404	2.74E-05	0.000111
CNAG_07955	254.2929	-1.30832	0.145917	-8.96621	3.07E-19	4.19E-18
CNAG_00550	650.1725	0.853608	0.102296	8.344461	7.15E-17	8.69E-16
CNAG_00551	5701.426	-0.23915	0.059042	-4.05044	5.11E-05	0.000197
CNAG_12069	88.87125	0.591586	0.241429	2.450354	0.014272	0.031625
CNAG_07367	807.0699	5.620102	0.170011	33.05725	1.22E-239	3.43E-237
CNAG_12070	79.01648	3.958264	0.344518	11.48929	1.49E-30	3.12E-29

CNAG_12071	11.83123	5.212269	1.178658	4.422206	9.77E-06	4.31E-05
CNAG_07368	1984.932	-0.15734	0.069318	-2.2699	0.023214	0.048281
CNAG_00556	2829.099	-0.30046	0.081279	-3.69667	0.000218	0.000745
CNAG_00557	1654.748	-0.35514	0.09407	-3.77526	0.00016	0.000563
CNAG_00558	2017.596	-0.19497	0.070958	-2.74772	0.006001	0.014661
CNAG_00560	4060.827	-0.20211	0.068345	-2.95725	0.003104	0.008167
CNAG_00564	1095.405	0.798824	0.088536	9.022594	1.84E-19	2.53E-18
CNAG_00565	6926.407	0.151823	0.062786	2.418095	0.015602	0.034117
CNAG_00566	1041.003	-0.56823	0.085552	-6.64192	3.10E-11	2.61E-10
CNAG_00567	1710.448	-0.28577	0.071724	-3.98432	6.77E-05	0.000256
CNAG_00570	3442.716	-0.36667	0.066428	-5.51983	3.39E-08	2.10E-07
CNAG_00572	1606.34	0.288404	0.086221	3.344949	0.000823	0.002474
CNAG_00573	3521.879	1.053018	0.079502	13.24524	4.81E-40	1.32E-38
CNAG_00575	2056.568	1.083216	0.374782	2.890257	0.003849	0.009898
CNAG_00576	1407.426	0.231697	0.077573	2.98682	0.002819	0.0075
CNAG_00577	703.8161	-0.26515	0.093812	-2.82644	0.004707	0.011841
CNAG_00580	4271.003	0.197032	0.063958	3.080649	0.002065	0.005662
CNAG_00581	5809.919	-0.35874	0.079738	-4.49897	6.83E-06	3.11E-05
CNAG_00582	2176.39	0.166453	0.084815	1.96255	0.049699	0.093183
CNAG_00583	4517.357	0.396479	0.06711	5.907867	3.47E-09	2.41E-08
CNAG_00586	431.465	2.36855	0.177639	13.33349	1.48E-40	4.09E-39
CNAG_00587	525.1943	3.861883	0.174071	22.18566	4.72E-109	3.88E-107
CNAG_00588	1120.537	3.16049	0.283442	11.15038	7.13E-29	1.41E-27
CNAG_12078	142.6597	1.209842	0.232599	5.201417	1.98E-07	1.11E-06
CNAG_00589	1153.75	-0.20436	0.090358	-2.26164	0.02372	0.049196
CNAG_12079	288.1586	0.382701	0.177704	2.153584	0.031273	0.062607
CNAG_00592	768.0805	0.958529	0.097574	9.823603	8.91E-23	1.43E-21
CNAG_00593	883.986	0.690598	0.092561	7.460992	8.59E-14	8.85E-13
CNAG_00594	3393.397	0.185863	0.067376	2.75858	0.005805	0.014251
CNAG_00595	512.6608	0.650603	0.157446	4.132226	3.59E-05	0.000143
CNAG_00596	240.8583	1.206579	0.161571	7.467802	8.15E-14	8.43E-13
CNAG_12080	15.98176	3.714935	0.736149	5.046447	4.50E-07	2.42E-06
CNAG_00597	538.8334	1.047606	0.160391	6.531569	6.51E-11	5.34E-10
CNAG_12081	51.065	1.154155	0.347133	3.32482	0.000885	0.002637
CNAG_00600	2970.695	-0.19585	0.076012	-2.57661	0.009977	0.023052
CNAG_00602	5220.697	-0.25783	0.071891	-3.58633	0.000335	0.001101
CNAG_00604	536.7545	0.284359	0.118232	2.405094	0.016168	0.035204
CNAG_07370	131.6267	0.530891	0.245586	2.161733	0.030639	0.061444
CNAG_00613	1227.129	-0.35111	0.078865	-4.45207	8.50E-06	3.80E-05
CNAG_07373	17586.65	0.236169	0.065501	3.605596	0.000311	0.001029
CNAG_07374	1110.991	-0.19984	0.087435	-2.28556	0.02228	0.046565
CNAG_00624	1469.859	-0.42631	0.0744	-5.72993	1.00E-08	6.61E-08
CNAG_00626	9998.39	0.449575	0.054748	8.211748	2.18E-16	2.58E-15
CNAG_00627	206.6885	1.261289	0.227886	5.534742	3.12E-08	1.94E-07
CNAG_00635	3594.882	0.210438	0.067277	3.127945	0.00176	0.004906
CNAG_00638	1895.893	-0.31832	0.119929	-2.65423	0.007949	0.018836
CNAG_00639	796.327	0.545799	0.104802	5.207884	1.91E-07	1.08E-06

CNAG_00640	92132.66	-0.24455	0.059991	-4.07648	4.57E-05	0.000178
CNAG_00641	4573.781	-0.24219	0.071744	-3.37578	0.000736	0.002246
CNAG_00642	795.1184	0.449268	0.088475	5.077921	3.82E-07	2.08E-06
CNAG_07959	1758.173	-0.18232	0.076173	-2.39355	0.016686	0.036225
CNAG_00648	881.5939	-0.33318	0.131995	-2.52421	0.011596	0.026357
CNAG_00649	8376.147	-0.12711	0.063953	-1.98755	0.046861	0.088723
CNAG_00651	318.8435	0.728593	0.13658	5.334556	9.58E-08	5.63E-07
CNAG_00652	751.2915	0.798641	0.10178	7.846744	4.27E-15	4.74E-14
CNAG_00653	229.3201	1.560594	0.187013	8.344823	7.13E-17	8.68E-16
CNAG_00654	3918.735	3.354689	0.097075	34.55788	1.09E-261	3.84E-259
CNAG_00656	62188.68	-0.20626	0.062302	-3.31068	0.000931	0.002761
CNAG_00658	493.9736	-0.32211	0.106818	-3.0155	0.002566	0.006885
CNAG_00659	374.3972	-0.42464	0.123868	-3.42819	0.000608	0.001887
CNAG_00660	700.7039	-0.23387	0.102973	-2.27116	0.023137	0.048134
CNAG_00662	2817.699	1.058867	0.077383	13.6834	1.28E-42	3.76E-41
CNAG_00663	1938.013	1.778469	0.082247	21.62352	1.08E-103	8.35E-102
CNAG_00664	109.1452	2.127225	0.237373	8.961513	3.20E-19	4.36E-18
CNAG_00665	3985.046	-0.15746	0.065743	-2.3951	0.016616	0.036101
CNAG_00668	3422.753	0.913773	0.066953	13.64788	2.08E-42	5.99E-41
CNAG_00669	915.8334	-0.24692	0.09009	-2.74086	0.006128	0.014935
CNAG_00671	1775.205	0.141441	0.07042	2.008535	0.044586	0.08509
CNAG_00672	49158.09	-0.26517	0.072524	-3.65635	0.000256	0.000859
CNAG_00673	2287.699	-0.31734	0.070746	-4.48564	7.27E-06	3.29E-05
CNAG_00674	1407.117	-0.28918	0.116555	-2.48106	0.013099	0.029371
CNAG_00676	804.8599	-0.19203	0.087538	-2.19369	0.028258	0.057363
CNAG_00678	5737.769	0.842179	0.062926	13.38362	7.54E-41	2.11E-39
CNAG_00680	1489.114	-0.47759	0.085066	-5.61443	1.97E-08	1.25E-07
CNAG_00681	1634.956	-0.33319	0.073794	-4.51511	6.33E-06	2.90E-05
CNAG_12088	13.40043	-1.14244	0.570544	-2.00237	0.045245	0.086164
CNAG_12090	2.944063	3.077477	1.47645	2.084377	0.037126	0.072556
CNAG_00683	1353.908	-0.41942	0.105963	-3.95823	7.55E-05	0.000284
CNAG_00684	3457.075	-0.43169	0.065284	-6.61251	3.78E-11	3.17E-10
CNAG_00686	11858.96	-0.24993	0.071683	-3.48656	0.000489	0.001546
CNAG_00691	850.8162	0.329131	0.089124	3.692951	0.000222	0.000755
CNAG_00692	466.6789	0.418344	0.114928	3.640055	0.000273	0.000909
CNAG_00694	6509.986	-0.1437	0.060249	-2.38512	0.017074	0.036898
CNAG_00696	706.6754	0.213979	0.095036	2.251558	0.02435	0.050335
CNAG_00698	1719.67	-0.41541	0.078767	-5.27385	1.34E-07	7.69E-07
CNAG_00699	230.3554	0.863581	0.165773	5.209411	1.89E-07	1.07E-06
CNAG_12092	237.1898	0.523011	0.171495	3.049717	0.002291	0.006216
CNAG_00700	14045.61	0.189211	0.054317	3.483477	0.000495	0.001559
CNAG_00701	2493.789	-0.18524	0.077478	-2.39084	0.01681	0.036435
CNAG_12093	255.4261	0.332856	0.140648	2.366597	0.017952	0.038603
CNAG_00702	3850.365	-0.23764	0.080702	-2.94472	0.003232	0.008459
CNAG_00703	43403.11	-0.15963	0.070562	-2.26227	0.02368	0.049138
CNAG_00705	4113.844	-0.1472	0.065403	-2.25075	0.024402	0.050429
CNAG_00707	2436.795	-0.4046	0.070509	-5.73822	9.57E-09	6.31E-08

CNAG_00708	2209.699	-0.29493	0.078744	-3.74544	0.00018	0.000626
CNAG_00711	370.8835	-0.36197	0.121905	-2.96926	0.002985	0.007878
CNAG_00713	1860.908	-0.22706	0.07413	-3.06294	0.002192	0.005972
CNAG_00715	2601.093	1.105202	0.094148	11.73901	8.04E-32	1.72E-30
CNAG_00716	34602.19	1.10056	0.091643	12.00925	3.18E-33	7.08E-32
CNAG_00720	1654.148	0.252958	0.079132	3.196679	0.00139	0.003958
CNAG_00721	2910.399	-0.40444	0.070876	-5.7063	1.15E-08	7.52E-08
CNAG_07381	1651.419	-0.26477	0.082924	-3.1929	0.001409	0.004002
CNAG_12098	12.1312	1.580763	0.659217	2.39794	0.016488	0.035851
CNAG_12099	21.31527	-1.15523	0.460985	-2.50599	0.012211	0.027585
CNAG_00733	1940.858	-0.23626	0.073641	-3.20822	0.001336	0.003813
CNAG_00735	479.7023	3.659307	0.150768	24.27116	3.95E-130	3.92E-128
CNAG_12101	81.52035	0.506475	0.247644	2.045168	0.040838	0.078825
CNAG_00738	86.02155	-1.15382	0.254912	-4.52635	6.00E-06	2.76E-05
CNAG_00739	1955.817	-0.26103	0.07635	-3.41888	0.000629	0.001947
CNAG_00741	12499.69	-0.33332	0.060077	-5.54825	2.89E-08	1.80E-07
CNAG_00744	1715.956	0.54808	0.082436	6.648527	2.96E-11	2.50E-10
CNAG_00745	2684.223	0.501162	0.095031	5.273672	1.34E-07	7.69E-07
CNAG_00747	8330.659	-0.30418	0.061699	-4.93008	8.22E-07	4.27E-06
CNAG_00748	387.7121	-0.43733	0.124605	-3.5097	0.000449	0.001429
CNAG_12105	24.60691	-1.10065	0.431517	-2.55066	0.010752	0.02468
CNAG_00750	1096.594	-0.58825	0.114441	-5.14026	2.74E-07	1.52E-06
CNAG_00752	470.0058	-0.26455	0.112002	-2.36203	0.018175	0.038989
CNAG_00753	1238.57	0.26173	0.080684	3.243881	0.001179	0.003409
CNAG_00754	9760.054	0.970532	0.06123	15.8506	1.39E-56	5.44E-55
CNAG_00755	778.4898	0.490548	0.111319	4.406673	1.05E-05	4.60E-05
CNAG_00756	488.346	-0.42021	0.124409	-3.3777	0.000731	0.002231
CNAG_00757	1436.674	-0.19593	0.087588	-2.2369	0.025293	0.052006
CNAG_00758	1072.687	1.183083	0.097892	12.08554	1.26E-33	2.84E-32
CNAG_00760	1940.311	-0.26651	0.070439	-3.78355	0.000155	0.000545
CNAG_00761	1617.719	-0.26561	0.080696	-3.29149	0.000997	0.002934
CNAG_00765	922.0556	1.018724	0.107352	9.489566	2.32E-21	3.52E-20
CNAG_00766	501.8959	-0.52226	0.117373	-4.44962	8.60E-06	3.84E-05
CNAG_12111	22.94239	-1.2386	0.454708	-2.72395	0.006451	0.015637
CNAG_00769	1501.102	0.21029	0.098039	2.144957	0.031956	0.063724
CNAG_00770	1562.674	0.317034	0.074244	4.270164	1.95E-05	8.11E-05
CNAG_00774	6657.061	-0.35278	0.057227	-6.16448	7.07E-10	5.26E-09
CNAG_12113	121.2239	2.115956	0.220389	9.600999	7.92E-22	1.22E-20
CNAG_00776	20997.53	0.909812	0.056327	16.15235	1.09E-58	4.49E-57
CNAG_00779	42416.85	-0.17125	0.070637	-2.42431	0.015337	0.033629
CNAG_00781	1163.475	-0.61892	0.113492	-5.4534	4.94E-08	3.00E-07
CNAG_00782	902.4532	0.614726	0.095728	6.421561	1.35E-10	1.08E-09
CNAG_00783	1017.238	-0.22656	0.089611	-2.52829	0.011462	0.026111
CNAG_00787	659.3878	-0.37082	0.100204	-3.70071	0.000215	0.000735
CNAG_12114	242.0139	-0.85845	0.176004	-4.87745	1.07E-06	5.48E-06
CNAG_00790	724.4073	-0.40107	0.091791	-4.36938	1.25E-05	5.37E-05
CNAG_00791	1950.014	0.751065	0.092938	8.081332	6.41E-16	7.43E-15

CNAG_00793	2025.447	-0.23228	0.073311	-3.16837	0.001533	0.004322
CNAG_12117	147.4699	0.432548	0.187951	2.301383	0.02137	0.044848
CNAG_00796	2891.956	1.671698	0.086372	19.35457	1.87E-83	1.08E-81
CNAG_00797	9102.64	0.324898	0.064642	5.026137	5.00E-07	2.68E-06
CNAG_07961	142.8089	1.177914	0.194208	6.065211	1.32E-09	9.53E-09
CNAG_00798	81.59156	0.860242	0.287016	2.997188	0.002725	0.007271
CNAG_00799	11953.73	0.258372	0.07551	3.421704	0.000622	0.001929
CNAG_00800	1484.556	3.253029	0.13934	23.34606	1.51E-120	1.34E-118
CNAG_00803	1308.066	0.296045	0.084146	3.518242	0.000434	0.00139
CNAG_00805	480.1864	-0.3838	0.130422	-2.94278	0.003253	0.008507
CNAG_00806	7138.843	-0.21088	0.104017	-2.02741	0.04262	0.08178
CNAG_00807	746.4357	-0.29438	0.106823	-2.75572	0.005856	0.014351
CNAG_00810	1275.618	-0.47413	0.081283	-5.83303	5.44E-09	3.71E-08
CNAG_00811	2443.384	-0.17798	0.076327	-2.33176	0.019713	0.041857
CNAG_00814	120.8409	1.00115	0.201447	4.9698	6.70E-07	3.52E-06
CNAG_00815	2837.788	1.557054	0.075641	20.58485	3.75E-94	2.52E-92
CNAG_00816	2174.345	-0.22185	0.092127	-2.40812	0.016035	0.034941
CNAG_00818	789.0289	0.454782	0.122899	3.700443	0.000215	0.000735
CNAG_00820	908.397	-0.98866	0.092879	-10.6446	1.85E-26	3.31E-25
CNAG_00821	26496.93	-0.19886	0.074941	-2.65363	0.007963	0.018864
CNAG_00822	5849.895	0.1776	0.061101	2.906647	0.003653	0.009448
CNAG_00823	856.5344	0.291421	0.093967	3.101302	0.001927	0.005334
CNAG_00828	314.0889	0.387563	0.143607	2.698776	0.006959	0.016714
CNAG_00829	2666.345	0.137806	0.069851	1.972867	0.048511	0.091335
CNAG_00830	714.3475	0.415494	0.099034	4.195464	2.72E-05	0.00011
CNAG_00831	1086.809	0.194898	0.082499	2.362445	0.018155	0.038966
CNAG_00832	353.3065	-0.2776	0.129825	-2.1383	0.032493	0.064635
CNAG_00834	823.6927	0.392122	0.156072	2.512448	0.01199	0.027161
CNAG_00836	987.3982	1.251948	0.095976	13.04439	6.84E-39	1.81E-37
CNAG_00837	874.6321	0.552041	0.098099	5.627367	1.83E-08	1.17E-07
CNAG_00838	965.5996	2.952668	0.118535	24.90957	5.86E-137	6.35E-135
CNAG_00839	289.4732	1.445234	0.149558	9.663397	4.31E-22	6.70E-21
CNAG_00840	405.6491	3.643071	0.153544	23.72651	1.92E-124	1.72E-122
CNAG_00841	516.7955	0.420917	0.134466	3.130273	0.001746	0.004871
CNAG_07386	335.0616	1.660035	0.133986	12.38963	2.97E-35	7.07E-34
CNAG_12123	12.22564	3.59251	0.800139	4.489857	7.13E-06	3.24E-05
CNAG_00843	917.2593	2.797725	0.123264	22.69698	4.80E-114	4.06E-112
CNAG_00844	459.5852	5.976354	0.244845	24.40874	1.38E-131	1.39E-129
CNAG_00845	2754.43	0.361189	0.076469	4.72332	2.32E-06	1.13E-05
CNAG_00847	651.0425	-0.23164	0.097405	-2.37815	0.0174	0.037513
CNAG_00848	3073.122	1.091748	0.295015	3.700659	0.000215	0.000735
CNAG_00849	162.1783	1.190115	0.204699	5.813968	6.10E-09	4.14E-08
CNAG_00851	109.0244	-0.54518	0.232403	-2.34582	0.018986	0.040512
CNAG_00852	1957.968	-0.19241	0.069652	-2.76244	0.005737	0.014126
CNAG_00854	2975.653	-0.86957	0.096531	-9.00822	2.09E-19	2.88E-18
CNAG_07387	1107.006	2.805631	0.12965	21.63999	7.55E-104	5.90E-102
CNAG_12126	23.94457	3.196999	0.534048	5.986355	2.15E-09	1.52E-08

CNAG_07388	11.62635	5.183479	1.166816	4.442412	8.90E-06	3.96E-05
CNAG_07389	879.4098	0.688906	0.097775	7.045801	1.84E-12	1.73E-11
CNAG_07391	129.265	1.223187	0.200349	6.105278	1.03E-09	7.50E-09
CNAG_07392	558.0798	0.996276	0.111606	8.926704	4.39E-19	5.95E-18
CNAG_00858	467.0977	0.444341	0.130464	3.405848	0.00066	0.002026
CNAG_00859	198.2663	0.405048	0.165677	2.444798	0.014493	0.032037
CNAG_07393	136.6967	-0.96661	0.212889	-4.54045	5.61E-06	2.60E-05
CNAG_07459	197.54	-0.38135	0.153704	-2.48107	0.013099	0.029371
CNAG_06801	872.6173	-0.27905	0.10263	-2.71896	0.006549	0.015851
CNAG_06799	3577.029	-0.33855	0.065527	-5.16659	2.38E-07	1.33E-06
CNAG_06796	1242.735	-0.20047	0.082458	-2.43115	0.015051	0.033064
CNAG_06795	3204.851	0.542488	0.077429	7.006237	2.45E-12	2.26E-11
CNAG_06794	1587.141	-0.43191	0.090686	-4.76274	1.91E-06	9.42E-06
CNAG_06793	1357.297	0.259722	0.087415	2.971134	0.002967	0.007832
CNAG_06784	153.6575	0.626513	0.184862	3.38909	0.000701	0.002146
CNAG_06782	1955.887	0.4502	0.086525	5.203092	1.96E-07	1.10E-06
CNAG_06781	902.4883	-0.38094	0.10033	-3.79689	0.000147	0.000519
CNAG_06779	2728.669	-0.39406	0.076243	-5.16845	2.36E-07	1.32E-06
CNAG_06777	371.2748	0.955134	0.132733	7.195904	6.20E-13	6.02E-12
CNAG_06776	173.5202	0.436998	0.177622	2.460273	0.013883	0.030873
CNAG_06775	2961.941	-0.12791	0.064027	-1.99768	0.045751	0.086946
CNAG_06774	1391.57	-0.19033	0.087652	-2.17143	0.029899	0.060197
CNAG_06772	1115.676	-0.44393	0.105492	-4.20816	2.57E-05	0.000104
CNAG_06770	27216.81	-0.24353	0.054034	-4.50698	6.58E-06	3.01E-05
CNAG_06767	11377.96	-0.39032	0.068847	-5.66936	1.43E-08	9.24E-08
CNAG_06766	3559.146	0.222028	0.072896	3.045817	0.00232	0.006289
CNAG_07962	93.10753	-1.82962	0.233586	-7.83277	4.77E-15	5.28E-14
CNAG_06765	1641.414	-0.34406	0.0872	-3.94566	7.96E-05	0.000298
CNAG_06764	1795.193	-1.5719	0.088919	-17.6778	6.22E-70	3.08E-68
CNAG_06763	1052.403	-0.36391	0.088881	-4.09432	4.23E-05	0.000166
CNAG_12134	225.3926	0.395066	0.162212	2.435487	0.014872	0.032749
CNAG_06762	1982.759	-1.60539	0.072607	-22.1107	2.49E-108	2.03E-106
CNAG_06761	655.872	2.078238	0.113174	18.36318	2.59E-75	1.36E-73
CNAG_06760	964.465	-1.07951	0.095117	-11.3494	7.47E-30	1.54E-28
CNAG_06758	1666.669	1.470499	0.238849	6.156598	7.43E-10	5.52E-09
CNAG_12136	20.14768	2.566369	0.584697	4.389227	1.14E-05	4.94E-05
CNAG_06755	6664.28	-0.22206	0.05942	-3.7371	0.000186	0.000645
CNAG_06753	5491.506	-0.19555	0.063299	-3.08925	0.002007	0.005527
CNAG_06752	1098.275	-0.36924	0.087173	-4.23566	2.28E-05	9.31E-05
CNAG_06751	3478.083	0.704919	0.074727	9.433301	3.97E-21	5.99E-20
CNAG_06750	697.0571	-0.79742	0.09846	-8.09886	5.55E-16	6.46E-15
CNAG_06749	956.7191	0.350504	0.110356	3.176116	0.001493	0.004212
CNAG_06747	27192.95	-1.12491	0.093629	-12.0146	2.98E-33	6.65E-32
CNAG_06746	27756.83	-1.0379	0.095253	-10.8963	1.20E-27	2.28E-26
CNAG_06745	24402.53	-1.22666	0.099647	-12.31	8.00E-35	1.87E-33
CNAG_06743	541.78	3.225349	0.135743	23.76064	8.53E-125	7.79E-123
CNAG_06742	3302.986	0.1996	0.068324	2.921387	0.003485	0.009064

CNAG_06741	3331.393	0.185594	0.06226	2.98096	0.002873	0.007618
CNAG_06738	1916.903	-0.25881	0.082414	-3.14034	0.001688	0.004723
CNAG_06737	1418.929	-0.25706	0.093735	-2.74241	0.006099	0.014878
CNAG_07463	1511.178	0.227818	0.077006	2.958451	0.003092	0.00814
CNAG_06730	4700.192	-0.3619	0.073714	-4.90958	9.13E-07	4.70E-06
CNAG_06729	858.9593	0.695432	0.107455	6.471849	9.68E-11	7.84E-10
CNAG_06728	2540.669	0.250019	0.070427	3.550031	0.000385	0.001247
CNAG_06727	334.5539	1.190843	0.150591	7.907821	2.62E-15	2.94E-14
CNAG_06723	8391.441	1.283334	0.063135	20.32681	7.45E-92	4.77E-90
CNAG_06722	93.49608	1.289657	0.227525	5.668205	1.44E-08	9.29E-08
CNAG_07464	3169.43	-0.15565	0.063207	-2.46259	0.013794	0.030717
CNAG_07465	850.4193	-0.19188	0.091882	-2.08831	0.03677	0.071929
CNAG_06717	2383.106	0.404548	0.075816	5.335951	9.50E-08	5.60E-07
CNAG_06713	3425.277	-0.24598	0.074502	-3.3017	0.000961	0.002841
CNAG_06712	1025.771	-0.24305	0.092187	-2.63652	0.008376	0.019739
CNAG_06711	694.071	-0.43576	0.115945	-3.75838	0.000171	0.000598
CNAG_06710	1719.24	-0.41854	0.076068	-5.50215	3.75E-08	2.31E-07
CNAG_06703	966.7072	0.40325	0.087289	4.619714	3.84E-06	1.82E-05
CNAG_07466	885.6219	-1.01962	0.4393	-2.32102	0.020286	0.042905
CNAG_03595	3212.028	0.364795	0.080161	4.550784	5.34E-06	2.48E-05
CNAG_03596	7943.144	0.194646	0.058785	3.311147	0.000929	0.002757
CNAG_03598	1938.487	-0.23201	0.06878	-3.37321	0.000743	0.002265
CNAG_07964	1873.408	0.417851	0.08221	5.082737	3.72E-07	2.03E-06
CNAG_03602	12506.82	0.128906	0.053936	2.389952	0.016851	0.036513
CNAG_03603	2397.253	-0.19508	0.070821	-2.75452	0.005878	0.014394
CNAG_03604	1161.112	0.323634	0.083496	3.876025	0.000106	0.000387
CNAG_03606	11359.81	0.28333	0.056491	5.015505	5.29E-07	2.83E-06
CNAG_03607	249.1935	-0.31707	0.141945	-2.23378	0.025497	0.0524
CNAG_03608	153.9924	-0.36499	0.182138	-2.0039	0.045081	0.085893
CNAG_03609	566.5416	-0.51103	0.106998	-4.77605	1.79E-06	8.87E-06
CNAG_12147	58.52828	-0.76825	0.297258	-2.58446	0.009753	0.022591
CNAG_03612	7176.922	-0.27954	0.067217	-4.15873	3.20E-05	0.000128
CNAG_03618	356.3774	-0.66752	0.131457	-5.07787	3.82E-07	2.08E-06
CNAG_03620	1580.937	0.161642	0.074968	2.156143	0.031072	0.062252
CNAG_03621	15393.83	0.257472	0.072832	3.535164	0.000408	0.001311
CNAG_03623	1873.74	1.426811	0.079089	18.04057	9.36E-73	4.75E-71
CNAG_03625	705.9968	-0.24306	0.109974	-2.21015	0.027094	0.055352
CNAG_03629	15899.7	0.852129	0.056176	15.16897	5.68E-52	2.00E-50
CNAG_03630	240.9745	1.050665	0.182505	5.756926	8.57E-09	5.67E-08
CNAG_03631	961.7198	-0.35106	0.096271	-3.64659	0.000266	0.000889
CNAG_03633	610.183	-0.29891	0.105146	-2.84277	0.004472	0.011303
CNAG_03636	1385.833	-0.21145	0.088407	-2.39173	0.016769	0.036375
CNAG_03638	1793.506	-0.2601	0.075835	-3.42974	0.000604	0.001877
CNAG_03639	254.9577	0.377027	0.15636	2.411274	0.015897	0.034687
CNAG_03642	323.1557	0.348715	0.13153	2.651228	0.00802	0.018977
CNAG_03645	11574.81	0.230159	0.059421	3.87337	0.000107	0.000391
CNAG_03646	1295.609	-0.17495	0.082419	-2.12271	0.033778	0.066816

CNAG_03648	3454.955	0.209726	0.068617	3.056469	0.00224	0.006092
CNAG_03649	1266.062	0.538624	0.089124	6.043566	1.51E-09	1.08E-08
CNAG_03652	681.6938	0.319858	0.111665	2.864443	0.004177	0.010654
CNAG_03654	1260.551	0.393508	0.079723	4.935913	7.98E-07	4.15E-06
CNAG_03655	3554.727	-0.36524	0.084218	-4.33687	1.45E-05	6.16E-05
CNAG_03660	1194.115	-0.38286	0.088481	-4.32702	1.51E-05	6.41E-05
CNAG_03663	4153.187	2.468461	0.087695	28.14832	2.51E-174	4.26E-172
CNAG_03664	3701.265	1.155786	0.066966	17.25934	9.52E-67	4.58E-65
CNAG_03665	2480.444	0.203102	0.068351	2.971442	0.002964	0.007827
CNAG_03666	952.6017	2.217248	0.109325	20.28122	1.88E-91	1.20E-89
CNAG_03667	2620.478	0.366538	0.113417	3.231779	0.00123	0.003539
CNAG_03670	1123.251	-0.28661	0.109938	-2.60699	0.009134	0.021384
CNAG_03671	370.3873	0.381344	0.123194	3.095483	0.001965	0.005429
CNAG_03672	866.2158	0.656904	0.096831	6.784041	1.17E-11	1.02E-10
CNAG_03674	4869.026	0.315686	0.068147	4.632421	3.61E-06	1.72E-05
CNAG_03675	5650.592	0.260979	0.067094	3.889736	0.0001	0.000368
CNAG_03676	833.3323	-0.23125	0.087549	-2.64137	0.008257	0.019493
CNAG_03677	3721.719	-0.63213	0.077003	-8.20909	2.23E-16	2.63E-15
CNAG_03679	294.183	-0.30488	0.133855	-2.27768	0.022746	0.047417
CNAG_03680	716.478	0.259784	0.099906	2.600292	0.009314	0.021755
CNAG_03681	3137.241	-0.14421	0.067216	-2.14551	0.031912	0.063698
CNAG_03682	13108.78	-0.52559	0.066444	-7.91018	2.57E-15	2.89E-14
CNAG_03683	814.7479	0.678645	0.124648	5.444497	5.20E-08	3.15E-07
CNAG_03684	4777.389	0.172277	0.061814	2.787009	0.00532	0.013219
CNAG_03685	420.4969	-0.57291	0.130701	-4.38336	1.17E-05	5.06E-05
CNAG_03688	1748.529	-0.29229	0.112725	-2.59296	0.009515	0.022109
CNAG_03694	1424.645	0.242022	0.082253	2.942395	0.003257	0.008512
CNAG_03695	721.7648	0.974998	0.097463	10.00382	1.47E-23	2.42E-22
CNAG_03696	278.54	1.821443	0.144698	12.58792	2.46E-36	6.08E-35
CNAG_03697	320.5701	0.296914	0.141509	2.098207	0.035887	0.070388
CNAG_03699	3992.71	0.1725	0.062339	2.767123	0.005655	0.013942
CNAG_03700	1155.751	-0.2106	0.081753	-2.57607	0.009993	0.023081
CNAG_03701	6506.444	-0.17495	0.058512	-2.98992	0.002791	0.007427
CNAG_03705	1928.788	-1.28127	0.085036	-15.0675	2.65E-51	9.13E-50
CNAG_03706	8087.18	-0.26773	0.055993	-4.78158	1.74E-06	8.65E-06
CNAG_03707	977.7683	0.471108	0.084014	5.607486	2.05E-08	1.30E-07
CNAG_03708	1723.487	-0.26365	0.075514	-3.49137	0.000481	0.00152
CNAG_03710	2034.168	0.19899	0.072064	2.761298	0.005757	0.014161
CNAG_12158	3.518367	2.724256	1.345643	2.024501	0.042919	0.082274
CNAG_03712	378.6808	-0.37607	0.131012	-2.87047	0.004099	0.010473
CNAG_03713	217.9844	-1.319	0.639149	-2.06368	0.039048	0.075839
CNAG_03715	1514.538	-0.22616	0.110959	-2.03822	0.041528	0.079928
CNAG_03716	3324.743	-0.68474	0.079042	-8.66302	4.59E-18	5.89E-17
CNAG_03718	1432.943	0.261847	0.094167	2.780672	0.005425	0.013455
CNAG_03719	1471.948	-0.25048	0.109517	-2.28713	0.022188	0.046422
CNAG_03721	2693.97	0.184436	0.065344	2.82255	0.004764	0.011959
CNAG_03725	6077.278	-0.43639	0.062337	-7.00039	2.55E-12	2.36E-11

CNAG_03726	3096.019	-0.61052	0.079714	-7.65891	1.88E-14	2.01E-13
CNAG_12161	180.603	0.760389	0.177427	4.285638	1.82E-05	7.61E-05
CNAG_03727	1735.33	0.232787	0.092314	2.52168	0.01168	0.02654
CNAG_03728	787.6637	-0.63439	0.128994	-4.91796	8.74E-07	4.52E-06
CNAG_03731	890.9305	-0.3354	0.092419	-3.62906	0.000284	0.000948
CNAG_03733	296.0824	2.203602	0.162263	13.58043	5.23E-42	1.48E-40
CNAG_03734	284.3304	0.766544	0.134084	5.71688	1.08E-08	7.09E-08
CNAG_03735	1223.394	-0.35057	0.101009	-3.47063	0.000519	0.00163
CNAG_07476	281.9448	1.867598	0.148897	12.54291	4.35E-36	1.06E-34
CNAG_03742	4463.661	0.22873	0.062538	3.65743	0.000255	0.000857
CNAG_03743	602.131	0.44849	0.10532	4.25834	2.06E-05	8.52E-05
CNAG_03746	921.3316	4.69875	0.133483	35.20122	1.92E-271	7.78E-269
CNAG_07477	968.3183	0.670911	0.11011	6.09311	1.11E-09	8.07E-09
CNAG_03752	327.2693	-0.361	0.154703	-2.33354	0.01962	0.041713
CNAG_03755	963.0318	1.407556	0.112738	12.48521	8.99E-36	2.17E-34
CNAG_07479	300.4344	0.522985	0.135376	3.863219	0.000112	0.000405
CNAG_07480	38.27334	0.664935	0.336486	1.976119	0.048141	0.090787
CNAG_03760	1785.252	-0.15283	0.077336	-1.97614	0.048139	0.090787
CNAG_03761	3414.181	-0.15359	0.065535	-2.34357	0.0191	0.040704
CNAG_03763	3739.615	0.161739	0.078331	2.064811	0.038941	0.075666
CNAG_03764	129.8356	1.830447	0.22045	8.303223	1.01E-16	1.22E-15
CNAG_12165	7.449947	1.782489	0.809142	2.202938	0.027599	0.056194
CNAG_03765	6633.157	-0.26239	0.080998	-3.23952	0.001197	0.003453
CNAG_03766	1326.312	0.320227	0.078747	4.066534	4.77E-05	0.000185
CNAG_12166	82.35345	0.46482	0.235653	1.972477	0.048555	0.091395
CNAG_12168	51.7309	-0.88837	0.326547	-2.72048	0.006519	0.015788
CNAG_03767	2468.69	0.19707	0.070368	2.800558	0.005101	0.012731
CNAG_03768	1302.302	0.641693	0.103043	6.227443	4.74E-10	3.58E-09
CNAG_03769	9327.863	-0.3049	0.064768	-4.70749	2.51E-06	1.22E-05
CNAG_03771	10287.94	-0.82704	0.262463	-3.15109	0.001627	0.004565
CNAG_03772	7199.003	-2.73168	0.0967	-28.249	1.47E-175	2.53E-173
CNAG_07965	15860.78	-0.66434	0.067169	-9.89053	4.58E-23	7.50E-22
CNAG_03775	3966.318	-0.21583	0.060643	-3.55901	0.000372	0.001211
CNAG_03776	755.4853	-0.50567	0.09115	-5.54762	2.90E-08	1.81E-07
CNAG_03777	354.319	-0.61018	0.133462	-4.57193	4.83E-06	2.25E-05
CNAG_07481	582.3554	-0.36515	0.107275	-3.40382	0.000665	0.00204
CNAG_03781	1583.362	-0.30099	0.071912	-4.18561	2.84E-05	0.000115
CNAG_12169	5.79013	-2.88235	1.072404	-2.68774	0.007194	0.017227
CNAG_03782	51.72812	2.318165	0.39321	5.895488	3.74E-09	2.59E-08
CNAG_03784	18.95683	-1.69999	0.547909	-3.10268	0.001918	0.005315
CNAG_03788	625.9915	-0.7389	0.105913	-6.97652	3.03E-12	2.77E-11
CNAG_03790	4026.609	-0.23826	0.071743	-3.32101	0.000897	0.002665
CNAG_03794	716.1781	-0.57914	0.135512	-4.27374	1.92E-05	8.00E-05
CNAG_03796	1443.723	-0.51319	0.075987	-6.75361	1.44E-11	1.25E-10
CNAG_03805	2345.949	-0.14792	0.069487	-2.12878	0.033272	0.065977
CNAG_03806	830.7638	-0.24349	0.092699	-2.62663	0.008624	0.020281
CNAG_03807	109.9753	-0.5624	0.226972	-2.47785	0.013218	0.029596

CNAG_03808	566.4195	0.848836	0.106442	7.974671	1.53E-15	1.74E-14
CNAG_03809	2699.794	-0.50757	0.083841	-6.05397	1.41E-09	1.02E-08
CNAG_03810	2090.181	-0.16608	0.081711	-2.03254	0.042099	0.080894
CNAG_03811	1422.197	-0.17862	0.079676	-2.24186	0.02497	0.051499
CNAG_03812	4034.925	-0.30834	0.065272	-4.72385	2.31E-06	1.13E-05
CNAG_03814	3050.201	-0.26425	0.082275	-3.21176	0.001319	0.003772
CNAG_03815	1515.012	-0.48201	0.081469	-5.91652	3.29E-09	2.29E-08
CNAG_03817	3189.042	0.281243	0.072404	3.884385	0.000103	0.000375
CNAG_03818	1542.884	-0.18613	0.080178	-2.32145	0.020263	0.042878
CNAG_03819	10238.52	-0.39392	0.065348	-6.02806	1.66E-09	1.19E-08
CNAG_03822	1362.834	0.210746	0.084124	2.505193	0.012238	0.027632
CNAG_12174	34.55009	0.884281	0.375127	2.357284	0.018409	0.039418
CNAG_03826	1165.982	-0.39242	0.081574	-4.81064	1.50E-06	7.52E-06
CNAG_03828	173.7103	-0.47345	0.186797	-2.5346	0.011258	0.025739
CNAG_03829	1118.698	-0.51938	0.082038	-6.331	2.44E-10	1.90E-09
CNAG_03830	26.24843	-0.88817	0.440514	-2.01622	0.043777	0.083702
CNAG_03834	601.2459	-0.2548	0.109253	-2.3322	0.01969	0.041841
CNAG_03835	399.7989	-0.44345	0.148547	-2.98523	0.002834	0.007537
CNAG_03836	1206.835	-0.19457	0.088908	-2.18847	0.028636	0.058013
CNAG_03837	232.181	-0.51903	0.160917	-3.22542	0.001258	0.003609
CNAG_03839	2222.829	-0.21504	0.069525	-3.09295	0.001982	0.00547
CNAG_03841	3334.929	0.501141	0.079845	6.276407	3.46E-10	2.65E-09
CNAG_03844	665.0681	0.416929	0.10785	3.86582	0.000111	0.000402
CNAG_03847	1737.15	-0.49089	0.084904	-5.78164	7.40E-09	4.95E-08
CNAG_03848	316.1861	0.423483	0.150419	2.81536	0.004872	0.012211
CNAG_03853	4651.283	-0.28221	0.073294	-3.85035	0.000118	0.000426
CNAG_03854	636.0906	-0.37439	0.101537	-3.68717	0.000227	0.00077
CNAG_03855	386.4081	-0.39562	0.127252	-3.10898	0.001877	0.005213
CNAG_03856	7952.723	0.215328	0.059077	3.644884	0.000268	0.000895
CNAG_03857	3400.031	0.801598	0.070852	11.3137	1.12E-29	2.28E-28
CNAG_03859	3074.191	0.173417	0.071851	2.413548	0.015798	0.03449
CNAG_03861	12784.04	0.354587	0.073123	4.84921	1.24E-06	6.27E-06
CNAG_03864	1794.302	-0.4157	0.085919	-4.83827	1.31E-06	6.60E-06
CNAG_03865	1553.642	-0.48436	0.118751	-4.07879	4.53E-05	0.000176
CNAG_03866	561.5421	0.515903	0.118443	4.35571	1.33E-05	5.68E-05
CNAG_03870	1175.583	0.18516	0.078982	2.344328	0.019061	0.040643
CNAG_03871	1043.903	-0.2065	0.09042	-2.28382	0.022382	0.046742
CNAG_03872	1231.915	-0.34631	0.097699	-3.54465	0.000393	0.001269
CNAG_03873	2820.896	-0.79702	0.218067	-3.65494	0.000257	0.000863
CNAG_03874	2782.805	2.534447	0.08185	30.96471	1.61E-210	3.54E-208
CNAG_03875	1496.167	-0.52527	0.081982	-6.40708	1.48E-10	1.18E-09
CNAG_03876	2692.559	-0.51923	0.085744	-6.05556	1.40E-09	1.01E-08
CNAG_03877	1551.092	-0.25858	0.093413	-2.76811	0.005638	0.013907
CNAG_03878	1724.711	-0.17106	0.079827	-2.14284	0.032126	0.064031
CNAG_03882	782.9503	-0.303	0.090678	-3.34155	0.000833	0.002501
CNAG_03884	2432.595	-0.50248	0.069695	-7.2097	5.61E-13	5.48E-12
CNAG_03885	2156.129	-0.33095	0.081513	-4.06009	4.91E-05	0.00019

CNAG_03887	670.7872	-0.3688	0.100141	-3.68285	0.000231	0.000782
CNAG_03888	3082.239	-0.15164	0.064283	-2.35897	0.018326	0.03928
CNAG_03890	1809.013	-0.34031	0.074075	-4.59417	4.34E-06	2.04E-05
CNAG_03891	26894.58	1.053919	0.066397	15.87309	9.73E-57	3.82E-55
CNAG_03892	12655.85	0.802669	0.067359	11.91623	9.74E-33	2.14E-31
CNAG_03894	45.57833	-2.31914	0.3828	-6.05837	1.38E-09	9.93E-09
CNAG_12179	57.90976	-0.8335	0.292083	-2.85363	0.004322	0.010993
CNAG_12181	120.7518	0.972672	0.20059	4.849055	1.24E-06	6.27E-06
CNAG_03896	299.2441	-0.33236	0.132703	-2.50455	0.012261	0.027674
CNAG_12182	40.74616	-0.90007	0.338182	-2.66151	0.007779	0.018488
CNAG_03898	2271.565	0.605401	0.084592	7.156709	8.26E-13	7.95E-12
CNAG_03899	5537.731	0.705595	0.072789	9.693731	3.21E-22	5.02E-21
CNAG_03900	1167.038	-0.27922	0.093637	-2.98195	0.002864	0.007598
CNAG_03901	736.7676	0.253693	0.097734	2.59576	0.009438	0.021994
CNAG_03902	3438.86	0.222186	0.067283	3.302281	0.000959	0.002839
CNAG_03903	1189.753	0.392375	0.085469	4.590849	4.41E-06	2.07E-05
CNAG_12185	47.88524	-1.29115	0.330687	-3.90446	9.44E-05	0.000348
CNAG_03904	3357.156	0.153146	0.067785	2.259303	0.023865	0.049457
CNAG_03906	427.4322	0.368436	0.126901	2.903343	0.003692	0.00953
CNAG_03905	548.8287	-0.46312	0.129989	-3.56276	0.000367	0.001195
CNAG_03908	1043.805	0.192402	0.088961	2.162765	0.030559	0.06133
CNAG_03909	669.4801	1.169867	0.143199	8.169534	3.10E-16	3.64E-15
CNAG_03910	1862.151	0.814941	0.320315	2.544188	0.010953	0.025092
CNAG_03911	273.6764	1.023427	0.143647	7.124617	1.04E-12	9.95E-12
CNAG_03912	1362.29	2.618863	0.090733	28.86337	3.44E-183	6.08E-181
CNAG_03914	855.9375	0.280139	0.093041	3.010916	0.002605	0.006978
CNAG_03915	572.7982	0.390488	0.099844	3.910958	9.19E-05	0.00034
CNAG_03916	11586.75	-0.18419	0.069242	-2.6601	0.007812	0.01856
CNAG_03919	636.5416	-0.63007	0.116387	-5.41357	6.18E-08	3.73E-07
CNAG_03920	7782.429	-0.14447	0.056207	-2.57032	0.01016	0.023421
CNAG_12188	49.8418	0.687238	0.315098	2.181028	0.029181	0.058928
CNAG_03922	940.0425	3.041622	0.15532	19.58297	2.16E-85	1.29E-83
CNAG_03924	383.3117	0.415471	0.124801	3.329081	0.000871	0.002605
CNAG_03927	1189.769	0.968445	0.09571	10.11852	4.57E-24	7.73E-23
CNAG_03930	697.9234	-0.20935	0.098226	-2.13133	0.033062	0.065608
CNAG_03931	10352.27	0.114169	0.056042	2.037204	0.04163	0.080087
CNAG_03933	433.7794	-0.22836	0.115325	-1.98011	0.047691	0.090065
CNAG_03937	749.518	-1.01462	0.104726	-9.68825	3.38E-22	5.29E-21
CNAG_03939	4862.001	1.893163	0.078179	24.21571	1.52E-129	1.49E-127
CNAG_03940	4407.726	0.275596	0.062957	4.377551	1.20E-05	5.19E-05
CNAG_07485	714.4179	-0.24484	0.092928	-2.63475	0.00842	0.019837
CNAG_03946	931.2078	-0.2725	0.111138	-2.4519	0.01421	0.031497
CNAG_03949	1082.808	-0.77074	0.082858	-9.30193	1.38E-20	2.01E-19
CNAG_03950	5367.558	-0.15671	0.066447	-2.35844	0.018352	0.039316
CNAG_03955	1106.278	-0.20178	0.093844	-2.15019	0.03154	0.063094
CNAG_03956	804.3783	0.254929	0.08976	2.840129	0.00451	0.011394
CNAG_03957	1813.46	-0.60459	0.0821	-7.36407	1.78E-13	1.80E-12

CNAG_03960	1315.393	0.559352	0.07741	7.225816	4.98E-13	4.90E-12
CNAG_03961	2089.726	-0.17988	0.069056	-2.60478	0.009193	0.021507
CNAG_03963	475.8948	-0.35783	0.117576	-3.04338	0.002339	0.006338
CNAG_03965	1845.176	0.165865	0.079763	2.079466	0.037575	0.073362
CNAG_03966	1989.782	0.671846	0.077647	8.65252	5.04E-18	6.44E-17
CNAG_03967	2393.284	-0.27075	0.070845	-3.82171	0.000133	0.000473
CNAG_03968	3614.566	-0.41799	0.072647	-5.7538	8.73E-09	5.77E-08
CNAG_07487	15823.83	-0.20952	0.086337	-2.42683	0.015231	0.033432
CNAG_07488	3164.919	-0.31959	0.08278	-3.86069	0.000113	0.000409
CNAG_03971	18.91067	-1.07176	0.510554	-2.09922	0.035798	0.07023
CNAG_03973	1997.724	1.005432	0.075282	13.35548	1.10E-40	3.06E-39
CNAG_03974	1372.652	0.693879	0.120552	5.755835	8.62E-09	5.71E-08
CNAG_03976	1111.845	-0.60427	0.125684	-4.80782	1.53E-06	7.62E-06
CNAG_03977	1470.77	-0.2422	0.092986	-2.60472	0.009195	0.021507
CNAG_03981	1443.7	0.268033	0.081419	3.292024	0.000995	0.002929
CNAG_03982	1822.952	0.194723	0.072815	2.674206	0.007491	0.01787
CNAG_03983	6410.341	-0.62815	0.056946	-11.0307	2.72E-28	5.25E-27
CNAG_03984	1586.935	1.160977	0.113052	10.26937	9.68E-25	1.68E-23
CNAG_03985	6645.362	2.145939	0.073664	29.13136	1.44E-186	2.66E-184
CNAG_03986	3468.734	-0.17151	0.080245	-2.13731	0.032572	0.064778
CNAG_03987	4011.175	-0.38931	0.079674	-4.88626	1.03E-06	5.26E-06
CNAG_03988	142.1106	-0.88263	0.202001	-4.36943	1.25E-05	5.37E-05
CNAG_03989	794.9008	-0.41507	0.093564	-4.43626	9.15E-06	4.06E-05
CNAG_03990	43.57508	-2.17886	0.375442	-5.80345	6.50E-09	4.38E-08
CNAG_03991	890.222	-0.5392	0.091657	-5.88285	4.03E-09	2.77E-08
CNAG_03992	616.9222	0.252234	0.098245	2.56741	0.010246	0.023586
CNAG_03994	748.6258	-0.34887	0.09908	-3.52111	0.00043	0.001376
CNAG_03995	529.9081	-0.41081	0.134103	-3.06337	0.002189	0.005965
CNAG_03996	6807.393	0.242093	0.067009	3.612842	0.000303	0.001002
CNAG_03997	120.7226	0.760606	0.219865	3.459429	0.000541	0.001695
CNAG_03998	1273.808	0.403728	0.079432	5.082676	3.72E-07	2.03E-06
CNAG_03999	337.7861	1.882006	0.162927	11.55122	7.28E-31	1.53E-29
CNAG_04000	907.0582	-0.23608	0.0887	-2.66154	0.007778	0.018488
CNAG_04002	1412.974	-0.53353	0.090292	-5.9089	3.44E-09	2.40E-08
CNAG_04004	99206.93	-0.33413	0.057001	-5.8618	4.58E-09	3.13E-08
CNAG_04005	3204.746	-0.21361	0.069226	-3.08568	0.002031	0.005582
CNAG_12197	120.5523	-0.62368	0.206287	-3.02336	0.0025	0.006724
CNAG_04011	25099.18	-0.38139	0.074469	-5.12155	3.03E-07	1.67E-06
CNAG_04013	1470.232	0.204	0.094427	2.160409	0.030741	0.061634
CNAG_04014	4936.87	0.189055	0.064779	2.918487	0.003517	0.009134
CNAG_12198	50.82786	-1.2021	0.308976	-3.89059	0.0001	0.000367
CNAG_04015	52.43847	-1.10821	0.300537	-3.68745	0.000227	0.000769
CNAG_04017	1196.78	0.821158	0.085547	9.598906	8.08E-22	1.24E-20
CNAG_07489	1175.621	0.457763	0.120967	3.78419	0.000154	0.000544
CNAG_04021	30411.85	-0.21032	0.073704	-2.8536	0.004323	0.010993
CNAG_04022	2896.522	-0.57045	0.071559	-7.97173	1.56E-15	1.78E-14
CNAG_04023	1660.112	-0.82501	0.121457	-6.79255	1.10E-11	9.69E-11

CNAG_04024	268.5063	-0.34017	0.139806	-2.43314	0.014968	0.032935
CNAG_04025	102.8059	1.194736	0.297875	4.010865	6.05E-05	0.000231
CNAG_04026	875.118	0.274448	0.098389	2.789412	0.00528	0.013129
CNAG_04027	473.6225	1.391235	0.13053	10.65836	1.59E-26	2.87E-25
CNAG_04028	13487.94	-0.14333	0.066912	-2.14211	0.032185	0.064113
CNAG_04029	514.457	1.235129	0.110245	11.2035	3.92E-29	7.83E-28
CNAG_04031	625.2437	-0.26119	0.105271	-2.48112	0.013097	0.029371
CNAG_04032	6355.326	0.144692	0.061238	2.362784	0.018138	0.03894
CNAG_04033	2193.001	-0.21259	0.072317	-2.93975	0.003285	0.008582
CNAG_04035	1101.1	0.270467	0.086854	3.114039	0.001845	0.00513
CNAG_04036	257.5827	1.02071	0.15937	6.404674	1.51E-10	1.19E-09
CNAG_04037	2108.83	0.201378	0.089725	2.244398	0.024807	0.051188
CNAG_12200	114.4661	1.506982	0.234569	6.424461	1.32E-10	1.06E-09
CNAG_04039	3161.673	0.803606	0.074836	10.73817	6.74E-27	1.24E-25
CNAG_04040	2032.135	-0.32612	0.071427	-4.5658	4.98E-06	2.31E-05
CNAG_04044	1867.174	-0.21156	0.081533	-2.59474	0.009466	0.022042
CNAG_04046	787.806	-0.45408	0.112368	-4.04103	5.32E-05	0.000205
CNAG_04048	2861.161	-0.25614	0.076202	-3.36138	0.000776	0.002347
CNAG_04050	5555.769	-0.24393	0.078759	-3.09717	0.001954	0.005401
CNAG_04052	1544.934	0.273404	0.077596	3.523456	0.000426	0.001365
CNAG_04053	988.9508	0.204919	0.089998	2.276927	0.022791	0.047498
CNAG_04055	744.4831	0.399052	0.096827	4.121279	3.77E-05	0.000149
CNAG_04056	2603.306	1.218776	0.072788	16.74408	6.26E-63	2.81E-61
CNAG_07491	1077.513	1.279498	0.113045	11.3185	1.06E-29	2.16E-28
CNAG_07492	39.75301	1.260264	0.352681	3.573382	0.000352	0.001151
CNAG_07493	163.2604	1.399439	0.186241	7.514147	5.73E-14	5.98E-13
CNAG_04062	3167.545	0.212394	0.063066	3.367812	0.000758	0.002301
CNAG_04064	7286.707	0.489169	0.078853	6.203533	5.52E-10	4.14E-09
CNAG_04065	181.067	-1.16993	0.19531	-5.99013	2.10E-09	1.49E-08
CNAG_04067	373.1085	-0.45722	0.136217	-3.35659	0.000789	0.002383
CNAG_04068	17653.83	-0.15425	0.075268	-2.04935	0.040428	0.07822
CNAG_12201	122.8126	-0.87331	0.203249	-4.29674	1.73E-05	7.28E-05
CNAG_04069	3026.105	-0.16917	0.06986	-2.4215	0.015457	0.033845
CNAG_04070	130.1682	-0.64315	0.201226	-3.19614	0.001393	0.003964
CNAG_04071	2707.554	0.179212	0.070904	2.527517	0.011487	0.026161
CNAG_04073	2869.957	0.398556	0.072605	5.489362	4.03E-08	2.47E-07
CNAG_04076	848.5114	0.373691	0.087434	4.273971	1.92E-05	7.99E-05
CNAG_12205	28.81701	1.64877	0.458397	3.596815	0.000322	0.001061
CNAG_04078	1963.956	0.25949	0.072788	3.565015	0.000364	0.001185
CNAG_04079	1829.294	0.233135	0.079141	2.945795	0.003221	0.008432
CNAG_12209	150.7929	0.422599	0.20553	2.05614	0.039769	0.077036
CNAG_12210	256.8104	0.600274	0.137048	4.380019	1.19E-05	5.13E-05
CNAG_04086	176.8476	0.628561	0.171668	3.6615	0.000251	0.000845
CNAG_04087	221.8126	0.310953	0.149734	2.076699	0.037829	0.073789
CNAG_04089	2587.229	-0.23952	0.082478	-2.90403	0.003684	0.009515
CNAG_04090	2492.377	-0.53077	0.083886	-6.32721	2.50E-10	1.95E-09
CNAG_04091	204.9462	1.159249	0.179525	6.457326	1.07E-10	8.59E-10

CNAG_04092	93.73175	-0.61645	0.218032	-2.82733	0.004694	0.011815
CNAG_04093	71.92766	-0.90903	0.274693	-3.30924	0.000936	0.002773
CNAG_04097	162.434	0.909424	0.17496	5.197905	2.02E-07	1.13E-06
CNAG_04098	452.9101	-0.48534	0.117884	-4.11711	3.84E-05	0.000152
CNAG_04099	427.9522	0.258182	0.111265	2.32043	0.020318	0.04295
CNAG_07497	59.43192	-1.35253	0.305909	-4.42135	9.81E-06	4.33E-05
CNAG_12212	594.6968	0.458687	0.112041	4.093915	4.24E-05	0.000166
CNAG_06984	1253.675	0.319977	0.08433	3.794336	0.000148	0.000524
CNAG_07498	876.1435	0.7091	0.089106	7.957947	1.75E-15	1.98E-14
CNAG_06973	126.3821	0.623627	0.211694	2.945887	0.00322	0.008432
CNAG_06963	8683.637	-0.33374	0.084614	-3.94432	8.00E-05	0.000299
CNAG_06962	428.1289	-0.57627	0.118919	-4.84591	1.26E-06	6.37E-06
CNAG_03081	3104.815	0.198868	0.061566	3.230134	0.001237	0.003557
CNAG_03079	703.9364	-0.3117	0.098007	-3.1804	0.001471	0.004158
CNAG_03078	1530.993	-0.22284	0.078353	-2.8441	0.004454	0.011274
CNAG_03076	212.5413	0.717108	0.15354	4.670492	3.00E-06	1.44E-05
CNAG_12217	829.1505	1.230059	0.120186	10.23466	1.39E-24	2.38E-23
CNAG_03072	44265.27	0.41312	0.053327	7.746978	9.41E-15	1.02E-13
CNAG_03071	779.4531	0.50613	0.100947	5.013824	5.34E-07	2.85E-06
CNAG_03070	1207.86	-0.38432	0.082732	-4.64537	3.39E-06	1.62E-05
CNAG_03069	831.035	-0.42582	0.098224	-4.33523	1.46E-05	6.20E-05
CNAG_03068	400.1139	-1.67928	0.141223	-11.891	1.32E-32	2.88E-31
CNAG_07502	9716.78	0.331171	0.06006	5.513986	3.51E-08	2.17E-07
CNAG_03065	5346.353	-0.39289	0.07485	-5.24901	1.53E-07	8.77E-07
CNAG_03064	2425.134	0.197644	0.086901	2.274359	0.022944	0.047782
CNAG_03063	3856.242	0.420295	0.066843	6.287815	3.22E-10	2.48E-09
CNAG_07967	1070.897	-0.29775	0.101161	-2.94331	0.003247	0.008495
CNAG_03059	902.5381	0.443535	0.090642	4.893267	9.92E-07	5.09E-06
CNAG_03058	10784.55	-0.94757	0.210454	-4.50251	6.72E-06	3.06E-05
CNAG_03057	293.2537	-0.70713	0.169906	-4.16188	3.16E-05	0.000126
CNAG_12220	7.122724	1.845759	0.818242	2.255761	0.024086	0.049877
CNAG_03056	63.30021	-1.72398	0.296668	-5.81114	6.20E-09	4.21E-08
CNAG_03051	1071.808	0.624848	0.08626	7.243775	4.36E-13	4.31E-12
CNAG_03049	2717.636	-0.27058	0.068171	-3.96914	7.21E-05	0.000272
CNAG_03047	815.1413	0.907579	0.092773	9.782791	1.33E-22	2.12E-21
CNAG_12222	65.39244	0.536919	0.269318	1.993623	0.046193	0.087642
CNAG_03040	2114.273	-0.60734	0.096622	-6.28572	3.26E-10	2.50E-09
CNAG_03038	3202.722	0.206263	0.08814	2.340169	0.019275	0.041034
CNAG_12223	101.9334	0.529867	0.260496	2.034072	0.041944	0.080635
CNAG_03033	769.1853	0.216822	0.09481	2.286907	0.022201	0.046436
CNAG_03031	636.4608	-0.52304	0.09848	-5.31111	1.09E-07	6.35E-07
CNAG_03027	3485.684	-0.13748	0.068729	-2.00035	0.045463	0.086498
CNAG_03025	1699.73	-0.23681	0.076801	-3.08345	0.002046	0.005613
CNAG_03024	1424.822	0.212868	0.081926	2.598278	0.009369	0.021845
CNAG_03023	1810.117	0.263119	0.08102	3.247562	0.001164	0.003372
CNAG_03022	674.5312	-0.5152	0.103587	-4.97356	6.57E-07	3.46E-06
CNAG_03020	760.0473	-0.33469	0.09563	-3.49986	0.000465	0.001477

CNAG_03019	1613.886	0.296231	0.084508	3.505347	0.000456	0.001451
CNAG_03016	1083.369	-0.19751	0.081673	-2.41834	0.015591	0.034103
CNAG_03013	1136.279	0.834093	0.08543	9.763474	1.62E-22	2.55E-21
CNAG_03012	57063.79	0.198323	0.097231	2.039712	0.041379	0.07968
CNAG_12229	56.68434	1.345707	0.309547	4.347341	1.38E-05	5.89E-05
CNAG_03011	608.7692	3.097129	0.11894	26.03951	1.77E-149	2.28E-147
CNAG_03009	675.4265	-0.37923	0.125143	-3.03034	0.002443	0.006593
CNAG_03007	12481.93	2.797286	0.086586	32.30632	5.70E-229	1.49E-226
CNAG_12230	85.24824	0.467178	0.227478	2.053731	0.040002	0.07745
CNAG_03004	1195.077	-0.83292	0.094832	-8.78312	1.59E-18	2.07E-17
CNAG_03000	56225.19	-0.27348	0.079815	-3.42639	0.000612	0.001898
CNAG_02999	777.1163	-0.25096	0.102551	-2.44717	0.014398	0.031853
CNAG_02998	1830.42	-0.40834	0.070874	-5.76154	8.34E-09	5.54E-08
CNAG_02995	3710.444	-0.40922	0.081879	-4.9979	5.80E-07	3.07E-06
CNAG_02994	9596.967	-0.96398	0.085172	-11.318	1.07E-29	2.17E-28
CNAG_02993	1074.547	-0.24552	0.092587	-2.65171	0.008009	0.018956
CNAG_02992	583.6595	0.463527	0.10977	4.222713	2.41E-05	9.82E-05
CNAG_02991	7961.987	-0.20497	0.066269	-3.09295	0.001982	0.00547
CNAG_02990	2419.551	0.654934	0.102759	6.37349	1.85E-10	1.46E-09
CNAG_02986	1133.015	-0.25653	0.12441	-2.06201	0.039207	0.076092
CNAG_02984	62.08059	-2.10612	0.311021	-6.77165	1.27E-11	1.11E-10
CNAG_02982	3053.373	0.192366	0.073324	2.623486	0.008704	0.020446
CNAG_02978	397.4024	1.807336	0.137395	13.15436	1.61E-39	4.32E-38
CNAG_02977	2846.645	0.187668	0.06765	2.774078	0.005536	0.013689
CNAG_02974	26253.5	-0.36967	0.072537	-5.09624	3.46E-07	1.89E-06
CNAG_02973	1004.69	-0.27283	0.082498	-3.30705	0.000943	0.002793
CNAG_02969	1130.82	1.578982	0.123852	12.7489	3.16E-37	7.98E-36
CNAG_02968	743.2274	1.80435	0.121658	14.83138	9.18E-50	3.11E-48
CNAG_02966	260.3101	1.160928	0.210389	5.518003	3.43E-08	2.12E-07
CNAG_02964	458.073	0.241491	0.117273	2.059225	0.039473	0.076553
CNAG_02963	742.3496	-0.23716	0.109167	-2.17247	0.02982	0.060069
CNAG_02960	416.8997	-0.78285	0.138274	-5.66157	1.50E-08	9.65E-08
CNAG_02959	27170.45	5.385603	0.093582	57.54935	0	0
CNAG_02958	10857.39	4.862178	0.088041	55.22649	0	0
CNAG_07508	866.8292	0.561479	0.101002	5.559097	2.71E-08	1.70E-07
CNAG_07509	2243.583	0.180614	0.073887	2.444485	0.014506	0.032039
CNAG_07510	4104.222	-0.31294	0.069937	-4.47459	7.66E-06	3.46E-05
CNAG_02955	491.4327	-0.48282	0.108879	-4.43448	9.23E-06	4.09E-05
CNAG_02951	812.0128	-0.30011	0.098343	-3.05166	0.002276	0.006182
CNAG_02950	2254.446	0.398645	0.072511	5.497733	3.85E-08	2.37E-07
CNAG_02948	10134.68	-0.25233	0.056048	-4.50197	6.73E-06	3.07E-05
CNAG_07511	6366.415	0.167888	0.072404	2.318777	0.020407	0.043075
CNAG_07512	613.9858	0.862814	0.103986	8.297413	1.06E-16	1.28E-15
CNAG_02944	9978.511	4.921192	0.070282	70.02076	0	0
CNAG_02943	14274.28	-0.23427	0.072901	-3.21358	0.001311	0.003752
CNAG_02941	1246.918	-0.44728	0.07912	-5.65312	1.58E-08	1.01E-07
CNAG_02940	1171.947	0.2761	0.085184	3.24121	0.00119	0.003437

CNAG_02939	1293.446	-0.51592	0.08667	-5.95275	2.64E-09	1.85E-08
CNAG_02938	9654.985	0.35374	0.078299	4.517813	6.25E-06	2.87E-05
CNAG_02936	5318.053	-0.20749	0.061143	-3.39357	0.00069	0.002113
CNAG_02935	2825.85	1.860464	0.06868	27.08885	1.33E-161	1.87E-159
CNAG_02934	157.7101	1.33767	0.177214	7.54832	4.41E-14	4.62E-13
CNAG_02933	700.4543	-0.71046	0.141703	-5.01375	5.34E-07	2.85E-06
CNAG_02928	58207.12	-0.1448	0.071485	-2.02564	0.042802	0.082069
CNAG_02925	4782.434	-0.7956	0.089075	-8.93174	4.19E-19	5.69E-18
CNAG_02923	2794.414	-0.20479	0.078693	-2.60233	0.009259	0.021639
CNAG_02922	1859.653	-0.30403	0.073079	-4.16028	3.18E-05	0.000127
CNAG_02921	3636.334	0.434502	0.068973	6.299579	2.98E-10	2.31E-09
CNAG_02919	544.7185	0.341441	0.109275	3.124603	0.00178	0.004958
CNAG_12238	165.8138	1.122372	0.198622	5.650806	1.60E-08	1.02E-07
CNAG_02918	10417.61	-0.73571	0.094133	-7.81557	5.47E-15	6.03E-14
CNAG_02910	678.6401	-0.34143	0.146522	-2.3302	0.019795	0.042021
CNAG_07513	1729.324	0.335157	0.082109	4.081868	4.47E-05	0.000174
CNAG_02904	307.6311	-0.32029	0.132479	-2.41767	0.01562	0.034148
CNAG_02903	1563.707	0.217299	0.085939	2.528524	0.011454	0.026101
CNAG_02902	430.449	-0.26122	0.113782	-2.29579	0.021688	0.045479
CNAG_02901	2586.876	-0.35254	0.094151	-3.74445	0.000181	0.000628
CNAG_02900	283.6691	1.701873	0.165188	10.30261	6.86E-25	1.19E-23
CNAG_12243	62.34547	0.757637	0.316654	2.392636	0.016728	0.036305
CNAG_02898	2852.961	-0.38151	0.077387	-4.92997	8.22E-07	4.27E-06
CNAG_02897	1021.888	-0.27847	0.097906	-2.84431	0.004451	0.01127
CNAG_02896	4106.511	-1.25793	0.082763	-15.1991	3.58E-52	1.27E-50
CNAG_02893	270.8289	0.717938	0.150346	4.775239	1.79E-06	8.90E-06
CNAG_02888	430.3248	1.236371	0.119191	10.37305	3.29E-25	5.77E-24
CNAG_02885	4446.753	-0.35522	0.066254	-5.36153	8.25E-08	4.89E-07
CNAG_12244	61.44315	-0.69612	0.287015	-2.42536	0.015293	0.033541
CNAG_02880	21901.02	-0.16842	0.0542	-3.10744	0.001887	0.005235
CNAG_02879	1566.105	-0.32296	0.075318	-4.28795	1.80E-05	7.54E-05
CNAG_02878	1552.261	0.194155	0.076827	2.527183	0.011498	0.026171
CNAG_02875	1414.369	-0.21423	0.085279	-2.51213	0.012	0.02717
CNAG_02871	458.9689	-0.42239	0.118709	-3.55819	0.000373	0.001214
CNAG_02870	2571.796	-0.63197	0.087495	-7.22289	5.09E-13	5.00E-12
CNAG_02869	227.149	-0.82948	0.153179	-5.41509	6.13E-08	3.70E-07
CNAG_12248	126.089	-0.9639	0.223313	-4.31636	1.59E-05	6.70E-05
CNAG_02868	1129.952	-0.45305	0.101405	-4.46769	7.91E-06	3.55E-05
CNAG_02867	2185.057	0.574823	0.078439	7.328262	2.33E-13	2.33E-12
CNAG_02866	1114.918	-0.5676	0.093575	-6.06576	1.31E-09	9.51E-09
CNAG_12249	41.66207	1.546807	0.35403	4.369145	1.25E-05	5.37E-05
CNAG_02865	227.5908	1.102678	0.179118	6.156156	7.45E-10	5.52E-09
CNAG_02864	708.8817	1.212536	0.136215	8.90164	5.50E-19	7.42E-18
CNAG_12250	1636.238	0.708615	0.084702	8.365934	5.96E-17	7.29E-16
CNAG_12252	29.39639	1.25731	0.468779	2.682098	0.007316	0.017495
CNAG_02860	9163.356	-0.14413	0.068322	-2.10963	0.03489	0.068666
CNAG_02859	578.9676	-0.39692	0.117986	-3.36413	0.000768	0.002328

CNAG_02858	22206.69	-0.25408	0.055049	-4.61545	3.92E-06	1.85E-05
CNAG_02856	3695.082	0.167519	0.062068	2.698938	0.006956	0.016713
CNAG_02854	1516.054	-0.52505	0.07603	-6.90587	4.99E-12	4.50E-11
CNAG_02852	1081.212	1.174118	0.100468	11.68648	1.49E-31	3.17E-30
CNAG_02850	12791.95	-0.52601	0.067678	-7.77217	7.72E-15	8.39E-14
CNAG_02848	382.2539	0.419414	0.147474	2.843992	0.004455	0.011274
CNAG_02847	621.825	-0.20773	0.105387	-1.97108	0.048715	0.091571
CNAG_02846	410.3426	0.384145	0.137008	2.803807	0.00505	0.012619
CNAG_12259	67.19973	1.012101	0.349403	2.896658	0.003772	0.00972
CNAG_02840	735.2634	0.390327	0.115908	3.367549	0.000758	0.002303
CNAG_02839	1388.165	5.30373	0.131708	40.26898	0	0
CNAG_07519	2867.946	2.437587	0.073116	33.33865	1.06E-243	3.20E-241
CNAG_02834	611.3254	-0.25644	0.099903	-2.56689	0.010262	0.023614
CNAG_02833	2884.369	0.443286	0.088606	5.002883	5.65E-07	3.00E-06
CNAG_07520	216.2484	0.758763	0.160052	4.740743	2.13E-06	1.05E-05
CNAG_02830	5172.701	-0.94767	0.085052	-11.1422	7.81E-29	1.54E-27
CNAG_02828	148.5009	0.502919	0.192911	2.606995	0.009134	0.021384
CNAG_02827	1190.376	0.22531	0.10149	2.220015	0.026418	0.054114
CNAG_02826	914.243	0.584532	0.094121	6.210403	5.28E-10	3.97E-09
CNAG_02825	5664.456	-0.14083	0.064525	-2.18263	0.029063	0.058748
CNAG_07521	211.4469	-0.627	0.152835	-4.10244	4.09E-05	0.000161
CNAG_12262	9.069974	-1.89385	0.783671	-2.41663	0.015665	0.034217
CNAG_02822	4769.818	-0.5493	0.065941	-8.33017	8.07E-17	9.79E-16
CNAG_02821	394.2103	-0.38297	0.126056	-3.03809	0.002381	0.00644
CNAG_02819	815.8178	0.41059	0.098154	4.183119	2.88E-05	0.000116
CNAG_02818	2108.433	0.814328	0.075789	10.74473	6.27E-27	1.16E-25
CNAG_02817	3964.763	-0.19868	0.063782	-3.11502	0.001839	0.005115
CNAG_02815	4220.686	0.783371	0.287198	2.72763	0.006379	0.015487
CNAG_02814	7153.239	-0.6262	0.079389	-7.88771	3.08E-15	3.44E-14
CNAG_02812	3751.418	-0.24494	0.064432	-3.80148	0.000144	0.00051
CNAG_07522	1664.705	-0.46794	0.106831	-4.38015	1.19E-05	5.13E-05
CNAG_02801	7856.096	-0.21305	0.068944	-3.09021	0.002	0.005515
CNAG_02794	3112.826	-0.38526	0.097202	-3.96343	7.39E-05	0.000278
CNAG_02790	2267.268	0.620686	0.068687	9.036462	1.62E-19	2.25E-18
CNAG_02788	1383.201	0.954211	0.087567	10.8969	1.19E-27	2.27E-26
CNAG_02787	1067.872	-0.56106	0.084416	-6.64645	3.00E-11	2.54E-10
CNAG_02786	1589.147	-0.19891	0.075345	-2.63999	0.008291	0.019559
CNAG_02783	1013.958	0.274556	0.087497	3.137901	0.001702	0.004758
CNAG_02782	1339.408	-0.35445	0.078847	-4.49533	6.95E-06	3.16E-05
CNAG_02781	69.68701	0.810764	0.258429	3.137283	0.001705	0.004766
CNAG_02780	2408.792	-0.31259	0.070727	-4.41962	9.89E-06	4.36E-05
CNAG_02779	2464.811	-0.17936	0.068207	-2.62965	0.008547	0.020108
CNAG_02777	9556.577	2.050762	0.103911	19.73575	1.06E-86	6.55E-85
CNAG_12271	120.161	3.805009	0.279688	13.60448	3.77E-42	1.07E-40
CNAG_07523	454.6127	0.246108	0.114627	2.147032	0.031791	0.063488
CNAG_02776	359.6756	0.366785	0.136951	2.678214	0.007402	0.017684
CNAG_02775	4863.762	1.704045	0.080324	21.21475	6.98E-100	5.11E-98

CNAG_02773	3847.16	0.178322	0.061685	2.890847	0.003842	0.009882
CNAG_02771	1342.11	0.480089	0.086376	5.558146	2.73E-08	1.70E-07
CNAG_02770	804.912	-0.34088	0.097871	-3.48298	0.000496	0.001562
CNAG_02769	1655.951	-0.23251	0.075254	-3.08964	0.002004	0.005523
CNAG_02768	89.65675	-0.68108	0.243856	-2.79295	0.005223	0.013006
CNAG_02763	4325.378	-0.2027	0.061542	-3.29361	0.000989	0.002916
CNAG_02762	11724.94	-0.24308	0.081507	-2.98234	0.002861	0.007591
CNAG_12272	25.83888	1.171141	0.431757	2.7125	0.006678	0.01613
CNAG_02759	220.8623	0.414368	0.147563	2.808088	0.004984	0.012464
CNAG_02758	641.1155	1.754309	0.108475	16.17245	7.89E-59	3.26E-57
CNAG_02753	3805.318	-0.5624	0.064767	-8.68343	3.84E-18	4.95E-17
CNAG_02752	3490.059	1.045955	0.078813	13.27133	3.40E-40	9.32E-39
CNAG_02750	354.8119	0.347336	0.135857	2.556634	0.010569	0.024288
CNAG_02745	2825.472	0.140686	0.065438	2.149909	0.031562	0.063094
CNAG_02744	911.9062	0.368674	0.091565	4.02638	5.66E-05	0.000217
CNAG_02742	1419.123	-0.23993	0.083809	-2.86278	0.004199	0.0107
CNAG_02739	1426.898	-0.51551	0.085681	-6.01664	1.78E-09	1.27E-08
CNAG_02738	2325.382	0.165257	0.073868	2.237195	0.025274	0.05198
CNAG_02736	6937.413	-0.1255	0.05952	-2.10847	0.034991	0.068846
CNAG_02735	169.4656	1.160996	0.23253	4.992888	5.95E-07	3.15E-06
CNAG_02734	19.07818	2.040913	0.581477	3.509878	0.000448	0.001428
CNAG_02733	83.25345	0.583315	0.252957	2.305984	0.021112	0.044328
CNAG_02730	917.0801	-0.34466	0.103625	-3.32604	0.000881	0.002627
CNAG_02726	4607.304	-0.16582	0.074068	-2.23881	0.025168	0.051803
CNAG_02725	3767.889	0.132007	0.06169	2.139835	0.032368	0.064451
CNAG_12274	149.4084	0.787107	0.200125	3.933086	8.39E-05	0.000312
CNAG_02723	1393.03	-0.35068	0.078713	-4.45513	8.38E-06	3.75E-05
CNAG_12277	48.16274	2.381169	0.350259	6.798317	1.06E-11	9.34E-11
CNAG_02721	2228.211	-0.23893	0.077008	-3.10265	0.001918	0.005315
CNAG_02718	1366.645	-0.29002	0.086008	-3.37201	0.000746	0.002273
CNAG_02717	1276.726	-0.35686	0.084476	-4.22441	2.40E-05	9.76E-05
CNAG_02716	827.7525	0.22444	0.091905	2.442082	0.014603	0.032236
CNAG_02715	1019.052	0.226243	0.090477	2.50056	0.0124	0.027957
CNAG_02714	34971.21	-0.15397	0.070675	-2.17862	0.02936	0.059259
CNAG_02713	748.4753	-0.40544	0.113338	-3.57727	0.000347	0.001136
CNAG_02712	2205.642	-0.7847	0.082798	-9.47728	2.61E-21	3.96E-20
CNAG_02711	46.1168	-1.25396	0.317104	-3.95442	7.67E-05	0.000288
CNAG_02710	8732.624	-0.17604	0.061607	-2.85739	0.004271	0.010877
CNAG_02708	525.4763	-0.48217	0.125024	-3.85661	0.000115	0.000416
CNAG_02705	152.7764	-1.60172	0.225954	-7.08871	1.35E-12	1.28E-11
CNAG_02704	1752.818	-0.3083	0.092502	-3.33293	0.000859	0.002575
CNAG_02703	2637.826	0.360315	0.081672	4.411714	1.03E-05	4.50E-05
CNAG_02702	2049.399	-0.33586	0.08561	-3.92311	8.74E-05	0.000325
CNAG_02701	374.5981	0.608484	0.208586	2.917188	0.003532	0.00916
CNAG_12282	10.99249	1.711717	0.678865	2.521441	0.011688	0.02655
CNAG_12285	285.8993	1.302626	0.146594	8.88592	6.34E-19	8.50E-18
CNAG_02697	1623.376	1.60856	0.087328	18.41971	9.13E-76	4.85E-74

CNAG_02695	1704.055	-0.18593	0.075892	-2.44999	0.014286	0.031648
CNAG_02694	60.00179	0.550812	0.275707	1.997818	0.045736	0.086938
CNAG_02692	6111.295	1.427691	0.094182	15.1589	6.62E-52	2.32E-50
CNAG_02690	1142.358	0.694509	0.107509	6.459998	1.05E-10	8.46E-10
CNAG_02689	453.6595	-0.31144	0.110367	-2.82188	0.004774	0.011977
CNAG_02687	3026.148	0.468014	0.066398	7.048587	1.81E-12	1.69E-11
CNAG_02686	3772.054	-0.26635	0.066817	-3.9863	6.71E-05	0.000254
CNAG_02684	1300.4	0.594786	0.088601	6.713082	1.91E-11	1.63E-10
CNAG_02683	1731.156	-0.68079	0.090241	-7.54412	4.55E-14	4.77E-13
CNAG_02675	3944.987	-0.36407	0.072611	-5.01401	5.33E-07	2.85E-06
CNAG_02674	549.876	0.839302	0.138214	6.072481	1.26E-09	9.16E-09
CNAG_02673	4521.968	-0.33711	0.105529	-3.19444	0.001401	0.003986
CNAG_02672	4515.097	0.448045	0.068691	6.522585	6.91E-11	5.66E-10
CNAG_02671	7126.599	-0.18252	0.067377	-2.70894	0.00675	0.01628
CNAG_02670	1107.84	-0.28279	0.088625	-3.19092	0.001418	0.004028
CNAG_02666	734.3896	0.372013	0.092801	4.008707	6.11E-05	0.000233
CNAG_02665	202.095	1.15127	0.183836	6.26249	3.79E-10	2.89E-09
CNAG_02664	3681.5	-0.27488	0.071002	-3.87151	0.000108	0.000393
CNAG_02663	1188.403	0.579051	0.096801	5.981881	2.21E-09	1.56E-08
CNAG_12292	67.754	1.847354	0.307487	6.007904	1.88E-09	1.34E-08
CNAG_12295	79.49224	1.318919	0.277405	4.754484	1.99E-06	9.81E-06
CNAG_02658	1845.084	-0.15916	0.080589	-1.97496	0.048273	0.090959
CNAG_02657	8262.559	-0.22984	0.063887	-3.59764	0.000321	0.001058
CNAG_02656	3018.164	-0.17377	0.073847	-2.35311	0.018617	0.039821
CNAG_02655	1649.271	-0.50211	0.081408	-6.16783	6.92E-10	5.16E-09
CNAG_02654	1604.475	0.418671	0.086549	4.837394	1.32E-06	6.62E-06
CNAG_07529	1659.513	-0.17519	0.082112	-2.13361	0.032875	0.0653
CNAG_07530	1943.043	-0.19626	0.095364	-2.05798	0.039592	0.076748
CNAG_07531	728.1733	0.26156	0.094586	2.765327	0.005687	0.01401
CNAG_07532	968.5041	-0.25684	0.11754	-2.18514	0.028879	0.058434
CNAG_07533	120.6888	0.430129	0.218132	1.971872	0.048624	0.091464
CNAG_07538	1575.935	1.905783	0.122539	15.55246	1.53E-54	5.63E-53
CNAG_07539	2348.846	0.249471	0.065283	3.821355	0.000133	0.000473
CNAG_07540	334.5358	-0.37538	0.151983	-2.46988	0.013516	0.03018
CNAG_07541	1900.062	0.91284	0.075825	12.03884	2.22E-33	4.97E-32
CNAG_07542	221.95	0.890232	0.16172	5.504789	3.70E-08	2.28E-07
CNAG_12299	21.2287	3.975148	0.689994	5.76113	8.36E-09	5.55E-08
CNAG_07544	120.402	1.145788	0.21947	5.220705	1.78E-07	1.01E-06
CNAG_07545	1081.98	0.194521	0.085524	2.274453	0.022939	0.047782
CNAG_07547	1108.479	0.522035	0.110531	4.722969	2.32E-06	1.13E-05
CNAG_07548	1165.382	0.991488	0.083923	11.81427	3.29E-32	7.10E-31
CNAG_07549	423.9964	0.838444	0.133487	6.281101	3.36E-10	2.58E-09
CNAG_07550	641.4152	0.722107	0.099008	7.293382	3.02E-13	3.01E-12
CNAG_07552	1489.991	0.704496	0.081967	8.594876	8.34E-18	1.05E-16
CNAG_07553	1048.33	0.392637	0.085303	4.602876	4.17E-06	1.96E-05
CNAG_07554	4446.986	-0.1684	0.066162	-2.5453	0.010918	0.025027
CNAG_07108	2424.731	-0.17339	0.077441	-2.23894	0.02516	0.051798

CNAG_07555	396.8036	0.933767	0.134105	6.962965	3.33E-12	3.03E-11
CNAG_07556	900.8896	-0.35854	0.086666	-4.13703	3.52E-05	0.00014
CNAG_07557	816.4633	-0.91075	0.102497	-8.88568	6.35E-19	8.51E-18
CNAG_07558	17881.99	0.503108	0.069448	7.24443	4.34E-13	4.29E-12
CNAG_07561	47826.76	-0.13516	0.060218	-2.24446	0.024803	0.051188
CNAG_07565	541.9002	-0.44758	0.102693	-4.35845	1.31E-05	5.62E-05
CNAG_07566	487.6013	0.281268	0.110464	2.546244	0.010889	0.024966
CNAG_07571	1798.754	-0.23146	0.087123	-2.65669	0.007891	0.018705
CNAG_07572	1624.498	0.402372	0.086288	4.663115	3.11E-06	1.49E-05
CNAG_02609	1607.053	0.174366	0.076743	2.272067	0.023082	0.048032
CNAG_07573	792.9257	-0.37308	0.089153	-4.1847	2.86E-05	0.000115
CNAG_02605	844.2059	0.233637	0.094882	2.462392	0.013801	0.030725
CNAG_02604	90.67147	0.761912	0.228231	3.338346	0.000843	0.002528
CNAG_12302	200.5664	2.372431	0.18526	12.80597	1.52E-37	3.86E-36
CNAG_02603	215.959	2.196834	0.173346	12.67309	8.34E-37	2.09E-35
CNAG_02602	193.1596	2.005705	0.174322	11.50573	1.23E-30	2.59E-29
CNAG_02600	417.9334	0.31704	0.115074	2.755086	0.005868	0.014374
CNAG_02599	38.19121	-0.78195	0.354284	-2.20714	0.027304	0.055705
CNAG_02598	173.2087	0.587566	0.198694	2.957138	0.003105	0.008168
CNAG_12303	29.92101	0.897252	0.426391	2.104295	0.035353	0.069458
CNAG_02595	412.5918	-0.33249	0.142503	-2.33323	0.019636	0.041737
CNAG_02594	353.0005	-0.33072	0.131262	-2.51952	0.011751	0.02668
CNAG_02593	719.084	-0.27541	0.114823	-2.39852	0.016461	0.035804
CNAG_02591	143.2009	-0.45852	0.230058	-1.99308	0.046252	0.087713
CNAG_02590	449.1127	1.300269	0.11469	11.33723	8.58E-30	1.76E-28
CNAG_02589	596.6411	0.382002	0.101675	3.757084	0.000172	0.000601
CNAG_02587	2085.141	1.497356	0.071469	20.95123	1.83E-97	1.28E-95
CNAG_02586	137.8576	2.040228	0.208811	9.770678	1.50E-22	2.38E-21
CNAG_02585	3062.216	0.540184	0.079289	6.812889	9.57E-12	8.51E-11
CNAG_12306	33.71509	-1.15278	0.399871	-2.88287	0.003941	0.010095
CNAG_02581	3553.66	1.455969	0.06795	21.4271	7.47E-102	5.57E-100
CNAG_02580	3772.633	1.578499	0.062006	25.45723	5.87E-143	7.12E-141
CNAG_02579	1574.616	0.463304	0.103324	4.483984	7.33E-06	3.32E-05
CNAG_02577	2787.046	-0.4297	0.085435	-5.02956	4.92E-07	2.64E-06
CNAG_02575	2703.577	-0.23901	0.066342	-3.60267	0.000315	0.00104
CNAG_02574	1261.597	0.165112	0.079481	2.077393	0.037765	0.073717
CNAG_12311	42.746	0.695745	0.336255	2.0691	0.038537	0.075024
CNAG_07574	99.36881	0.634227	0.229811	2.759771	0.005784	0.014212
CNAG_12313	153.319	0.562711	0.242789	2.317691	0.020466	0.043174
CNAG_12314	99.60187	2.827129	0.286713	9.860469	6.18E-23	9.96E-22
CNAG_02569	749.5491	-0.2655	0.095792	-2.77167	0.005577	0.013779
CNAG_02568	3326.95	-0.28999	0.090977	-3.18748	0.001435	0.004069
CNAG_02567	618.3446	-0.32114	0.120514	-2.66474	0.007705	0.018333
CNAG_02566	2084.2	0.18245	0.088242	2.067625	0.038675	0.075254
CNAG_02565	7937.487	1.172001	0.060067	19.51158	8.75E-85	5.16E-83
CNAG_02564	1712.727	1.00186	0.078196	12.81221	1.40E-37	3.57E-36
CNAG_02563	1262.083	0.178936	0.079449	2.252204	0.024309	0.050276

CNAG_07972	202.3628	0.544642	0.159415	3.416505	0.000634	0.001957
CNAG_06879	632.7217	-0.35626	0.097791	-3.64311	0.000269	0.0009
CNAG_06880	907.5488	-0.2053	0.090281	-2.27404	0.022964	0.047797
CNAG_06881	1599.383	-0.19336	0.082306	-2.34921	0.018813	0.040203
CNAG_07576	561.1288	0.397733	0.115315	3.44911	0.000562	0.001756
CNAG_07579	596.3554	-0.26402	0.107274	-2.46117	0.013849	0.030805
CNAG_07580	1288.691	-0.25154	0.099545	-2.5269	0.011507	0.026185
CNAG_06887	464.6232	0.44928	0.111769	4.019708	5.83E-05	0.000223
CNAG_12318	34.25002	0.911617	0.368388	2.474613	0.013338	0.029815
CNAG_06891	1054.973	-0.52252	0.099694	-5.24119	1.60E-07	9.10E-07
CNAG_06892	1228.268	-0.27523	0.091321	-3.01388	0.002579	0.006917
CNAG_06893	1121.43	-0.54936	0.089529	-6.13619	8.45E-10	6.21E-09
CNAG_06896	1807.901	-0.19391	0.076762	-2.52609	0.011534	0.026231
CNAG_06897	1049.748	-0.69607	0.084123	-8.2744	1.29E-16	1.54E-15
CNAG_12319	35.97954	1.530299	0.360516	4.244747	2.19E-05	8.99E-05
CNAG_12322	17.75523	2.067403	0.562419	3.675913	0.000237	0.000803
CNAG_12323	97.24229	1.395041	0.247272	5.641717	1.68E-08	1.08E-07
CNAG_06898	2498.388	0.473068	0.069533	6.803514	1.02E-11	9.04E-11
CNAG_06899	6865.633	0.271353	0.07228	3.754207	0.000174	0.000607
CNAG_06900	24646.99	0.326523	0.067573	4.832138	1.35E-06	6.78E-06
CNAG_06901	651.9261	1.346073	0.113676	11.84129	2.39E-32	5.17E-31
CNAG_06902	1108.302	-0.44501	0.086733	-5.13078	2.89E-07	1.60E-06
CNAG_06903	913.9355	-0.27892	0.101084	-2.75926	0.005793	0.014226
CNAG_06905	1346.312	-0.82716	0.089084	-9.28508	1.62E-20	2.35E-19
CNAG_06906	11201.92	-0.30235	0.067624	-4.47106	7.78E-06	3.50E-05
CNAG_06908	12870.16	0.277174	0.062771	4.415634	1.01E-05	4.43E-05
CNAG_06909	639.7245	0.266439	0.099598	2.675154	0.007469	0.01783
CNAG_12328	157.1227	0.77452	0.182503	4.243868	2.20E-05	9.02E-05
CNAG_06910	503.3857	2.528741	0.122742	20.60206	2.63E-94	1.78E-92
CNAG_06911	8.749487	3.702141	0.974172	3.800295	0.000145	0.000512
CNAG_06912	358.764	0.769757	0.137957	5.579695	2.41E-08	1.51E-07
CNAG_06913	678.2259	1.716682	0.112697	15.23277	2.14E-52	7.67E-51
CNAG_06914	1579.045	-0.31531	0.073398	-4.29593	1.74E-05	7.30E-05
CNAG_06916	1462.174	0.448777	0.080948	5.544052	2.96E-08	1.84E-07
CNAG_12331	74.34948	0.952478	0.261655	3.640205	0.000272	0.000909
CNAG_06920	4145.38	-0.23002	0.090852	-2.53179	0.011348	0.025903
CNAG_06922	1377.784	-0.96084	0.270933	-3.54642	0.000391	0.001262
CNAG_06923	14457.53	2.099632	0.075563	27.78663	6.29E-170	9.47E-168
CNAG_06924	1106.951	-0.30592	0.128484	-2.38101	0.017265	0.037268
CNAG_06925	923.2477	-0.40717	0.09177	-4.43688	9.13E-06	4.05E-05
CNAG_06929	1640.382	-0.66822	0.075085	-8.8995	5.61E-19	7.55E-18
CNAG_06930	1942.762	-0.16123	0.073099	-2.2056	0.027412	0.055869
CNAG_06931	149.0415	-1.15425	0.182557	-6.32264	2.57E-10	2.00E-09
CNAG_06932	212.2935	0.871521	0.17595	4.95324	7.30E-07	3.82E-06
CNAG_07583	751.9705	0.366884	0.101472	3.615617	0.0003	0.000992
CNAG_06934	349.5647	-0.36148	0.135361	-2.67051	0.007574	0.018047
CNAG_12333	97.0693	0.570861	0.236044	2.418451	0.015587	0.034102

CNAG_06935	358.2044	1.541798	0.195488	7.886922	3.10E-15	3.45E-14
CNAG_07793	339.885	0.412586	0.14511	2.843255	0.004466	0.01129
CNAG_04932	489.9836	-0.39425	0.107721	-3.65991	0.000252	0.00085
CNAG_04933	135.9218	-0.46926	0.20322	-2.30911	0.020937	0.043997
CNAG_04936	2845.58	-0.31063	0.090385	-3.43679	0.000589	0.001832
CNAG_04937	382.3149	-0.30447	0.124731	-2.441	0.014647	0.032315
CNAG_07796	1534.995	0.963685	0.090387	10.6618	1.54E-26	2.77E-25
CNAG_04943	1513.661	0.574534	0.084458	6.80262	1.03E-11	9.09E-11
CNAG_04945	248.8406	-0.46418	0.168687	-2.75175	0.005928	0.014508
CNAG_04948	3475.145	-0.2436	0.06375	-3.82119	0.000133	0.000473
CNAG_04949	1178.045	-0.6393	0.107655	-5.93843	2.88E-09	2.01E-08
CNAG_04950	1880.662	-0.44589	0.085313	-5.22655	1.73E-07	9.82E-07
CNAG_04951	7956.066	1.072241	0.062626	17.12134	1.03E-65	4.86E-64
CNAG_04952	2272.483	0.171424	0.069151	2.478989	0.013176	0.029518
CNAG_12336	110.9508	-0.40836	0.207039	-1.97239	0.048565	0.091395
CNAG_04953	8751.313	-0.51448	0.067557	-7.6155	2.63E-14	2.79E-13
CNAG_04954	2758.315	0.204269	0.087213	2.342202	0.01917	0.040843
CNAG_04955	745.0789	-0.37713	0.128639	-2.9317	0.003371	0.008791
CNAG_04959	992.3606	-0.23633	0.100638	-2.3483	0.018859	0.040286
CNAG_04960	528.0498	2.619765	0.169112	15.49127	3.97E-54	1.45E-52
CNAG_04962	4259.113	-0.18985	0.07428	-2.55589	0.010592	0.024333
CNAG_12339	147.228	0.425416	0.206885	2.056295	0.039754	0.077025
CNAG_04963	5423.409	0.555034	0.082344	6.740435	1.58E-11	1.37E-10
CNAG_07797	3647.622	1.9583	0.06424	30.48413	4.23E-204	8.82E-202
CNAG_07798	424.7204	3.909511	0.156448	24.98921	8.01E-138	8.80E-136
CNAG_04967	393.7943	0.586788	0.11819	4.964793	6.88E-07	3.61E-06
CNAG_04968	732.7209	0.968993	0.094613	10.2416	1.29E-24	2.22E-23
CNAG_04969	12984.26	-0.22961	0.060808	-3.77595	0.000159	0.000562
CNAG_07800	879.907	-0.2005	0.091771	-2.18477	0.028906	0.058473
CNAG_04973	2499.694	0.293119	0.074437	3.937787	8.22E-05	0.000307
CNAG_12342	41.54759	1.837355	0.352004	5.219696	1.79E-07	1.02E-06
CNAG_12345	81.89419	-0.57431	0.244912	-2.34497	0.019029	0.040584
CNAG_12346	266.5849	1.016817	0.195087	5.212121	1.87E-07	1.06E-06
CNAG_04981	5124.736	1.360711	0.216356	6.289235	3.19E-10	2.46E-09
CNAG_04982	280.4606	1.48055	0.14788	10.01184	1.35E-23	2.24E-22
CNAG_04983	1359.899	0.452238	0.075167	6.016442	1.78E-09	1.27E-08
CNAG_04985	15040.49	-0.32052	0.081623	-3.92681	8.61E-05	0.00032
CNAG_12348	64.20114	1.070315	0.295001	3.628178	0.000285	0.00095
CNAG_12349	48.59229	2.647918	0.348027	7.608366	2.78E-14	2.94E-13
CNAG_04988	201.5274	4.506704	0.248518	18.13431	1.71E-73	8.84E-72
CNAG_12350	213.6776	1.783522	0.195966	9.101175	8.94E-20	1.25E-18
CNAG_04989	725.0531	-0.41136	0.102397	-4.01734	5.89E-05	0.000225
CNAG_04991	2065.11	-0.28318	0.084529	-3.35008	0.000808	0.002433
CNAG_12354	24.22724	1.45634	0.434496	3.351795	0.000803	0.002421
CNAG_04993	47.31311	-1.00094	0.324114	-3.08825	0.002013	0.005542
CNAG_04994	1019.132	-0.35779	0.107489	-3.32866	0.000873	0.002607
CNAG_07801	2105.491	-0.47128	0.069705	-6.76102	1.37E-11	1.19E-10

CNAG_07802	888.0694	4.756849	0.323808	14.69032	7.44E-49	2.45E-47
CNAG_04997	1289.079	0.391217	0.08451	4.629243	3.67E-06	1.74E-05
CNAG_04999	278.7704	-0.44261	0.138802	-3.18876	0.001429	0.004054
CNAG_05002	19.90533	1.034946	0.493398	2.097587	0.035942	0.070479
CNAG_05003	955.2489	1.249398	0.090779	13.76309	4.25E-43	1.28E-41
CNAG_05004	1405.703	0.371134	0.076786	4.833329	1.34E-06	6.74E-06
CNAG_05005	550.0823	0.426923	0.111946	3.813643	0.000137	0.000487
CNAG_05011	803.5507	-0.20727	0.093244	-2.22285	0.026226	0.053774
CNAG_05012	671.0477	0.341175	0.096738	3.526785	0.000421	0.00135
CNAG_05013	9137.499	-0.30488	0.072458	-4.20762	2.58E-05	0.000105
CNAG_05015	335.0409	1.893688	0.131301	14.42249	3.74E-47	1.20E-45
CNAG_05016	32.448	1.771368	0.403398	4.39112	1.13E-05	4.90E-05
CNAG_05017	719.6035	1.248052	0.09502	13.13465	2.08E-39	5.59E-38
CNAG_05018	146.9048	1.124596	0.206462	5.446981	5.12E-08	3.11E-07
CNAG_05020	326.5855	0.908408	0.12959	7.009844	2.39E-12	2.21E-11
CNAG_05023	907.7706	-0.66486	0.088613	-7.50298	6.24E-14	6.49E-13
CNAG_05024	256.4629	0.525251	0.143631	3.656941	0.000255	0.000857
CNAG_05028	4161.375	-0.40561	0.065159	-6.22498	4.82E-10	3.63E-09
CNAG_05030	934.0634	-0.23955	0.092715	-2.58369	0.009775	0.022635
CNAG_05031	2219.891	1.283286	0.105416	12.17354	4.30E-34	9.90E-33
CNAG_05032	949.1459	3.74759	0.287351	13.04187	7.07E-39	1.87E-37
CNAG_05033	2213.652	-0.1753	0.075462	-2.32295	0.020182	0.042718
CNAG_05036	1827.725	0.38678	0.074252	5.209032	1.90E-07	1.07E-06
CNAG_05038	406.5965	0.339821	0.123014	2.762448	0.005737	0.014126
CNAG_05039	802.35	-0.31566	0.104967	-3.00725	0.002636	0.007053
CNAG_05041	10220.64	0.982959	0.066609	14.75718	2.77E-49	9.21E-48
CNAG_05043	1263.938	-0.40706	0.092246	-4.41275	1.02E-05	4.48E-05
CNAG_07805	4517.346	0.231309	0.071282	3.244976	0.001175	0.0034
CNAG_07807	25738.57	-1.10582	0.084811	-13.0386	7.38E-39	1.94E-37
CNAG_05053	662.6556	-0.35686	0.108355	-3.29343	0.00099	0.002916
CNAG_05057	1311.571	0.441489	0.096174	4.590506	4.42E-06	2.07E-05
CNAG_05059	12107.97	-0.43815	0.079908	-5.48317	4.18E-08	2.55E-07
CNAG_05060	870.4215	-0.49555	0.100222	-4.94453	7.63E-07	3.98E-06
CNAG_05061	65.32604	-1.36737	0.308108	-4.43795	9.08E-06	4.03E-05
CNAG_12361	55.31546	-0.96071	0.292071	-3.28931	0.001004	0.002953
CNAG_05067	51.39233	0.901125	0.324796	2.774434	0.00553	0.013681
CNAG_05068	7225.774	-0.27472	0.071315	-3.85223	0.000117	0.000423
CNAG_05069	5579.525	0.835188	0.096159	8.685443	3.77E-18	4.88E-17
CNAG_12362	20.45336	2.324042	0.512804	4.532031	5.84E-06	2.69E-05
CNAG_05070	14629.43	0.68106	0.058527	11.63669	2.68E-31	5.68E-30
CNAG_05072	11.54336	-1.76605	0.680793	-2.5941	0.009484	0.022056
CNAG_07808	3010.839	0.705937	0.084193	8.384742	5.08E-17	6.23E-16
CNAG_05077	2015.802	0.169181	0.085403	1.980964	0.047595	0.089945
CNAG_05079	73.28773	-2.07735	0.322044	-6.45052	1.11E-10	8.96E-10
CNAG_05082	1383.624	-0.17808	0.076448	-2.32948	0.019834	0.042069
CNAG_05083	2436.412	-0.35526	0.072617	-4.89228	9.97E-07	5.11E-06
CNAG_05085	2140.562	0.786827	0.070753	11.12082	9.94E-29	1.94E-27

CNAG_05086	1152.817	0.87687	0.098234	8.926363	4.40E-19	5.95E-18
CNAG_07810	9738.756	-0.32224	0.079066	-4.07559	4.59E-05	0.000178
CNAG_05090	2380.403	0.442873	0.086706	5.107756	3.26E-07	1.79E-06
CNAG_05091	1808.589	-0.30853	0.073113	-4.2199	2.44E-05	9.92E-05
CNAG_05095	1569.343	-0.40495	0.127725	-3.17048	0.001522	0.004292
CNAG_05096	1292.447	-0.19151	0.08477	-2.2592	0.023871	0.049457
CNAG_05097	1177.917	-0.40455	0.103374	-3.91349	9.10E-05	0.000337
CNAG_05098	1216.253	0.190289	0.085742	2.219317	0.026465	0.054184
CNAG_05105	9325.032	-0.20987	0.06399	-3.27967	0.001039	0.003045
CNAG_05109	4779.12	0.527276	0.063888	8.253106	1.54E-16	1.84E-15
CNAG_05110	1680.742	-0.2185	0.111369	-1.96198	0.049764	0.093264
CNAG_05111	3080.163	-0.321	0.081008	-3.96263	7.41E-05	0.000279
CNAG_12368	143.3247	1.552887	0.211247	7.351061	1.97E-13	1.98E-12
CNAG_05112	2301.202	1.006007	0.076467	13.15608	1.57E-39	4.25E-38
CNAG_05113	1117.493	0.312703	0.079913	3.913029	9.11E-05	0.000337
CNAG_05114	256.7402	2.369697	0.159936	14.81657	1.14E-49	3.86E-48
CNAG_05115	1606.862	1.110322	0.074678	14.86803	5.32E-50	1.82E-48
CNAG_12369	27.75856	2.055094	0.478831	4.291901	1.77E-05	7.43E-05
CNAG_05117	4567.603	0.253958	0.067696	3.751465	0.000176	0.000612
CNAG_05118	1355.787	0.388879	0.089159	4.361645	1.29E-05	5.55E-05
CNAG_05119	575.8568	1.274026	0.136506	9.333129	1.03E-20	1.51E-19
CNAG_05120	2100.934	-0.4123	0.077203	-5.34055	9.27E-08	5.47E-07
CNAG_05121	2730.212	0.291094	0.082601	3.524088	0.000425	0.001363
CNAG_05123	504.3681	-0.49683	0.113761	-4.3673	1.26E-05	5.41E-05
CNAG_05124	2296.328	-0.28153	0.080055	-3.51664	0.000437	0.001397
CNAG_05125	2716.751	-0.47431	0.092792	-5.11158	3.19E-07	1.75E-06
CNAG_07814	988.4651	-0.43556	0.091771	-4.74614	2.07E-06	1.02E-05
CNAG_05130	1544.575	-1.23167	0.098751	-12.4726	1.05E-35	2.53E-34
CNAG_05132	11617.38	0.462987	0.072879	6.352804	2.11E-10	1.66E-09
CNAG_05131	376.9968	-0.41223	0.117419	-3.51071	0.000447	0.001425
CNAG_05134	1169.327	-0.30972	0.083713	-3.69985	0.000216	0.000736
CNAG_05136	6004.101	-0.13809	0.069273	-1.99348	0.046209	0.087651
CNAG_05137	1755.779	-0.2048	0.102592	-1.99626	0.045905	0.087198
CNAG_05138	3125.271	1.110811	0.080524	13.79471	2.74E-43	8.32E-42
CNAG_12370	18.05912	2.004128	0.669131	2.995123	0.002743	0.007318
CNAG_05140	10255.64	0.12173	0.057355	2.122406	0.033804	0.066834
CNAG_05141	1712.432	0.579137	0.075357	7.685225	1.53E-14	1.64E-13
CNAG_05144	11230.34	-0.44646	0.079205	-5.63678	1.73E-08	1.11E-07
CNAG_05147	219.1772	0.821716	0.193004	4.257499	2.07E-05	8.55E-05
CNAG_05149	1119.569	0.236133	0.086694	2.723748	0.006455	0.015642
CNAG_05150	2973.953	0.356125	0.063554	5.6035	2.10E-08	1.33E-07
CNAG_05153	1216.98	0.181734	0.092317	1.968587	0.049001	0.092066
CNAG_05154	7007.322	1.851586	0.062938	29.41943	3.10E-190	5.86E-188
CNAG_12373	6.681207	1.78673	0.894214	1.998102	0.045706	0.0869
CNAG_05157	143.3155	-0.50479	0.182127	-2.77164	0.005577	0.013779
CNAG_12377	149.6482	0.567683	0.254114	2.23397	0.025485	0.052388
CNAG_05159	122.584	-0.53072	0.244078	-2.17441	0.029675	0.05982

CNAG_05161	137.2744	-1.79375	0.213288	-8.41	4.10E-17	5.05E-16
CNAG_05163	601.4705	0.327541	0.107829	3.037595	0.002385	0.006448
CNAG_05164	2271.49	-0.15869	0.071227	-2.22794	0.025885	0.053143
CNAG_05166	4634.123	-0.39392	0.069023	-5.707	1.15E-08	7.49E-08
CNAG_05167	2328.707	-0.52594	0.117605	-4.47209	7.75E-06	3.49E-05
CNAG_12380	17.99518	1.428376	0.520617	2.743622	0.006077	0.014827
CNAG_05169	3110.038	2.702743	0.109397	24.70572	9.28E-135	9.80E-133
CNAG_12381	69.96935	2.084069	0.34994	5.955495	2.59E-09	1.82E-08
CNAG_05170	1033.9	0.596352	0.089463	6.66589	2.63E-11	2.23E-10
CNAG_05171	1422.345	-0.4661	0.089883	-5.18568	2.15E-07	1.20E-06
CNAG_05177	1456.422	0.227718	0.086574	2.630328	0.00853	0.020074
CNAG_05179	17994.69	0.464056	0.060751	7.638688	2.19E-14	2.34E-13
CNAG_07974	626.1113	1.11659	0.104539	10.68106	1.25E-26	2.26E-25
CNAG_05180	510.1348	0.501982	0.1104	4.546924	5.44E-06	2.52E-05
CNAG_12383	16.96595	1.114616	0.527643	2.112444	0.034648	0.068239
CNAG_05181	811.1847	0.349849	0.097495	3.588381	0.000333	0.001094
CNAG_05182	811.9484	-0.37707	0.098427	-3.83097	0.000128	0.000457
CNAG_05185	156.3645	-0.58834	0.211815	-2.7776	0.005476	0.013566
CNAG_05186	499.7637	-0.35836	0.127335	-2.81429	0.004889	0.012241
CNAG_05187	1602.931	0.361433	0.073595	4.911086	9.06E-07	4.67E-06
CNAG_05189	1387.132	0.288566	0.083663	3.449132	0.000562	0.001756
CNAG_05190	2652.407	0.322733	0.069991	4.611058	4.01E-06	1.89E-05
CNAG_05192	118.4266	0.477778	0.192558	2.481219	0.013093	0.029371
CNAG_05194	4024.229	1.301325	0.075339	17.27284	7.53E-67	3.64E-65
CNAG_05196	1398.305	-0.47529	0.102119	-4.65425	3.25E-06	1.55E-05
CNAG_05197	5626.599	0.403848	0.070613	5.719196	1.07E-08	7.00E-08
CNAG_05198	1090.519	0.455592	0.087778	5.190248	2.10E-07	1.18E-06
CNAG_05199	28688.81	0.568871	0.060934	9.335857	1.00E-20	1.48E-19
CNAG_05200	809.4788	0.197031	0.087657	2.247751	0.024592	0.050797
CNAG_05212	59.10329	-0.62568	0.270824	-2.31028	0.020873	0.043906
CNAG_05217	415.4146	-0.65667	0.127491	-5.15069	2.60E-07	1.44E-06
CNAG_05218	2932.12	-0.22938	0.072224	-3.17598	0.001493	0.004213
CNAG_05219	1735.686	-0.40949	0.07927	-5.16582	2.39E-07	1.33E-06
CNAG_05221	2675.099	-1.18651	0.075933	-15.6257	4.86E-55	1.83E-53
CNAG_05224	174.721	-0.5736	0.166136	-3.4526	0.000555	0.001737
CNAG_05226	1536.912	-0.2271	0.078464	-2.89428	0.0038	0.009784
CNAG_05229	570.6193	-1.53384	0.1166	-13.1547	1.60E-39	4.32E-38
CNAG_05230	683.7068	-0.30471	0.123929	-2.45878	0.013941	0.03096
CNAG_05231	1700.637	-0.50361	0.086937	-5.79278	6.92E-09	4.65E-08
CNAG_05232	83884.47	-0.34219	0.069452	-4.92708	8.35E-07	4.32E-06
CNAG_05234	3295.855	0.20499	0.095432	2.148012	0.031713	0.063367
CNAG_05235	22620.91	-0.19384	0.067958	-2.85237	0.004339	0.011026
CNAG_05236	1697.382	-0.52071	0.076757	-6.78391	1.17E-11	1.02E-10
CNAG_05241	1581.134	-0.1829	0.076462	-2.39207	0.016754	0.036351
CNAG_05242	4061.156	-0.12499	0.063657	-1.96343	0.049596	0.093034
CNAG_07975	334.1651	1.440868	0.176762	8.151478	3.60E-16	4.21E-15
CNAG_05245	334.1354	-0.31375	0.158461	-1.98	0.047703	0.090065

CNAG_05248	3066.916	-0.17159	0.065968	-2.6011	0.009292	0.02171
CNAG_07822	653.6228	-0.42077	0.105599	-3.98463	6.76E-05	0.000256
CNAG_12389	171.9461	2.348205	0.238384	9.850507	6.82E-23	1.10E-21
CNAG_05251	796.9937	1.629703	0.101504	16.05561	5.22E-58	2.09E-56
CNAG_05252	2431.576	0.97652	0.066849	14.60791	2.50E-48	8.16E-47
CNAG_05253	1080.852	1.693833	0.105026	16.12769	1.63E-58	6.66E-57
CNAG_05256	2128.427	2.228433	0.092132	24.18741	3.02E-129	2.92E-127
CNAG_05258	369.8197	3.57619	0.167193	21.38959	1.67E-101	1.23E-99
CNAG_05259	99.38353	0.736059	0.249523	2.949857	0.003179	0.008346
CNAG_05262	3014.407	-0.33388	0.066045	-5.0553	4.30E-07	2.32E-06
CNAG_05264	366.7278	0.842401	0.390429	2.157631	0.030957	0.062035
CNAG_12392	95.38313	1.593755	0.311633	5.11421	3.15E-07	1.73E-06
CNAG_05266	135.2581	0.487034	0.187548	2.596848	0.009408	0.02193
CNAG_05267	3150.78	0.447121	0.073293	6.100419	1.06E-09	7.73E-09
CNAG_05268	65.76442	-0.85486	0.282433	-3.02678	0.002472	0.006657
CNAG_05270	2973.033	0.166399	0.067399	2.468862	0.013554	0.030242
CNAG_05273	2121.856	0.28265	0.087418	3.233301	0.001224	0.003524
CNAG_07824	447.3292	0.611747	0.127884	4.783608	1.72E-06	8.57E-06
CNAG_05276	1420.416	-0.18051	0.085853	-2.10253	0.035507	0.069693
CNAG_05277	3466.7	0.898932	0.068906	13.0457	6.72E-39	1.79E-37
CNAG_05278	61.7579	7.624976	1.052415	7.24522	4.32E-13	4.28E-12
CNAG_12395	21.96557	3.173725	0.591152	5.368714	7.93E-08	4.73E-07
CNAG_05279	2631.03	6.126184	0.249191	24.58428	1.86E-133	1.89E-131
CNAG_05280	1114.549	0.284893	0.080093	3.557051	0.000375	0.001218
CNAG_05282	1497.062	-0.31736	0.08947	-3.54716	0.000389	0.001258
CNAG_05283	1215.124	1.110832	0.083616	13.28498	2.83E-40	7.80E-39
CNAG_12396	12.93101	1.858902	0.685384	2.712206	0.006684	0.016139
CNAG_05288	804.1079	0.195837	0.095713	2.046091	0.040747	0.078669
CNAG_12400	38.51375	0.898263	0.376109	2.388308	0.016926	0.036648
CNAG_05290	719.1727	0.679484	0.126197	5.384296	7.27E-08	4.35E-07
CNAG_12401	13.92106	1.532528	0.605308	2.531817	0.011347	0.025903
CNAG_05291	1973.892	0.302427	0.080337	3.764455	0.000167	0.000585
CNAG_05292	4126.643	-0.48625	0.077514	-6.27304	3.54E-10	2.71E-09
CNAG_05293	2957.086	-0.82293	0.094977	-8.66453	4.53E-18	5.82E-17
CNAG_05297	402.3901	-0.31514	0.129897	-2.42609	0.015263	0.033483
CNAG_05298	436.5836	0.472016	0.117258	4.025431	5.69E-05	0.000218
CNAG_05299	251.3216	2.529455	0.249328	10.1451	3.48E-24	5.91E-23
CNAG_05300	624.4155	2.068554	0.116803	17.7097	3.53E-70	1.76E-68
CNAG_05301	3102.69	-0.1702	0.070222	-2.42379	0.01536	0.033669
CNAG_05302	1648.588	0.641128	0.081689	7.848433	4.21E-15	4.68E-14
CNAG_05303	151.0915	0.691016	0.184073	3.754033	0.000174	0.000607
CNAG_12402	32.41819	1.876859	0.488442	3.842544	0.000122	0.000439
CNAG_05306	758.6465	-0.29178	0.104849	-2.78284	0.005389	0.013382
CNAG_05307	1945.364	-0.17331	0.078894	-2.19681	0.028034	0.056979
CNAG_05309	2693.974	-0.61949	0.09393	-6.59524	4.25E-11	3.54E-10
CNAG_05310	62.6933	-1.20365	0.301628	-3.99051	6.59E-05	0.00025
CNAG_05311	7830.278	-0.20912	0.070558	-2.9638	0.003039	0.008007

CNAG_05312	3299.63	-0.53008	0.090541	-5.85462	4.78E-09	3.27E-08
CNAG_05313	1060.437	-0.2633	0.095757	-2.74963	0.005966	0.014593
CNAG_05315	291.9634	0.895501	0.169058	5.296994	1.18E-07	6.83E-07
CNAG_12405	204.7944	0.561534	0.170389	3.295591	0.000982	0.002897
CNAG_05316	1392.86	1.760264	0.092722	18.98439	2.30E-80	1.28E-78
CNAG_05317	744.2843	0.323216	0.098656	3.276176	0.001052	0.003076
CNAG_05318	870.0095	0.608821	0.096632	6.300397	2.97E-10	2.30E-09
CNAG_05320	563.85	0.87678	0.125773	6.971118	3.14E-12	2.87E-11
CNAG_12407	474.6261	1.103692	0.154485	7.144325	9.04E-13	8.66E-12
CNAG_05321	307.5637	1.021512	0.129011	7.918006	2.41E-15	2.72E-14
CNAG_07830	1862.322	2.128592	0.0757	28.11882	5.77E-174	9.57E-172
CNAG_05324	645.1815	4.513806	0.151582	29.77789	7.55E-195	1.46E-192
CNAG_05327	2061.017	0.325903	0.074185	4.393086	1.12E-05	4.86E-05
CNAG_07977	1475.823	1.211218	0.076701	15.79136	3.57E-56	1.37E-54
CNAG_05329	839.1827	5.311932	0.175415	30.28216	1.97E-201	4.00E-199
CNAG_05330	119.6345	4.965588	0.348919	14.23133	5.86E-46	1.84E-44
CNAG_05331	52.04084	0.960884	0.320577	2.997361	0.002723	0.007269
CNAG_06876	779.2441	1.368371	0.100185	13.65848	1.80E-42	5.20E-41
CNAG_06875	609.493	0.333635	0.097782	3.412033	0.000645	0.001986
CNAG_06874	550.5545	-0.38057	0.122542	-3.10562	0.001899	0.005266
CNAG_06873	110.8584	1.491463	0.230971	6.457364	1.07E-10	8.59E-10
CNAG_12409	23.17785	-1.00846	0.442666	-2.27816	0.022717	0.047369
CNAG_06871	1359.419	-0.28467	0.097255	-2.92705	0.003422	0.008912
CNAG_12411	19.48531	0.966965	0.484718	1.994901	0.046054	0.087432
CNAG_06867	727.372	0.397621	0.130497	3.046965	0.002312	0.006269
CNAG_06864	2532.572	-0.21576	0.089076	-2.42219	0.015427	0.033789
CNAG_06863	338.4678	-0.38995	0.133204	-2.92746	0.003417	0.008904
CNAG_12412	348.271	0.315261	0.147741	2.133879	0.032853	0.065272
CNAG_06853	326.0104	1.118203	0.145059	7.708609	1.27E-14	1.37E-13
CNAG_07400	10186.01	-0.21976	0.069463	-3.16376	0.001557	0.004386
CNAG_06849	4344.826	-0.59848	0.06547	-9.14126	6.17E-20	8.71E-19
CNAG_07401	177.0753	-0.42519	0.194878	-2.18182	0.029123	0.058839
CNAG_06846	382.6651	-0.33515	0.139111	-2.40923	0.015986	0.034845
CNAG_06845	750.5663	0.189469	0.092849	2.040624	0.041288	0.079543
CNAG_07403	911.792	-0.23172	0.093389	-2.48129	0.013091	0.029371
CNAG_06839	3383.295	-0.27282	0.065563	-4.16125	3.17E-05	0.000127
CNAG_06837	1278.167	-0.51672	0.09185	-5.62566	1.85E-08	1.18E-07
CNAG_06836	204.3801	1.704907	0.192043	8.877736	6.82E-19	9.11E-18
CNAG_12417	37.2923	1.75197	0.372295	4.705861	2.53E-06	1.23E-05
CNAG_06835	794.9398	1.234079	0.135537	9.105138	8.62E-20	1.21E-18
CNAG_06834	3261.638	0.643057	0.078762	8.164613	3.22E-16	3.79E-15
CNAG_06833	27.9277	1.403582	0.456817	3.072523	0.002123	0.005801
CNAG_06832	920.3238	-0.95112	0.096367	-9.86972	5.63E-23	9.12E-22
CNAG_06829	2553.808	0.308911	0.068702	4.496424	6.91E-06	3.14E-05
CNAG_06828	2575.363	1.427275	0.075482	18.90892	9.63E-80	5.33E-78
CNAG_06827	49.42285	1.041851	0.3452	3.018111	0.002544	0.006832
CNAG_06821	2895.425	1.551905	0.064507	24.05797	6.89E-128	6.51E-126

CNAG_06820	1439.494	0.509245	0.074755	6.812211	9.61E-12	8.54E-11
CNAG_06819	2807.721	0.345364	0.079771	4.329446	1.49E-05	6.36E-05
CNAG_07404	115.385	0.801751	0.247383	3.240925	0.001191	0.003439
CNAG_07405	450.8453	2.085257	0.124001	16.81646	1.85E-63	8.40E-62
CNAG_06818	1035.31	2.050663	0.090748	22.59727	4.61E-113	3.86E-111
CNAG_06817	1193.794	3.065309	0.098589	31.09195	3.09E-212	7.18E-210
CNAG_06816	340.0522	1.070711	0.124717	8.585135	9.07E-18	1.14E-16
CNAG_12420	252.9743	2.547229	0.181866	14.00606	1.43E-44	4.42E-43
CNAG_06815	125.4043	1.334185	0.225921	5.905546	3.51E-09	2.44E-08
CNAG_06814	478.9307	1.496282	0.115648	12.93826	2.74E-38	7.13E-37
CNAG_12421	190.4192	-1.26329	0.1755	-7.19823	6.10E-13	5.93E-12
CNAG_06809	1241.476	-0.38262	0.077268	-4.95181	7.35E-07	3.84E-06
CNAG_06808	605.9115	1.228807	0.121677	10.0989	5.59E-24	9.38E-23
CNAG_06805	33.0125	1.917873	0.401044	4.782198	1.73E-06	8.62E-06
CNAG_06804	1380.488	0.352979	0.083232	4.240922	2.23E-05	9.13E-05
CNAG_07406	54.93966	0.968245	0.393585	2.460067	0.013891	0.030883
CNAG_07407	71.61637	0.79104	0.299317	2.642813	0.008222	0.019421
CNAG_06971	4050.666	0.159789	0.062603	2.55239	0.010699	0.024572
CNAG_07408	2062.916	-0.25742	0.069533	-3.7021	0.000214	0.000731
CNAG_07409	3660.77	0.271444	0.068006	3.991477	6.57E-05	0.000249
CNAG_07005	576.1913	-0.43309	0.116119	-3.72967	0.000192	0.000663
CNAG_07004	18224.74	-0.33796	0.063394	-5.33116	9.76E-08	5.73E-07
CNAG_07410	4413.381	0.172776	0.060649	2.848803	0.004388	0.011133
CNAG_07015	1398.029	-0.25379	0.083981	-3.02199	0.002511	0.006752
CNAG_01457	1598.423	-0.39639	0.073737	-5.37577	7.63E-08	4.55E-07
CNAG_01456	29.17747	-1.83892	0.453971	-4.05075	5.11E-05	0.000197
CNAG_12428	57.94383	-1.08559	0.318963	-3.4035	0.000665	0.002042
CNAG_01454	3979.85	-0.13834	0.064345	-2.14995	0.031559	0.063094
CNAG_01451	1274.038	0.419753	0.127815	3.284057	0.001023	0.003002
CNAG_07414	731.8338	-0.37022	0.092076	-4.02075	5.80E-05	0.000222
CNAG_01446	3272.351	-0.81524	0.124593	-6.54327	6.02E-11	4.95E-10
CNAG_01445	381.0788	-0.47668	0.146904	-3.24484	0.001175	0.0034
CNAG_01444	921.4469	-0.43515	0.10566	-4.11836	3.82E-05	0.000151
CNAG_01443	826.2441	1.197111	0.104811	11.4216	3.26E-30	6.76E-29
CNAG_01442	2785.478	0.169416	0.078872	2.147986	0.031715	0.063367
CNAG_01441	1642.068	0.518177	0.097635	5.307309	1.11E-07	6.48E-07
CNAG_01440	367.6182	-0.4922	0.121569	-4.04867	5.15E-05	0.000199
CNAG_01439	2406.278	-0.2884	0.07476	-3.85773	0.000114	0.000414
CNAG_07415	2189.223	-0.38215	0.079042	-4.8348	1.33E-06	6.70E-06
CNAG_01431	447.9901	-0.27954	0.117409	-2.38089	0.017271	0.037268
CNAG_01430	1081.559	-0.27635	0.106715	-2.5896	0.009609	0.022289
CNAG_12433	27.66627	0.954041	0.447586	2.131524	0.033046	0.065592
CNAG_01426	2020.998	-0.43327	0.078936	-5.48888	4.05E-08	2.48E-07
CNAG_01425	211.7247	-0.49022	0.160176	-3.06051	0.00221	0.006017
CNAG_01424	909.5627	0.526314	0.109995	4.784903	1.71E-06	8.52E-06
CNAG_01423	3020.289	-0.15825	0.066677	-2.37342	0.017624	0.037967
CNAG_01422	1566.352	-0.27827	0.088003	-3.16202	0.001567	0.004411

CNAG_01420	874.3747	0.903063	0.089198	10.12426	4.31E-24	7.30E-23
CNAG_01419	1333.771	0.377807	0.092692	4.075959	4.58E-05	0.000178
CNAG_07416	1080.944	-0.29903	0.103088	-2.9007	0.003723	0.009604
CNAG_01418	1011.259	-0.43137	0.122413	-3.52389	0.000425	0.001364
CNAG_01416	1617.684	-0.21997	0.077936	-2.82239	0.004767	0.011962
CNAG_07418	819.1113	-0.36503	0.099912	-3.65355	0.000259	0.000867
CNAG_01408	1382.9	-0.37368	0.086311	-4.32941	1.50E-05	6.36E-05
CNAG_01405	1726.749	-0.49785	0.075989	-6.55166	5.69E-11	4.70E-10
CNAG_01402	3784.006	-0.17377	0.075923	-2.28872	0.022095	0.046275
CNAG_01400	13099.25	0.750417	0.057338	13.08753	3.88E-39	1.04E-37
CNAG_01399	1025.703	0.23153	0.097738	2.368878	0.017842	0.038375
CNAG_01395	2654.35	-0.29907	0.067322	-4.44244	8.89E-06	3.96E-05
CNAG_07419	610.634	-0.55483	0.111215	-4.9888	6.08E-07	3.21E-06
CNAG_01392	666.3458	-0.28657	0.109113	-2.62636	0.00863	0.020292
CNAG_01391	1423.612	-0.52469	0.084375	-6.21847	5.02E-10	3.79E-09
CNAG_01389	821.4383	-0.28803	0.096545	-2.98338	0.002851	0.00757
CNAG_01388	1141.011	-0.25767	0.091784	-2.8074	0.004994	0.012487
CNAG_01385	2055.247	0.222417	0.078767	2.82375	0.004747	0.011918
CNAG_07421	74.44494	-0.70718	0.272675	-2.59347	0.009501	0.022083
CNAG_01382	494.9979	-0.52339	0.106364	-4.92078	8.62E-07	4.46E-06
CNAG_01381	1436.649	-0.28948	0.08469	-3.41806	0.000631	0.001948
CNAG_01380	750.7387	-0.38477	0.102202	-3.76479	0.000167	0.000584
CNAG_01379	1225.438	0.22708	0.089143	2.547369	0.010854	0.0249
CNAG_01378	2213.198	-0.23949	0.068199	-3.5117	0.000445	0.00142
CNAG_01377	2717.208	-0.18283	0.067933	-2.69128	0.007118	0.017066
CNAG_01375	1400.126	0.245073	0.094319	2.598351	0.009367	0.021845
CNAG_01372	1690.558	-0.77771	0.091854	-8.46686	2.52E-17	3.13E-16
CNAG_01371	1163.959	-0.33067	0.082914	-3.98812	6.66E-05	0.000252
CNAG_01370	76.79685	-0.83865	0.252804	-3.31738	0.000909	0.002697
CNAG_12438	50.38505	-0.83896	0.326114	-2.5726	0.010094	0.023285
CNAG_01367	178.3791	0.992982	0.18146	5.47219	4.45E-08	2.71E-07
CNAG_01365	866.7227	-0.74102	0.097226	-7.6216	2.51E-14	2.67E-13
CNAG_01364	3479.36	-0.39721	0.074576	-5.32622	1.00E-07	5.87E-07
CNAG_01363	2093.251	0.209091	0.074841	2.793803	0.005209	0.01298
CNAG_01362	3084.091	-0.28146	0.068374	-4.11643	3.85E-05	0.000152
CNAG_01360	2232.745	0.341957	0.084497	4.04697	5.19E-05	0.0002
CNAG_01359	3117.308	-0.28212	0.079918	-3.53012	0.000415	0.001335
CNAG_01352	1892.656	-0.31462	0.089872	-3.50077	0.000464	0.001473
CNAG_01350	761.8542	-0.29976	0.096682	-3.10044	0.001932	0.005348
CNAG_01348	1021.243	-0.44395	0.132066	-3.36154	0.000775	0.002346
CNAG_01347	1962.954	-0.24271	0.072084	-3.36705	0.00076	0.002305
CNAG_01346	930.2224	-0.31072	0.085322	-3.64173	0.000271	0.000905
CNAG_12440	49.37505	0.594102	0.302739	1.962424	0.049713	0.093189
CNAG_01345	873.322	-0.33194	0.09276	-3.57842	0.000346	0.001132
CNAG_01344	1102.533	0.68156	0.114803	5.936774	2.91E-09	2.03E-08
CNAG_01343	717.3022	-0.19376	0.092907	-2.08554	0.03702	0.072401
CNAG_01342	857.3186	-0.38255	0.087477	-4.37314	1.22E-05	5.28E-05

CNAG_01340	3179.947	-0.1909	0.07114	-2.68345	0.007287	0.017435
CNAG_01338	647.4369	-0.48448	0.107178	-4.52034	6.17E-06	2.84E-05
CNAG_01336	120.0598	-0.74351	0.198226	-3.75084	0.000176	0.000613
CNAG_01335	887.6743	0.262348	0.089909	2.917933	0.003524	0.009144
CNAG_01333	1106.078	-0.18507	0.080228	-2.30674	0.021069	0.044262
CNAG_01332	40468.67	-0.23806	0.077465	-3.07309	0.002119	0.005792
CNAG_01331	429.3237	1.275925	0.122159	10.44477	1.55E-25	2.73E-24
CNAG_12443	254.4594	-0.60832	0.162863	-3.73517	0.000188	0.00065
CNAG_01324	434.7166	0.364342	0.120431	3.025315	0.002484	0.006685
CNAG_01323	13348.85	0.68163	0.075403	9.03982	1.57E-19	2.18E-18
CNAG_01321	9189.215	0.290437	0.074232	3.912561	9.13E-05	0.000338
CNAG_01319	1050.777	-0.31326	0.085339	-3.67081	0.000242	0.000818
CNAG_01317	1280.698	-0.51078	0.09856	-5.1824	2.19E-07	1.23E-06
CNAG_01316	1736.422	0.322372	0.072781	4.429331	9.45E-06	4.18E-05
CNAG_01314	1237.865	-0.56482	0.086024	-6.5658	5.18E-11	4.29E-10
CNAG_01313	630.5879	-0.30224	0.105304	-2.87015	0.004103	0.01048
CNAG_07427	11436.94	-0.19588	0.074228	-2.63895	0.008316	0.01961
CNAG_01310	1844.005	0.189684	0.076856	2.468052	0.013585	0.030302
CNAG_01307	2994.308	-0.72766	0.073912	-9.84494	7.21E-23	1.16E-21
CNAG_01306	557.6999	-0.52008	0.107784	-4.82517	1.40E-06	7.01E-06
CNAG_01305	6851.738	-0.20959	0.05918	-3.5416	0.000398	0.001281
CNAG_01302	1256.672	0.33551	0.096269	3.485119	0.000492	0.001552
CNAG_01301	698.3895	-0.41592	0.096159	-4.32536	1.52E-05	6.46E-05
CNAG_01299	175.7139	0.528005	0.214475	2.461851	0.013822	0.030763
CNAG_01295	1034.21	-0.71355	0.12137	-5.87914	4.12E-09	2.83E-08
CNAG_01293	1737.143	0.459354	0.080273	5.72239	1.05E-08	6.88E-08
CNAG_01289	299.9433	-0.34672	0.149567	-2.31812	0.020443	0.043135
CNAG_01288	1831.802	0.80116	0.071153	11.25968	2.08E-29	4.19E-28
CNAG_01287	23692.78	0.781389	0.054807	14.25699	4.06E-46	1.28E-44
CNAG_01286	272.7706	-0.29343	0.137822	-2.12903	0.033252	0.065951
CNAG_01285	1950.389	-0.1912	0.078348	-2.44045	0.014669	0.032355
CNAG_01284	2655.682	-0.14177	0.068574	-2.06745	0.038692	0.075254
CNAG_01283	2484.651	-0.24541	0.079901	-3.07142	0.00213	0.005817
CNAG_01282	1876.684	-0.18943	0.07462	-2.53867	0.011127	0.02547
CNAG_01281	576.469	-0.37843	0.107674	-3.51459	0.00044	0.001407
CNAG_01279	1564.592	-0.44801	0.077333	-5.79329	6.90E-09	4.64E-08
CNAG_07428	2530.841	0.416415	0.067931	6.129953	8.79E-10	6.45E-09
CNAG_01278	2492.259	0.531718	0.082536	6.442281	1.18E-10	9.43E-10
CNAG_01276	3893.991	0.446782	0.065079	6.865168	6.64E-12	5.94E-11
CNAG_01274	9016.198	-0.13409	0.063442	-2.11357	0.034552	0.068081
CNAG_01273	9680.944	-0.35977	0.065067	-5.52926	3.22E-08	2.00E-07
CNAG_01272	1446.564	2.629865	0.329001	7.993486	1.31E-15	1.50E-14
CNAG_01271	2763.873	-0.23543	0.080738	-2.91603	0.003545	0.009188
CNAG_01270	9444.939	-0.18063	0.059024	-3.06027	0.002211	0.006019
CNAG_01268	44.34688	0.772267	0.341297	2.262744	0.023651	0.049091
CNAG_01263	6158.607	0.36737	0.060804	6.041896	1.52E-09	1.09E-08
CNAG_01262	727.4044	-0.26572	0.125351	-2.11978	0.034024	0.067153

CNAG_07430	1544.572	0.302793	0.086794	3.488629	0.000486	0.001534
CNAG_06946	4045.942	0.733073	0.067648	10.83651	2.31E-27	4.35E-26
CNAG_06949	3565.539	-0.59263	0.083645	-7.08507	1.39E-12	1.31E-11
CNAG_07431	1497.128	0.331562	0.100419	3.30177	0.000961	0.002841
CNAG_01257	1540.966	0.728807	0.139977	5.20661	1.92E-07	1.08E-06
CNAG_01254	373.6897	-0.27793	0.128461	-2.16357	0.030497	0.061236
CNAG_01253	3931.575	-0.23031	0.067426	-3.41579	0.000636	0.001961
CNAG_01252	139.2392	-0.66864	0.184218	-3.62962	0.000284	0.000946
CNAG_01251	2823.317	0.34842	0.065375	5.329537	9.85E-08	5.77E-07
CNAG_01249	1176.658	0.358341	0.081923	4.374123	1.22E-05	5.26E-05
CNAG_01245	1393.129	-0.44635	0.090538	-4.93	8.22E-07	4.27E-06
CNAG_01244	96.9469	1.191573	0.266504	4.471131	7.78E-06	3.50E-05
CNAG_01243	2336.725	0.390031	0.067113	5.811553	6.19E-09	4.20E-08
CNAG_01242	3166.214	3.077547	0.07549	40.76755	0	0
CNAG_01241	1130.394	-0.29828	0.088586	-3.36718	0.000759	0.002305
CNAG_12453	240.5769	0.394423	0.158625	2.486511	0.0129	0.029013
CNAG_12454	11.03374	1.452886	0.677575	2.144243	0.032013	0.063823
CNAG_07981	108.0254	1.957868	0.235437	8.315881	9.11E-17	1.10E-15
CNAG_01237	442.8714	0.316192	0.113853	2.777189	0.005483	0.013579
CNAG_01235	7291.199	-0.19791	0.066888	-2.95888	0.003088	0.008132
CNAG_01233	801.3455	0.229337	0.091125	2.516717	0.011845	0.026871
CNAG_01232	1946.516	0.391454	0.076792	5.097624	3.44E-07	1.88E-06
CNAG_01231	813.0892	0.660369	0.098321	6.716452	1.86E-11	1.60E-10
CNAG_12456	58.09923	2.39526	0.326467	7.336902	2.19E-13	2.19E-12
CNAG_01230	17281.4	1.842432	0.073187	25.17437	7.65E-140	8.88E-138
CNAG_01229	3830.6	2.421271	0.112974	21.43204	6.72E-102	5.06E-100
CNAG_01228	800.7058	-0.68464	0.103317	-6.62663	3.43E-11	2.89E-10
CNAG_07432	252.5063	0.608779	0.156138	3.898973	9.66E-05	0.000355
CNAG_01224	57444.81	-0.15635	0.070883	-2.20567	0.027407	0.055869
CNAG_01223	542.4388	1.413264	0.160364	8.812855	1.22E-18	1.60E-17
CNAG_01222	2110.153	-0.52005	0.089703	-5.79749	6.73E-09	4.53E-08
CNAG_01217	2098.67	-0.25401	0.070841	-3.58556	0.000336	0.001103
CNAG_01214	356.304	0.578273	0.148532	3.893246	9.89E-05	0.000363
CNAG_01213	2554.216	0.169124	0.0661	2.558595	0.01051	0.024165
CNAG_01212	1197.908	0.325497	0.087493	3.72028	0.000199	0.000686
CNAG_01211	3635.135	-0.22246	0.06279	-3.54285	0.000396	0.001276
CNAG_01208	1303.139	0.184794	0.086712	2.131128	0.033079	0.065624
CNAG_07434	621.2933	-0.35499	0.108064	-3.28495	0.00102	0.002994
CNAG_01204	12968.52	-0.21226	0.087021	-2.43924	0.014718	0.032437
CNAG_01203	1229.082	0.313562	0.080868	3.877442	0.000106	0.000385
CNAG_01200	622.8057	-0.21435	0.108258	-1.98002	0.047701	0.090065
CNAG_01197	2586.471	0.159905	0.078197	2.044902	0.040865	0.078839
CNAG_01196	1461.846	-0.37252	0.075227	-4.95196	7.35E-07	3.84E-06
CNAG_01192	346.0905	-0.31058	0.125513	-2.47453	0.013341	0.029815
CNAG_01188	958.8408	-0.52404	0.096794	-5.41398	6.16E-08	3.72E-07
CNAG_01187	2712.875	0.193291	0.083151	2.324563	0.020095	0.042557
CNAG_01185	2425.607	-0.28046	0.0789	-3.55457	0.000379	0.001227

CNAG_01182	8740.112	-0.26949	0.057352	-4.69885	2.62E-06	1.27E-05
CNAG_01180	559.4952	0.397759	0.115521	3.443176	0.000575	0.001793
CNAG_01178	868.9376	-0.29942	0.107718	-2.77966	0.005442	0.013493
CNAG_07437	645.7539	-0.42838	0.107125	-3.99889	6.36E-05	0.000242
CNAG_01175	1170.028	-0.55385	0.082344	-6.72606	1.74E-11	1.50E-10
CNAG_01174	3683.143	0.522114	0.09428	5.537896	3.06E-08	1.90E-07
CNAG_01173	1295.94	-0.24734	0.095144	-2.59968	0.009331	0.021788
CNAG_01172	1795.519	-0.45121	0.070932	-6.36113	2.00E-10	1.58E-09
CNAG_01170	60488.08	-0.2647	0.068564	-3.86061	0.000113	0.000409
CNAG_01168	11464.79	-0.18784	0.062196	-3.0201	0.002527	0.006792
CNAG_01167	2296.103	0.200298	0.066776	2.999562	0.002704	0.007222
CNAG_01166	759.7753	-0.3548	0.095369	-3.72033	0.000199	0.000686
CNAG_01165	900.7655	-0.62257	0.121594	-5.12011	3.05E-07	1.68E-06
CNAG_01163	1071.395	0.485134	0.096451	5.029857	4.91E-07	2.63E-06
CNAG_01162	905.4934	-0.24634	0.08853	-2.78257	0.005393	0.013389
CNAG_07439	4338.571	0.123685	0.062166	1.989593	0.046636	0.088358
CNAG_01159	1933.835	-0.32445	0.097776	-3.31833	0.000906	0.002689
CNAG_01158	6399.104	-0.13744	0.064267	-2.13862	0.032466	0.064607
CNAG_01156	3125.706	-0.30074	0.063927	-4.7045	2.54E-06	1.23E-05
CNAG_01154	299.2748	0.451031	0.137135	3.288961	0.001006	0.002956
CNAG_01152	52378.57	-0.16518	0.066238	-2.4938	0.012638	0.028463
CNAG_01151	527.9899	-0.67316	0.114203	-5.8944	3.76E-09	2.60E-08
CNAG_01150	10043.38	0.175738	0.055157	3.18613	0.001442	0.004084
CNAG_01149	2102.857	0.282117	0.080503	3.504438	0.000458	0.001454
CNAG_01147	1672.685	-0.32912	0.083079	-3.96147	7.45E-05	0.00028
CNAG_01146	3970.788	0.20079	0.06144	3.268069	0.001083	0.003155
CNAG_01145	1176.094	-0.44703	0.115974	-3.85458	0.000116	0.000419
CNAG_01144	3335.934	0.304761	0.070864	4.300653	1.70E-05	7.16E-05
CNAG_01143	1067.549	0.243857	0.083568	2.918085	0.003522	0.009143
CNAG_01140	3822.884	0.289018	0.067638	4.272992	1.93E-05	8.01E-05
CNAG_12461	245.3238	1.914247	0.18483	10.35679	3.90E-25	6.83E-24
CNAG_01138	67505.67	3.841985	0.055919	68.70658	0	0
CNAG_01137	17753.72	1.50746	0.072946	20.66541	7.10E-95	4.89E-93
CNAG_01136	3448.257	-0.28536	0.113598	-2.51204	0.012004	0.02717
CNAG_01133	501.4316	0.275825	0.107408	2.568004	0.010229	0.023552
CNAG_01132	1811.254	0.189828	0.086077	2.205321	0.027432	0.055895
CNAG_01131	400.1403	0.480029	0.140433	3.418216	0.00063	0.001948
CNAG_01129	1711.851	-0.38779	0.082349	-4.70905	2.49E-06	1.21E-05
CNAG_01128	1390.035	-0.36525	0.082376	-4.43395	9.25E-06	4.10E-05
CNAG_01127	701.9703	-0.36283	0.097999	-3.70242	0.000214	0.000731
CNAG_01126	3548.529	0.201344	0.068178	2.953224	0.003145	0.008264
CNAG_01123	1510.977	-0.16653	0.075155	-2.21578	0.026706	0.05465
CNAG_01121	92.76694	0.580384	0.248006	2.340198	0.019274	0.041034
CNAG_01120	17100.03	-0.24343	0.058911	-4.13214	3.59E-05	0.000143
CNAG_12466	117.6591	0.554607	0.214051	2.591012	0.009569	0.022217
CNAG_01119	672.7811	0.342356	0.101066	3.38744	0.000705	0.002157
CNAG_01118	464.7329	2.074244	0.122767	16.89576	4.83E-64	2.21E-62

CNAG_12469	12.52967	2.135194	0.646831	3.301008	0.000963	0.002847
CNAG_01117	52523.4	0.132492	0.055494	2.387501	0.016963	0.036719
CNAG_01116	291.2001	-0.53271	0.133458	-3.99155	6.56E-05	0.000249
CNAG_01114	347.1643	-0.41592	0.151725	-2.74128	0.00612	0.014925
CNAG_01113	1554.553	-0.28664	0.092882	-3.08604	0.002028	0.005577
CNAG_01111	4578.916	-0.1917	0.065286	-2.9363	0.003322	0.008667
CNAG_12470	38.0379	1.206936	0.418422	2.884495	0.00392	0.010055
CNAG_12471	32.47593	2.182544	0.418137	5.219682	1.79E-07	1.02E-06
CNAG_01108	4363.617	3.07931	0.08568	35.93947	7.39E-283	3.34E-280
CNAG_01107	1767.089	-0.17253	0.076989	-2.24099	0.025027	0.051576
CNAG_01106	3434.355	-0.32113	0.07542	-4.25789	2.06E-05	8.54E-05
CNAG_01103	2361.144	-0.34044	0.068172	-4.99382	5.92E-07	3.13E-06
CNAG_01097	1353.131	-0.25897	0.092052	-2.81325	0.004904	0.012274
CNAG_01096	480.2473	-0.36763	0.11039	-3.33029	0.000868	0.002596
CNAG_01094	2169.378	0.169866	0.067227	2.52673	0.011513	0.02619
CNAG_01092	1922.535	-0.29269	0.075693	-3.86673	0.00011	0.0004
CNAG_01091	6658.76	-0.24248	0.059439	-4.07945	4.51E-05	0.000176
CNAG_01090	380.798	0.755625	0.148827	5.077201	3.83E-07	2.08E-06
CNAG_01089	1506.425	0.283463	0.075243	3.76731	0.000165	0.000579
CNAG_01082	2601.044	0.673825	0.081554	8.262324	1.43E-16	1.71E-15
CNAG_01081	382.9221	2.031832	0.170674	11.90476	1.12E-32	2.45E-31
CNAG_01080	233.8248	0.962316	0.152879	6.294643	3.08E-10	2.38E-09
CNAG_01079	81.43918	1.062399	0.239713	4.431967	9.34E-06	4.13E-05
CNAG_01078	526.7559	1.77733	0.146041	12.17007	4.49E-34	1.03E-32
CNAG_01077	118.1479	1.994959	0.213509	9.343697	9.30E-21	1.37E-19
CNAG_12476	17.06924	1.813566	0.561631	3.229107	0.001242	0.003565
CNAG_01076	557.0657	3.970526	0.149205	26.61117	5.04E-156	6.94E-154
CNAG_01075	1403.917	5.468708	0.141305	38.70146	0	0
CNAG_01074	92.60523	1.358058	0.237084	5.728172	1.02E-08	6.67E-08
CNAG_01073	594.9086	0.671135	0.109682	6.118931	9.42E-10	6.89E-09
CNAG_01072	428.8517	0.333808	0.132469	2.519892	0.011739	0.02666
CNAG_01070	33.59571	0.936632	0.372594	2.513814	0.011943	0.027071
CNAG_01069	630.6072	0.291517	0.104131	2.799508	0.005118	0.012768
CNAG_07442	3824.921	0.186556	0.069399	2.688176	0.007184	0.017215
CNAG_07443	66.65091	1.266167	0.285114	4.440911	8.96E-06	3.98E-05
CNAG_01065	345.1568	0.425005	0.126043	3.371907	0.000746	0.002273
CNAG_01064	244.4291	-0.64701	0.155008	-4.17404	2.99E-05	0.00012
CNAG_01063	3130.487	-0.16724	0.074196	-2.25403	0.024195	0.050077
CNAG_01062	1996.646	0.490165	0.068907	7.113444	1.13E-12	1.08E-11
CNAG_01061	617.1712	1.407611	0.121106	11.62299	3.15E-31	6.65E-30
CNAG_01060	8694.024	-0.61577	0.068176	-9.03213	1.68E-19	2.34E-18
CNAG_01059	314.2859	-0.30564	0.129677	-2.35691	0.018428	0.039447
CNAG_01058	1339.235	-0.23646	0.09809	-2.41066	0.015924	0.034727
CNAG_01057	1906.061	-0.33109	0.070548	-4.69316	2.69E-06	1.30E-05
CNAG_07444	253.7611	1.075165	0.20559	5.229661	1.70E-07	9.66E-07
CNAG_01055	12414.69	1.082171	0.065157	16.6087	6.03E-62	2.65E-60
CNAG_01054	799.4585	-0.23333	0.093831	-2.4867	0.012893	0.029006

CNAG_01051	2088.873	0.18884	0.073197	2.579899	0.009883	0.022866
CNAG_01049	1581.828	0.304936	0.096887	3.147337	0.001648	0.004618
CNAG_01048	150.5836	0.990971	0.221621	4.471469	7.77E-06	3.50E-05
CNAG_01047	170.6498	-0.4442	0.217393	-2.0433	0.041023	0.079069
CNAG_01046	1991.05	-0.43631	0.078488	-5.55901	2.71E-08	1.70E-07
CNAG_01044	1543.289	0.911595	0.103098	8.84205	9.40E-19	1.24E-17
CNAG_01043	6539.098	-0.99375	0.078696	-12.6277	1.48E-36	3.70E-35
CNAG_01042	643.7699	-1.31387	0.106156	-12.3768	3.49E-35	8.25E-34
CNAG_01041	220.7664	-0.92296	0.154706	-5.9659	2.43E-09	1.72E-08
CNAG_01040	134.9821	1.493442	0.213882	6.98255	2.90E-12	2.66E-11
CNAG_12485	34.81236	0.902876	0.387532	2.329808	0.019816	0.042043
CNAG_01038	773.6461	-0.23682	0.097583	-2.42686	0.01523	0.033432
CNAG_01036	487.659	-0.41735	0.11329	-3.68389	0.00023	0.00078
CNAG_01034	112.8403	0.44861	0.213035	2.105811	0.035221	0.069232
CNAG_01032	376.6367	0.471888	0.133158	3.543808	0.000394	0.001273
CNAG_01031	1937.739	-0.70575	0.304369	-2.31875	0.020409	0.043075
CNAG_01029	1483.98	-0.41512	0.104679	-3.96566	7.32E-05	0.000276
CNAG_01028	463.1188	-0.66166	0.11562	-5.72278	1.05E-08	6.87E-08
CNAG_01025	1006.796	-0.64088	0.10317	-6.21187	5.24E-10	3.93E-09
CNAG_01023	1229.798	-0.37417	0.0914	-4.09379	4.24E-05	0.000166
CNAG_01021	3915.744	-0.4384	0.069202	-6.33513	2.37E-10	1.86E-09
CNAG_01020	1468.999	-0.33015	0.080759	-4.08805	4.35E-05	0.00017
CNAG_01019	22425.22	-1.16815	0.079411	-14.7102	5.54E-49	1.83E-47
CNAG_01018	6977.898	-0.12935	0.065565	-1.97287	0.048511	0.091335
CNAG_01015	786.4554	-0.22912	0.102912	-2.2264	0.025987	0.053313
CNAG_01014	1719.407	0.385414	0.117637	3.276303	0.001052	0.003076
CNAG_01013	939.7223	0.182314	0.09072	2.009634	0.04447	0.084922
CNAG_01010	1908.597	-0.65357	0.06915	-9.45144	3.34E-21	5.05E-20
CNAG_01009	989.5335	-0.36198	0.09509	-3.80672	0.000141	0.0005
CNAG_01008	593.4608	-0.39853	0.136631	-2.91686	0.003536	0.009167
CNAG_01006	382.3441	-0.2666	0.129598	-2.0571	0.039677	0.076894
CNAG_01004	2283.246	-0.39546	0.084421	-4.68444	2.81E-06	1.35E-05
CNAG_01002	548.6005	-0.55858	0.112797	-4.95213	7.34E-07	3.83E-06
CNAG_01001	365.3954	0.285095	0.134458	2.120325	0.033979	0.067082
CNAG_00999	1693.989	1.15991	0.071704	16.17631	7.41E-59	3.07E-57
CNAG_00997	1234.693	0.760202	0.107092	7.098578	1.26E-12	1.19E-11
CNAG_00994	1900.965	-0.39645	0.083663	-4.73869	2.15E-06	1.06E-05
CNAG_00993	699.4328	-0.52629	0.099456	-5.29169	1.21E-07	7.03E-07
CNAG_00992	15998.33	0.310512	0.058325	5.323819	1.02E-07	5.94E-07
CNAG_00989	900.4566	-0.44	0.101115	-4.35145	1.35E-05	5.79E-05
CNAG_00988	5173.49	0.257751	0.073955	3.485258	0.000492	0.001552
CNAG_00987	1362.664	-0.56943	0.079027	-7.20545	5.79E-13	5.63E-12
CNAG_00986	1046.844	-0.23944	0.086882	-2.75589	0.005853	0.014351
CNAG_00982	1829.457	-0.34194	0.090044	-3.79744	0.000146	0.000518
CNAG_12487	28.27434	-0.91108	0.424702	-2.14522	0.031935	0.063698
CNAG_12488	113.1367	-1.02769	0.207338	-4.95658	7.17E-07	3.76E-06
CNAG_00979	346.7778	0.490353	0.193793	2.530292	0.011397	0.025991

CNAG_00978	5868.876	1.045342	0.059979	17.42853	5.01E-68	2.44E-66
CNAG_00977	1969.13	0.352553	0.076464	4.610682	4.01E-06	1.89E-05
CNAG_00976	7551.328	-0.33929	0.061209	-5.54319	2.97E-08	1.85E-07
CNAG_00972	5461.195	1.036695	0.075969	13.64629	2.12E-42	6.10E-41
CNAG_00971	356.079	-0.48115	0.131648	-3.65479	0.000257	0.000863
CNAG_00970	3732.659	-0.13032	0.061704	-2.11204	0.034683	0.068274
CNAG_00969	189.8661	-0.83391	0.158952	-5.24632	1.55E-07	8.88E-07
CNAG_00966	2276.502	0.257238	0.078595	3.272954	0.001064	0.003109
CNAG_00964	375.3805	0.76054	0.133365	5.702675	1.18E-08	7.68E-08
CNAG_07446	1454.971	-0.16835	0.081058	-2.07692	0.037809	0.073766
CNAG_00961	852.8509	-0.48591	0.163676	-2.96874	0.00299	0.007889
CNAG_00960	3647.439	-0.14921	0.063355	-2.3552	0.018513	0.039619
CNAG_12491	51.63093	0.905416	0.451315	2.006173	0.044838	0.08551
CNAG_12492	382.3648	-0.30476	0.138394	-2.2021	0.027658	0.0563
CNAG_00941	113.2531	-0.74375	0.207346	-3.58701	0.000334	0.001099
CNAG_00940	2989.848	-0.3442	0.068676	-5.01191	5.39E-07	2.87E-06
CNAG_00936	1584.873	1.109779	0.30659	3.619748	0.000295	0.000978
CNAG_00935	10297.12	-0.59675	0.083566	-7.14105	9.26E-13	8.86E-12
CNAG_00934	107.0924	1.22233	0.24625	4.963776	6.91E-07	3.63E-06
CNAG_00930	13857.36	-0.14461	0.058485	-2.4726	0.013413	0.029968
CNAG_00928	780.6188	0.330392	0.097923	3.374006	0.000741	0.002259
CNAG_00925	308.6275	0.6155	0.134863	4.563892	5.02E-06	2.33E-05
CNAG_00923	487.9718	0.576877	0.114618	5.033048	4.83E-07	2.59E-06
CNAG_00916	1950.661	-0.58631	0.071966	-8.14705	3.73E-16	4.36E-15
CNAG_00915	266.2126	0.823741	0.195008	4.224143	2.40E-05	9.76E-05
CNAG_00914	6039.559	0.410361	0.071429	5.745047	9.19E-09	6.07E-08
CNAG_07448	565.9349	0.699716	0.13331	5.248788	1.53E-07	8.78E-07
CNAG_12499	151.8747	3.415096	0.243235	14.04029	8.83E-45	2.75E-43
CNAG_07449	195.367	0.719312	0.161452	4.455257	8.38E-06	3.75E-05
CNAG_07450	172.0367	0.385859	0.16902	2.282915	0.022435	0.04683
CNAG_07982	2389.557	0.656605	0.068093	9.642707	5.28E-22	8.19E-21
CNAG_00909	936.7265	0.994861	0.101425	9.808833	1.03E-22	1.65E-21
CNAG_00908	29.81876	0.88906	0.446994	1.988975	0.046704	0.088466
CNAG_00907	130.9669	0.815992	0.220138	3.706731	0.00021	0.000719
CNAG_00906	5178.014	0.18692	0.069417	2.69273	0.007087	0.016997
CNAG_00905	511.9902	1.411981	0.186131	7.585948	3.30E-14	3.49E-13
CNAG_00904	1033.311	0.969631	0.122094	7.941671	1.99E-15	2.25E-14
CNAG_00903	662.8758	-0.5129	0.120989	-4.23928	2.24E-05	9.18E-05
CNAG_00902	757.3756	-0.55523	0.096189	-5.77227	7.82E-09	5.21E-08
CNAG_00897	2419.339	-2.43663	0.095368	-25.5497	5.53E-144	6.92E-142
CNAG_00896	340.9561	0.302034	0.135883	2.222758	0.026232	0.053774
CNAG_12505	112.2884	5.366901	0.427168	12.56392	3.33E-36	8.21E-35
CNAG_12506	54.10843	0.827621	0.298583	2.771832	0.005574	0.013779
CNAG_00895	1038.194	-0.9404	0.096103	-9.78538	1.30E-22	2.07E-21
CNAG_00893	1093.848	0.199908	0.096896	2.063118	0.039101	0.075919
CNAG_00891	13370.87	0.642402	0.073098	8.788284	1.52E-18	1.98E-17
CNAG_00890	1750.491	0.175599	0.073345	2.394151	0.016659	0.036185

CNAG_00886	37300.14	-0.2816	0.05819	-4.83939	1.30E-06	6.56E-06
CNAG_00884	1382.399	0.394122	0.074485	5.291271	1.21E-07	7.04E-07
CNAG_00883	609.6072	0.427221	0.110403	3.869638	0.000109	0.000396
CNAG_07453	2365.365	-0.27058	0.075087	-3.60354	0.000314	0.001037
CNAG_07454	13.07649	2.615423	0.66743	3.918648	8.90E-05	0.00033
CNAG_00879	2856.496	0.439308	0.09	4.881224	1.05E-06	5.39E-06
CNAG_00877	1222.624	0.462497	0.088661	5.216482	1.82E-07	1.03E-06
CNAG_00875	681.8511	-0.42559	0.108688	-3.91572	9.01E-05	0.000334
CNAG_00873	424.6345	0.332826	0.122493	2.717112	0.006585	0.015921
CNAG_00872	2044.24	0.186476	0.070506	2.644815	0.008174	0.019318
CNAG_07455	17.59246	3.346372	0.66007	5.069723	3.98E-07	2.16E-06
CNAG_00871	550.8597	2.443552	0.124852	19.57151	2.71E-85	1.61E-83
CNAG_00870	75.60761	0.740276	0.319046	2.320283	0.020326	0.042952
CNAG_12511	26.71083	2.138439	0.458038	4.66869	3.03E-06	1.45E-05
CNAG_00869	1976.259	1.761523	0.103284	17.05517	3.20E-65	1.50E-63
CNAG_12512	18.31125	3.144855	0.603027	5.215115	1.84E-07	1.04E-06
CNAG_00867	83.20172	2.637454	0.290351	9.083686	1.05E-19	1.47E-18
CNAG_07456	224.0475	0.698115	0.179848	3.881688	0.000104	0.000379
CNAG_00864	20.88274	-1.59441	0.498451	-3.19874	0.00138	0.003934
CNAG_07457	55.18671	-1.13267	0.306465	-3.69593	0.000219	0.000747
CNAG_00863	101.0154	-0.73541	0.215807	-3.4077	0.000655	0.002013
CNAG_00862	706.6025	-0.73462	0.099645	-7.37242	1.68E-13	1.69E-12
CNAG_00860	394.9065	0.465827	0.124478	3.742257	0.000182	0.000633
CNAG_02558	119.4073	-0.7009	0.220059	-3.18507	0.001447	0.004097
CNAG_02555	1101.056	-0.32987	0.082855	-3.98127	6.85E-05	0.000259
CNAG_02549	735.6237	0.220433	0.106447	2.070828	0.038375	0.074745
CNAG_02548	3698.579	2.63527	0.220695	11.94079	7.25E-33	1.61E-31
CNAG_10064	5.95209	3.544977	1.155003	3.069235	0.002146	0.005857
CNAG_02546	5165.663	0.708721	0.065769	10.77597	4.47E-27	8.30E-26
CNAG_02545	13569.65	-0.29626	0.065262	-4.53951	5.64E-06	2.60E-05
CNAG_02543	1034.75	0.291426	0.08529	3.416879	0.000633	0.001956
CNAG_02542	1966.361	0.785048	0.105458	7.44418	9.75E-14	1.00E-12
CNAG_02541	3208.828	0.355128	0.078322	4.534234	5.78E-06	2.67E-05
CNAG_02540	1021.753	0.569843	0.086315	6.601897	4.06E-11	3.40E-10
CNAG_02539	1702.555	0.928912	0.083706	11.0973	1.29E-28	2.52E-27
CNAG_12515	112.7204	0.98231	0.214285	4.584123	4.56E-06	2.13E-05
CNAG_02536	798.4722	-0.23256	0.106341	-2.1869	0.02875	0.058202
CNAG_02532	501.4713	1.23408	0.12058	10.23456	1.39E-24	2.38E-23
CNAG_12516	107.2199	0.461685	0.221494	2.084416	0.037122	0.072556
CNAG_02531	257.3539	0.366293	0.144669	2.531933	0.011344	0.025903
CNAG_02529	608.1006	-0.62925	0.118124	-5.32702	9.98E-08	5.85E-07
CNAG_02528	1125.701	-0.46569	0.099244	-4.69237	2.70E-06	1.30E-05
CNAG_02527	5354.113	1.296765	0.092661	13.99477	1.68E-44	5.15E-43
CNAG_12518	123.7797	0.599721	0.293975	2.04004	0.041346	0.079636
CNAG_02526	415.6408	4.412258	0.216974	20.33541	6.25E-92	4.03E-90
CNAG_02525	132.1359	1.201995	0.205447	5.850637	4.90E-09	3.34E-08
CNAG_02523	957.9047	0.502109	0.091093	5.512073	3.55E-08	2.19E-07

CNAG_02517	94.32553	0.58883	0.223544	2.634067	0.008437	0.019871
CNAG_02516	642.0123	0.634374	0.105108	6.035457	1.59E-09	1.13E-08
CNAG_02515	519.6383	0.565228	0.126639	4.463305	8.07E-06	3.62E-05
CNAG_07622	1244.495	-0.22434	0.101964	-2.20015	0.027797	0.056549
CNAG_02512	1322.255	0.284673	0.077044	3.694958	0.00022	0.000749
CNAG_02511	584.0263	0.205959	0.104287	1.974922	0.048277	0.090959
CNAG_12522	132.9902	1.375213	0.308914	4.451768	8.52E-06	3.80E-05
CNAG_02510	1600.064	0.661073	0.102638	6.44084	1.19E-10	9.51E-10
CNAG_02508	305.4746	0.326281	0.145276	2.245945	0.024707	0.051009
CNAG_02507	4682.186	-0.1914	0.079736	-2.40041	0.016377	0.035638
CNAG_02503	1495.369	-0.17527	0.076356	-2.29549	0.021705	0.045504
CNAG_02502	4326.722	0.156879	0.061265	2.560667	0.010447	0.024035
CNAG_02501	1077.942	-0.31164	0.102135	-3.05127	0.002279	0.006188
CNAG_02500	11808.25	0.708851	0.069547	10.19236	2.14E-24	3.65E-23
CNAG_02497	160.8685	0.671769	0.208936	3.215183	0.001304	0.003734
CNAG_02496	548.1451	-0.2932	0.116083	-2.52583	0.011543	0.026243
CNAG_02495	2251.785	-0.32416	0.076261	-4.25074	2.13E-05	8.79E-05
CNAG_12525	22.45864	0.99119	0.473005	2.095516	0.036125	0.070821
CNAG_02492	877.1868	-0.25327	0.099423	-2.54742	0.010852	0.0249
CNAG_02490	969.4046	0.3093	0.089096	3.471527	0.000518	0.001625
CNAG_02488	532.0962	1.524336	0.129254	11.7933	4.23E-32	9.09E-31
CNAG_02487	987.6137	-0.57497	0.11747	-4.89464	9.85E-07	5.05E-06
CNAG_02486	4658.327	-0.18237	0.060498	-3.01454	0.002574	0.006904
CNAG_02485	16804.44	-0.34171	0.071475	-4.78082	1.75E-06	8.67E-06
CNAG_02481	2312.506	0.159888	0.073093	2.187467	0.028708	0.058132
CNAG_02479	508.4982	0.923975	0.115105	8.027212	9.97E-16	1.15E-14
CNAG_12528	26.5949	0.866241	0.413599	2.094396	0.036225	0.070999
CNAG_02478	1083.409	1.486347	0.092238	16.11434	2.02E-58	8.18E-57
CNAG_02476	444.2318	1.102332	0.119161	9.250752	2.23E-20	3.23E-19
CNAG_02475	881.9709	0.509023	0.09868	5.158304	2.49E-07	1.38E-06
CNAG_07626	391.3638	1.532161	0.124522	12.3043	8.59E-35	2.00E-33
CNAG_02469	1682.086	0.428107	0.095793	4.469095	7.86E-06	3.53E-05
CNAG_02466	421.6873	0.487297	0.110944	4.392257	1.12E-05	4.88E-05
CNAG_02464	835.6742	0.429167	0.100305	4.278598	1.88E-05	7.83E-05
CNAG_02463	186.411	1.018076	0.182889	5.566634	2.60E-08	1.63E-07
CNAG_07627	1229.753	-0.24315	0.087495	-2.77896	0.005453	0.013518
CNAG_02458	2456.966	0.352773	0.074658	4.725184	2.30E-06	1.12E-05
CNAG_02457	5511.929	0.207105	0.060629	3.415939	0.000636	0.001961
CNAG_07628	12246.84	-0.19236	0.068621	-2.80326	0.005059	0.012636
CNAG_12536	14.36655	2.434539	0.700211	3.476868	0.000507	0.001595
CNAG_02455	1004.306	0.714144	0.114014	6.263623	3.76E-10	2.87E-09
CNAG_02454	1801.946	0.419962	0.074795	5.614869	1.97E-08	1.25E-07
CNAG_02452	152.1631	-0.43487	0.181882	-2.39095	0.016805	0.036435
CNAG_02445	2202.135	-0.33912	0.081129	-4.17998	2.92E-05	0.000117
CNAG_02444	364.1082	-0.6856	0.124773	-5.49476	3.91E-08	2.40E-07
CNAG_02443	3683.578	0.507226	0.102699	4.938965	7.85E-07	4.09E-06
CNAG_02440	3642.231	0.620873	0.066376	9.353875	8.45E-21	1.25E-19

CNAG_02438	162.1094	-0.80998	0.194912	-4.15563	3.24E-05	0.00013
CNAG_02437	1869.034	0.443735	0.075384	5.886303	3.95E-09	2.72E-08
CNAG_02435	3283.171	-0.29929	0.080482	-3.71873	0.0002	0.000689
CNAG_02434	1685.226	0.602218	0.092294	6.524982	6.80E-11	5.58E-10
CNAG_02432	964.5717	0.380716	0.092434	4.118781	3.81E-05	0.000151
CNAG_02431	808.8768	-0.69365	0.092769	-7.47719	7.59E-14	7.87E-13
CNAG_02429	81.94989	-1.52014	0.28246	-5.38178	7.38E-08	4.41E-07
CNAG_02427	460.786	-0.31292	0.122977	-2.54456	0.010942	0.025073
CNAG_02426	4379.529	-0.30042	0.068587	-4.3801	1.19E-05	5.13E-05
CNAG_02425	284.862	-0.58066	0.140945	-4.11976	3.79E-05	0.00015
CNAG_02422	1161.089	-0.51729	0.080757	-6.40542	1.50E-10	1.19E-09
CNAG_02421	4555.462	-0.15458	0.065483	-2.36065	0.018243	0.039113
CNAG_02420	70.05482	0.74521	0.268605	2.774372	0.005531	0.013681
CNAG_02418	10505.16	-0.14909	0.060699	-2.45629	0.014038	0.031166
CNAG_02417	141.3121	-0.56246	0.215933	-2.60477	0.009194	0.021507
CNAG_12541	182.7254	-0.50857	0.172524	-2.94783	0.0032	0.00839
CNAG_02415	1782.168	0.562026	0.077821	7.222024	5.12E-13	5.03E-12
CNAG_02413	3567.284	-0.64411	0.069987	-9.20333	3.47E-20	4.95E-19
CNAG_07984	17.24001	-1.50098	0.545791	-2.75011	0.005958	0.014576
CNAG_12543	41.01777	-1.20673	0.400445	-3.01348	0.002583	0.006924
CNAG_02409	658.5635	0.560079	0.102042	5.488728	4.05E-08	2.48E-07
CNAG_02407	1730.354	0.367961	0.085123	4.3227	1.54E-05	6.53E-05
CNAG_02405	662.5594	0.787129	0.116953	6.730296	1.69E-11	1.46E-10
CNAG_02404	8525.972	0.451476	0.080529	5.60636	2.07E-08	1.31E-07
CNAG_02403	3824.781	0.265439	0.062758	4.229594	2.34E-05	9.55E-05
CNAG_02402	959.7893	-0.38284	0.102911	-3.72012	0.000199	0.000686
CNAG_02400	2547.021	0.147982	0.06682	2.214641	0.026785	0.054796
CNAG_02397	355.3747	0.347311	0.129414	2.683725	0.007281	0.017426
CNAG_02387	1850.033	0.954118	0.084587	11.27977	1.65E-29	3.34E-28
CNAG_02386	1013.712	0.655448	0.094241	6.955051	3.52E-12	3.20E-11
CNAG_02385	1023.462	0.472036	0.090123	5.237668	1.63E-07	9.26E-07
CNAG_02383	572.7471	-0.29879	0.128949	-2.31712	0.020497	0.043228
CNAG_07985	226.8192	0.411336	0.150593	2.731436	0.006306	0.015327
CNAG_07986	792.4544	0.212938	0.090706	2.347561	0.018897	0.040345
CNAG_02378	8614.322	-0.30677	0.066297	-4.62712	3.71E-06	1.76E-05
CNAG_02377	1655.562	-0.3441	0.080941	-4.25128	2.13E-05	8.77E-05
CNAG_02376	1302.189	-0.15936	0.078397	-2.03269	0.042084	0.080883
CNAG_02373	1161.431	-0.21395	0.08739	-2.44824	0.014356	0.031785
CNAG_02372	1983.983	-0.407	0.06904	-5.89517	3.74E-09	2.59E-08
CNAG_12551	27.67231	1.674737	0.431141	3.884427	0.000103	0.000375
CNAG_02368	1320.19	-0.21736	0.081963	-2.65198	0.008002	0.018952
CNAG_02367	6304.017	-0.15401	0.057251	-2.69006	0.007144	0.017124
CNAG_02366	1246.524	0.380043	0.085535	4.443113	8.87E-06	3.95E-05
CNAG_02365	828.7584	2.72287	0.108759	25.03586	2.49E-138	2.77E-136
CNAG_02362	650.5263	-0.22201	0.111089	-1.99846	0.045667	0.086847
CNAG_12554	123.4374	0.478001	0.191069	2.501721	0.012359	0.027881
CNAG_02361	1601.361	0.312584	0.080638	3.876383	0.000106	0.000386

CNAG_02358	370.7217	-0.58474	0.12797	-4.56936	4.89E-06	2.28E-05
CNAG_02357	968.6082	0.228814	0.088998	2.570993	0.010141	0.023383
CNAG_02356	1293.741	-0.17134	0.087025	-1.96883	0.048972	0.092034
CNAG_02353	372.259	0.731939	0.129998	5.630383	1.80E-08	1.15E-07
CNAG_07634	2237.043	0.479492	0.088077	5.444032	5.21E-08	3.15E-07
CNAG_02351	2679.591	0.621234	0.088281	7.037038	1.96E-12	1.84E-11
CNAG_07987	472.011	-0.39674	0.114936	-3.45187	0.000557	0.00174
CNAG_02345	580.7666	-0.29807	0.108765	-2.7405	0.006135	0.014946
CNAG_12557	56.34422	5.155652	0.589345	8.748108	2.17E-18	2.82E-17
CNAG_02343	2393.029	1.492089	0.06793	21.96512	6.21E-107	4.95E-105
CNAG_02342	533.6535	0.483851	0.117207	4.128161	3.66E-05	0.000145
CNAG_02340	626.369	-0.24761	0.101177	-2.44725	0.014395	0.031853
CNAG_02338	24243.49	-0.20451	0.073203	-2.79367	0.005211	0.012981
CNAG_02336	638.276	0.668878	0.102575	6.520853	6.99E-11	5.72E-10
CNAG_02335	8196.066	-0.53412	0.09769	-5.46751	4.56E-08	2.78E-07
CNAG_12558	173.3537	-0.72939	0.219014	-3.33034	0.000867	0.002596
CNAG_02333	425.4437	-0.38195	0.124268	-3.07362	0.002115	0.005784
CNAG_02332	1119.977	-0.46516	0.085168	-5.46169	4.72E-08	2.87E-07
CNAG_02331	28831.43	-0.31182	0.069046	-4.51619	6.30E-06	2.88E-05
CNAG_02330	43423.93	-0.17785	0.07497	-2.37226	0.01768	0.038066
CNAG_02328	4667.413	0.256799	0.063198	4.06343	4.84E-05	0.000188
CNAG_02327	521.2414	-0.23992	0.103354	-2.32135	0.020268	0.042878
CNAG_02326	4083.647	-0.24182	0.064682	-3.73864	0.000185	0.000642
CNAG_02325	793.6832	-0.2366	0.090129	-2.6251	0.008662	0.020361
CNAG_02324	952.0468	-0.23897	0.100149	-2.38609	0.017028	0.03683
CNAG_02323	2369.601	-0.51399	0.074146	-6.93212	4.15E-12	3.76E-11
CNAG_12560	22.48223	-2.37188	0.552286	-4.29466	1.75E-05	7.34E-05
CNAG_02322	948.6456	-0.33303	0.08831	-3.77116	0.000162	0.000571
CNAG_02318	890.6368	0.461544	0.098727	4.674957	2.94E-06	1.41E-05
CNAG_02317	2509.293	0.659798	0.069396	9.507778	1.95E-21	2.98E-20
CNAG_02316	1744.714	-0.30349	0.1079	-2.81267	0.004913	0.012291
CNAG_02315	21494.39	0.571614	0.0722	7.917103	2.43E-15	2.74E-14
CNAG_02314	149.981	-0.78094	0.178208	-4.38221	1.17E-05	5.09E-05
CNAG_02312	919.2693	0.238664	0.094104	2.536158	0.011208	0.025639
CNAG_02310	1823.443	-0.28025	0.078412	-3.57411	0.000351	0.001148
CNAG_02305	857.3013	1.220867	0.111097	10.98921	4.31E-28	8.30E-27
CNAG_02304	181.9371	-0.57536	0.182455	-3.15346	0.001613	0.004533
CNAG_02302	296.1312	0.693478	0.130056	5.332147	9.71E-08	5.70E-07
CNAG_02301	10414.37	-0.15578	0.071874	-2.16742	0.030203	0.06075
CNAG_02300	11759.61	-0.56209	0.062067	-9.05628	1.35E-19	1.88E-18
CNAG_02299	1073.853	0.182267	0.08507	2.142566	0.032148	0.06406
CNAG_02294	4675.407	-0.20967	0.062386	-3.36078	0.000777	0.002351
CNAG_02292	2671.25	-0.74258	0.079221	-9.37347	7.02E-21	1.04E-19
CNAG_02289	468.4094	-0.23124	0.112949	-2.04728	0.040631	0.078499
CNAG_02287	746.8986	-0.33453	0.103392	-3.23551	0.001214	0.003499
CNAG_02286	2282.794	-0.14553	0.069167	-2.10401	0.035378	0.069476
CNAG_02285	27123.64	0.240754	0.103764	2.320212	0.020329	0.042952

CNAG_02284	235.8217	-0.562	0.14671	-3.83071	0.000128	0.000457
CNAG_02282	1253.563	-0.29164	0.091069	-3.20247	0.001363	0.003886
CNAG_02281	1037.512	-0.30769	0.091896	-3.34821	0.000813	0.002446
CNAG_02280	1138.219	-0.24838	0.110842	-2.24087	0.025034	0.051579
CNAG_07635	1778.458	-0.54617	0.070358	-7.76279	8.31E-15	9.03E-14
CNAG_07638	1663.493	0.855541	0.072743	11.7612	6.19E-32	1.32E-30
CNAG_07640	662.9757	-0.27756	0.109727	-2.52957	0.01142	0.02603
CNAG_02270	1572.568	-0.72004	0.081812	-8.80114	1.35E-18	1.77E-17
CNAG_02269	686.0902	-0.45992	0.11236	-4.09326	4.25E-05	0.000167
CNAG_02268	469.1995	-0.25168	0.118856	-2.1175	0.034217	0.067488
CNAG_12567	34.6486	-1.49065	0.377466	-3.94909	7.84E-05	0.000294
CNAG_02267	1649.019	-0.18284	0.075072	-2.43554	0.014869	0.032749
CNAG_07164	610.9024	-0.30955	0.106036	-2.9193	0.003508	0.009119
CNAG_07163	2074.541	0.841797	0.075082	11.21172	3.57E-29	7.15E-28
CNAG_02266	6788.835	0.893063	0.075184	11.87835	1.53E-32	3.34E-31
CNAG_02265	3889.434	0.180001	0.063912	2.816399	0.004857	0.012176
CNAG_02264	2280.555	1.818539	0.105457	17.24433	1.23E-66	5.90E-65
CNAG_02263	405.4538	-0.46496	0.133001	-3.49594	0.000472	0.001498
CNAG_02262	3134.535	0.470026	0.073748	6.373388	1.85E-10	1.46E-09
CNAG_02260	2258.387	-0.29791	0.070274	-4.23934	2.24E-05	9.18E-05
CNAG_02259	1132.402	0.317484	0.093161	3.407919	0.000655	0.002012
CNAG_02258	1476.611	-0.46334	0.07337	-6.31519	2.70E-10	2.10E-09
CNAG_02257	19369.72	-0.12909	0.059594	-2.16616	0.030299	0.060913
CNAG_12570	74.14115	0.626969	0.257747	2.432501	0.014995	0.032967
CNAG_12571	62.69955	1.617559	0.316061	5.117873	3.09E-07	1.70E-06
CNAG_02240	2579.249	-0.19746	0.067201	-2.93839	0.003299	0.008617
CNAG_02237	4580.069	-0.28309	0.079013	-3.58284	0.00034	0.001114
CNAG_02236	2231.945	-0.48534	0.08084	-6.00379	1.93E-09	1.37E-08
CNAG_02234	54647.86	-0.17455	0.063114	-2.76557	0.005682	0.014004
CNAG_02232	2064.647	-0.32226	0.0727	-4.43278	9.30E-06	4.12E-05
CNAG_07646	192.9672	-0.63822	0.177311	-3.59942	0.000319	0.001052
CNAG_02226	290.7281	1.295351	0.192703	6.72202	1.79E-11	1.54E-10
CNAG_02220	2138.508	0.555004	0.088675	6.258879	3.88E-10	2.95E-09
CNAG_02219	401.4697	-0.32794	0.130585	-2.5113	0.012029	0.027211
CNAG_02217	248.0087	0.881595	0.179727	4.905192	9.33E-07	4.80E-06
CNAG_02215	904.7927	0.577362	0.09027	6.395963	1.60E-10	1.26E-09
CNAG_02214	471.1354	0.99057	0.126748	7.815288	5.48E-15	6.03E-14
CNAG_02213	2320.999	-0.25379	0.074219	-3.41955	0.000627	0.001943
CNAG_02212	14265.89	-0.16704	0.067426	-2.47739	0.013235	0.029626
CNAG_02210	889.3993	-0.19471	0.090668	-2.14745	0.031758	0.063437
CNAG_02209	9774.168	-0.13385	0.066818	-2.00322	0.045153	0.086011
CNAG_02208	6309.214	0.493114	0.064058	7.697913	1.38E-14	1.49E-13
CNAG_02205	378.5779	-0.59425	0.13694	-4.3395	1.43E-05	6.10E-05
CNAG_02204	1858.213	0.36369	0.074193	4.901955	9.49E-07	4.88E-06
CNAG_02203	1351.173	0.264511	0.081921	3.228872	0.001243	0.003567
CNAG_02202	4318.931	-0.25438	0.067199	-3.7854	0.000153	0.000542
CNAG_02201	402.8127	0.495608	0.165727	2.990511	0.002785	0.007415

CNAG_02198	530.0916	-0.37607	0.103843	-3.62157	0.000293	0.000971
CNAG_02197	1975.271	-0.23779	0.07844	-3.03145	0.002434	0.006572
CNAG_02193	719.9626	-0.48156	0.109591	-4.39417	1.11E-05	4.84E-05
CNAG_02192	43.79218	0.660569	0.332463	1.986895	0.046934	0.08884
CNAG_02190	897.7692	0.90834	0.099621	9.117917	7.66E-20	1.08E-18
CNAG_02189	4139.646	-0.17899	0.08831	-2.0268	0.042683	0.081862
CNAG_02187	785.0951	-0.21535	0.092421	-2.33005	0.019803	0.042026
CNAG_02183	977.0796	0.18991	0.08772	2.16496	0.030391	0.061067
CNAG_02182	4996.153	2.465408	0.106585	23.13095	2.26E-118	1.93E-116
CNAG_02181	1008.951	-0.49593	0.085462	-5.80295	6.52E-09	4.39E-08
CNAG_02180	2752.141	0.146875	0.073652	1.994175	0.046133	0.087548
CNAG_02179	1535.918	-0.37298	0.079795	-4.6742	2.95E-06	1.42E-05
CNAG_02177	6125.277	-0.40906	0.062196	-6.57694	4.80E-11	3.98E-10
CNAG_02176	1828.888	-0.36238	0.089855	-4.03293	5.51E-05	0.000212
CNAG_02175	2207.645	-0.20454	0.079114	-2.58533	0.009729	0.022541
CNAG_02174	4590.067	-0.36617	0.064377	-5.68789	1.29E-08	8.33E-08
CNAG_02171	908.9165	-0.2255	0.087159	-2.58717	0.009677	0.02244
CNAG_02168	1718.291	0.295349	0.09928	2.974919	0.002931	0.007754
CNAG_02166	7743.14	-0.23434	0.057834	-4.05193	5.08E-05	0.000196
CNAG_02165	1102.655	-0.65376	0.106287	-6.1509	7.70E-10	5.70E-09
CNAG_02164	1125.922	0.433311	0.113753	3.809238	0.000139	0.000495
CNAG_02163	394.2006	-0.52015	0.144214	-3.60679	0.00031	0.001025
CNAG_02161	2479.829	-0.15743	0.066748	-2.35864	0.018342	0.039305
CNAG_02156	556.8918	1.293404	0.12506	10.34223	4.54E-25	7.93E-24
CNAG_02154	825.0226	-0.56992	0.101212	-5.63093	1.79E-08	1.14E-07
CNAG_02153	2344.987	-0.52899	0.087122	-6.07182	1.26E-09	9.19E-09
CNAG_02152	936.1077	0.933541	0.09445	9.884001	4.88E-23	7.99E-22
CNAG_02151	1436.805	-0.37281	0.099195	-3.75838	0.000171	0.000598
CNAG_02147	842.367	2.089229	0.099306	21.03834	2.92E-98	2.09E-96
CNAG_02143	778.6342	0.230126	0.111073	2.071847	0.03828	0.074595
CNAG_02141	516.5441	0.263177	0.110994	2.37108	0.017736	0.038168
CNAG_02140	6646.148	0.975164	0.069665	13.99783	1.61E-44	4.95E-43
CNAG_02139	2745.232	-0.67433	0.073193	-9.21312	3.17E-20	4.54E-19
CNAG_02138	371.2661	0.556219	0.14646	3.797742	0.000146	0.000517
CNAG_12586	65.64317	0.698802	0.291925	2.393775	0.016676	0.036212
CNAG_02134	3098.066	-0.47462	0.082181	-5.77533	7.68E-09	5.12E-08
CNAG_02133	5403.462	-0.34233	0.058173	-5.88466	3.99E-09	2.75E-08
CNAG_02131	1984.337	1.504181	0.075234	19.99334	6.29E-89	3.97E-87
CNAG_02130	5279.926	-0.33588	0.078792	-4.26292	2.02E-05	8.36E-05
CNAG_02128	10016	-0.34751	0.060787	-5.71692	1.08E-08	7.09E-08
CNAG_02127	712.0854	-0.61068	0.092147	-6.62731	3.42E-11	2.88E-10
CNAG_02124	899.9151	0.310934	0.09781	3.178973	0.001478	0.004177
CNAG_02123	1388.113	0.775077	0.090864	8.530079	1.46E-17	1.83E-16
CNAG_02121	1027.681	-0.25391	0.091968	-2.76086	0.005765	0.014174
CNAG_02120	4972.992	1.254182	0.063126	19.86789	7.72E-88	4.83E-86
CNAG_02119	814.6334	-0.37655	0.089182	-4.22224	2.42E-05	9.84E-05
CNAG_02117	916.1622	-0.44967	0.095987	-4.68463	2.80E-06	1.35E-05

CNAG_02115	1652.054	-0.89429	0.07736	-11.5601	6.57E-31	1.38E-29
CNAG_02114	2752.276	0.533714	0.078612	6.789206	1.13E-11	9.91E-11
CNAG_02113	1739.393	-0.2686	0.081983	-3.27627	0.001052	0.003076
CNAG_02111	1151.189	-0.19846	0.081242	-2.44278	0.014575	0.032182
CNAG_02110	3014.102	-0.18564	0.071884	-2.58246	0.00981	0.02271
CNAG_02109	1838.329	0.208678	0.088903	2.347264	0.018912	0.040367
CNAG_02107	937.1705	1.165204	0.105041	11.09281	1.36E-28	2.64E-27
CNAG_02105	185.9056	-0.92715	0.185412	-5.0005	5.72E-07	3.04E-06
CNAG_02104	2317.213	2.553399	0.076327	33.45342	2.30E-245	7.46E-243
CNAG_12593	153.7627	1.514446	0.210071	7.209201	5.63E-13	5.50E-12
CNAG_02103	836.1567	0.568935	0.101878	5.584495	2.34E-08	1.47E-07
CNAG_02100	13571.52	0.147563	0.066154	2.230602	0.025708	0.052805
CNAG_02099	13953.42	0.149251	0.065243	2.287614	0.02216	0.046374
CNAG_02096	270.5046	-0.36766	0.135704	-2.70929	0.006743	0.016268
CNAG_02094	2176.524	-0.35423	0.081321	-4.35592	1.33E-05	5.68E-05
CNAG_02093	975.0277	0.601651	0.095329	6.311302	2.77E-10	2.15E-09
CNAG_02091	14558.67	-0.12398	0.060545	-2.04779	0.04058	0.078457
CNAG_02089	547.5381	0.397727	0.138067	2.880692	0.003968	0.010152
CNAG_02088	1101.882	-0.78899	0.089201	-8.84501	9.15E-19	1.21E-17
CNAG_02084	4169.406	-0.45284	0.067501	-6.70867	1.96E-11	1.68E-10
CNAG_02083	1843.461	3.650457	0.08736	41.7862	0	0
CNAG_12597	100.5393	1.030305	0.23634	4.359421	1.30E-05	5.60E-05
CNAG_12598	94.77128	1.531732	0.233576	6.557746	5.46E-11	4.52E-10
CNAG_02079	6590.19	-1.18816	0.096032	-12.3725	3.68E-35	8.67E-34
CNAG_02077	547.2243	-0.34897	0.103557	-3.36984	0.000752	0.002288
CNAG_02076	1487.989	-0.52803	0.085429	-6.18093	6.37E-10	4.76E-09
CNAG_02071	2644.508	0.327048	0.086652	3.774275	0.00016	0.000565
CNAG_02067	2846.841	0.613433	0.07179	8.544846	1.29E-17	1.61E-16
CNAG_02066	121.3151	0.430799	0.213538	2.017432	0.04365	0.0835
CNAG_02064	3610.97	-0.17246	0.061982	-2.78243	0.005395	0.01339
CNAG_02062	966.3409	1.234059	0.090281	13.66908	1.55E-42	4.53E-41
CNAG_02061	432.5537	0.409217	0.120045	3.408856	0.000652	0.002007
CNAG_02060	1330.485	-0.83891	0.115797	-7.2446	4.34E-13	4.29E-12
CNAG_12600	394.5593	0.442632	0.155168	2.852596	0.004336	0.011025
CNAG_02057	741.6325	-0.70689	0.099967	-7.07128	1.54E-12	1.45E-11
CNAG_02053	38.43995	-1.20251	0.381376	-3.15309	0.001616	0.004536
CNAG_12603	20.53524	-1.0066	0.484654	-2.07694	0.037807	0.073766
CNAG_02049	69.07862	2.346075	0.29702	7.898718	2.82E-15	3.16E-14
CNAG_02048	864.6425	0.562478	0.090792	6.195221	5.82E-10	4.36E-09
CNAG_02044	159.0894	0.823781	0.170404	4.834285	1.34E-06	6.71E-06
CNAG_02043	659.3559	2.261997	0.107691	21.0045	5.97E-98	4.22E-96
CNAG_12604	4.785441	3.925355	1.355199	2.896517	0.003773	0.009721
CNAG_07649	6307.124	0.609632	0.060355	10.10077	5.48E-24	9.22E-23
CNAG_02041	2063.891	1.127422	0.265422	4.247658	2.16E-05	8.89E-05
CNAG_02040	290.5437	1.082761	0.161106	6.720786	1.81E-11	1.55E-10
CNAG_02039	108.5635	1.502606	0.2277	6.599053	4.14E-11	3.46E-10
CNAG_02038	2830.853	0.437361	0.075268	5.810737	6.22E-09	4.21E-08

CNAG_02037	2821.645	-0.26909	0.078003	-3.44976	0.000561	0.001753
CNAG_02035	12806.45	-0.61174	0.059928	-10.2078	1.83E-24	3.13E-23
CNAG_02030	10123.13	0.911363	0.09035	10.08701	6.31E-24	1.06E-22
CNAG_02029	1103.648	-0.32472	0.09597	-3.38351	0.000716	0.002188
CNAG_02027	1428.811	0.75035	0.077348	9.700927	2.99E-22	4.70E-21
CNAG_02026	196.0705	0.560761	0.166419	3.369571	0.000753	0.002289
CNAG_02024	1640.495	-0.36927	0.089604	-4.1212	3.77E-05	0.000149
CNAG_02023	93.04264	-0.6967	0.226032	-3.0823	0.002054	0.005633
CNAG_02021	1010.775	-0.3598	0.085639	-4.20135	2.65E-05	0.000107
CNAG_02020	1340.402	-0.3991	0.090693	-4.40059	1.08E-05	4.71E-05
CNAG_07651	1061.089	-0.49046	0.084232	-5.8227	5.79E-09	3.94E-08
CNAG_07652	14.92404	-1.09606	0.552682	-1.98317	0.047349	0.089542
CNAG_06524	11903.13	6.660527	0.265914	25.04769	1.85E-138	2.09E-136
CNAG_06525	194.1101	0.381853	0.175714	2.173151	0.029769	0.059981
CNAG_06526	2173.671	-0.42079	0.079539	-5.29034	1.22E-07	7.07E-07
CNAG_06527	34.10265	-1.22777	0.379315	-3.23681	0.001209	0.003485
CNAG_12608	35.48674	-0.95	0.375344	-2.53101	0.011374	0.025946
CNAG_06529	130.994	0.936306	0.203989	4.589991	4.43E-06	2.07E-05
CNAG_06531	1204.65	1.183443	0.10637	11.12567	9.41E-29	1.85E-27
CNAG_06534	8628.562	0.155802	0.065308	2.385651	0.017049	0.036855
CNAG_06536	559.4933	1.020711	0.110659	9.223941	2.86E-20	4.13E-19
CNAG_06539	165.1746	1.695483	0.217882	7.781657	7.16E-15	7.80E-14
CNAG_06540	171.9774	1.050047	0.18074	5.809714	6.26E-09	4.24E-08
CNAG_06541	5801.022	0.222732	0.111303	2.001136	0.045378	0.086357
CNAG_06544	12398.42	-0.73008	0.061917	-11.7912	4.34E-32	9.30E-31
CNAG_06545	4565.542	-0.55237	0.076865	-7.18626	6.66E-13	6.44E-12
CNAG_06549	898.1072	-0.44638	0.090848	-4.91344	8.95E-07	4.62E-06
CNAG_06550	3559.551	0.178539	0.064267	2.778092	0.005468	0.01355
CNAG_06551	372.5635	1.891276	0.175304	10.78858	3.90E-27	7.25E-26
CNAG_06554	1400.016	2.017842	0.081997	24.60859	1.02E-133	1.05E-131
CNAG_06555	1935.554	1.118308	0.073364	15.24322	1.83E-52	6.57E-51
CNAG_06556	382.7093	4.282597	0.178615	23.97673	4.86E-127	4.54E-125
CNAG_06557	437.5445	4.624025	0.181178	25.52202	1.12E-143	1.38E-141
CNAG_06559	774.1712	0.87585	0.126961	6.898553	5.25E-12	4.73E-11
CNAG_06563	5253.998	-0.31001	0.061644	-5.02911	4.93E-07	2.64E-06
CNAG_06565	900.7694	-0.38519	0.098055	-3.92828	8.56E-05	0.000318
CNAG_06566	1388.743	-0.42173	0.078846	-5.34874	8.86E-08	5.23E-07
CNAG_06567	1219.505	-0.74622	0.09063	-8.23377	1.81E-16	2.16E-15
CNAG_06568	1593.284	-1.71583	0.083282	-20.6026	2.60E-94	1.78E-92
CNAG_12616	189.2387	1.532499	0.173552	8.830222	1.04E-18	1.38E-17
CNAG_06569	931.257	0.188646	0.088723	2.126239	0.033483	0.066363
CNAG_06570	506.2656	0.356896	0.108917	3.276775	0.00105	0.003073
CNAG_07660	10627.15	-0.18291	0.067874	-2.69488	0.007041	0.016893
CNAG_06573	1249.401	0.288432	0.088191	3.270548	0.001073	0.003129
CNAG_06576	3567.04	1.583163	0.250754	6.3136	2.73E-10	2.12E-09
CNAG_06577	1001.566	1.408274	0.368995	3.816516	0.000135	0.000482
CNAG_06578	1014.675	1.171521	0.116937	10.01837	1.27E-23	2.10E-22

CNAG_12620	95.53988	0.514437	0.248419	2.070846	0.038373	0.074745
CNAG_07989	920.3455	-0.23649	0.094222	-2.50989	0.012077	0.027313
CNAG_07661	304.8418	0.591475	0.129979	4.550526	5.35E-06	2.48E-05
CNAG_12622	90.45288	1.662988	0.251998	6.599202	4.13E-11	3.46E-10
CNAG_06583	366.4325	1.026905	0.153122	6.706448	1.99E-11	1.70E-10
CNAG_06584	846.645	0.322785	0.08601	3.752863	0.000175	0.000609
CNAG_06588	1155.928	0.733754	0.085032	8.629132	6.18E-18	7.88E-17
CNAG_06589	797.146	-0.22268	0.110278	-2.01923	0.043464	0.083182
CNAG_06590	282.5501	-0.75178	0.147585	-5.09386	3.51E-07	1.92E-06
CNAG_06591	4582.476	-0.35052	0.078556	-4.46196	8.12E-06	3.64E-05
CNAG_06592	2160.723	0.156082	0.077458	2.01504	0.0439	0.083919
CNAG_06594	5776.146	-0.20892	0.065551	-3.1872	0.001437	0.004071
CNAG_06597	2189.753	-0.31114	0.067639	-4.6	4.22E-06	1.98E-05
CNAG_06598	52.61524	-0.89678	0.353143	-2.53943	0.011103	0.025422
CNAG_12627	131.1066	0.877389	0.194968	4.500165	6.79E-06	3.10E-05
CNAG_06602	1355.351	-0.45323	0.115812	-3.91347	9.10E-05	0.000337
CNAG_06603	1901.355	-0.28429	0.074458	-3.81807	0.000135	0.000479
CNAG_06605	65669.78	-0.19109	0.061851	-3.08954	0.002005	0.005523
CNAG_06606	2383.366	0.346181	0.082783	4.181778	2.89E-05	0.000116
CNAG_06607	2387.877	0.402517	0.071677	5.615692	1.96E-08	1.24E-07
CNAG_06608	1876.622	0.213911	0.071919	2.974323	0.002936	0.007767
CNAG_06610	259.7129	0.446899	0.138257	3.232369	0.001228	0.003534
CNAG_12630	81.16586	-0.48734	0.238381	-2.04438	0.040916	0.078919
CNAG_06611	3455.8	0.33311	0.062586	5.322453	1.02E-07	5.98E-07
CNAG_06612	1698.227	-0.26861	0.082867	-3.24149	0.001189	0.003435
CNAG_06614	995.0128	0.790473	0.12016	6.578509	4.75E-11	3.95E-10
CNAG_06616	878.1151	-0.40782	0.101395	-4.02212	5.77E-05	0.000221
CNAG_06617	1548.454	0.547188	0.0854	6.407331	1.48E-10	1.18E-09
CNAG_12635	1.404214	4.011354	2.018199	1.987591	0.046857	0.088723
CNAG_06621	7997.407	1.077191	0.064778	16.62908	4.29E-62	1.90E-60
CNAG_06622	276.6771	1.936454	0.183913	10.52921	6.34E-26	1.13E-24
CNAG_06623	10833.01	1.745868	0.108289	16.12234	1.78E-58	7.22E-57
CNAG_06624	1299.27	-0.34426	0.089903	-3.82924	0.000129	0.00046
CNAG_06625	2520.035	-0.15779	0.072554	-2.17475	0.029649	0.059783
CNAG_06628	2487.6	0.185375	0.08618	2.15101	0.031475	0.062982
CNAG_06631	1893.247	-0.18352	0.087876	-2.08842	0.03676	0.071927
CNAG_06633	45493.07	-0.18644	0.068883	-2.70656	0.006798	0.016363
CNAG_06635	3605.617	-0.21643	0.070717	-3.06049	0.00221	0.006017
CNAG_06636	281.4348	0.44404	0.149822	2.963776	0.003039	0.008007
CNAG_06638	8329.498	1.118952	0.081647	13.70478	9.51E-43	2.81E-41
CNAG_12642	73.83616	1.834861	0.26137	7.020161	2.22E-12	2.07E-11
CNAG_06643	567.8747	-0.31164	0.110263	-2.82631	0.004709	0.011842
CNAG_06645	2701.82	0.303559	0.080736	3.75989	0.00017	0.000595
CNAG_06646	13198.47	-0.39511	0.05647	-6.99669	2.62E-12	2.41E-11
CNAG_06647	1563.318	0.530744	0.080216	6.616455	3.68E-11	3.09E-10
CNAG_06649	247.9662	1.020099	0.142251	7.171113	7.44E-13	7.17E-12
CNAG_12643	2.53375	4.871188	1.779941	2.736713	0.006206	0.015111

CNAG_06650	2892.261	4.894956	0.106018	46.17105	0	0
CNAG_06651	285.0091	5.594552	0.273071	20.48751	2.78E-93	1.82E-91
CNAG_06652	224.7739	1.039835	0.163154	6.373321	1.85E-10	1.46E-09
CNAG_06656	2281.66	0.444066	0.073207	6.065867	1.31E-09	9.51E-09
CNAG_06657	303.1378	-0.42535	0.135002	-3.15071	0.001629	0.00457
CNAG_06658	714.0265	1.749418	0.120285	14.54396	6.38E-48	2.07E-46
CNAG_06660	822.0004	-0.42787	0.103774	-4.12309	3.74E-05	0.000148
CNAG_06661	1741.925	-0.35149	0.088157	-3.98706	6.69E-05	0.000253
CNAG_06663	9808.138	0.630087	0.062517	10.07859	6.87E-24	1.15E-22
CNAG_06664	629.6517	-0.66503	0.097638	-6.81118	9.68E-12	8.59E-11
CNAG_06666	2858.628	1.009984	0.079466	12.7096	5.23E-37	1.32E-35
CNAG_06669	1524.097	-0.34668	0.091706	-3.78034	0.000157	0.000552
CNAG_06670	1856.235	-0.31037	0.07556	-4.10768	4.00E-05	0.000158
CNAG_06671	2069.844	0.196748	0.075708	2.598774	0.009356	0.021839
CNAG_12646	10.9569	1.45831	0.660955	2.20637	0.027358	0.055787
CNAG_12647	201.8711	0.832118	0.164022	5.073202	3.91E-07	2.12E-06
CNAG_06674	856.2055	-0.25551	0.088437	-2.88916	0.003863	0.009925
CNAG_06676	452.5856	-0.35296	0.111813	-3.15667	0.001596	0.004488
CNAG_06678	1037.394	-0.54259	0.103386	-5.24824	1.54E-07	8.80E-07
CNAG_06679	2983.574	-0.27255	0.066726	-4.0846	4.42E-05	0.000172
CNAG_06680	751.8678	-0.34253	0.103072	-3.32321	0.00089	0.002648
CNAG_06681	769.904	-0.40196	0.121471	-3.30914	0.000936	0.002773
CNAG_07665	5035.624	0.270308	0.090371	2.991102	0.00278	0.007408
CNAG_07177	4747.938	0.590991	0.097914	6.035845	1.58E-09	1.13E-08
CNAG_06690	2194.134	-0.46581	0.068742	-6.77631	1.23E-11	1.08E-10
CNAG_06692	857.251	0.380393	0.088188	4.313421	1.61E-05	6.78E-05
CNAG_06693	544.0034	0.581685	0.110276	5.274786	1.33E-07	7.67E-07
CNAG_06694	253.2691	-0.9857	0.141912	-6.94582	3.76E-12	3.42E-11
CNAG_06696	4931.863	-0.35481	0.061103	-5.80682	6.37E-09	4.30E-08
CNAG_06697	1037.256	-0.29127	0.090646	-3.21325	0.001312	0.003755
CNAG_07990	1308.991	-0.50961	0.081061	-6.28671	3.24E-10	2.49E-09
CNAG_06698	1332.955	-0.50847	0.094695	-5.36954	7.89E-08	4.71E-07
CNAG_06699	107055.7	-0.19361	0.054938	-3.52421	0.000425	0.001363
CNAG_05671	634.5978	-0.2087	0.102756	-2.03104	0.042251	0.081167
CNAG_05673	1387.815	-0.28741	0.077782	-3.69509	0.00022	0.000749
CNAG_05674	953.5084	-0.48126	0.085842	-5.60631	2.07E-08	1.31E-07
CNAG_05676	376.8417	0.452265	0.139215	3.248674	0.001159	0.00336
CNAG_05678	1086.117	0.474574	0.082428	5.757422	8.54E-09	5.66E-08
CNAG_05679	2130.506	-0.31815	0.069962	-4.54744	5.43E-06	2.51E-05
CNAG_05683	201.367	-0.70774	0.248515	-2.84789	0.004401	0.011157
CNAG_05685	1186.61	0.415377	0.088009	4.719699	2.36E-06	1.15E-05
CNAG_12663	111.422	-0.8765	0.314099	-2.79051	0.005262	0.013088
CNAG_05686	558.6661	-0.28601	0.115179	-2.48322	0.01302	0.029241
CNAG_05688	1066.846	-0.44291	0.08064	-5.49247	3.96E-08	2.43E-07
CNAG_05689	1469.287	-0.39981	0.088611	-4.51196	6.42E-06	2.94E-05
CNAG_05690	3600.809	-0.24467	0.060771	-4.02607	5.67E-05	0.000217
CNAG_05692	1185.242	-0.45946	0.081346	-5.64819	1.62E-08	1.04E-07

CNAG_05694	4494.972	-0.1908	0.071414	-2.67168	0.007547	0.018
CNAG_05695	4425.786	0.222124	0.064345	3.452099	0.000556	0.00174
CNAG_05697	1147.595	0.446853	0.086002	5.19582	2.04E-07	1.14E-06
CNAG_05698	876.847	-0.33959	0.09342	-3.63509	0.000278	0.000927
CNAG_05699	1004.797	0.395428	0.095801	4.127607	3.67E-05	0.000145
CNAG_05700	1869.737	-0.20627	0.06981	-2.95476	0.003129	0.008228
CNAG_12667	198.0589	0.801316	0.227067	3.52899	0.000417	0.00134
CNAG_05702	1258.888	-0.21674	0.082982	-2.61187	0.009005	0.021117
CNAG_05703	2187.043	-0.18797	0.072178	-2.60431	0.009206	0.021527
CNAG_05705	486.2795	-0.32543	0.124746	-2.6087	0.009089	0.021295
CNAG_05707	736.017	0.391049	0.112835	3.465662	0.000529	0.001657
CNAG_05708	1422.371	-0.44722	0.089503	-4.99672	5.83E-07	3.09E-06
CNAG_12668	95.77436	-1.13111	0.228195	-4.95677	7.17E-07	3.76E-06
CNAG_05711	769.964	-0.48295	0.109697	-4.40259	1.07E-05	4.68E-05
CNAG_05714	1266.001	-0.31126	0.085106	-3.65729	0.000255	0.000857
CNAG_05715	893.9332	-0.40047	0.104941	-3.81619	0.000136	0.000482
CNAG_05717	358.4681	-0.53145	0.135422	-3.9244	8.69E-05	0.000323
CNAG_05719	900.2185	-0.19596	0.094987	-2.06305	0.039108	0.075919
CNAG_05720	1462.305	-0.50723	0.07934	-6.39311	1.63E-10	1.28E-09
CNAG_05721	1116.409	0.244735	0.081503	3.002782	0.002675	0.007148
CNAG_05722	4170.526	0.272164	0.061867	4.399186	1.09E-05	4.74E-05
CNAG_05724	1546.485	1.019672	0.082057	12.42631	1.88E-35	4.48E-34
CNAG_05725	51171.06	-0.19715	0.056256	-3.50454	0.000457	0.001454
CNAG_12670	67.92234	0.639563	0.257282	2.485848	0.012924	0.029051
CNAG_05727	33.58321	2.346283	0.408969	5.737068	9.63E-09	6.35E-08
CNAG_05731	1677.538	0.68805	0.076514	8.992524	2.42E-19	3.32E-18
CNAG_05732	1713.358	-1.07287	0.112951	-9.49857	2.13E-21	3.24E-20
CNAG_05737	1300.338	-0.41287	0.091394	-4.51748	6.26E-06	2.87E-05
CNAG_05738	130.3272	1.587178	0.266107	5.964439	2.45E-09	1.73E-08
CNAG_05740	535.3075	-0.30864	0.126258	-2.44456	0.014503	0.032039
CNAG_05742	1343.683	-0.48152	0.084722	-5.68357	1.32E-08	8.52E-08
CNAG_05743	478.3062	-0.25576	0.106554	-2.40032	0.016381	0.035638
CNAG_05744	660.2837	-0.32266	0.099901	-3.22978	0.001239	0.003561
CNAG_05747	809.4054	-0.21355	0.09568	-2.23194	0.025619	0.052637
CNAG_05749	786.0784	-0.33285	0.108366	-3.07156	0.002129	0.005816
CNAG_05750	70535.33	-0.13882	0.069135	-2.00798	0.044645	0.085182
CNAG_05751	1386.388	-0.23309	0.11082	-2.10332	0.035438	0.069575
CNAG_05752	2369.488	-0.36354	0.086176	-4.21852	2.46E-05	9.98E-05
CNAG_05753	9648.222	-0.12539	0.060836	-2.06113	0.03929	0.076236
CNAG_07672	2175.701	0.391972	0.078834	4.972124	6.62E-07	3.49E-06
CNAG_05755	3201.566	0.173287	0.070561	2.455839	0.014056	0.031197
CNAG_05756	1564.075	0.249691	0.100404	2.486864	0.012887	0.029
CNAG_05757	942.5591	-0.6881	0.099316	-6.9284	4.26E-12	3.85E-11
CNAG_05758	1002.13	-0.24862	0.090017	-2.76187	0.005747	0.014147
CNAG_05759	10944.13	-0.23881	0.065932	-3.622	0.000292	0.000971
CNAG_12675	94.8963	1.149169	0.226515	5.073265	3.91E-07	2.12E-06
CNAG_05760	32.28191	0.776522	0.376984	2.059828	0.039415	0.076459

CNAG_05761	1178.286	0.194691	0.088697	2.195015	0.028162	0.057198
CNAG_05762	65203.54	-0.23426	0.075353	-3.10888	0.001878	0.005213
CNAG_05763	516.9623	0.507744	0.107752	4.712142	2.45E-06	1.19E-05
CNAG_05764	2973.673	-0.36787	0.080322	-4.57988	4.65E-06	2.17E-05
CNAG_05768	1607.972	-0.17048	0.080317	-2.12259	0.033789	0.066821
CNAG_05769	938.0488	-0.25329	0.085738	-2.95422	0.003135	0.00824
CNAG_05771	1167.274	0.205051	0.091015	2.25294	0.024263	0.050193
CNAG_05774	813.9959	-0.26069	0.091539	-2.84791	0.004401	0.011157
CNAG_05776	1137.759	-0.23943	0.087516	-2.73579	0.006223	0.015149
CNAG_05777	351.8169	-0.29998	0.125952	-2.38169	0.017233	0.037224
CNAG_05778	715.7151	0.44942	0.097681	4.600873	4.21E-06	1.98E-05
CNAG_05782	3058.462	-0.48672	0.071513	-6.80601	1.00E-11	8.89E-11
CNAG_05783	4364.034	0.405535	0.064662	6.271599	3.57E-10	2.73E-09
CNAG_05784	277.2093	-0.54569	0.158545	-3.44184	0.000578	0.001801
CNAG_12679	54.1046	1.430673	0.297479	4.809327	1.51E-06	7.57E-06
CNAG_05786	364.8285	-0.43383	0.123183	-3.52185	0.000429	0.001372
CNAG_05788	1693.099	-0.22375	0.091131	-2.4553	0.014077	0.031235
CNAG_05789	2268.328	0.289106	0.078345	3.690172	0.000224	0.000762
CNAG_05790	1562.683	0.662679	0.079601	8.324956	8.44E-17	1.02E-15
CNAG_05793	888.9825	-0.50974	0.08624	-5.91073	3.41E-09	2.37E-08
CNAG_07675	1082.649	0.24148	0.0963	2.507587	0.012156	0.027483
CNAG_05797	4457.83	-0.15132	0.064007	-2.36415	0.018072	0.038838
CNAG_05798	1203.501	-0.3355	0.100814	-3.32793	0.000875	0.002613
CNAG_05799	6608.682	0.356063	0.063036	5.648551	1.62E-08	1.04E-07
CNAG_05802	276.3479	0.980823	0.138275	7.093284	1.31E-12	1.24E-11
CNAG_05803	2434.174	1.015321	0.068432	14.837	8.44E-50	2.87E-48
CNAG_05804	2993.716	0.129836	0.064243	2.02102	0.043278	0.082884
CNAG_05807	440.0747	0.621075	0.122408	5.073824	3.90E-07	2.12E-06
CNAG_05809	2911.465	0.410097	0.069006	5.942908	2.80E-09	1.96E-08
CNAG_05810	532.2629	-0.2809	0.106405	-2.63992	0.008293	0.019559
CNAG_05811	768.768	-0.20236	0.092264	-2.19324	0.02829	0.057414
CNAG_05812	1062.79	-0.34823	0.085001	-4.09676	4.19E-05	0.000165
CNAG_05814	39991.86	-0.18197	0.069732	-2.60958	0.009065	0.021253
CNAG_05815	306.5484	0.70594	0.168437	4.191122	2.78E-05	0.000112
CNAG_05817	8240.169	0.467146	0.065205	7.164303	7.82E-13	7.53E-12
CNAG_05818	2394.86	1.566144	0.07544	20.76009	9.94E-96	6.90E-94
CNAG_05819	1050.591	-0.27069	0.095804	-2.8255	0.004721	0.011861
CNAG_05820	424.1291	0.624382	0.119348	5.23161	1.68E-07	9.56E-07
CNAG_12683	349.0375	0.54754	0.135089	4.053191	5.05E-05	0.000196
CNAG_05821	32.22518	0.781347	0.398088	1.962749	0.049675	0.093161
CNAG_05822	1487.059	-0.31597	0.080292	-3.93524	8.31E-05	0.00031
CNAG_05825	4056.606	-0.18334	0.063283	-2.89717	0.003765	0.009707
CNAG_05826	6645.308	-0.24801	0.066939	-3.70503	0.000211	0.000724
CNAG_05828	2510.708	-0.1965	0.095741	-2.05243	0.040128	0.077675
CNAG_05829	2166.484	-0.5151	0.074905	-6.87668	6.13E-12	5.49E-11
CNAG_05831	301.102	-0.68217	0.139785	-4.88015	1.06E-06	5.41E-06
CNAG_05835	1560.033	-0.58866	0.079166	-7.43584	1.04E-13	1.06E-12

CNAG_05836	1626.726	0.218491	0.093909	2.326626	0.019985	0.042368
CNAG_05839	11065.22	0.463559	0.070376	6.586915	4.49E-11	3.74E-10
CNAG_05841	699.2273	0.417923	0.11448	3.650629	0.000262	0.000876
CNAG_05843	936.2309	0.581679	0.086807	6.700852	2.07E-11	1.77E-10
CNAG_05845	2958.892	-0.24944	0.070356	-3.54539	0.000392	0.001266
CNAG_05847	41109.52	3.385007	0.063693	53.14585	0	0
CNAG_05848	5642.634	-0.49111	0.080152	-6.12725	8.94E-10	6.55E-09
CNAG_07676	17621.73	0.332906	0.056382	5.904472	3.54E-09	2.46E-08
CNAG_07677	406.2587	0.567079	0.133754	4.239708	2.24E-05	9.17E-05
CNAG_05851	1420.216	0.53519	0.079514	6.730798	1.69E-11	1.46E-10
CNAG_05852	1487.308	0.581924	0.076457	7.611124	2.72E-14	2.88E-13
CNAG_05853	787.7304	-0.1941	0.09884	-1.96375	0.049559	0.093001
CNAG_05855	1174.82	-0.33368	0.104005	-3.20833	0.001335	0.003813
CNAG_07678	150.6252	-0.50598	0.205115	-2.46682	0.013632	0.030398
CNAG_07679	1760.93	-0.40743	0.091671	-4.4445	8.81E-06	3.93E-05
CNAG_05859	504.4987	-0.52313	0.12304	-4.25174	2.12E-05	8.76E-05
CNAG_05860	432.6775	-0.39381	0.126555	-3.1118	0.00186	0.005167
CNAG_05862	549.7671	-0.29937	0.119072	-2.5142	0.01193	0.027053
CNAG_05863	861.1287	-1.01301	0.113846	-8.89811	5.68E-19	7.63E-18
CNAG_05864	2273.278	1.081369	0.119744	9.030692	1.71E-19	2.36E-18
CNAG_05866	483.6915	0.784715	0.113888	6.890225	5.57E-12	5.00E-11
CNAG_05867	420.3886	-0.96607	0.149871	-6.44601	1.15E-10	9.22E-10
CNAG_05868	194.6641	0.731428	0.157154	4.654205	3.25E-06	1.55E-05
CNAG_05870	91.03128	-0.94269	0.260283	-3.62177	0.000293	0.000971
CNAG_05871	717.9209	-0.52096	0.106747	-4.88032	1.06E-06	5.41E-06
CNAG_05872	2610.036	1.59104	0.269503	5.903614	3.56E-09	2.46E-08
CNAG_05874	1122.582	0.463833	0.127859	3.627682	0.000286	0.000951
CNAG_05875	3754.714	1.758624	0.070777	24.84756	2.75E-136	2.94E-134
CNAG_05876	573.2032	0.985799	0.11561	8.526928	1.50E-17	1.87E-16
CNAG_05878	5295.487	-0.12985	0.063424	-2.04735	0.040624	0.078499
CNAG_05880	724.1231	0.572031	0.103034	5.55187	2.83E-08	1.77E-07
CNAG_05881	4570.707	0.8177	0.059778	13.67894	1.36E-42	3.98E-41
CNAG_05884	10099.01	0.462766	0.057027	8.114824	4.86E-16	5.68E-15
CNAG_05886	2291.617	-0.31067	0.08076	-3.8469	0.00012	0.000432
CNAG_05887	1939.651	-0.4194	0.077786	-5.39167	6.98E-08	4.18E-07
CNAG_05888	1813.212	-0.44861	0.077242	-5.80791	6.33E-09	4.28E-08
CNAG_05889	1080.509	-0.56791	0.107017	-5.30673	1.12E-07	6.49E-07
CNAG_05892	2617.552	-0.40041	0.075624	-5.29479	1.19E-07	6.91E-07
CNAG_05893	6325.749	-0.13974	0.067103	-2.08252	0.037295	0.072851
CNAG_05894	2588.161	0.372524	0.068365	5.449042	5.06E-08	3.08E-07
CNAG_05895	3190.159	-0.15547	0.063037	-2.4663	0.013652	0.030433
CNAG_05898	545.1958	-0.56843	0.13416	-4.23697	2.27E-05	9.27E-05
CNAG_05900	8936.938	-0.12363	0.055979	-2.20857	0.027205	0.055516
CNAG_05901	606.1403	-0.32343	0.105941	-3.05291	0.002266	0.006161
CNAG_05902	499.4046	-0.33335	0.131464	-2.53567	0.011223	0.025668
CNAG_12695	102.3212	-0.56383	0.256277	-2.20008	0.027801	0.056549
CNAG_05903	954.8289	-0.39609	0.089652	-4.41812	9.96E-06	4.39E-05

CNAG_05904	32431.53	-0.17078	0.078978	-2.16234	0.030592	0.061381
CNAG_05905	1289.939	0.237952	0.086468	2.751905	0.005925	0.014505
CNAG_05907	16721.5	0.334193	0.063734	5.243593	1.57E-07	9.00E-07
CNAG_05908	168.0279	0.486155	0.168474	2.885639	0.003906	0.010022
CNAG_12696	33.23852	1.121845	0.373287	3.005316	0.002653	0.007093
CNAG_05909	25433.97	0.460848	0.066888	6.889796	5.59E-12	5.01E-11
CNAG_05911	150.9264	-0.64991	0.176195	-3.68857	0.000226	0.000767
CNAG_12698	256.4066	-0.56998	0.150258	-3.79331	0.000149	0.000525
CNAG_05912	932.0002	0.200283	0.085537	2.34147	0.019208	0.040913
CNAG_05913	1909.699	1.656487	0.086603	19.12729	1.50E-81	8.39E-80
CNAG_05914	379.2217	1.01494	0.144635	7.017261	2.26E-12	2.11E-11
CNAG_05915	388.4839	0.511617	0.140402	3.643946	0.000268	0.000898
CNAG_05921	761.2049	-0.27488	0.090655	-3.03216	0.002428	0.006561
CNAG_05926	1967.517	-0.31569	0.073134	-4.31668	1.58E-05	6.69E-05
CNAG_05927	1394.8	-0.41942	0.074605	-5.62183	1.89E-08	1.20E-07
CNAG_05929	360.0313	-0.2944	0.131436	-2.23987	0.025099	0.0517
CNAG_05930	1069.307	-0.399	0.086399	-4.61815	3.87E-06	1.83E-05
CNAG_05931	2284.725	-0.30765	0.07704	-3.99336	6.51E-05	0.000247
CNAG_05932	7829.644	0.140639	0.064622	2.176341	0.02953	0.059558
CNAG_05937	736.6723	0.349357	0.102208	3.418103	0.000631	0.001948
CNAG_12702	51.87046	1.730859	0.34303	5.0458	4.52E-07	2.43E-06
CNAG_07685	353.3726	0.46253	0.141109	3.277811	0.001046	0.003063
CNAG_07686	1957.716	0.160172	0.072503	2.209186	0.027162	0.055442
CNAG_12705	7.413872	3.859265	1.118053	3.451772	0.000557	0.00174
CNAG_07687	139.2701	1.861156	0.247829	7.50984	5.92E-14	6.17E-13
CNAG_07689	3361.548	-0.29043	0.067201	-4.32179	1.55E-05	6.55E-05
CNAG_07690	430.3046	-0.30917	0.126408	-2.44585	0.014451	0.031956
CNAG_07691	152.7546	1.042312	0.190672	5.466518	4.59E-08	2.80E-07
CNAG_12707	27.59879	2.15741	0.456919	4.721642	2.34E-06	1.14E-05
CNAG_07188	326.0514	1.089742	0.155475	7.009103	2.40E-12	2.22E-11
CNAG_07992	41.59829	1.039566	0.338185	3.073956	0.002112	0.005779
CNAG_07693	15.92936	1.254194	0.59141	2.120686	0.033948	0.067069
CNAG_07694	2117.929	-0.23942	0.07124	-3.36071	0.000777	0.002351
CNAG_07695	7572.613	0.835097	0.070615	11.82609	2.86E-32	6.19E-31
CNAG_07703	231.556	-0.39573	0.169136	-2.33971	0.019299	0.041063
CNAG_05964	106.0095	-0.49109	0.221375	-2.21835	0.026531	0.054305
CNAG_05965	368.4091	-0.49222	0.140163	-3.51176	0.000445	0.00142
CNAG_05967	412.5318	-0.81197	0.147639	-5.49972	3.80E-08	2.34E-07
CNAG_05969	471.2135	-0.311	0.157021	-1.98063	0.047632	0.089995
CNAG_05970	476.0293	-0.28221	0.117802	-2.39564	0.016592	0.036058
CNAG_12712	445.7508	0.259605	0.108743	2.387326	0.016971	0.036726
CNAG_12713	75.23501	2.714783	0.291088	9.32633	1.10E-20	1.61E-19
CNAG_05975	4129.714	0.149901	0.06381	2.349165	0.018816	0.040203
CNAG_05976	5689.809	0.20728	0.063799	3.248973	0.001158	0.003357
CNAG_05977	4659.428	0.265495	0.067771	3.9175	8.95E-05	0.000332
CNAG_05979	962.4853	0.199406	0.100754	1.979138	0.0478	0.090228
CNAG_05980	4357.644	-0.32648	0.074171	-4.40177	1.07E-05	4.70E-05

CNAG_05981	471.021	-0.56383	0.118252	-4.76805	1.86E-06	9.20E-06
CNAG_07706	529.0163	0.271525	0.111016	2.445809	0.014453	0.031956
CNAG_03084	472.2164	2.572927	0.351177	7.326582	2.36E-13	2.36E-12
CNAG_03085	68.45281	4.038878	0.417125	9.682657	3.57E-22	5.56E-21
CNAG_03086	1120.436	0.287917	0.104766	2.748184	0.005993	0.014645
CNAG_03090	273.176	0.834369	0.161034	5.181319	2.20E-07	1.23E-06
CNAG_03092	951.1183	-0.25601	0.093716	-2.73171	0.006301	0.015319
CNAG_12715	39.7449	-1.39589	0.345071	-4.04523	5.23E-05	0.000201
CNAG_03097	507.9971	-0.35325	0.123337	-2.86412	0.004182	0.010662
CNAG_03101	439.2993	0.497169	0.115631	4.299631	1.71E-05	7.19E-05
CNAG_03106	2055.659	-0.26008	0.082477	-3.15335	0.001614	0.004533
CNAG_03108	798.463	0.377504	0.101683	3.712567	0.000205	0.000705
CNAG_03110	2130.182	0.846424	0.090012	9.403442	5.28E-21	7.90E-20
CNAG_03115	1546.139	1.646773	0.088208	18.66926	8.81E-78	4.74E-76
CNAG_07711	49.88375	1.820482	0.371057	4.906204	9.29E-07	4.78E-06
CNAG_12718	77.17537	1.667077	0.264758	6.296615	3.04E-10	2.35E-09
CNAG_12719	4.623654	3.757957	1.369686	2.743663	0.006076	0.014827
CNAG_12720	135.0057	0.518639	0.218304	2.375764	0.017513	0.037747
CNAG_03116	2208.586	-0.32456	0.077347	-4.19616	2.71E-05	0.00011
CNAG_03120	9257.024	0.331448	0.072013	4.602613	4.17E-06	1.96E-05
CNAG_03121	1516.529	-0.21916	0.076434	-2.86733	0.004139	0.010567
CNAG_03123	743.3144	-0.502	0.093444	-5.37223	7.78E-08	4.64E-07
CNAG_03125	603.8943	-0.40399	0.097366	-4.14914	3.34E-05	0.000133
CNAG_03127	48643.23	-0.13852	0.068282	-2.02869	0.04249	0.081549
CNAG_03128	2926.277	2.854045	0.089548	31.87153	6.62E-223	1.63E-220
CNAG_03130	834.8777	-0.43975	0.102698	-4.28197	1.85E-05	7.73E-05
CNAG_03132	47.4629	1.640332	0.348313	4.709357	2.48E-06	1.21E-05
CNAG_03133	802.6362	2.121197	0.129317	16.40304	1.82E-60	7.78E-59
CNAG_03135	1103.441	0.229359	0.099009	2.316541	0.020529	0.043261
CNAG_03136	2394.845	-0.27629	0.076824	-3.59644	0.000323	0.001062
CNAG_12722	47.65008	1.055901	0.314407	3.35839	0.000784	0.002369
CNAG_03139	4207.567	-0.49446	0.07654	-6.4602	1.05E-10	8.46E-10
CNAG_12723	20.80785	2.707543	0.536043	5.05098	4.40E-07	2.37E-06
CNAG_03142	8060.698	-0.24056	0.121986	-1.972	0.048609	0.091458
CNAG_03146	12868.05	0.440451	0.065629	6.711259	1.93E-11	1.65E-10
CNAG_03148	2694.374	-0.35267	0.077888	-4.52794	5.96E-06	2.74E-05
CNAG_03150	1515.206	0.657046	0.079708	8.243186	1.68E-16	2.00E-15
CNAG_03151	887.8501	0.291385	0.094714	3.076483	0.002095	0.005734
CNAG_03152	1755.276	0.182311	0.076573	2.380859	0.017272	0.037268
CNAG_03153	8137.128	-0.1656	0.062098	-2.66681	0.007657	0.018231
CNAG_03154	1198.846	1.940473	0.098907	19.61911	1.06E-85	6.39E-84
CNAG_12726	39.04536	0.822126	0.378704	2.170894	0.029939	0.060249
CNAG_03156	1724.607	1.224475	0.089159	13.73363	6.39E-43	1.89E-41
CNAG_03158	1110.154	-0.18461	0.079858	-2.31169	0.020795	0.043754
CNAG_03159	1218.617	-0.25672	0.097831	-2.62413	0.008687	0.020413
CNAG_03161	354.4363	0.462404	0.125556	3.682863	0.000231	0.000782
CNAG_03163	503.978	0.320386	0.105774	3.028963	0.002454	0.006615

CNAG_03166	210.3726	-0.45651	0.193605	-2.35795	0.018376	0.039358
CNAG_03167	1345.407	-0.28204	0.091174	-3.0934	0.001979	0.005465
CNAG_03168	12291.89	0.387368	0.063184	6.130798	8.74E-10	6.42E-09
CNAG_12730	37.07372	1.356664	0.382081	3.550726	0.000384	0.001244
CNAG_03170	1179.129	0.719691	0.081107	8.873363	7.10E-19	9.46E-18
CNAG_03172	552.3662	0.334237	0.118723	2.815267	0.004874	0.012211
CNAG_12731	51.12334	2.013837	0.378058	5.32679	1.00E-07	5.85E-07
CNAG_03173	1570.343	0.75236	0.107829	6.977309	3.01E-12	2.75E-11
CNAG_03176	2228.251	1.057545	0.085607	12.35343	4.67E-35	1.10E-33
CNAG_12733	337.9669	0.559522	0.175387	3.190209	0.001422	0.004036
CNAG_03178	798.8059	-0.32343	0.10008	-3.23172	0.00123	0.003539
CNAG_03182	1677.029	-0.19647	0.07434	-2.64287	0.008221	0.019421
CNAG_03183	2724.746	0.389278	0.077626	5.014778	5.31E-07	2.84E-06
CNAG_03185	980.1492	0.736274	0.098655	7.4631	8.45E-14	8.72E-13
CNAG_03186	2560.752	-0.52987	0.075421	-7.02541	2.13E-12	1.99E-11
CNAG_03187	452.3346	0.333234	0.117912	2.826128	0.004711	0.011845
CNAG_03190	3683.652	-0.16609	0.068921	-2.40991	0.015956	0.034789
CNAG_03191	1529.28	-0.32625	0.074125	-4.4014	1.08E-05	4.70E-05
CNAG_03192	406.758	-0.41129	0.118541	-3.46963	0.000521	0.001636
CNAG_03193	34.83883	-0.72958	0.356564	-2.04614	0.040743	0.078669
CNAG_03194	121.3339	-0.99057	0.245831	-4.02948	5.59E-05	0.000215
CNAG_03196	2432.824	-0.37869	0.080211	-4.72122	2.34E-06	1.14E-05
CNAG_12735	14.5142	1.272471	0.609116	2.089044	0.036704	0.071852
CNAG_03199	133.6702	0.736633	0.244699	3.010358	0.002609	0.006988
CNAG_03204	265.9572	0.776903	0.155543	4.994795	5.89E-07	3.12E-06
CNAG_03205	2275.43	0.15229	0.072248	2.10788	0.035041	0.068913
CNAG_03206	857.2381	0.821438	0.087276	9.411954	4.87E-21	7.30E-20
CNAG_03210	992.1379	-0.31637	0.100077	-3.1613	0.001571	0.004419
CNAG_03212	829.9802	-0.3518	0.115654	-3.04181	0.002352	0.006369
CNAG_03216	1061.717	0.556778	0.09896	5.626275	1.84E-08	1.17E-07
CNAG_07720	1793.486	-0.26609	0.07853	-3.38843	0.000703	0.00215
CNAG_03222	522.9654	0.54727	0.112723	4.854977	1.20E-06	6.10E-06
CNAG_03223	5370.501	0.76422	0.082635	9.248142	2.28E-20	3.30E-19
CNAG_12739	57.45058	1.636411	0.312083	5.243509	1.58E-07	9.00E-07
CNAG_03224	40.78848	1.05222	0.370944	2.836599	0.00456	0.011506
CNAG_03225	14410.01	-0.90662	0.072837	-12.4471	1.45E-35	3.47E-34
CNAG_03226	10612.54	1.570201	0.060347	26.01961	2.97E-149	3.77E-147
CNAG_12740	185.5085	1.594283	0.17406	9.159408	5.22E-20	7.39E-19
CNAG_03227	500.0812	0.250996	0.112559	2.229904	0.025754	0.052887
CNAG_03229	26.80175	-3.7031	0.566338	-6.53867	6.21E-11	5.10E-10
CNAG_12741	7.414266	-4.34142	1.245717	-3.48508	0.000492	0.001552
CNAG_03230	55.70652	1.121004	0.302029	3.711572	0.000206	0.000708
CNAG_03231	438.3286	-0.25817	0.129693	-1.99061	0.046524	0.088169
CNAG_03232	1015.163	-0.25346	0.114683	-2.21011	0.027097	0.055352
CNAG_03234	160.1105	1.461958	0.222224	6.57875	4.74E-11	3.94E-10
CNAG_03235	2507.679	-0.28041	0.073068	-3.83775	0.000124	0.000446
CNAG_03237	694.1036	0.236872	0.11214	2.112287	0.034662	0.068249

CNAG_03238	181.8372	2.722762	0.188857	14.41708	4.04E-47	1.29E-45
CNAG_03239	629.1135	-0.35245	0.104607	-3.36932	0.000754	0.00229
CNAG_03240	2710.473	0.181445	0.066457	2.730266	0.006328	0.015373
CNAG_03245	9298.725	-0.17648	0.064524	-2.73503	0.006238	0.015179
CNAG_03246	1751.861	0.208758	0.095268	2.191276	0.028432	0.057673
CNAG_03247	2369.973	0.248574	0.07112	3.495133	0.000474	0.001501
CNAG_03248	1615.98	-0.41958	0.078215	-5.36441	8.12E-08	4.83E-07
CNAG_03249	5288.497	-0.39334	0.063187	-6.22504	4.81E-10	3.63E-09
CNAG_03251	15235.79	-0.13864	0.068275	-2.03062	0.042293	0.081229
CNAG_12745	15.21748	-1.93327	0.58066	-3.32944	0.00087	0.002602
CNAG_12746	6.182892	-2.28205	0.935287	-2.43995	0.014689	0.032389
CNAG_12748	65.0347	-1.22443	0.284082	-4.31013	1.63E-05	6.87E-05
CNAG_03259	131.8268	1.153353	0.224919	5.127864	2.93E-07	1.62E-06
CNAG_03261	3535.215	-0.24791	0.066025	-3.75481	0.000173	0.000606
CNAG_03262	2053.874	-0.27903	0.071594	-3.89744	9.72E-05	0.000357
CNAG_03263	14582.29	0.267896	0.062117	4.312786	1.61E-05	6.79E-05
CNAG_03266	5218.36	-0.25382	0.068445	-3.70839	0.000209	0.000715
CNAG_03268	2649.046	-0.70411	0.104727	-6.72332	1.78E-11	1.53E-10
CNAG_03269	1187.77	-0.25104	0.086463	-2.90347	0.00369	0.009529
CNAG_03271	3464.009	-0.25168	0.064667	-3.89199	9.94E-05	0.000365
CNAG_03273	2191.356	-0.19374	0.079973	-2.42255	0.015412	0.033765
CNAG_03275	914.4967	0.222039	0.104147	2.131976	0.033009	0.06555
CNAG_03279	532.3013	0.83815	0.117194	7.151789	8.57E-13	8.23E-12
CNAG_03280	978.4387	0.319849	0.094941	3.368941	0.000755	0.002293
CNAG_03281	24387.14	-0.23612	0.059653	-3.95816	7.55E-05	0.000284
CNAG_03282	1328.539	-0.37596	0.084208	-4.46469	8.02E-06	3.60E-05
CNAG_03283	38806.68	-0.31202	0.067495	-4.62292	3.78E-06	1.79E-05
CNAG_03284	1599.634	0.290964	0.078085	3.726263	0.000194	0.000672
CNAG_03288	1348.69	-0.60253	0.079018	-7.62526	2.44E-14	2.60E-13
CNAG_03289	5837.225	0.226987	0.059148	3.837598	0.000124	0.000446
CNAG_03290	1207.076	-0.20506	0.079835	-2.56849	0.010214	0.023525
CNAG_03291	1564.102	0.23745	0.079211	2.997683	0.00272	0.007264
CNAG_03296	607.0495	-0.47238	0.11834	-3.9917	6.56E-05	0.000249
CNAG_03297	2302.667	-0.38581	0.08356	-4.61721	3.89E-06	1.84E-05
CNAG_03298	4476.919	-0.24297	0.108581	-2.23767	0.025242	0.051942
CNAG_03300	2506.499	-0.1491	0.075349	-1.97873	0.047847	0.090295
CNAG_03303	17906.41	-0.15856	0.073553	-2.15576	0.031102	0.062297
CNAG_12753	234.5905	-0.37103	0.155315	-2.38892	0.016898	0.036597
CNAG_03306	775.9346	-0.65394	0.104804	-6.23963	4.39E-10	3.33E-09
CNAG_03308	1399.8	0.381929	0.086833	4.398415	1.09E-05	4.75E-05
CNAG_03309	1765.774	-0.69305	0.095193	-7.28045	3.33E-13	3.31E-12
CNAG_03314	1216.798	0.4491	0.100879	4.451864	8.51E-06	3.80E-05
CNAG_03316	2156.581	0.276205	0.067685	4.08076	4.49E-05	0.000175
CNAG_03317	2354.664	-0.32835	0.069444	-4.72828	2.26E-06	1.11E-05
CNAG_03318	198.9116	1.890252	0.166109	11.37958	5.29E-30	1.09E-28
CNAG_03319	3174.858	-0.36849	0.098029	-3.75902	0.000171	0.000597
CNAG_03321	2156.737	-0.16467	0.081925	-2.00995	0.044437	0.084884

CNAG_03323	3840.949	-0.32007	0.089885	-3.56088	0.00037	0.001203
CNAG_03328	1355.372	-0.48173	0.107877	-4.46555	7.99E-06	3.59E-05
CNAG_03329	823.6213	-0.25089	0.099892	-2.5116	0.012019	0.027196
CNAG_03331	2216.382	-0.47924	0.073112	-6.55484	5.57E-11	4.61E-10
CNAG_03335	4562.354	0.341912	0.058114	5.883445	4.02E-09	2.77E-08
CNAG_03337	1333.135	0.275728	0.089385	3.084712	0.002037	0.005597
CNAG_03342	3742.511	0.145545	0.064936	2.241374	0.025002	0.051551
CNAG_03343	1060.202	-0.19055	0.09318	-2.04495	0.04086	0.078839
CNAG_03345	4098.498	0.293502	0.077019	3.810769	0.000139	0.000492
CNAG_07723	59.76205	-1.03806	0.294589	-3.52374	0.000425	0.001364
CNAG_03346	412.6671	-1.81934	0.125492	-14.4977	1.25E-47	4.03E-46
CNAG_03347	2122.64	0.747257	0.106533	7.014306	2.31E-12	2.15E-11
CNAG_03349	4086.508	-0.19056	0.068165	-2.7955	0.005182	0.012916
CNAG_07724	1825.89	-0.38544	0.098779	-3.90206	9.54E-05	0.000352
CNAG_03352	1020.417	0.390991	0.093086	4.200323	2.67E-05	0.000108
CNAG_03353	2411.298	-0.19492	0.073338	-2.65786	0.007864	0.018651
CNAG_03354	856.1046	0.224065	0.10084	2.221993	0.026284	0.053867
CNAG_03355	5350.264	0.534478	0.071904	7.433227	1.06E-13	1.08E-12
CNAG_03356	695.4869	-0.37048	0.096185	-3.85179	0.000117	0.000423
CNAG_03357	1210.892	-0.18577	0.088746	-2.09325	0.036327	0.071182
CNAG_03358	27827.09	0.679391	0.053206	12.76904	2.44E-37	6.18E-36
CNAG_03359	2966.502	0.715945	0.095822	7.471621	7.92E-14	8.20E-13
CNAG_03361	581.8787	-0.21229	0.098077	-2.16455	0.030422	0.061114
CNAG_03363	1859.21	1.311373	0.085772	15.28901	9.05E-53	3.27E-51
CNAG_03365	1742.146	-0.41089	0.077689	-5.28892	1.23E-07	7.12E-07
CNAG_03366	463.8954	0.577908	0.115951	4.984073	6.23E-07	3.28E-06
CNAG_12760	18.15565	1.077987	0.490766	2.19654	0.028053	0.057004
CNAG_03367	1364.645	-0.27657	0.075562	-3.66012	0.000252	0.00085
CNAG_12761	119.961	-0.80329	0.2002	-4.01245	6.01E-05	0.00023
CNAG_03370	4315.321	-0.46948	0.071731	-6.54499	5.95E-11	4.90E-10
CNAG_03372	1501.917	-0.22585	0.098922	-2.28313	0.022423	0.046815
CNAG_03373	1686.272	-0.25615	0.085653	-2.99049	0.002785	0.007415
CNAG_03375	5962.825	-0.7152	0.065493	-10.9202	9.23E-28	1.77E-26
CNAG_03376	1272.1	-0.26219	0.082938	-3.16134	0.00157	0.004419
CNAG_03378	2569.557	0.227516	0.069797	3.259665	0.001115	0.003244
CNAG_03380	738.4693	-0.22411	0.096616	-2.31962	0.020362	0.042998
CNAG_03381	675.8875	1.294686	0.115872	11.17339	5.50E-29	1.09E-27
CNAG_03382	1455.667	-0.40591	0.083398	-4.86718	1.13E-06	5.75E-06
CNAG_03386	2319.894	0.313796	0.069898	4.489325	7.14E-06	3.24E-05
CNAG_03389	5275.637	-0.19621	0.071079	-2.76045	0.005772	0.014187
CNAG_03390	1263.519	-0.15418	0.076856	-2.00606	0.04485	0.085513
CNAG_03391	719.4727	-0.30501	0.124003	-2.45973	0.013904	0.030903
CNAG_03392	455.9731	0.361378	0.124366	2.905768	0.003664	0.009468
CNAG_03395	4538.935	2.176032	0.078235	27.81388	2.95E-170	4.52E-168
CNAG_03397	2848.771	-0.14639	0.062948	-2.32551	0.020045	0.042467
CNAG_03400	315.9039	1.010238	0.140696	7.180293	6.96E-13	6.72E-12
CNAG_03401	1873.952	0.550971	0.074281	7.417349	1.19E-13	1.21E-12

CNAG_03405	2961.436	0.547496	0.072116	7.591891	3.15E-14	3.34E-13
CNAG_03406	648.5251	0.268457	0.098863	2.715434	0.006619	0.015997
CNAG_03407	32.41668	-0.87253	0.371589	-2.3481	0.018869	0.040297
CNAG_03413	1093.767	-0.71801	0.083904	-8.55754	1.15E-17	1.45E-16
CNAG_03416	1543.501	-0.2277	0.082219	-2.76947	0.005615	0.013859
CNAG_03419	1587.026	-0.28987	0.073693	-3.93341	8.37E-05	0.000312
CNAG_03420	1612.89	1.038702	0.082991	12.51589	6.11E-36	1.48E-34
CNAG_03421	154.1653	0.626173	0.191852	3.263837	0.001099	0.003199
CNAG_03425	1434.832	0.231369	0.102123	2.265579	0.023477	0.048766
CNAG_03426	1870.482	-0.93526	0.087546	-10.6831	1.22E-26	2.22E-25
CNAG_03427	536.1417	0.221985	0.102235	2.171323	0.029907	0.060199
CNAG_03431	773.2234	-0.44564	0.097648	-4.56373	5.03E-06	2.33E-05
CNAG_03432	1173.805	0.928347	0.119611	7.761356	8.40E-15	9.12E-14
CNAG_03434	1386.722	-0.41245	0.088089	-4.68219	2.84E-06	1.37E-05
CNAG_03435	9118.811	0.169206	0.070056	2.415309	0.015722	0.034333
CNAG_03436	2575.009	0.447285	0.079976	5.592728	2.24E-08	1.41E-07
CNAG_12770	865.544	-0.81705	0.124664	-6.55407	5.60E-11	4.62E-10
CNAG_07733	25.82767	1.726485	0.490378	3.520724	0.00043	0.001377
CNAG_03439	9211.138	-0.17998	0.061597	-2.92196	0.003478	0.00905
CNAG_03440	416.8385	-0.33483	0.116178	-2.88205	0.003951	0.010115
CNAG_03441	1493.923	-0.18803	0.07679	-2.44869	0.014338	0.031754
CNAG_03446	700.1372	-0.20856	0.10089	-2.06722	0.038713	0.075278
CNAG_03449	424.0807	0.321188	0.12023	2.671449	0.007552	0.018007
CNAG_03450	2694.892	1.278572	0.077074	16.58892	8.38E-62	3.66E-60
CNAG_03452	1668.031	0.448084	0.088637	5.055261	4.30E-07	2.32E-06
CNAG_03454	2250.633	-1.0415	0.106476	-9.78157	1.35E-22	2.14E-21
CNAG_03455	468.5504	0.743696	0.108362	6.863053	6.74E-12	6.03E-11
CNAG_03456	1801.692	-0.36476	0.080151	-4.5509	5.34E-06	2.48E-05
CNAG_03458	1790.094	-0.62452	0.072612	-8.60081	7.92E-18	1.00E-16
CNAG_03459	6044.185	-0.14608	0.066029	-2.21238	0.02694	0.055073
CNAG_03461	773.5077	2.087963	0.139771	14.93846	1.85E-50	6.35E-49
CNAG_03463	2863.365	0.292807	0.069236	4.22914	2.35E-05	9.57E-05
CNAG_03464	758.1376	3.402955	0.112538	30.23823	7.45E-201	1.48E-198
CNAG_12774	18.79915	2.833285	0.587649	4.82139	1.43E-06	7.14E-06
CNAG_12776	254.2729	-0.29896	0.147348	-2.02892	0.042467	0.081523
CNAG_12777	22.17856	2.240813	0.498757	4.492799	7.03E-06	3.20E-05
CNAG_07734	7.739304	3.986292	1.074392	3.710277	0.000207	0.000711
CNAG_03465	2525.417	2.261959	0.081305	27.82055	2.45E-170	3.83E-168
CNAG_03467	358.588	-0.27715	0.122241	-2.26727	0.023374	0.048576
CNAG_07736	755.2722	-1.55346	0.112328	-13.8297	1.69E-43	5.14E-42
CNAG_03473	481.8728	0.397535	0.107419	3.700773	0.000215	0.000735
CNAG_03475	2464.046	1.03465	0.07135	14.50101	1.19E-47	3.85E-46
CNAG_03476	10824.54	-0.16286	0.063307	-2.57254	0.010096	0.023285
CNAG_12781	163.517	-0.43153	0.178288	-2.42042	0.015503	0.033936
CNAG_03479	2366.2	-0.32126	0.082173	-3.90958	9.25E-05	0.000341
CNAG_03480	781.144	1.223538	0.113652	10.76563	5.00E-27	9.24E-26
CNAG_03481	1188.746	0.570882	0.080296	7.109703	1.16E-12	1.10E-11

CNAG_03482	48147.37	2.592493	0.054149	47.87699	0	0
CNAG_03483	1664.727	-0.22077	0.077083	-2.86401	0.004183	0.010662
CNAG_03484	1399.44	-0.82013	0.110419	-7.42744	1.11E-13	1.13E-12
CNAG_03485	2140.941	0.889583	0.094895	9.374414	6.96E-21	1.04E-19
CNAG_03486	4927.493	0.225494	0.076497	2.947752	0.003201	0.00839
CNAG_03489	878.2861	-0.45747	0.095474	-4.79161	1.65E-06	8.25E-06
CNAG_03492	2580.609	0.75579	0.311317	2.427723	0.015194	0.033368
CNAG_03493	528.4586	0.397373	0.114566	3.468504	0.000523	0.001641
CNAG_03494	1521.813	-0.2488	0.08888	-2.79925	0.005122	0.012775
CNAG_12786	327.8924	-0.84369	0.188066	-4.48611	7.25E-06	3.29E-05
CNAG_03498	7663.09	4.709352	0.083825	56.18106	0	0
CNAG_12787	85.92498	0.86896	0.249666	3.480485	0.000501	0.001576
CNAG_03502	3300.719	0.438271	0.103742	4.22463	2.39E-05	9.75E-05
CNAG_03503	931.7551	-0.34651	0.118366	-2.92742	0.003418	0.008904
CNAG_03508	1055.206	-0.40591	0.094656	-4.28828	1.80E-05	7.54E-05
CNAG_03509	4873.037	-0.23783	0.067348	-3.53128	0.000414	0.00133
CNAG_03510	26558.93	-0.31375	0.072304	-4.33925	1.43E-05	6.10E-05
CNAG_03511	316.895	1.047814	0.141014	7.430576	1.08E-13	1.10E-12
CNAG_03512	1729.447	0.284768	0.079322	3.590018	0.000331	0.001088
CNAG_12791	2.748698	3.001983	1.504295	1.995608	0.045977	0.087313
CNAG_07743	73.25834	0.742384	0.253828	2.924748	0.003447	0.008975
CNAG_12792	13.5	-1.62452	0.615964	-2.63736	0.008355	0.019696
CNAG_03520	250.0637	-0.44499	0.152609	-2.91588	0.003547	0.00919
CNAG_03522	256.6275	-0.38409	0.154625	-2.48398	0.012992	0.029188
CNAG_03524	3095.827	-0.6162	0.064326	-9.57937	9.76E-22	1.50E-20
CNAG_03526	265.1309	0.438979	0.156041	2.813227	0.004905	0.012274
CNAG_03533	1123.615	-0.54478	0.089843	-6.06367	1.33E-09	9.61E-09
CNAG_03534	328.2239	0.55876	0.131697	4.242779	2.21E-05	9.06E-05
CNAG_07744	2196.712	0.143679	0.070209	2.046455	0.040712	0.078637
CNAG_07745	4140.008	1.470661	0.094275	15.59974	7.31E-55	2.72E-53
CNAG_07746	10376.49	0.126943	0.058721	2.161799	0.030634	0.061444
CNAG_03539	1305.68	0.505807	0.078141	6.472995	9.61E-11	7.79E-10
CNAG_03541	1703.818	0.152187	0.073717	2.064459	0.038974	0.075713
CNAG_03542	1885.163	0.42797	0.080743	5.300384	1.16E-07	6.71E-07
CNAG_12795	332.0213	0.766334	0.142907	5.362459	8.21E-08	4.88E-07
CNAG_03543	3433.371	1.70896	0.094621	18.06103	6.46E-73	3.30E-71
CNAG_03544	7895.165	0.563882	0.070397	8.010031	1.15E-15	1.32E-14
CNAG_12796	26.98867	1.505479	0.46884	3.211069	0.001322	0.003779
CNAG_03550	339.9965	-0.66996	0.122092	-5.48734	4.08E-08	2.50E-07
CNAG_03552	1304.831	1.549863	0.095423	16.24197	2.55E-59	1.07E-57
CNAG_12799	20.08189	2.047167	0.506966	4.038074	5.39E-05	0.000207
CNAG_03553	5237.363	0.236812	0.068338	3.465298	0.00053	0.001659
CNAG_03555	482.949	0.253543	0.126855	1.998686	0.045642	0.08682
CNAG_03556	308.7895	-0.48272	0.147675	-3.26876	0.00108	0.003148
CNAG_03557	898.1946	0.202693	0.087616	2.313411	0.0207	0.043599
CNAG_03560	409.6252	1.338128	0.132311	10.11349	4.81E-24	8.12E-23
CNAG_03562	592.2024	0.36385	0.097792	3.720664	0.000199	0.000685

CNAG_03565	3590.595	0.943045	0.099284	9.49842	2.13E-21	3.24E-20
CNAG_03566	2614.121	-1.05468	0.244188	-4.31915	1.57E-05	6.62E-05
CNAG_03567	2733.325	0.300895	0.072268	4.163607	3.13E-05	0.000125
CNAG_03570	163.4919	1.83487	0.275903	6.650412	2.92E-11	2.48E-10
CNAG_12801	76.49322	2.401483	0.418616	5.736716	9.65E-09	6.36E-08
CNAG_03573	677.1761	0.766967	0.100062	7.664949	1.79E-14	1.92E-13
CNAG_03575	562.4521	-0.35021	0.107061	-3.27111	0.001071	0.003125
CNAG_03577	54884.43	-0.14643	0.059142	-2.47586	0.013291	0.029731
CNAG_03576	55.57131	-0.72064	0.29818	-2.4168	0.015657	0.034212
CNAG_03578	1510.129	0.686462	0.079623	8.621399	6.61E-18	8.39E-17
CNAG_07749	750.9711	-0.43258	0.091225	-4.74191	2.12E-06	1.04E-05
CNAG_03583	826.8051	-0.30439	0.098204	-3.09958	0.001938	0.00536
CNAG_03584	2364.431	-0.39946	0.080948	-4.93473	8.03E-07	4.17E-06
CNAG_07996	1016.919	-0.40099	0.082911	-4.83642	1.32E-06	6.65E-06
CNAG_03586	513.6189	-0.35198	0.105792	-3.32712	0.000877	0.002619
CNAG_03587	667.4555	-0.27866	0.108224	-2.57488	0.010027	0.023148
CNAG_03589	1769.42	0.364623	0.087023	4.189956	2.79E-05	0.000112
CNAG_03590	2565.712	-0.30527	0.086019	-3.54887	0.000387	0.001252
CNAG_03591	4956.451	-0.22495	0.065961	-3.4104	0.000649	0.001997
CNAG_07751	2455.128	2.49988	0.075991	32.89688	2.44E-237	6.60E-235
CNAG_12806	59.76047	4.831634	0.479281	10.081	6.70E-24	1.12E-22
CNAG_12807	126.0081	5.293662	1.124631	4.707022	2.51E-06	1.22E-05
CNAG_06977	141.5437	0.430764	0.20206	2.131861	0.033018	0.065553
CNAG_06978	293.1094	0.494608	0.157675	3.136879	0.001708	0.004769
CNAG_06993	3787.32	-0.29224	0.092969	-3.14339	0.00167	0.004676
CNAG_06994	982.8045	-0.3044	0.091339	-3.33261	0.00086	0.002577
CNAG_06966	38.04036	-1.84571	0.402529	-4.58528	4.53E-06	2.12E-05
CNAG_06967	2326.867	-0.46149	0.066552	-6.93427	4.08E-12	3.70E-11
CNAG_06968	1458.4	-0.82254	0.105042	-7.83062	4.85E-15	5.36E-14
CNAG_06969	996.9388	-0.59983	0.092518	-6.48341	8.97E-11	7.29E-10
CNAG_06942	540.7463	-0.58551	0.103852	-5.63787	1.72E-08	1.10E-07
CNAG_06943	222.7318	0.412769	0.145441	2.83805	0.004539	0.011461
CNAG_04100	118.7084	-0.75129	0.195836	-3.83631	0.000125	0.000449
CNAG_04101	1814.781	-0.89599	0.074144	-12.0845	1.27E-33	2.87E-32
CNAG_04102	355.3392	-0.6172	0.125693	-4.91036	9.09E-07	4.68E-06
CNAG_04103	423.4972	2.334165	0.169438	13.77595	3.56E-43	1.07E-41
CNAG_04104	1075.31	0.357138	0.095059	3.757025	0.000172	0.000601
CNAG_04108	1133.192	-0.30219	0.101667	-2.97233	0.002955	0.007812
CNAG_04110	441.0806	-0.22997	0.117114	-1.96368	0.049567	0.093001
CNAG_04111	2338.893	-0.20135	0.069747	-2.88688	0.003891	0.009989
CNAG_12812	95.75616	0.890112	0.293856	3.029072	0.002453	0.006615
CNAG_04112	439.5254	-0.56166	0.142599	-3.93877	8.19E-05	0.000306
CNAG_04113	3742.86	-0.20319	0.07831	-2.5947	0.009467	0.022042
CNAG_04115	1297.729	0.355449	0.087435	4.065299	4.80E-05	0.000186
CNAG_04122	224.1128	-0.77591	0.163578	-4.7434	2.10E-06	1.03E-05
CNAG_12817	25.84617	-1.15188	0.41767	-2.75788	0.005818	0.014278
CNAG_04131	3118.811	0.139247	0.068288	2.039123	0.041438	0.079774

CNAG_04132	2602.374	-0.41777	0.070947	-5.88841	3.90E-09	2.69E-08
CNAG_07757	1401.705	0.266031	0.083418	3.189122	0.001427	0.00405
CNAG_04137	250.9376	-0.55847	0.143809	-3.88343	0.000103	0.000376
CNAG_04139	123.1084	-0.65067	0.215143	-3.02436	0.002492	0.006704
CNAG_12820	65.65765	-0.92004	0.269164	-3.41814	0.00063	0.001948
CNAG_04141	2908.962	-0.96125	0.07028	-13.6775	1.38E-42	4.05E-41
CNAG_04142	1869.106	0.476033	0.117196	4.061862	4.87E-05	0.000189
CNAG_04143	29.80756	1.354155	0.391983	3.454625	0.000551	0.001725
CNAG_12821	524.5282	0.489239	0.139113	3.516836	0.000437	0.001396
CNAG_07758	505.8195	0.285608	0.10725	2.663019	0.007744	0.018416
CNAG_04148	1833.809	0.193378	0.07458	2.592879	0.009518	0.022109
CNAG_04150	1690.796	-0.52216	0.082487	-6.33021	2.45E-10	1.91E-09
CNAG_04151	2558.041	-0.13804	0.070258	-1.96471	0.049448	0.092843
CNAG_04152	837.9981	-0.37823	0.087317	-4.33176	1.48E-05	6.29E-05
CNAG_04155	2619.717	-0.37984	0.090236	-4.20938	2.56E-05	0.000104
CNAG_04156	2308.718	-0.38869	0.075435	-5.1526	2.57E-07	1.43E-06
CNAG_04157	905.4701	-0.37027	0.110257	-3.35824	0.000784	0.002369
CNAG_04158	1418.989	-0.20521	0.081041	-2.53211	0.011338	0.0259
CNAG_04159	1366.832	-0.2172	0.078813	-2.7559	0.005853	0.014351
CNAG_04161	1005.554	-0.28167	0.082892	-3.39803	0.000679	0.002083
CNAG_04163	1998.03	-0.90442	0.251219	-3.60011	0.000318	0.00105
CNAG_04164	1076.06	-0.18332	0.08285	-2.21262	0.026924	0.055053
CNAG_04165	437.3919	-0.30769	0.138178	-2.22675	0.025964	0.053292
CNAG_04166	1836.174	-0.2316	0.082637	-2.80258	0.00507	0.012659
CNAG_04167	170.4251	0.671214	0.172392	3.893541	9.88E-05	0.000363
CNAG_04168	6472.061	-1.27106	0.084299	-15.078	2.26E-51	7.82E-50
CNAG_04169	545.6578	0.639088	0.109492	5.836826	5.32E-09	3.62E-08
CNAG_04170	682.6	0.306357	0.106157	2.885892	0.003903	0.010017
CNAG_04171	3133.066	0.308297	0.079344	3.885569	0.000102	0.000373
CNAG_04172	765.9888	-0.31064	0.117144	-2.65176	0.008007	0.018956
CNAG_04175	2807.24	-0.2286	0.09338	-2.44811	0.014361	0.031788
CNAG_04177	928.5381	-0.19899	0.088932	-2.23757	0.025249	0.051942
CNAG_04178	413.0029	-0.76698	0.144462	-5.30921	1.10E-07	6.42E-07
CNAG_04180	2559.599	-0.19653	0.069462	-2.82936	0.004664	0.011752
CNAG_12827	1144.077	-0.29415	0.11225	-2.6205	0.00878	0.020614
CNAG_04184	908.7566	0.816952	0.088053	9.277965	1.73E-20	2.51E-19
CNAG_07997	114.1534	0.719581	0.24397	2.949465	0.003183	0.008352
CNAG_04185	843.0872	0.928566	0.155733	5.96256	2.48E-09	1.74E-08
CNAG_04186	939.4431	1.013128	0.109825	9.224922	2.84E-20	4.10E-19
CNAG_04187	1574.288	0.530502	0.075934	6.986339	2.82E-12	2.59E-11
CNAG_04189	20311.82	1.286983	0.057847	22.24813	1.18E-109	9.75E-108
CNAG_04190	5780.054	-0.19292	0.064512	-2.99052	0.002785	0.007415
CNAG_04191	1647.322	-0.2981	0.082324	-3.62109	0.000293	0.000973
CNAG_04192	7765.069	-0.17881	0.056783	-3.14902	0.001638	0.004593
CNAG_04193	1150.005	0.235504	0.081515	2.889084	0.003864	0.009925
CNAG_04194	4551.731	-0.16359	0.06466	-2.52995	0.011408	0.026009
CNAG_04195	2100.156	2.038069	0.080449	25.3337	1.36E-141	1.62E-139

CNAG_04196	3956.058	-0.34751	0.07291	-4.76636	1.88E-06	9.27E-06
CNAG_04198	1363.661	0.276281	0.0848	3.258024	0.001122	0.003261
CNAG_04200	886.3866	1.522187	0.10995	13.84437	1.38E-43	4.20E-42
CNAG_04201	177.3294	1.928538	0.267147	7.219014	5.24E-13	5.13E-12
CNAG_04202	1969.384	1.568638	0.087146	18.00009	1.95E-72	9.76E-71
CNAG_04204	1321.856	-0.30337	0.09855	-3.0783	0.002082	0.005703
CNAG_04205	1773.113	-0.30468	0.089095	-3.41969	0.000627	0.001943
CNAG_04206	635.7365	1.274941	0.108691	11.72991	8.96E-32	1.91E-30
CNAG_12830	67.41798	0.935317	0.296337	3.156258	0.001598	0.004493
CNAG_04208	1005.587	-0.41827	0.089619	-4.66718	3.05E-06	1.46E-05
CNAG_04209	4740.993	0.850339	0.098377	8.643697	5.44E-18	6.94E-17
CNAG_04210	4982.188	2.554371	0.080569	31.7043	1.36E-220	3.24E-218
CNAG_04215	13216.55	0.298071	0.059167	5.037793	4.71E-07	2.53E-06
CNAG_12832	129.7409	0.914195	0.234492	3.898626	9.67E-05	0.000356
CNAG_04217	2120.597	1.115081	0.314747	3.542788	0.000396	0.001276
CNAG_04218	154.676	-0.68627	0.19001	-3.61177	0.000304	0.001006
CNAG_04219	4750.55	0.765423	0.068802	11.12507	9.47E-29	1.86E-27
CNAG_04221	1914.536	0.429603	0.069939	6.142558	8.12E-10	5.99E-09
CNAG_04222	5056.236	0.142885	0.072301	1.976242	0.048127	0.090787
CNAG_04224	2299.048	-0.66885	0.082957	-8.06263	7.47E-16	8.63E-15
CNAG_04225	249.7219	0.5438	0.203085	2.6777	0.007413	0.017706
CNAG_04227	1424.233	0.197497	0.080183	2.46307	0.013775	0.030686
CNAG_04228	244.1602	0.713495	0.174322	4.092977	4.26E-05	0.000167
CNAG_04231	315.0142	-0.37308	0.133831	-2.78771	0.005308	0.013194
CNAG_04234	799.8823	-0.19415	0.096848	-2.00473	0.044992	0.085763
CNAG_04236	3819.328	-0.20452	0.067572	-3.02676	0.002472	0.006657
CNAG_04237	706.9689	-0.48962	0.119058	-4.1125	3.91E-05	0.000155
CNAG_04239	356.1775	-0.46627	0.153393	-3.03967	0.002368	0.00641
CNAG_04240	290.4571	-0.34435	0.14929	-2.30661	0.021077	0.044266
CNAG_04241	2502.326	2.683379	0.077486	34.63029	8.85E-263	3.42E-260
CNAG_04242	1468.773	1.801944	0.087771	20.52996	1.16E-93	7.68E-92
CNAG_12835	20.56384	1.199186	0.486872	2.463043	0.013776	0.030686
CNAG_04243	2023.651	-0.2572	0.08694	-2.95837	0.003093	0.00814
CNAG_04244	1058.983	0.526694	0.099354	5.301205	1.15E-07	6.69E-07
CNAG_04245	8885.769	0.292654	0.061401	4.766232	1.88E-06	9.27E-06
CNAG_04246	159.6716	0.587798	0.190548	3.08478	0.002037	0.005597
CNAG_04248	527.4485	-0.35116	0.108235	-3.24446	0.001177	0.003404
CNAG_07763	438.9119	-0.61091	0.113942	-5.36158	8.25E-08	4.89E-07
CNAG_04253	548.888	-0.51672	0.123744	-4.17572	2.97E-05	0.000119
CNAG_04256	287.6402	0.424661	0.149038	2.849354	0.004381	0.01112
CNAG_04257	6691.423	0.908518	0.059833	15.1843	4.49E-52	1.59E-50
CNAG_04259	6759.154	-0.32409	0.084814	-3.82124	0.000133	0.000473
CNAG_04262	355.3273	0.45497	0.130238	3.493377	0.000477	0.00151
CNAG_04263	520.9823	-0.38341	0.134759	-2.84512	0.004439	0.011245
CNAG_04264	853.2895	-0.17398	0.087797	-1.98157	0.047527	0.089837
CNAG_04265	2683.983	-0.34668	0.085106	-4.07354	4.63E-05	0.00018
CNAG_04267	1445.415	0.770674	0.077913	9.891466	4.53E-23	7.44E-22

CNAG_12838	24.69194	2.22658	0.457932	4.862246	1.16E-06	5.89E-06
CNAG_04269	1122.592	-0.21827	0.100812	-2.16513	0.030378	0.061056
CNAG_04271	978.387	0.703585	0.092104	7.639033	2.19E-14	2.34E-13
CNAG_04272	2688.908	0.599222	0.071697	8.357683	6.40E-17	7.79E-16
CNAG_04273	1974.393	-0.43477	0.06839	-6.35722	2.05E-10	1.61E-09
CNAG_04275	800.786	-0.35143	0.142299	-2.46962	0.013526	0.030186
CNAG_04276	2514.372	0.196657	0.068809	2.858027	0.004263	0.010858
CNAG_04277	1235.368	0.661379	0.090251	7.328243	2.33E-13	2.33E-12
CNAG_04278	1474.891	0.377131	0.076313	4.941917	7.74E-07	4.03E-06
CNAG_04281	2450.358	-0.1604	0.075735	-2.11786	0.034187	0.067445
CNAG_04284	4343.728	-0.66153	0.083769	-7.89706	2.86E-15	3.19E-14
CNAG_04285	709.8897	-0.37669	0.126298	-2.98258	0.002858	0.007587
CNAG_04286	2512.025	-0.281	0.08016	-3.5055	0.000456	0.00145
CNAG_04288	710.0328	-0.32293	0.112595	-2.86811	0.004129	0.010545
CNAG_04289	4082.679	-0.29491	0.083976	-3.51181	0.000445	0.00142
CNAG_04294	347.7864	0.565691	0.122393	4.621928	3.80E-06	1.80E-05
CNAG_04297	752.9171	0.595135	0.10781	5.520222	3.39E-08	2.10E-07
CNAG_04299	634.3847	-0.34879	0.103248	-3.37822	0.00073	0.002228
CNAG_04302	158.7116	-0.47456	0.194969	-2.43402	0.014932	0.032865
CNAG_04303	4372.933	-0.15491	0.067711	-2.28788	0.022144	0.046365
CNAG_04304	7655.161	-0.18002	0.062417	-2.88419	0.003924	0.010062
CNAG_04305	525.59	-0.2676	0.116902	-2.28911	0.022073	0.046239
CNAG_04307	318.0846	3.635913	0.169571	21.44178	5.45E-102	4.14E-100
CNAG_04309	353.923	0.616953	0.13831	4.460654	8.17E-06	3.66E-05
CNAG_04310	1030.921	-0.17349	0.085847	-2.02086	0.043294	0.082896
CNAG_04312	2523.024	0.484981	0.074456	6.513643	7.33E-11	5.99E-10
CNAG_04313	457.2462	0.908217	0.125339	7.246064	4.29E-13	4.26E-12
CNAG_04315	2656.986	0.481986	0.086031	5.60244	2.11E-08	1.33E-07
CNAG_04319	256.0929	-0.35296	0.142141	-2.48314	0.013023	0.029241
CNAG_04320	2657.543	0.570161	0.063414	8.99108	2.45E-19	3.36E-18
CNAG_04321	1986.855	0.384393	0.083916	4.580682	4.63E-06	2.16E-05
CNAG_04322	1234.27	0.736481	0.325359	2.263593	0.023599	0.048995
CNAG_04325	7426.228	3.795506	0.11419	33.23848	3.00E-242	8.70E-240
CNAG_04327	7223.642	0.172847	0.060641	2.850317	0.004368	0.01109
CNAG_04330	52.33667	1.020008	0.331531	3.076661	0.002093	0.005733
CNAG_12843	44.08418	0.829396	0.344001	2.411028	0.015908	0.034701
CNAG_04333	138.4455	0.935351	0.249192	3.753541	0.000174	0.000608
CNAG_04334	2479.42	-0.21427	0.090674	-2.36312	0.018122	0.038915
CNAG_04335	1740.468	-0.23647	0.076077	-3.10827	0.001882	0.005222
CNAG_04336	343.5665	-0.41053	0.149339	-2.74899	0.005978	0.014618
CNAG_04338	466.1821	-0.48068	0.123598	-3.88905	0.000101	0.000368
CNAG_04340	1943.17	-0.23142	0.075331	-3.07212	0.002125	0.005807
CNAG_04341	1807.322	-0.18147	0.07671	-2.36564	0.017999	0.038693
CNAG_12845	23.60756	3.38583	0.563035	6.013531	1.82E-09	1.29E-08
CNAG_04344	423.1715	0.685727	0.147522	4.648308	3.35E-06	1.59E-05
CNAG_04345	759.7339	0.297079	0.14496	2.049383	0.040425	0.07822
CNAG_04347	5348.149	-0.19321	0.060113	-3.21412	0.001308	0.003746

CNAG_04348	3142.573	0.502338	0.07204	6.973059	3.10E-12	2.83E-11
CNAG_04349	1552.564	0.388105	0.083783	4.632256	3.62E-06	1.72E-05
CNAG_12847	9.522774	2.036823	0.810752	2.512265	0.011996	0.027167
CNAG_04350	1212.217	1.267266	0.09034	14.02768	1.06E-44	3.27E-43
CNAG_04351	421.2563	0.389005	0.120463	3.229235	0.001241	0.003565
CNAG_04358	3070.463	-0.27063	0.074682	-3.62376	0.00029	0.000964
CNAG_04359	1237.44	-0.31263	0.094671	-3.30223	0.000959	0.002839
CNAG_04360	1641.37	-0.29446	0.076623	-3.843	0.000122	0.000438
CNAG_04361	4322.155	-0.76218	0.087825	-8.67841	4.01E-18	5.17E-17
CNAG_04362	3615.74	-0.25901	0.063245	-4.09529	4.22E-05	0.000165
CNAG_04364	4436.238	0.303629	0.077221	3.931974	8.43E-05	0.000314
CNAG_04365	7113.379	0.13798	0.062991	2.19048	0.028489	0.057761
CNAG_04368	1290.765	-0.31482	0.086776	-3.62798	0.000286	0.00095
CNAG_04369	289.041	-0.54094	0.134241	-4.02962	5.59E-05	0.000215
CNAG_04372	1882.496	-0.18327	0.075292	-2.43419	0.014925	0.032858
CNAG_04373	8005.141	0.407245	0.060918	6.685128	2.31E-11	1.96E-10
CNAG_04375	2652.021	-0.14816	0.068371	-2.16707	0.03023	0.060789
CNAG_04377	2005.513	-0.22802	0.096141	-2.37169	0.017707	0.038115
CNAG_04380	1348.627	0.306475	0.084441	3.629458	0.000284	0.000946
CNAG_04382	1545.029	0.172736	0.077075	2.241136	0.025017	0.05157
CNAG_04383	1399.147	-0.21514	0.086896	-2.47585	0.013292	0.029731
CNAG_04387	719.5862	-0.73444	0.098388	-7.46467	8.35E-14	8.62E-13
CNAG_07766	697.1233	0.380223	0.099237	3.831462	0.000127	0.000456
CNAG_07765	135.9897	-0.94572	0.302321	-3.12819	0.001759	0.004904
CNAG_04393	2277.012	0.230832	0.07221	3.19666	0.00139	0.003958
CNAG_04396	301.28	-0.33203	0.13415	-2.47506	0.013321	0.029787
CNAG_04397	463.9313	-0.30507	0.111781	-2.7292	0.006349	0.015418
CNAG_04398	1141.478	-0.42737	0.087765	-4.86943	1.12E-06	5.70E-06
CNAG_04399	963.0344	-0.30041	0.095796	-3.13595	0.001713	0.004781
CNAG_04401	2261.805	-0.57424	0.076754	-7.48157	7.34E-14	7.62E-13
CNAG_04402	1836.793	-0.25314	0.088789	-2.851	0.004358	0.01107
CNAG_04405	936.4631	-0.33805	0.096536	-3.5018	0.000462	0.001468
CNAG_04409	345.8262	-0.62907	0.15956	-3.94255	8.06E-05	0.000301
CNAG_04411	565.8979	0.245613	0.117819	2.084665	0.0371	0.072539
CNAG_04415	253.6549	-0.35361	0.146616	-2.41183	0.015873	0.034643
CNAG_04416	135.1849	2.365735	0.217715	10.86618	1.67E-27	3.16E-26
CNAG_04417	976.4353	1.368636	0.134151	10.20222	1.94E-24	3.31E-23
CNAG_12853	219.0377	-1.03217	0.159241	-6.48181	9.06E-11	7.36E-10
CNAG_07769	2984.121	0.372769	0.065489	5.692106	1.25E-08	8.14E-08
CNAG_04432	700.5972	-0.36376	0.092363	-3.9384	8.20E-05	0.000306
CNAG_04434	916.0327	-0.59328	0.115875	-5.12001	3.06E-07	1.68E-06
CNAG_07770	1899.458	0.279581	0.078995	3.539226	0.000401	0.001292
CNAG_04436	925.7561	0.210584	0.086615	2.431268	0.015046	0.033062
CNAG_04437	635.2994	-0.83121	0.099957	-8.31566	9.12E-17	1.10E-15
CNAG_04438	823.8402	-0.20432	0.103478	-1.97457	0.048316	0.091012
CNAG_04439	3606.757	-0.20434	0.062799	-3.25383	0.001139	0.003306
CNAG_04440	603.2056	-0.50654	0.122157	-4.14666	3.37E-05	0.000135

CNAG_04443	1023.959	-0.71821	0.086635	-8.29004	1.13E-16	1.36E-15
CNAG_04444	4837.277	-0.35001	0.065351	-5.3558	8.52E-08	5.05E-07
CNAG_04445	44810.06	-0.15916	0.076132	-2.09061	0.036563	0.071593
CNAG_04447	849.4895	0.302276	0.090868	3.326536	0.000879	0.002624
CNAG_04449	1296.243	0.319054	0.094017	3.393564	0.00069	0.002113
CNAG_04450	3470.287	0.164616	0.063822	2.579297	0.0099	0.022893
CNAG_04455	364.1478	0.384048	0.123897	3.099734	0.001937	0.005359
CNAG_04457	28.09225	-0.87207	0.40394	-2.1589	0.030858	0.061852
CNAG_04458	772.2769	-0.40102	0.090961	-4.40869	1.04E-05	4.56E-05
CNAG_04459	69.4329	2.163756	0.421816	5.129619	2.90E-07	1.60E-06
CNAG_12856	166.7236	0.774609	0.23622	3.279177	0.001041	0.003049
CNAG_04460	2608.456	-0.46032	0.068654	-6.70496	2.01E-11	1.72E-10
CNAG_12860	16.55816	1.39202	0.537201	2.591246	0.009563	0.022208
CNAG_04465	3035.888	-0.27465	0.070046	-3.92096	8.82E-05	0.000327
CNAG_04466	492.6355	0.253092	0.112131	2.257098	0.024002	0.049717
CNAG_04467	768.1813	0.34874	0.101605	3.432318	0.000598	0.001861
CNAG_12863	145.9734	4.916215	0.310424	15.83709	1.73E-56	6.71E-55
CNAG_04469	646.1785	5.005497	0.161303	31.03171	2.01E-211	4.55E-209
CNAG_04470	1356.706	3.410557	0.100188	34.04156	5.41E-254	1.83E-251
CNAG_04471	749.9792	1.295941	0.114192	11.34884	7.52E-30	1.54E-28
CNAG_04472	226.0888	2.622711	0.198949	13.18285	1.10E-39	2.99E-38
CNAG_12864	21.59614	2.818814	0.583922	4.827382	1.38E-06	6.94E-06
CNAG_04474	251.3096	0.954612	0.162332	5.880632	4.09E-09	2.81E-08
CNAG_04475	2389.194	0.559705	0.07846	7.133602	9.78E-13	9.33E-12
CNAG_04476	169.9747	0.972572	0.249037	3.905331	9.41E-05	0.000347
CNAG_07771	1827.681	-0.33352	0.072652	-4.59058	4.42E-06	2.07E-05
CNAG_04478	2931.099	0.265981	0.084748	3.138491	0.001698	0.00475
CNAG_04479	556.4628	-0.55447	0.129049	-4.29659	1.73E-05	7.28E-05
CNAG_07774	487.5299	0.526371	0.110127	4.779688	1.76E-06	8.72E-06
CNAG_04485	5924.183	-0.21	0.079791	-2.6319	0.008491	0.019987
CNAG_04488	2465.59	-0.27511	0.076351	-3.60322	0.000314	0.001038
CNAG_07775	564.2893	-0.60429	0.183173	-3.299	0.00097	0.002866
CNAG_07777	271.6706	-0.35886	0.138336	-2.59412	0.009483	0.022056
CNAG_04494	1650.21	-0.34159	0.095717	-3.56876	0.000359	0.00117
CNAG_04495	184.9344	-0.49302	0.167155	-2.94946	0.003183	0.008352
CNAG_04496	1046.504	-0.42923	0.099194	-4.32716	1.51E-05	6.41E-05
CNAG_04497	585.4135	-0.29	0.106664	-2.71878	0.006552	0.015855
CNAG_04501	3623.471	-0.2779	0.068553	-4.05384	5.04E-05	0.000195
CNAG_04503	698.4389	-0.33767	0.096573	-3.49651	0.000471	0.001495
CNAG_04504	2738.914	1.113768	0.07627	14.60302	2.69E-48	8.74E-47
CNAG_12866	76.81096	0.908053	0.254476	3.56832	0.000359	0.001171
CNAG_04505	2358.23	-0.18973	0.082938	-2.28762	0.022159	0.046374
CNAG_12867	21.30836	1.267126	0.489213	2.590134	0.009594	0.022261
CNAG_04510	2299.193	-0.21947	0.073764	-2.97535	0.002927	0.007748
CNAG_04511	307.4679	-0.47423	0.141562	-3.34997	0.000808	0.002433
CNAG_04512	461.5559	-0.28524	0.113324	-2.51704	0.011835	0.026854
CNAG_04514	1847.871	0.209991	0.071114	2.952874	0.003148	0.008271

CNAG_04515	2186.919	-1.5057	0.098987	-15.211	2.99E-52	1.07E-50
CNAG_12869	139.1626	-1.64183	0.230812	-7.11327	1.13E-12	1.08E-11
CNAG_04518	578.4792	-0.27859	0.114573	-2.43155	0.015034	0.033045
CNAG_04519	168.9949	0.553988	0.17945	3.087149	0.002021	0.005559
CNAG_12871	381.9505	1.962366	0.144113	13.61682	3.18E-42	9.07E-41
CNAG_04521	2224.449	5.652485	0.117147	48.25123	0	0
CNAG_04522	1243.057	0.81986	0.094615	8.665251	4.51E-18	5.79E-17
CNAG_04523	30.45696	1.208954	0.391565	3.087492	0.002019	0.005554
CNAG_04524	1694.854	1.162084	0.090082	12.9003	4.48E-38	1.15E-36
CNAG_04527	665.4656	-0.26372	0.100965	-2.61195	0.009003	0.021117
CNAG_04528	291.0624	-0.39786	0.154461	-2.57582	0.01	0.023091
CNAG_04529	312.7125	-0.50169	0.146759	-3.4185	0.00063	0.001948
CNAG_04530	2065.873	-0.27333	0.080187	-3.40862	0.000653	0.002008
CNAG_04531	1226.633	-0.6371	0.091302	-6.97801	2.99E-12	2.74E-11
CNAG_07778	8817.827	-0.29339	0.068381	-4.29052	1.78E-05	7.47E-05
CNAG_07779	71.30721	3.894425	0.356776	10.9156	9.71E-28	1.86E-26
CNAG_04535	102.7336	0.861518	0.21187	4.066249	4.78E-05	0.000186
CNAG_04536	75.89429	1.255344	0.270819	4.635363	3.56E-06	1.69E-05
CNAG_04537	490.1641	-0.35278	0.145854	-2.41869	0.015576	0.034088
CNAG_04539	1137.841	0.590134	0.103625	5.694913	1.23E-08	8.02E-08
CNAG_04541	1504.555	-0.17638	0.084986	-2.0754	0.03795	0.074005
CNAG_04544	470.4639	0.255747	0.121019	2.113281	0.034577	0.068114
CNAG_04545	702.5206	-0.25307	0.092795	-2.72715	0.006388	0.015504
CNAG_04546	57.21206	1.050033	0.290227	3.61797	0.000297	0.000984
CNAG_12873	118.0525	0.724858	0.215302	3.3667	0.000761	0.002307
CNAG_07780	647.6429	0.340508	0.099328	3.428109	0.000608	0.001887
CNAG_07781	1218.093	0.832405	0.09042	9.20596	3.39E-20	4.84E-19
CNAG_07020	1709.533	0.195951	0.077825	2.517833	0.011808	0.026801
CNAG_07782	987.6819	1.122647	0.092275	12.16637	4.69E-34	1.07E-32
CNAG_06997	565.0639	0.461283	0.125061	3.68847	0.000226	0.000767
CNAG_06999	341.4196	-0.76415	0.16994	-4.49659	6.91E-06	3.14E-05
CNAG_12876	573.6393	-0.24973	0.107323	-2.32688	0.019972	0.042351
CNAG_07784	488.4333	4.482754	0.16974	26.40947	1.07E-153	1.45E-151
CNAG_06953	79.0404	-1.04772	0.246726	-4.24649	2.17E-05	8.93E-05
CNAG_04927	117.1542	0.844996	0.205823	4.105458	4.04E-05	0.000159
CNAG_04926	179.5091	0.725712	0.164114	4.421996	9.78E-06	4.32E-05
CNAG_04925	1540.547	-0.37198	0.072974	-5.09748	3.44E-07	1.88E-06
CNAG_04921	36.32467	0.840088	0.351375	2.390858	0.016809	0.036435
CNAG_04920	1979.958	1.694896	0.123208	13.75633	4.67E-43	1.40E-41
CNAG_04919	475.3407	0.926412	0.118542	7.815036	5.49E-15	6.03E-14
CNAG_07838	827.7808	-0.27632	0.088096	-3.13656	0.001709	0.004773
CNAG_07839	50250.59	-0.25772	0.072266	-3.56621	0.000362	0.00118
CNAG_04914	4730.539	-0.60735	0.068391	-8.88052	6.65E-19	8.90E-18
CNAG_04913	1635.881	-0.3026	0.082843	-3.65265	0.00026	0.00087
CNAG_04911	576.0887	0.807788	0.108276	7.460474	8.62E-14	8.87E-13
CNAG_04909	1642.072	2.263187	0.077803	29.08867	4.99E-186	9.02E-184
CNAG_12880	36.28588	0.983441	0.381567	2.577374	0.009955	0.023014

CNAG_04908	926.5003	0.530334	0.124481	4.260377	2.04E-05	8.45E-05
CNAG_04906	2993.129	0.184861	0.064123	2.882917	0.00394	0.010095
CNAG_04905	87.22985	0.908046	0.234109	3.878735	0.000105	0.000383
CNAG_07842	158.839	0.875406	0.174025	5.030332	4.90E-07	2.63E-06
CNAG_04903	1158.993	1.198809	0.341246	3.513037	0.000443	0.001415
CNAG_04901	560.8244	1.491422	0.108469	13.74973	5.11E-43	1.53E-41
CNAG_04899	1575.295	-0.23343	0.081462	-2.86553	0.004163	0.010624
CNAG_04898	185.2022	0.482628	0.169548	2.846565	0.004419	0.011197
CNAG_04894	347.0558	0.749208	0.19514	3.839328	0.000123	0.000444
CNAG_04892	193.0196	-0.67935	0.170803	-3.97737	6.97E-05	0.000263
CNAG_04891	94.38786	1.747955	0.243005	7.193089	6.33E-13	6.14E-12
CNAG_04890	110.9697	3.555587	0.287057	12.38634	3.10E-35	7.34E-34
CNAG_04889	1020.384	1.367997	0.109427	12.50149	7.33E-36	1.77E-34
CNAG_04887	239.4791	1.828331	0.163019	11.21548	3.42E-29	6.87E-28
CNAG_04886	477.991	0.254259	0.117539	2.163181	0.030527	0.061281
CNAG_12883	56.57471	1.402576	0.36102	3.885031	0.000102	0.000374
CNAG_07844	174.9827	4.314451	0.250669	17.21176	2.17E-66	1.03E-64
CNAG_04883	37856.6	-0.26537	0.078163	-3.39515	0.000686	0.002104
CNAG_04882	1238.459	-0.37905	0.079014	-4.79722	1.61E-06	8.03E-06
CNAG_04880	472.3937	0.550636	0.123329	4.464761	8.02E-06	3.60E-05
CNAG_04879	3675.307	-0.85572	0.078893	-10.8466	2.07E-27	3.90E-26
CNAG_12885	70.86427	0.767714	0.2606	2.945944	0.00322	0.008432
CNAG_04877	34.47079	1.243258	0.455897	2.727056	0.00639	0.015504
CNAG_04876	1933.753	0.21863	0.070482	3.101932	0.001923	0.005324
CNAG_04874	549.8908	0.904636	0.126511	7.150676	8.64E-13	8.29E-12
CNAG_04873	289.5207	0.868168	0.134454	6.456983	1.07E-10	8.60E-10
CNAG_04872	2675.406	0.51763	0.06847	7.559923	4.03E-14	4.24E-13
CNAG_04871	438.5878	0.280833	0.112089	2.505443	0.01223	0.02762
CNAG_12886	171.8027	0.922662	0.180878	5.101032	3.38E-07	1.85E-06
CNAG_04870	56.17476	0.587118	0.292907	2.004455	0.045021	0.085799
CNAG_04869	3483.26	1.294807	0.079199	16.3487	4.44E-60	1.88E-58
CNAG_04868	1478.635	0.493533	0.104201	4.736356	2.18E-06	1.07E-05
CNAG_04866	901.4675	0.386639	0.103663	3.729781	0.000192	0.000663
CNAG_04865	101.3745	1.149945	0.323755	3.551893	0.000382	0.001239
CNAG_12888	46.92136	2.254059	0.340983	6.61047	3.83E-11	3.21E-10
CNAG_04864	2188.266	-1.14947	0.087939	-13.0712	4.81E-39	1.28E-37
CNAG_04863	1107.163	1.625056	0.096701	16.80489	2.25E-63	1.01E-61
CNAG_12889	103.3623	4.739899	0.368383	12.86677	6.92E-38	1.77E-36
CNAG_04862	10918.01	3.190223	0.07032	45.36703	0	0
CNAG_12890	48.21804	2.104236	0.362379	5.806731	6.37E-09	4.30E-08
CNAG_04861	2039.636	0.722291	0.084073	8.59127	8.60E-18	1.09E-16
CNAG_04860	2096.062	0.229304	0.085705	2.675499	0.007462	0.017817
CNAG_04859	885.9186	-0.68803	0.105985	-6.4918	8.48E-11	6.91E-10
CNAG_04857	3919.748	0.38279	0.09455	4.048554	5.15E-05	0.000199
CNAG_04853	579.8694	-0.51732	0.106259	-4.86844	1.12E-06	5.72E-06
CNAG_04851	13322.32	0.222387	0.055511	4.006153	6.17E-05	0.000235
CNAG_12892	141.7236	-1.17008	0.21919	-5.3382	9.39E-08	5.53E-07

CNAG_04849	403.3195	-0.5012	0.1247	-4.01924	5.84E-05	0.000223
CNAG_04848	2954.941	-0.45986	0.065599	-7.01011	2.38E-12	2.21E-11
CNAG_04847	980.2396	0.18501	0.084722	2.183715	0.028983	0.058601
CNAG_04846	2519.608	1.217549	0.105994	11.48701	1.53E-30	3.20E-29
CNAG_04845	1128.78	-0.42084	0.08674	-4.85177	1.22E-06	6.20E-06
CNAG_04842	1351.386	0.187446	0.079358	2.362022	0.018176	0.038989
CNAG_04840	5801.675	-0.42221	0.062496	-6.75579	1.42E-11	1.23E-10
CNAG_04838	2777.035	-0.17883	0.069595	-2.56956	0.010183	0.023466
CNAG_04837	133.324	-0.66905	0.209491	-3.1937	0.001405	0.003993
CNAG_04836	676.624	0.341658	0.099557	3.431787	0.0006	0.001864
CNAG_04833	1010.677	-0.31285	0.134534	-2.32546	0.020048	0.042467
CNAG_04829	416.6248	-0.39454	0.138743	-2.84365	0.00446	0.011282
CNAG_12895	220.458	0.514793	0.154651	3.328736	0.000872	0.002607
CNAG_04828	7826.192	-0.54314	0.064745	-8.38893	4.91E-17	6.02E-16
CNAG_04827	257.8583	0.479145	0.165913	2.887936	0.003878	0.009958
CNAG_04824	554.745	-0.56047	0.103584	-5.41084	6.27E-08	3.78E-07
CNAG_04823	1883.063	-0.55183	0.080025	-6.89572	5.36E-12	4.82E-11
CNAG_04822	1543.099	0.197977	0.076193	2.598379	0.009367	0.021845
CNAG_04820	1790.474	-0.19831	0.083355	-2.37906	0.017357	0.037441
CNAG_04819	1233.434	1.610855	0.091272	17.64904	1.04E-69	5.07E-68
CNAG_04818	565.761	3.616379	0.137294	26.34036	6.62E-153	8.82E-151
CNAG_07845	118.7506	-0.45791	0.212551	-2.15437	0.031212	0.0625
CNAG_04813	1250.813	-0.352	0.089899	-3.91547	9.02E-05	0.000334
CNAG_04810	432.3292	-0.3073	0.119638	-2.56859	0.010211	0.023525
CNAG_04808	3944.613	-0.13168	0.064303	-2.04782	0.040578	0.078457
CNAG_04807	1488.21	-0.97434	0.115804	-8.41373	3.97E-17	4.90E-16
CNAG_04806	5165.772	-0.20037	0.064142	-3.12393	0.001785	0.004967
CNAG_04805	1424.632	-0.3564	0.078937	-4.51495	6.33E-06	2.90E-05
CNAG_04804	1709.57	-0.95862	0.077038	-12.4435	1.52E-35	3.62E-34
CNAG_04802	1826.075	-0.19244	0.075236	-2.55786	0.010532	0.024209
CNAG_04800	6425.96	-0.36325	0.062808	-5.7835	7.32E-09	4.90E-08
CNAG_04799	46517.5	-0.21547	0.070237	-3.06779	0.002156	0.005882
CNAG_04798	2701.914	-0.39408	0.084348	-4.6721	2.98E-06	1.43E-05
CNAG_04796	4036.942	0.615802	0.066201	9.30201	1.38E-20	2.01E-19
CNAG_04795	671.7788	0.493396	0.122458	4.029091	5.60E-05	0.000215
CNAG_04792	2030.593	0.384647	0.072876	5.278088	1.31E-07	7.54E-07
CNAG_04791	1548.782	-0.20133	0.075768	-2.65721	0.007879	0.018682
CNAG_04790	1045.995	-0.25323	0.093563	-2.70655	0.006799	0.016363
CNAG_04788	2388.88	-0.37976	0.084324	-4.50355	6.68E-06	3.05E-05
CNAG_04787	199.0635	-0.54652	0.175034	-3.12235	0.001794	0.004992
CNAG_04785	674.9248	0.437202	0.113155	3.863754	0.000112	0.000405
CNAG_04784	103.3832	2.764237	0.279968	9.873415	5.43E-23	8.81E-22
CNAG_04783	1851.051	-0.18489	0.076791	-2.40772	0.016052	0.03497
CNAG_04782	1775.389	-0.3079	0.072727	-4.23367	2.30E-05	9.39E-05
CNAG_07848	1026.853	-0.34477	0.097403	-3.5396	0.000401	0.00129
CNAG_07851	7246.306	-0.30089	0.067224	-4.47587	7.61E-06	3.44E-05
CNAG_04777	1050.699	0.527891	0.089857	5.874775	4.23E-09	2.90E-08

CNAG_04776	4722.717	-0.30964	0.070092	-4.41753	9.98E-06	4.40E-05
CNAG_04775	492.5194	-0.37442	0.111043	-3.37189	0.000747	0.002273
CNAG_04774	2362.801	0.952925	0.073409	12.98104	1.57E-38	4.11E-37
CNAG_12905	56.86448	1.956782	0.338185	5.786134	7.20E-09	4.83E-08
CNAG_04773	181.3694	2.04332	0.211027	9.682763	3.57E-22	5.56E-21
CNAG_12906	255.9963	2.766887	0.182737	15.14139	8.64E-52	3.01E-50
CNAG_04772	2566.76	-0.16606	0.076274	-2.17715	0.02947	0.059451
CNAG_04770	2991.439	0.189052	0.069835	2.707131	0.006787	0.016349
CNAG_04768	1316.009	0.448293	0.085494	5.243567	1.58E-07	9.00E-07
CNAG_04767	947.1594	-0.39965	0.095936	-4.16587	3.10E-05	0.000124
CNAG_04765	1257.22	-0.49021	0.093275	-5.25557	1.48E-07	8.47E-07
CNAG_04764	576.8756	-0.30326	0.10078	-3.00916	0.00262	0.007011
CNAG_04763	2786.684	0.172175	0.080982	2.12609	0.033496	0.066371
CNAG_04761	601.7613	-0.2218	0.109908	-2.018	0.043591	0.083405
CNAG_04758	913.4176	0.351986	0.108109	3.255847	0.001131	0.003285
CNAG_04756	509.7001	-1.12236	0.125321	-8.95592	3.37E-19	4.58E-18
CNAG_12912	56.70851	0.66464	0.28736	2.312914	0.020727	0.043631
CNAG_04755	2944.124	0.325909	0.082074	3.97092	7.16E-05	0.00027
CNAG_12914	127.9346	1.639486	0.209219	7.836225	4.64E-15	5.14E-14
CNAG_04753	2118.192	2.04778	0.073487	27.86573	6.95E-171	1.11E-168
CNAG_04752	1476.989	0.521769	0.083246	6.267812	3.66E-10	2.80E-09
CNAG_04751	601.2702	-0.37739	0.098201	-3.84307	0.000122	0.000438
CNAG_04750	135.2371	-0.86471	0.204637	-4.22561	2.38E-05	9.71E-05
CNAG_04749	1987.857	0.456595	0.073248	6.233569	4.56E-10	3.45E-09
CNAG_04747	729.4129	0.807479	0.10856	7.438091	1.02E-13	1.05E-12
CNAG_04746	1307.347	-0.28814	0.080565	-3.57656	0.000348	0.001138
CNAG_04743	4385.723	0.268898	0.061956	4.340135	1.42E-05	6.08E-05
CNAG_04742	879.1484	0.575823	0.089357	6.444086	1.16E-10	9.33E-10
CNAG_04738	3694.606	-0.27595	0.074746	-3.69181	0.000223	0.000758
CNAG_04737	5028.361	0.223069	0.104999	2.12449	0.033629	0.06657
CNAG_04736	596.0919	0.868159	0.117139	7.411354	1.25E-13	1.27E-12
CNAG_04735	3766.564	1.403166	0.071438	19.64172	6.80E-86	4.13E-84
CNAG_04734	1243.365	0.300769	0.079449	3.785704	0.000153	0.000541
CNAG_04733	1604.896	-0.17147	0.074807	-2.29212	0.021899	0.045887
CNAG_04732	1171.847	-0.29053	0.081218	-3.57714	0.000347	0.001136
CNAG_04731	499.8423	-0.21392	0.106502	-2.00858	0.044582	0.08509
CNAG_04730	1646.574	0.428003	0.09067	4.720441	2.35E-06	1.15E-05
CNAG_04729	3308.143	0.292199	0.071632	4.079154	4.52E-05	0.000176
CNAG_04724	1839.187	0.323874	0.071364	4.538348	5.67E-06	2.62E-05
CNAG_12920	39.72841	0.984232	0.342175	2.876403	0.004022	0.010288
CNAG_04719	789.1179	-0.29206	0.095753	-3.05013	0.002287	0.00621
CNAG_04718	238.1775	-0.66368	0.169229	-3.92182	8.79E-05	0.000326
CNAG_04716	4239.217	-0.17326	0.062296	-2.78121	0.005416	0.013437
CNAG_04715	2617.862	0.271512	0.090103	3.013341	0.002584	0.006924
CNAG_04714	891.9219	0.998688	0.092167	10.83565	2.33E-27	4.38E-26
CNAG_04713	1445.907	0.232957	0.079299	2.93771	0.003306	0.008633
CNAG_04712	1525.596	0.608104	0.076223	7.977923	1.49E-15	1.70E-14

CNAG_04711	74.07886	-0.9227	0.316504	-2.9153	0.003553	0.009204
CNAG_04710	2107.418	-0.2382	0.071675	-3.32332	0.00089	0.002648
CNAG_04709	3241.691	-0.39364	0.084217	-4.67409	2.95E-06	1.42E-05
CNAG_04708	484.9134	-0.34262	0.117944	-2.90491	0.003674	0.009491
CNAG_04707	284.0546	1.102592	0.145627	7.571343	3.69E-14	3.89E-13
CNAG_07855	4744.044	0.369005	0.061307	6.018983	1.76E-09	1.25E-08
CNAG_04705	2260.096	0.888332	0.067138	13.23141	5.78E-40	1.58E-38
CNAG_04704	78.19078	2.416737	0.269611	8.963778	3.14E-19	4.28E-18
CNAG_12926	82.24474	1.07396	0.246072	4.36442	1.27E-05	5.48E-05
CNAG_04703	2331.947	0.295742	0.083505	3.541621	0.000398	0.001281
CNAG_04698	4125.193	-0.18765	0.059269	-3.16605	0.001545	0.004355
CNAG_04697	1245.027	-0.47365	0.09464	-5.00477	5.59E-07	2.98E-06
CNAG_04696	727.1423	0.897399	0.112708	7.962132	1.69E-15	1.92E-14
CNAG_04695	95.68835	0.638658	0.275997	2.314003	0.020668	0.043542
CNAG_04694	6165.313	0.294821	0.061887	4.763861	1.90E-06	9.38E-06
CNAG_04691	124.2103	1.756828	0.217666	8.071217	6.96E-16	8.06E-15
CNAG_04690	1235.546	1.470936	0.086866	16.93343	2.55E-64	1.18E-62
CNAG_04689	708.8216	1.183143	0.10926	10.82871	2.52E-27	4.71E-26
CNAG_04688	1068.144	2.166457	0.086247	25.11925	3.06E-139	3.51E-137
CNAG_07856	1860.005	1.006916	0.097749	10.30106	6.97E-25	1.21E-23
CNAG_04684	3683.634	-0.18567	0.070322	-2.64032	0.008283	0.019548
CNAG_04683	1450.513	-0.19345	0.087534	-2.21002	0.027104	0.055352
CNAG_04681	188.8275	0.671706	0.169778	3.956368	7.61E-05	0.000286
CNAG_04677	2061.993	1.723547	0.258405	6.669957	2.56E-11	2.17E-10
CNAG_04673	127.859	-0.46863	0.190543	-2.45946	0.013915	0.03091
CNAG_07999	502.5953	0.312853	0.114473	2.732988	0.006276	0.015264
CNAG_04671	1275.437	-0.29771	0.085126	-3.49723	0.00047	0.001491
CNAG_04669	1308.156	0.313547	0.076278	4.110598	3.95E-05	0.000156
CNAG_04668	620.2378	-0.3511	0.107227	-3.27436	0.001059	0.003095
CNAG_04667	575.358	-0.38618	0.103247	-3.74031	0.000184	0.000638
CNAG_04666	5251.775	0.165907	0.056793	2.921287	0.003486	0.009064
CNAG_04665	452.1273	-0.49102	0.111453	-4.40564	1.05E-05	4.62E-05
CNAG_04662	2019.223	0.158745	0.071836	2.20983	0.027117	0.055365
CNAG_12933	60.77652	-0.94272	0.291339	-3.2358	0.001213	0.003496
CNAG_04658	765.4076	1.058954	0.109217	9.695887	3.14E-22	4.93E-21
CNAG_04657	480.4941	0.375817	0.125919	2.984604	0.002839	0.007547
CNAG_04655	1663.514	0.745254	0.081213	9.176582	4.45E-20	6.33E-19
CNAG_04654	1077.794	-0.29842	0.090904	-3.28284	0.001028	0.003014
CNAG_04652	1233.637	0.213878	0.085556	2.499848	0.012425	0.02799
CNAG_04651	2181.828	-0.21493	0.076971	-2.79239	0.005232	0.013021
CNAG_04650	1466.895	-0.19226	0.079244	-2.42615	0.01526	0.033483
CNAG_04647	1238.291	-0.20488	0.086679	-2.36369	0.018094	0.038876
CNAG_04646	675.4883	-0.2655	0.099692	-2.66317	0.007741	0.018413
CNAG_07857	4651.79	-0.206	0.077479	-2.65875	0.007843	0.018608
CNAG_04642	2109.348	0.375775	0.070751	5.311243	1.09E-07	6.35E-07
CNAG_04641	1476.931	-0.3018	0.086501	-3.48901	0.000485	0.001533
CNAG_04638	367.2114	0.557991	0.163309	3.416789	0.000634	0.001956

CNAG_04637	6622.257	-0.14479	0.064019	-2.26163	0.02372	0.049196
CNAG_12934	31.05135	1.130077	0.411838	2.743984	0.00607	0.01482
CNAG_04635	2683.759	0.749505	0.086596	8.655163	4.92E-18	6.30E-17
CNAG_04634	1049.953	0.782912	0.106735	7.335135	2.21E-13	2.22E-12
CNAG_04633	1257.48	0.463213	0.080044	5.786972	7.17E-09	4.81E-08
CNAG_12938	47.0696	-1.1608	0.326532	-3.55494	0.000378	0.001226
CNAG_04630	1621.816	0.241455	0.100704	2.39766	0.0165	0.035869
CNAG_04628	3350.216	-0.16908	0.062689	-2.69714	0.006994	0.016783
CNAG_04626	269.7551	0.519277	0.191656	2.709413	0.00674	0.016266
CNAG_04623	145.6624	0.898845	0.267224	3.363634	0.000769	0.002331
CNAG_04621	5495.95	-0.50707	0.072762	-6.96889	3.19E-12	2.91E-11
CNAG_04617	905.7667	1.927932	0.102277	18.85005	2.94E-79	1.61E-77
CNAG_04615	591.7843	0.701973	0.102472	6.850383	7.37E-12	6.57E-11
CNAG_04614	250.8909	-0.52417	0.142896	-3.66819	0.000244	0.000825
CNAG_04613	3292.759	-0.14155	0.068772	-2.05822	0.039569	0.076722
CNAG_04612	4015.624	0.418975	0.081133	5.164025	2.42E-07	1.34E-06
CNAG_04609	9337.656	0.124772	0.061932	2.014682	0.043938	0.083971
CNAG_04606	259.4606	1.066474	0.171593	6.215123	5.13E-10	3.86E-09
CNAG_04605	5191.082	-0.63748	0.071011	-8.9772	2.78E-19	3.80E-18
CNAG_04603	561.9257	0.455842	0.124578	3.659086	0.000253	0.000852
CNAG_04601	21636.08	-0.21991	0.060055	-3.66184	0.00025	0.000845
CNAG_04600	1050.518	0.479907	0.084801	5.659225	1.52E-08	9.77E-08
CNAG_04599	1812.189	1.001598	0.077546	12.91615	3.65E-38	9.42E-37
CNAG_04597	550.6653	0.621573	0.113068	5.497339	3.86E-08	2.37E-07
CNAG_12943	14.98676	1.895333	0.58447	3.24282	0.001184	0.003421
CNAG_04596	758.6796	0.279953	0.098258	2.849172	0.004383	0.011123
CNAG_04595	393.0421	0.28232	0.118702	2.378394	0.017388	0.037498
CNAG_04594	544.9614	1.050001	0.109098	9.624419	6.31E-22	9.76E-21
CNAG_04590	196.4843	-0.69359	0.162367	-4.27177	1.94E-05	8.05E-05
CNAG_04589	458.4424	0.339373	0.17183	1.975054	0.048262	0.090959
CNAG_04584	5023.115	-0.37536	0.08561	-4.38459	1.16E-05	5.04E-05
CNAG_04583	3915.492	-0.15094	0.071182	-2.12044	0.033969	0.06708
CNAG_04581	1043.722	0.266535	0.087968	3.029909	0.002446	0.006599
CNAG_04580	6822.232	0.539548	0.060968	8.84964	8.78E-19	1.16E-17
CNAG_07861	1038.618	-0.26548	0.114443	-2.31979	0.020352	0.042989
CNAG_04576	470.3276	0.658918	0.137879	4.778966	1.76E-06	8.74E-06
CNAG_12946	101.079	0.847513	0.278962	3.038101	0.002381	0.00644
CNAG_04574	421.3525	0.303036	0.130579	2.320711	0.020302	0.042929
CNAG_07862	3529.849	3.24875	0.101401	32.0385	3.18E-225	8.06E-223
CNAG_07863	9204.621	0.164756	0.061792	2.666294	0.007669	0.018254
CNAG_07865	2910.96	0.638582	0.06793	9.400564	5.43E-21	8.11E-20
CNAG_04566	6738.779	0.498377	0.058991	8.448338	2.95E-17	3.66E-16
CNAG_04563	681.139	-0.37478	0.102049	-3.6725	0.00024	0.000813
CNAG_04561	415.2263	-0.37961	0.135514	-2.80129	0.00509	0.012706
CNAG_04558	168.6141	-0.48007	0.198081	-2.4236	0.015367	0.033677
CNAG_04557	214.2613	-0.65441	0.164819	-3.9705	7.17E-05	0.000271
CNAG_04553	22.25841	2.198796	0.496576	4.427918	9.51E-06	4.20E-05

CNAG_07868	313.003	0.313	0.142876	2.190714	0.028473	0.057741
CNAG_01461	1376.557	0.305404	0.08516	3.586228	0.000335	0.001101
CNAG_08002	3324.865	0.257863	0.07764	3.321243	0.000896	0.002663
CNAG_01463	689.0847	1.226532	0.110004	11.14989	7.17E-29	1.41E-27
CNAG_12948	60.59855	1.066155	0.277743	3.838636	0.000124	0.000445
CNAG_12949	33.59957	1.552233	0.379464	4.090592	4.30E-05	0.000168
CNAG_01464	24947.22	2.40627	0.111411	21.59807	1.87E-103	1.44E-101
CNAG_12950	46.04185	1.588659	0.340773	4.661932	3.13E-06	1.50E-05
CNAG_01466	1205.658	1.258303	0.099472	12.64984	1.12E-36	2.80E-35
CNAG_12951	105.0709	0.858918	0.24154	3.556014	0.000377	0.001222
CNAG_01470	8810.195	1.076782	0.067061	16.05675	5.13E-58	2.06E-56
CNAG_01471	2889.329	1.259458	0.066853	18.83915	3.61E-79	1.97E-77
CNAG_01472	1168.69	0.586746	0.081189	7.226899	4.94E-13	4.87E-12
CNAG_01473	125.1268	-1.39423	0.207695	-6.71287	1.91E-11	1.63E-10
CNAG_12954	134.3387	-0.40986	0.207863	-1.97175	0.048638	0.091469
CNAG_01476	2297.719	-0.47753	0.094121	-5.07354	3.90E-07	2.12E-06
CNAG_01477	2027.065	-0.3524	0.080666	-4.36864	1.25E-05	5.38E-05
CNAG_01478	526.8113	-0.57606	0.109365	-5.26735	1.38E-07	7.95E-07
CNAG_01479	478.8856	-0.48638	0.110156	-4.41532	1.01E-05	4.44E-05
CNAG_01480	41098.03	-0.27179	0.068454	-3.97032	7.18E-05	0.000271
CNAG_01483	1019.242	-0.37575	0.087711	-4.28396	1.84E-05	7.67E-05
CNAG_01486	58753.56	-0.22501	0.073343	-3.06788	0.002156	0.005882
CNAG_01489	163.2601	-1.14674	0.196217	-5.84425	5.09E-09	3.47E-08
CNAG_01490	974.2733	1.606308	0.08912	18.02419	1.26E-72	6.35E-71
CNAG_01491	1418.504	-0.29094	0.0889	-3.27272	0.001065	0.003111
CNAG_01492	8256.777	-0.47459	0.059285	-8.00518	1.19E-15	1.37E-14
CNAG_01493	262.0623	-0.5317	0.170893	-3.11132	0.001863	0.005174
CNAG_12960	40.52686	-1.62232	0.34909	-4.64729	3.36E-06	1.60E-05
CNAG_01494	89.34482	-1.52058	0.254935	-5.96457	2.45E-09	1.73E-08
CNAG_01495	949.4206	0.249432	0.110688	2.253456	0.02423	0.050139
CNAG_01497	242.2539	0.607731	0.141238	4.302873	1.69E-05	7.10E-05
CNAG_01498	805.089	0.271448	0.090935	2.985096	0.002835	0.007537
CNAG_01499	518.1244	1.041728	0.103803	10.03558	1.06E-23	1.77E-22
CNAG_12961	63.64945	1.117699	0.350592	3.18803	0.001432	0.004062
CNAG_12962	82.6556	2.60468	0.282783	9.210893	3.23E-20	4.63E-19
CNAG_01504	1330.407	0.267762	0.089134	3.004033	0.002664	0.007121
CNAG_01506	153.4773	1.626237	0.235438	6.907281	4.94E-12	4.46E-11
CNAG_01510	3037.481	0.14779	0.065251	2.264946	0.023516	0.048834
CNAG_12963	197.832	0.501321	0.172703	2.902797	0.003698	0.009543
CNAG_01512	1267.667	2.138551	0.089649	23.8546	9.07E-126	8.38E-124
CNAG_01513	608.1731	-0.82321	0.104226	-7.89831	2.83E-15	3.17E-14
CNAG_07590	2199.896	-0.36899	0.080652	-4.57507	4.76E-06	2.22E-05
CNAG_07591	1254.248	-0.28875	0.083045	-3.47702	0.000507	0.001595
CNAG_01519	385.5613	0.38999	0.118415	3.293426	0.00099	0.002916
CNAG_01520	1384.16	-0.18404	0.074207	-2.48014	0.013133	0.029439
CNAG_01522	1641.649	0.236411	0.087314	2.707603	0.006777	0.016336
CNAG_01523	3989.639	-0.42349	0.062392	-6.78757	1.14E-11	1.00E-10

CNAG_01524	1657.691	-0.25648	0.075093	-3.41549	0.000637	0.001962
CNAG_01525	288.6828	0.7402	0.15021	4.927772	8.32E-07	4.31E-06
CNAG_12967	212.7659	2.26393	0.180268	12.5587	3.56E-36	8.75E-35
CNAG_01526	4339.263	0.978428	0.063971	15.29485	8.28E-53	3.00E-51
CNAG_12968	105.7123	2.605074	0.244708	10.64566	1.83E-26	3.29E-25
CNAG_01527	54.83171	3.064558	0.374606	8.180747	2.82E-16	3.33E-15
CNAG_01528	1676.61	0.813218	0.07537	10.78962	3.85E-27	7.18E-26
CNAG_01529	1498.115	0.421954	0.089243	4.728143	2.27E-06	1.11E-05
CNAG_01531	1097.263	-0.48729	0.082688	-5.89304	3.79E-09	2.62E-08
CNAG_01532	1899.261	-0.2116	0.074875	-2.82598	0.004714	0.011847
CNAG_01533	1807.66	1.184298	0.074606	15.87396	9.60E-57	3.79E-55
CNAG_01535	4783.081	0.218647	0.058337	3.748006	0.000178	0.00062
CNAG_12969	34.47326	1.944206	0.419607	4.633397	3.60E-06	1.71E-05
CNAG_01539	12073.55	0.381822	0.082222	4.643815	3.42E-06	1.63E-05
CNAG_12970	56.32869	0.995131	0.286485	3.473585	0.000514	0.001614
CNAG_01540	1088.248	1.248253	0.155188	8.04349	8.73E-16	1.01E-14
CNAG_01541	356.5454	1.247839	0.159994	7.799262	6.23E-15	6.81E-14
CNAG_01542	292.1181	3.475725	0.176933	19.64431	6.47E-86	3.95E-84
CNAG_01544	5537.488	0.26776	0.063192	4.237261	2.26E-05	9.26E-05
CNAG_01545	878.7536	1.799638	0.093674	19.21172	2.95E-82	1.68E-80
CNAG_01548	5614.319	-0.35258	0.060141	-5.86259	4.56E-09	3.12E-08
CNAG_01549	3194.767	-0.15862	0.069562	-2.2803	0.02259	0.047128
CNAG_01550	1473.259	-0.18119	0.08237	-2.19973	0.027826	0.056586
CNAG_01551	574.8408	-0.46076	0.128727	-3.57941	0.000344	0.001128
CNAG_01552	743.696	-0.28	0.10527	-2.65984	0.007818	0.018569
CNAG_01554	1353.316	-0.27083	0.091349	-2.96477	0.003029	0.007988
CNAG_01558	10926.01	-0.55204	0.108619	-5.08237	3.73E-07	2.03E-06
CNAG_01560	76.6077	-0.63483	0.282605	-2.24636	0.024681	0.050967
CNAG_01561	1869.352	-0.30235	0.090247	-3.35022	0.000807	0.002433
CNAG_01562	19430.9	-1.69192	0.0822	-20.583	3.90E-94	2.60E-92
CNAG_01563	1579.579	-0.26382	0.088601	-2.97766	0.002905	0.007695
CNAG_01565	2401.765	-0.39105	0.085111	-4.59453	4.34E-06	2.04E-05
CNAG_01566	1006.604	-0.29912	0.091931	-3.25375	0.001139	0.003306
CNAG_01567	210.4955	0.855731	0.156917	5.453407	4.94E-08	3.00E-07
CNAG_01568	6099.012	-0.13795	0.070223	-1.96441	0.049482	0.092885
CNAG_01572	684.6005	0.745701	0.114481	6.513766	7.33E-11	5.99E-10
CNAG_01574	4257.227	0.925974	0.086721	10.6776	1.30E-26	2.35E-25
CNAG_01575	5299.924	0.134021	0.065439	2.04804	0.040556	0.078448
CNAG_01576	1723.864	0.192725	0.082484	2.33653	0.019464	0.041392
CNAG_01577	33814.57	-0.32209	0.060027	-5.36583	8.06E-08	4.79E-07
CNAG_01583	2504.068	-0.16111	0.069648	-2.31319	0.020712	0.043614
CNAG_01584	673.3143	-0.52874	0.138357	-3.82154	0.000133	0.000473
CNAG_01586	14952.77	-0.20053	0.087723	-2.28598	0.022255	0.046538
CNAG_01587	2048.136	-0.21102	0.08286	-2.54677	0.010873	0.024936
CNAG_01592	184.6095	0.466178	0.164894	2.827129	0.004697	0.011819
CNAG_01593	3671.467	0.138407	0.068534	2.019535	0.043432	0.08314
CNAG_01594	5685.922	0.721405	0.080867	8.920836	4.63E-19	6.25E-18

CNAG_01596	1793.527	0.459464	0.08178	5.6183	1.93E-08	1.22E-07
CNAG_01598	4256.041	-0.27364	0.060394	-4.53092	5.87E-06	2.70E-05
CNAG_01600	1839.798	-0.22207	0.094672	-2.34573	0.01899	0.040512
CNAG_01601	463.6564	0.836511	0.111035	7.533777	4.93E-14	5.15E-13
CNAG_01602	1170.942	0.349538	0.09021	3.874694	0.000107	0.000389
CNAG_01603	4136.171	0.480547	0.070807	6.786708	1.15E-11	1.00E-10
CNAG_01604	907.9513	1.641777	0.096552	17.00409	7.66E-65	3.56E-63
CNAG_01605	542.7246	1.283427	0.144915	8.856392	8.26E-19	1.10E-17
CNAG_01609	2448.384	-0.34005	0.067907	-5.00765	5.51E-07	2.93E-06
CNAG_01610	1005.981	-0.3936	0.091212	-4.31525	1.59E-05	6.73E-05
CNAG_12977	56.54712	0.971854	0.292528	3.32226	0.000893	0.002656
CNAG_01613	1914.763	0.196603	0.079877	2.461319	0.013843	0.0308
CNAG_01614	1556.004	-0.35452	0.08283	-4.28011	1.87E-05	7.79E-05
CNAG_01615	626.4529	-0.75215	0.110819	-6.78724	1.14E-11	1.00E-10
CNAG_01616	3300.052	0.178521	0.062502	2.856266	0.004287	0.010912
CNAG_07593	1550.832	0.34153	0.076507	4.464034	8.04E-06	3.61E-05
CNAG_01619	682.8328	0.426307	0.094918	4.491339	7.08E-06	3.22E-05
CNAG_12978	89.51796	0.95793	0.257369	3.722003	0.000198	0.000682
CNAG_01621	2845.052	0.637304	0.107021	5.954916	2.60E-09	1.82E-08
CNAG_01622	781.0034	-0.53171	0.092864	-5.7257	1.03E-08	6.76E-08
CNAG_01626	2270.683	-0.43489	0.069986	-6.21405	5.16E-10	3.89E-09
CNAG_01627	1041.752	0.189836	0.086986	2.182386	0.029081	0.05877
CNAG_01631	1537.35	-0.30854	0.07357	-4.1939	2.74E-05	0.000111
CNAG_01633	672.9171	-0.25283	0.102377	-2.46963	0.013525	0.030186
CNAG_01634	4382.875	0.15542	0.067085	2.316762	0.020517	0.043258
CNAG_01636	1143.986	-0.4319	0.08655	-4.99016	6.03E-07	3.19E-06
CNAG_01637	3100.924	-0.14912	0.070443	-2.11694	0.034265	0.067565
CNAG_01639	7860.516	0.670802	0.061101	10.97866	4.84E-28	9.30E-27
CNAG_01641	5827.808	0.214536	0.061867	3.467713	0.000525	0.001645
CNAG_01643	1254.507	0.251656	0.090631	2.776701	0.005491	0.013595
CNAG_01644	1938.18	-0.79862	0.071023	-11.2445	2.47E-29	4.96E-28
CNAG_01645	138.2921	0.872557	0.258752	3.372172	0.000746	0.002273
CNAG_01648	23364.05	-0.67409	0.08938	-7.54187	4.63E-14	4.85E-13
CNAG_01650	1619.552	-0.33577	0.105119	-3.19418	0.001402	0.003988
CNAG_01651	202.7324	-0.34428	0.171565	-2.00669	0.044783	0.085425
CNAG_01652	1375.289	0.215648	0.086548	2.491655	0.012715	0.02862
CNAG_01653	4598.715	5.715659	0.112314	50.89017	0	0
CNAG_12980	7.150087	3.78792	1.08427	3.493521	0.000477	0.001509
CNAG_01654	1252.45	-0.15913	0.078893	-2.01707	0.043688	0.083553
CNAG_12981	23.98624	1.001018	0.482929	2.072806	0.03819	0.074439
CNAG_01655	4844.696	0.218883	0.057978	3.775253	0.00016	0.000563
CNAG_01656	531.8065	0.318019	0.119968	2.650857	0.008029	0.018987
CNAG_01657	6646.726	-0.51629	0.063787	-8.09395	5.78E-16	6.71E-15
CNAG_08004	133.7896	0.453826	0.209691	2.164261	0.030444	0.061144
CNAG_01665	719.8431	-0.4759	0.098035	-4.8544	1.21E-06	6.12E-06
CNAG_01668	83.38234	2.890876	0.293048	9.864846	5.91E-23	9.55E-22
CNAG_01669	853.5653	-0.25013	0.102011	-2.45199	0.014207	0.031497

CNAG_01670	305.2806	-0.42014	0.15055	-2.79069	0.00526	0.013085
CNAG_01673	1737.929	-0.24697	0.084616	-2.9187	0.003515	0.009131
CNAG_01674	135.0829	1.416179	0.196797	7.196133	6.19E-13	6.02E-12
CNAG_01675	892.9991	-0.36346	0.08554	-4.24898	2.15E-05	8.85E-05
CNAG_01676	1916.713	-0.23701	0.078181	-3.03162	0.002432	0.006571
CNAG_01677	1664.872	-0.25322	0.079057	-3.20299	0.00136	0.00388
CNAG_01678	2611.137	-0.34663	0.071138	-4.87262	1.10E-06	5.61E-06
CNAG_01679	4209.135	-0.22739	0.071076	-3.19928	0.001378	0.003928
CNAG_12984	37.67941	1.507274	0.393089	3.834438	0.000126	0.000452
CNAG_12985	903.1014	-0.6411	0.099749	-6.42708	1.30E-10	1.04E-09
CNAG_12987	174.0332	0.721531	0.228729	3.154519	0.001608	0.004518
CNAG_01682	10676.52	-0.22357	0.056548	-3.95361	7.70E-05	0.000288
CNAG_07596	129.8324	-0.61808	0.294091	-2.10165	0.035584	0.069827
CNAG_12989	54.4519	1.026096	0.317762	3.229137	0.001242	0.003565
CNAG_01683	8836.042	-0.39295	0.088546	-4.43782	9.09E-06	4.03E-05
CNAG_01686	1682.173	-0.73475	0.114495	-6.41732	1.39E-10	1.11E-09
CNAG_12991	98.29096	0.988705	0.245458	4.027998	5.63E-05	0.000216
CNAG_01689	61.47184	1.073352	0.275009	3.902973	9.50E-05	0.00035
CNAG_01690	732.5371	0.480825	0.12549	3.831566	0.000127	0.000456
CNAG_01691	1071.48	0.230483	0.092772	2.484395	0.012977	0.029162
CNAG_01692	1509.529	-0.42052	0.080276	-5.23837	1.62E-07	9.23E-07
CNAG_01695	178.2578	0.868498	0.194138	4.473616	7.69E-06	3.47E-05
CNAG_01696	4271.873	0.147149	0.073674	1.997294	0.045793	0.087005
CNAG_01698	575.0264	0.269561	0.112148	2.403611	0.016234	0.035337
CNAG_01699	917.0707	0.53031	0.087209	6.080891	1.20E-09	8.70E-09
CNAG_07598	5238.9	0.273053	0.060077	4.545019	5.49E-06	2.54E-05
CNAG_12994	304.4258	0.613866	0.162969	3.766773	0.000165	0.00058
CNAG_01701	1334.867	0.495819	0.11267	4.400626	1.08E-05	4.71E-05
CNAG_01702	613.8367	0.622193	0.098176	6.33753	2.33E-10	1.83E-09
CNAG_01704	7899.673	0.987604	0.076643	12.88582	5.41E-38	1.39E-36
CNAG_08005	161.9636	0.65394	0.183442	3.564833	0.000364	0.001186
CNAG_01707	1467.605	-0.22823	0.093477	-2.44154	0.014625	0.032275
CNAG_01709	5519.012	0.363363	0.063593	5.713917	1.10E-08	7.21E-08
CNAG_01711	3793.125	-0.32132	0.061957	-5.18616	2.15E-07	1.20E-06
CNAG_01712	1178.347	-0.32988	0.0825	-3.99852	6.37E-05	0.000242
CNAG_01714	71.10475	1.580522	0.339507	4.655351	3.23E-06	1.54E-05
CNAG_01715	5198.375	0.3136	0.066773	4.696494	2.65E-06	1.28E-05
CNAG_01716	1253.266	-0.21381	0.105038	-2.03551	0.0418	0.080395
CNAG_01717	799.2558	-0.32894	0.095659	-3.43872	0.000584	0.001819
CNAG_01719	216.5917	0.866299	0.170337	5.0858	3.66E-07	2.00E-06
CNAG_01721	5368.869	1.2109	0.081862	14.79195	1.65E-49	5.52E-48
CNAG_01722	3888.825	-0.44049	0.069956	-6.29669	3.04E-10	2.35E-09
CNAG_01727	46865.22	-0.21154	0.077938	-2.71418	0.006644	0.016053
CNAG_01728	1034.499	-0.18899	0.087027	-2.17158	0.029888	0.06019
CNAG_01729	2163.749	0.324275	0.072816	4.45334	8.45E-06	3.78E-05
CNAG_01732	1722.953	0.177347	0.082928	2.13857	0.03247	0.064607
CNAG_01733	6152.503	-0.4459	0.072623	-6.13997	8.25E-10	6.07E-09

CNAG_01735	1185.054	-1.32795	0.107729	-12.3267	6.51E-35	1.52E-33
CNAG_01736	424.4583	-0.64424	0.119243	-5.40275	6.56E-08	3.94E-07
CNAG_01737	3588.793	-0.98039	0.088476	-11.0808	1.56E-28	3.01E-27
CNAG_01741	408.2973	-0.3335	0.134152	-2.48599	0.012919	0.029048
CNAG_01742	71.82826	-2.70509	0.309668	-8.73544	2.43E-18	3.15E-17
CNAG_01743	309.4366	-0.27237	0.128234	-2.12404	0.033667	0.066629
CNAG_01744	8366.651	-0.74415	0.059045	-12.603	2.03E-36	5.05E-35
CNAG_01745	7925.409	-0.81936	0.07861	-10.4231	1.95E-25	3.42E-24
CNAG_07600	602.9265	0.444179	0.104401	4.254537	2.09E-05	8.66E-05
CNAG_01750	1572.564	-0.52569	0.138319	-3.8006	0.000144	0.000512
CNAG_01751	699.2442	-0.43716	0.166093	-2.63203	0.008488	0.019985
CNAG_01753	3988.523	-0.78571	0.08605	-9.13088	6.79E-20	9.55E-19
CNAG_01754	2200.266	0.327471	0.067356	4.861809	1.16E-06	5.90E-06
CNAG_01755	5101.757	0.823059	0.067757	12.14717	5.94E-34	1.36E-32
CNAG_08006	243.1226	-0.36328	0.152244	-2.38618	0.017025	0.03683
CNAG_01758	113.8314	-0.60563	0.20299	-2.98354	0.002849	0.007568
CNAG_01759	23.32815	-1.71668	0.505748	-3.39434	0.000688	0.002108
CNAG_07601	476.9639	-0.63311	0.118323	-5.35069	8.76E-08	5.18E-07
CNAG_01760	331.2346	0.360463	0.145384	2.479382	0.013161	0.029493
CNAG_01761	12938.34	-0.58808	0.065206	-9.01876	1.90E-19	2.62E-18
CNAG_01764	1003.135	0.181351	0.086159	2.104854	0.035304	0.069379
CNAG_07604	425.2685	0.538403	0.136278	3.95077	7.79E-05	0.000292
CNAG_01768	1589.034	0.46817	0.100593	4.654119	3.25E-06	1.55E-05
CNAG_01773	553.691	-0.38653	0.108699	-3.55599	0.000377	0.001222
CNAG_13002	10.22632	1.428006	0.673682	2.119704	0.034031	0.067153
CNAG_01776	210.2642	0.664073	0.17325	3.833036	0.000127	0.000454
CNAG_01777	1491.263	-0.43144	0.109777	-3.93013	8.49E-05	0.000316
CNAG_01778	1573.026	-0.19981	0.081022	-2.46615	0.013657	0.030438
CNAG_01780	5157.107	-0.19855	0.074355	-2.67031	0.007578	0.018052
CNAG_01789	2955.037	-0.19095	0.074624	-2.55887	0.010501	0.024153
CNAG_01790	887.3911	-0.26427	0.094135	-2.8073	0.004996	0.012487
CNAG_01792	1778.478	0.176669	0.080189	2.203161	0.027583	0.056176
CNAG_13006	148.1429	-0.43681	0.187328	-2.33181	0.019711	0.041857
CNAG_01793	875.4238	0.329598	0.091211	3.613569	0.000302	0.001
CNAG_01794	1648.059	2.146031	0.253511	8.465243	2.56E-17	3.17E-16
CNAG_01795	311.5357	-0.51386	0.135346	-3.79661	0.000147	0.000519
CNAG_01796	401.0963	1.360657	0.157787	8.623405	6.50E-18	8.25E-17
CNAG_01797	1956.758	-0.2351	0.076226	-3.08425	0.002041	0.005602
CNAG_01799	11767.21	0.414891	0.073045	5.679941	1.35E-08	8.70E-08
CNAG_01800	2297.946	0.635342	0.111531	5.69654	1.22E-08	7.95E-08
CNAG_01802	2235.179	1.428805	0.0743	19.23027	2.07E-82	1.18E-80
CNAG_01803	598.5234	0.375238	0.127735	2.937622	0.003307	0.008633
CNAG_13007	138.9394	-0.46862	0.201726	-2.32304	0.020177	0.042718
CNAG_01809	1317.502	-0.30367	0.100212	-3.0303	0.002443	0.006593
CNAG_01813	12079.99	-0.18008	0.053289	-3.37928	0.000727	0.00222
CNAG_01816	197.1749	0.412194	0.167593	2.459498	0.013913	0.03091
CNAG_01824	3502.081	-0.15331	0.071417	-2.14664	0.031822	0.063534

CNAG_01828	1181.246	-0.29515	0.080366	-3.67263	0.00024	0.000813
CNAG_01832	590.2706	-0.35989	0.109653	-3.28205	0.001031	0.003022
CNAG_01834	1847.638	-0.22534	0.073489	-3.06638	0.002167	0.005908
CNAG_01835	2243.464	-0.2597	0.068826	-3.77332	0.000161	0.000567
CNAG_01836	265.7054	1.31533	0.158303	8.308921	9.66E-17	1.16E-15
CNAG_01837	2838.49	0.403177	0.068289	5.904015	3.55E-09	2.46E-08
CNAG_01839	2406.459	-0.58345	0.079497	-7.33924	2.15E-13	2.16E-12
CNAG_01841	223.9684	-0.54736	0.208703	-2.62268	0.008724	0.020488
CNAG_01843	2542.432	0.362402	0.082424	4.396779	1.10E-05	4.79E-05
CNAG_01845	5825.171	0.348485	0.065588	5.313268	1.08E-07	6.29E-07
CNAG_01846	15998.86	2.751478	0.104733	26.2714	4.07E-152	5.34E-150
CNAG_01847	286.6384	-0.49452	0.175329	-2.82053	0.004794	0.012024
CNAG_01848	2772.102	0.198753	0.066662	2.981506	0.002868	0.007606
CNAG_01852	2696.529	-0.31517	0.084929	-3.71095	0.000206	0.000709
CNAG_01853	1228.237	-0.25172	0.089942	-2.79864	0.005132	0.012795
CNAG_01854	23628.15	1.238998	0.074159	16.7074	1.16E-62	5.17E-61
CNAG_01855	1191.397	0.751194	0.092781	8.096388	5.66E-16	6.58E-15
CNAG_01856	285.7656	0.771902	0.143829	5.366808	8.01E-08	4.77E-07
CNAG_01858	35.12308	-1.74619	0.408628	-4.27331	1.93E-05	8.01E-05
CNAG_01861	1214.456	0.400776	0.097047	4.129687	3.63E-05	0.000144
CNAG_01863	4493.617	-0.28124	0.0817	-3.44236	0.000577	0.001798
CNAG_01864	1286.559	-0.22993	0.079395	-2.89601	0.003779	0.009733
CNAG_01865	543.3564	-0.53712	0.113284	-4.74134	2.12E-06	1.04E-05
CNAG_01868	1163.654	-0.33455	0.08183	-4.08839	4.34E-05	0.00017
CNAG_01870	4177.938	-0.23732	0.068355	-3.47191	0.000517	0.001624
CNAG_01871	917.5772	-0.60201	0.099415	-6.05557	1.40E-09	1.01E-08
CNAG_01872	202.0434	-0.43154	0.159522	-2.70519	0.006827	0.016426
CNAG_01876	1341.534	0.191366	0.086227	2.219337	0.026464	0.054184
CNAG_01879	891.4736	-0.48108	0.087953	-5.46972	4.51E-08	2.75E-07
CNAG_01880	1421.555	-0.32957	0.096415	-3.41826	0.00063	0.001948
CNAG_01881	4172.309	0.477224	0.063003	7.574655	3.60E-14	3.80E-13
CNAG_01882	505.909	-0.79235	0.150005	-5.28212	1.28E-07	7.38E-07
CNAG_07611	24.65236	1.225239	0.513554	2.385805	0.017042	0.036849
CNAG_07612	78.18468	-1.58011	0.252796	-6.25055	4.09E-10	3.11E-09
CNAG_13015	51.00597	-2.46097	0.34141	-7.20824	5.67E-13	5.53E-12
CNAG_01889	525.0027	-0.65005	0.125863	-5.16471	2.41E-07	1.34E-06
CNAG_01891	686.3532	0.271657	0.098382	2.761252	0.005758	0.014161
CNAG_01892	1516.158	-0.34158	0.103976	-3.28516	0.001019	0.002993
CNAG_01893	1435.606	-0.58651	0.082182	-7.13677	9.55E-13	9.13E-12
CNAG_01897	4476.417	-0.39616	0.064235	-6.16735	6.94E-10	5.17E-09
CNAG_01898	2982.163	0.22751	0.07377	3.084048	0.002042	0.005604
CNAG_01900	933.323	-0.3075	0.089566	-3.43322	0.000596	0.001855
CNAG_13017	139.236	0.674592	0.218205	3.091558	0.001991	0.005492
CNAG_01901	10.63588	1.565967	0.749014	2.090705	0.036555	0.071593
CNAG_01902	2555.075	-1.00509	0.070696	-14.2171	7.18E-46	2.24E-44
CNAG_01904	1335.877	0.879144	0.077598	11.32953	9.37E-30	1.91E-28
CNAG_01905	2345.201	0.26769	0.070123	3.817421	0.000135	0.00048

CNAG_01906	500.7163	-0.37253	0.106922	-3.48412	0.000494	0.001557
CNAG_13020	58.37859	1.942038	0.30549	6.357135	2.06E-10	1.61E-09
CNAG_01908	2458.667	2.669371	0.074433	35.86269	1.17E-281	4.99E-279
CNAG_01909	465.5863	-0.28999	0.114521	-2.53222	0.011334	0.0259
CNAG_01910	1176.004	-0.19055	0.083785	-2.27427	0.02295	0.047782
CNAG_01912	4896.121	-0.18496	0.07763	-2.38252	0.017195	0.03715
CNAG_01913	262.207	0.784953	0.156873	5.003762	5.62E-07	2.99E-06
CNAG_01914	1274.712	-0.38559	0.09194	-4.19396	2.74E-05	0.000111
CNAG_01915	8669.28	0.19609	0.060475	3.242484	0.001185	0.003424
CNAG_01916	2262.735	0.500304	0.080793	6.192434	5.92E-10	4.43E-09
CNAG_13022	57.86226	0.628216	0.287988	2.181396	0.029154	0.058888
CNAG_01919	691.5372	-0.29125	0.102711	-2.83567	0.004573	0.011536
CNAG_01921	126.286	1.176132	0.192878	6.09781	1.08E-09	7.85E-09
CNAG_13023	22.80761	1.698066	0.475521	3.570963	0.000356	0.001161
CNAG_01924	766.6617	-0.77417	0.099483	-7.78188	7.15E-15	7.80E-14
CNAG_01925	895.5735	1.000138	0.147703	6.771256	1.28E-11	1.11E-10
CNAG_01926	396.4383	-0.32935	0.120152	-2.7411	0.006123	0.014928
CNAG_01928	1237.556	-0.3454	0.082081	-4.20809	2.58E-05	0.000104
CNAG_01929	356.4759	0.385753	0.129667	2.974945	0.00293	0.007754
CNAG_01930	1989.422	0.441793	0.077675	5.687731	1.29E-08	8.33E-08
CNAG_01936	92.20081	1.08337	0.310347	3.490836	0.000482	0.001523
CNAG_01938	3047.72	-0.16272	0.073748	-2.2064	0.027356	0.055787
CNAG_01942	383.3093	0.465814	0.125448	3.713204	0.000205	0.000704
CNAG_13033	101.4379	2.029565	0.236753	8.572493	1.01E-17	1.27E-16
CNAG_01944	21.33703	1.662007	0.508729	3.26698	0.001087	0.003164
CNAG_01945	108.3131	0.884363	0.256992	3.441207	0.000579	0.001804
CNAG_01946	1006.426	0.623742	0.121121	5.149757	2.61E-07	1.45E-06
CNAG_01947	1911.37	1.926479	0.294211	6.547957	5.83E-11	4.81E-10
CNAG_01948	1470.265	0.57997	0.076498	7.581545	3.41E-14	3.60E-13
CNAG_01949	6496.545	2.615744	0.249348	10.49035	9.57E-26	1.70E-24
CNAG_01951	30819.16	-0.16828	0.078562	-2.14204	0.032191	0.064113
CNAG_13034	9.492597	2.109664	0.745262	2.830766	0.004644	0.011711
CNAG_01952	1255.404	2.418577	0.088607	27.29554	4.79E-164	6.96E-162
CNAG_01953	591.5363	1.47608	0.137614	10.72624	7.66E-27	1.40E-25
CNAG_13035	55.9242	0.611677	0.289233	2.114826	0.034445	0.067887
CNAG_01954	63.37195	1.49256	0.28891	5.166169	2.39E-07	1.33E-06
CNAG_13036	86.06338	0.589837	0.232775	2.533938	0.011279	0.02578
CNAG_01958	2965.044	-0.29114	0.07328	-3.97294	7.10E-05	0.000268
CNAG_01959	2274.248	-0.48614	0.071523	-6.797	1.07E-11	9.41E-11
CNAG_01960	200.1807	0.927886	0.201372	4.60783	4.07E-06	1.92E-05
CNAG_01961	5575.974	-0.23193	0.062352	-3.71968	0.000199	0.000687
CNAG_01962	779.5885	-0.62658	0.092112	-6.80239	1.03E-11	9.09E-11
CNAG_01963	230.2542	1.400707	0.171364	8.173891	2.99E-16	3.52E-15
CNAG_01964	823.0087	1.427093	0.131233	10.87449	1.53E-27	2.89E-26
CNAG_01965	712.9114	3.776662	0.123628	30.5486	5.90E-205	1.26E-202
CNAG_07613	119.1671	0.791825	0.221288	3.578262	0.000346	0.001132
CNAG_01968	27.29019	1.252348	0.442629	2.82934	0.004664	0.011752

CNAG_01969	288.5904	0.315845	0.135785	2.326068	0.020015	0.04242
CNAG_01972	1662.282	0.182883	0.090417	2.02267	0.043107	0.082597
CNAG_01973	2606.961	-0.25759	0.072965	-3.53031	0.000415	0.001335
CNAG_01975	1376.224	0.292511	0.08335	3.509419	0.000449	0.00143
CNAG_01976	36463.94	-0.14777	0.073041	-2.02311	0.043062	0.082529
CNAG_01978	298.1564	-0.39556	0.146136	-2.70677	0.006794	0.016362
CNAG_01981	6712.118	-0.38677	0.058731	-6.58543	4.54E-11	3.78E-10
CNAG_01982	590.2342	0.316366	0.114025	2.77454	0.005528	0.013681
CNAG_13040	104.4732	0.614048	0.305563	2.009563	0.044478	0.084922
CNAG_01983	2146.188	0.232218	0.085183	2.726127	0.006408	0.015543
CNAG_01984	41288.61	-0.2575	0.052897	-4.86789	1.13E-06	5.73E-06
CNAG_01986	1370.437	0.549155	0.138761	3.957573	7.57E-05	0.000284
CNAG_13042	97.06914	1.173745	0.249535	4.70372	2.55E-06	1.24E-05
CNAG_01987	1945.859	0.279401	0.07462	3.744334	0.000181	0.000628
CNAG_01990	53628.57	-0.21297	0.064098	-3.32251	0.000892	0.002654
CNAG_01991	13334.45	0.467756	0.064838	7.214241	5.42E-13	5.31E-12
CNAG_01992	124.5093	0.60408	0.207295	2.914115	0.003567	0.009236
CNAG_01993	250.2752	1.384313	0.147024	9.415557	4.71E-21	7.08E-20
CNAG_01994	79.20204	0.848565	0.31072	2.730965	0.006315	0.015345
CNAG_01995	211.6614	4.571167	0.251791	18.15463	1.18E-73	6.15E-72
CNAG_01997	3030.574	-0.3811	0.087784	-4.34131	1.42E-05	6.05E-05
CNAG_08012	2522.001	-0.36475	0.088265	-4.13239	3.59E-05	0.000143
CNAG_01999	587.0693	-0.43362	0.131545	-3.29632	0.00098	0.002891
CNAG_02000	1532.042	-0.58237	0.093263	-6.2444	4.25E-10	3.23E-09
CNAG_02001	1213.861	0.327511	0.081883	3.999746	6.34E-05	0.000241
CNAG_08013	771.5507	0.577659	0.098232	5.880571	4.09E-09	2.81E-08
CNAG_02004	2923.978	0.17213	0.072826	2.363565	0.0181	0.038879
CNAG_02005	28.89942	0.879785	0.413641	2.126929	0.033426	0.066265
CNAG_02006	1150.643	-0.19878	0.089313	-2.22566	0.026037	0.053401
CNAG_02007	7954.175	-0.21758	0.065455	-3.32404	0.000887	0.002643
CNAG_02009	1181.017	0.61645	0.097821	6.301846	2.94E-10	2.28E-09
CNAG_02011	436.7141	0.737595	0.114254	6.455752	1.08E-10	8.67E-10
CNAG_02013	377.7059	-0.31418	0.12679	-2.47798	0.013213	0.029593
CNAG_13043	158.7199	-0.7279	0.188416	-3.86326	0.000112	0.000405
CNAG_02016	39.74492	1.545181	0.372789	4.144923	3.40E-05	0.000136
CNAG_06956	286.1157	-0.75683	0.146823	-5.15473	2.54E-07	1.41E-06
CNAG_13045	129.7858	-0.86677	0.196633	-4.40807	1.04E-05	4.57E-05
CNAG_05986	32.70191	-1.16556	0.383307	-3.0408	0.00236	0.006388
CNAG_05989	689.5866	-0.2277	0.108077	-2.10682	0.035133	0.069077
CNAG_05990	311.9276	-0.60866	0.156517	-3.88878	0.000101	0.000369
CNAG_05991	133.3364	-0.58012	0.194439	-2.98354	0.002849	0.007568
CNAG_05992	117.9794	-0.4065	0.205735	-1.97583	0.048174	0.090827
CNAG_05993	609.2117	-0.29545	0.129258	-2.28573	0.02227	0.046556
CNAG_05995	1646.063	0.265477	0.080752	3.287553	0.001011	0.00297
CNAG_08014	115.8849	2.176807	0.233437	9.325015	1.11E-20	1.63E-19
CNAG_05996	1208.327	1.454345	0.08796	16.53424	2.08E-61	8.99E-60
CNAG_05997	5903.273	0.406413	0.068149	5.963592	2.47E-09	1.73E-08

CNAG_05998	1330.26	0.330297	0.082014	4.027321	5.64E-05	0.000217
CNAG_05999	793.7848	-0.33679	0.118588	-2.84002	0.004511	0.011394
CNAG_06000	1593.41	-0.81578	0.097535	-8.36398	6.06E-17	7.40E-16
CNAG_06001	1061.194	-0.55827	0.10364	-5.38668	7.18E-08	4.30E-07
CNAG_06003	96.05841	1.073406	0.239272	4.486128	7.25E-06	3.29E-05
CNAG_06006	1337.591	-0.19985	0.101377	-1.97137	0.048681	0.091529
CNAG_06008	1089.697	1.689201	0.087381	19.3314	2.92E-83	1.69E-81
CNAG_06009	318.5889	3.02462	0.191414	15.80148	3.04E-56	1.18E-54
CNAG_06013	550.9414	-0.34588	0.119565	-2.89283	0.003818	0.009823
CNAG_06016	1712.809	0.32708	0.086208	3.794069	0.000148	0.000524
CNAG_06017	179.0337	1.433507	0.2129	6.733229	1.66E-11	1.44E-10
CNAG_06019	1310.989	-0.31379	0.104875	-2.99204	0.002771	0.007387
CNAG_06020	138.6405	0.508835	0.202929	2.507448	0.012161	0.027487
CNAG_13051	41.70825	1.152891	0.34835	3.309578	0.000934	0.002771
CNAG_06021	2483.568	0.325283	0.093775	3.468755	0.000523	0.00164
CNAG_06023	372.2496	-0.46857	0.118126	-3.96671	7.29E-05	0.000275
CNAG_06026	1510.393	-0.55345	0.085353	-6.48425	8.92E-11	7.26E-10
CNAG_06027	22.49336	1.528335	0.530436	2.881279	0.003961	0.010136
CNAG_06028	704.3165	0.561989	0.100926	5.568338	2.57E-08	1.61E-07
CNAG_06029	575.1484	0.403116	0.103639	3.889624	0.0001	0.000368
CNAG_06030	605.1458	0.301057	0.105542	2.852482	0.004338	0.011025
CNAG_06031	561.4359	1.289433	0.166866	7.727373	1.10E-14	1.19E-13
CNAG_13053	30.75384	0.856914	0.422144	2.029907	0.042366	0.081349
CNAG_06034	1971.194	0.302798	0.095144	3.182518	0.00146	0.00413
CNAG_06035	621.4994	0.45642	0.111385	4.097679	4.17E-05	0.000164
CNAG_06048	1024.304	-0.35404	0.088657	-3.99335	6.51E-05	0.000247
CNAG_06049	1460.385	-0.37932	0.075874	-4.99936	5.75E-07	3.05E-06
CNAG_13055	120.7904	1.592523	0.258688	6.156164	7.45E-10	5.52E-09
CNAG_06050	242.161	1.400902	0.158714	8.826596	1.08E-18	1.42E-17
CNAG_06051	1416.182	1.119044	0.117372	9.534187	1.51E-21	2.32E-20
CNAG_06052	1601.523	1.18166	0.09909	11.92513	8.75E-33	1.93E-31
CNAG_13056	36.67863	3.621844	0.488247	7.41806	1.19E-13	1.21E-12
CNAG_13057	419.0959	1.852964	0.135818	13.64304	2.22E-42	6.36E-41
CNAG_07897	245.1798	2.044705	0.205963	9.927539	3.16E-23	5.21E-22
CNAG_06055	797.6791	1.554841	0.140197	11.09039	1.40E-28	2.71E-27
CNAG_06056	574.2178	1.371578	0.112027	12.24325	1.83E-34	4.24E-33
CNAG_07898	61.39751	0.907028	0.281703	3.219807	0.001283	0.003676
CNAG_06060	2277.261	0.450991	0.06852	6.58191	4.64E-11	3.86E-10
CNAG_13059	28.3067	0.953243	0.407362	2.340042	0.019282	0.041037
CNAG_06061	6057.817	-0.17186	0.064262	-2.6744	0.007486	0.017865
CNAG_06063	3616.425	1.916314	0.075788	25.28524	4.64E-141	5.47E-139
CNAG_06064	760.7448	-0.26508	0.098757	-2.68417	0.007271	0.017408
CNAG_06065	1262.188	-0.44786	0.09474	-4.72728	2.28E-06	1.11E-05
CNAG_06066	53.947	1.084753	0.323953	3.348491	0.000813	0.002445
CNAG_06071	588.7678	-0.27209	0.098723	-2.75613	0.005849	0.01435
CNAG_13060	67.92098	3.620986	0.345695	10.47451	1.13E-25	2.00E-24
CNAG_13061	242.6266	0.297439	0.146207	2.034371	0.041914	0.080596

CNAG_06074	667.979	-0.71923	0.142361	-5.05214	4.37E-07	2.36E-06
CNAG_06075	6904.386	-0.59812	0.111767	-5.35147	8.72E-08	5.16E-07
CNAG_06076	2588.443	-0.19895	0.064415	-3.08862	0.002011	0.005537
CNAG_06077	2655.354	-0.1602	0.070875	-2.26028	0.023804	0.049356
CNAG_06081	10366.04	0.326016	0.061819	5.273712	1.34E-07	7.69E-07
CNAG_13063	622.956	-0.54888	0.107201	-5.12007	3.05E-07	1.68E-06
CNAG_06085	1299.183	1.545004	0.096948	15.93637	3.54E-57	1.40E-55
CNAG_06087	1564.722	-0.3967	0.084444	-4.69782	2.63E-06	1.27E-05
CNAG_06088	3460.521	-0.64215	0.06924	-9.27419	1.79E-20	2.60E-19
CNAG_13065	147.311	0.689533	0.198155	3.479765	0.000502	0.001579
CNAG_06094	181.1933	-0.66954	0.161576	-4.14382	3.42E-05	0.000136
CNAG_06095	96910.64	-0.17046	0.068971	-2.47141	0.013458	0.03006
CNAG_06096	19198.5	0.891302	0.065677	13.57101	5.95E-42	1.68E-40
CNAG_06098	550.6801	-0.50113	0.157746	-3.17679	0.001489	0.004204
CNAG_06100	944.2578	0.26789	0.099255	2.699005	0.006955	0.016713
CNAG_06103	3545.761	0.193404	0.070975	2.724971	0.006431	0.015593
CNAG_06104	578.2713	-1.68605	0.129917	-12.978	1.63E-38	4.26E-37
CNAG_06105	124.0833	-0.5208	0.205719	-2.53159	0.011355	0.02591
CNAG_06106	4552.307	-0.65812	0.082537	-7.97358	1.54E-15	1.75E-14
CNAG_06107	829.337	-0.49575	0.105225	-4.71132	2.46E-06	1.20E-05
CNAG_06108	527.6809	-0.28681	0.107867	-2.65897	0.007838	0.018601
CNAG_06113	75164.22	-0.16212	0.078411	-2.06752	0.038685	0.075254
CNAG_06115	492.0308	0.848253	0.145606	5.825674	5.69E-09	3.87E-08
CNAG_07904	1571.281	-0.16973	0.084795	-2.00168	0.045319	0.086285
CNAG_08016	1690.669	0.202593	0.103064	1.965711	0.049332	0.092667
CNAG_06118	1508.229	-0.30628	0.083472	-3.66928	0.000243	0.000822
CNAG_06120	1248.313	-0.32016	0.087532	-3.65766	0.000255	0.000856
CNAG_06122	1735.724	1.010294	0.083476	12.10283	1.02E-33	2.32E-32
CNAG_06125	335725.8	-0.13705	0.061986	-2.21095	0.027039	0.055262
CNAG_06127	4200.349	0.182284	0.081086	2.24802	0.024575	0.050774
CNAG_06128	368.371	-0.43578	0.138907	-3.13717	0.001706	0.004766
CNAG_06129	840.768	-0.30394	0.091505	-3.32162	0.000895	0.002661
CNAG_06134	7555.058	0.384672	0.062628	6.142199	8.14E-10	6.00E-09
CNAG_06136	857.0709	0.716895	0.132636	5.404989	6.48E-08	3.90E-07
CNAG_06138	3742.196	0.936832	0.068164	13.74383	5.55E-43	1.65E-41
CNAG_06140	4832.84	-0.42677	0.098625	-4.32724	1.51E-05	6.41E-05
CNAG_06142	1008.835	0.292933	0.087214	3.358783	0.000783	0.002366
CNAG_06144	29151.08	-0.19456	0.077815	-2.50035	0.012407	0.027966
CNAG_06145	976.6917	0.504107	0.084807	5.944182	2.78E-09	1.95E-08
CNAG_08018	1138.376	-0.32246	0.103092	-3.12787	0.001761	0.004906
CNAG_06147	2795.445	0.420297	0.118113	3.558435	0.000373	0.001213
CNAG_06148	915.1966	-0.2154	0.096744	-2.22649	0.025981	0.053313
CNAG_06151	3050.127	-0.1853	0.068447	-2.70724	0.006785	0.016349
CNAG_06157	1199.916	-0.46231	0.080013	-5.77791	7.56E-09	5.05E-08
CNAG_06158	2255.801	0.288699	0.074495	3.875419	0.000106	0.000388
CNAG_06162	1210.178	0.4851	0.089849	5.399037	6.70E-08	4.02E-07
CNAG_06164	279.5409	-0.33329	0.137011	-2.43258	0.014992	0.032967

CNAG_06167	3750.047	0.202484	0.065486	3.092036	0.001988	0.005485
CNAG_06168	8342.771	0.67608	0.06055	11.16566	6.00E-29	1.19E-27
CNAG_06169	699.7949	0.417947	0.148001	2.823953	0.004744	0.011915
CNAG_06170	2297.452	-0.40925	0.074049	-5.52676	3.26E-08	2.02E-07
CNAG_06173	742.1221	-0.48392	0.110559	-4.377	1.20E-05	5.20E-05
CNAG_06175	5945.228	0.151396	0.06554	2.309958	0.02089	0.043921
CNAG_07908	9364.887	0.972432	0.061766	15.74389	7.56E-56	2.89E-54
CNAG_07909	226.7368	1.642422	0.17373	9.453875	3.27E-21	4.94E-20
CNAG_06180	4559.364	1.064392	0.070396	15.12006	1.19E-51	4.15E-50
CNAG_06185	956.5084	0.261933	0.088356	2.964507	0.003032	0.007993
CNAG_06186	728.82	0.804746	0.117282	6.861646	6.81E-12	6.08E-11
CNAG_13076	13.54578	4.823436	0.995011	4.847619	1.25E-06	6.32E-06
CNAG_07911	1420.995	3.564105	0.103134	34.55804	1.08E-261	3.84E-259
CNAG_06188	1137.533	1.44968	0.0894	16.21573	3.90E-59	1.63E-57
CNAG_06191	863.9461	0.894418	0.090507	9.882334	4.97E-23	8.11E-22
CNAG_06193	1175.562	0.324524	0.097253	3.336889	0.000847	0.00254
CNAG_06194	60.05841	2.520912	0.333222	7.565253	3.87E-14	4.07E-13
CNAG_06197	1265.012	0.240693	0.102278	2.353317	0.018607	0.039809
CNAG_06200	877.4704	-0.50661	0.090065	-5.62495	1.86E-08	1.18E-07
CNAG_06201	1035.546	-0.48669	0.115313	-4.22058	2.44E-05	9.90E-05
CNAG_13081	16.88897	1.862264	0.565813	3.291306	0.000997	0.002935
CNAG_06203	529.379	2.603123	0.131862	19.74131	9.53E-87	5.91E-85
CNAG_13082	1143.947	0.750989	0.090652	8.284277	1.19E-16	1.42E-15
CNAG_06204	6314.81	0.817562	0.079304	10.30921	6.40E-25	1.12E-23
CNAG_06206	3388.933	0.212776	0.078276	2.718286	0.006562	0.015869
CNAG_06207	1011.767	0.577332	0.135437	4.262732	2.02E-05	8.37E-05
CNAG_06209	96.07593	0.701206	0.243208	2.883155	0.003937	0.010092
CNAG_07912	102.1258	0.52765	0.233454	2.260189	0.02381	0.049356
CNAG_07914	4610.116	-0.33693	0.065704	-5.128	2.93E-07	1.62E-06
CNAG_06214	1331.993	-0.69368	0.088761	-7.81516	5.49E-15	6.03E-14
CNAG_13084	54.5278	0.823867	0.340893	2.416792	0.015658	0.034212
CNAG_06216	714.2896	-0.73861	0.115523	-6.39363	1.62E-10	1.28E-09
CNAG_06218	912.1412	-0.32425	0.099237	-3.2674	0.001085	0.003161
CNAG_06219	1652.598	-0.63384	0.07432	-8.52857	1.48E-17	1.84E-16
CNAG_06220	5880.974	-1.09341	0.237786	-4.59829	4.26E-06	2.00E-05
CNAG_13085	204.7453	0.370549	0.179168	2.068161	0.038625	0.075178
CNAG_06221	1490.518	0.353308	0.08189	4.314443	1.60E-05	6.75E-05
CNAG_06222	40352.15	-0.22759	0.083236	-2.73422	0.006253	0.015212
CNAG_06223	1287.324	-0.36252	0.079304	-4.57122	4.85E-06	2.26E-05
CNAG_06224	1273.854	-0.70778	0.091452	-7.73937	9.99E-15	1.08E-13
CNAG_06225	117.6344	1.666085	0.291801	5.709664	1.13E-08	7.38E-08
CNAG_06226	6759.996	1.052833	0.068709	15.32317	5.35E-53	1.95E-51
CNAG_06227	1493.97	0.477649	0.073582	6.491343	8.51E-11	6.93E-10
CNAG_06229	1433.508	-0.45409	0.083906	-5.41191	6.24E-08	3.76E-07
CNAG_13087	39.74563	-0.77674	0.35392	-2.19467	0.028187	0.057233
CNAG_06231	52349.31	-0.25058	0.068141	-3.6774	0.000236	0.000798
CNAG_06232	630.749	-0.33194	0.098703	-3.36297	0.000771	0.002335

CNAG_13089	251.0394	-0.37796	0.190396	-1.98514	0.047129	0.089147
CNAG_06235	2461.189	-0.33366	0.065392	-5.10244	3.35E-07	1.84E-06
CNAG_06236	1597.785	-0.37297	0.083137	-4.48629	7.25E-06	3.29E-05
CNAG_06237	2329.597	0.61961	0.070181	8.828768	1.06E-18	1.39E-17
CNAG_06238	347.165	1.815958	0.183838	9.878025	5.18E-23	8.44E-22
CNAG_06239	486.1242	1.991559	0.124585	15.9855	1.61E-57	6.43E-56
CNAG_06240	8882.27	0.59066	0.064634	9.138538	6.33E-20	8.92E-19
CNAG_06241	5295.096	2.089887	0.089923	23.24093	1.76E-119	1.52E-117
CNAG_06242	10034.01	1.763058	0.083113	21.21286	7.27E-100	5.27E-98
CNAG_13091	163.4241	1.28055	0.207752	6.163842	7.10E-10	5.28E-09
CNAG_06246	22335.11	-0.41135	0.06396	-6.43134	1.26E-10	1.01E-09
CNAG_06248	7399.99	0.365301	0.06006	6.082271	1.18E-09	8.63E-09
CNAG_06249	266.6735	-0.38017	0.149471	-2.54342	0.010977	0.025141
CNAG_06251	413.9747	1.03906	0.136379	7.618905	2.56E-14	2.72E-13
CNAG_06252	1041.909	1.326685	0.105836	12.53526	4.79E-36	1.17E-34
CNAG_07917	1077.024	0.718706	0.092749	7.748932	9.27E-15	1.00E-13
CNAG_07008	105.091	0.479389	0.211324	2.268501	0.023299	0.048433
CNAG_07011	1308.056	-0.52008	0.081101	-6.41275	1.43E-10	1.14E-09
CNAG_06985	783.3759	-0.29372	0.090293	-3.25292	0.001142	0.003313
CNAG_06986	536.5764	-0.26137	0.113186	-2.3092	0.020933	0.043997
CNAG_13094	98.35944	-0.76575	0.236349	-3.23991	0.001196	0.00345
CNAG_06259	497.613	2.121455	0.129204	16.4194	1.39E-60	5.97E-59
CNAG_06260	1622.098	-1.06868	0.089609	-11.9261	8.65E-33	1.91E-31
CNAG_07921	1130.885	-0.27129	0.108792	-2.49371	0.012642	0.028463
CNAG_07922	1158.185	0.212093	0.082418	2.573369	0.010071	0.023243
CNAG_06265	313.4909	-0.54958	0.128161	-4.28819	1.80E-05	7.54E-05
CNAG_13099	504.2077	0.339164	0.115112	2.946367	0.003215	0.008425
CNAG_13100	104.0605	0.967556	0.220699	4.38406	1.16E-05	5.05E-05
CNAG_07924	2240.013	0.543821	0.095782	5.677689	1.37E-08	8.81E-08
CNAG_06272	414.8203	0.386822	0.12341	3.134432	0.001722	0.004804
CNAG_06274	11719.11	0.50048	0.098466	5.082783	3.72E-07	2.03E-06
CNAG_06276	1341.008	0.244322	0.088258	2.768283	0.005635	0.013905
CNAG_13104	137.3187	0.400409	0.183225	2.185343	0.028864	0.058417
CNAG_06277	4805.237	0.260363	0.061524	4.231894	2.32E-05	9.46E-05
CNAG_06278	2264.581	-0.70064	0.07133	-9.82254	9.00E-23	1.44E-21
CNAG_07927	275.1368	-0.99311	0.150304	-6.60733	3.91E-11	3.28E-10
CNAG_06282	1391.969	-0.17421	0.084346	-2.06542	0.038884	0.075573
CNAG_06283	1173.627	-0.39456	0.087046	-4.53273	5.82E-06	2.68E-05
CNAG_06285	283.9851	-0.39309	0.141898	-2.7702	0.005602	0.013832
CNAG_06287	2701.728	-0.21755	0.07807	-2.78658	0.005327	0.013232
CNAG_06288	2280.038	-0.34792	0.071449	-4.86945	1.12E-06	5.70E-06
CNAG_06290	520.0453	0.763066	0.175897	4.338148	1.44E-05	6.13E-05
CNAG_06291	462.3321	1.668814	0.171552	9.727741	2.30E-22	3.62E-21
CNAG_06293	308.205	-0.61219	0.162471	-3.76798	0.000165	0.000578
CNAG_06294	169.4888	0.884217	0.177635	4.977719	6.43E-07	3.39E-06
CNAG_06297	528.3282	1.075917	0.170456	6.311977	2.75E-10	2.14E-09
CNAG_06298	2896.7	1.696721	0.108696	15.6098	6.24E-55	2.34E-53

CNAG_06299	1131.778	0.359395	0.08458	4.249191	2.15E-05	8.84E-05
CNAG_13108	37.07939	3.903559	0.497704	7.843141	4.39E-15	4.87E-14
CNAG_06300	2562.578	0.198764	0.076731	2.590384	0.009587	0.022251
CNAG_06302	130.6913	0.642227	0.214628	2.992284	0.002769	0.007384
CNAG_13110	499.3985	-0.43706	0.108981	-4.01041	6.06E-05	0.000231
CNAG_06309	266.4615	0.726636	0.220066	3.301894	0.00096	0.002841
CNAG_06310	768.9585	0.579779	0.094345	6.145277	7.98E-10	5.90E-09
CNAG_06311	1551.019	0.783696	0.083548	9.380198	6.58E-21	9.82E-20
CNAG_06313	9990.69	-0.63781	0.067048	-9.51272	1.86E-21	2.84E-20
CNAG_06314	10552.4	-0.30207	0.062388	-4.84188	1.29E-06	6.49E-06
CNAG_06315	2746.821	-0.32645	0.080551	-4.05268	5.06E-05	0.000196
CNAG_06316	3258.513	0.252416	0.0652	3.871404	0.000108	0.000393
CNAG_06320	2514.648	-0.63531	0.0908	-6.99688	2.62E-12	2.41E-11
CNAG_06322	583.5923	-0.65075	0.105965	-6.14119	8.19E-10	6.03E-09
CNAG_13113	27.33144	0.977446	0.450385	2.170244	0.029988	0.060333
CNAG_06323	446.9104	0.368275	0.130214	2.828241	0.00468	0.011787
CNAG_06324	623.0548	0.501308	0.126768	3.954524	7.67E-05	0.000288
CNAG_06325	2462.014	-0.3428	0.077922	-4.39921	1.09E-05	4.74E-05
CNAG_06327	2259.374	0.513931	0.085976	5.977576	2.26E-09	1.60E-08
CNAG_06329	334.934	1.806592	0.148268	12.18463	3.75E-34	8.67E-33
CNAG_06330	452.9037	1.002877	0.121954	8.223411	1.98E-16	2.34E-15
CNAG_06331	425.2422	1.409816	0.133084	10.59347	3.20E-26	5.72E-25
CNAG_06336	1375.636	1.009596	0.098514	10.2483	1.20E-24	2.08E-23
CNAG_13119	5.120899	3.352802	1.177326	2.84781	0.004402	0.011157
CNAG_06339	979.4262	0.541918	0.109369	4.954938	7.24E-07	3.78E-06
CNAG_06340	2790.547	0.157381	0.070267	2.239773	0.025106	0.0517
CNAG_06343	743.7277	-0.59004	0.095168	-6.20003	5.65E-10	4.23E-09
CNAG_06345	1046.091	-0.29816	0.10011	-2.97835	0.002898	0.00768
CNAG_06346	2931.502	-2.57746	0.110513	-23.3228	2.61E-120	2.28E-118
CNAG_13120	217.6473	-0.4933	0.178212	-2.76803	0.00564	0.013907
CNAG_06347	6774.593	-1.70235	0.104142	-16.3464	4.62E-60	1.94E-58
CNAG_06348	2493.18	0.894631	0.083543	10.70866	9.27E-27	1.69E-25
CNAG_06352	1952.128	-0.26694	0.088523	-3.01546	0.002566	0.006885
CNAG_06354	500.3047	0.348926	0.118581	2.942527	0.003255	0.008511
CNAG_06355	270.5631	-0.56647	0.156106	-3.62872	0.000285	0.000948
CNAG_06357	546.7886	0.278497	0.107703	2.585777	0.009716	0.022525
CNAG_06358	412.6546	0.400756	0.127434	3.144817	0.001662	0.004655
CNAG_06360	1040.911	-0.29692	0.097068	-3.05893	0.002221	0.006044
CNAG_06361	4017.749	0.159689	0.068474	2.332099	0.019696	0.041841
CNAG_06365	462.6091	-0.37851	0.117389	-3.22443	0.001262	0.00362
CNAG_06368	598.3816	-0.55191	0.111201	-4.96323	6.93E-07	3.64E-06
CNAG_06369	649.3642	-0.30932	0.115299	-2.68278	0.007301	0.017464
CNAG_06370	2652.519	-0.64194	0.074864	-8.57482	9.92E-18	1.25E-16
CNAG_06371	347.7795	0.47815	0.13178	3.62841	0.000285	0.000949
CNAG_06372	306.4218	1.461071	0.159491	9.160825	5.15E-20	7.31E-19
CNAG_06374	4844.457	2.723878	0.125019	21.78774	3.03E-105	2.39E-103
CNAG_06375	205.9661	-1.91934	0.168377	-11.3991	4.22E-30	8.74E-29

CNAG_06377	36996.68	-0.14868	0.060824	-2.4445	0.014505	0.032039
CNAG_06380	1283.374	-0.29316	0.080891	-3.62416	0.00029	0.000963
CNAG_13122	114.8944	-0.53216	0.262526	-2.02707	0.042655	0.081827
CNAG_06383	328.1615	0.419895	0.124114	3.38315	0.000717	0.00219
CNAG_06384	1232.776	0.253804	0.07891	3.216391	0.001298	0.003719
CNAG_06385	783.0877	-0.55124	0.101281	-5.44269	5.25E-08	3.18E-07
CNAG_06386	1360.394	-0.27166	0.081582	-3.32996	0.000869	0.002598
CNAG_06390	197.7906	0.495755	0.156032	3.177266	0.001487	0.004199
CNAG_06392	942.3536	-0.5187	0.086557	-5.99263	2.06E-09	1.47E-08
CNAG_06393	619.6121	-0.39589	0.106384	-3.72131	0.000198	0.000684
CNAG_06396	386.4617	1.130254	0.143391	7.882301	3.21E-15	3.58E-14
CNAG_07936	1947.441	0.942426	0.074811	12.59746	2.18E-36	5.40E-35
CNAG_06399	536.6224	-0.4277	0.115282	-3.71006	0.000207	0.000711
CNAG_06400	83351.39	0.254143	0.062007	4.098622	4.16E-05	0.000164
CNAG_13126	55.22741	-1.19703	0.321486	-3.72344	0.000197	0.000679
CNAG_06401	693.5521	-0.30016	0.098346	-3.05207	0.002273	0.006176
CNAG_06402	2104.44	0.154411	0.076362	2.022097	0.043166	0.08269
CNAG_06404	13693.85	2.96606	0.088749	33.42069	6.86E-245	2.15E-242
CNAG_06405	1358.961	0.653041	0.078045	8.367489	5.89E-17	7.20E-16
CNAG_06406	869.3644	-0.18996	0.089779	-2.11583	0.034359	0.067735
CNAG_06407	3803.798	0.530166	0.093615	5.663251	1.49E-08	9.56E-08
CNAG_06408	286.9907	0.457636	0.151614	3.018421	0.002541	0.006827
CNAG_06409	408.345	-0.28766	0.140775	-2.0434	0.041012	0.079068
CNAG_06410	964.3909	-0.49483	0.094926	-5.21282	1.86E-07	1.05E-06
CNAG_06412	1460.213	0.295385	0.080279	3.679491	0.000234	0.000792
CNAG_06413	139.3752	-0.97412	0.198095	-4.91744	8.77E-07	4.53E-06
CNAG_06414	675.0753	0.529846	0.100466	5.273907	1.34E-07	7.69E-07
CNAG_06415	1235.296	0.617758	0.100579	6.142019	8.15E-10	6.00E-09
CNAG_06416	1637.345	-0.33266	0.086926	-3.82701	0.00013	0.000464
CNAG_06417	413.5796	-0.38518	0.131967	-2.91876	0.003514	0.009131
CNAG_06419	365.4729	-0.462	0.125225	-3.68939	0.000225	0.000764
CNAG_06420	3447.366	-0.38047	0.06931	-5.48944	4.03E-08	2.47E-07
CNAG_06421	3500.238	-0.36298	0.096297	-3.76936	0.000164	0.000575
CNAG_06423	524.0156	-0.50053	0.109359	-4.57692	4.72E-06	2.20E-05
CNAG_06424	997.0277	-0.21405	0.088959	-2.40613	0.016122	0.035113
CNAG_07937	1956.56	-0.24564	0.070498	-3.48437	0.000493	0.001556
CNAG_07938	1180.727	0.508002	0.090545	5.610514	2.02E-08	1.28E-07
CNAG_06431	489.4846	-0.58259	0.110332	-5.28031	1.29E-07	7.45E-07
CNAG_06432	2323.579	-0.6242	0.1146	-5.44678	5.13E-08	3.11E-07
CNAG_07939	6074.986	-1.02914	0.323527	-3.18099	0.001468	0.004151
CNAG_13129	173.1888	-1.20276	0.208458	-5.76977	7.94E-09	5.28E-08
CNAG_06437	1621.943	-0.43715	0.101297	-4.31549	1.59E-05	6.72E-05
CNAG_13131	37.91817	-2.07831	0.389572	-5.33486	9.56E-08	5.63E-07
CNAG_06438	9.217949	-2.69543	0.819005	-3.2911	0.000998	0.002936
CNAG_06439	1943.37	-0.30506	0.074442	-4.09799	4.17E-05	0.000164
CNAG_06440	929.5038	-0.63638	0.085002	-7.48665	7.07E-14	7.34E-13
CNAG_07940	212.011	-0.6067	0.158348	-3.83144	0.000127	0.000456

CNAG_06443	30492.8	0.604726	0.071925	8.4077	4.18E-17	5.14E-16
CNAG_06444	1027.411	-0.2933	0.087437	-3.35439	0.000795	0.0024
CNAG_06446	2453.337	0.239762	0.065518	3.65949	0.000253	0.000851
CNAG_06450	2339.413	-0.19041	0.065255	-2.91786	0.003524	0.009144
CNAG_06452	1119.728	-0.21728	0.093791	-2.31666	0.020522	0.043259
CNAG_06454	1592.05	0.252212	0.075873	3.324151	0.000887	0.002642
CNAG_07941	4426.618	0.668801	0.063591	10.51714	7.20E-26	1.28E-24
CNAG_06459	850.5833	-0.2595	0.106357	-2.43988	0.014692	0.032389
CNAG_06460	2673.243	0.798789	0.091071	8.771092	1.77E-18	2.30E-17
CNAG_06464	984.8213	-0.23495	0.09345	-2.51414	0.011932	0.027053
CNAG_06465	1808.912	-0.43012	0.089119	-4.82637	1.39E-06	6.97E-06
CNAG_06466	439.3082	-0.56078	0.118296	-4.74048	2.13E-06	1.05E-05
CNAG_06468	5194.15	0.138931	0.063491	2.188217	0.028654	0.058036
CNAG_06469	3147.177	-0.25146	0.070108	-3.58677	0.000335	0.001099
CNAG_06472	6559.502	0.271922	0.066145	4.111002	3.94E-05	0.000155
CNAG_06474	5957.511	-0.28969	0.074896	-3.86792	0.00011	0.000399
CNAG_13134	193.5674	-0.71452	0.166967	-4.2794	1.87E-05	7.81E-05
CNAG_06482	514.8719	-0.22775	0.107191	-2.12475	0.033607	0.066543
CNAG_06483	1310.504	0.760094	0.081233	9.356977	8.21E-21	1.22E-19
CNAG_06484	99.51664	0.482815	0.21742	2.220657	0.026374	0.054038
CNAG_06485	299.9853	1.067345	0.148075	7.20815	5.67E-13	5.53E-12
CNAG_06487	1604.724	0.213406	0.074043	2.88218	0.003949	0.010113
CNAG_07943	639.3607	2.191708	0.140217	15.63079	4.49E-55	1.70E-53
CNAG_06489	5676.122	-0.23618	0.102988	-2.29329	0.021831	0.045756
CNAG_06493	1000.479	-0.91741	0.401657	-2.28406	0.022368	0.046725
CNAG_06497	651.1786	-0.35554	0.125625	-2.83014	0.004653	0.01173
CNAG_06499	1032.991	0.194832	0.090542	2.151844	0.03141	0.062866
CNAG_06500	809.2307	0.396824	0.11305	3.510161	0.000448	0.001427
CNAG_06501	1678.38	0.33362	0.09109	3.662526	0.00025	0.000843
CNAG_06503	144.7733	2.906641	0.217985	13.33416	1.46E-40	4.06E-39
CNAG_06505	938.9552	-0.36145	0.085326	-4.23609	2.27E-05	9.30E-05
CNAG_06506	591.7325	-0.27989	0.105249	-2.65933	0.00783	0.018592
CNAG_06508	5537.129	0.310233	0.078795	3.937197	8.24E-05	0.000307
CNAG_06510	852.5143	-0.28957	0.098872	-2.92874	0.003403	0.008872
CNAG_06512	1636.087	2.070139	0.087247	23.72733	1.88E-124	1.70E-122
CNAG_06515	281.6783	0.285864	0.133761	2.137128	0.032588	0.064793
CNAG_06516	389.0127	1.622666	0.141532	11.46499	1.98E-30	4.11E-29
CNAG_06517	3773.514	-0.3888	0.088491	-4.39366	1.11E-05	4.85E-05
CNAG_06518	14.21652	-1.63396	0.604301	-2.70389	0.006853	0.01648
CNAG_06519	447.9681	-0.34629	0.119468	-2.89861	0.003748	0.009665
CNAG_06520	489.9676	-0.49224	0.10786	-4.56373	5.03E-06	2.33E-05
CNAG_05333	7.392897	1.796938	0.823977	2.180811	0.029197	0.058946
CNAG_05334	39.09676	-0.86111	0.368264	-2.33829	0.019372	0.041209
CNAG_05336	1149.731	0.426093	0.115467	3.69019	0.000224	0.000762
CNAG_05337	1128.001	0.365005	0.087651	4.164291	3.12E-05	0.000125
CNAG_05339	1526.021	-0.22635	0.078217	-2.89382	0.003806	0.009795
CNAG_05340	434.9493	0.726496	0.171315	4.240709	2.23E-05	9.14E-05

CNAG_05341	418.5035	1.758742	0.161275	10.90526	1.09E-27	2.08E-26
CNAG_05343	1408.173	-0.27566	0.108032	-2.55163	0.010722	0.024618
CNAG_05344	2795.028	0.325526	0.06552	4.968315	6.75E-07	3.55E-06
CNAG_13145	31.44399	0.892927	0.407816	2.189532	0.028558	0.057886
CNAG_05346	138.4807	-0.44265	0.194257	-2.27868	0.022686	0.047316
CNAG_07032	3311.287	-0.56318	0.070164	-8.02669	1.00E-15	1.15E-14
CNAG_05348	5097.948	-0.3839	0.062235	-6.16856	6.89E-10	5.14E-09
CNAG_05351	9219.481	0.224393	0.074366	3.017398	0.00255	0.006846
CNAG_05352	1618.148	0.277652	0.073304	3.787657	0.000152	0.000537
CNAG_05354	1083.89	-0.28498	0.090557	-3.14702	0.001649	0.004621
CNAG_05356	178.5587	2.692567	0.20133	13.37392	8.59E-41	2.40E-39
CNAG_05357	47.59441	2.860715	0.400359	7.145369	8.98E-13	8.60E-12
CNAG_13147	410.1513	2.654722	0.168263	15.77726	4.46E-56	1.71E-54
CNAG_13148	58.25823	5.722221	0.620385	9.223654	2.87E-20	4.13E-19
CNAG_07870	11.23604	7.015354	1.31581	5.331586	9.74E-08	5.72E-07
CNAG_05358	208.6	3.555495	0.213659	16.64096	3.52E-62	1.56E-60
CNAG_05359	1097.254	-0.18067	0.090698	-1.99202	0.046369	0.087913
CNAG_08021	133.945	0.755641	0.186702	4.047314	5.18E-05	0.0002
CNAG_08022	2447.666	0.258058	0.071739	3.597174	0.000322	0.00106
CNAG_05364	385.3338	0.250311	0.126242	1.982783	0.047392	0.089602
CNAG_05365	2773.571	0.578135	0.064035	9.028372	1.74E-19	2.41E-18
CNAG_07873	1565.861	-0.21141	0.078837	-2.68159	0.007327	0.017517
CNAG_05371	2507.333	0.249586	0.07444	3.35286	0.0008	0.002412
CNAG_05373	648.8763	-0.24618	0.10971	-2.24393	0.024837	0.051237
CNAG_05374	1535.251	0.463392	0.076708	6.040989	1.53E-09	1.10E-08
CNAG_05375	24284.28	-0.12965	0.059117	-2.19309	0.028301	0.057422
CNAG_05377	139.8929	2.768565	0.233364	11.86372	1.83E-32	3.97E-31
CNAG_05378	462.8577	0.390106	0.123238	3.165478	0.001548	0.004362
CNAG_05379	2638.46	1.277535	0.070527	18.11405	2.47E-73	1.27E-71
CNAG_05380	1371.999	0.418383	0.08129	5.146782	2.65E-07	1.47E-06
CNAG_05381	294.0478	1.960582	0.483928	4.051393	5.09E-05	0.000197
CNAG_13155	87.76734	1.73097	0.245987	7.036842	1.97E-12	1.84E-11
CNAG_13156	158.357	1.798877	0.182141	9.876298	5.27E-23	8.57E-22
CNAG_05383	725.2154	0.462342	0.141284	3.272432	0.001066	0.003113
CNAG_05384	2069.077	-0.26614	0.071099	-3.74326	0.000182	0.000631
CNAG_05387	8274.853	-1.49092	0.118865	-12.5431	4.34E-36	1.06E-34
CNAG_05391	1752.761	-0.28231	0.092638	-3.04741	0.002308	0.006262
CNAG_05392	180.3928	0.364724	0.185614	1.964957	0.049419	0.092809
CNAG_05393	1007.089	-0.17374	0.083384	-2.08366	0.037192	0.072666
CNAG_05394	657.3767	0.266104	0.099691	2.669276	0.007602	0.018103
CNAG_07875	741.0708	0.470929	0.126647	3.718441	0.0002	0.00069
CNAG_05396	320.2088	-0.63738	0.147515	-4.32077	1.55E-05	6.58E-05
CNAG_05397	203.441	1.256531	0.161326	7.788765	6.77E-15	7.39E-14
CNAG_05398	3823.886	0.344647	0.068756	5.0126	5.37E-07	2.86E-06
CNAG_05401	1654.963	0.385792	0.074345	5.189206	2.11E-07	1.18E-06
CNAG_05402	2982.656	-0.24615	0.064073	-3.84169	0.000122	0.00044
CNAG_05403	483.17	0.337755	0.107583	3.139484	0.001692	0.004735

CNAG_05404	1437.738	0.469794	0.076745	6.121477	9.27E-10	6.79E-09
CNAG_05410	568.6152	0.275594	0.11574	2.381151	0.017259	0.037268
CNAG_05411	1531.784	0.811785	0.077508	10.47363	1.14E-25	2.02E-24
CNAG_05412	49.80278	2.624956	0.35428	7.409273	1.27E-13	1.29E-12
CNAG_05413	697.7319	-0.40425	0.09432	-4.28594	1.82E-05	7.61E-05
CNAG_05415	1862.954	-0.95377	0.101332	-9.41234	4.85E-21	7.29E-20
CNAG_05416	3619.854	-0.21599	0.077346	-2.79252	0.00523	0.013019
CNAG_05419	1756.011	-0.24817	0.083477	-2.97288	0.00295	0.007801
CNAG_05420	578.219	-0.36838	0.119439	-3.08424	0.002041	0.005602
CNAG_13168	218.5939	0.353241	0.15335	2.303492	0.021251	0.04461
CNAG_05423	751.8424	1.252165	0.103958	12.04488	2.06E-33	4.63E-32
CNAG_05424	15238.29	0.918624	0.068209	13.46773	2.42E-41	6.81E-40
CNAG_05425	13525.19	-0.24521	0.054121	-4.53071	5.88E-06	2.71E-05
CNAG_05426	157.1185	0.444575	0.20955	2.121572	0.033874	0.066957
CNAG_08024	359.6774	-0.44048	0.122545	-3.59443	0.000325	0.00107
CNAG_05428	1822.333	-0.27952	0.081972	-3.40995	0.00065	0.002
CNAG_05431	980.7471	-0.45015	0.10059	-4.4751	7.64E-06	3.45E-05
CNAG_05434	4727.412	0.883453	0.072218	12.23321	2.07E-34	4.78E-33
CNAG_05437	18290.44	-0.29503	0.081996	-3.5981	0.000321	0.001057
CNAG_05438	747.7716	-0.32985	0.097245	-3.39198	0.000694	0.002124
CNAG_05440	979.93	-0.41845	0.09656	-4.33362	1.47E-05	6.25E-05
CNAG_05441	446.8728	0.441889	0.141676	3.119018	0.001815	0.005047
CNAG_13178	80.53969	-0.70486	0.246033	-2.8649	0.004171	0.010642
CNAG_05444	1658.575	1.572879	0.089059	17.66111	8.36E-70	4.12E-68
CNAG_05448	18.30143	1.514748	0.535586	2.828204	0.004681	0.011787
CNAG_07876	162.754	0.626942	0.168371	3.723582	0.000196	0.000678
CNAG_05449	9037.467	0.658089	0.104626	6.289936	3.18E-10	2.45E-09
CNAG_05450	350.4994	0.322299	0.135856	2.372363	0.017675	0.038066
CNAG_05455	9900.422	0.278006	0.069362	4.008047	6.12E-05	0.000233
CNAG_05457	3881.833	0.152284	0.062593	2.432935	0.014977	0.032945
CNAG_05458	267.743	1.665772	0.228056	7.304223	2.79E-13	2.78E-12
CNAG_05459	3604.832	-0.29115	0.068545	-4.24762	2.16E-05	8.89E-05
CNAG_05460	465.5538	0.317645	0.119456	2.65909	0.007835	0.018599
CNAG_05461	151.1317	1.501946	0.223199	6.729187	1.71E-11	1.47E-10
CNAG_05462	9386.991	-0.19069	0.064587	-2.9525	0.003152	0.008278
CNAG_05463	1729.119	-0.37078	0.083717	-4.42896	9.47E-06	4.18E-05
CNAG_05464	688.8991	-0.46182	0.108752	-4.24652	2.17E-05	8.93E-05
CNAG_05468	1084.763	-0.29069	0.081706	-3.55773	0.000374	0.001215
CNAG_05470	837.3481	0.186505	0.087753	2.125342	0.033558	0.066464
CNAG_07878	4465.691	0.150629	0.06917	2.177654	0.029432	0.05939
CNAG_05472	61.47351	0.780361	0.30082	2.594115	0.009483	0.022056
CNAG_07879	268.513	-0.50195	0.140641	-3.56899	0.000358	0.001169
CNAG_13182	10.19985	2.465593	0.736377	3.348274	0.000813	0.002446
CNAG_05479	3602.749	2.974927	0.074789	39.77749	0	0
CNAG_05480	7815.294	-0.14832	0.069496	-2.13417	0.032829	0.065241
CNAG_13183	18.91325	-1.13916	0.536634	-2.12279	0.033771	0.066816
CNAG_07881	28.48793	-1.1824	0.455012	-2.5986	0.00936	0.021844

CNAG_05482	282.2259	-0.52215	0.134152	-3.89224	9.93E-05	0.000364
CNAG_05483	1104.585	-0.36196	0.08452	-4.2826	1.85E-05	7.71E-05
CNAG_05484	843.5024	-0.26953	0.092521	-2.91318	0.003578	0.009261
CNAG_08026	191.7243	-1.04351	0.174907	-5.96605	2.43E-09	1.72E-08
CNAG_05496	6015.336	-0.2098	0.070491	-2.97621	0.002918	0.007729
CNAG_05497	22059.24	0.836101	0.053742	15.55772	1.41E-54	5.21E-53
CNAG_05499	3806.739	-0.53986	0.06319	-8.54345	1.30E-17	1.63E-16
CNAG_05501	257.265	1.404728	0.179575	7.822495	5.18E-15	5.71E-14
CNAG_05502	1835.562	0.849001	0.070796	11.9922	3.90E-33	8.67E-32
CNAG_05503	427.9764	-0.56121	0.12423	-4.51749	6.26E-06	2.87E-05
CNAG_05505	531.6081	-0.25494	0.118831	-2.1454	0.031921	0.063698
CNAG_07883	1366.226	0.239647	0.083906	2.856143	0.004288	0.010913
CNAG_07884	5997.638	0.151863	0.074332	2.04304	0.041049	0.0791
CNAG_05507	2239.2	-0.2412	0.092981	-2.59411	0.009484	0.022056
CNAG_05508	450.7679	-0.26254	0.115065	-2.28166	0.022509	0.046972
CNAG_05509	3139.787	0.670108	0.084481	7.932049	2.16E-15	2.43E-14
CNAG_05511	4097.337	0.261935	0.071623	3.657127	0.000255	0.000857
CNAG_05512	1635.127	-0.2064	0.079579	-2.59362	0.009497	0.02208
CNAG_05516	1050.137	-0.33128	0.099137	-3.34164	0.000833	0.002501
CNAG_05517	1675.14	-0.30387	0.085639	-3.54823	0.000388	0.001254
CNAG_05518	1176.659	-0.20705	0.099841	-2.07378	0.0381	0.07428
CNAG_05519	600.5453	0.31216	0.100836	3.095719	0.001963	0.005426
CNAG_05520	605.9062	0.310193	0.10252	3.025693	0.002481	0.006679
CNAG_05521	2566.391	0.511451	0.094658	5.403152	6.55E-08	3.94E-07
CNAG_13188	140.6003	0.779488	0.190354	4.094929	4.22E-05	0.000166
CNAG_05523	210.6718	1.535548	0.176146	8.717468	2.84E-18	3.68E-17
CNAG_13189	48.45548	-0.98499	0.327617	-3.00651	0.002643	0.007068
CNAG_05524	1113.449	-0.23811	0.085922	-2.77123	0.005585	0.013792
CNAG_05525	47762.56	-0.20035	0.069676	-2.87539	0.004035	0.010317
CNAG_05526	417.127	-0.27735	0.129657	-2.13912	0.032426	0.064551
CNAG_05527	1822.543	0.346949	0.077014	4.505014	6.64E-06	3.03E-05
CNAG_05529	930.6531	-0.19277	0.087641	-2.19953	0.02784	0.056599
CNAG_05537	1254.265	0.203882	0.096141	2.120657	0.033951	0.067069
CNAG_05539	1089.8	0.376348	0.087035	4.324081	1.53E-05	6.49E-05
CNAG_05540	9583.24	0.478747	0.056126	8.529871	1.47E-17	1.83E-16
CNAG_05543	153.4894	0.931466	0.187401	4.970454	6.68E-07	3.51E-06
CNAG_05546	611.6221	-0.30986	0.105531	-2.93618	0.003323	0.008667
CNAG_05547	249.7992	0.426939	0.151693	2.814498	0.004885	0.012237
CNAG_05553	491.0408	-0.29993	0.115251	-2.60245	0.009256	0.021637
CNAG_05554	3633.556	0.669305	0.073029	9.164905	4.96E-20	7.05E-19
CNAG_05558	1017.541	0.360166	0.08532	4.221344	2.43E-05	9.87E-05
CNAG_05563	638.2739	-0.24823	0.097843	-2.53701	0.01118	0.025584
CNAG_05564	618.6709	-0.28962	0.110526	-2.62034	0.008784	0.020617
CNAG_05566	437.835	-0.2634	0.115271	-2.28503	0.022311	0.046618
CNAG_05567	723.304	-0.4347	0.092535	-4.69773	2.63E-06	1.27E-05
CNAG_05568	936.9637	-0.30043	0.085796	-3.50165	0.000462	0.001469
CNAG_05570	794.6046	-0.21971	0.106113	-2.07055	0.038401	0.074777

CNAG_05571	4359.338	0.234721	0.068251	3.439077	0.000584	0.001818
CNAG_05573	1041.657	0.393055	0.119829	3.280142	0.001038	0.003041
CNAG_05575	925.0902	-0.63836	0.088298	-7.22968	4.84E-13	4.78E-12
CNAG_05577	751.1243	-0.35032	0.089823	-3.90008	9.62E-05	0.000354
CNAG_05578	2321.189	0.608725	0.091546	6.64938	2.94E-11	2.49E-10
CNAG_05580	38.17557	0.842001	0.424086	1.985449	0.047095	0.089123
CNAG_05581	5758.291	0.688787	0.071631	9.615834	6.86E-22	1.06E-20
CNAG_05582	102.7275	0.756322	0.233934	3.233061	0.001225	0.003526
CNAG_05584	1298.366	-0.18683	0.087907	-2.12533	0.033559	0.066464
CNAG_07887	1874.824	0.592185	0.098782	5.994865	2.04E-09	1.45E-08
CNAG_05590	1923.235	0.256213	0.102205	2.506856	0.012181	0.027525
CNAG_05591	531.1361	-0.4286	0.106878	-4.0102	6.07E-05	0.000231
CNAG_05592	6611.226	0.542867	0.066572	8.154621	3.50E-16	4.11E-15
CNAG_08028	409.2906	-1.01034	0.142145	-7.10779	1.18E-12	1.12E-11
CNAG_05595	2316.093	1.548779	0.104984	14.75251	2.96E-49	9.83E-48
CNAG_05596	3681.064	-0.3663	0.082031	-4.46535	7.99E-06	3.59E-05
CNAG_05598	1502.297	0.639549	0.085936	7.442142	9.91E-14	1.02E-12
CNAG_05602	1359.724	0.821678	0.088264	9.309284	1.29E-20	1.88E-19
CNAG_05605	763.5273	0.356694	0.095247	3.744945	0.00018	0.000627
CNAG_05606	844.3683	-0.21719	0.093905	-2.31285	0.020731	0.043631
CNAG_05607	1702.556	-1.00814	0.136149	-7.40463	1.32E-13	1.33E-12
CNAG_07888	27487.9	0.43159	0.063141	6.83532	8.18E-12	7.29E-11
CNAG_05609	1873.824	0.17016	0.072405	2.350118	0.018767	0.040121
CNAG_07889	2065.437	0.172054	0.078594	2.189157	0.028585	0.057926
CNAG_05612	858.0849	-0.39181	0.088778	-4.41332	1.02E-05	4.47E-05
CNAG_05613	2079.617	0.194157	0.071366	2.720567	0.006517	0.015788
CNAG_05615	6092.74	-0.15634	0.076498	-2.04372	0.040981	0.079026
CNAG_05616	2088.405	0.37811	0.086701	4.361063	1.29E-05	5.56E-05
CNAG_05617	1777.871	0.528026	0.084429	6.254089	4.00E-10	3.04E-09
CNAG_05618	2504.828	0.184102	0.067016	2.747162	0.006011	0.014682
CNAG_05621	1299.285	-0.27786	0.0867	-3.20488	0.001351	0.003856
CNAG_05623	5529.689	-0.19553	0.072183	-2.70878	0.006753	0.016283
CNAG_05626	1033.431	0.261348	0.084245	3.102249	0.001921	0.005321
CNAG_05627	1339.486	-0.20176	0.101628	-1.98522	0.047119	0.089147
CNAG_05628	870.6824	0.344148	0.091364	3.766795	0.000165	0.00058
CNAG_05629	2227.985	0.227797	0.069822	3.262526	0.001104	0.003212
CNAG_05631	15954.73	0.862894	0.055393	15.57765	1.03E-54	3.83E-53
CNAG_13197	208.1841	0.614373	0.168216	3.652284	0.00026	0.000871
CNAG_05633	4860.968	0.559851	0.096793	5.784008	7.29E-09	4.89E-08
CNAG_05634	1010.847	-0.2994	0.08935	-3.35085	0.000806	0.002428
CNAG_05637	1077.492	0.255953	0.094103	2.719926	0.00653	0.01581
CNAG_05638	3691.335	0.177853	0.06454	2.755698	0.005857	0.014351
CNAG_05639	977.4448	-0.61942	0.095704	-6.47222	9.66E-11	7.83E-10
CNAG_05640	1256.495	-0.41975	0.086733	-4.8396	1.30E-06	6.56E-06
CNAG_05642	1394.509	-0.32203	0.09059	-3.55476	0.000378	0.001227
CNAG_05643	706.3935	-0.47755	0.144713	-3.29996	0.000967	0.002857
CNAG_05644	395.998	-0.44857	0.122862	-3.65106	0.000261	0.000875

CNAG_05645	1322.215	-0.20563	0.08607	-2.38912	0.016889	0.036587
CNAG_05650	8365.346	-0.17871	0.06013	-2.97205	0.002958	0.007817
CNAG_05652	1729.767	0.692662	0.102283	6.771996	1.27E-11	1.11E-10
CNAG_05653	1247.006	0.614528	0.101662	6.044824	1.50E-09	1.07E-08
CNAG_05654	1058.943	2.372662	0.275033	8.626834	6.31E-18	8.02E-17
CNAG_05656	170.8465	0.623933	0.174342	3.57878	0.000345	0.00113
CNAG_05657	1000.2	-1.13033	0.110885	-10.1937	2.12E-24	3.60E-23
CNAG_05658	65.61507	-1.8822	0.302228	-6.22777	4.73E-10	3.58E-09
CNAG_05661	993.7504	0.190643	0.081341	2.343751	0.019091	0.040695
CNAG_05662	1193.332	-2.1719	0.478425	-4.53968	5.63E-06	2.60E-05
CNAG_05664	3653.358	-0.29066	0.061137	-4.75435	1.99E-06	9.81E-06
CNAG_13206	256.8247	0.396081	0.181354	2.184022	0.028961	0.05857
CNAG_05668	116.2856	-0.79247	0.210325	-3.76785	0.000165	0.000578
CNAG_11000	3.380565	-4.11158	1.643758	-2.50133	0.012373	0.027904
